# Gold-catalyzed (4+3)-annulations of 2-alkenyl-1-alkynylbenzenes with anthranils with alkyne-dependent chemoselectivity: skeletal rearrangement *versus* non-rearrangement†
†Electronic supplementary information (ESI) available. CCDC 1853703–1853706. For ESI and crystallographic data in CIF or other electronic format see DOI: 10.1039/c8sc03619e


**DOI:** 10.1039/c8sc03619e

**Published:** 2018-11-12

**Authors:** RahulKumar Rajmani Singh, Manisha Skaria, Liang-Yu Chen, Mu-Jeng Cheng, Rai-Shung Liu

**Affiliations:** a Frontier Research Centers on Fundamental and Applied Science of Materials , Department of Chemistry , National Tsing-Hua University , Hsinchu , Taiwan , Republic of China . Email: rsliu@mx.nthu.edu.tw; b Department of Chemistry , National Cheng Kung University , Tainan 701 , Taiwan . Email: mjcheng@mail.ncku.edu.tw

## Abstract

Two distinct (4+3)-nitroxy annulations between 1,5-enynes and anthranils have been developed to access tetrahydro-1*H*-benzo[*b*]azepine derivatives; the chemoselectivity varies with the types of alkynes. Terminal alkyne substrates deliver benzo[*b*]azepine derivatives *via* a novel skeletal rearrangement while internal 1,5-enynes afford products without a rearrangement process. To elucidate the mechanism of rearrangement, we performed ^13^C- and ^2^H-labeling experiments to identify the gold-containing isobenzofulvene intermediates, but their formation relies on the presence of anthranils.

## Abstract 2

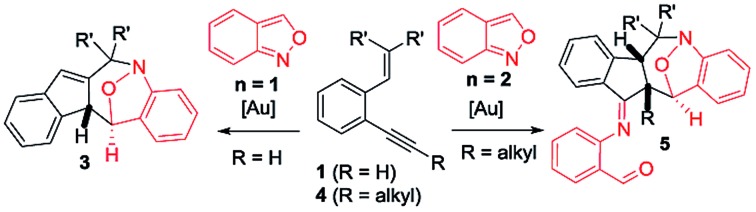
Two distinct (4+3)-nitroxy annulations between 1,5-enynes and anthranils have been developed to access tetrahydro-1*H*-benzo[*b*]azepine derivatives.

## Introduction

Cyclic nitroxy species (N–O) are widespread functionalities in numerous bioactive molecules and natural products.^1^ Tetrahydro-1*H*-benzo[*b*]azepines bearing a hydroxyl (**I–IV**) represent a family of privileged seven-membered azacycles,^2^ possessing potent activities in antiparasitic disease, antidiuretic hormone receptors and β_2_ adrenergic agonists.^3^ Synthetic procedures for compounds **I–IV** are generally long and tedious.^2^ A short route to construct tetrahydrobenzo[*b*]azepine cores involves the development of stereoselective (4+3)-annulations between anthranils and all-carbon 1,3-dipoles (eqn (1)), but only donor–acceptor cyclopropanes were shown to be applicable substrates.^4^ We are aware of no π-bond motifs that can serve as effective 1,3-dipoles.^5^

**Fig. 1:**
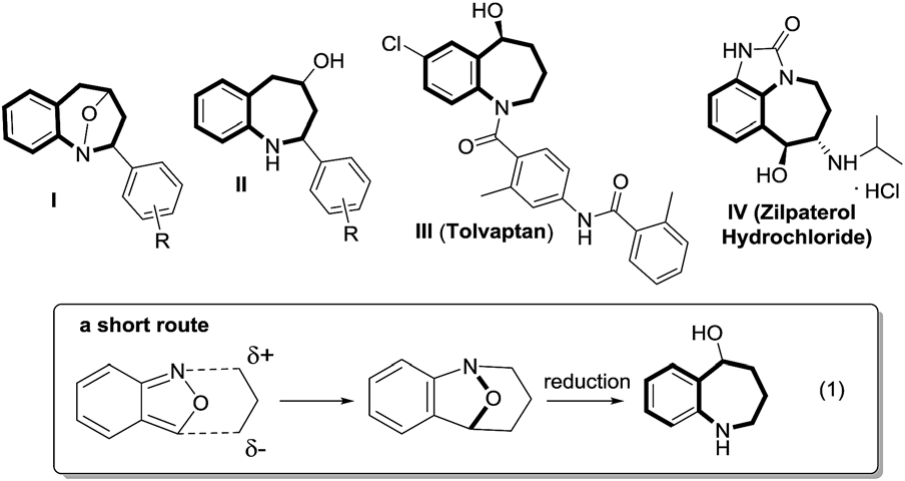
Representative molecules and a postulated short route.

Synthetic interest in isoxazoles and anthranils is rapidly growing in Au- and Pt-catalysis because of their various annulations with alkynes.^6^,^7^ Nevertheless, these hetero-aromatics serve as nucleophiles that attack π-alkynes *via* a N- or O-attack route, inevitably cleaving the N–O bonds; selected examples are provided in eqn (2) and (3). We sought the first (4+3)-nitroxy annulations using alkyne-based 1,3-dipoles and anthranils. This work reports two distinct (4+3)-annulations of 1,5-enynes with anthranils; interestingly, the chemoselectivity varies with the alkynes. Terminal 1,5-enynes **1** (R = H) afford seven-membered nitroxy heterocycles **3***via* an unprecedented rearrangement in gold catalysis;^8^ the mechanism of this novel rearrangement has been elucidated. Annulation products **5** derived from internal alkynes **4** are not skeletally rearranged, but are elaborated into various benzo[*b*]azepine frameworks (Fig. 1).

Annulations with N–O cleavages
2

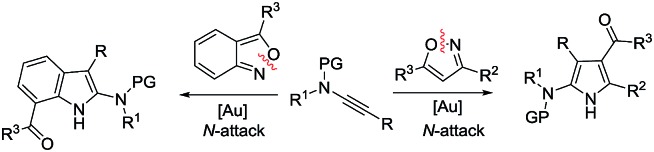



3

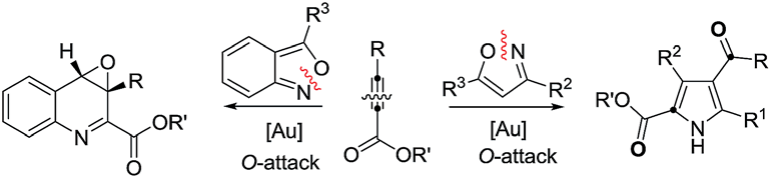




This work: (4+3)-nitroxy annulations
4

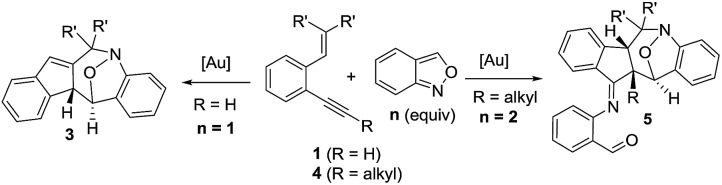




## Results and discussion

We optimized the reactions of terminal 1,5-enyne **1a** with anthranil **2a** (1.2 equiv.) using various gold catalysts; the results are shown in Table 1. Operations in dry dichloroethane (DCE, 25 °C) with L′AuCl/AgNTf_2_ (L′ = P(*t*-Bu)_2_(*o*-biphenyl), IPr, PPh_3_) afforded seven-membered nitroxy product **3a** in 8–68% yield (entries 1–3), with P(*t*-Bu)_2_(*o*-biphenyl)AuCl/AgNTf_2_ being the most effective. To our delight, (PhO)_3_PAuCl/AgNTf_2_ increased the yield of the desired **3a** up to 73% (entry 4); different silver salts as those in (PhO)_3_AuCl/AgX (X = SbF_6_ and OTf) delivered compound **3a** in relatively low yields (35–42%, entries 5 and 6). With (PhO)_3_PAuCl/AgNTf_2_, the yields of compound **3a** in different solvents were as follows: DCM (62%), acetonitrile (30%) and MeNO_2_ (0%, entries 7–9). AgNTf_2_ alone was completely inactive (entry 10). The molecular structure of compound **3a** was characterized by X-ray diffraction^9^ to reveal a (4+3)-annulation with an intact N–O bond. In the absence of anthranil **2a**, 1,5-enyne **1a** was isomerized by a gold catalyst to afford 1′-methylvinyl-1*H*-indene **1a′**, which was structurally unrelated to our target **3a**. Anthranil **2a** is obviously indispensable to enabling the (4+3)-annulations with structural rearrangement.

**Table 1:** Optimized conditions over various gold catalysts

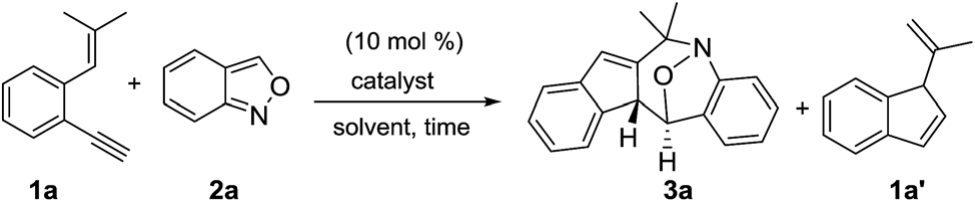								
Entry	Catalyst^*a*^ (mol %)	**2a** *n* equiv.	Solvent	Time (h)	Temp (*t* °C)	Yields^*b*^ (%)		
						**1a**	**3a**	**1a′**
1	LAuCl/AgNTf_2_	1.2	DCE	5	25	—	68	—
2	IPrAuCl/AgNtf_2_	1.2	DCE	15	25	25	8	—
3	Ph_3_PAuCl/AgNtf_2_	1.2	DCE	12	25	—	35	—
4	(PhO)_3_PAuCl/AgNtf_2_	1.2	DCE	4	25	—	73	—
5	(PhO)_3_PAuCl/AgSbF_6_	1.2	DCE	10	25	10	35	—
6	(PhO)_3_PAuCl/AgOTf	1.2	DCE	2	60	—	42	—
7	(PhO)_3_PAuCl/AgNtf_2_	1.2	DCE	10	25	—	62	—
8	(PhO)_3_PAuCl/AgNtf_2_	1.2	MeCN	10	25	—	30	—
9	(PhO)_3_PAuCl/AgNtf_2_	1.2	MeNO_2_	20	25	80	—	—
10	AgNtf_2_	1.2	DCE	24	25	85	>5	—
11	(PhO)_3_PAuCl/AgNtf_2_	0	DCE	4	25	—	—	65

^*a*^ **1a** (0.20 M), **2a** (1.2 equiv.).

^*b*^Product yields are given after purification on a silica gel column, L = P(*t*-Bu)_2_(*o*-biphenyl), IPr = 1,3-bis(diisopropylphenyl)imidazol-2-ylidene).

Under these optimized conditions, we assess the generality of these new annulations with various terminal 1,5-enynes and anthranils. The results are provided in Table 2; only a single diastereomeric product was obtained for all instances. In several instances, vinyl-1*H*-indene **1a′** was present as a byproduct in a minor proportion (5–15%). The annulations of anthranil **2a** (1.2 equiv.) with terminal 1,5-enynes **1b–1d** bearing various 4-phenyl substituents (X = Me, Cl, and F) proceeded smoothly to yield **3b–3d** in 68–77% yields (entries 2–4). For their 5-phenyl analogues **1e–1g**, the resulting annulation products **3e–3g** (Y = Me, Cl and F) were obtained in 65–74% yields (entries 5–7). Variations of the olefin substituents as those in species **1h–1j** (R, R = cyclopentyl, cyclohexyl and dipropyl) were still compatible with these new N–O annulations to afford compounds **3h–3j** in 55–67% yields (entries 8–10). We have also prepared a terminal alkyne such as 1-ethynyl-2-styrylbenzene **1k** that gave a recovery yield (>95%) of two reactants under the standard conditions.

**Table 2:** Reactions with terminal 1,5-enynes and anthranils

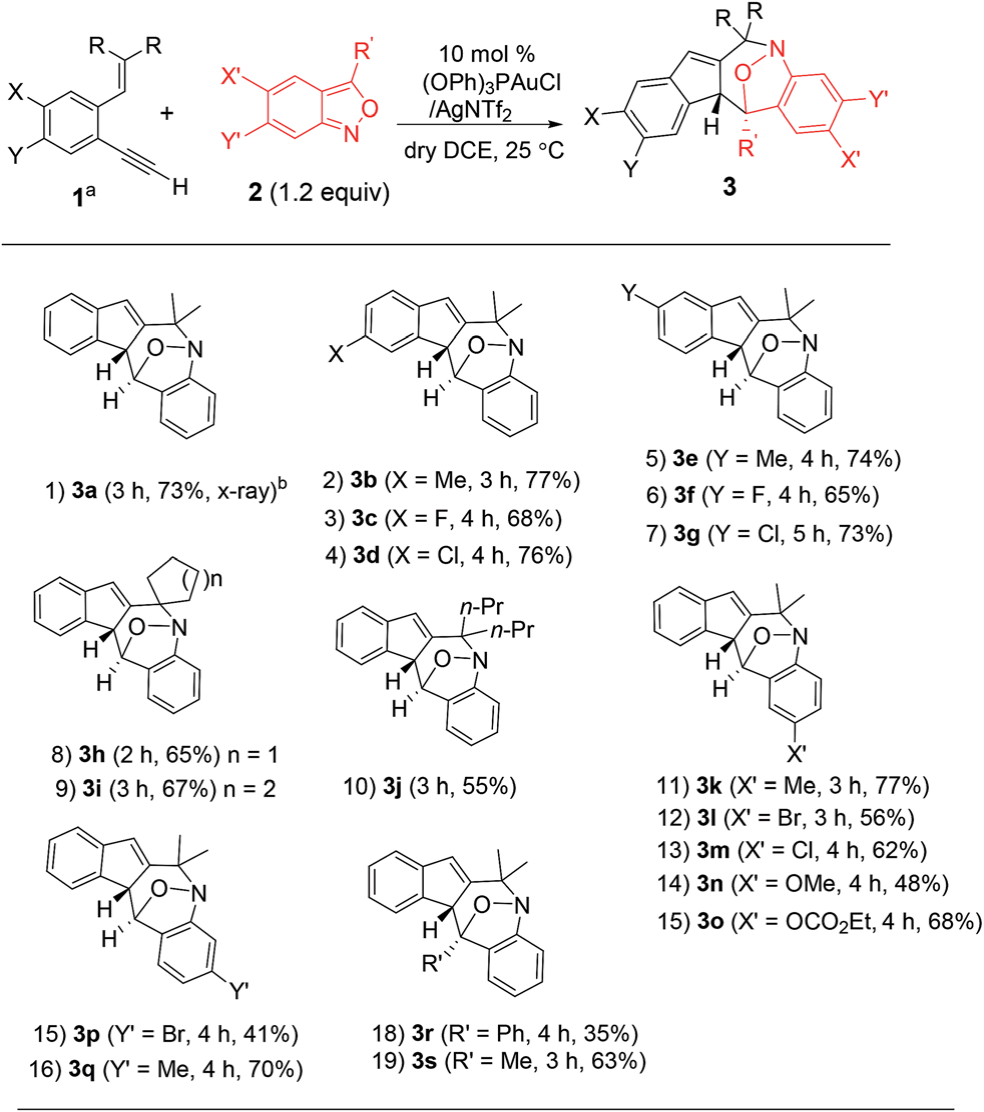

^*a*^[**1**] 0.20 M.

^*b*^Yields of the products were reported after isolation on a silica gel column.

We next examined anthranils **2b–2f** bearing various C(5)-substituents (X′ = Me, Cl, Br, OMe and OCO_2_Et), yielding cyclic nitroxy species **3k–3o** in 48–77% yields, with X′ = OMe becoming less efficient (entries 11–15). Methoxy-containing anthranil **2e** renders the gold catalyst less reactive because of its high basicity. This gold catalysis worked well with additional anthranils **2g** and **2h** bearing C(6)-substituents (Y′ = Br and Me), yielding the desired **3p** and **3q** in 41% and 70% yields, respectively (entries 15 and 16). We also varied the C(3)-substituents of anthranils (R′ = Ph **2i**; Me **2j**) to yield the desired **3r** and **3s** in 35% and 63% yields, respectively (entries 18 and 19). An effective range of alkynes and anthranils manifests the practicability of these new nitroxy annulations.

This gold-catalyzed reaction was also extensible to an internal alkyne **4a**, but led to a distinct (4+3)-annulation reaction without a skeletal rearrangement. Among various gold catalysts, P(OPh)_3_AuCl/AgSbF_6_ was superior to its NTf_2_ catalyst analogue, delivering a nitroxy product **5a** with respective yields of 78% and 68%; a molar ratio of **4a**/**2a** = 1 : 2.1 was the optimized condition. The molecular structure of **5a** was inferred from its **5b** analogue (Table 3, entry 1).^9^
5

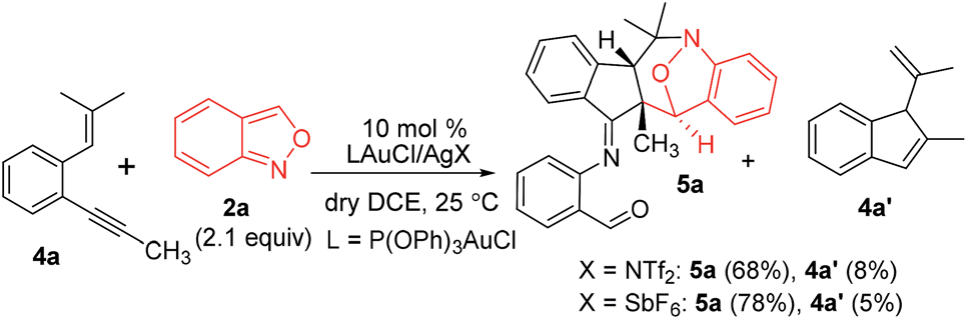




**Table 3:** Reactions with internal 1,5-enynes and anthranils

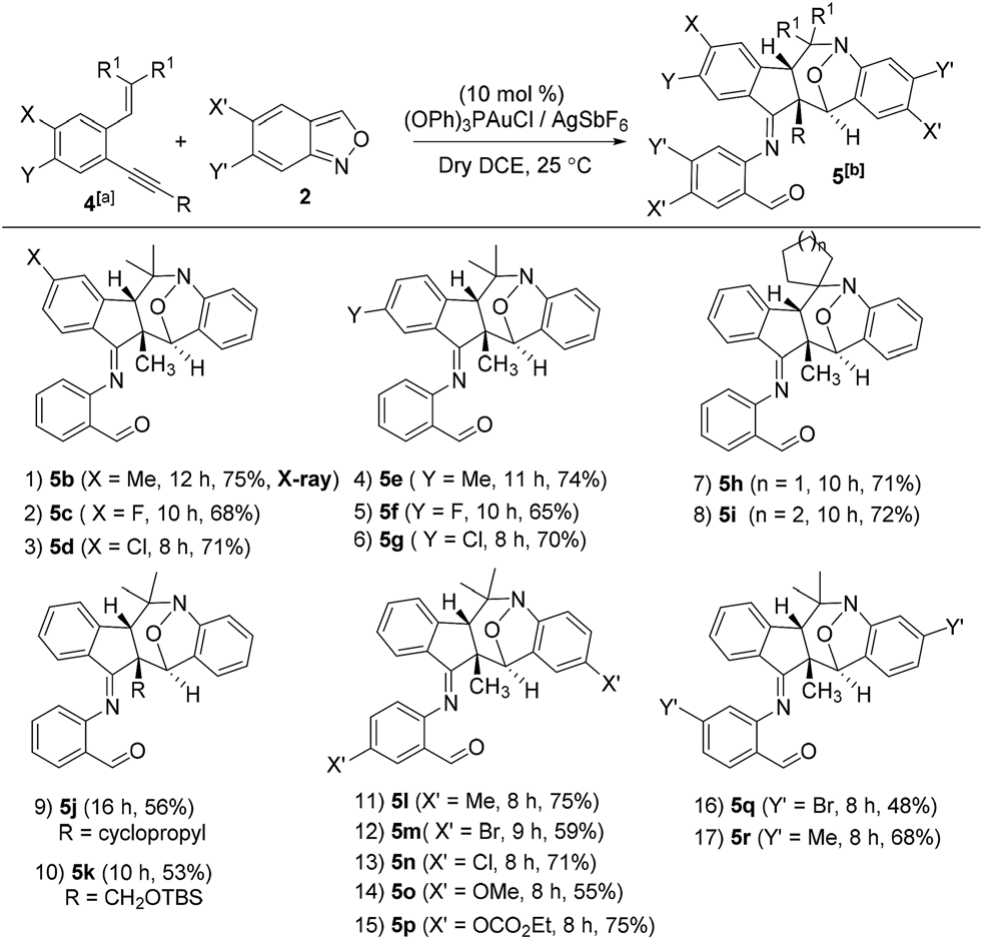

^*a*^ **4**/**2** = 1 : 2.1, [**4**] 0.20 M.

^*b*^Yields of the products were reported after isolation on a silica gel column.

We assess the scope of these nitroxy annulations with various internal 1,5-enynes **4** and anthranils **2**; only one diastereomeric product was obtained without exception. Entries 1–6 show the compatibility of these reactions with 1,5-enynes **4b–4d** and **4e–4g** bearing 4- and 5-phenyl substituents (X = Me, F and Cl or Y = Me, F and Cl), delivering compounds **5b–5d** and **5e–5g** in 65–75% yields (entries 1–6). An X-ray diffraction study^9^ confirms the molecular structure of compound **5b** showing no skeletal rearrangement. 1,5-Enynes **4h** and **4i** bearing varied trisubstituted alkenes were also suitable for the reactions, affording the desired nitroxy species **5h** and **5i** in 71–72% yields (entries 7 and 8). When the alkyl substituents R were a cyclopropyl or CH_2_OTBS group, the corresponding compounds **5j** and **5k** were obtained in 56% and 53% yields, respectively (entries 9 and 10). We tested the reactions of various anthranils **2b–2f** bearing various C(5)-substituents (X′ = Me, Br, Cl, OMe and OCO_2_Et), giving the expected products **5l–5p** in 55–75% yields with the methoxy substituent being less efficient (entries 11–15). For additional anthranils **2g** and **2h** bearing 6-substituents (Y′ = Br and Me), the resulting products **5q** and **5r** were obtained in 48% and 68% yields, respectively (entries 16 and 17).

**Scheme 1:**
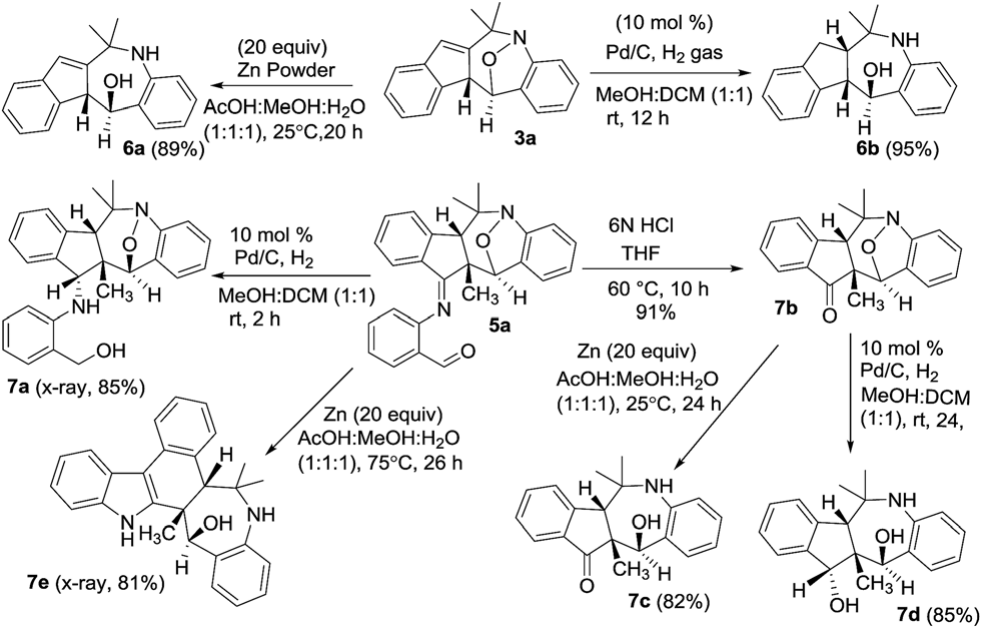
Reductive cleavage of the N–O bonds.

We performed the reductive N–O cleavage of compounds **3a** and **5a** to manifest their synthetic utility. Treatment of species **3a** with Zn in AcOH/MeOH/H_2_O^10^ gave compound **6a** in 89% yield while the reaction with Pd/H_2_ gave compound **6b** efficiently. Alternatively, compound **5a** was hydrolyzed with HCl/water to yield ketone derivative **7b** that was convertible to 1-amino-5-ol **7c** with Zn/AcOH reduction, and to the diol derivative **7d** with Pd/H_2_ reduction. An imine reduction of species **5a** was achieved with Pd/H_2_ to afford species **7a**. Unexpectedly, Zn-reduction of species **5a** in HOAc/MeOH/water led to a structural rearrangement to form compound **7e** in 81% yield. The imine moiety of the initial **5a** was incorporated into the structural skeleton of product **7e**, but the mechanism is not clear at this stage. Molecular structures of compounds **7a** and **7e** were verified by X-ray diffraction.^9^ The mechanism for the transformation of **5a** into **7e** will be elucidated in a future study (Scheme 1).

Among the two nitroxy annulations, the mechanism for terminal 1,5-enynes **1a** is difficult to deduce because its cycloisomerization product **1a′** is not skeletally rearranged. We prepared **^13^C-1a** containing 12% ^13^C at only the =C–H carbon, and its resulting product **3a** contained the ^13^C-content only at the alkyl C–H carbon (eqn (6)). Isobenzofulvene species **In 1** was unlikely to occur here although it was observed in a ruthenium-catalyzed cycloisomerization.^11^ In the presence of D_2_O, we found that the resulting **d_1_-3a** contained deuterium (*X* = 0.29D) only at its alkenyl C–H moiety (eqn (7)). Accordingly, gold-containing isobenzofulvene **In 2** is compatible with these ^13^C and ^2^H-labeling experiments.
6

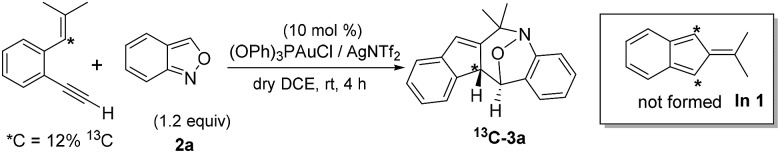



7

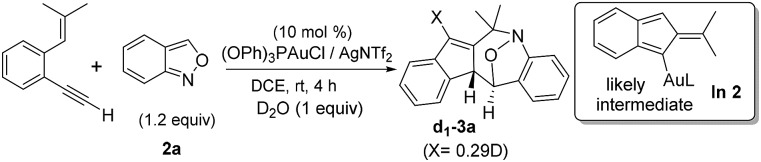




**Scheme 2:**
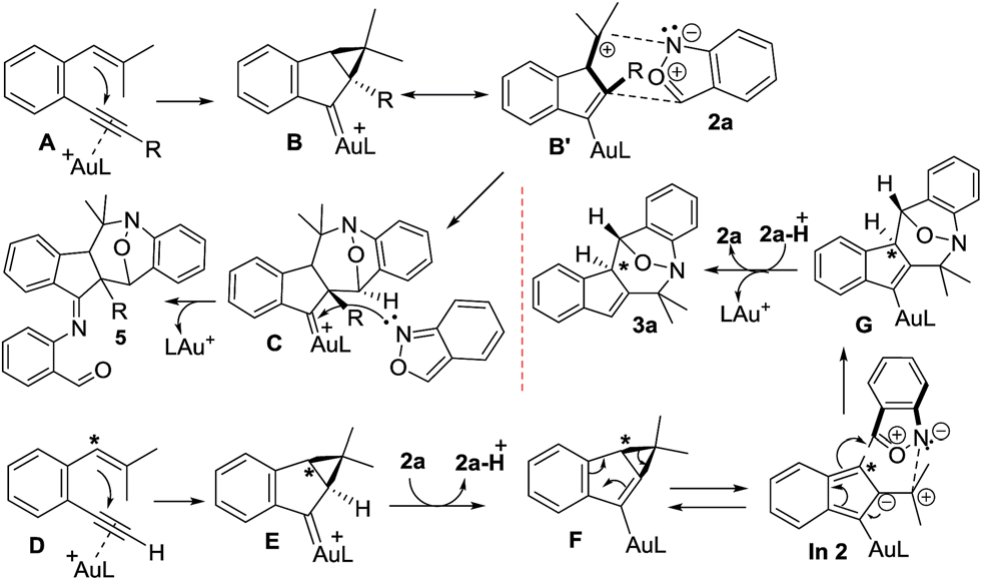
Plausible mechanisms for rearrangement and non-rearrangement.

Scheme 2 depicts the mechanisms of the two annulations. Internal 1,5-enynes **4** react with LAu^+^ to form cyclopropyl gold carbenes **B** (or **B′**) in two resonance forms; *exo*-(4+3)-annulations of species **B′** with anthranils **2a** likely yield gold-carbene species **C** that subsequently capture a second anthranil to yield products **5**. This mechanism is essentially the same as that of their annulations with nitrosoarenes.^12^ Herein, a stepwise mechanism for the annulation of anthranils with 1,3-dipoles **B**/**B′** is also likely to occur. Terminal 1,5-enyne **1a** also generates cyclopropylgold carbene **E** because its cycloisomerization product **1a′** is also a 1-vinylindene derivative. We envisage that the cyclopropyl C–H proton of gold carbene **E** is acidic because of its proximity to the gold carbene functionality; the deprotonation with anthranil **2a** generates cyclopropylidenylgold species **F** that undergoes a “methylenecyclopropane-trimethylenemethane” rearrangement,^13^ further generating gold-containing isobenzofulvene species **In 2**. An *exo*-(3+4)-annulation between fulvene **In 2** and anthranil **2a** affords the observed product **3a**. The intermediacy of organogold species **G** is supported by ^2^H and ^13^C-labeling experiments.

Density functional theory calculations were then performed to investigate the feasibility for the key steps **D** → **G**. Four possible paths 1–4 are considered; Path 1 is our proposed mechanism in Scheme 2. The energy profile is provided in Scheme 4. The formation of cyclopropylgold carbenes **E** from π-alkyne **D** has a low barrier of 9.1 kcal mol^–1^; the anion-promoted deprotonation of gold carbene **E** to form cyclopropylidenylgold species **F** is operable as the enthalpy cost is 16.9 kcal mol^–1^; the energy of species **F** is slightly higher than that of π-alkyne **D** by only 6.6 kcal mol^–1^. The remaining steps **F** → **In 2** and **In 2** → **G** are also operable as the transition states **TS-F-In2** and **TS-In2-G** are close to π-alkyne **D** energy levels. One notable feature is that the enthalpy of transition state **TS-F-ln2** is surprisingly smaller than that of species **F** by –0.3 kcal. This atypical case has similar precedents in the literature.^14^ This is because **TS-F-In2** has less zero-point vibration energy than **F**, due to the loss of one degree of freedom in the transition state. This also means that **F** → **In2** is a barrierless process.

We next examined the energy profiles in the (4+3) annulations (Path 2) between cyclopropyl gold carbenes **E** and anthranil **2a**. The reaction proceeds in a stepwise manner. As shown in Scheme 5, the N-attack of anthranil **2a** at gold carbene **E** produces species **E_step_** by an endothermic process (*H* = 13.6 kcal mol^–1^); its activation energy is as high as 25.4 kcal mol^–1^. In the next step involving **E_step_** → **GH**, the energy level of **TS-E_step_-GH** is higher than that of 1,5-enyne **D** by 18.1 kcal mol^–1^. We conclude that Path 2 is not as feasible as Path 1 according to Scheme 5.

**Scheme 3:**
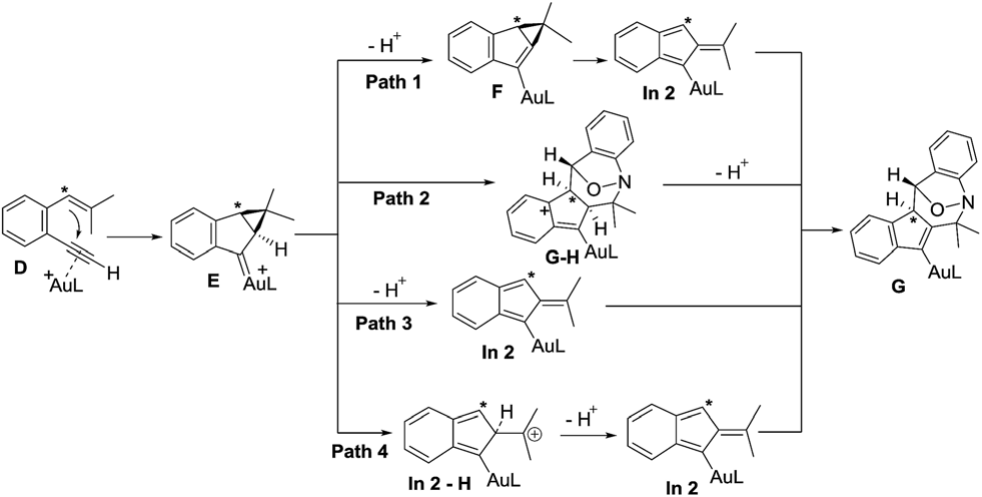
Four possible paths for the **D** → **G** transformation.

We also considered the remaining Paths 3 and 4, as depicted in Scheme 3. In Path 3, the deprotonation and ring rearrangement take place simultaneously (**E** → **In2**), in contrast to a stepwise process in Path 1 (**E** → F → **In2**). Despite multiple attempts, we were unable to locate the transition state for the direct **E** → **In2** step, suggesting that Path 3 probably does not exist. In Path 4, a ring opening takes place initially (**E** → **In2-H**), followed by deprotonation (**In2-H**→**In2**). However, our calculations show that this pathway is unlikely to occur as we are unable to locate **In2-H**; all geometry optimizations lead to **E**.

**Scheme 4:**
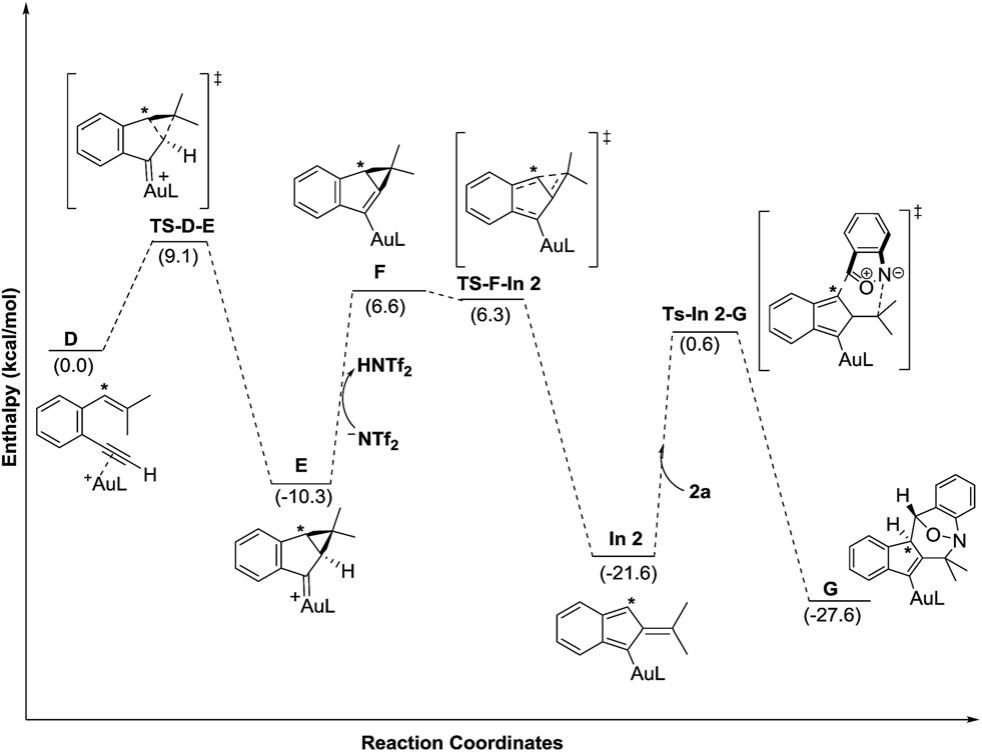
DFT calculation and energy profiles of Path 1.

**Scheme 5:**
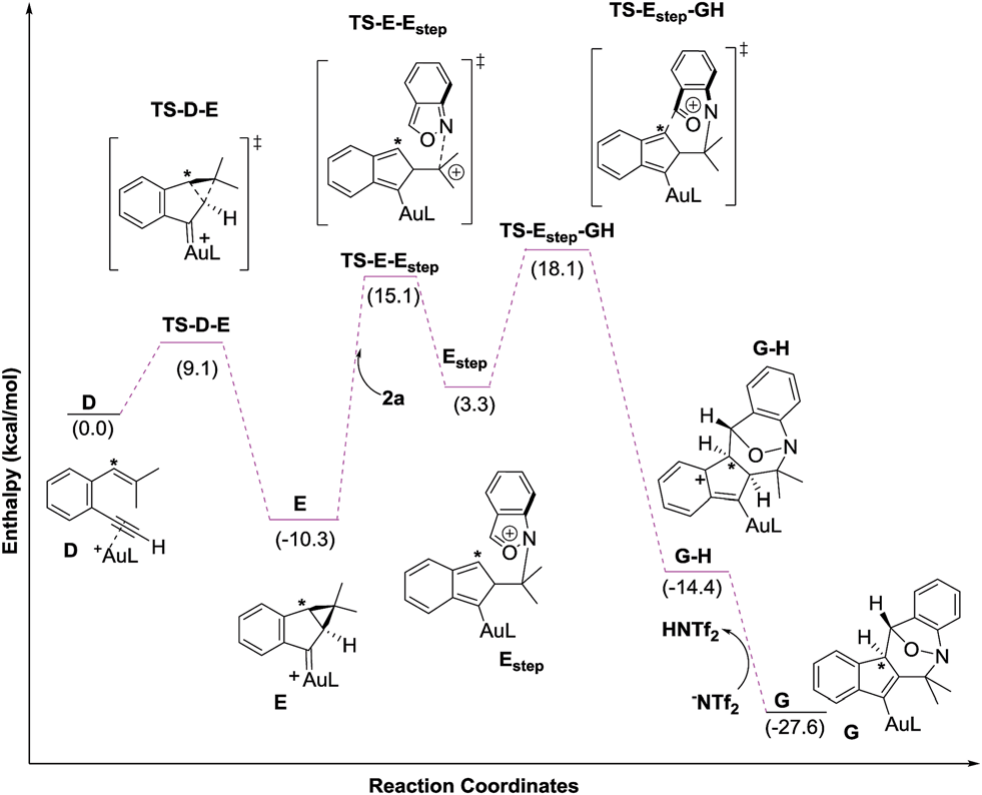
DFT calculation and energy profiles of Path 2.

## Conclusions

Before this work, Au- and Pt-catalyzed annulations of anthranils with alkynes typically produced azacyclic products that cleaved the N–O bonds. To develop new (4+3)-annulations of alkyne-derived 1,3-dipoles^15^ with anthranils, we achieve stereoselective synthesis of two classes of tetrahydrobenzo[*b*]azepines using 1,5-enynes, anthranils and a gold catalyst. Internal 1,5-enynes deliver these cyclic nitroxy species without skeletal rearrangement while their terminal alkyne analogues afford distinct annulation products with skeletal rearrangement. To elucidate the mechanism of this rearrangement, ^2^H and ^13^C-labeling experiments were performed to identify the intermediates of gold-containing isobenzofulvene species, the formation of which is dependent on the presence of anthranils.

## Conflicts of interest

There are no conflicts of interest to declare.

## Supplementary Material

Supplementary information

Crystal structure data
