# Supplementary material for: Gold-catalyzed (4+3)-annulations of 2-alkenyl-1-alkynylbenzenes with anthranils with alkyne-dependent chemoselectivity: skeletal rearrangement versus non-rearrangement
Source: Chem Sci. 2018 Nov 12;10(4):1201–6. doi: 10.1039/c8sc03619e (PMC6349068; doi:10.1039/c8sc03619e)
Supplement: Supplementary file 1 [file SC-010-C8SC03619E-s001.pdf]

Electronic Supplementary Material (ESI) for Chemical Science.
This journal is © The Royal Society of Chemistry 2018

Supporting Information

Gold-catalyzed [4+3]-Annulations of 2-Alkenyl-1-Alkynylbenzenes with Anthranils with Alkyne-Dependent Chemoselectivity: Skeletal Rearrangement versus Non-Rearrangement

RahulKumar Rajmani Singh[a], Manisha Skaria[a], Liang-Yu Chen[b], Mu-Jeng Cheng*[b] and Rai-Shung Liu*[a]

[a]Frontier Research Center for Matter Science and Technology and Department of Chemistry, National Tsing-Hua University, Hsinchu, Taiwan, ROC--------------
Email : rsliu@mx.nthu.edu.tw
[b]Department of Chemistry, National Cheng Kung University, Tainan 701, Taiwan,

Table of Contents:

(I) General synthetic procedures for of 1,5-enynes ----------------------------1

(II) Procedures for catalytic operations--2

(III) Computational details--3

(IV) Spectral data for new compounds ---5

(V) X-ray crystallographic data for compound 3a, 5b, 7a and 7e----------28

(VI) Coordinates for the Optimized Structures ------------------------------70

(VII) ^{1}H & [13]C Spectra --92

(I) General Synthetic Procedures:

(a) General procedure:

Unless otherwise noted, preparations of the substrates were performed in oven-dried glassware under nitrogen atmosphere with freshly distilled solvents. The catalytic reactions were performed under nitrogen atmosphere. DCE and DCM were distilled from CaH_2 under nitrogen. THF were distilled from Na metal under nitrogen. All other commercial reagents were used without further purification, unless otherwise indicated. ^{1}H NMR and ^{13}C NMR spectra were recorded on a Varian 400 MHz, Bruker 400 and 600 MHz Spectrometers using chloroform-*d* ($CDCl_3$) and d-Acetone as the internal standards.

All 1, 5-enynes **1a**, **1c**, **1d**, **1f, 1h** and **4a**, **4d**, **4g-4h** were prepared from the reported procedure in the literature. [S1]. All benzisoxazoles (**2a-2h**) were prepared according to literature procedure.[S2]

[S1] a) R. J. Madhushaw, C. -Y. Lo, C. -W. Hwang, M. -D. Su, H. -C. Shen, S. Pal, I. R. Shaikh, R.-S. Liu, *J. Am. Chem. Soc.*, 2004, **126**, 15560 - 15565. b) C.-H. Chen, Y. -C. Tsai, R. -S. Liu, *Angew. Chem. Int. Ed.* 2013, **52**, 4599 –4603

[S2] a)Sahani, R. L.; Liu, R.-S. *Angew. Chem. Int. Ed.* 2017, **56**, 1026; b) Chauhana, J.; Fletcher, S. *Tetrahedron Lett.* 2012, **53**, 4951.

(b) Preparation of 1-(2-methylprop-1-en-1-yl)-2-(prop-1-yn-1-yl)benzene (4a).

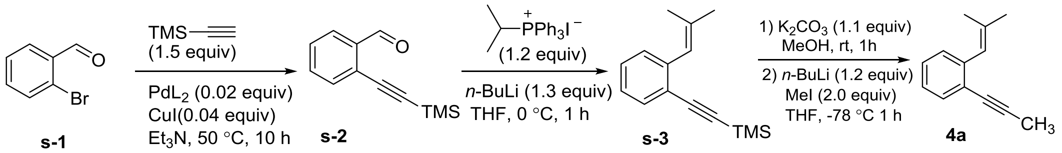

To a triethylamine (40 ml) solution of 2-bromobenzaldehyde **(s-1)** (1.90 g, 10.3 mmol), $PdCl_2(PPh_3)_2$ (143.8 mg, 0.2 mmol), and CuI (78.2 mg, 0.4 mmol) was added the trimethylsilylacetylene (1.5 g, 15.3 mmol). The resulting mixture was heated at 50 °C for 8 h under N_2 atmosphere. The reaction mixture was treated with water, and extracted with CH_2Cl_2 (three times). The organic layer was washed with brine, dried over $MgSO_4$, and concentrated in vacuum. The residue was purified on a silica column to afford 2-((trimethylsilyl)ethynyl)benzaldehyde **(s-2)** as a yellowish solid (1.7 g, 8.5 mmol, 82 %).

To a THF solution (25 mL) of isopropyltriphenylphosphonium iodide (4.8 g, 11.1 mmol) was added *n*-BuLi (2.5 M, 3.6 mL, 8.9 mmol) at 0 ^{o}C, and the mixture was

stirred for 30 min at same temperature before 2-((trimethylsilyl)ethynyl)benzaldehyde **(s-2)** (1.5 g, 7.4 mmol) was added. The solution was slowly warmed to room temperature and stirred for 1 h at 25 ^oC before it was quenched with aqueous NH_4Cl solution. The organic layer was extracted with diethyl ether, dried over $MgSO_4$ and chromatographed on a silica column to give trimethyl ((2-(2-methylprop-1-en-1-yl) phenyl)ethynyl)silane **(s-3)** (1.5 g, 6.6 mmol, 89 %) as a yellow oil.

To a MeOH solution (15 mL) of trimethyl((2-(2-methylprop-1-en-1-yl)phenyl) ethynyl)silane **(s-3)** (1.5 g, 6.6 mmol) was added K_2CO_3 (1.0 g, 7.2 mmol) at 25 ^oC; the resulting solution was stirred at room temperature for 1 h before it was quenched with distilled water. The organic layer was extracted with diethyl ether, dried over $MgSO_4$ and chromatographed on a silica column to give 1-ethynyl-2-(2-methylprop-1 -en-1-yl)benzene (1.0 g, 6.4 mmol, 97 %).

To a dry THF solution (22.5 mL) of 1-ethynyl-2-(2-methylprop-1-en-1-yl)benzene (1.0 g, 6.4 mmol) was added *n*-BuLi (2.5 M, 2.8 mL, 7.0 mmol) at -78 ^oC, and the mixture was stirred for 30 min before MeI (1.8 g, 12.8 mmol) was added at -78 ^oC. The resulting solution was slowly warmed to room temperature and stirred for 2 h. The reaction was quenched with distilled water and extracted with diethyl ether (30 mL * 2 times); the organic layer was combined and dried over $MgSO_4$ and chromategraphed on a silica column to give 1-(2-methylprop-1-en-1-yl)-2-(prop-1-yn-1-yl) benzene **(4a)** (1.1 g, 99 %).

(II) Standard procedures for catalytic operations:

(a) Typical procedure for the synthesis of (3a).

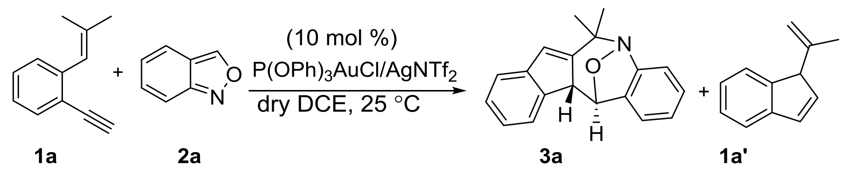

A 1,2-dichloroethane (1.0 mL) solution of $(OPh)_3PAuCl$ (35 mg, 0.064 mmol) and $AgNTf_2$ (25.0 mg, 0.064 mmol) was stirred at 25 ^oC for 5 min; to this DCE solution was added 1-ethynyl-2-(2-methylprop-1-en-1-yl)benzene **(1a)** (100 mg, 0.64 mmol) and anthranils (**2a**) (84 mg, 0.71 mmol, 1.1 equiv) over a period of 5 min at 25 ^oC; the solution was stirred for 4 h at the same temperature before it was filtered over a celite bed. The solvent was evaporated under reduced pressure, and the residue was purified on a flash silica gel column to give compound **3a** (128.7 mg, 73 % yield, 0.46 mmol)

as a yellow solid.

(b) Typical procedure for the synthesis of (5a).

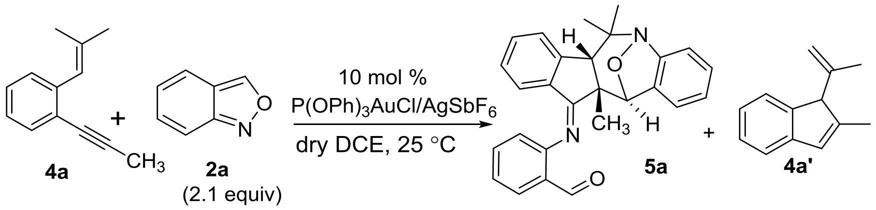

A 1,2-dichloroethane (1.0 mL) solution of $(OPh)_3PAuCl$ (24.0 mg, 0.059 mmol) and $AgSbF_6$ (15.0 mg, 0.059 mmol) was stirred at 25 °C for 5 min; to this DCE solution of 1-(2-methylprop-1-en-1-yl)-2-(prop-1-yn-1-yl)benzenee **(4a)** (100 mg, 0.59 mmol) and anthranils (**2a**) (147 mg, 1.2 mmol, 2.1 equiv) was slowly added over a period of 5 min at 25 °C. The solution was stirred for 10 h at the same temperature before filteration over a celite bed. The solvent was evaporated under reduced pressure; the residues were purified on a silica column to give compound **5a** (187.4 mg, 0.46 mmol, 78%) as a yellow solid and **4a'** [S3] (5.0 mg, 0.29 mmol, 5 %).

[S3] C.-H. Chen, Y. -C. Tsai, R. -S. Liu, *Angew. Chem. Int. Ed*. 2013, **52**, 4599 – 4603.

(III) Computational details:

The geometry optimizations and zero-point vibrational energy (ZPVE) were carried out using the B3LYP functional[S4-S6] with the 6-31G** basis set[S7-S8] for all atoms except Au. For Au the first four shells of core electrons were described by the Los Alamos angular momentum projected effective core potential (ECP) using the double-ζ contraction of valence functions (denoted as LACVP**) leading to 19 explict electons for neural Au.[S9] In order to obtain a more accurate electronic energy, we performed single-point energy calculations based on the same functional, but using a larger basis set, where Au was described with the triple-ζ contraction of valence functions (the core electrons were described by the same ECP), with the other atoms described with the 6-311++G** basis set. Solvation energies G_{solv} were calculated using the Poisson-Boltzmann self-consistent polarizable continuum method[S10-S11] implemented in *Jaguar* to represent dichloroethane (dielectric constant = 10.65 and effective radius = 2.51 Å). The solvation calculations used the B3LYP/LACVP** level of theory and the gas-phase optimized structures. All energies discussed in this work are $H(298\ K) = E_{SCF} + G_{solv} + ZPVE + H_{trans} + H_{rot} + H_{vib} + PV$, where E_{SCF} is electronic energy, and H_{trans}, H_{rot}, and H_{vib} are translational, rotational, and vibrational thermal corrections, respectively.

[S4] A. D. Becke, Density-Functional Exchange-Energy Approximation with Correct Asymptotic-Behavior. *Phys Rev A* 1988, **38**, 3098-3100.

[S5] A. D. Becke, Density-Functional Thermochemistry .3. The Role of Exact Exchange. *J. Chem. Phys.* 1993, **98**, 5648-5652.

[S6] C. T. Lee, W. T. Yang, R. G. Parr, Development of the Colle-Salvetti Correlation-Energy Formula into a Functional of the Electron-Density. *Phys Rev B* 1988, **37**, 785-789.

[S7] M. M. Francl, W. J. Pietro, W. J. Hehre, J. S. Binkley, M. S. Gordon, D. J. Defrees, J. A. Pople, Self-Consistent Molecular-Orbital Methods .23. A Polarization-Type Basis Set for 2nd-Row Elements. *J. Chem. Phys.* 1982, **77**, 3654-3665.

[S8] W. J. Hehre, R. Ditchfie, J. A. Pople, Self-Consistent Molecular-Orbital Methods .12. Further Extensions of Gaussian-Type Basis Sets for Use in Molecular-Orbital Studies of Organic-Molecules. *J. Chem. Phys.* 1972, **56**, 2257-2261.

[S9] P. J. Hay, W. R. Wadt, Abinitio Effective Core Potentials for Molecular Calculations - Potentials for K to Au Including the Outermost Core Orbitals. *J. Chem. Phys.* 1985, **82**, 299-310.

[S10] B. Marten, K. Kim, C. Cortis, R. A. Friesner, R. B. Murphy, M. N. Ringnalda, D. Sitkoff, B. Honig, New Model for Calculation of Solvation Free Energies: Correction of Self-Consistent Reaction Field Continuum Dielectric Theory for Short-Range Hydrogen-Bonding Effects. *J. Phys. Chem.* 1996, **100**, 11775-11788.

[S11] D. J. Tannor, , B. Marten, R. Murphy, R. A. Friesner, D. Sitkoff, A. Nicholls, M. Ringnalda, W. A. Goddard, B. Honig, Accurate First Principles Calculation of Molecular Charge-Distributions and Solvation Energies from Ab-Initio Quantum-Mechanics and Continuum Dielectric Theory. *J. Am. Chem. Soc.* 1994, **116**, 11875-11882.

(IV) Spectral data of key compound:

Spectral data for 1-ethynyl-4-methyl-2-(2-methylprop-1-en-1-yl)benzene (1b).

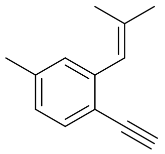

1b

Colorless liquid, ^{1}H NMR (400 MHz, $CDCl_3$): δ 7.32 (s, 1H), 7.16 (d, J = 8.0 Hz, 1H), 7.10 (d, J = 8.0 Hz, 1H), 6.47 (s, 1H), 3.23 (s, 1H), 2.30 (s, 3H), 1.94 (s, 3H), 1.56 (s, 3H); ^{13}C NMR (100 MHz, $CDCl_3$): δ 138.2, 136.3, 135.4, 133.2, 129.2, 129.0, 123.4, 121.2, 82.9, 80.6, 26.6, 20.8, 19.6.

Spectral data for 2-ethynyl-4-methyl-1-(2-methylprop-1-en-1-yl)benzene (1e).

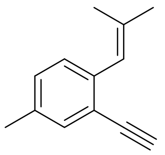

1e

Colorless liquid, ^{1}H NMR (400 MHz, $CDCl_3$): δ 7.36 (s, 1H), 7.16 (d, J = 48.0 Hz, 1H), 7.13 (d, J = 20.0 Hz, 1H), 6.47 (s, 1H), 3.23 (s, 1H), 2.32 (s, 3H), 1.94 (s, 3H), 1.55 (s, 3H); ^{13}C NMR (100 MHz, $CDCl_3$): δ 138.4, 136.3, 135.4, 133.3, 129.2, 129.0, 123.4, 121.4, 82.9, 80.6, 26.6, 20.8, 19.6.

Spectral data for 4-chloro-2-ethynyl-1-(2-methylprop-1-en-1-yl)benzene (1g).

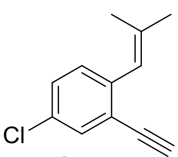

1g

Light yellow color liquid, ^{1}H NMR (400 MHz, $CDCl_3$): δ 7.40 (d, J = 8.4 Hz, 1H), 7.25 (s, 1H), 7.13 (d, J = 8.4 Hz, 1H), 6.43 (s, 1H), 3.28 (s, 1H), 1.94 (s, 3H), 1.82 (s, 3H); ^{13}C NMR (100 MHz, $CDCl_3$): δ 142.6, 138.2, 134.1, 133.8, 129.0, 126.0, 122.6, 119.9, 81.9, 81.7, 26.6, 19.5.

Spectral data for 1-ethynyl-2-(2-propylpent-1-en-1-yl)benzene (1j).

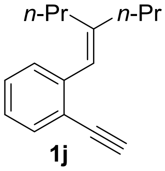

1j

Colorless liquid, ^{1}H NMR (400 MHz, $CDCl_3$): δ 7.47 (d, J = 8.0 Hz, 1H), 7.28 (t, J = 7.6 Hz, 1H), 7.21 (d, J = 6.8 Hz, 1H), 7.14 (t, J = 6.8 Hz, 1H), 6.44 (s, 1H), 3.22 (s, 1H), 2.18~2.09 (m, 4H), 1.59~1.51 (m, 2H), 1.49~1.42 (m, 2H), 0.96 (t, J = 7.2 Hz, 3H), 0.84 (t, J = 7.6 Hz, 3H); ^{13}C NMR (100 MHz, $CDCl_3$): δ 144.4, 141.5, 132.7, 128.9, 128.3, 125.8, 123.7, 121.5, 82.8, 80.9, 38.8, 32.8, 21.4, 21.2, 14.1, 13.9.

Spectral data for 1-(prop-1-en-2-yl)-1*H*-indene (1a').

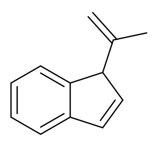

1a'

Colorless liquid, ^{1}H NMR (400 MHz, $CDCl_3$): δ 7.35 (d, J = 7.6 Hz, 2H), 7.25 (d, J = 7.2 Hz, 1H), 7.18 (t, J = 7.2 Hz, 1H), 6.84 (dd, J = 7.0, 2.0 Hz, 1H), 6.41 (dd, J = 5.2, 2.0 Hz, 1H), 5.01 (d, J = 56.0 Hz, 2H), 4.10 (s, 1H), 1.35 (s, 3H); ^{13}C NMR (100 MHz, $CDCl_3$): δ 145.9, 144.6, 143.8, 138.3, 132.0, 126.7, 125.0, 123.4, 120.9, 113.3, 58.8, 18.2. ESI-MS calcd. for $C_{12}H_{12}$: 156.0939, found 157.0962 [M+H].

Spectral data for 4-methyl-2-(2-methylprop-1-en-1-yl)-1-(prop-1-yn-1-yl)benzene (4b).

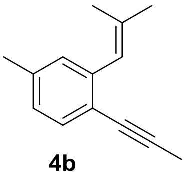

4b

Colorless liquid, ^{1}H NMR (400 MHz, $CDCl_3$): δ 7.36 (t, J = 7.2 Hz, 1H), 7.11 (d, J = 4.8 Hz, 1H), 7.00~6.97 (m, 1H), 6.54 (d, J = 5.2 Hz, 1H), 2.37 (s, 3H), 2.11 (s, 3H), 1.99 (s, 3H), 1.89 (s, 3H); ^{13}C NMR (100 MHz, $CDCl_3$): δ 140.0, 136.6, 135.7, 132.0, 129.6, 126.5, 124.1, 120.3, 88.7, 78.8, 26.6, 21.3, 19.5, 4.3.

Spectral data for 4-fluoro-2-(2-methylprop-1-en-1-yl)-1-(prop-1-yn-1-yl)benzene (4c).

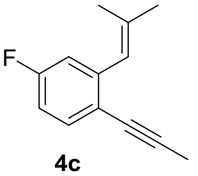

4c

Colorless liquid, ^{1}H NMR (400 MHz, $CDCl_3$): δ 7.15 (dd, J = 8.4, 6.4 Hz, 1H), 7.07 (d, J = 9.6 Hz, 1H), 6.92 (t, J = 10.0 Hz, 1H), 6.37 (s, 1H), 2.08 (s, 3H), 1.92 (s, 3H), 1.77 (s, 3H); ^{13}C NMR (100 MHz, $CDCl_3$): δ 160.5 (d, J = 253 Hz), 136.4, 136.2,

130.3 (d, J = 6 Hz), 124.9 (d, J = 10 Hz), 123.1, 118.5 (d, J = 21 Hz), 114.2 (d, J = 21 Hz), 90.8, 77.8, 26.5, 19.4, 4.3.

Spectral data for 4-methyl-1-(2-methylprop-1-en-1-yl)-2-(prop-1-yn-1-yl)benzene (4e).

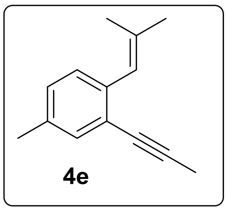

Colorless liquid, ^{1}H NMR (400 MHz, CDCl$_3$): δ 7.26 (s, 1H), 7.16 (d, J = 8.0 Hz, 1H), 7.05 (d, J = 8.0 Hz, 1H), 6.49 (s, 1H), 2.31 (s, 3H), 2.10 (s, 3H), 1.96 (s, 3H), 1.85 (s, 3H); ^{13}C NMR (100 MHz, CDCl$_3$): δ 137.3, 135.4, 135.2, 132.7, 128.8, 127.9, 123.9, 123.1, 89.3, 78.9, 26.7, 20.7, 19.5, 4.5

Spectral data for 4-fluoro-1-(2-methylprop-1-en-1-yl)-2-(prop-1-yn-1-yl)benzene (4f).

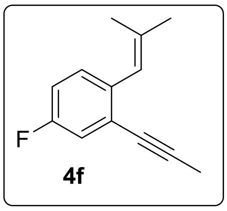

Colorless liquid, ^{1}H NMR (400 MHz, CDCl$_3$): δ 7.16 (dd, J = 8.4, 5.6 Hz, 1H), 7.08 (dd, J = 8.8, 2. Hz, 1H), 6.91 (t, J = 8.8 Hz, 1H), 6.38 (s, 1H), 2.07 (s, 3H), 1.92 (s, 3H), 1.78 (s, 3H); ^{13}C NMR (100 MHz, CDCl$_3$): δ 160.5 (d, J = 240 Hz), 136.4, 136.1, 130.4 (d, J = 8 Hz), 124.9 (d, J = 10 Hz), 123.1, 118.6 (d, J = 22 Hz), 114.3 (d, J = 21 Hz), 90.9, 77.9, 26.6, 19.4, 4.4.

Spectral data for tert-butyldimethyl((3-(2-(2-methylprop-1-en-1-yl)phenyl)prop -2-yn-1-yl)oxy)silane (4k).

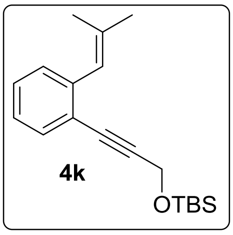

Light yellow color liquid, ^{1}H NMR (400 MHz, CDCl$_3$): δ 7.43 (d, J = 7.6 Hz, 1H), 7.26~7.24 (m, 2H), 7.15~7.11 (m, 1H), 6.48 (s, 1H), 4.58 (s, 2H), 1.93 (d, J = 1.4 Hz, 3H), 1.81 (d, J = 1.3 Hz, 3H), 0.95 (s, 9H), 0.18 (s, 6H); ^{13}C NMR (100 MHz, CDCl$_3$): δ 140.5, 136.4, 132.3, 129.0, 127.7, 125.7, 123.9, 122.2, 91.8, 83.9, 52.3, 26.6, 25.8,

19.5, 18.3.

Spectral data for (11b*R*,12*R*)-6,6-dimethyl-11b,12-dihydro-6*H*-5,12-epoxybenzo [*b*]indeno[1,2-*e*]azepine (3a).

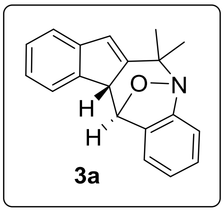

Yellow solid, mp: 171-172 °C; ^{1}H NMR (400 MHz, $CDCl_3$): δ 7.52 (d, *J* = 6.0 Hz, 1H), 7.20~7.18 (m, 2H), 7.12~7.10 (m, 1H), 7.02 (t, *J* = 7.6 Hz, 1H), 6.97 (d, *J* = 7.6 Hz, 1H), 6.72 (t, *J* = 7.2 Hz, 1H), 6.41 (d, *J* = 7.2 Hz, 1H), 6.17 (s, 1H), 5.69 (d, *J* = 4.8 Hz, 1H), 4.11 (d, *J* = 4.8 Hz, 1H), 1.81 (s, 3H), 1.42 (s, 3H); ^{13}C NMR (100 MHz, $CDCl_3$): δ 149.7, 149.1, 144.4, 140.5, 135.8, 127.2, 127.1, 126.7, 124.9, 124.5, 123.0, 121.0, 120.8, 116.6, 84.4, 65.5, 50.5, 27.6, 23.5; ESI-MS calcd. for $C_{19}H_{18}NO$ [M+H]: 276.1388, found: 276.1386.

Spectral data for ^{13}C Labelled (11b*R*,12*R*)-6,6-dimethyl-11b,12-dihydro-6*H*-5,12 -epoxybenzo[*b*]indeno[1,2-*e*]azepine (^{13}C-3a).

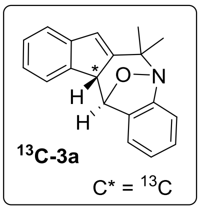

Yellow solid, mp: 178-179 °C; ^{1}H NMR (400 MHz, $CDCl_3$): δ 7.52 (d, *J* = 6.0 Hz, 1H), 7.20~7.18 (m, 2H), 7.12~7.10 (m, 1H), 7.02 (t, *J* = 7.6 Hz, 1H), 6.95 (d, *J* = 7.6 Hz, 1H), 6.72 (t, *J* = 7.2 Hz, 1H), 6.41 (d, *J* = 7.2 Hz, 1H), 6.17 (s, 1H), 5.69 (d, *J* = 4.8 Hz, 1H), 4.11 (d, *J* = 4.8 Hz, 1H), 1.80 (s, 3H), 1.42 (s, 3H); ^{13}C NMR (100 MHz, $CDCl_3$): δ 149.6, 149.1, 144.3, 140.5, 135.8, 127.2, 127.1, 126.7, 124.9, 124.5, 123.0, 121.0, 120.8, 116.6, 84.4, 65.5, 50.5 (^{13}C CH), 27.6, 23.5; ESI-MS calcd. for $C_{19}H_{18}NO$ [M+H]: 277.1388, found: 277.1381.

Spectral data for (11b*R*,12*R*)-6,6-dimethyl-11b,12-dihydro-6*H*-5,12-epoxybenzo [*b*]indeno[1,2-*e*]azepine (d_1-3a).

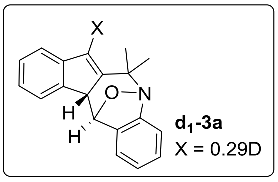

Light yellow solid, mp: 174-175 °C; ^{1}H NMR (400 MHz, $CDCl_3$): δ 7.52 (d, *J* = 6.4 Hz, 1H), 7.20~7.17 (m, 2H), 7.12~7.10 (m, 1H), 7.01 (t, *J* = 7.6 Hz, 1H), 6.94 (d, *J* = 7.6 Hz, 1H), 6.72 (t, *J* = 7.2 Hz, 1H), 6.41 (d, *J* = 7.2 Hz, 1H), 6.17 (s, 0.71H/0.29D),

5.70 (d, J = 5.2 Hz, 1H), 4.11 (d, J = 4.4 Hz, 1H), 1.80 (s, 3H), 1.41 (s, 3H); ^{13}C NMR (100 MHz, $CDCl_3$): δ 149.7, 149.1, 149.0, 144.4, 140.5, 135.8, 127.2, 127.1, 126.7, 125.0, 124.5, 123.0, 121.0, 120.8, 116.6, 84.4, 65.5, 50.5, 27.6, 23.5; ESI-MS calcd. for $C_{19}H_{17}NO$: 275.1310, found: 276.1381 [M+H] and $C_{19}H_{17}{}^{2}H_{1}NO$:277.1399 [M+H].

Spectral data for (11b*R*,12*R*)-6,6,9-trimethyl-11b,12-dihydro-6*H*-5,12-epoxyben -zo[*b*]indeno[1,2-*e*]azepine (3b).

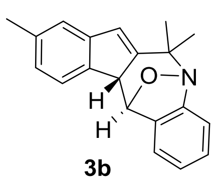

3b

Yellow solid, mp: 168-169 ^{o}C; ^{1}H NMR (400 MHz, $CDCl_3$): δ 7.38 (d, J =7.6 Hz, 1H), 7.03~6.91 (m, 4H), 6.73 (t, J = 7.2 Hz, 1H), 6.44 (d, J = 7.2 Hz, 1H), 6.11 (s, 1H), 5.66 (d, J = 5.2 Hz, 1H), 4.07 (d, J = 4.8 Hz, 1H), 2.31 (s, 3H), 1.79 (s, 3H), 1.40 (s, 3H); ^{13}C NMR (100 MHz, $CDCl_3$): δ 149.7, 149.3, 144.6, 137.6, 136.9, 135.9, 127.1, 126.7, 125.3, 124.9, 122.7, 121.7, 121.0, 116.5, 84.5, 65.5, 50.1, 27.6, 23.5, 21.5; ESI-MS calcd. for $C_{20}H_{20}NO$ [M+H]: 290.1545, found: 290.1546.

Spectral data for (11b*R*,12*R*)-9-fluoro-6,6-dimethyl-11b,12-dihydro-6*H*-5,12-epo -xybenzo[*b*]indeno[1,2-*e*]azepine (3c).

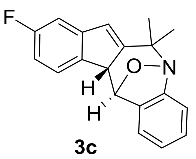

3c

Light reddish solid, mp: 163-164 ^{o}C; ^{1}H NMR (400 MHz, $CDCl_3$): δ 7.25~7.22 (m, 1H), 7.05~7.00 (m, 2H), 6.96 (s, 1H), 6.94~6.88 (m, 1H), 6.77~6.73 (m, 1H), 6.47 (d, J = 7.2 Hz, 1H), 6.11 (s, 1H) 5.65 (d, J = 5.2 Hz, 1H), 4.09 (d, J = 5.2 Hz, 1H), 1.79 (s, 3H), 1.40 (s, 3H); ^{13}C NMR (100 MHz, $CDCl_3$): δ 161.1 (d, J = 242 Hz), 149.6, 149.0 (d, J = 3 Hz), 142.4 (d, J = 8 Hz), 140.3, 135.5, 126.9, 126.3, 125.0, 121.5(d, J = 8 Hz) 120.8, 116.6, 114.1 (d, J = 23 Hz), 110.7 (d, J = 23 Hz), 84.1, 65.4, 50.6, 27.5, 23.4; ESI-MS calcd. for $C_{19}H_{17}FNO$ [M+H]: 294.1294, found: 294.1298.

Spectral data for (11b*R*,12*R*)-9-chloro-6,6-dimethyl-11b,12-dihydro-6*H*-5,12-epoxybenzo[*b*]indeno[1,2-*e*]azepine (3d).

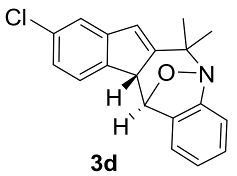

3d

Yellow solid, mp: 153-154 °C; ^{1}H NMR (400 MHz, $CDCl_3$): δ 7.50 (s, 1H), 7.17 (d, *J* = 8.0 Hz, 1H), 7.04~7.00 (m, 2H), 6.94 (d, *J* = 7.6 Hz, 1H), 6.75 (t, *J* = 7.6 Hz, 1H), 6.48 (d, *J* = 7.6 Hz, 1H), 6.12 (s, 1H), 5.67 (d, *J* = 5.2 Hz, 1H), 4.09 (d, *J* = 4.8 Hz, 1H), 1.79 (s, 3H), 1.40 (s, 3H); ^{13}C NMR (100 MHz, $CDCl_3$): δ 149.8, 149.5, 142.8, 142.2, 135.4, 130.5, 127.5, 126.9, 126.4, 125.1, 123.4, 121.7, 120.9, 116.6, 84.1, 65.5, 50.5, 27.5, 23.4; ESI-MS calcd. for $C_{19}H_{17}ClNO$ [M+H].: 310.0999, found: 310.0992

Spectral data for (11b*R*,12*R*)-6,6,10-trimethyl-11b,12-dihydro-6*H*-5,12-epoxy -benzo[*b*]indeno[1,2-*e*]azepine (3e).

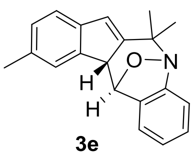

3e

Yellow solid, mp: 168-169 °C; ^{1}H NMR (400 MHz, $CDCl_3$): δ 7.39 (d, *J* =7.6 Hz, 1H), 7.03~6.92 (m, 4H), 6.73 (t, *J* = 7.2 Hz, 1H), 6.43 (d, *J* = 7.2 Hz, 1H), 6.11 (s, 1H), 5.66 (d, *J* = 5.2 Hz, 1H), 4.07 (d, *J* = 5.2 Hz, 1H), 2.31 (s, 3H), 1.79 (s, 3H), 1.40 (s, 3H); ^{13}C NMR (100 MHz, $CDCl_3$): δ 149.7, 149.3, 144.6, 137.6, 136.8, 135.9, 127.1, 126.7, 125.3, 124.9, 122.6, 121.7, 120.9, 116.5, 84.5, 65.5, 50.1, 27.6, 23.5, 21.5; ESI-MS calcd. for $C_{20}H_{20}NO$ [M+H]: 290.1545, found: 290.1546.

Spectral data for (11b*R*,12*R*)-10-fluoro-6,6-dimethyl-11b,12-dihydro-6*H*-5,12-epoxybenzo[*b*]indeno[1,2-*e*]azepine (3f).

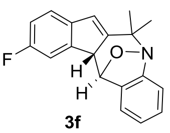

3f

Light reddish solid, mp: 163-164 °C; ^{1}H NMR (400 MHz, $CDCl_3$): δ 7.44 (dd, *J* = 8.0, 5.2 Hz, 1H), 7.03 (t, *J* = 7.6 Hz, 1H), 6.95 (d, *J* = 7.6 Hz, 1H), 6.90~6.85 (m, 1H), 6.80~6.73 (m, 2H), 6.43 (d, *J* = 7.6 Hz, 1H), 6.12 (s, 1H) 5.67 (d, *J* = 4.8 Hz, 1H), 4.06 (d, *J* = 5.2 Hz, 1H), 1.79 (s, 3H), 1.40 (s, 3H); ^{13}C NMR (100 MHz, $CDCl_3$): δ 162.6 (d, *J* = 242 Hz), 151.7, 149.5, 146.3 (d, *J* = 9 Hz), 136.0, 135.5, 126.9, 126.7, 125.1, 123.7(d, *J* = 9 Hz) 120.9, 116.6, 111.2 (d, *J* = 24 Hz), 108.1 (d, *J* = 23 Hz), 84.4, 65.5, 49.9, 27.5, 23.4; ESI-MS calcd. for $C_{19}H_{17}FNO$ [M+H]: 294.1294, found: 294.1282.

Spectral data for (11b*R*,12*R*)-10-chloro-6,6-dimethyl-11b,12-dihydro-6*H*-5,12-epoxybenzo[*b*]indeno[1,2-*e*]azepine (3g).

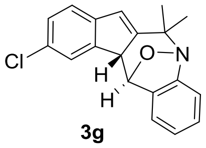

3g

Yellow solid, mp: 153-154 °C; ^{1}H NMR (400 MHz, $CDCl_3$): δ 7.50 (d, *J* = 1.2 Hz, 1H), 7.17 (dd, *J* = 8.0, 2.0 Hz, 1H), 7.05~7.00 (m, 2H), 6.94 (d, *J* = 7.6 Hz, 1H), 6.77~6.73 (m, 1H), 6.48 (d, *J* = 7.6 Hz, 1H), 6.12 (s, 1H), 5.67 (d, *J* = 5.2 Hz, 1H), 4.08 (d, *J* = 5.2 Hz, 1H), 1.79 (s, 3H), 1.40 (s, 3H); ^{13}C NMR (100 MHz, $CDCl_3$): δ 149.8, 149.5, 142.8, 142.2, 135.3, 130.5, 127.4, 126.9, 126.4, 125.1, 123.4, 121.7, 120.9, 116.6, 84.1, 65.4, 50.5, 27.5, 23.4; ESI-MS calcd. for $C_{19}H_{17}ClNO$ [M+H]: 310.0999, found: 310.0999.

Spectral data for (11b'*R*,12'*R*)-11b',12'-dihydrospiro[cyclopentane-1,6'-[5,12]epo -xybenzo[*b*]indeno[1,2-*e*]azepine] (3h).

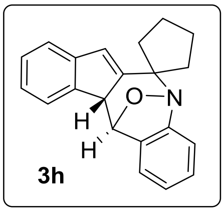

White solid, mp: 218-219 °C; ^{1}H NMR (400 MHz, $CDCl_3$): δ 7.51 (d, *J* = 6.0 Hz, 1H), 7.22~7.17 (m, 2H), 7.11~7.09 (m, 1H), 7.02 (t, *J* = 7.6 Hz, 1H), 6.94 (d, *J* = 7.2 Hz, 1H), 6.71 (t, *J* =7.6 Hz, 1H), 6.41 (d, *J* =7.2 Hz, 1H), 6.18 (s, 1H), 5.70 (d, *J* = 5.2 Hz, 1H), 4.04 (d, *J* = 5.2 Hz, 1H), 2.61~2.54 (m, 1H), 2.18~2.12 (m, 1H), 2.05~1.93 (m, 3H), 1.88~1.82 (m, 2H), 1.80~1.73 (m, 1H); ^{13}C NMR (100 MHz, $CDCl_3$): δ 150.4, 148.1, 144.4, 140.6, 135.8, 127.2, 127.1, 127.0, 124.9, 124.5, 123.0, 120.9, 120.8, 115.6, 84.3, 51.3, 38.4, 33.7, 24.0, 23.8; ESI-MS calcd. for $C_{21}H_{20}NO$ [M+H]: 302.1545, found: 302.1543.

Spectral data for (11b'*R*,12'*R*)-11b',12'-dihydrospiro[cyclohexane-1,6'-[5,12]epo -xybenzo[*b*]indeno[1,2-*e*]azepine] (3i).

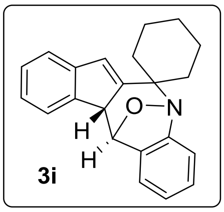

White solid, mp: 225-226 °C; ^{1}H NMR (400 MHz, $CDCl_3$): δ 7.51 (d, *J* = 6.0 Hz, 1H), 7.22~7.18 (m, 2H), 7.13~7.10 (m, 1H), 7.00 (t, *J* = 8.0 Hz, 1H), 6.92 (d, *J* = 7.6 Hz, 1H), 6.69 (t, *J* =7.6 Hz, 1H), 6.37 (d, *J* =7.2 Hz, 1H), 6.22 (s, 1H), 5.69 (d, *J* = 5.2 Hz, 1H), 4.10 (d, *J* = 4.8 Hz, 1H), 2.47~2.42 (m, 1H), 2.16~2.14 (m, 1H), 2.09~1.95 (m, 2H), 1.85~1.74 (m, 2H), 1.71~1.63 (m, 2H), 1.59~1.52 (m, 1H), 1.51~1.47 (m, 1H); ^{13}C NMR (100 MHz, $CDCl_3$): δ 149.5, 148.5, 144.6, 140.4, 135.9, 128.0, 127.2, 126.7, 124.6, 124.4, 122.9, 120.9, 120.8, 116.1, 84.6, 68.0, 50.3, 36.1, 32.3, 26.2, 22.1, 22.0; ESI-MS calcd. for $C_{22}H_{22}NO$ [M+H]: 316.1701, found: 316.1714.

Spectral data for (11b*R*,12*R*)-6,6-dipropyl-11b,12-dihydro-6*H*-5,12-epoxybenzo [*b*]indeno[1,2-*e*]azepine (3j).

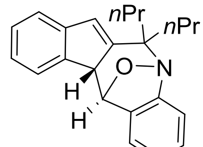

3j

Light brown solid, mp: 215-216 °C; ^{1}H NMR (400 MHz, $CDCl_3$): δ 7.51 (d, *J* = 5.6 Hz, 1H), 7.19~7.16 (m, 2H), 7.10 (d, *J* = 7.6 Hz, 1H), 7.01 (t, *J* = 7.6 Hz, 1H), 6.85 (d, *J* = 7.6 Hz, 1H), 6.70 (t, *J* = 7.2 Hz, 1H), 6.39 (d, *J* = 7.6 Hz, 1H), 6.16 (s, 1H), 5.67 (d, *J* = 5.2 Hz, 1H), 4.07 (d, *J* = 4.8 Hz, 1H), 2.36~2.29 (m, 1H), 2.18~2.13 (m, 1H), 1.90~1.85 (m, 1H), 1.79~1.71 (m, 1H), 1.59~1.53 (m, 1H), 1.46~1.37 (m, 2H), 1.18~1.11 (m, 1H), 1.02 (t, *J* = 7.2 Hz, 3H), 0.94 (t, *J* = 7.6 Hz, 3H); ^{13}C NMR (100 MHz, $CDCl_3$): δ 149.5, 148.7, 144.6, 140.6, 136.2, 128.3, 127.2, 126.8, 124.7, 124.4, 122.9, 120.8, 116.3, 84.3, 71.3, 50.3, 37.1, 34.4, 17.7, 15.8, 14.7, 14.6; ESI-MS calcd. for $C_{23}H_{26}NO$[M+H]: 332.2014, found: 332.2008.

Spectral data for (11b*R*,12*R*)-2,6,6-trimethyl-11b,12-dihydro-6*H*-5,12-epoxy -benzo[*b*]indeno[1,2-*e*]azepine (3k).

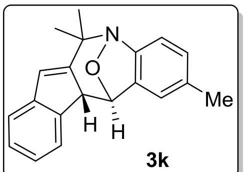

3k

Off-white solid, mp: 101-102 °C; ^{1}H NMR (400 MHz, $CDCl_3$): δ 7.51~7.50 (m, 1H), 7.20~7.17 (m, 2H), 7.12~7.11 (m, 2H), 6.83~6.79 (m, 2H), 6.23 (s, 1H), 6.18 (s, 1H), 5.65 (d, *J* = 3.4 Hz, 1H), 4.09 (d, *J* = 3.4 Hz, 1H), 2.02 (s, 3H), 1.78 (s, 3H), 1.39 (s, 3H). ^{13}C NMR (100 MHz, $CDCl_3$): δ 149.3, 147.2, 144.4, 140.5, 136.0, 134.6, 127.2, 127.1, 127.1, 124.4, 122.9, 121.6, 121.0, 116.2, 84.4, 65.3, 50.5, 27.6, 23.5, 20.9; ESI-MS calcd for $C_{20}H_{20}NO$ [M+H] : 290.1545, found: 290.1555.

Spectral data for (11b*R*,12*R*)-2-bromo-6,6-dimethyl-11b,12-dihydro-6*H*-5,12-epo -xybenzo[*b*]indeno[1,2-*e*]azepine (3l).

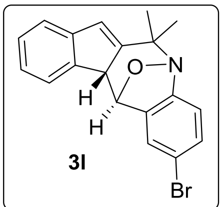

3l

Light brown solid, mp: 178-179 °C; ^{1}H NMR (400 MHz, $CDCl_3$): δ 7.49 (d, *J* = 7.6 Hz, 1H), 7.25~7.21 (m, 2H), 7.20~7.13 (m, 2H), 6.81 (d, *J* = 8.4 Hz, 1H), 6.53 (s, 1H),

6.21 (s, 1H), 5.66 (d, *J* = 5.2 Hz, 1H), 4.09 (d, *J* = 5.2 Hz, 1H), 1.78 (s, 3H), 1.39 (s, 3H); ^{13}C NMR (100 MHz, CDCl$_3$): δ 149.1, 148.4, 144.1, 139.9, 138.3, 129.8 127.5, 124.8, 124.1, 122.9, 121.3, 118.0, 84.2, 65.6, 50.2, 27.5, 23.4; ESI-MS calcd. for $C_{19}H_{17}BrNO$ [M+H]: 354.0494, found: 354.0504.

Spectral data for (11b*R*,12*R*)-2-chloro-6,6-dimethyl-11b,12-dihydro-6*H*-5,12-epo-xybenzo[*b*]indeno[1,2-*e*]azepine (3m).

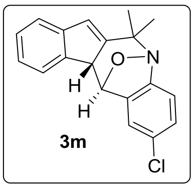

Light brown solid, mp: 156-157 ^{o}C; ^{1}H NMR (400 MHz, CDCl$_3$): δ 7.50 (d, *J* = 6.4 Hz, 1H), 7.23~7.19 (m, 2H), 7.18~7.14 (m, 1H), 6.98 (d, *J* = 8.0 Hz, 1H), 6.86 (d, *J* = 8.4 Hz, 1H), 6.38 (s, 1H), 6.21 (s, 1H), 5.66 (d, *J* = 5.2 Hz, 1H), 4.09 (d, *J* = 5.2 Hz, 1H), 1.78 (s, 3H), 1.40 (s, 3H); ^{13}C NMR (100 MHz, CDCl$_3$): δ 148.5, 148.4, 144.1, 140.0, 138.0, 130.3 127.5, 126.8, 124.8, 122.9, 121.3, 117.5, 84.3, 65.6, 50.2, 27.5, 23.4; ESI-MS calcd. for $C_{19}H_{17}ClNO$ [M+H]: 310.0999, found: 310.0991.

Spectral data for (11b*R*,12*R*)-2-methoxy-6,6-dimethyl-11b,12-dihydro-6*H*-5,12-epoxybenzo[*b*]indeno[1,2-*e*]azepine (3n).

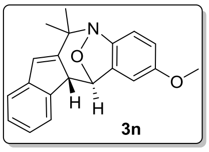

Semi solid, ^{1}H NMR (400 MHz, CDCl$_3$): δ 7.50 (d, *J* = 6.36, 1H), 7.33~7.31 (m, 1H), 7.23~7.11 (m, 3H), 6.83 (d, *J* = 8.4, 1H), 6.50 (dd, $J_{1,3}$= 2.4 Hz, $J_{2,3}$= 8.4 Hz, 1H), 6.18 (s, 1H), 5.96 (d, *J* = 2.4 Hz, 1H), 5.64 (d, *J* = 5.1 Hz, 1H), 4.09 (d, *J* = 5.04 Hz, 1H), 3.48 (s, 3H), 1.77 (s, 3H), 1.38 (s, 3H); ^{13}C NMR (100 MHz, CDCl$_3$): δ 157.1, 149.2, 144.4, 142.6, 140.5, 137.4, 127.3, 127.2, 124.5, 122.9, 121.0, 116.9, 111.2, 107.6, 84.7, 65.4, 55.4, 50.4, 27.5, 23.4; ESI-MS calcd for $C_{20}H_{20}O_2$ [M+H]: 306.1494, found: 306.1489.

Spectral data for (11b*R*,12*R*)-6,6-dimethyl-11b,12-dihydro-6*H*-5,12-epoxybenzo[*b*]indeno[1,2-*e*]azepin-2-yl ethyl carbonate (3o).

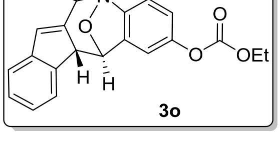

White solid, mp: 124-125 °C; ^{1}H NMR (400 MHz, CDCl$_3$): δ 7.48 (d, *J* = 6.9, 1H), 7.20~7.17 (m, 2H), 7.15~7.12 (m, 1H), 6.92~6.90 (m, 1H), 6.84~6.82 (m, 1H), 6.23 (d, *J* = 2.1, 1H), 6.20 (s, 1H), 5.68 (d, *J* = 5.08, 1H), 4.17 (q, *J* = 14.3, 7.1, 2H), 4.10

(d, J = 4.9, 1H), 1.78 (s, 3H), 1.40 (s, 3H), 1.27 (t, J = 7.2, 3H); ^{13}C NMR (100 MHz, $CDCl_3$): δ 153.2, 148.6, 148.1, 147.4, 144.3, 140.1, 137.5, 127.5, 127.5, 124.7, 122.9, 121.2, 119.2, 116.9, 114.4, 84.5, 65.6, 64.8, 50.3, 27.5, 23.4, 14.1; ESI-MS calcd. for $C_{22}H_{22}NO_4$ [M+H]: 364.1549, found: 364.1546.

Spectral data for(11b*R*,12*R*)-3-bromo-6,6-dimethyl-11b,12-dihydro-6*H*-5,12-epoxybenzo[*b*]indeno[1,2-*e*]azepine (3p).

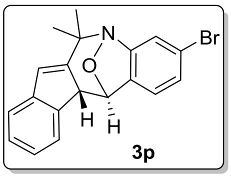

Gummy solid; ^{1}H NMR (400 MHz, $CDCl_3$): δ 7.49 (d, J = 4.7, 1H), 7.24~7.18 (m, 2H), 7.16~ 7.13 (m, 2H), 6.81 (d, J = 5.4, 1H), 6.52 (d, J = 1.2, 1H), 6.21 (s, 1H), 5.66 (d, J = 3.4, 1H), 4.09 (d, J = 3.3, 1H), 1.78 (s, 3H), 1.39 (s, 3H); ^{13}C NMR (100 MHz, $CDCl_3$): δ 149.1, 148.4, 144.1, 139.9, 138.3, 136.9, 129.8, 127.6, 124.9, 124.1, 122.9, 121.3, 118.0, 84.2, 65.6, 50.3, 27.5, 23.4, one peak merged; ESI-MS calcd for $C_{19}H_{17}BrNO$ [M+H]: 354.0494, found: 354.0490.

Spectral data for (11b*R*,12*R*)-3,6,6-trimethyl-11b,12-dihydro-6*H*-5,12-epoxybe -nzo[*b*]indeno[1,2-*e*]azepine (3q).

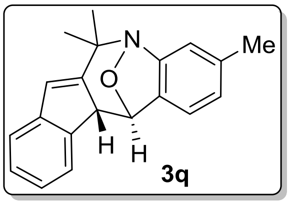

Off-white solid, mp: 177-178 °C; ^{1}H NMR (400 MHz, $CDCl_3$): δ 7.50 (d, J = 4.7, 1H), 7.18 (m, 2H), 7.12~7.11 (m, 1H), 6.74 (s, 1H), 6.52 (d, J = 4.9, 1H), 6.28 (d, J = 5.0, 1H), 6.17 (s, 1H), 5.65 (d, J = 3.4, 1H), 4.08 (d, J = 3.3, 1H), 2.21 (s, 3H) ,1.78 (s, 3H), 1.40 (s, 3H); ^{13}C NMR (100 MHz, $CDCl_3$): δ 149.9, 149.4, 144.4, 140.6, 136.7, 132.9, 127.2, 127.1, 125.5, 124.4, 123.0, 120.9, 120.4, 117.4, 84.4, 65.4, 50.6, 27.6, 23.5, 21.5; ESI-MS calcd. for $C_{20}H_{20}NO$ [M+H]: 290.1545, found: 290.1542.

Spectral data for (11b*R*,12*S*)-6,6-dimethyl-12-phenyl-11b,12-dihydro-6*H*-5,12-ep -oxybenzo[*b*]indeno[1,2-*e*]azepine (3r).

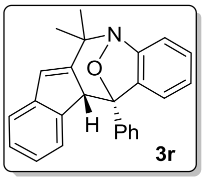

White solid, mp: 130-132 °C; ^{1}H NMR (400 MHz, $CDCl_3$): δ 7.85~7.82 (m, 2H), 7.51~7.50 (m, 3H), 7.16~7.12 (m, 1H), 7.09~7.06 (m, 3H), 7.02~6.95(m, 2H), 6.78 (t, J = 7.4, 1H), 6.37 (d, J = 7.6, 1H), 6.21 (s, 1H), 4.60 (s, 1H), 1.85 (s, 3H), 1.47 (s, 3H);

^{13}C NMR (100 MHz, $CDCl_3$): δ 151.7, 150.7, 144.4, 140.3, 136.9, 136.7, 129.5, 128.9, 128.6, 127.2, 127.2, 126.7, 124.9, 124.4, 124.3, 122.4, 120.7, 116.6, 94.6, 64.8, 51.9, 27.7, 23.6, two peaks merged; ESI-MS calcd. for $C_{25}H_{22}NO$ [M+H: 352.1701, found: 352.1699].

Spectral data for (11b*R*,12*R*)-6,6,12-trimethyl-11b,12-dihydro-6*H*-5,12-epoxyben-zo[*b*]indeno[1,2-*e*]azepine (3s).

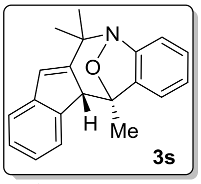

White solid, mp: 192-193 °C; ^{1}H NMR (400 MHz, $CDCl_3$): δ 7.57~7.55 (m, 1H), 7.21~7.15 (m, 2H), 7.10~7.08 (m, 1H), 7.03~7.00 (m, 1H), 6.95~6.93 (m, 1H), 6.75 (t, J = 7.4, 1H), 6.33 (d, J = 7.2, 1H), 6.12 (s, 1H), 3.84 (s,1 H), 2.00 (s, 3H), 1.79 (s, 3H), 1.42 (s, 3H); ^{13}C NMR (100 MHz, $CDCl_3$): δ150.3, 150.2, 144.3, 140.5, 138.4, 127.2, 126.5, 126.5, 125.0, 124.4, 123.8, 120.9, 120.0, 116.7, 90.5, 64.6, 55.9, 27.6, 23.4,20.9; ESI-MS calcd. for $C_{20}H_{20}NO$ [M+H]: 290.1545, found: 290.1541.

Spectral data for 2-(((6a*S*,11a*S*,12*S*,*E*)-6,6,11a-trimethyl-6,6a,11a,12-tetrahydro-11*H*-5,12-epoxybenzo[*b*]indeno[2,1-*e*]azepin-11-ylidene)amino)benzaldehyde(5a).

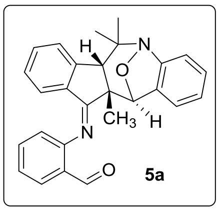

Yellow solid, mp: 225-226 ^{o}C; ^{1}H NMR (600 MHz, $CDCl_3$): δ 10.14 (s, 1H), 7.88 (d, J = 7.8 Hz, 1H), 7.53 (d, J = 7.2 Hz, 1H), 7.24~7.18 (m, 2H), 7.05~7.01 (m, 2H), 6.94 (d, J = 6.0 Hz, 1H), 6.84~6.79 (m, 2H), 6.69 (t, J = 7.2 Hz, 1H), 6.65 (t, J = 7.2 Hz, 1H), 6.43 (d, J = 7.2 Hz, 1H), 5.01 (s, 1H), 3.10 (s, 1H), 1.96 (s, 3H), 1.88 (s, 3H), 1.56 (s, 3H); ^{13}C NMR (150 MHz, $CDCl_3$): δ 189.8, 175.4, 154.5, 151.3, 149.0, 138.1, 135.1, 133.9, 131.2, 129.4, 127.2, 127.1, 126.1, 125.8, 125.2, 125.0, 123.5, 122.2, 119.5, 117.6, 84.5, 59.9, 52.6, 49.8, 33.3, 26.8, 25.6; ESI-MS calcd. for $C_{27}H_{25}N_2O_2$ [M+H]: 409.1916, found: 409.1909.

Spectral data for 2-(((6a*S*,11a*S*,12*S*,*E*)-6,6,8,11a-tetramethyl-6,6a,11a,12-tetrahy-dro-11*H*-5,12-epoxybenzo[*b*]indeno[2,1-*e*]azepin-11-ylidene)amino)benzaldehyde (5b).

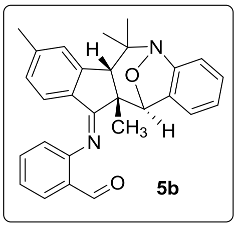

Yellow solid, mp: 238-239 °C; ^{1}H NMR (600 MHz, $CDCl_3$): δ 10.13 (s, 1H), 7.87 (dd, *J* = 7.8, 1.8 Hz, 1H), 7.53 (t, *J* = 7.2 Hz, 1H), 7.18 (t, *J* = 7.8 Hz, 1H), 7.05 (d, *J* = 6.6 Hz, 1H), 7.02 (s, 1H), 6.95 (s, 1H), 6.85~6.81 (m, 2H), 6.72 (t, *J* = 6.0 Hz, 1H), 6.47 (d, *J* = 7.8 Hz, 1H), 6.27 (d, *J* = 7.8 Hz, 1H), 5.00 (s, 1H), 3.01 (s, 1H), 2.12 (s, 3H), 1.93 (s, 3H), 1.86 (s, 3H), 1.54 (s, 3H); ^{13}C NMR (150 MHz, $CDCl_3$): δ 189.9, 175.4, 154.9, 151.7, 148.9, 142.0, 138.1, 135.2, 131.3, 129.1, 127.8, 127.3, 127.0, 125.6, 125.1, 125.0, 123.3, 122.2, 119.7, 117.5, 84.4, 59.8, 52.6, 49.7, 33.4, 26.8, 25.8, 21.8; ESI-MS calcd. for $C_{28}H_{27}N_2O_2$ [M+H]: 423.2071, found: 423.2073.

Spectral data for 2-(((6a*S*,11a*S*,12*S*,*E*)-8-fluoro-6,6,11a-trimethyl-6,6a,11a,12-tetrahydro-11*H*-5,12-epoxybenzo[*b*]indeno[2,1-*e*]azepin-11-ylidene)amino)benzaldehyde (5c).

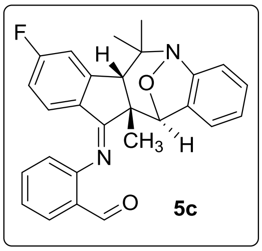

Light brownish solid, mp: 192-193 °C; ^{1}H NMR (600 MHz, $CDCl_3$): δ 10.10 (s, 1H), 7.87 (d, *J* = 7.8 Hz 1H), 7.55 (s, 1H), 7.23~7.19 (m, 1H), 7.01~6.85 (m, 5H), 6.72 (s, 1H), 6.35 (d, *J* = 6.0 Hz, 2H), 5.00 (s, 1H), 3.04 (s, 1H), 1.95 (s, 3H), 1.86 (s, 3H), 1.56 (s, 3H); ^{13}C NMR (150 MHz, $CDCl_3$): δ 189.8, 173.8, 163.9 (d, *J* = 253 Hz), 154.2, 148.6, 137.7, 135.3, 130.1, 129.6, 127.4 (d, *J* = 9.6 Hz), 127.3, 125.4, 124.7, 123.6, 122.0, 119.4, 117.6, 114.1 (d, *J* = 156 Hz), 114.0 (d, *J* = 158 Hz), 84.1, 59.8, 52.1, 50.3, 33.0, 26.6, 25.3; ESI-MS calcd. for $C_{27}H_{24}FN_2O_2$ [M+H]: 427.1822, found: 427.1826.

Spectral data for 2-(((6a*S*,11a*S*,12*S*,*E*)-8-chloro-6,6,11a-trimethyl-6,6a,11a,12-tetrahydro-11*H*-5,12-epoxybenzo[*b*]indeno[2,1-*e*]azepin-11-ylidene)amino)benzaldehyde (5d).

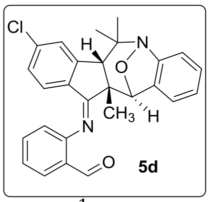

Light yellow solid, mp: 187-188 °C; ^{1}H NMR (600 MHz, $CDCl_3$): δ 10.10 (s, 1H), 7.87 (d, *J* = 7.8 Hz, 1H), 7.56 (s, 1H), 7.24~7.21 (m, 2H), 7.02 (s, 1H), 6.93 (s, 1H), 6.87 (s, 2H), 6.73 (s, 1H), 6.63 (d, *J* = 8.4 Hz, 1H), 6.31 (d, *J* = 5.8 Hz, 1H), 5.00 (s, 1H), 3.05 (s, 1H), 1.95 (s, 3H), 1.87 (s, 3H), 1.57 (s, 3H); ^{13}C NMR (150 MHz, $CDCl_3$): δ 189.7, 174.1, 154.0, 152.9, 148.8, 137.9, 137.8, 135.2, 132.4, 129.9, 127.3, 126.7, 126.5, 125.4, 124.9, 123.7, 122.1, 119.3, 117.7, 84.2, 59.9, 52.3, 50.2,

33.1, 26.7, 25.4; ESI-MS calcd. for $C_{27}H_{24}N_2O_2$ [M+H]: 443.1526, found: 443.1529.

Spectral data for 2-(((6a*S*,11a*S*,12*S*,*E*)-6,6,9,11a-tetramethyl-6,6a,11a,12-tetrahy-dro-11*H*-5,12-epoxybenzo[*b*]indeno[2,1-*e*]azepin-11-ylidene)amino)benzaldehyde (5e).

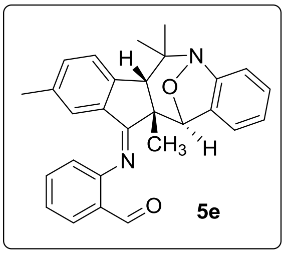

Yellow solid, mp: 203-204 °C; ^{1}H NMR (600 MHz, $CDCl_3$): δ 10.13 (s, 1H), 7.88 (d, *J* = 7.2 Hz, 1H), 7.54 (s, 1H), 7.24~7.19 (m, 1H), 7.08 (d, *J* = 7.8 Hz, 1H), 7.03 (s, 1H), 6.95 (s, 1H), 6.83 (d, *J* = 10.8 Hz, 3H), 6.72 (s, 1H), 6.15 (s, 1H), 5.00 (s, 1H), 3.02 (s, 1H), 1.92 (s, 3H), 1.86 (s, 6H), 1.53 (s, 3H); ^{13}C NMR (150 MHz, $CDCl_3$): δ 190.0 175.7, 154.8, 148.8, 148.5, 138.0, 135.7, 135.2, 133.8, 132.5, 129.0, 127.0, 126.9, 126.0, 125.1, 124.8, 123.4, 122.0, 119.5, 117.5, 84.4, 59.7, 52.1, 49.9, 33.3, 26.7, 25.7, 20.8; ESI-MS calcd. for $C_{28}H_{27}N_2O_2$ [M+H]: 423.2073, found: 423.2080.

Spectral data for 2-(((6a*S*,11a*S*,12*S*,*E*)-9-fluoro-6,6,11a-trimethyl-6,6a,11a,12-tetrahydro-11*H*-5,12-epoxybenzo[*b*]indeno[2,1-*e*]azepin-11-ylidene)amino)benzaldehyde (5f).

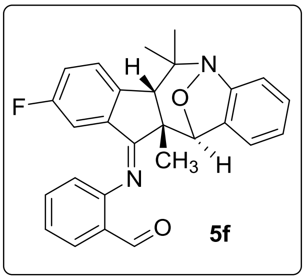

Light brownish solid, mp: 191-192 °C; ^{1}H NMR (600 MHz, $CDCl_3$): δ 10.11 (s, 1H), 7.88 (d, *J* = 7.8 Hz, 1H), 7.57 (t, *J* = 7.2 Hz, 1H), 7.24 (d, *J* = 7.8 Hz, 1H), 7.19 (dd, *J* = 7.8, 4.8, Hz, 1H), 7.04 (d, *J* = 6.6 Hz, 1H), 6.94 (s, 1H), 6.84 (s, 2H), 6.76~6.73 (m, 2H), 6.06 (s, 1H), 5.01 (s, 1H), 3.04 (s, 1H), 1.95 (s, 3H), 1.86 (s, 3H), 1.56 (s, 3H); ^{13}C NMR (150 MHz, $CDCl_3$): δ 189.8, 174.4, 160.2 (d, *J* = 246 Hz), 153.6, 148.8, 146.7, 137.9, 135.3, 130.0, 128.4 (d, *J* = 7.0 Hz), 127.3, 125.4, 124.7, 123.6, 122.0, 119.1, 118.7 (d, *J* = 22.8 Hz), 117.6, 111.7 (d, *J* = 23 Hz), 84.3, 59.7, 51.9, 50.5, 33.1, 26.8, 25.4; ESI-MS calcd. for $C_{27}H_{24}FN_2O_2$[M+H]: 427.1822, found: 427.1826.

Spectral data for 2-(((6a*S*,11a*S*,12*S*,*E*)-9-chloro-6,6,11a-trimethyl-6,6a,11a,12-tetrahydro-11*H*-5,12-epoxybenzo[*b*]indeno[2,1-*e*]azepin-11-ylidene)amino)benzaldehyde (5g).

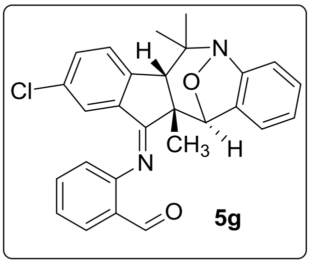

Light yellow solid, mp: 187-188 °C; ^{1}H NMR (600 MHz, $CDCl_3$): δ 10.10 (s, 1H), 7.87 (d, *J* = 7.8, Hz, 1H), 7.55 (t, *J* = 7.8 Hz, 1H), 7.24~7.21 (m, 2H), 7.03 (d, *J* = 6.6, Hz, 1H), 6.93 (s, 1H), 6.89~6.87 (m, 3H), 6.74~6.72 (m, 1H), 6.64 (d, *J* = 7.2 Hz, 1H), 6.33 (d, *J* = 8.4 Hz, 1H), 5.00 (s, 1H), 3.06 (s, 1H), 1.95 (s, 3H), 1.87 (s, 3H), 1.56 (s, 3H); ^{13}C NMR (150 MHz, $CDCl_3$): δ 189.8, 174.1, 154.0, 152.8, 148.7, 137.8, 137.7, 135.3, 132.3, 129.8, 127.3, 126.7, 126.5, 125.4, 124.7, 123.7, 122.0, 119.2, 117.7, 84.1, 59.8, 52.2, 50.1 33.1, 26.7, 25.4; ESI-MS calcd. for $C_{27}H_{24}ClN_2O_2$ [M+H]: 443.1526, found: 443.1529.

Spectral data for 2-(((6a'*S*,11a'*S*,12'*S*,*E*)-11a'-methyl-11a',12'-dihydrospiro-[cyclopentane-1,6'-[5,12]epoxybenzo[*b*]indeno[2,1-*e*]azepin]-11'(6a'*H*)-ylidene)amino)benzaldehyde (5h).

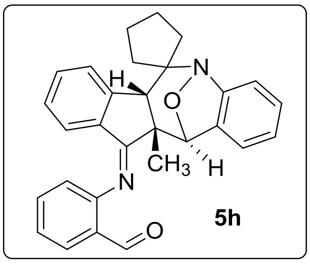

White solid, mp: 204-205 °C; ^{1}H NMR (600 MHz, $CDCl_3$): δ 10.19 (s, 1H), 7.88 (d, *J* = 7.8, Hz, 1H), 7.57 (t, *J* = 7.2 Hz, 1H), 7.20 (t, *J* = 8.4 Hz, 2H), 6.93 (t, *J* = 7.8 Hz, 2H), 6.80 (d, *J* = 7.8 Hz, 2H), 6.77 (t, *J* = 7.2 Hz, 1H), 6.60 (t, *J* = 7.8 Hz, 1H), 6.55 (t, *J* = 7.2 Hz, 1H), 6.37 (d, *J* = 7.2 Hz, 1H), 4.96 (s, 1H), 3.11 (t, *J* = 9.6 Hz, 1H), 3.07 (s, 1H), 2.36~2.33 (m, 1H), 2.15~2.04 (m, 3H), 1.94~1.91 (m, 4H), 1.86~1.82 (m, 2H); ^{13}C NMR (150 MHz, $CDCl_3$): δ 189.9, 174.7, 154.7, 152.1, 149.0, 138.2, 135.2, 133.9, 131.4, 129.5, 127.3, 125.9, 125.6, 125.5, 125.3, 124.9, 123.4, 122.0, 119.5, 116.8, 84.6, 71.9, 51.7, 51.6, 41.9, 36.5, 23.1, 22.7, 22.6; ESI-MS calcd. for $C_{29}H_{27}N_2O_2$ [M+H]: 435.2073, found: 435.2078.

Spectral data for 2-(((6a'*S*,11a'*S*,12'*S*,*E*)-11a'-methyl-11a',12'-dihydrospiro[cyclo

-hexane-1,6'-[5,12]epoxybenzo[*b*]indeno[2,1-*e*]azepin]-11'(6a'*H*)-ylidene)amino)benzaldehyde (5i).

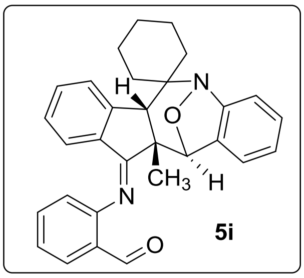

White solid, mp: 228-229 °C; ^{1}H NMR (600 MHz, CDCl$_3$): δ 10.15 (s, 1H), 7.87 (d, *J* = 7.8, Hz, 1H), 7.54 (t, *J* = 9.0 Hz, 1H), 7.25 (d, *J* = 7.8 Hz, 1H), 7.19 (t, *J* = 7.8 Hz, 1H), 6.97 (t, *J* = 7.2 Hz, 3H), 6.81 (d, *J* = 7.8 Hz, 1H), 6.77 (t, *J* = 7.8 Hz, 1H), 6.62 (t, *J* = 7.8 Hz, 2H), 6.42 (d, *J* = 7.8 Hz, 1H), 4.98 (s, 1H), 3.04 (s, 1H), 2.87~2.84 (m, 1H), 2.17~2.02 (m, 4H), 1.97 (s, 3H), 1.93~1.89 (m, 1H), 1.83~1.80 (m, 1H), 1.72~1.70 (m, 1H), 1.63~1.52 (m, 2H); ^{13}C NMR (150 MHz, CDCl$_3$): δ 189.8, 174.4, 154.7, 151.0, 148.9, 138.2, 135.2, 134.1, 131.2, 129.5, 127.1, 126.9, 125.9, 125.8, 125.1, 124.9, 123.4, 122.0, 119.5, 116.9, 84.6, 62.1, 52.1, 50.6, 40.3, 34.8, 26.4, 25.3, 22.4, 22.2; ESI-MS calcd. for $C_{30}H_{29}N_2O_2$ [M+H]: 449.2229, found: 449.2224.

Spectral data for 2-(((6a*S*,11a*S*,12*S*,*E*)-11a-cyclopropyl-6,6-dimethyl-6,6a,11a,12-tetrahydro-11*H*-5,12-epoxybenzo[*b*]indeno[2,1-*e*]azepin-11-ylidene)amino)benzaldehyde (5j).

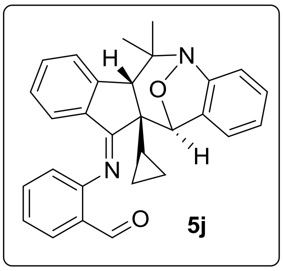

Yellow solid, mp: 212-213 °C; ^{1}H NMR (400 MHz, CDCl$_3$): δ 10.26 (s, 1H), 7.94 (d, *J* = 7.6 Hz, 1H), 7.53 (t, *J* = 7.2 Hz, 1H), 7.43 (d, *J* = 6.8 Hz, 1H), 7.31~7.22 (m, 4H), 7.19~7.15 (m, 2H), 6.98~6.94 (m, 1H), 6.85 (d, *J* = 8.0 Hz, 1H), 6.46 (d, *J* = 8.0 Hz, 1H), 5.52 (s, 1H), 2.76 (s, 1H), 1.34 (s, 3H), 0.84 (s, 3H), 0.66~0.59 (m, 1H), 0.55~0.39 (m, 2H), 0.22~0.05 (m, 1H), -0.30~-0.34 (m, 1H),; ^{13}C NMR (150 MHz, CDCl$_3$): δ 190.6, 175.5, 154.9, 151.6, 148.4, 138.4, 135.5, 135.4, 131.8, 128.0, 127.3, 127.2, 126.3, 126.2, 125.4, 125.2, 124.0, 122.8, 119.8, 117.8, 80.3, 61.8, 52.8, 50.7, 27.2, 27.1, 18.0, 3.1, -0.7; ESI-MS calcd. for $C_{29}H_{27}N_2O_2$ [M+H]: 435.2073, found: 435.2068.

Spectral data for 2-(((6a*S*,11a*S*,12S,*E*)-11a-(((tert-butyldimethylsilyl)oxy)methyl)-6,6-dimethyl-6,6a,11a,12-tetrahydro-11*H*-5,12-epoxybenzo[*b*]indeno[2,1-*e*]azepin-11-ylidene)amino)benzaldehyde (5k).

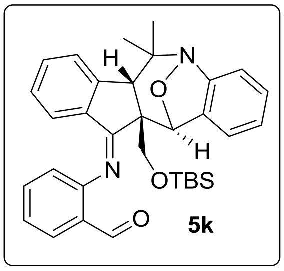

Light yellow Liquid; ^{1}H NMR (400 MHz, $CDCl_3$): δ 10.18 (s, 1H), 7.92 (d, *J* = 7.9 Hz, 1H), 7.54 (s, 1H), 7.26 (d, *J* = 8.0 Hz, 1H), 7.20 (t, *J* = 7.2, 1H), 7.06 (s, 1H), 7.02 (t, *J* = 7.2, 1H), 6.93 (s, 1H), 6.87~6.83 (m, 2H), 6.73 (s, 1H), 6.63 (t, *J* = 8.0 Hz, 1H), 6.35 (d, *J* = 8.0 Hz, 1H), 4.93 (s, 1H), 4.65 (d, *J* = 9.5 Hz, 1H), 4.46 (d, *J* = 9.5 Hz, 1H), 3.75 (s, 1H), 1.85 (s, 3H), 1.57 (s, 3H), 0.81 (s, 9H), 0.09 (d, *J* = 15.5 Hz, 6H); ^{13}C NMR (150 MHz, $CDCl_3$): δ 190.5, 174.0, 155.1, 152.4, 149.1, 137.6, 135.2, 133.9, 131.2, 128.4, 127.4, 127.1, 125.9, 125.7, 125.3, 123.6, 122.2, 119.5, 117.7, 81.0, 67.8, 60.0, 55.2, 45.0, 33.5, 26.8, 25.9, 18.2, -5.3, -5.5; ESI-MS calcd. for $C_{33}H_{38}N_2O3Si$ [M+H]: 539.2730, found: 539.2718.

Spectral data for 5-methyl-2-(((6a*S*,11a*S*,12*S*,*E*)-2,6,6,11a-tetramethyl-6,6a,11a,12-tetrahydro-11*H*-5,12-epoxybenzo[*b*]indeno[2,1-*e*]azepin-11-ylidene)amino)benzaldehyde (5l).

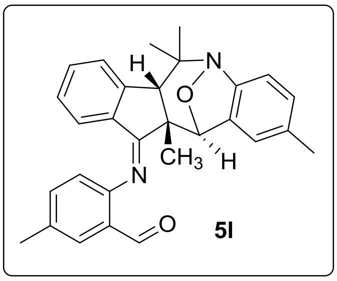

Light yellow solid, mp: 202-203 ^{o}C; ^{1}H NMR (600 MHz, $CDCl_3$): δ 10.13 (s, 1H), 7.70 (s, 1H), 7.36 (d, *J* = 8.4 Hz, 1H), 7.23 (t, *J* = 5.8 Hz, 1H), 7.02 (t, *J* = 8.4 Hz, 1H), 6.83 (d, *J* = 11.4 Hz, 2H), 6.70~6.59 (m, 3H), 6.46 (d, *J* = 7.2 Hz, 1H), 4.95 (s, 1H), 3.07 (s, 1H), 2.41 (s, 3H), 2.02 (s, 3H), 1.93 (s, 3H), 1.86 (s, 3H), 1.52 (s, 3H); ^{13}C NMR (150 MHz, $CDCl_3$): δ 190.1 175.8, 152.4, 151.2, 146.5, 138.2, 136.1, 134.9, 134.0, 133.2, 131.1, 129.1, 127.4, 127.3, 125.9, 125.6, 124.8, 123.1, 119.4, 117.2, 84.6, 84.4, 60.1, 52.6, 49.7, 33.3, 26.8, 25.7, 20.7, 20.6; ESI-MS calcd. for $C_{29}H_{29}N_2O_2$ [M+H]: 437.2229, found: 437.2237.

Spectral data for 5-bromo-2-(((6a*S*,11a*S*,12*S*,*E*)-2-bromo-6,6,11a-trimethyl-6,6a,11a,12-tetrahydro-11*H*-5,12-epoxybenzo[*b*]indeno[2,1-*e*]azepin-11-ylidene)amino)benzaldehyde (5m).

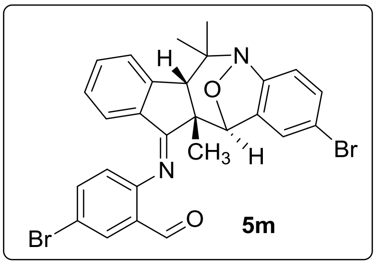

Light brown solid, mp: 189-190 °C; ^{1}H NMR (600 MHz, $CDCl_3$): δ 10.02 (s, 1H), 7.99 (d, *J* = 1.8 Hz, 1H), 7.66 (d, *J* = 8.4 Hz, 1H), 7.25 (d, *J* = 7.8 Hz, 1H), 7.15 (s, 1H), 7.10 (d, *J* = 7.8 Hz, 1H), 6.94 (d, *J* = 8.4 Hz, 1H), 6.83~6.79 (m, 2H), 6.68 (d, *J* = 7.8 Hz, 1H), 6.54 (d, *J* = 7.8 Hz, 1H), 4.97 (s, 1H), 3.11 (s, 1H), 1.91 (s, 3H), 1.85 (s, 3H), 1.50 (s, 3H); ^{13}C NMR (150 MHz, $CDCl_3$): δ 188.3, 175.9, 152.8, 151.1, 148.2, 140.6, 138.0, 133.4, 132.5, 131.8, 130.0, 127.6, 126.6, 126.3, 125.8, 125.7, 121.3, 118.9, 118.4, 116.7, 84.2, 60.1, 52.9, 49.7, 33.4, 26.6, 25.9; ESI-MS calcd. for $C_{27}H_{23}Br_2N_2O_2$ [M+H]: 565.0126, found: 565.0067.

Spectral data for 5-chloro-2-(((6a*S*,11a*S*,12*S*,*E*)-2-chloro-6,6,11a-trimethyl-6,6a,11a,12-tetrahydro-11*H*-5,12-epoxybenzo[*b*]indeno[2,1-*e*]azepin-11-ylidene)amino)benzaldehyde (5n).

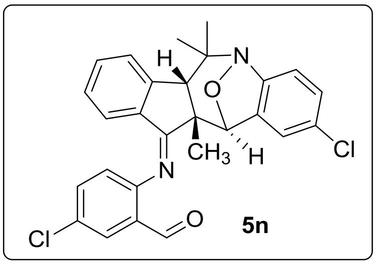

Light yellow solid, mp: 184-185 °C; ^{1}H NMR (600 MHz, $CDCl_3$): δ 10.04 (s, 1H), 7.85 (d, *J* = 2.4 Hz, 1H), 7.53 (d, *J* = 8.4 Hz, 1H), 7.26 (d, *J* = 7.8 Hz, 1H), 7.11 (d, *J* = 7.2 Hz, 1H), 7.00 (s, 1H), 6.88 (d, *J* = 8.4 Hz, 1H), 6.81~6.79 (m, 2H), 6.74 (d, *J* = 8.4 Hz, 1H), 6.53 (d, *J* = 8.4 Hz, 1H), 4.99 (s, 1H), 3.12 (s, 1H), 1.93 (s, 3H), 1.86 (s, 3H), 1.51 (s, 3H); ^{13}C NMR (150 MHz, $CDCl_3$): δ 188.5, 176.1, 152.4, 151.2, 147.7, 140.2, 135.2, 133.5, 131.9, 130.8, 129.6, 129.4, 127.6, 127.1, 126.6, 125.8, 122.8, 121.0, 118.5, 84.3, 60.2, 52.8, 49.7, 33.4, 26.7, 25.9; ESI-MS calcd. for $C_{27}H_{23}Cl_2N_2O_2$ [M+H]: 477.1137, found: 477.1140.

Spectral data for 5-methoxy-2-((*E*)-((6a*S*,11a*S*,12*S*)-2-methoxy-6,6,11a-trimethyl-11a,12-dihydro-6*H*-5,12-epoxybenzo[*b*]indeno[2,1-*e*]azepin-11(6a*H*)-ylidene)amino)benzaldehyde (5o).

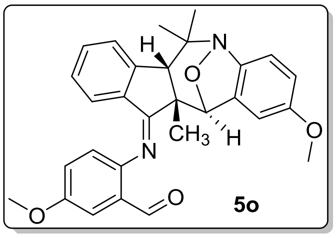

Yellow solid, mp: 160-161 °C; ^{1}H NMR (400 MHz, $CDCl_3$): δ 10.14 (s, 1H), 7.38 (d, J = 5.0, 1H), 7.25~7.22 (m, 2H), 7.16 (dd, $J_{1,3}$ = 5.8 Hz, $J_{2,3}$ = 3.9 Hz, 1H), 7.05 (t, J = 5.2 Hz, 1H), 6.89~6.85 (m, 1H) 6.72~6.68 (m, 2H), 6.55 (s, 1H), 6.31 (dd, $J_{1,3}$ = 5.6 Hz, $J_{2,3}$ = 3.9 Hz, 1H), 4.94 (s, 1H), 3.89 (s, 3H), 3.86 (s, 3H), 3.07 (s, 1 H), 1.93 (s, 3H), 1.85 (s, 3H), 1.53 (s, 3H); ^{13}C NMR (100 MHz, CDCl3): δ 189.7, 176.5, 164.2, 157.7, 156.4, 151.3, 148.7, 139.4, 131.2, 127.2, 126.1, 125.7, 123.7, 123.7, 121.0, 118.1, 113.2, 112.6, 110.8, 108.5, 84.7, 60.2, 55.7, 55.7, 52.2, 33.1, 26.8; ESI-MS calcd. for $C_{29}H_{29}N_2O_4$ [M+H]: 469.2124, found: 469.2127.

Spectral data for (6a*S*,11a*S*,12*S*,*E*)-11-((5-((ethoxycarbonyl)oxy)-2-formylphenyl)imino)-6,6,11a-trimethyl-6a,11,11a,12-tetrahydro-6*H*-5,12-epoxybenzo[*b*]indeno[2,1-*e*]azepin-3-yl ethyl carbonate (5p).

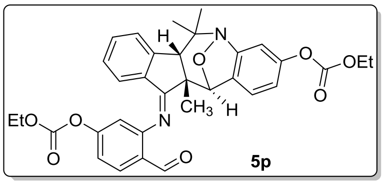

Gummy solid; ^{1}H NMR (400 MHz, $CDCl_3$): δ 10.07 (s, 1H), 7.70~7.69 (m, 1H), 7.38 (d, J = 5.6 Hz, 1H), 7.25 (d, J = 5.4 Hz, 1H), 7.09 (t, J = 5.0, 1H), 6.98 (s, 1H), 6.92 (s, 1H), 6.81 (d, J = 5.5 Hz, 1H), 6.75 (t, J = 5.0 Hz, 1H), 6.63 (d, J = 5.5 Hz, 1H), 6.55 (d, J = 5.2 Hz, 1H), 4.99 (s, 1H), 4.33 (q, J =9.5, 4.8 Hz, 2H), 4.24~4.20 (m, 2H), 3.11 (s, 1H), 1.93 (s, 3H), 1.86 (s, 3H), 1.54 (s, 3H), 1.39 (t, J = 6.4 Hz, 3H), 1.33 (t, J = 4.8 Hz, 3H); ^{13}C NMR (100 MHz, $CDCl_3$): δ 188.9, 176.1, 153.5, 153.1, 151.9, 151.0, 148.7, 147.5, 146.3, 139.5, 133.3, 131.7, 128.1, 127.3, 126.6, 126.3, 125.2, 121.2, 120.9, 119.5, 118.1, 115.3, 84.4, 65.0, 64.8, 60.0, 52.5, 49.5, 33.3, 26.7, 25.8, 14.2, 14.13; ESI-MS calcd. for $C_{33}H_{33}N_2O_8$ [M+H]: 585.2237, found: 585.2239.

Spectral data for 4-bromo-2-((*E*)-((6a*S*,11a*S*,12*S*)-3-bromo-6,6,11a-trimethyl-11a,12-dihydro-6*H*-5,12-epoxybenzo[*b*]indeno[2,1-*e*]azepin-11(6a*H*)-ylidene)amino)benzaldehyde (5q).

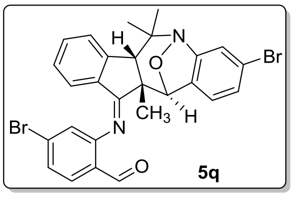

Light brown solid, mp: 211-212 ℃; ^{1}H NMR (400 MHz, $CDCl_3$): δ 10.03 (s, 1H), 7.75 (d, J = 5.6, 1H), 7.38~7.36 (m, 1H), 7.27~7.24 (m, 1H), 7.20 (s, 1H), 7.14~7.12 (m, 2H), 6.96 (t, J = 5.2, 1H), 6.84~6.81 (m, 1H), 6.70 (d, J = 5.4, 1H), 6.53 (d, J = 5.3, 1H), 4.99 (s, 1H), 3.11 (s, 1H), 1.91 (s, 3H), 1.86 (s, 3H), 1.51 (s, 3H); ^{13}C NMR (100 MHz, $CDCl_3$): δ 188.9, 154.8, 151.2, 148.0, 140.4, 138.1, 133.3, 132.4, 132.0, 131.2, 130.2, 130.0, 127.6, 126.9, 126.8, 125.8, 125.7, 125.6, 122.3, 121.3, 118.9, 84.1, 60.1, 52.8, 33.4, 26.6, one carbon merged; ESI-MS calcd. for $C_{27}H_{23}Br_2N_2O_2$ [M+H]: 565.0126, found: 565.0121.

Spectral data for 4-methyl-2-((*E*)-((6a*S*,11a*S*,12*S*)-3,6,6,11a-tetramethyl-11a,12-di-hydro-6*H*-5,12-epoxybenzo[*b*]indeno[2,1-*e*]azepin-11(6a*H*)-ylidene)amino)benzaldehyde (5r).

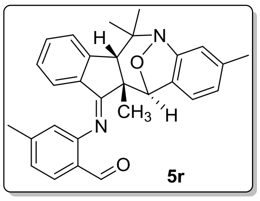

Pale yellow solid, mp: 220-221 ℃; ^{1}H NMR (400 MHz, $CDCl_3$): δ 10.05 (s, 1H), 7.78 (d, J = 5.3, 1H),7.23~7.22 (m, 1H), 7.04~ 6.99 (m, 2H), 6.90 (bs, 1H), 6.73 (s, 1H), 6.73~6.65 (m, 2H), 6.51 (d, J = 4.6, 1H), 6.46 (d, J = 5.1, 1H), 4.97 (s, 1H), 3.07 (s, 1H), 2.39 (s, 3H), 2.08 (s, 3H), 1.93 (s, 3H), 1.86 (s, 3H), 1.55 (s, 3H); ^{13}C NMR (100 MHz, $CDCl_3$): δ 189.5, 175.4, 154.8, 151.4, 149.1, 146.5, 137.0, 135.2, 133.9, 131.1, 129.4, 127.2, 126.1, 125.8, 125.7, 124.6, 121.8, 119.6, 118.3, 84.3, 56.8, 52.6, 49.7, 33.3, 26.8, 25.6, 21.9, 21.2, one carbon merged; ESI-MS calcd. for $C_{29}H_{29}N_2O_2$ [M+H]: 437.2229, found: 437.2236.

Spectral data for (11b*R*,12*S*)-6,6-dimethyl-5,6,11b,12-tetrahydrobenzo[*b*]indeno[1,2-*e*]azepin-12-ol (6a).

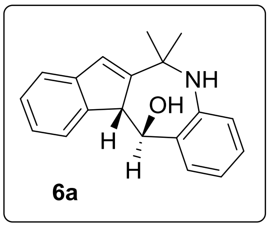

Light yellow solid, mp: 170-171 ^{o}C; ^{1}H NMR (400 MHz, d-Acetone): δ 7.24 (d, J =

6.0 Hz, 1H), 7.19~7.13 (m, 3H), 7.10~7.03 (m, 2H), 6.94 (d, J = 6.8 Hz, 1H), 6.83 (t, J = 7.6 Hz, 1H), 6.48 (s, 1H), 6.10 (s, 1H), 4.68 (d, J = 4.4 Hz, 1H), 4.38 (s, 1H), 3.84 (d, J = 7.6 Hz, 1H), 1.61 (s, 3H), 1.45 (s, 3H); ^{13}C NMR (100 MHz, $CDCl_3$): δ 153.7, 144.3, 143.0, 142.9, 135.2, 128.3, 127.7, 127.1, 126.9, 125.0, 123.5, 123.2, 120.6, 76.2, 58.0, 57.2, 31.7, 28.0; ESI-MS calcd. for $C_{19}H_{20}NO$ [M+H]: 278.1545, found: 278.1549.

^{1}H NOE Data of Compound (6a).

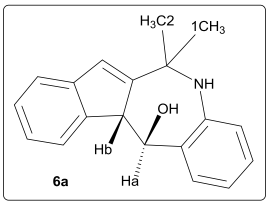

Irradiation	Intensity increase (%)
Hb (δ 3.84)	Ha (δ 4.68, 2.84), 2CH$_3$ (δ 1.45, 4.48)
Ha (δ 4.68)	Hb (δ 3.84, 4.36),
OH (δ 4.38)	Hb (δ 3.09, 3.18), 1CH$_3$ (δ 1.61, 4.32), 2CH$_3$ (δ 1.45, 4.73)

Spectral data for (6a*S*,11b*S*,12*S*)-6,6-dimethyl-5,6,6a,7,11b,12-hexahydrobenzo [*b*]indeno[1,2-*e*]azepin-12-ol (6b).

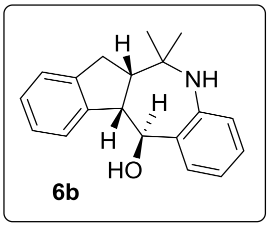

Light yellow solid, mp: 137-138 ^{o}C; ^{1}H NMR (600 MHz, $CDCl_3$): δ 7.53 (d, J = 7.2 Hz, 1H), 7.44 (d, J = 6.0 Hz, 1H), 7.19 (d, J = 6.6 Hz, 1H), 7.13~7.09 (m, 2H), 7.03 (t, J = 7.8 Hz, 1H), 6.93 (t, J = 7.2 Hz, 1H), 6.87 (d, J = 7.8 Hz, 1H), 4.72 (d, J = 9.6 Hz, 1H), 4.04 (s, 1H), 3.79 (s, 1H), 3.24~3.20 (m, 1H), 3.10~3.08 (m, 1H), 2.83~2.77 (m, 1H), 2.42~2.38 1H), 2.05~2.03(m, 1H), 1.28 (s, 3H), 1.04 (s, 3H); ^{13}C NMR (100 MHz, $CDCl_3$): δ 145.0, 144.0, 143.0, 134.7, 127.2, 127.1, 126.1, 125.9, 125.5, 124.9, 122.4, 121.8, 70.9, 53.8, 50.7, 33.0, 30.4, 29.9; ESI-MS calcd. for $C_{19}H_{22}NO$ [M+H]: 280.1701, found: 280.1695.

^{1}H NOE Data of Compound (6b).

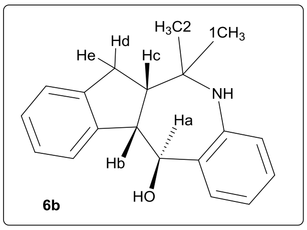

Irradiation	Intensity increase (%)
Hc (δ 2.40)	Hb (δ 3.09, 8.35), Hd (δ 2.78, 3.48), 1CH_3 (δ 1.28, 1.37), 2CH_3 (δ 1.04, 3.45).
Hb (δ 3.09)	Hc (δ 2.40, 7.36), Ha (δ 4.72, 3.55), Hd (δ 2.78, 1.56), 2CH_3 (δ 1.04, 2.32).
Ha (δ 4.72)	Hb (δ 3.09, 3.07), Hc (δ 2.40, 0.84), He (δ 3.22, 4.64), 1CH_3 (δ 1.28, 1.37), 2CH_3 (δ 1.04, 3.45).
Hd (δ 2.80)	He (δ 3.22, 16.4), Hc (δ 2.40, 4.87), Ha (δ 4.72, 2.85), 1CH_3 (δ 1.28, 4.47).
He (δ 3.22)	Hd (δ 2.80, 23.87), Hc (δ 2.40, 1.23), Ha (δ 4.72, 8.16), 1CH_3 (δ 1.28, 2.37), 2CH_3 (δ 1.04, 1.46).

Spectral data for (2-(((6a*S*,11R,11a*S*,12*R*)-6,6,11a-trimethyl-6a,11,11a,12-tetrah -ydro-6*H*-5,12-epoxybenzo[*b*]indeno[2,1-*e*]azepin-11-yl)amino)phenyl)methanol (7a).

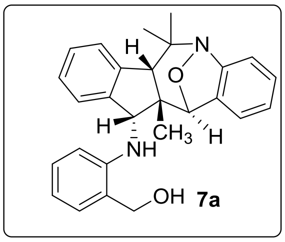

White solid, mp: 210-211 ^{o}C; ^{1}H NMR (400 MHz, $CDCl_3$): δ 7.26~7.21 (m, 1H), 7.17~7.14 (m, 2H), 7.01 (d, *J* = 7.6, Hz, 1H), 6.93 (t, *J* = 8.8 Hz, 2H), 6.82 (t, *J* = 7.6 Hz, 2H), 6.76 (t, *J* = 7.6, Hz, 2H), 6.72 (t, *J* = 7.6 Hz, 1H), 6.63 (t, *J* = 7.6 Hz, 1H), 5.58 (d, *J* = 9.2 Hz, 1H), 4.95 (s, 1H), 4.93~4.82 (m, 3H), 2.98 (s, 1H), 1.90 (s, 3H), 1.86~1.82 (m, 1H), 1.81 (s, 3H), 1.66 (s, 3H); ^{13}C NMR (100 MHz, $CDCl_3$): δ 149.1, 148.6, 143.2, 142.2, 138.5, 130.0, 129.5, 126.5, 126.2, 125.6, 124.9, 124.3, 122.6, 122.3, 117.3, 116.9, 110.5, 82.0, 65.5, 65.2, 59.2, 52.8, 50.6, 32.1, 27.2, 27.0; ESI-MS calcd. for $C_{27}H_{29}N_2O_2$ [M+H]: 413.2229, found: 413.2253.

Spectral data for (6a*S*,11a*R*,12*S*)-6,6,11a-trimethyl-6,6a,11a,12-tetrahydro-11*H*-5,12-epoxybenzo[*b*]indeno[2,1-*e*]azepin-11-one (7b).

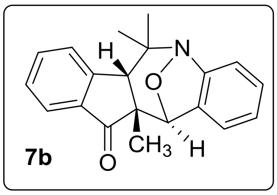

White solid, mp: 195-196 °C; ^{1}H NMR (400 MHz, $CDCl_3$): δ 7.47 (d, *J* = 7.6 Hz, 1H), 7.29~7.23 (m, 2H), 7.07 (t, *J* = 7.2, Hz, 1H), 6.80~6.74 (m, 3H), 6.63 (t, *J* = 7.6 Hz, 1H), 4.88 (s, 1H), 2.96 (s, 1H), 1.80 (s, 3H), 1.73 (s, 3H), 1.59 (s, 3H); ^{13}C NMR (100 MHz, $CDCl_3$): δ 207.1, 153.5, 147.7, 137.0, 136.3, 133.8, 127.1, 127.0, 126.9, 125.4, 123.5, 121.8, 117.2, 82.8, 59.7, 50.5, 32.9, 26.4, 22.4; ESI-MS calcd. for $C_{20}H_{20}NO_2$ [M+H]: 306.1494, found: 306.1489.

Spectral data for (6a*S*,11a*R*,12*R*)-12-hydroxy-6,6,11a-trimethyl-6,6a,11a,12-tetra-hydrobenzo[*b*]indeno[2,1-*e*]azepin-11(5*H*)-one (7c).

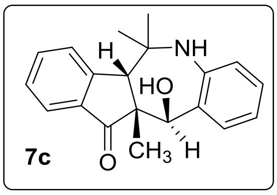

White solid, mp: 182-183 °C; ^{1}H NMR (400 MHz, $CDCl_3$): δ 7.77 (d, *J* = 8.0 Hz, 1H), 7.57~7.49 (m, 2H), 7.45~7.42 (m, 2H), 7.10 (t, *J* = 7.6, Hz, 1H), 6.97 (t, *J* = 7.2 Hz, 1H), 6.67 (d, *J* = 7.6 Hz, 1H), 5.57 (s, 1H), 4.29 (s, 1H), 3.39 (s, 1H), 2.84 (s, 1H), 1.48 (s, 3H), 1.04 (s, 3H), 0.73 (s, 3H); ^{13}C NMR (100 MHz, $CDCl_3$): δ 211.9, 153.7, 142.0, 136.5, 133.8, 129.9, 128.3, 127.9, 127.3, 125.9, 124.1, 120.6, 119.1, 69.6, 58.5, 57.8, 55.6, 33.6, 28.4, 21.6; ESI-MS calcd. for $C_{20}H_{22}NO_2$ [M+H]: 308.1651, found: 308.1663.

Spectral data for (6a*S*,11*R*,11a*S*,12*R*)-6,6,11a-trimethyl-5,6,6a,11,11a,12-hexahy-drobenzo[*b*]indeno[2,1-*e*]azepine-11,12-diol (7d).

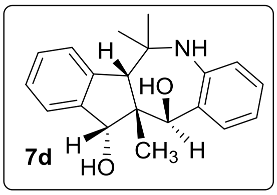

White solid, mp: 178-179 °C; ^{1}H NMR (400 MHz, d-Acetone): δ 7.45 (d, *J* = 4.8 Hz, 2H), 7.29~7.19 (m, 3H), 7.02 (t, *J* = 7.6, Hz, 1H), 6.87 (t, *J* = 7.6 Hz, 1H), 6.79 (d, *J* = 7.6 Hz, 1H), 5.73 (d, *J* = 3.6 Hz, 1H), 4.78 (d, *J* = 4.0 Hz, 1H), 4.36 (d, *J* = 4.0 Hz, 1H), 4.05 (s, 1H), 3.95 (d, *J* = 4.0 Hz, 1H), 2.35 (s, 1H), 1.50 (s, 3H), 1.05 (s, 3H),

0.71 (s, 3H); ^{13}C NMR (100 MHz, $CDCl_3$): δ 146.1, 146.0, 143.7, 134.8, 128.2, 127.8, 127.5, 127.4, 127.1, 126.3, 120.4, 120.1, 81.0, 68.3, 62.5, 58.0, 51.3, 34.2, 29.2, 22.1; ESI-MS calcd. for $C_{20}H_{24}NO_2$ [M+H]: 310.1807, found: 310.1827.

^{1}H NOE Data of Compound (7d).

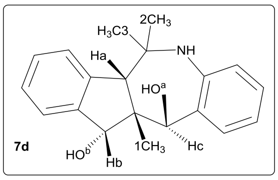

Irradiation	Intensity increase (%)
$1CH_3$ (δ 1.50)	Ha (δ 2.35, 3.51), Hb (δ 4.78, 3.29)
Hb (δ 4.78)	Hc (δ 5.74, 0.71), $1CH_3$ (δ 0.71, 4.06),
Hc (δ 5.74)	Ha (δ 2.35, 0.60), ^{a}OH (δ 3.94, 1.32), ^{b}OH (δ 4.36, 1.02), $2CH_3$ (δ 1.05, 3.91)
^{a}OH (δ 3.94)	Hb (δ 4.78, 4.32), Hc (δ 5.74, 9.70), $1CH_3$ (δ 0.71, 2.17), $2CH_3$ (δ 1.05, 2.62), $3CH_3$ (δ 1.50, 3.81)
^{b}OH (δ 4.36)	Hb (δ 4.78, 7.76), Hc (δ 5.74, 4.89),

Spectral data for (6a*S*,15b*S*,16*R*)-6,6,15b-trimethyl-5,6,6a,15,15b,16-hexahy-drobenzo[*c*]benzo[6,7]azepino[4,3-*a*]carbazol-16-ol (7e).

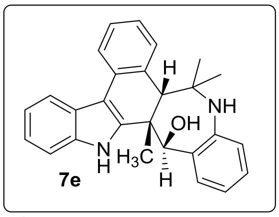

White solid, mp: 284-285 °C; ^{1}H NMR (400 MHz, d-Acetone): δ 10.45 (s, 1H), 7.97 (d, *J* = 8.4 Hz, 1H), 7.81 (t, *J* = 7.6 Hz, 1H), 7.58~7.56 (m, 1H), 7.47 (d, *J* = 7.6 Hz, 1H), 7.28 (t, *J* = 7.2 Hz, 1H), 7.16~7.08 (m, 4H), 6.99~6.95 (m, 2H), 6.85 (d, *J* = 7.6 Hz, 1H), 5.83 (d, *J* = 4.4 Hz, 1H), 4.84 (d, *J* = 4.0 Hz, 1H), 4.07 (s, 1H), 2.26 (s, 1H), 1.13 (s, 3H), 1.07 (s, 3H), 0.76 (s, 3H); ^{13}C NMR (150 MHz, $CDCl_3$): δ 144.9, 144.5, 137.8, 135.7, 134.0, 133.5, 133.3, 128.2, 128.1, 125.9, 125.1, 123.8, 122.7, 121.5, 121.1, 120.8, 120.3, 119.7, 112.9, 110.2, 72.3, 57.7, 56.1, 40.4, 33.7, 27.5, 18.8; ESI-MS calcd. for $C_{27}H_{27}N_2O$ [M+H]: 395.2123, found: 395.2092.

V) X-ray crystallographic structure and data

(a) X-ray data for compound 3a :

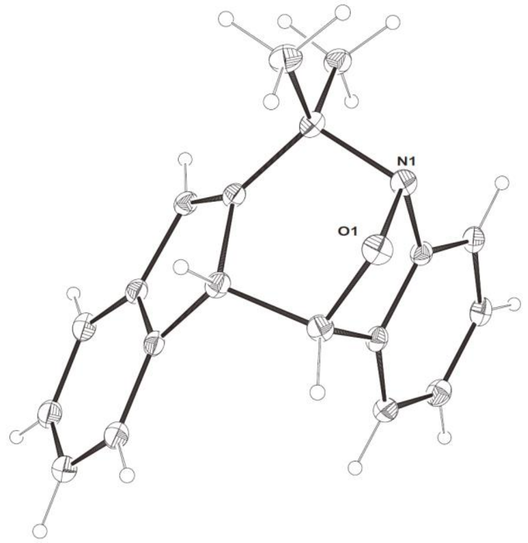

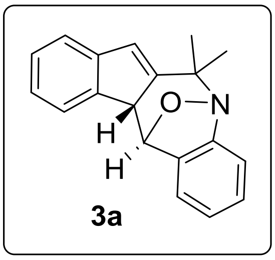

3a

Table 1. Crystal data and structure refinement for d19458.

Identification code	d19458	
Empirical formula	$C_{19} H_{17} N O$	
Formula weight	275.34	
Temperature	200(2) K	
Wavelength	0.71073 Å	
Crystal system	Orthorhombic	
Space group	P 21 21 21	
Unit cell dimensions	a = 6.2744(3) Å	a= 90°.
	b = 14.5625(9) Å	b= 90°.
	c = 15.6489(7) Å	g = 90°.
Volume	1429.85(13) Å 3	
Z	4	
Density (calculated)	1.279 Mg/m3	
Absorption coefficient	0.079 mm-1	
F(000)	584	
Crystal size	0.49 x 0.08 x 0.03 mm3	
Theta range for data collection	3.09 to 25.04°.	
Index ranges	-7<=h<=7, -17<=k<=17, -18<=l<=18	
Reflections collected	8485	
Independent reflections	2509 [R(int) = 0.0575]	
Completeness to theta = 25.04°	98.7 %	
Absorption correction	multi-scan	

Max. and min. transmission 0.9976 and 0.9625

Refinement method Full-matrix least-squares on F2

Data / restraints / parameters 2509 / 0 / 192

Goodness-of-fit on F2 1.111

Final R indices [I>2sigma(I)] R1 = 0.0421, wR2 = 0.0838

R indices (all data) R1 = 0.0725, wR2 = 0.0994

Absolute structure parameter 0(2)

Largest diff. peak and hole 0.179 and -0.176 e.Å -3

Table 2. Atomic coordinates (x 104) and equivalent isotropic displacement parameters (Å 2x 103)

for d19458. U(eq) is defined as one third of the trace of the orthogonalized Uij tensor.

	x	y	z	U(eq)
C(1)	5852(3)	4035(2)	5671(1)	27(1)
C(2)	7742(3)	3553(2)	5640(2)	32(1)
C(3)	8398(4)	3227(2)	4844(2)	35(1)
C(4)	7199(4)	3398(2)	4117(2)	37(1)
C(5)	5302(4)	3891(2)	4162(2)	35(1)
C(6)	4639(3)	4201(2)	4952(1)	27(1)
C(7)	2753(4)	4751(2)	5246(2)	32(1)
C(8)	3234(3)	5788(2)	5254(2)	30(1)
C(9)	3755(4)	6231(2)	4414(2)	31(1)
C(10)	2554(4)	6302(2)	3671(2)	40(1)
C(11)	3429(5)	6740(2)	2968(2)	47(1)
C(12)	5442(5)	7121(2)	3012(2)	45(1)
C(13)	6657(4)	7066(2)	3758(2)	38(1)
C(14)	5800(4)	6609(2)	4455(2)	30(1)
C(15)	6689(4)	6400(2)	5298(2)	30(1)
C(16)	5278(3)	5925(2)	5760(1)	27(1)
C(17)	5541(4)	5407(2)	6588(1)	31(1)
C(18)	7849(3)	5378(2)	6897(2)	36(1)
C(19)	4112(4)	5788(2)	7297(2)	41(1)
N(1)	4842(3)	4418(1)	6429(1)	31(1)
O(1)	2612(2)	4473(1)	6132(1)	35(1)

Table 3. Bond lengths [Å] and angles [°] for d19458.

C(1)-C(2)	1.378(3)
C(1)-C(6)	1.380(3)
C(1)-N(1)	1.457(3)
C(2)-C(3)	1.395(3)
C(2)-H(2)	0.9500
C(3)-C(4)	1.386(3)
C(3)-H(3)	0.9500
C(4)-C(5)	1.392(3)
C(4)-H(4)	0.9500
C(5)-C(6)	1.380(3)
C(5)-H(5)	0.9500
C(6)-C(7)	1.501(3)
C(7)-O(1)	1.447(3)
C(7)-C(8)	1.540(3)
C(7)-H(7)	1.0000
C(8)-C(9)	1.501(3)
C(8)-C(16)	1.520(3)
C(8)-H(8)	1.0000
C(9)-C(10)	1.389(3)
C(9)-C(14)	1.398(3)
C(10)-C(11)	1.385(4)
C(10)-H(10)	0.9500
C(11)-C(12)	1.381(4)
C(11)-H(11)	0.9500
C(12)-C(13)	1.396(3)
C(12)-H(12)	0.9500
C(13)-C(14)	1.386(3)
C(13)-H(13)	0.9500
C(14)-C(15)	1.465(3)
C(15)-C(16)	1.336(3)
C(15)-H(15)	0.9500
C(16)-C(17)	1.508(3)
C(17)-N(1)	1.526(3)
C(17)-C(18)	1.527(3)
C(17)-C(19)	1.531(3)
C(18)-H(18A)	0.9800
C(18)-H(18B)	0.9800

C(18)-H(18C)	0.9800
C(19)-H(19A)	0.9800
C(19)-H(19B)	0.9800
C(19)-H(19C)	0.9800
N(1)-O(1)	1.477(2)

C(2)-C(1)-C(6)	122.4(2)
C(2)-C(1)-N(1)	126.7(2)
C(6)-C(1)-N(1)	110.91(18)
C(1)-C(2)-C(3)	117.3(2)
C(1)-C(2)-H(2)	121.4
C(3)-C(2)-H(2)	121.4
C(4)-C(3)-C(2)	120.7(2)
C(4)-C(3)-H(3)	119.6
C(2)-C(3)-H(3)	119.6
C(3)-C(4)-C(5)	121.0(2)
C(3)-C(4)-H(4)	119.5
C(5)-C(4)-H(4)	119.5
C(6)-C(5)-C(4)	118.1(2)
C(6)-C(5)-H(5)	120.9
C(4)-C(5)-H(5)	120.9
C(1)-C(6)-C(5)	120.4(2)
C(1)-C(6)-C(7)	106.20(18)
C(5)-C(6)-C(7)	133.3(2)
O(1)-C(7)-C(6)	101.12(18)
O(1)-C(7)-C(8)	106.10(18)
C(6)-C(7)-C(8)	111.76(18)
O(1)-C(7)-H(7)	112.4
C(6)-C(7)-H(7)	112.4
C(8)-C(7)-H(7)	112.4
C(9)-C(8)-C(16)	102.46(18)
C(9)-C(8)-C(7)	117.2(2)
C(16)-C(8)-C(7)	107.41(19)
C(9)-C(8)-H(8)	109.8
C(16)-C(8)-H(8)	109.8
C(7)-C(8)-H(8)	109.8
C(10)-C(9)-C(14)	120.5(2)
C(10)-C(9)-C(8)	130.3(2)

C(14)-C(9)-C(8)	109.2(2)
C(11)-C(10)-C(9)	118.9(2)
C(11)-C(10)-H(10)	120.5
C(9)-C(10)-H(10)	120.5
C(12)-C(11)-C(10)	120.5(3)
C(12)-C(11)-H(11)	119.7
C(10)-C(11)-H(11)	119.7
C(11)-C(12)-C(13)	121.2(3)
C(11)-C(12)-H(12)	119.4
C(13)-C(12)-H(12)	119.4
C(14)-C(13)-C(12)	118.2(2)
C(14)-C(13)-H(13)	120.9
C(12)-C(13)-H(13)	120.9
C(13)-C(14)-C(9)	120.6(2)
C(13)-C(14)-C(15)	131.4(2)
C(9)-C(14)-C(15)	108.0(2)
C(16)-C(15)-C(14)	110.04(19)
C(16)-C(15)-H(15)	125.0
C(14)-C(15)-H(15)	125.0
C(15)-C(16)-C(17)	130.6(2)
C(15)-C(16)-C(8)	110.2(2)
C(17)-C(16)-C(8)	118.3(2)
C(16)-C(17)-N(1)	107.56(17)
C(16)-C(17)-C(18)	112.9(2)
N(1)-C(17)-C(18)	107.32(19)
C(16)-C(17)-C(19)	112.2(2)
N(1)-C(17)-C(19)	106.94(19)
C(18)-C(17)-C(19)	109.59(19)
C(17)-C(18)-H(18A)	109.5
C(17)-C(18)-H(18B)	109.5
H(18A)-C(18)-H(18B)	109.5
C(17)-C(18)-H(18C)	109.5
H(18A)-C(18)-H(18C)	109.5
H(18B)-C(18)-H(18C)	109.5
C(17)-C(19)-H(19A)	109.5
C(17)-C(19)-H(19B)	109.5
H(19A)-C(19)-H(19B)	109.5
C(17)-C(19)-H(19C)	109.5

H(19A)-C(19)-H(19C)	109.5
H(19B)-C(19)-H(19C)	109.5
C(1)-N(1)-O(1)	100.14(15)
C(1)-N(1)-C(17)	111.62(17)
O(1)-N(1)-C(17)	105.77(17)
C(7)-O(1)-N(1)	105.06(14)

Symmetry transformations used to generate equivalent atoms:

Table 4. Anisotropic displacement parameters (Å 2x 103)for d19458. The anisotropic
displacement factor exponent takes the form: -2p2[h2a*2U11 + ... + 2 h k a* b* U12]

	U11	U22	U33	U23	U13	U12
C(1)	29(1)	24(1)	29(1)	1(1)	2(1)	-5(1)
C(2)	31(1)	25(1)	38(1)	3(1)	-1(1)	0(1)
C(3)	32(1)	26(1)	47(2)	1(1)	5(1)	2(1)
C(4)	42(2)	33(2)	36(2)	-4(1)	8(1)	-2(1)
C(5)	41(1)	31(2)	32(1)	0(1)	-3(1)	-4(1)
C(6)	26(1)	23(1)	34(1)	1(1)	1(1)	-4(1)
C(7)	25(1)	34(2)	36(1)	2(1)	-2(1)	-3(1)
C(8)	24(1)	28(1)	38(1)	-3(1)	-2(1)	-1(1)
C(9)	34(1)	20(1)	39(1)	-3(1)	-6(1)	4(1)
C(10)	42(1)	28(1)	49(2)	-3(1)	-12(1)	1(1)
C(11)	70(2)	31(2)	41(2)	0(1)	-14(2)	2(2)
C(12)	69(2)	30(2)	35(2)	1(1)	2(2)	2(2)
C(13)	44(1)	29(1)	41(2)	0(1)	6(1)	1(1)
C(14)	32(1)	24(1)	35(2)	-4(1)	-1(1)	2(1)
C(15)	26(1)	27(1)	37(1)	-5(1)	-3(1)	-2(1)
C(16)	26(1)	24(1)	32(1)	-4(1)	-1(1)	1(1)
C(17)	31(1)	28(1)	34(1)	-5(1)	2(1)	-1(1)
C(18)	31(1)	36(2)	42(2)	-5(1)	-8(1)	-1(1)
C(19)	42(2)	46(2)	35(1)	-7(1)	5(1)	2(1)
N(1)	26(1)	33(1)	34(1)	1(1)	0(1)	-2(1)
O(1)	25(1)	42(1)	38(1)	2(1)	4(1)	-4(1)

Table 5. Hydrogen coordinates (x 104) and isotropic displacement parameters (Å 2x 103)
for d19458.

	x	y	z	U(eq)
H(2)	8564	3448	6140	38
H(3)	9681	2884	4800	42
H(4)	7680	3175	3580	45
H(5)	4486	4011	3663	42
H(7)	1430	4601	4917	38
H(8)	2037	6125	5536	36
H(10)	1155	6054	3646	48
H(11)	2638	6778	2452	57
H(12)	6009	7426	2526	54
H(13)	8035	7334	3786	46
H(15)	8067	6576	5489	36
H(18A)	7954	4975	7397	54
H(18B)	8310	5998	7053	54
H(18C)	8763	5141	6440	54
H(19A)	4520	6423	7421	61
H(19B)	4281	5414	7814	61
H(19C)	2622	5769	7111	61

(b) X-ray data for compound 5b :

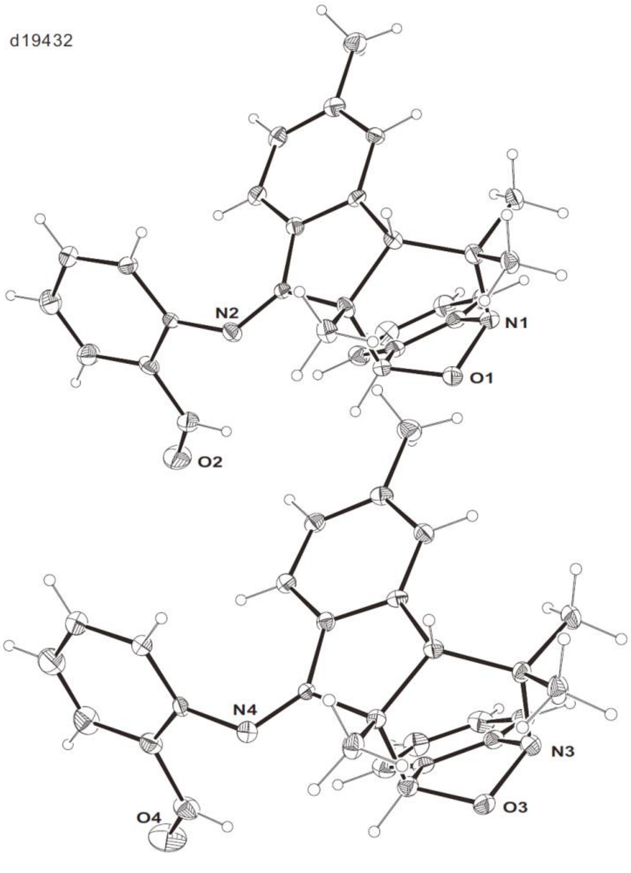

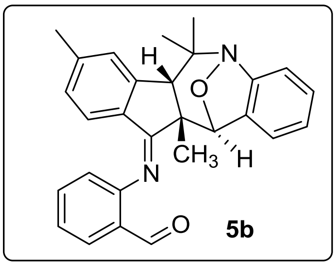

Table 1. Crystal data and structure refinement for d19432.

Identification code	d19432
Empirical formula	$C_{28} H_{26} N_2 O_2$
Formula weight	422.51
Temperature	200(2) K
Wavelength	0.71073 Å
Crystal system	Monoclinic
Space group	P 21/c
Unit cell dimensions	a = 21.9831(15) Å = 90°.
	b = 13.3250(11) Å = 112.177(2)°.
	c = 16.5868(14) Å = 90°.
Volume	4499.2(6) Å 3
Z	8
Density (calculated)	1.247 Mg/m3
Absorption coefficient	0.079 mm-1
F(000)	1792
Crystal size	0.34 x 0.07 x 0.03 mm3

Theta range for data collection 2.46 to 25.03°.
Index ranges -26<=h<=26, -15<=k<=15, -19<=l<=16
Reflections collected 59880
Independent reflections 7906 [R(int) = 0.0709]
Completeness to theta = 25.03° 99.4 %
Absorption correction multi-scan
Max. and min. transmission 0.9976 and 0.9737
Refinement method Full-matrix least-squares on F2
Data / restraints / parameters 7906 / 0 / 578
Goodness-of-fit on F2 1.080
Final R indices [I>2sigma(I)] R1 = 0.0899, wR2 = 0.2224
R indices (all data) R1 = 0.1198, wR2 = 0.2619
Largest diff. peak and hole 0.980 and -0.337 e.Å -3

Table 2. Atomic coordinates (x 104) and equivalent isotropic displacement parameters (Å 2x 103)
for d19432. U(eq) is defined as one third of the trace of the orthogonalized Uij tensor.

	x	y	z	U(eq)
C(1)	-269(2)	6106(4)	1783(3)	37(1)
C(2)	-612(3)	5867(4)	2397(4)	39(1)
C(3)	-391(3)	5611(4)	3264(4)	46(1)
C(4)	-866(3)	5451(5)	3615(4)	56(2)
C(5)	-1522(3)	5548(5)	3122(4)	53(2)
C(6)	-1742(3)	5798(4)	2248(4)	45(1)
C(7)	-1271(3)	5969(3)	1906(4)	39(1)
C(8)	-1395(2)	7315(4)	842(4)	38(1)
C(9)	-1454(3)	7446(4)	-104(4)	39(1)
C(10)	-2044(2)	7665(4)	877(4)	41(1)
C(11)	-770(2)	7872(3)	1474(3)	32(1)
C(12)	-748(2)	8320(3)	2324(3)	31(1)
C(13)	-1208(2)	8944(3)	2461(4)	35(1)
C(14)	-1083(3)	9336(4)	3297(4)	41(1)
C(15)	-1588(3)	10007(5)	3430(5)	57(2)
C(16)	-504(2)	9110(4)	3969(3)	40(1)
C(17)	-31(2)	8522(4)	3841(3)	37(1)

C(18)	-148(2)	8145(4)	3004(3)	32(1)
C(19)	278(2)	7538(3)	2689(3)	24(1)
C(20)	-113(2)	7248(4)	1760(3)	32(1)
C(21)	274(2)	7426(4)	1175(4)	37(1)
C(22)	1309(2)	7525(3)	3873(3)	27(1)
C(23)	1488(2)	8531(4)	4079(4)	40(1)
C(24)	1939(3)	8808(4)	4857(4)	51(1)
C(25)	2240(3)	8082(5)	5480(4)	60(2)
C(26)	2094(3)	7087(5)	5329(4)	57(2)
C(27)	1624(2)	6796(4)	4498(3)	40(1)
C(28)	1480(3)	5738(4)	4311(4)	52(2)
C(29)	4720(2)	3883(4)	8009(3)	35(1)
C(30)	4364(2)	4141(3)	7053(3)	35(1)
C(31)	4570(3)	4448(4)	6408(4)	45(1)
C(32)	4083(3)	4611(4)	5575(4)	52(1)
C(33)	3433(3)	4493(4)	5411(4)	51(2)
C(34)	3227(3)	4192(4)	6069(4)	43(1)
C(35)	3701(2)	4002(3)	6883(3)	35(1)
C(36)	3608(2)	2596(4)	7799(3)	31(1)
C(37)	2970(2)	2255(4)	7121(4)	46(1)
C(38)	3591(3)	2454(5)	8700(4)	44(1)
C(39)	4259(2)	2085(3)	7789(3)	31(1)
C(40)	4285(2)	1682(3)	6944(3)	29(1)
C(41)	3830(2)	1079(4)	6313(3)	34(1)
C(42)	3965(2)	704(4)	5610(3)	34(1)
C(43)	3473(3)	68(4)	4917(4)	50(1)
C(44)	4561(2)	961(3)	5547(3)	34(1)
C(45)	5024(2)	1532(3)	6168(3)	32(1)
C(46)	4891(2)	1876(3)	6884(3)	30(1)
C(47)	5298(2)	2485(3)	7625(3)	24(1)
C(48)	4893(2)	2756(4)	8171(3)	31(1)
C(49)	5293(2)	2569(4)	9137(3)	37(1)
C(50)	6325(2)	2494(3)	7486(3)	28(1)
C(51)	6523(2)	1489(4)	7576(4)	39(1)
C(52)	6973(3)	1173(5)	7243(4)	51(1)
C(53)	7237(3)	1837(6)	6846(5)	68(2)
C(54)	7074(3)	2831(6)	6775(4)	57(2)
C(55)	6609(2)	3164(4)	7103(3)	42(1)

C(56)	6446(3)	4247(4)	7085(4)	52(2)
N(1)	-1379(2)	6200(3)	1008(3)	38(1)
N(2)	870(2)	7244(3)	3042(3)	36(1)
N(3)	3605(2)	3730(3)	7671(3)	39(1)
N(4)	5888(2)	2810(3)	7879(3)	37(1)
O(1)	-768(2)	5838(3)	950(2)	40(1)
O(2)	1676(2)	5053(3)	4826(3)	76(1)
O(3)	4216(2)	4113(3)	8340(2)	39(1)
O(4)	6640(3)	4887(4)	6710(4)	90(2)

Table 3. Bond lengths [Å] and angles [°] for d19432.

C(1)-O(1)	1.448(6)
C(1)-C(2)	1.512(8)
C(1)-C(20)	1.563(7)
C(1)-H(1)	1.0000
C(2)-C(3)	1.375(8)
C(2)-C(7)	1.376(7)
C(3)-C(4)	1.392(8)
C(3)-H(3)	0.9500
C(4)-C(5)	1.369(9)
C(4)-H(4)	0.9500
C(5)-C(6)	1.385(9)
C(5)-H(5)	0.9500
C(6)-C(7)	1.375(8)
C(6)-H(6)	0.9500
C(7)-N(1)	1.450(7)
C(8)-N(1)	1.509(7)
C(8)-C(10)	1.523(7)
C(8)-C(9)	1.536(8)
C(8)-C(11)	1.566(7)
C(9)-H(9A)	0.9800
C(9)-H(9B)	0.9800
C(9)-H(9C)	0.9800
C(10)-H(10A)	0.9800
C(10)-H(10B)	0.9800
C(10)-H(10C)	0.9800
C(11)-C(12)	1.515(7)
C(11)-C(20)	1.577(6)

C(11)-H(11)	1.0000
C(12)-C(18)	1.394(6)
C(12)-C(13)	1.393(6)
C(13)-C(14)	1.408(8)
C(13)-H(13)	0.9500
C(14)-C(16)	1.373(8)
C(14)-C(15)	1.505(8)
C(15)-H(15A)	0.9800
C(15)-H(15B)	0.9800
C(15)-H(15C)	0.9800
C(16)-C(17)	1.381(7)
C(16)-H(16)	0.9500
C(17)-C(18)	1.406(7)
C(17)-H(17)	0.9500
C(18)-C(19)	1.474(7)
C(19)-N(2)	1.271(6)
C(19)-C(20)	1.505(6)
C(20)-C(21)	1.531(7)
C(21)-H(21A)	0.9800
C(21)-H(21B)	0.9800
C(21)-H(21C)	0.9800
C(22)-N(2)	1.402(6)
C(22)-C(27)	1.399(7)
C(22)-C(23)	1.402(7)
C(23)-C(24)	1.348(8)
C(23)-H(23)	0.9500
C(24)-C(25)	1.388(9)
C(24)-H(24)	0.9500
C(25)-C(26)	1.365(8)
C(25)-H(25)	0.9500
C(26)-C(27)	1.430(8)
C(26)-H(26)	0.9500
C(27)-C(28)	1.452(8)
C(28)-O(2)	1.212(7)
C(28)-H(28)	0.9500
C(29)-O(3)	1.443(6)
C(29)-C(30)	1.520(7)
C(29)-C(48)	1.547(7)

C(29)-H(29)	1.0000
C(30)-C(31)	1.372(8)
C(30)-C(35)	1.389(7)
C(31)-C(32)	1.409(8)
C(31)-H(31)	0.9500
C(32)-C(33)	1.357(8)
C(32)-H(32)	0.9500
C(33)-C(34)	1.390(8)
C(33)-H(33)	0.9500
C(34)-C(35)	1.383(7)
C(34)-H(34)	0.9500
C(35)-N(3)	1.446(7)
C(36)-C(37)	1.500(6)
C(36)-N(3)	1.525(7)
C(36)-C(38)	1.521(8)
C(36)-C(39)	1.589(6)
C(37)-H(37A)	0.9800
C(37)-H(37B)	0.9800
C(37)-H(37C)	0.9800
C(38)-H(38A)	0.9800
C(38)-H(38B)	0.9800
C(38)-H(38C)	0.9800
C(39)-C(40)	1.523(6)
C(39)-C(48)	1.575(6)
C(39)-H(39)	1.0000
C(40)-C(46)	1.396(6)
C(40)-C(41)	1.396(6)
C(41)-C(42)	1.400(7)
C(41)-H(41)	0.9500
C(42)-C(44)	1.394(7)
C(42)-C(43)	1.507(7)
C(43)-H(43A)	0.9800
C(43)-H(43B)	0.9800
C(43)-H(43C)	0.9800
C(44)-C(45)	1.373(6)
C(44)-H(44)	0.9500
C(45)-C(46)	1.402(7)
C(45)-H(45)	0.9500

C(46)-C(47)	1.462(6)
C(47)-N(4)	1.278(6)
C(47)-C(48)	1.534(6)
C(48)-C(49)	1.530(7)
C(49)-H(49A)	0.9800
C(49)-H(49B)	0.9800
C(49)-H(49C)	0.9800
C(50)-C(55)	1.375(7)
C(50)-C(51)	1.398(7)
C(50)-N(4)	1.415(6)
C(51)-C(52)	1.367(7)
C(51)-H(51)	0.9500
C(52)-C(53)	1.358(9)
C(52)-H(52)	0.9500
C(53)-C(54)	1.366(10)
C(53)-H(53)	0.9500
C(54)-C(55)	1.399(8)
C(54)-H(54)	0.9500
C(55)-C(56)	1.485(8)
C(56)-O(4)	1.223(7)
C(56)-H(56)	0.9500
N(1)-O(1)	1.463(5)
N(3)-O(3)	1.474(5)

O(1)-C(1)-C(2)	101.5(4)
O(1)-C(1)-C(20)	108.1(4)
C(2)-C(1)-C(20)	113.0(4)
O(1)-C(1)-H(1)	111.2
C(2)-C(1)-H(1)	111.2
C(20)-C(1)-H(1)	111.2
C(3)-C(2)-C(7)	121.4(5)
C(3)-C(2)-C(1)	133.4(5)
C(7)-C(2)-C(1)	105.2(5)
C(2)-C(3)-C(4)	116.8(5)
C(2)-C(3)-H(3)	121.6
C(4)-C(3)-H(3)	121.6
C(5)-C(4)-C(3)	121.6(6)
C(5)-C(4)-H(4)	119.2

C(3)-C(4)-H(4)	119.2
C(4)-C(5)-C(6)	121.4(6)
C(4)-C(5)-H(5)	119.3
C(6)-C(5)-H(5)	119.3
C(7)-C(6)-C(5)	116.9(5)
C(7)-C(6)-H(6)	121.6
C(5)-C(6)-H(6)	121.5
C(6)-C(7)-C(2)	121.9(5)
C(6)-C(7)-N(1)	127.0(5)
C(2)-C(7)-N(1)	110.9(5)
N(1)-C(8)-C(10)	104.6(4)
N(1)-C(8)-C(9)	106.6(4)
C(10)-C(8)-C(9)	106.6(5)
N(1)-C(8)-C(11)	113.0(4)
C(10)-C(8)-C(11)	115.3(4)
C(9)-C(8)-C(11)	110.1(5)
C(8)-C(9)-H(9A)	109.5
C(8)-C(9)-H(9B)	109.5
H(9A)-C(9)-H(9B)	109.5
C(8)-C(9)-H(9C)	109.5
H(9A)-C(9)-H(9C)	109.5
H(9B)-C(9)-H(9C)	109.5
C(8)-C(10)-H(10A)	109.5
C(8)-C(10)-H(10B)	109.5
H(10A)-C(10)-H(10B)	109.5
C(8)-C(10)-H(10C)	109.5
H(10A)-C(10)-H(10C)	109.5
H(10B)-C(10)-H(10C)	109.5
C(12)-C(11)-C(8)	121.4(4)
C(12)-C(11)-C(20)	103.0(4)
C(8)-C(11)-C(20)	115.0(4)
C(12)-C(11)-H(11)	105.3
C(8)-C(11)-H(11)	105.4
C(20)-C(11)-H(11)	105.3
C(18)-C(12)-C(13)	119.5(4)
C(18)-C(12)-C(11)	112.0(4)
C(13)-C(12)-C(11)	128.0(4)
C(12)-C(13)-C(14)	119.8(5)

C(12)-C(13)-H(13)	120.1
C(14)-C(13)-H(13)	120.1
C(16)-C(14)-C(13)	119.8(5)
C(16)-C(14)-C(15)	121.3(5)
C(13)-C(14)-C(15)	118.9(5)
C(14)-C(15)-H(15A)	109.5
C(14)-C(15)-H(15B)	109.5
H(15A)-C(15)-H(15B)	109.5
C(14)-C(15)-H(15C)	109.5
H(15A)-C(15)-H(15C)	109.5
H(15B)-C(15)-H(15C)	109.5
C(14)-C(16)-C(17)	121.3(5)
C(14)-C(16)-H(16)	119.3
C(17)-C(16)-H(16)	119.3
C(16)-C(17)-C(18)	119.2(5)
C(16)-C(17)-H(17)	120.4
C(18)-C(17)-H(17)	120.4
C(12)-C(18)-C(17)	120.2(4)
C(12)-C(18)-C(19)	109.8(4)
C(17)-C(18)-C(19)	130.0(4)
N(2)-C(19)-C(18)	133.1(5)
N(2)-C(19)-C(20)	118.8(4)
C(18)-C(19)-C(20)	108.1(4)
C(19)-C(20)-C(21)	111.7(4)
C(19)-C(20)-C(1)	105.7(4)
C(21)-C(20)-C(1)	110.3(4)
C(19)-C(20)-C(11)	106.0(4)
C(21)-C(20)-C(11)	113.1(4)
C(1)-C(20)-C(11)	109.7(4)
C(20)-C(21)-H(21A)	109.5
C(20)-C(21)-H(21B)	109.5
H(21A)-C(21)-H(21B)	109.5
C(20)-C(21)-H(21C)	109.5
H(21A)-C(21)-H(21C)	109.5
H(21B)-C(21)-H(21C)	109.5
N(2)-C(22)-C(27)	120.4(4)
N(2)-C(22)-C(23)	121.4(5)
C(27)-C(22)-C(23)	117.9(5)

C(24)-C(23)-C(22)	122.2(5)
C(24)-C(23)-H(23)	118.9
C(22)-C(23)-H(23)	118.9
C(23)-C(24)-C(25)	119.5(5)
C(23)-C(24)-H(24)	120.2
C(25)-C(24)-H(24)	120.2
C(26)-C(25)-C(24)	121.9(6)
C(26)-C(25)-H(25)	119.1
C(24)-C(25)-H(25)	119.1
C(25)-C(26)-C(27)	118.3(6)
C(25)-C(26)-H(26)	120.8
C(27)-C(26)-H(26)	120.8
C(22)-C(27)-C(26)	120.1(5)
C(22)-C(27)-C(28)	120.6(5)
C(26)-C(27)-C(28)	119.3(5)
O(2)-C(28)-C(27)	126.2(6)
O(2)-C(28)-H(28)	116.9
C(27)-C(28)-H(28)	116.9
O(3)-C(29)-C(30)	100.5(4)
O(3)-C(29)-C(48)	108.3(4)
C(30)-C(29)-C(48)	113.3(4)
O(3)-C(29)-H(29)	111.4
C(30)-C(29)-H(29)	111.4
C(48)-C(29)-H(29)	111.4
C(31)-C(30)-C(35)	120.8(5)
C(31)-C(30)-C(29)	133.6(5)
C(35)-C(30)-C(29)	105.6(4)
C(30)-C(31)-C(32)	117.2(5)
C(30)-C(31)-H(31)	121.4
C(32)-C(31)-H(31)	121.4
C(33)-C(32)-C(31)	122.1(6)
C(33)-C(32)-H(32)	119.0
C(31)-C(32)-H(32)	118.9
C(32)-C(33)-C(34)	120.4(5)
C(32)-C(33)-H(33)	119.8
C(34)-C(33)-H(33)	119.8
C(35)-C(34)-C(33)	118.1(5)
C(35)-C(34)-H(34)	121.0

C(33)-C(34)-H(34)	121.0
C(34)-C(35)-C(30)	121.3(5)
C(34)-C(35)-N(3)	128.0(5)
C(30)-C(35)-N(3)	110.5(4)
C(37)-C(36)-N(3)	104.0(4)
C(37)-C(36)-C(38)	109.6(5)
N(3)-C(36)-C(38)	105.0(4)
C(37)-C(36)-C(39)	116.9(4)
N(3)-C(36)-C(39)	112.4(4)
C(38)-C(36)-C(39)	108.2(4)
C(36)-C(37)-H(37A)	109.5
C(36)-C(37)-H(37B)	109.5
H(37A)-C(37)-H(37B)	109.5
C(36)-C(37)-H(37C)	109.5
H(37A)-C(37)-H(37C)	109.5
H(37B)-C(37)-H(37C)	109.5
C(36)-C(38)-H(38A)	109.5
C(36)-C(38)-H(38B)	109.5
H(38A)-C(38)-H(38B)	109.5
C(36)-C(38)-H(38C)	109.5
H(38A)-C(38)-H(38C)	109.5
H(38B)-C(38)-H(38C)	109.5
C(40)-C(39)-C(48)	103.6(4)
C(40)-C(39)-C(36)	120.5(4)
C(48)-C(39)-C(36)	114.6(4)
C(40)-C(39)-H(39)	105.7
C(48)-C(39)-H(39)	105.7
C(36)-C(39)-H(39)	105.7
C(46)-C(40)-C(41)	119.1(4)
C(46)-C(40)-C(39)	111.6(4)
C(41)-C(40)-C(39)	128.8(4)
C(40)-C(41)-C(42)	120.8(5)
C(40)-C(41)-H(41)	119.6
C(42)-C(41)-H(41)	119.6
C(44)-C(42)-C(41)	118.4(4)
C(44)-C(42)-C(43)	120.3(5)
C(41)-C(42)-C(43)	121.3(5)
C(42)-C(43)-H(43A)	109.5

C(42)-C(43)-H(43B)	109.5
H(43A)-C(43)-H(43B)	109.5
C(42)-C(43)-H(43C)	109.5
H(43A)-C(43)-H(43C)	109.5
H(43B)-C(43)-H(43C)	109.5
C(45)-C(44)-C(42)	122.1(4)
C(45)-C(44)-H(44)	118.9
C(42)-C(44)-H(44)	118.9
C(44)-C(45)-C(46)	118.9(4)
C(44)-C(45)-H(45)	120.5
C(46)-C(45)-H(45)	120.5
C(40)-C(46)-C(45)	120.5(4)
C(40)-C(46)-C(47)	109.9(4)
C(45)-C(46)-C(47)	129.5(4)
N(4)-C(47)-C(46)	133.5(5)
N(4)-C(47)-C(48)	117.9(4)
C(46)-C(47)-C(48)	108.6(4)
C(49)-C(48)-C(47)	110.4(3)
C(49)-C(48)-C(29)	110.2(4)
C(47)-C(48)-C(29)	106.6(4)
C(49)-C(48)-C(39)	113.5(4)
C(47)-C(48)-C(39)	104.6(4)
C(29)-C(48)-C(39)	111.1(3)
C(48)-C(49)-H(49A)	109.5
C(48)-C(49)-H(49B)	109.5
H(49A)-C(49)-H(49B)	109.5
C(48)-C(49)-H(49C)	109.5
H(49A)-C(49)-H(49C)	109.5
H(49B)-C(49)-H(49C)	109.5
C(55)-C(50)-C(51)	119.6(5)
C(55)-C(50)-N(4)	121.7(4)
C(51)-C(50)-N(4)	118.3(4)
C(52)-C(51)-C(50)	119.6(5)
C(52)-C(51)-H(51)	120.2
C(50)-C(51)-H(51)	120.2
C(53)-C(52)-C(51)	120.2(6)
C(53)-C(52)-H(52)	119.9
C(51)-C(52)-H(52)	119.9

C(52)-C(53)-C(54)	121.8(6)
C(52)-C(53)-H(53)	119.1
C(54)-C(53)-H(53)	119.1
C(53)-C(54)-C(55)	118.7(6)
C(53)-C(54)-H(54)	120.7
C(55)-C(54)-H(54)	120.7
C(50)-C(55)-C(54)	120.0(5)
C(50)-C(55)-C(56)	119.5(5)
C(54)-C(55)-C(56)	120.4(5)
O(4)-C(56)-C(55)	124.1(7)
O(4)-C(56)-H(56)	117.9
C(55)-C(56)-H(56)	117.9
C(7)-N(1)-O(1)	101.5(4)
C(7)-N(1)-C(8)	112.3(4)
O(1)-N(1)-C(8)	105.7(4)
C(19)-N(2)-C(22)	125.1(4)
C(35)-N(3)-O(3)	101.1(4)
C(35)-N(3)-C(36)	112.3(4)
O(3)-N(3)-C(36)	106.4(3)
C(47)-N(4)-C(50)	121.8(4)
C(1)-O(1)-N(1)	103.9(3)
C(29)-O(3)-N(3)	104.6(3)

Symmetry transformations used to generate equivalent atoms:

Table 4. Anisotropic displacement parameters (Å 2x 103)for d19432. The anisotropic
displacement factor exponent takes the form: -2 2[h2a*2U11 + ... + 2 h k a* b* U12]

	U11	U22	U33	U23	U13	U12
C(1)	32(2)	33(3)	42(3)	-9(2)	9(2)	3(2)
C(2)	41(3)	26(2)	48(3)	-3(2)	16(2)	1(2)
C(3)	47(3)	41(3)	48(3)	7(2)	16(3)	6(2)
C(4)	69(4)	57(4)	50(4)	4(3)	31(3)	-8(3)
C(5)	52(3)	53(4)	61(4)	0(3)	28(3)	-4(3)
C(6)	38(3)	43(3)	56(4)	-7(2)	19(3)	-7(2)

C(7)	44(3)	24(2)	48(3)	-3(2)	17(2)	-3(2)
C(8)	32(3)	43(3)	35(3)	-3(2)	9(2)	4(2)
C(9)	35(3)	49(3)	28(3)	-1(2)	5(2)	1(2)
C(10)	27(2)	51(3)	40(3)	-1(2)	8(2)	2(2)
C(11)	29(2)	34(3)	34(3)	2(2)	12(2)	3(2)
C(12)	36(2)	23(2)	36(3)	-5(2)	15(2)	-5(2)
C(13)	28(2)	28(2)	48(3)	0(2)	15(2)	2(2)
C(14)	44(3)	36(3)	53(3)	0(2)	28(3)	-3(2)
C(15)	52(3)	52(4)	70(4)	-18(3)	28(3)	1(3)
C(16)	44(3)	42(3)	37(3)	-8(2)	19(2)	-9(2)
C(17)	39(3)	35(3)	39(3)	-5(2)	16(2)	-6(2)
C(18)	32(2)	31(2)	32(2)	2(2)	12(2)	-2(2)
C(19)	26(2)	22(2)	26(3)	0(2)	11(2)	-1(2)
C(20)	24(2)	46(3)	28(3)	1(2)	11(2)	4(2)
C(21)	33(3)	51(3)	31(3)	0(2)	18(3)	-1(2)
C(22)	21(2)	28(2)	33(3)	-4(2)	13(2)	2(2)
C(23)	35(2)	31(3)	58(3)	-8(2)	21(2)	-2(2)
C(24)	39(3)	43(3)	65(4)	-14(3)	14(3)	-6(2)
C(25)	53(3)	65(4)	45(4)	-1(3)	-2(3)	4(3)
C(26)	59(3)	51(4)	47(3)	-8(3)	3(3)	7(3)
C(27)	34(2)	39(3)	42(3)	10(2)	10(2)	11(2)
C(28)	51(3)	37(3)	60(4)	2(3)	11(3)	5(2)
C(29)	33(2)	35(3)	33(3)	-4(2)	9(2)	2(2)
C(30)	41(3)	24(2)	38(3)	-5(2)	13(2)	1(2)
C(31)	48(3)	38(3)	46(3)	1(2)	14(3)	-4(2)
C(32)	59(3)	51(3)	38(3)	5(3)	9(3)	5(3)
C(33)	57(3)	41(3)	45(3)	7(3)	6(3)	-1(3)
C(34)	43(3)	36(3)	46(3)	-5(2)	10(2)	2(2)
C(35)	36(2)	27(2)	39(3)	-1(2)	12(2)	3(2)
C(36)	28(2)	37(3)	29(3)	-5(2)	11(2)	1(2)
C(37)	26(2)	56(3)	51(4)	-12(3)	10(2)	-4(2)
C(38)	37(3)	65(4)	37(3)	-4(2)	23(3)	-1(2)
C(39)	30(2)	31(3)	31(2)	-5(2)	12(2)	-2(2)
C(40)	36(2)	24(2)	25(2)	-2(2)	9(2)	0(2)
C(41)	30(2)	33(3)	36(3)	1(2)	10(2)	-2(2)
C(42)	41(3)	31(2)	25(2)	-1(2)	6(2)	-1(2)
C(43)	45(3)	48(3)	48(3)	-9(3)	7(3)	-5(2)
C(44)	40(3)	34(3)	28(2)	-4(2)	12(2)	3(2)

C(45)	33(2)	33(3)	30(2)	0(2)	10(2)	1(2)
C(46)	27(2)	30(2)	29(2)	1(2)	6(2)	4(2)
C(47)	23(2)	23(2)	24(3)	-2(2)	9(2)	1(2)
C(48)	27(2)	33(3)	31(3)	-6(2)	10(2)	0(2)
C(49)	35(3)	52(3)	27(3)	-2(2)	14(3)	6(2)
C(50)	19(2)	36(3)	28(3)	-4(2)	9(2)	-3(2)
C(51)	33(2)	34(3)	49(3)	-6(2)	15(2)	0(2)
C(52)	41(3)	50(3)	62(4)	-12(3)	18(3)	2(2)
C(53)	61(4)	89(5)	65(4)	-33(4)	37(4)	-15(4)
C(54)	58(3)	79(5)	48(4)	-3(3)	35(3)	-11(3)
C(55)	41(3)	45(3)	39(3)	-6(2)	14(2)	-8(2)
C(56)	55(3)	37(3)	61(4)	10(3)	16(3)	-8(2)
N(1)	37(2)	36(2)	41(2)	-7(2)	15(2)	2(2)
N(2)	32(2)	41(2)	31(2)	2(2)	9(2)	7(2)
N(3)	29(2)	42(2)	39(2)	-8(2)	7(2)	0(2)
N(4)	33(2)	36(2)	41(2)	-2(2)	13(2)	1(2)
O(1)	34(2)	44(2)	41(2)	-9(2)	14(2)	4(1)
O(2)	88(3)	47(3)	78(3)	18(2)	15(3)	13(2)
O(3)	37(2)	41(2)	39(2)	-13(2)	13(2)	1(1)
O(4)	125(4)	50(3)	98(4)	20(3)	44(4)	-21(3)

Table 5. Hydrogen coordinates (x 104) and isotropic displacement parameters (Å 2x 103)
for d19432.

	x	y	z	U(eq)
H(1)	135	5688	1915	44
H(3)	65	5547	3607	55
H(4)	-733	5269	4211	67
H(5)	-1833	5442	3385	64
H(6)	-2197	5849	1901	54
H(9A)	-1048	7217	-160	59
H(9B)	-1526	8156	-266	59
H(9C)	-1825	7050	-490	59
H(10A)	-2030	7592	1472	62
H(10B)	-2404	7257	479	62
H(10C)	-2117	8371	702	62

H(11)	-704	8451	1132	38
H(13)	-1606	9104	1991	42
H(15A)	-1963	10085	2878	85
H(15B)	-1393	10666	3635	85
H(15C)	-1736	9707	3864	85
H(16)	-427	9363	4535	48
H(17)	368	8375	4312	45
H(21A)	1	7243	574	55
H(21B)	672	7013	1377	55
H(21C)	395	8136	1198	55
H(23)	1284	9033	3657	48
H(24)	2049	9496	4977	61
H(25)	2556	8284	6027	72
H(26)	2300	6600	5766	69
H(28)	1207	5567	3731	63
H(29)	5116	4316	8285	42
H(31)	5022	4548	6519	54
H(32)	4213	4810	5114	63
H(33)	3118	4617	4843	62
H(34)	2773	4118	5961	52
H(37A)	2985	2337	6541	68
H(37B)	2610	2658	7161	68
H(37C)	2898	1546	7216	68
H(38A)	4014	2655	9142	66
H(38B)	3507	1747	8783	66
H(38C)	3240	2869	8755	66
H(39)	4341	1493	8188	37
H(41)	3424	920	6363	41
H(43A)	3087	-41	5065	75
H(43B)	3673	-580	4882	75
H(43C)	3341	411	4355	75
H(44)	4649	734	5059	41
H(45)	5428	1692	6114	39
H(49A)	5028	2747	9477	56
H(49B)	5691	2982	9326	56
H(49C)	5415	1859	9227	56
H(51)	6347	1028	7868	46
H(52)	7100	488	7290	62

H(53)	7544	1603	6610	81
H(54)	7273	3288	6508	68
H(56)	6174	4456	7383	63

(c) X-ray data for compound 7a :

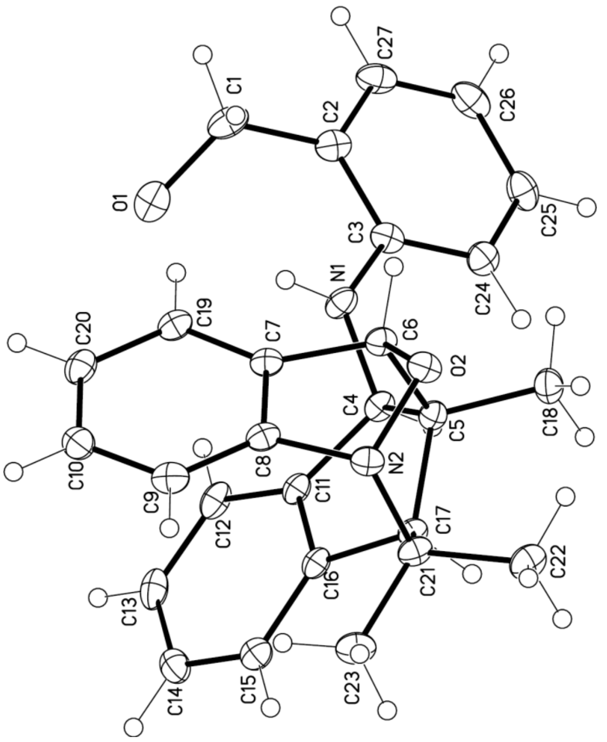

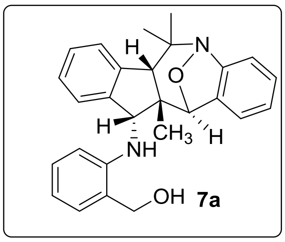

1. Crystal data and structure refinement for mo_180134lt_0m.

Identification code	mo_180134lt_0m	
Empirical formula	$C_{27} H_{28} N_2 O_2$	
Formula weight	412.51	
Temperature	100(2) K	
Wavelength	0.71073 Å	
Crystal system	Triclinic	
Space group	P -1	
Unit cell dimensions	a = 7.5024(14) Å	a= 93.783(5)°.
	b = 9.4863(17) Å	b= 95.871(5)°.
	c = 15.949(3) Å	g =
109.707(5)°.		
Volume	1056.9(3) Å ^{3}	
Z	2	
Density (calculated)	1.296 Mg/m^3	

Absorption coefficient	0.082 mm^{-1}
F(000)	440
Crystal size	0.14 x 0.12 x 0.09 mm^3
Theta range for data collection	1.291 to 26.362°.
Index ranges	-9<=h<=9, -9<=k<=11, -19<=l<=19
Reflections collected	24878
Independent reflections	4304 [R(int) = 0.0273]
Completeness to theta = 25.242°	99.9 %
Absorption correction	Semi-empirical from equivalents
Max. and min. transmission	0.9485 and 0.8638
Refinement method	Full-matrix least-squares on F^2
Data / restraints / parameters	4304 / 0 / 286
Goodness-of-fit on F^2	1.034
Final R indices [I>2sigma(I)]	R1 = 0.0421, wR2 = 0.1020
R indices (all data)	R1 = 0.0505, wR2 = 0.1087
Extinction coefficient	n/a
Largest diff. peak and hole	0.589 and -0.617 e.Å $^{-3}$

Table 2. Atomic coordinates (x 10^4) and equivalent isotropic displacement parameters (Å ^{2}x 10^3)
for mo_180134lt_0m. U(eq) is defined as one third of the trace of the orthogonalized U^{ij} tensor.

	x	y	z	U(eq)
O(1)	-1710(2)	2260(1)	2238(1)	22(1)
O(2)	17(2)	9318(1)	1728(1)	17(1)
N(1)	1023(2)	5162(1)	1955(1)	16(1)
N(2)	47(2)	9986(1)	2585(1)	16(1)
C(1)	-1812(2)	2364(2)	1343(1)	21(1)
C(2)	118(2)	2846(2)	1042(1)	17(1)
C(3)	1528(2)	4245(2)	1374(1)	15(1)
C(4)	2356(2)	6535(2)	2433(1)	14(1)
C(5)	2179(2)	8026(2)	2154(1)	14(1)
C(6)	84(2)	7826(2)	1846(1)	14(1)
C(7)	-1255(2)	7368(2)	2503(1)	14(1)
C(8)	-1176(2)	8703(2)	2943(1)	15(1)
C(9)	-2183(2)	8733(2)	3619(1)	18(1)
C(10)	-3282(2)	7355(2)	3858(1)	19(1)

C(11)	2205(2)	6634(2)	3365(1)	14(1)
C(12)	1943(2)	5474(2)	3878(1)	18(1)
C(13)	2104(2)	5798(2)	4747(1)	20(1)
C(14)	2548(2)	7276(2)	5093(1)	19(1)
C(15)	2803(2)	8437(2)	4575(1)	17(1)
C(16)	2577(2)	8117(2)	3697(1)	14(1)
C(17)	2894(2)	9157(2)	2991(1)	14(1)
C(18)	3358(2)	8522(2)	1426(1)	18(1)
C(19)	-2401(2)	6002(2)	2723(1)	16(1)
C(20)	-3409(2)	6013(2)	3410(1)	18(1)
C(21)	2088(2)	10490(2)	3004(1)	16(1)
C(22)	3265(2)	11738(2)	2516(1)	20(1)
C(23)	2091(2)	11204(2)	3890(1)	20(1)
C(24)	3322(2)	4629(2)	1101(1)	18(1)
C(25)	3727(2)	3640(2)	532(1)	20(1)
C(26)	2351(2)	2282(2)	200(1)	22(1)
C(27)	556(2)	1907(2)	460(1)	20(1)

Table 3. Bond lengths [Å] and angles [°] for mo_180134lt_0m.

O(1)-C(1)	1.433(2)
O(1)-H(1)	0.88(2)
O(2)-C(6)	1.4561(17)
O(2)-N(2)	1.4634(15)
N(1)-C(3)	1.3956(18)
N(1)-C(4)	1.4537(18)
N(1)-H(1A)	0.8800
N(2)-C(8)	1.4475(19)
N(2)-C(21)	1.5081(19)
C(1)-C(2)	1.504(2)
C(1)-H(28)	0.9900
C(1)-H(29)	0.9900
C(2)-C(27)	1.385(2)
C(2)-C(3)	1.416(2)
C(3)-C(24)	1.394(2)
C(4)-C(11)	1.501(2)
C(4)-C(5)	1.554(2)
C(4)-H(22)	1.0000

C(5)-C(18)	1.5344(19)
C(5)-C(6)	1.542(2)
C(5)-C(17)	1.5808(19)
C(6)-C(7)	1.509(2)
C(6)-H(19)	1.0000
C(7)-C(19)	1.383(2)
C(7)-C(8)	1.388(2)
C(8)-C(9)	1.381(2)
C(9)-C(10)	1.393(2)
C(9)-H(10)	0.9500
C(10)-C(20)	1.387(2)
C(10)-H(2)	0.9500
C(11)-C(12)	1.388(2)
C(11)-C(16)	1.394(2)
C(12)-C(13)	1.382(2)
C(12)-H(21)	0.9500
C(13)-C(14)	1.388(2)
C(13)-H(4)	0.9500
C(14)-C(15)	1.394(2)
C(14)-H(5)	0.9500
C(15)-C(16)	1.394(2)
C(15)-H(6)	0.9500
C(16)-C(17)	1.530(2)
C(17)-C(21)	1.574(2)
C(17)-H(20)	1.0000
C(18)-H(7)	0.9800
C(18)-H(8)	0.9800
C(18)-H(9)	0.9800
C(19)-C(20)	1.394(2)
C(19)-H(12)	0.9500
C(20)-H(11)	0.9500
C(21)-C(23)	1.526(2)
C(21)-C(22)	1.530(2)
C(22)-H(14)	0.9800
C(22)-H(13)	0.9800
C(22)-H(15)	0.9800
C(23)-H(18)	0.9800
C(23)-H(16)	0.9800

C(23)-H(17)	0.9800
C(24)-C(25)	1.391(2)
C(24)-H(24)	0.9500
C(25)-C(26)	1.381(2)
C(25)-H(25)	0.9500
C(26)-C(27)	1.386(2)
C(26)-H(26)	0.9500
C(27)-H(27)	0.9500
C(1)-O(1)-H(1)	106.9(13)
C(6)-O(2)-N(2)	104.33(10)
C(3)-N(1)-C(4)	124.38(12)
C(3)-N(1)-H(1A)	117.8
C(4)-N(1)-H(1A)	117.8
C(8)-N(2)-O(2)	101.07(10)
C(8)-N(2)-C(21)	112.08(11)
O(2)-N(2)-C(21)	106.61(10)
O(1)-C(1)-C(2)	112.87(12)
O(1)-C(1)-H(28)	109.0
C(2)-C(1)-H(28)	109.0
O(1)-C(1)-H(29)	109.0
C(2)-C(1)-H(29)	109.0
H(28)-C(1)-H(29)	107.8
C(27)-C(2)-C(3)	119.31(14)
C(27)-C(2)-C(1)	120.40(14)
C(3)-C(2)-C(1)	120.25(13)
C(24)-C(3)-N(1)	123.91(13)
C(24)-C(3)-C(2)	118.61(13)
N(1)-C(3)-C(2)	117.48(13)
N(1)-C(4)-C(11)	113.90(12)
N(1)-C(4)-C(5)	115.53(12)
C(11)-C(4)-C(5)	104.34(11)
N(1)-C(4)-H(22)	107.6
C(11)-C(4)-H(22)	107.6
C(5)-C(4)-H(22)	107.6
C(18)-C(5)-C(6)	108.36(12)
C(18)-C(5)-C(4)	109.89(11)
C(6)-C(5)-C(4)	111.13(11)
C(18)-C(5)-C(17)	114.16(12)

C(6)-C(5)-C(17)	109.32(11)
C(4)-C(5)-C(17)	103.96(11)
O(2)-C(6)-C(7)	100.70(11)
O(2)-C(6)-C(5)	107.10(11)
C(7)-C(6)-C(5)	114.59(12)
O(2)-C(6)-H(19)	111.3
C(7)-C(6)-H(19)	111.3
C(5)-C(6)-H(19)	111.3
C(19)-C(7)-C(8)	120.06(13)
C(19)-C(7)-C(6)	134.38(13)
C(8)-C(7)-C(6)	105.55(12)
C(9)-C(8)-C(7)	122.39(14)
C(9)-C(8)-N(2)	126.99(13)
C(7)-C(8)-N(2)	110.62(12)
C(8)-C(9)-C(10)	117.35(14)
C(8)-C(9)-H(10)	121.3
C(10)-C(9)-H(10)	121.3
C(20)-C(10)-C(9)	120.79(14)
C(20)-C(10)-H(2)	119.6
C(9)-C(10)-H(2)	119.6
C(12)-C(11)-C(16)	121.87(14)
C(12)-C(11)-C(4)	126.63(13)
C(16)-C(11)-C(4)	111.15(12)
C(13)-C(12)-C(11)	119.29(14)
C(13)-C(12)-H(21)	120.4
C(11)-C(12)-H(21)	120.4
C(12)-C(13)-C(14)	119.76(14)
C(12)-C(13)-H(4)	120.1
C(14)-C(13)-H(4)	120.1
C(13)-C(14)-C(15)	120.80(14)
C(13)-C(14)-H(5)	119.6
C(15)-C(14)-H(5)	119.6
C(14)-C(15)-C(16)	119.97(14)
C(14)-C(15)-H(6)	120.0
C(16)-C(15)-H(6)	120.0
C(11)-C(16)-C(15)	118.18(13)
C(11)-C(16)-C(17)	110.35(12)
C(15)-C(16)-C(17)	131.04(13)

C(16)-C(17)-C(21)	119.98(12)
C(16)-C(17)-C(5)	103.37(11)
C(21)-C(17)-C(5)	114.14(11)
C(16)-C(17)-H(20)	106.1
C(21)-C(17)-H(20)	106.1
C(5)-C(17)-H(20)	106.1
C(5)-C(18)-H(7)	109.5
C(5)-C(18)-H(8)	109.5
H(7)-C(18)-H(8)	109.5
C(5)-C(18)-H(9)	109.5
H(7)-C(18)-H(9)	109.5
H(8)-C(18)-H(9)	109.5
C(7)-C(19)-C(20)	118.21(14)
C(7)-C(19)-H(12)	120.9
C(20)-C(19)-H(12)	120.9
C(10)-C(20)-C(19)	121.14(14)
C(10)-C(20)-H(11)	119.4
C(19)-C(20)-H(11)	119.4
N(2)-C(21)-C(23)	105.73(12)
N(2)-C(21)-C(22)	107.10(12)
C(23)-C(21)-C(22)	107.45(12)
N(2)-C(21)-C(17)	111.83(11)
C(23)-C(21)-C(17)	114.30(12)
C(22)-C(21)-C(17)	110.06(12)
C(21)-C(22)-H(14)	109.5
C(21)-C(22)-H(13)	109.5
H(14)-C(22)-H(13)	109.5
C(21)-C(22)-H(15)	109.5
H(14)-C(22)-H(15)	109.5
H(13)-C(22)-H(15)	109.5
C(21)-C(23)-H(18)	109.5
C(21)-C(23)-H(16)	109.5
H(18)-C(23)-H(16)	109.5
C(21)-C(23)-H(17)	109.5
H(18)-C(23)-H(17)	109.5
H(16)-C(23)-H(17)	109.5
C(25)-C(24)-C(3)	120.48(14)
C(25)-C(24)-H(24)	119.8

C(3)-C(24)-H(24)	119.8
C(26)-C(25)-C(24)	121.09(15)
C(26)-C(25)-H(25)	119.5
C(24)-C(25)-H(25)	119.5
C(25)-C(26)-C(27)	118.56(14)
C(25)-C(26)-H(26)	120.7
C(27)-C(26)-H(26)	120.7
C(2)-C(27)-C(26)	121.91(15)
C(2)-C(27)-H(27)	119.0
C(26)-C(27)-H(27)	119.0

Symmetry transformations used to generate equivalent atoms:

Table 4. Anisotropic displacement parameters (Å ^{2}x 10^3) for mo_180134lt_0m. The anisotropic
displacement factor exponent takes the form: -2p^2[h^2 a*^{2}U^{11} + ... + 2 h k a* b* U^{12}]

	U^{11}	U^{22}	U^{33}	U^{23}	U^{13}	U^{12}
O(1)	20(1)	17(1)	31(1)	3(1)	6(1)	9(1)
O(2)	23(1)	13(1)	15(1)	2(1)	1(1)	8(1)
N(1)	13(1)	12(1)	22(1)	-2(1)	3(1)	4(1)
N(2)	18(1)	15(1)	16(1)	0(1)	0(1)	7(1)
C(1)	17(1)	14(1)	30(1)	-2(1)	-3(1)	5(1)
C(2)	20(1)	13(1)	19(1)	3(1)	-2(1)	8(1)
C(3)	20(1)	13(1)	15(1)	2(1)	0(1)	9(1)
C(4)	14(1)	11(1)	18(1)	1(1)	2(1)	5(1)
C(5)	14(1)	12(1)	16(1)	1(1)	3(1)	5(1)
C(6)	17(1)	10(1)	16(1)	1(1)	0(1)	5(1)
C(7)	12(1)	16(1)	16(1)	1(1)	-2(1)	7(1)
C(8)	13(1)	14(1)	17(1)	2(1)	-2(1)	6(1)
C(9)	16(1)	20(1)	21(1)	-2(1)	0(1)	10(1)
C(10)	14(1)	26(1)	18(1)	2(1)	2(1)	8(1)
C(11)	10(1)	13(1)	19(1)	2(1)	2(1)	5(1)
C(12)	14(1)	13(1)	27(1)	6(1)	3(1)	5(1)
C(13)	14(1)	22(1)	25(1)	13(1)	5(1)	7(1)
C(14)	15(1)	28(1)	16(1)	5(1)	3(1)	9(1)

C(15)	14(1)	17(1)	20(1)	2(1)	2(1)	7(1)
C(16)	9(1)	13(1)	19(1)	3(1)	2(1)	4(1)
C(17)	14(1)	11(1)	16(1)	1(1)	1(1)	4(1)
C(18)	20(1)	17(1)	19(1)	4(1)	6(1)	6(1)
C(19)	13(1)	15(1)	21(1)	1(1)	-1(1)	6(1)
C(20)	13(1)	19(1)	23(1)	5(1)	1(1)	5(1)
C(21)	17(1)	11(1)	20(1)	1(1)	-1(1)	5(1)
C(22)	22(1)	13(1)	26(1)	4(1)	0(1)	5(1)
C(23)	23(1)	14(1)	22(1)	-2(1)	-1(1)	9(1)
C(24)	21(1)	14(1)	19(1)	3(1)	4(1)	6(1)
C(25)	27(1)	20(1)	19(1)	7(1)	8(1)	12(1)
C(26)	36(1)	18(1)	17(1)	2(1)	5(1)	15(1)
C(27)	29(1)	13(1)	18(1)	-1(1)	-3(1)	8(1)

Table 5. Hydrogen coordinates (x 10^4) and isotropic displacement parameters ($Å^2$x 10^3)
for mo_180134lt_0m.

	x	y	z	U(eq)
H(1)	-1150(30)	1600(20)	2346(13)	32
H(1A)	-183	4891	2038	19
H(28)	-2444	3096	1200	25
H(29)	-2609	1371	1040	25
H(22)	3677	6560	2366	17
H(19)	-404	7132	1309	17
H(10)	-2127	9658	3908	22
H(2)	-3954	7333	4333	23
H(21)	1656	4468	3636	21
H(4)	1911	5013	5105	24
H(5)	2680	7498	5691	23
H(6)	3132	9447	4821	20
H(20)	4309	9631	3010	16
H(7)	2958	7704	962	28
H(8)	4716	8766	1629	28
H(9)	3154	9414	1220	28
H(12)	-2500	5081	2415	19

H(11)	-4195	5085	3574	22
H(14)	3132	11351	1920	30
H(13)	4612	12074	2760	30
H(15)	2803	12588	2557	30
H(18)	1551	12007	3846	29
H(16)	3405	11627	4178	29
H(17)	1320	10436	4215	29
H(24)	4275	5574	1306	21
H(25)	4972	3902	369	24
H(26)	2628	1620	-197	26
H(27)	-401	978	231	24

(d) X-ray data for compound 7e :

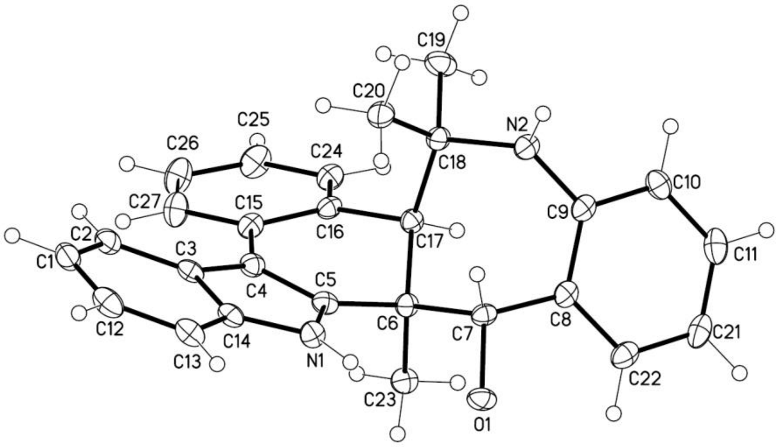

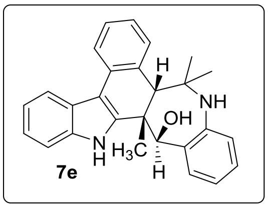

Table 1. Crystal data and structure refinement for mo_180316lt_0m_sq.

Identification code mo_180316lt_0m_sq

Empirical formula $C_{27} H_{25} N_2 O$

Formula weight 393.49

Temperature 100(2) K

Wavelength 0.71073 Å

Crystal system Monoclinic

Space group P 21/c

Unit cell dimensions a = 9.3848(5) Å a= 90°.

b = 17.3334(8) Å b= 94.091(2)°.

c = 12.7967(6) Å g = 90°.

Volume 2076.34(18) Å 3

Z 4
Density (calculated) 1.259 Mg/m3
Absorption coefficient 0.077 mm-1
F(000) 836
Crystal size 0.18 x 0.15 x 0.15 mm3
Theta range for data collection 1.981 to 26.417°.
Index ranges -11<=h<=11, -21<=k<=21, -16<=l<=15
Reflections collected 36450
Independent reflections 4257 [R(int) = 0.0652]
Completeness to theta = 25.242° 100.0 %
Absorption correction Semi-empirical from equivalents
Max. and min. transmission 0.9485 and 0.9086
Refinement method Full-matrix least-squares on F2
Data / restraints / parameters 4257 / 0 / 274
Goodness-of-fit on F2 1.020
Final R indices [I>2sigma(I)] R1 = 0.0481, wR2 = 0.1155
R indices (all data) R1 = 0.0700, wR2 = 0.1274
Extinction coefficient n/a
Largest diff. peak and hole 0.738 and -0.684 e.Å -3

Table 2. Atomic coordinates (x 104) and equivalent isotropic displacement parameters (Å 2x 103)
for mo_180316lt_0m_sq. U(eq) is defined as one third of the trace of the orthogonalized Uij tensor.

	x	y	z	U(eq)
O(1)	3288(1)	10669(1)	5610(1)	18(1)
N(1)	1479(2)	9324(1)	5849(1)	16(1)
N(2)	6469(2)	9147(1)	6677(1)	16(1)
C(1)	-1266(2)	7512(1)	6232(2)	21(1)
C(2)	-324(2)	7774(1)	7036(2)	19(1)
C(3)	564(2)	8402(1)	6860(1)	16(1)
C(4)	1681(2)	8806(1)	7459(1)	16(1)
C(5)	2214(2)	9349(1)	6817(1)	15(1)
C(6)	3469(2)	9860(1)	7128(1)	14(1)
C(7)	4223(2)	10135(1)	6159(1)	14(1)
C(8)	5681(2)	10467(1)	6464(1)	15(1)
C(9)	6778(2)	9944(1)	6759(1)	15(1)

C(10)	8150(2)	10212(1)	7039(1)	19(1)
C(11)	8432(2)	10996(1)	7035(2)	23(1)
C(12)	-1367(2)	7876(1)	5253(2)	22(1)
C(13)	-509(2)	8498(1)	5051(2)	20(1)
C(14)	468(2)	8747(1)	5849(1)	17(1)
C(15)	2309(2)	8716(1)	8530(1)	16(1)
C(16)	3674(2)	9034(1)	8763(1)	14(1)
C(17)	4503(2)	9388(1)	7894(1)	14(1)
C(18)	5471(2)	8770(1)	7396(1)	16(1)
C(19)	6413(2)	8382(1)	8271(2)	23(1)
C(20)	4667(2)	8145(1)	6751(2)	21(1)
C(21)	7362(2)	11514(1)	6732(2)	21(1)
C(22)	5988(2)	11251(1)	6452(1)	19(1)
C(23)	2938(2)	10570(1)	7726(1)	17(1)
C(24)	4251(2)	9020(1)	9797(1)	18(1)
C(25)	3532(2)	8672(1)	10579(2)	23(1)
C(26)	2217(2)	8332(1)	10341(2)	25(1)
C(27)	1601(2)	8366(1)	9327(2)	22(1)

Table 3. Bond lengths [Å] and angles [°] for mo_180316lt_0m_sq.

O(1)-C(7)	1.427(2)
N(1)-C(5)	1.375(2)
N(1)-C(14)	1.379(2)
N(1)-H(12)	0.8800
N(2)-C(9)	1.414(2)
N(2)-C(18)	1.509(2)
N(2)-H(11)	0.8800
C(1)-C(2)	1.384(3)
C(1)-C(12)	1.400(3)
C(1)-H(1)	0.9500
C(2)-C(3)	1.400(3)
C(2)-H(4)	0.9500
C(3)-C(14)	1.422(3)
C(3)-C(4)	1.436(3)
C(4)-C(5)	1.368(2)
C(4)-C(15)	1.461(3)
C(5)-C(6)	1.504(3)
C(6)-C(7)	1.546(2)

C(6)-C(23)	1.550(2)
C(6)-C(17)	1.561(2)
C(7)-C(8)	1.509(3)
C(7)-H(16)	1.0000
C(8)-C(22)	1.390(3)
C(8)-C(9)	1.403(3)
C(9)-C(10)	1.391(3)
C(10)-C(11)	1.384(3)
C(10)-H(15)	0.9500
C(11)-C(21)	1.382(3)
C(11)-H(2)	0.9500
C(12)-C(13)	1.381(3)
C(12)-H(3)	0.9500
C(13)-C(14)	1.392(3)
C(13)-H(25)	0.9500
C(15)-C(27)	1.395(3)
C(15)-C(16)	1.407(3)
C(16)-C(24)	1.393(3)
C(16)-C(17)	1.531(2)
C(17)-C(18)	1.569(2)
C(17)-H(20)	1.0000
C(18)-C(20)	1.528(3)
C(18)-C(19)	1.533(3)
C(19)-H(6)	0.9800
C(19)-H(5)	0.9800
C(19)-H(7)	0.9800
C(20)-H(9)	0.9800
C(20)-H(8)	0.9800
C(20)-H(10)	0.9800
C(21)-C(22)	1.390(3)
C(21)-H(14)	0.9500
C(22)-H(13)	0.9500
C(23)-H(18)	0.9800
C(23)-H(19)	0.9800
C(23)-H(17)	0.9800
C(24)-C(25)	1.385(3)
C(24)-H(24)	0.9500
C(25)-C(26)	1.382(3)

C(25)-H(23)	0.9500
C(26)-C(27)	1.383(3)
C(26)-H(21)	0.9500
C(27)-H(22)	0.9500

C(5)-N(1)-C(14)	108.90(15)
C(5)-N(1)-H(12)	125.6
C(14)-N(1)-H(12)	125.6
C(9)-N(2)-C(18)	120.71(14)
C(9)-N(2)-H(11)	119.6
C(18)-N(2)-H(11)	119.6
C(2)-C(1)-C(12)	121.12(18)
C(2)-C(1)-H(1)	119.4
C(12)-C(1)-H(1)	119.4
C(1)-C(2)-C(3)	119.39(18)
C(1)-C(2)-H(4)	120.3
C(3)-C(2)-H(4)	120.3
C(2)-C(3)-C(14)	118.31(17)
C(2)-C(3)-C(4)	135.57(17)
C(14)-C(3)-C(4)	106.05(16)
C(5)-C(4)-C(3)	107.47(16)
C(5)-C(4)-C(15)	119.76(17)
C(3)-C(4)-C(15)	132.73(17)
C(4)-C(5)-N(1)	109.71(16)
C(4)-C(5)-C(6)	124.03(16)
N(1)-C(5)-C(6)	126.19(15)
C(5)-C(6)-C(7)	111.32(14)
C(5)-C(6)-C(23)	108.97(14)
C(7)-C(6)-C(23)	109.44(14)
C(5)-C(6)-C(17)	107.29(14)
C(7)-C(6)-C(17)	111.40(14)
C(23)-C(6)-C(17)	108.32(14)
O(1)-C(7)-C(8)	113.22(14)
O(1)-C(7)-C(6)	107.11(14)
C(8)-C(7)-C(6)	111.64(14)
O(1)-C(7)-H(16)	108.2
C(8)-C(7)-H(16)	108.2
C(6)-C(7)-H(16)	108.2

C(22)-C(8)-C(9)	119.04(17)
C(22)-C(8)-C(7)	123.69(17)
C(9)-C(8)-C(7)	117.26(16)
C(10)-C(9)-C(8)	120.15(17)
C(10)-C(9)-N(2)	121.73(17)
C(8)-C(9)-N(2)	117.90(16)
C(11)-C(10)-C(9)	119.98(18)
C(11)-C(10)-H(15)	120.0
C(9)-C(10)-H(15)	120.0
C(21)-C(11)-C(10)	120.26(18)
C(21)-C(11)-H(2)	119.9
C(10)-C(11)-H(2)	119.9
C(13)-C(12)-C(1)	121.10(18)
C(13)-C(12)-H(3)	119.5
C(1)-C(12)-H(3)	119.5
C(12)-C(13)-C(14)	117.83(18)
C(12)-C(13)-H(25)	121.1
C(14)-C(13)-H(25)	121.1
N(1)-C(14)-C(13)	129.94(17)
N(1)-C(14)-C(3)	107.86(16)
C(13)-C(14)-C(3)	122.20(18)
C(27)-C(15)-C(16)	119.25(17)
C(27)-C(15)-C(4)	123.46(17)
C(16)-C(15)-C(4)	117.23(16)
C(24)-C(16)-C(15)	118.78(16)
C(24)-C(16)-C(17)	120.90(16)
C(15)-C(16)-C(17)	120.31(15)
C(16)-C(17)-C(6)	109.95(14)
C(16)-C(17)-C(18)	111.11(14)
C(6)-C(17)-C(18)	117.08(14)
C(16)-C(17)-H(20)	106.0
C(6)-C(17)-H(20)	106.0
C(18)-C(17)-H(20)	106.0
N(2)-C(18)-C(20)	106.37(14)
N(2)-C(18)-C(19)	106.55(15)
C(20)-C(18)-C(19)	108.66(16)
N(2)-C(18)-C(17)	110.76(14)
C(20)-C(18)-C(17)	115.23(15)

C(19)-C(18)-C(17)	108.89(15)
C(18)-C(19)-H(6)	109.5
C(18)-C(19)-H(5)	109.5
H(6)-C(19)-H(5)	109.5
C(18)-C(19)-H(7)	109.5
H(6)-C(19)-H(7)	109.5
H(5)-C(19)-H(7)	109.5
C(18)-C(20)-H(9)	109.5
C(18)-C(20)-H(8)	109.5
H(9)-C(20)-H(8)	109.5
C(18)-C(20)-H(10)	109.5
H(9)-C(20)-H(10)	109.5
H(8)-C(20)-H(10)	109.5
C(11)-C(21)-C(22)	120.08(18)
C(11)-C(21)-H(14)	120.0
C(22)-C(21)-H(14)	120.0
C(21)-C(22)-C(8)	120.47(18)
C(21)-C(22)-H(13)	119.8
C(8)-C(22)-H(13)	119.8
C(6)-C(23)-H(18)	109.5
C(6)-C(23)-H(19)	109.5
H(18)-C(23)-H(19)	109.5
C(6)-C(23)-H(17)	109.5
H(18)-C(23)-H(17)	109.5
H(19)-C(23)-H(17)	109.5
C(25)-C(24)-C(16)	121.15(18)
C(25)-C(24)-H(24)	119.4
C(16)-C(24)-H(24)	119.4
C(26)-C(25)-C(24)	119.97(18)
C(26)-C(25)-H(23)	120.0
C(24)-C(25)-H(23)	120.0
C(25)-C(26)-C(27)	119.71(18)
C(25)-C(26)-H(21)	120.1
C(27)-C(26)-H(21)	120.1
C(26)-C(27)-C(15)	121.04(19)
C(26)-C(27)-H(22)	119.5
C(15)-C(27)-H(22)	119.5

Symmetry transformations used to generate equivalent atoms:

Table 4. Anisotropic displacement parameters (Å 2x 103) for mo_180316lt_0m_sq. The anisotropic displacement factor exponent takes the form: -2p2[h2 a*2U11 + ... + 2 h k a* b* U12]

	U11	U22	U33	U23	U13	U12
O(1)	19(1)	20(1)	16(1)	5(1)	1(1)	6(1)
N(1)	17(1)	20(1)	12(1)	2(1)	1(1)	0(1)
N(2)	21(1)	15(1)	13(1)	-1(1)	6(1)	1(1)
C(1)	14(1)	20(1)	30(1)	-4(1)	4(1)	-1(1)
C(2)	15(1)	19(1)	22(1)	-2(1)	4(1)	2(1)
C(3)	14(1)	18(1)	17(1)	-3(1)	4(1)	5(1)
C(4)	16(1)	16(1)	15(1)	-1(1)	3(1)	2(1)
C(5)	16(1)	16(1)	12(1)	-1(1)	3(1)	3(1)
C(6)	15(1)	15(1)	12(1)	1(1)	3(1)	1(1)
C(7)	18(1)	14(1)	12(1)	2(1)	1(1)	2(1)
C(8)	20(1)	17(1)	9(1)	1(1)	4(1)	-1(1)
C(9)	21(1)	16(1)	10(1)	1(1)	5(1)	-1(1)
C(10)	16(1)	25(1)	16(1)	-1(1)	3(1)	0(1)
C(11)	21(1)	28(1)	20(1)	-4(1)	7(1)	-8(1)
C(12)	16(1)	28(1)	22(1)	-9(1)	-1(1)	2(1)
C(13)	16(1)	27(1)	16(1)	-2(1)	1(1)	4(1)
C(14)	14(1)	18(1)	18(1)	-3(1)	3(1)	4(1)
C(15)	20(1)	14(1)	15(1)	-1(1)	3(1)	0(1)
C(16)	19(1)	12(1)	13(1)	1(1)	3(1)	1(1)
C(17)	15(1)	15(1)	13(1)	1(1)	2(1)	0(1)
C(18)	18(1)	15(1)	16(1)	3(1)	5(1)	2(1)
C(19)	23(1)	28(1)	21(1)	9(1)	6(1)	9(1)
C(20)	22(1)	15(1)	27(1)	-2(1)	8(1)	1(1)
C(21)	28(1)	18(1)	18(1)	-2(1)	9(1)	-8(1)
C(22)	26(1)	16(1)	15(1)	1(1)	6(1)	1(1)
C(23)	20(1)	18(1)	14(1)	0(1)	3(1)	2(1)
C(24)	21(1)	16(1)	16(1)	1(1)	2(1)	-1(1)
C(25)	31(1)	25(1)	13(1)	3(1)	1(1)	-2(1)
C(26)	33(1)	28(1)	15(1)	4(1)	8(1)	-7(1)
C(27)	24(1)	25(1)	18(1)	0(1)	5(1)	-7(1)

Table 5. Hydrogen coordinates (x 104) and isotropic displacement parameters (Å 2x 10 3)
for mo_180316lt_0m_sq.

	x	y	z	U(eq)
H(12)	1629	9627	5316	20
H(11)	6864	8868	6200	19
H(1)	-1854	7079	6347	26
H(4)	-281	7529	7702	22
H(16)	4347	9680	5695	17
H(15)	8893	9857	7234	22
H(2)	9364	11178	7241	27
H(3)	-2037	7692	4719	27
H(25)	-583	8747	4388	23
H(20)	5172	9772	8245	17
H(6)	7067	8019	7963	35
H(5)	5809	8102	8737	35
H(7)	6966	8775	8672	35
H(9)	4095	8384	6168	32
H(8)	4038	7863	7197	32
H(10)	5354	7786	6474	32
H(14)	7566	12050	6714	26
H(13)	5254	11610	6251	23
H(18)	2238	10856	7271	26
H(19)	3750	10907	7930	26
H(17)	2490	10397	8354	26
H(24)	5154	9253	9968	21
H(23)	3943	8667	11279	28
H(21)	1737	8077	10871	30
H(22)	682	8148	9171	27

(VI) Coordinates for the Optimized Structures

D

C	-5.8191660799	-0.4598301982	2.7689033864
C	-4.8323713054	-1.4197035353	2.8858462900
C	-3.5902811593	-1.2626870663	2.2073165035
C	-3.3592064087	-0.1279799777	1.3639416837
C	-4.3695265754	0.8396947191	1.3040828140
C	-5.5756963301	0.6775376977	1.9836766109
H	-6.7641933602	-0.5767835757	3.2884647068
H	-4.9778461560	-2.2959665203	3.5088698697
C	-2.5734363647	-2.1730277928	2.5008796443
C	-2.1103236459	0.0838862243	0.6019033750
H	-4.2118640763	1.7213366673	0.6903979809
H	-6.3422334148	1.4421787640	1.8943737834
C	-1.7067949331	-3.0384679529	2.7836213345
H	-1.5757612007	0.9987840147	0.8574539032
H	-0.9654578297	-3.1431423705	3.5651952058
C	-1.6464781061	-0.6517866396	-0.4273022630
C	-0.3773630920	-0.2532648325	-1.1370524927
H	-0.5716739908	-0.0480320955	-2.1973269833
H	0.0841051146	0.6329444748	-0.6946971014
H	0.3537719737	-1.0714859174	-1.1080253690
C	-2.3563793426	-1.8459058284	-1.0081872480
H	-3.2769671295	-2.1012475317	-0.4792028939
H	-2.6142387104	-1.6497428860	-2.0569459700
H	-1.7045335729	-2.7285084371	-1.0174303914
Au	-1.7558414901	-4.5724483201	1.3179292193
P	-1.6946917012	-6.2371555460	-0.3257342125
O	-0.2989619380	-6.9982883620	-0.5797396321
O	-2.7118786609	-7.4793643328	-0.0924181465
O	-2.0155939064	-5.6005854604	-1.7648438570
C	0.4826190236	-7.4329086761	0.5155023958
C	1.4241292194	-6.5561089500	1.0464759717
C	0.3252944907	-8.7313061872	0.9866596357
C	2.2280006371	-6.9975577604	2.0984438099
H	1.5347603602	-5.5611901081	0.6263111185

C	1.1395140590	-9.1578051113	2.0373526012
H	-0.4077852032	-9.3871381845	0.5293888912
C	2.0851891677	-8.2941868147	2.5946551084
H	2.9727980010	-6.3297214736	2.5207582612
H	1.0363902199	-10.1698805257	2.4165206572
H	2.7166549821	-8.6349213986	3.4093356151
C	-3.9626350499	-7.3057314356	0.5290998166
C	-4.8330231222	-6.2885335395	0.1377576131
C	-4.3011689646	-8.2225619653	1.5198184395
C	-6.0760911958	-6.1925304701	0.7695607510
H	-4.5579451243	-5.5973666889	-0.6525845036
C	-5.5477310621	-8.1156219527	2.1348549069
H	-3.5971394005	-9.0021906450	1.7906329401
C	-6.4348570551	-7.1018349567	1.7638961041
H	-6.7663730324	-5.4097207034	0.4695140362
H	-5.8251953271	-8.8274457381	2.9062299304
H	-7.4052397933	-7.0269697617	2.2448356188
C	-1.9923722397	-6.3436755757	-2.9799089045
C	-0.8426687883	-6.2923804790	-3.7586946881
C	-3.1303294446	-7.0392013765	-3.3711321514
C	-0.8362431139	-6.9727827020	-4.9767200691
H	0.0213735278	-5.7337580034	-3.4157385237
C	-3.1054726958	-7.7161222908	-4.5916971722
H	-4.0126418076	-7.0506274248	-2.7407754290
C	-1.9629573388	-7.6847639046	-5.3922513610
H	0.0527064954	-6.9454428002	-5.5993415970
H	-3.9838408936	-8.2667672076	-4.9141434216
H	-1.9510524841	-8.2134059975	-6.3403337307

TS-D-E

C	0.3685388601	-1.6125536070	-0.8391399970
C	-0.4911158084	-2.1613255189	0.1097041742
C	-1.3845686718	-1.3301217254	0.7926395574
C	-1.4313338589	0.0665092776	0.5320270142
C	-0.5573286564	0.5901708764	-0.4231046548
C	0.3359563439	-0.2425448958	-1.1020137150

H	1.0631460536	-2.2532388141	-1.3732021704
H	-0.4680437791	-3.2256384632	0.3214302106
C	-2.3160623011	-1.7674968195	1.8159540740
C	-2.4408715108	0.8959100490	1.2380391920
H	-0.5811469114	1.6529678826	-0.6427959892
H	1.0059351881	0.1827961628	-1.8431567743
C	-3.1250750157	-1.1384908415	2.5544300089
H	-3.4146039635	0.9733527057	0.7512426187
H	-3.8745283583	-0.8457779120	3.2601215223
C	-2.2374562865	1.6037969615	2.3791131326
C	-3.3374012542	2.4492863985	2.9646121324
H	-3.0418135262	3.5059661648	2.9636047057
H	-4.2732540885	2.3591185072	2.4070516877
H	-3.5236796771	2.1859307855	4.0133702371
C	-0.9190083634	1.6359271482	3.1027060816
H	-0.1814039449	0.9634709894	2.6619551054
H	-0.5095514894	2.6537423628	3.0848229520
H	-1.0494503605	1.3756404205	4.1596616236
Au	-2.7209900491	-3.7312465939	2.5816400515
P	-3.0192210549	-5.9178711266	3.3890059805
O	-1.6477452450	-6.7843997585	3.4321737579
O	-3.5536108008	-6.0910387784	4.8981098107
O	-4.1016896255	-6.7334594094	2.5364471029
C	-0.6385471014	-6.6451952662	2.4626005931
C	-0.9319317778	-6.6863054224	1.0992185013
C	0.6666389814	-6.5210268137	2.9319011874
C	0.1236430212	-6.5984043141	0.1862466330
H	-1.9541452501	-6.8082610482	0.7545469605
C	1.7070322708	-6.4410155693	2.0077497931
H	0.8514412489	-6.4983245591	4.0006654750
C	1.4387541136	-6.4782554704	0.6363838066
H	-0.0890780808	-6.6405158776	-0.8779260820
H	2.7298644386	-6.3514712793	2.3614959923
H	2.2546552335	-6.4231295983	-0.0775946905
C	-2.9866607363	-5.3482608088	5.9580425503
C	-3.6023473069	-4.1568122503	6.3299711883
C	-1.8806250045	-5.8556097149	6.6310677422
C	-3.0787745882	-3.4441174828	7.4101556579

H	-4.4856540606	-3.8172514691	5.7979313689
C	-1.3695531855	-5.1300984415	7.7087233032
H	-1.4466497377	-6.8005101983	6.3232111102
C	-1.9629915499	-3.9271065129	8.0960481945
H	-3.5518640367	-2.5177739174	7.7217070084
H	-0.5096282558	-5.5120349527	8.2504772201
H	-1.5624226706	-3.3718498025	8.9386541536
C	-4.5282966759	-8.0517417084	2.8761466784
C	-3.7757619206	-9.1398218279	2.4512805942
C	-5.7181520803	-8.1923641681	3.5791477100
C	-4.2372502442	-10.4216399848	2.7552452470
H	-2.8565151504	-8.9916050534	1.8955753461
C	-6.1654393329	-9.4809231164	3.8722391056
H	-6.2757940090	-7.3141551086	3.8853048715
C	-5.4268112717	-10.5931277306	3.4642234167
H	-3.6634268328	-11.2850968232	2.4334393556
H	-7.0928125465	-9.6126182750	4.4211518524
H	-5.7796146318	-11.5929544964	3.6970488164

E

C	1.1534854093	0.3395315054	2.5095069896
C	0.3232734750	-0.7684855969	2.4276280550
C	-0.8888369158	-0.6519391553	1.7130060460
C	-1.2508716115	0.5845993410	1.0962559360
C	-0.4292126157	1.6962937103	1.2125970421
C	0.7793311827	1.5544565144	1.9064856476
H	2.0995255249	0.2756259032	3.0373635561
H	0.5956704894	-1.7132991072	2.8890152954
C	-1.9154820530	-1.6234886022	1.5160760454
C	-2.5813214516	0.4329316088	0.4484889790
H	-0.6956431406	2.6469594726	0.7615208692
H	1.4512467785	2.4054970096	1.9778865093
C	-2.9748037285	-1.0041291791	0.7467442025
H	-2.7704301826	0.8674886633	-0.5293611638
H	-3.6181681495	-1.5999078977	0.1101186371
C	-3.7574478260	0.2622432327	1.3822053044
C	-5.1302756818	0.4576096274	0.7712188284

H	-5.4409549042	1.5008531861	0.8994498422
H	-5.1458662297	0.2280633622	-0.2977701050
H	-5.8730649639	-0.1709956644	1.2731146333
C	-3.7009828325	0.5721418853	2.8620474162
H	-2.7232382152	0.3964711267	3.3101279734
H	-3.9546885861	1.6277319991	3.0116868870
H	-4.4434536998	-0.0240726102	3.4011559073
Au	-1.9698627471	-3.5576713786	2.1306754466
P	-2.2247170037	-5.8402128859	2.7857490864
O	-1.0791541563	-6.8829840868	2.2919670686
O	-2.3188596481	-6.2078083419	4.3574854022
O	-3.6368286677	-6.3658040636	2.2307092259
C	-0.3329826948	-6.6849745640	1.1178602072
C	-0.9479985377	-6.3388230771	-0.0846644765
C	1.0396382061	-6.8971759949	1.2062307634
C	-0.1527629734	-6.1889280643	-1.2245954148
H	-2.0245801868	-6.2077233654	-0.1387718420
C	1.8160189236	-6.7528777170	0.0578718112
H	1.4776235537	-7.1733317382	2.1594281393
C	1.2240373165	-6.3954607293	-1.1564409141
H	-0.6197056387	-5.9221096124	-2.1680824574
H	2.8872921599	-6.9212639234	0.1130356794
H	1.8347684246	-6.2867753122	-2.0472933716
C	-1.2895927423	-5.8220291760	5.2428655384
C	-1.3722211636	-4.5802286537	5.8667701548
C	-0.2587117562	-6.7172055909	5.5088922282
C	-0.3796199425	-4.2242768454	6.7818278274
H	-2.2080589280	-3.9218152891	5.6515961388
C	0.7246581479	-6.3469652434	6.4270225547
H	-0.2425785520	-7.6820971829	5.0140209168
C	0.6678616402	-5.1035166005	7.0603658790
H	-0.4336600445	-3.2630301451	7.2843492174
H	1.5332706786	-7.0361493351	6.6510054619
H	1.4342107596	-4.8246644665	7.7771541259
C	-4.1413874277	-7.6777483956	2.4401263486
C	-3.8149992862	-8.6761975536	1.5295592385
C	-4.9984265103	-7.8953422035	3.5123821295
C	-4.3694590749	-9.9443020621	1.7087314470

H	-3.1483008538	-8.4656901485	0.7002242040
C	-5.5447093093	-9.1682518328	3.6761963601
H	-5.2284441532	-7.0855087375	4.1959960598
C	-5.2313745202	-10.1911759114	2.7785774263
H	-4.1266007863	-10.7368483933	1.0074175116
H	-6.2165455029	-9.3576816378	4.5076764688
H	-5.6607285226	-11.1793616063	2.9114850196

F

C	-6.1583196247	-0.6128786696	0.9873851286
C	-5.1456155521	-1.3855587123	1.5444091506
C	-3.8190739717	-0.9267641442	1.4816051466
C	-3.5285600645	0.3029752704	0.8114809664
C	-4.5628280021	1.0851842382	0.2865730111
C	-5.8718874222	0.6165835475	0.3670781206
H	-7.1885633425	-0.9578825213	1.0324192512
H	-5.3720129205	-2.3317185064	2.0297618232
C	-2.5579422011	-1.5518699008	1.8757957647
C	-2.0924252716	0.5855407864	0.9630671604
H	-4.3475016330	2.0342866697	-0.1989144062
H	-6.6819046269	1.1990995083	-0.0647663425
C	-1.5720786558	-0.6085551817	1.6754745351
H	-1.7800302398	1.6048821265	1.1738784061
C	-0.7949143731	-0.2945861556	0.4812318396
C	0.4748249114	0.5369525713	0.5703437086
H	0.6131569109	1.1470432172	-0.3294878200
H	0.4666721060	1.1927448030	1.4454269465
H	1.3440016784	-0.1265891708	0.6596562531
C	-0.8549333559	-1.2036720901	-0.7309996161
H	-1.8446511712	-1.6541155496	-0.8335468685
H	-0.6155103830	-0.6481158478	-1.6456502501
H	-0.1249057070	-2.0167565574	-0.6367328699
Au	-2.2480488424	-3.5268978453	2.2250975066
P	-1.8163723334	-5.8523043719	2.4618482309
O	-0.8136466672	-6.3470686259	3.6666939257
O	-3.0335049328	-6.9149805808	2.6941800793
O	-1.1648231244	-6.4683338158	1.1100072297

C	0.2117872911	-5.5416768491	4.1631311634
C	1.0398747474	-4.8087687193	3.3114556353
C	0.4101699673	-5.5386938622	5.5412537420
C	2.0739859240	-4.0486037902	3.8610120916
H	0.8825543670	-4.8279774016	2.2376018624
C	1.4523918402	-4.7823077619	6.0732493835
H	-0.2521002927	-6.1207584822	6.1732360923
C	2.2856860906	-4.0336652307	5.2384098604
H	2.7138508291	-3.4687929893	3.2021055766
H	1.6110404089	-4.7719375331	7.1476318333
H	3.0931616859	-3.4416009770	5.6582476726
C	-3.9401892238	-6.7279966156	3.7492600597
C	-5.0216097086	-5.8670046406	3.5773737762
C	-3.7589688379	-7.4528934749	4.9238079868
C	-5.9405333315	-5.7260427148	4.6184014235
H	-5.1323972577	-5.3208312544	2.6460533034
C	-4.6876468796	-7.3042747663	5.9541466460
H	-2.9035202133	-8.1132246318	5.0155593705
C	-5.7757456306	-6.4414640919	5.8052994644
H	-6.7854582034	-5.0543032097	4.4983003114
H	-4.5589567995	-7.8642727932	6.8759203427
H	-6.4941417606	-6.3275656524	6.6117686092
C	-0.7690139477	-7.8096682425	0.9586762085
C	0.5359870198	-8.1688301857	1.2832687793
C	-1.6718130627	-8.7264297832	0.4289217854
C	0.9427080982	-9.4868264903	1.0727116106
H	1.2149033717	-7.4288880601	1.6936260382
C	-1.2507550942	-10.0399449924	0.2238887738
H	-2.6807556650	-8.4087734054	0.1907299990
C	0.0530036873	-10.4234879233	0.5444469807
H	1.9587032884	-9.7782244989	1.3228797527
H	-1.9458027229	-10.7656040444	-0.1884845691
H	0.3755567256	-11.4476475613	0.3823134288

TS-F-In2

C	-3.3350589110	0.5476275962	4.3692262431
C	-2.8451585167	-0.5870496278	3.7459738221

C	-2.6338358051	-0.5745723838	2.3493044757
C	-2.9676150930	0.6117011111	1.6072402793
C	-3.4382094737	1.7646402012	2.2662501151
C	-3.6247019904	1.7237165880	3.6374105711
H	-3.5013338061	0.5414221051	5.4439545644
H	-2.6213404730	-1.4825737062	4.3206713386
C	-2.1882299343	-1.6001217434	1.4364599718
C	-2.5832363154	0.4020899768	0.2401505712
H	-3.6633430952	2.6675112098	1.7033593202
H	-4.0074832699	2.5968664321	4.1595317122
C	-2.0963022451	-0.9817763144	0.1872755845
H	-2.3946780341	1.1919815307	-0.4729979006
C	-2.9399179018	-1.0795955950	-0.9506727767
C	-2.5299835588	-0.5679712540	-2.3114291882
H	-3.3542343582	-0.0635814157	-2.8282001848
H	-1.6661401191	0.0983838173	-2.2582964812
H	-2.2401995563	-1.4292894386	-2.9297573039
C	-4.1666236192	-1.9547255783	-0.9287719929
H	-4.5935903090	-1.9917081908	0.0763979118
H	-4.9206760421	-1.6122245234	-1.6465535859
H	-3.8912504566	-2.9825952764	-1.2042314221
Au	-1.8892886217	-3.5603166694	1.8374593843
P	-1.4532598625	-5.8452835796	2.2847098034
O	-1.2410575261	-6.3160129055	3.8428416736
O	-2.5319509793	-6.9909359691	1.8268280790
O	-0.1365358100	-6.3870120205	1.5047172369
C	-0.8758031786	-5.4508136105	4.8749940396
C	0.1768931225	-4.5462767774	4.7376794903
C	-1.5790970440	-5.5707827115	6.0713289797
C	0.5141669449	-3.7350931286	5.8230192455
H	0.7193313995	-4.4697573680	3.8008208845
C	-1.2232086606	-4.7621186020	7.1494650924
H	-2.3921878580	-6.2861554818	6.1383599553
C	-0.1801197154	-3.8408489534	7.0279098990
H	1.3244527805	-3.0193135874	5.7201286423
H	-1.7681156244	-4.8485222025	8.0852356597
H	0.0887758506	-3.2079407836	7.8684032287
C	-3.8183496874	-7.0126945772	2.3841279132

C	-4.8004935699	-6.1540852444	1.8963450048
C	-4.0886018494	-7.9396253946	3.3883474345
C	-6.0853254975	-6.2271378670	2.4375071590
H	-4.5538176395	-5.4424828056	1.1152690433
C	-5.3784215241	-8.0049111321	3.9153553294
H	-3.2944319524	-8.5905935010	3.7378528773
C	-6.3764499443	-7.1494785338	3.4435665125
H	-6.8585632866	-5.5593392368	2.0692574806
H	-5.6021194138	-8.7253312792	4.6972262574
H	-7.3784207846	-7.2020310550	3.8595317992
C	0.4280370538	-7.6591143935	1.7053597528
C	1.4910866921	-7.7798259599	2.5960132220
C	-0.0407075828	-8.7451650450	0.9720497965
C	2.0970775035	-9.0255374592	2.7576804317
H	1.8321435609	-6.9101797041	3.1476917903
C	0.5747699711	-9.9856071494	1.1447656285
H	-0.8706028908	-8.6121395631	0.2874767158
C	1.6405969558	-10.1291047387	2.0348324219
H	2.9267477213	-9.1310332028	3.4508517651
H	0.2181775005	-10.8417428200	0.5792855021
H	2.1153746497	-11.0974599586	2.1637134018

In2

C	-1.6334046866	0.0354949169	5.2018727028
C	-1.7285620239	-0.9876546754	4.3050935897
C	-2.1510397448	-0.7192575753	2.9588168431
C	-2.4752743565	0.6729232000	2.5902797427
C	-2.3582394843	1.7151844815	3.5714054917
C	-1.9502401122	1.3968941188	4.8320787482
H	-1.3129315560	-0.1631008675	6.2216065822
H	-1.4852170321	-2.0069997617	4.5955849447
C	-2.3241212215	-1.5563882262	1.8662120635
C	-2.8519954151	0.6941069707	1.2739126755
H	-2.5939729255	2.7420811385	3.3020362605
H	-1.8560893160	2.1742978054	5.5864911037
C	-2.7758767545	-0.6758704223	0.7550662429
H	-3.1433631302	1.5717214826	0.7141255626

C	-3.0748181662	-1.0146603402	-0.5375766069
C	-3.5307749424	-0.0008632021	-1.5563776693
H	-4.4969292256	-0.3034205853	-1.9821980387
H	-3.6354064381	1.0073046229	-1.1595467012
H	-2.8243615714	0.0324379937	-2.3969394599
C	-2.9870155159	-2.4036205668	-1.1046825063
H	-2.6204487966	-3.1362839318	-0.3858538307
H	-3.9732399020	-2.7275626771	-1.4659413425
H	-2.3217054804	-2.4131749154	-1.9788055926
Au	-1.9725019351	-3.5501684740	1.9804869447
P	-1.5351566139	-5.8683285093	2.1399476882
O	-1.3878737753	-6.5560470860	3.6246470922
O	-2.6058302830	-6.9240539285	1.4964280918
O	-0.1960942396	-6.3073901949	1.3321562120
C	-0.8811038265	-5.8995202872	4.7449383399
C	0.2554178643	-5.0927944749	4.6772687752
C	-1.5321263070	-6.1361922630	5.9543944110
C	0.7354874563	-4.5058916334	5.8503392217
H	0.7553274315	-4.9220839006	3.7291112698
C	-1.0347930924	-5.5511146383	7.1171943092
H	-2.4132906884	-6.7690968488	5.9656244648
C	0.0973571237	-4.7340239906	7.0691406303
H	1.6150271007	-3.8703933048	5.8044102825
H	-1.5377101685	-5.7311251636	8.0629937545
H	0.4789559086	-4.2772609701	7.9773644136
C	-3.9303183527	-6.9564184522	1.9573814864
C	-4.8523382940	-6.0304422452	1.4760815239
C	-4.2958443561	-7.9558667557	2.8556959364
C	-6.1742861922	-6.1074249525	1.9174509546
H	-4.5328752335	-5.2648846110	0.7767642392
C	-5.6218985354	-8.0243503962	3.2827329352
H	-3.5461365595	-8.6585858684	3.2028475552
C	-6.5607105355	-7.1010139055	2.8179223673
H	-6.9009328726	-5.3863002248	1.5550820105
H	-5.9198898456	-8.8001744850	3.9822970056
H	-7.5909266022	-7.1558113266	3.1573545037
C	0.2987602754	-7.6229706124	1.2765297455
C	1.2268050813	-8.0363785433	2.2282835825

C	-0.1007889295	-8.4537829977	0.2337319339
C	1.7632375448	-9.3207198346	2.1314826969
H	1.5203663358	-7.3623474982	3.0258993385
C	0.4447130261	-9.7348726072	0.1492516641
H	-0.8251799951	-8.0954459576	-0.4891017991
C	1.3743467366	-10.1707923317	1.0952524991
H	2.4874200393	-9.6542061827	2.8692026445
H	0.1404838596	-10.3926810619	-0.6597621148
H	1.7956708292	-11.1692810038	1.0240210455

TS-In2-G

C	2.8365285672	-1.1459904433	1.5823837882
C	1.6303825729	-1.7795619121	1.7832189000
C	0.4293919780	-1.0182939634	1.7670659859
C	0.5086316443	0.3976833973	1.5218925840
C	1.7532618887	1.0223898656	1.3086574876
C	2.9020585368	0.2555617174	1.3489180595
H	3.7589968945	-1.7205863062	1.5995811019
H	1.5862893689	-2.8520483627	1.9555300068
C	-0.9192796585	-1.4085072822	1.9698516488
C	-0.8234209520	0.9117912037	1.4401985861
H	1.8091257803	2.0932991856	1.1248424561
H	3.8719581228	0.7236046081	1.2017647714
C	-1.7119170637	-0.2013609676	1.9028209242
H	-1.0352496012	1.9526326840	1.6567430682
C	-3.0572871629	-0.0461826028	2.1655198481
C	-3.9652350598	-1.2013891889	2.5042260866
H	-5.0139745797	-0.8918010320	2.4681565532
H	-3.8269482067	-2.0309207658	1.8066980363
H	-3.7637740023	-1.5838522022	3.5132960404
C	-3.7119127626	1.2987770550	2.2946587865
H	-3.0612568924	2.1382371228	2.0546563119
H	-4.6025738139	1.3662395071	1.6591983846
H	-4.0609680023	1.4199321927	3.3306491696
Au	-1.4566210838	-3.3263157546	2.3504872740
P	-2.1628840188	-5.5217876714	2.8601736320
O	-3.0877325504	-6.3321934349	1.7884884706

O	-1.0504113587	-6.6917972655	3.1651678566
O	-3.1466644289	-5.5220016771	4.1508654909
C	-2.6418219310	-6.4705653554	0.4619024591
C	-2.7944065802	-5.4138005010	-0.4320689992
C	-2.0949653931	-7.6901745399	0.0734113816
C	-2.3749414639	-5.5876098916	-1.7518077196
H	-3.2246128972	-4.4749522803	-0.0989851554
C	-1.6883105508	-7.8516614932	-1.2511542524
H	-1.9985797140	-8.4872597350	0.8029489802
C	-1.8244338969	-6.8024640544	-2.1625808291
H	-2.4808696222	-4.7671051253	-2.4549965737
H	-1.2615679521	-8.7988478230	-1.5687827419
H	-1.5014941582	-6.9316979386	-3.1914555793
C	0.2046305219	-6.4421633287	3.7187278067
C	0.3981453190	-5.4964747284	4.7254955206
C	1.2575231173	-7.2259153945	3.2496668851
C	1.6799600061	-5.3232142911	5.2501059061
H	-0.4321808182	-4.9022857369	5.0935705999
C	2.5283259917	-7.0507396639	3.7934520567
H	1.0671788840	-7.9536859748	2.4677492056
C	2.7447509977	-6.0972611439	4.7915852428
H	1.8384215892	-4.5809228859	6.0270328764
H	3.3525863834	-7.6585050276	3.4313763060
H	3.7375641509	-5.9601186977	5.2095090879
C	-3.7156179775	-6.6770067208	4.7185833135
C	-4.9609155374	-7.1103520287	4.2733393329
C	-3.0464019686	-7.3125574679	5.7605875692
C	-5.5460619312	-8.2157260666	4.8913739680
H	-5.4515898370	-6.5879632761	3.4597173201
C	-3.6438014062	-8.4171129245	6.3684109329
H	-2.0791658642	-6.9438285363	6.0855346571
C	-4.8912113567	-8.8701076982	5.9363150528
H	-6.5176513731	-8.5642836257	4.5533437186
H	-3.1309953307	-8.9217315214	7.1821891944
H	-5.3526786417	-9.7295496485	6.4137722879
C	-1.1965946293	0.9053194579	-0.5605918094
H	-0.1704894406	0.9059524310	-0.9090510969
C	-2.1551662698	1.9490781990	-0.8049384041

C	-3.3943840489	1.2499160345	-0.8963089169
C	-2.1010299103	3.3444450839	-0.8552272235
C	-4.6049326679	1.9809416576	-1.0296936798
C	-3.2898231322	4.0476840059	-1.0147808216
H	-1.1516388038	3.8693589070	-0.7812055062
C	-4.5249741293	3.3601181429	-1.0954052514
H	-5.5562827171	1.4624746271	-1.0949840852
H	-3.2756491833	5.1315914048	-1.0805161316
H	-5.4380434323	3.9385637752	-1.2140187703
N	-3.2445103750	-0.0854245557	-0.8300225247
O	-1.8381046202	-0.2801319687	-0.8037861867

G

C	2.6227274180	-0.9286234732	2.7794369607
C	1.4061622174	-1.6115960474	2.6799347270
C	0.3265831528	-0.9907803830	2.0463905161
C	0.4805879808	0.3121385107	1.5304409077
C	1.6835999538	0.9952128524	1.6389188526
C	2.7664647758	0.3613300943	2.2625975096
H	3.4696974649	-1.4098399620	3.2627141641
H	1.3053510057	-2.6161769247	3.0825348863
C	-1.0399812216	-1.4834659860	1.7612809917
C	-0.8449240435	0.7573339596	0.9675895400
H	1.7928704484	2.0045436585	1.2468430393
H	3.7206063486	0.8752005723	2.3451233057
C	-1.7145747446	-0.4979839260	1.1096259268
H	-1.2376690944	1.5558485445	1.6145450484
C	-3.0737054657	-0.5076937345	0.4157577071
C	-3.4911287981	-1.9013724483	-0.0765615417
H	-4.4271029410	-1.8198459432	-0.6381801553
H	-2.7221171286	-2.3180222180	-0.7318623218
H	-3.6423107397	-2.5846073170	0.7645812307
C	-4.1729773856	0.0605638155	1.3312446082
H	-3.9157210604	1.0465549826	1.7261898277
H	-5.1287201264	0.1399791509	0.8012113506
H	-4.3068545547	-0.6198079859	2.1771165159
Au	-1.6073143583	-3.3689895452	2.3054804406

P	-2.1541648309	-5.5871085720	2.9626801454
O	-3.1753433537	-6.4913774133	2.0659321126
O	-0.9090261259	-6.6530173723	3.0582177449
O	-2.9011315640	-5.6657119201	4.4017941606
C	-2.8729792516	-6.8168955287	0.7337611384
C	-3.2509323833	-5.9459676559	-0.2841800870
C	-2.2504254254	-8.0340793304	0.4661238850
C	-2.9874575312	-6.3053088948	-1.6071495998
H	-3.7390583009	-5.0086673071	-0.0392893853
C	-1.9968157964	-8.3807141341	-0.8609235665
H	-1.9778558811	-8.6860997895	1.2887687050
C	-2.3614907209	-7.5184132016	-1.8970830344
H	-3.2729159808	-5.6320223526	-2.4099963614
H	-1.5124395229	-9.3273526591	-1.0837659761
H	-2.1586970859	-7.7922112055	-2.9283694523
C	0.3925330948	-6.2919562278	3.4053339236
C	0.6575030882	-5.4745009972	4.5042825003
C	1.4219334538	-6.8370835108	2.6408697746
C	1.9858993121	-5.1985826507	4.8339719241
H	-0.1557729676	-5.0584935932	5.0902258084
C	2.7427747038	-6.5577142136	2.9869014369
H	1.1751408448	-7.4653756747	1.7914158259
C	3.0288793755	-5.7391069232	4.0821915081
H	2.2005720400	-4.5592396031	5.6854025042
H	3.5505083374	-6.9777627773	2.3944466374
H	4.0596894243	-5.5225208701	4.3461661397
C	-3.2599429105	-6.8736812995	5.0313079606
C	-4.5272994159	-7.4033415150	4.8080148175
C	-2.3585199980	-7.4730443467	5.9066339312
C	-4.8947942051	-8.5694428236	5.4798898274
H	-5.2019047229	-6.9077182615	4.1188326630
C	-2.7397501880	-8.6392806466	6.5707651369
H	-1.3791176357	-7.0335356573	6.0612759174
C	-4.0046945491	-9.1892123740	6.3589233002
H	-5.8806048275	-8.9936683694	5.3129867421
H	-2.0434081031	-9.1166176090	7.2543201337
H	-4.2963121355	-10.0975549701	6.8780092183
C	-0.9135044033	1.2573117037	-0.5094393972

H	0.0668782849	1.4286904129	-0.9584700304
C	-1.9104008935	2.3904846958	-0.6247901233
C	-3.1438421456	1.7471661013	-0.7654575418
C	-1.8310176848	3.7709980719	-0.5571374193
C	-4.3280927582	2.4646128236	-0.8602671121
C	-3.0217857699	4.5096768697	-0.6484749171
H	-0.8760583384	4.2768411417	-0.4402479908
C	-4.2495903898	3.8629546254	-0.7940148489
H	-5.2794176189	1.9606204418	-0.9968932540
H	-2.9857798920	5.5948385156	-0.6148366351
H	-5.1598105550	4.4508905948	-0.8740580003
N	-2.9629604041	0.3265544186	-0.8684614751
O	-1.5731458245	0.2369083771	-1.2945812596

TS-E-E$_{step}$

C	-4.7365066346	-1.5676798045	-1.7182171094
C	-4.0846507771	-2.0987348013	-0.6450093624
C	-2.9390495660	-1.4151118919	-0.1124330869
C	-2.4941437245	-0.1674437967	-0.7602037922
C	-3.2297409265	0.3596075716	-1.8791742709
C	-4.3096899650	-0.3319970081	-2.3346943286
H	-5.6071752024	-2.0720153228	-2.1275034602
H	-4.4162094547	-3.0262147177	-0.1875962835
C	-2.0880292023	-1.7820165628	0.9170616750
C	-1.3643320541	0.2771399323	-0.1361791688
H	-2.9146354215	1.2860416972	-2.3497438824
H	-4.8753937903	0.0435780099	-3.1827252912
C	-1.0116438705	-0.6801960476	0.9627647947
H	-0.8228149947	1.1915461266	-0.3393469858
C	-1.1795481857	-0.1281570658	2.3383678572
C	-2.3895928302	0.6811038088	2.6933725675
H	-2.1034573795	1.5611336836	3.2762185586
H	-2.9502721489	1.0001228009	1.8150807925
H	-3.0339041253	0.0731148725	3.3392531661
C	-0.4735345767	-0.7904765312	3.4830248888
H	0.5038093918	-1.1877196329	3.2036079865
H	-0.3689701139	-0.1121197904	4.3318218972

H	-1.0964121609	-1.6375501194	3.7982234989
Au	-2.0556248891	-3.5803882839	1.8767982519
P	-1.8047445120	-5.7385244497	2.8210152083
O	-1.8312645737	-5.9567537653	4.4307762032
O	-2.8345728099	-6.9143533340	2.3553767800
O	-0.3180368792	-6.2446810923	2.4619030338
C	-2.9521671172	-5.5654868175	5.1868757799
C	-3.0050885885	-4.2677635659	5.6887265042
C	-3.9444115257	-6.5018712370	5.4617375502
C	-4.0922924552	-3.8967818894	6.4813823467
H	-2.1977704929	-3.5751808530	5.4722230985
C	-5.0241172351	-6.1169287975	6.2579313156
H	-3.8574928859	-7.5077194132	5.0655020699
C	-5.1016617943	-4.8181253467	6.7646508660
H	-4.1453710920	-2.8894636976	6.8840536931
H	-5.8037822239	-6.8375855866	6.4859010279
H	-5.9436450279	-4.5271687164	7.3853987041
C	-3.5451363860	-6.8882938243	1.1469669558
C	-2.9164121986	-6.6032157965	-0.0645520591
C	-4.8984990207	-7.2101938477	1.2106984543
C	-3.6760904958	-6.6287395034	-1.2371023789
H	-1.8556945174	-6.3742588739	-0.0961483788
C	-5.6396311116	-7.2422695961	0.0303716346
H	-5.3486981988	-7.4330906148	2.1722734417
C	-5.0330732559	-6.9480735739	-1.1933266249
H	-3.1975531187	-6.4052467876	-2.1859783241
H	-6.6943603183	-7.4979193757	0.0691986016
H	-5.6154677225	-6.9747050954	-2.1092468828
C	0.2076918096	-7.5159590360	2.7946361950
C	0.8411455613	-7.6873014025	4.0204863637
C	0.1365358215	-8.5312716872	1.8474290130
C	1.4179028296	-8.9259254531	4.3031793196
H	0.8776166534	-6.8685403083	4.7304218760
C	0.7187601375	-9.7638209535	2.1452645968
H	-0.3595478750	-8.3562335662	0.8985698497
C	1.3579529799	-9.9623810199	3.3698979946
H	1.9163670987	-9.0777523414	5.2558273468
H	0.6724652585	-10.5667631434	1.4157579754

H	1.8109847622	-10.9228071201	3.5957425695
C	0.5552886600	3.6274456813	1.5181304815
H	0.2107439433	4.6490787878	1.4426561588
C	1.6977540955	2.9690478668	1.1204402867
C	1.4810561069	1.6192444455	1.5707698859
C	2.8936732003	3.3253930469	0.4337855831
C	2.4618640405	0.6155601342	1.3351469096
C	3.8155088748	2.3380438549	0.2134290396
H	3.0605343916	4.3443155523	0.1009549022
C	3.5956206530	0.9947371007	0.6633781196
H	2.3211093597	-0.4026641305	1.6790870326
H	4.7392145648	2.5656681767	-0.3084317530
H	4.3636542509	0.2531252204	0.4643862866
N	0.3022483415	1.5132899406	2.1958784154
O	-0.2705386876	2.7797122327	2.1368080104
H	-0.0140124671	-1.1157988622	0.8347028424

E_{step}

C	-3.1174055619	-1.4266124670	-1.8486208533
C	-2.6939710485	-2.0367908942	-0.7078609219
C	-1.6534524429	-1.4266057143	0.0802610282
C	-1.0723846419	-0.1518810426	-0.3878667489
C	-1.5498773720	0.4445003029	-1.6092332772
C	-2.5396540228	-0.1796058922	-2.3025035621
H	-3.9052116169	-1.8768626759	-2.4462185552
H	-3.1322431173	-2.9760537332	-0.3817444411
C	-1.0618487019	-1.8391246775	1.2559118182
C	-0.1122063351	0.2510655938	0.4900765091
H	-1.1182238161	1.3765061835	-1.9626560611
H	-2.9127328839	0.2562736247	-3.2254379608
C	0.0270053539	-0.8023047445	1.5632323381
H	0.5167491577	1.1268236770	0.3946410605
C	0.1940459600	-0.2760474698	3.0207545082
C	0.4871005178	-1.4164376053	4.0104692489
H	0.6624973758	-1.0402374225	5.0218123871
H	-0.3292586627	-2.1399201629	4.0343783426
H	1.3938876393	-1.9356130123	3.6887440689

C	1.3035730335	0.7935673034	3.0885568401
H	1.0492885884	1.6767567725	2.5003395242
H	1.4982261559	1.1080531513	4.1173528042
H	2.2304409458	0.3705796401	2.6917774287
Au	-1.3201797273	-3.7095751546	2.0276464414
P	-1.4399907027	-6.0187801658	2.5877009819
O	-1.2236024323	-6.5705705747	4.1028261649
O	-2.8095535065	-6.8268575356	2.2107176863
O	-0.2338297653	-6.7627453392	1.8174791272
C	-2.1039517954	-6.2119472837	5.1366249354
C	-1.8088219715	-5.0945883547	5.9127629070
C	-3.2108020408	-7.0157885010	5.3959733507
C	-2.6558620666	-4.7703771714	6.9736741918
H	-0.9216439156	-4.5092643302	5.6924922066
C	-4.0477378875	-6.6793190637	6.4602095910
H	-3.3973658654	-7.8862887859	4.7769126939
C	-3.7749291839	-5.5583630355	7.2468591971
H	-2.4340597753	-3.9059929893	7.5929845758
H	-4.9122827166	-7.2997225854	6.6771188503
H	-4.4286932516	-5.3055739340	8.0761891039
C	-3.6344963463	-6.5036004207	1.1288657493
C	-3.1136359387	-6.1504964329	-0.1163188032
C	-5.0074532484	-6.6080985245	1.3404637476
C	-3.9986056806	-5.8880235478	-1.1655654948
H	-2.0412490180	-6.0894493219	-0.2717845101
C	-5.8760337950	-6.3503849784	0.2813894567
H	-5.3746468277	-6.8923057118	2.3210476637
C	-5.3759350995	-5.9881095276	-0.9718903800
H	-3.6014211652	-5.6154596661	-2.1390247746
H	-6.9475418939	-6.4348492178	0.4369870252
H	-6.0574124007	-5.7922803078	-1.7941473996
C	-0.0214107450	-8.1628815288	1.8222447215
C	0.8307537606	-8.7085202518	2.7757836171
C	-0.6154106109	-8.9366932238	0.8306759179
C	1.0891208780	-10.0790039433	2.7326387442
H	1.2782731596	-8.0696060075	3.5289888545
C	-0.3479873674	-10.3059766447	0.8020856371
H	-1.2680921210	-8.4771682483	0.0963494800

C	0.5013904618	-10.8778354891	1.7502904414
H	1.7532460631	-10.5208357261	3.4693279593
H	-0.8044325644	-10.9231693201	0.0339719746
H	0.7073954728	-11.9434743870	1.7216528775
H	0.9788077354	-1.3296985580	1.3770079107
C	-1.5068556107	0.9969039087	4.5903576349
C	-2.8505285358	1.4503509758	4.3733703679
C	-0.8571323891	1.2272695940	5.8245521794
C	-3.1401583891	1.0646619997	3.0779853903
C	-3.5618161665	2.1367725029	5.3925846204
C	-1.5786500431	1.8978883651	6.7882481838
H	0.1570809825	0.9008713493	6.0100350025
H	-4.0033881897	1.1604596175	2.4347987702
C	-2.9163026355	2.3499660711	6.5836411369
H	-4.5770391985	2.4772430626	5.2225442482
H	-1.1080283044	2.0937869527	7.7470032297
H	-3.4213135155	2.8722369039	7.3891605355
N	-1.0893897334	0.3868063405	3.4614760760
O	-2.1030506541	0.4403179950	2.5356090296

TS-E_{step}-GH

C	-2.9678266356	0.7797910346	4.7960318815
C	-2.4862983898	-0.3498482242	4.1960892061
C	-2.0384673491	-0.2851525362	2.8361940816
C	-2.0686456207	0.9921573679	2.1310686057
C	-2.6147140645	2.1375539326	2.7822227245
C	-3.0422635244	2.0213218164	4.0793773207
H	-3.3068086380	0.7508599928	5.8270991313
H	-2.4330032163	-1.2933272195	4.7319086749
C	-1.6468539985	-1.3077614729	1.9817879108
C	-1.5859242576	0.8247199728	0.8347500633
H	-2.6820446509	3.0873931555	2.2598505594
H	-3.4499653582	2.8894064302	4.5901407494
C	-1.2784997255	-0.6789579920	0.6560755989
H	-1.9258430657	1.4374994808	0.0129189402
C	0.1600794447	-1.1545657470	0.1114639374
C	1.1082682886	-1.6912673728	1.2030332830

H	2.1174329055	-1.7734753215	0.7878323068
H	1.1411329555	-1.0444333196	2.0800786498
H	0.7836524528	-2.6814244495	1.5244064036
C	-0.0215111511	-2.2259249734	-0.9689539679
H	-0.6689809962	-1.8901324458	-1.7827930628
H	0.9442453079	-2.5364134603	-1.3778029619
H	-0.4930125561	-3.1006968480	-0.5125333032
Au	-1.7972096988	-3.2984473242	2.4293950650
P	-2.0693381081	-5.5753471600	3.0682181207
O	-3.5037171689	-6.0669946192	3.6328034228
O	-1.8145590199	-6.7038891366	1.9131321058
O	-1.1047013685	-5.9362152253	4.3068252601
C	-4.6801045327	-5.9309213728	2.8685858015
C	-5.4228574180	-4.7592036979	2.9800648215
C	-5.0950750698	-6.9975000383	2.0769349043
C	-6.6158246848	-4.6525924374	2.2627925578
H	-5.0766550424	-3.9591716513	3.6268628971
C	-6.2911260012	-6.8760900558	1.3683276555
H	-4.4948087824	-7.8996939507	2.0325478238
C	-7.0491011667	-5.7067395881	1.4570812232
H	-7.2089341010	-3.7464188326	2.3420587977
H	-6.6324969498	-7.7010909528	0.7500984791
H	-7.9801994227	-5.6205274995	0.9050824886
C	-0.8652578938	-6.5422565853	0.8988563381
C	0.4321078654	-6.1091070925	1.1761018430
C	-1.2596333719	-6.8857905167	-0.3924075849
C	1.3478659611	-6.0136983617	0.1250983384
H	0.7280352204	-5.8691846396	2.1925295575
C	-0.3312375630	-6.7923156640	-1.4281083014
H	-2.2753430997	-7.2256395601	-0.5660861769
C	0.9714090879	-6.3545123095	-1.1738428998
H	2.3620301479	-5.6843028473	0.3320339770
H	-0.6275227024	-7.0659306938	-2.4364535812
H	1.6916429265	-6.2904078124	-1.9838144634
C	-1.0965979575	-7.1940745878	4.9641014788
C	-1.8638777694	-7.3481103890	6.1131142850
C	-0.2778408117	-8.2068152130	4.4767412920
C	-1.8085563804	-8.5653718549	6.7924356848

H	-2.4869504883	-6.5317460646	6.4615905387
C	-0.2349635017	-9.4190536741	5.1673495437
H	0.3140887960	-8.0493945918	3.5816743692
C	-0.9980937396	-9.5999951705	6.3215152463
H	-2.4015482660	-8.7028798578	7.6915656832
H	0.3991457132	-10.2205097692	4.8003488223
H	-0.9594855350	-10.5450357184	6.8546932229
C	0.5270326489	2.0336915622	0.3201391121
H	0.6350204559	2.8085411150	1.0690974027
C	0.2086033677	2.1207273473	-1.0590179941
C	0.4030050625	0.8053424750	-1.5394849329
C	-0.2778150145	3.1394923112	-1.8943322429
C	0.1306211837	0.4792187154	-2.8692457366
C	-0.5569624390	2.8096145206	-3.2120212788
H	-0.4144442801	4.1503952812	-1.5251092440
C	-0.3551754356	1.4979767570	-3.6864624983
H	0.3079881383	-0.5137294750	-3.2619105630
H	-0.9216321472	3.5707038541	-3.8940117156
H	-0.5716365773	1.2755358165	-4.7268470365
N	0.9137229397	0.0015618195	-0.5112126708
O	1.1818530461	0.9050266475	0.5722481810
H	-2.0081026327	-1.0424196386	-0.0844087751

G-H

C	2.8095121223	-1.0112958271	2.4552394867
C	1.6253262216	-1.7131354081	2.3262065090
C	0.4676404579	-1.0161935493	1.8979836269
C	0.5198540481	0.3849839505	1.6361017775
C	1.7149756903	1.0790757597	1.7773751020
C	2.8507327256	0.3709919991	2.1796580868
H	3.7163915350	-1.5189679760	2.7677480404
H	1.5756624339	-2.7786319216	2.5319026260
C	-0.8394520410	-1.5193595980	1.6750004196
C	-0.8571729393	0.8829776634	1.3018152943
H	1.7787559930	2.1461292714	1.5864878210
H	3.7930566424	0.9007097394	2.2906017015
C	-1.6514691891	-0.4495071922	1.0498037529

H	-1.2731938899	1.3343625817	2.2146877647
C	-1.8770112968	-0.7753587277	-0.5523529388
C	-0.6854778290	-1.5029586728	-1.1933079006
H	-0.8679483413	-1.5866076653	-2.2682208313
H	0.2513022204	-0.9631043110	-1.0541863018
H	-0.5824065342	-2.5084939305	-0.7758228389
C	-3.1488056954	-1.6138459726	-0.7125780996
H	-4.0263262202	-1.1230028614	-0.2842726804
H	-3.3341193860	-1.7963874286	-1.7751797234
H	-3.0192880675	-2.5810357616	-0.2195469668
Au	-1.4715077801	-3.3818687091	2.2222789995
P	-2.1934527077	-5.5274504755	2.9934627645
O	-3.4391679206	-6.2654912030	2.2782508893
O	-1.0809586180	-6.7157461633	2.9431855915
O	-2.7134123526	-5.4024272602	4.5077525822
C	-3.4269461404	-6.5621082154	0.8995305075
C	-4.0238901648	-5.6603052794	0.0237212655
C	-2.8831301065	-7.7671654010	0.4653777357
C	-4.0619948564	-5.9736247752	-1.3362670619
H	-4.4678808497	-4.7480891366	0.4099649441
C	-2.9294276767	-8.0650599882	-0.8973539924
H	-2.4483036594	-8.4528634119	1.1840944855
C	-3.5127913864	-7.1710446663	-1.7973731988
H	-4.5307866740	-5.2849008325	-2.0327211385
H	-2.5142122170	-9.0030364899	-1.2536605090
H	-3.5484988242	-7.4126339087	-2.8552752887
C	0.2945205738	-6.4952175912	3.1049284333
C	0.7902785414	-5.7618269341	4.1824055454
C	1.1387396145	-7.0932636494	2.1729012738
C	2.1750081825	-5.6238276192	4.3171580879
H	0.1147667991	-5.3265910340	4.9122574810
C	2.5169955486	-6.9510211525	2.3255047213
H	0.7115400250	-7.6609538872	1.3530274437
C	3.0374621769	-6.2158990120	3.3941688451
H	2.5744847310	-5.0654676892	5.1587793338
H	3.1850533705	-7.4184570491	1.6084367032
H	4.1121087121	-6.1156520878	3.5119451599
C	-3.2944394217	-6.4801459877	5.2318985106

C	-4.6803644572	-6.5710092613	5.2772718622
C	-2.4659447926	-7.3657625460	5.9111151312
C	-5.2542115910	-7.5955457883	6.0302099822
H	-5.2887860468	-5.8555196119	4.7349936057
C	-3.0567037499	-8.3863146205	6.6583656122
H	-1.3878163747	-7.2592413473	5.8600401595
C	-4.4459615288	-8.5028763106	6.7177211336
H	-6.3353852583	-7.6829982083	6.0770385013
H	-2.4259969115	-9.0885062260	7.1950091486
H	-4.8988837948	-9.2986049302	7.3010198906
C	-1.0193157726	1.9194230761	0.1726484348
H	-0.2961521724	2.7362080063	0.2277035932
C	-2.4716993405	2.3354267215	0.0718388539
C	-3.0482938456	1.4083737768	-0.7997391269
C	-3.2356618907	3.3291224688	0.6636018843
C	-4.3961934877	1.4563830649	-1.1305117529
C	-4.6008451169	3.3871737380	0.3456389157
H	-2.7962637118	4.0541630376	1.3429777704
C	-5.1679756132	2.4641418573	-0.5370904064
H	-4.8309840483	0.7579432412	-1.8372949801
H	-5.2201968721	4.1672368289	0.7770671934
H	-6.2228204205	2.5380408448	-0.7835611981
N	-2.0400991881	0.5014305305	-1.2819997573
O	-0.8187942469	1.2523558401	-1.0882093371
H	-2.6627193938	-0.4091153376	1.4639494927

BRUKER

```
Current Data Parameters
NAME              20171128
EXPNO                    5
PROCNO                   1

F2 - Acquisition Parameters
Date_             20171128
Time                 15.22
INSTRUM              spect
PROBHD   5 mm DUL 13C-1
PULPROG               zg30
TD                   32768
SOLVENT              CDCl3
NS                      10
DS                       0
SWH               6410.256 Hz
FIDRES            0.195625 Hz
AQ               2.5559540 sec
RG                       4
DW                  78.000 usec
DE                    6.00 usec
TE                   300.0 K
D1              2.00000000 sec
TD0                      1

======== CHANNEL f1 ========
NUC1                    1H
P1                   10.00 usec
PL1                  -2.40 dB
SFO1           400.1528010 MHz

F2 - Processing parameters
SI                   16384
SF             400.1500167 MHz
WDW                     EM
SSB                      0
LB                    0.00 Hz
GB                       0
PC                    1.00
```

4-Me-alkyne sub

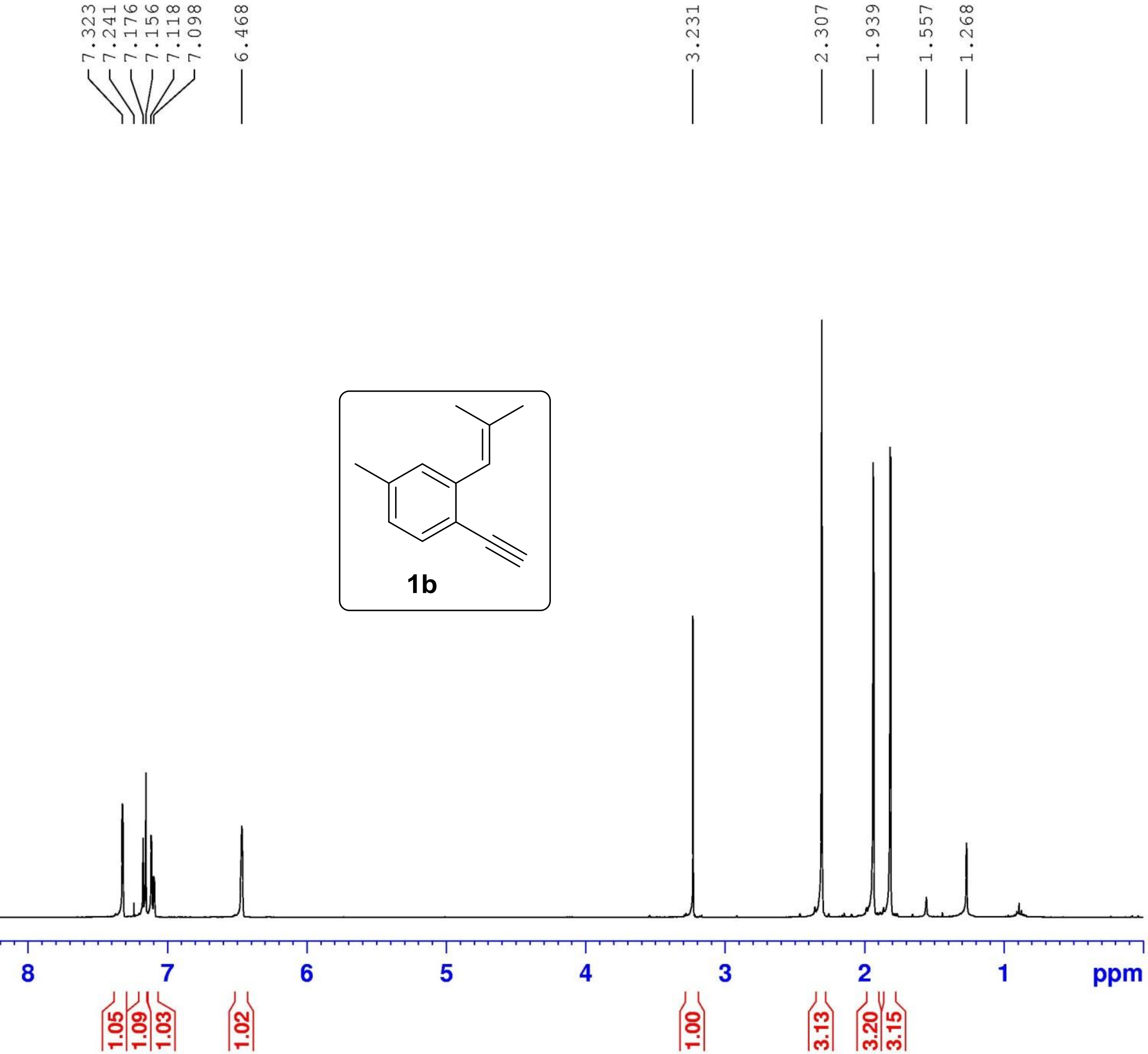

Current Data Parameters
NAME 20171128
EXPNO 6
PROCNO 1

F2 - Acquisition Parameters
Date_ 20171128
Time 15.24
INSTRUM spect
PROBHD 5 mm DUL 13C-1
PULPROG zgpg30
TD 65536
SOLVENT CDCl3
NS 77
DS 0
SWH 22727.273 Hz
FIDRES 0.346791 Hz
AQ 1.4418420 sec
RG 36
DW 22.000 usec
DE 6.00 usec
TE 300.0 K
D1 2.00000000 sec
d11 0.03000000 sec
DELTA 1.89999998 sec
TD0 1

======== CHANNEL f1 ========
NUC1 13C
P1 9.70 usec
PL1 -0.50 dB
SFO1 100.6288660 MHz

======== CHANNEL f2 ========
CPDPRG2 waltz16
NUC2 1H
PCPD2 90.00 usec
PL2 -2.40 dB
PL12 15.10 dB
PL13 18.10 dB
SFO2 400.1516010 MHz

F2 - Processing parameters
SI 32768
SF 100.6178052 MHz
WDW EM
SSB 0
LB 3.00 Hz
GB 0
PC 1.00

4-Me-alkyne sub

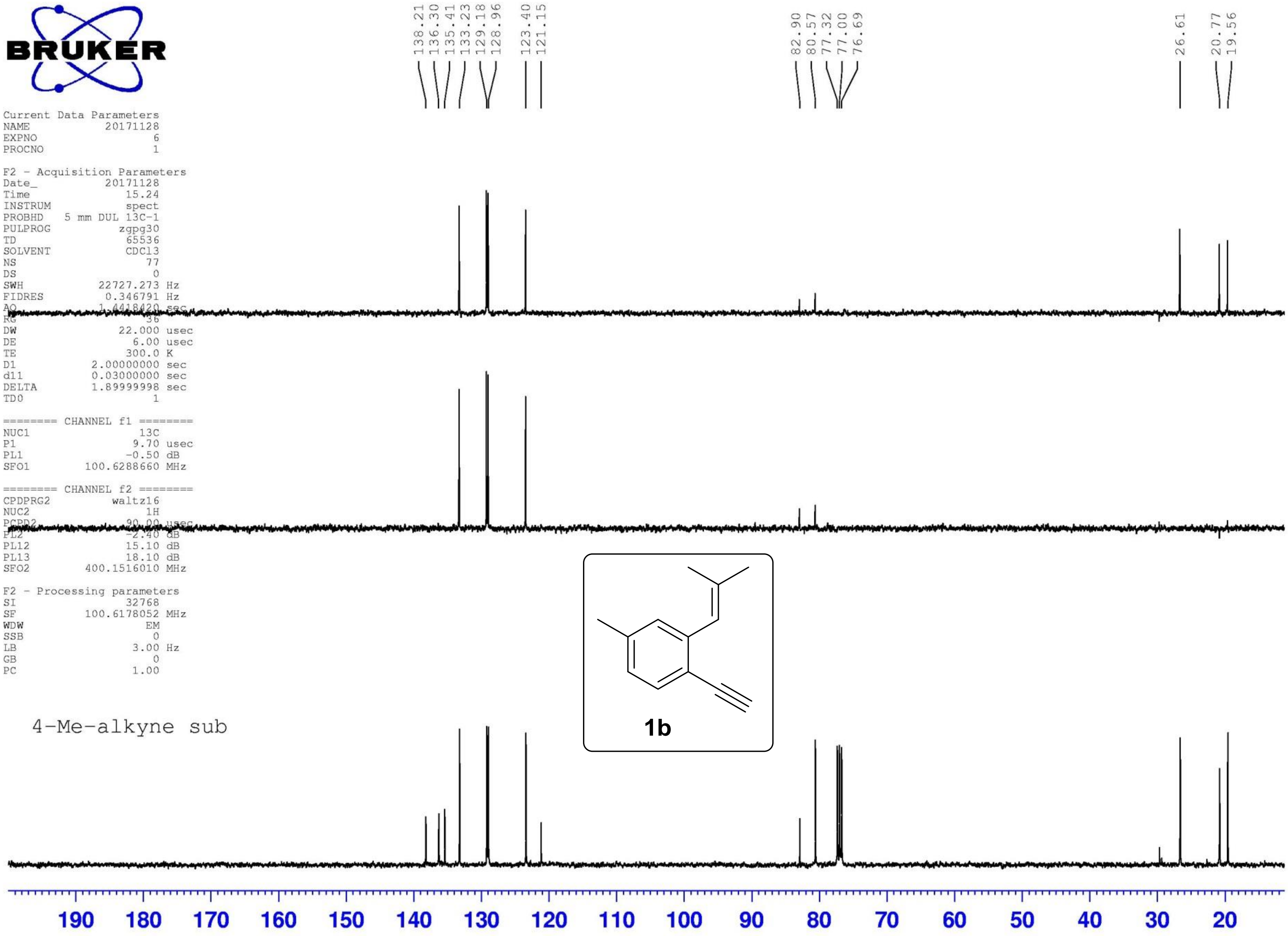

1b

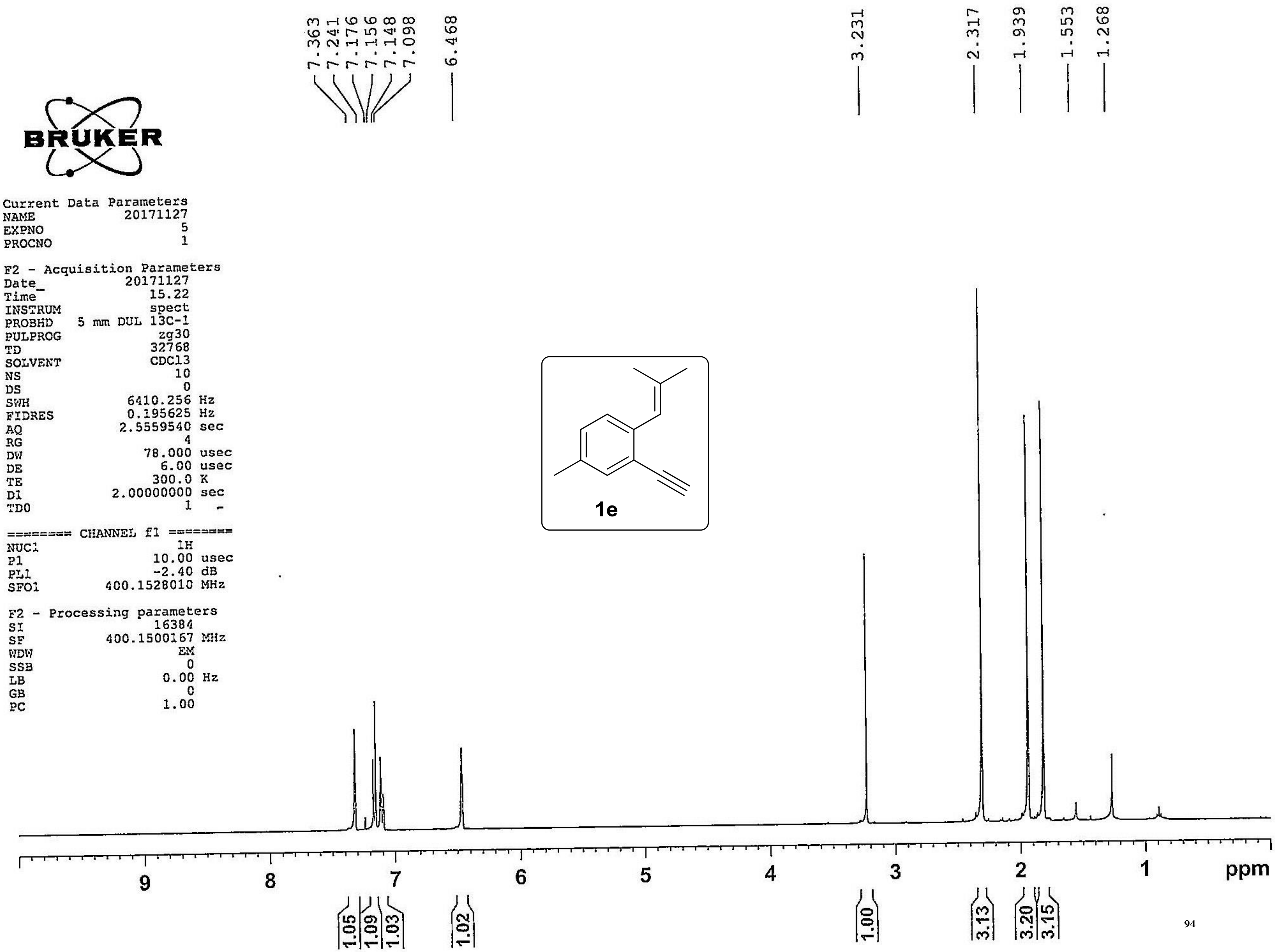
BRUKER
Current Data Parameters
NAME 20171127
EXPNO 5
PROCNO 1
F2 - Acquisition Parameters
Date_ 20171127
Time 15.22
INSTRUM spect
PROBHD 5 mm DUL 13C-1
PULPROG zg30
TD 32768
SOLVENT CDCl3
NS 10
DS 0
SWH 6410.256 Hz
FIDRES 0.195625 Hz
AQ 2.5559540 sec
RG 4
DW 78.000 usec
DE 6.00 usec
TE 300.0 K
D1 2.00000000 sec
TD0 1
======== CHANNEL f1 ========
NUC1 1H
P1 10.00 usec
PL1 -2.40 dB
SFO1 400.1528010 MHz
F2 - Processing parameters
SI 16384
SF 400.1500167 MHz
WDW EM
SSB 0
LB 0.00 Hz
GB 0
PC 1.00
7.363
7.241
7.176
7.156
7.148
7.098
6.468
3.231
2.317
1.939
1.553
1.268
1e
9
8
7
6
5
4
3
2
1
ppm
1.05
1.09
1.03
1.02
1.00
3.13
3.20
3.15

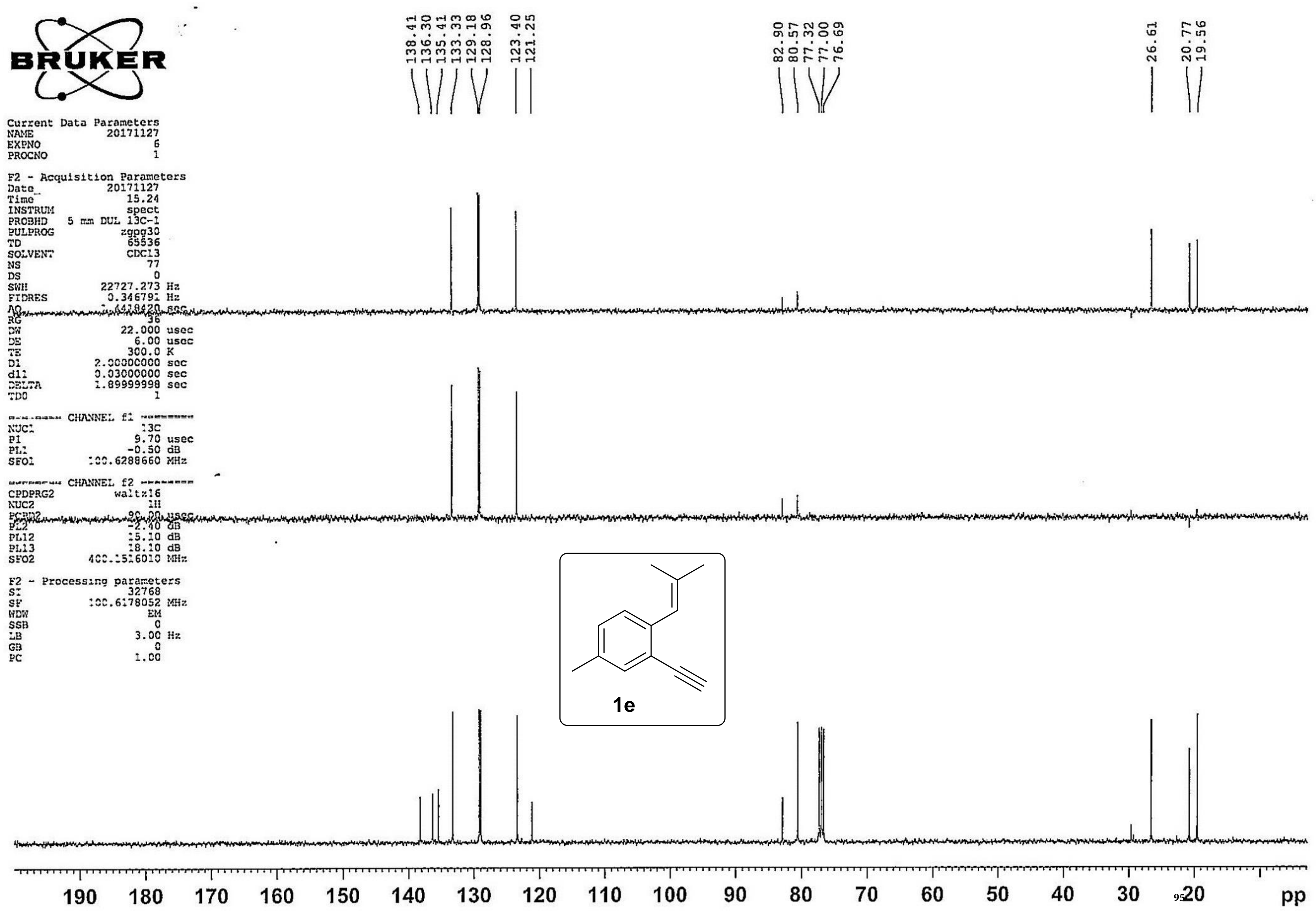
BRUKER
138.41
136.30
135.41
133.33
129.18
128.96
123.40
121.25
82.90
80.57
77.32
77.00
76.69
26.61
20.77
19.56
Current Data Parameters
NAME 20171127
EXPNO 6
PROCNO 1
F2 - Acquisition Parameters
Date_ 20171127
Time 15.24
INSTRUM spect
PROBHD 5 mm DUL 13C-1
PULPROG zgpg30
TD 65536
SOLVENT CDCl3
NS 77
DS 0
SWH 22727.273 Hz
FIDRES 0.346791 Hz
RG 36
DW 22.000 usec
DE 6.00 usec
TE 300.0 K
D1 2.00000000 sec
d11 0.03000000 sec
DELTA 1.89999998 sec
TD0 1
CHANNEL f1
NUC1 13C
P1 9.70 usec
PL1 -0.50 dB
SFO1 100.6288660 MHz
CHANNEL f2
CPDPRG2 waltz16
NUC2 1H
PL12 15.10 dB
PL13 18.10 dB
SFO2 400.1516010 MHz
F2 - Processing parameters
SI 32768
SF 100.6178052 MHz
WDW EM
SSB 0
LB 3.00 Hz
GB 0
PC 1.00
1e
190 180 170 160 150 140 130 120 110 100 90 80 70 60 50 40 30 20
pp

```
Current Data Parameters
NAME              20171108
EXPNO                    3
PROCNO                   1

F2 - Acquisition Parameters
Date_             20171108
Time                 15.42
INSTRUM              spect
PROBHD   5 mm DUL 13C-1
PULPROG               zg30
TD                   32768
SOLVENT              CDCl3
NS                      14
DS                       0
SWH               6410.256 Hz
FIDRES            0.195625 Hz
AQ               2.5559540 sec
RG                       4
DW                  78.000 usec
DE                    6.00 usec
TE                   300.0 K
D1             2.00000000 sec
TD0                      1

======== CHANNEL f1 ========
NUC1                    1H
P1                   10.00 usec
PL1                  -2.40 dB
SFO1           400.1528010 MHz

F2 - Processing parameters
SI                   16384
SF             400.1500168 MHz
WDW                     EM
SSB                      0
LB                    0.00 Hz
GB                       0
PC                    1.00
```

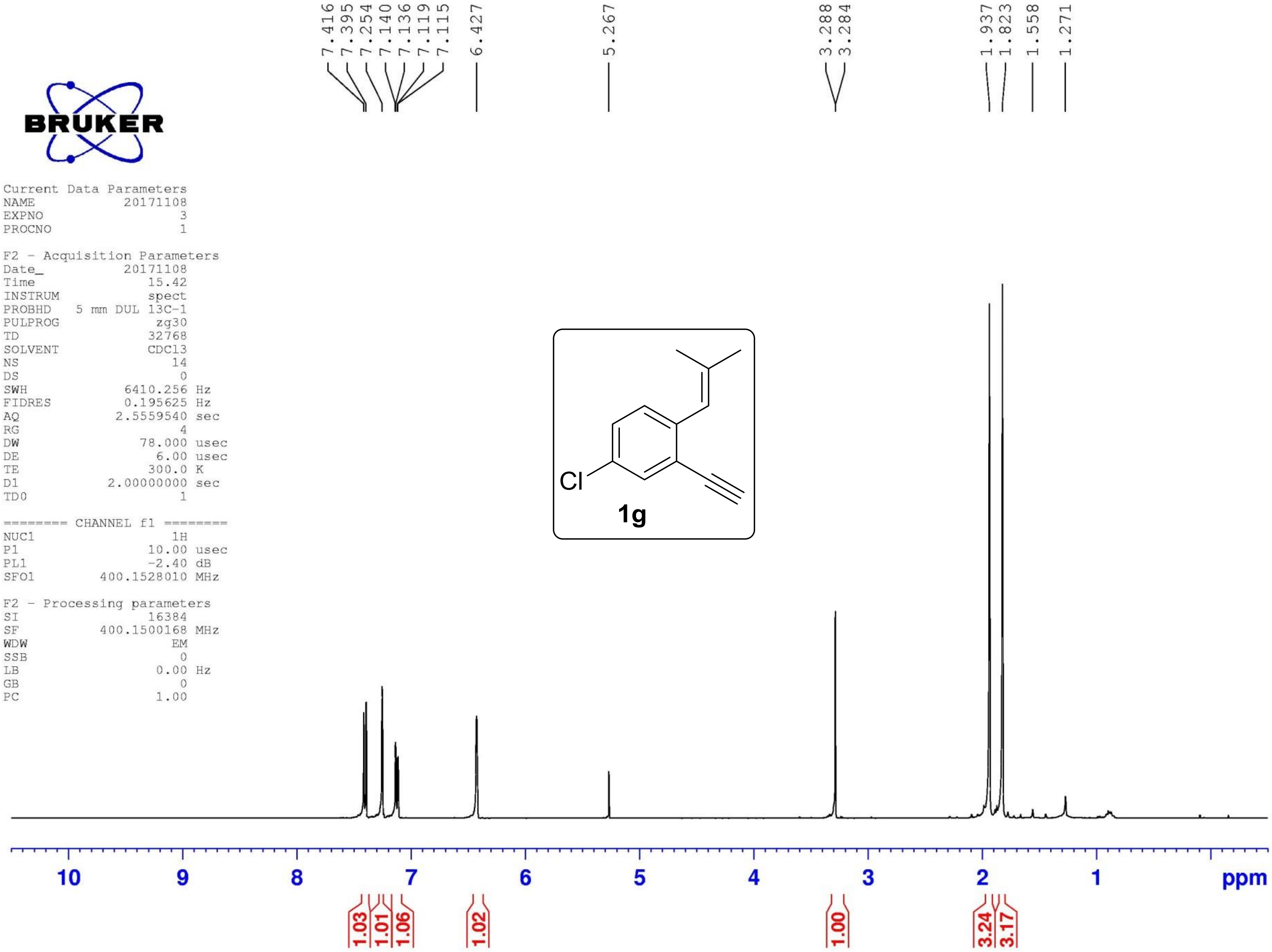

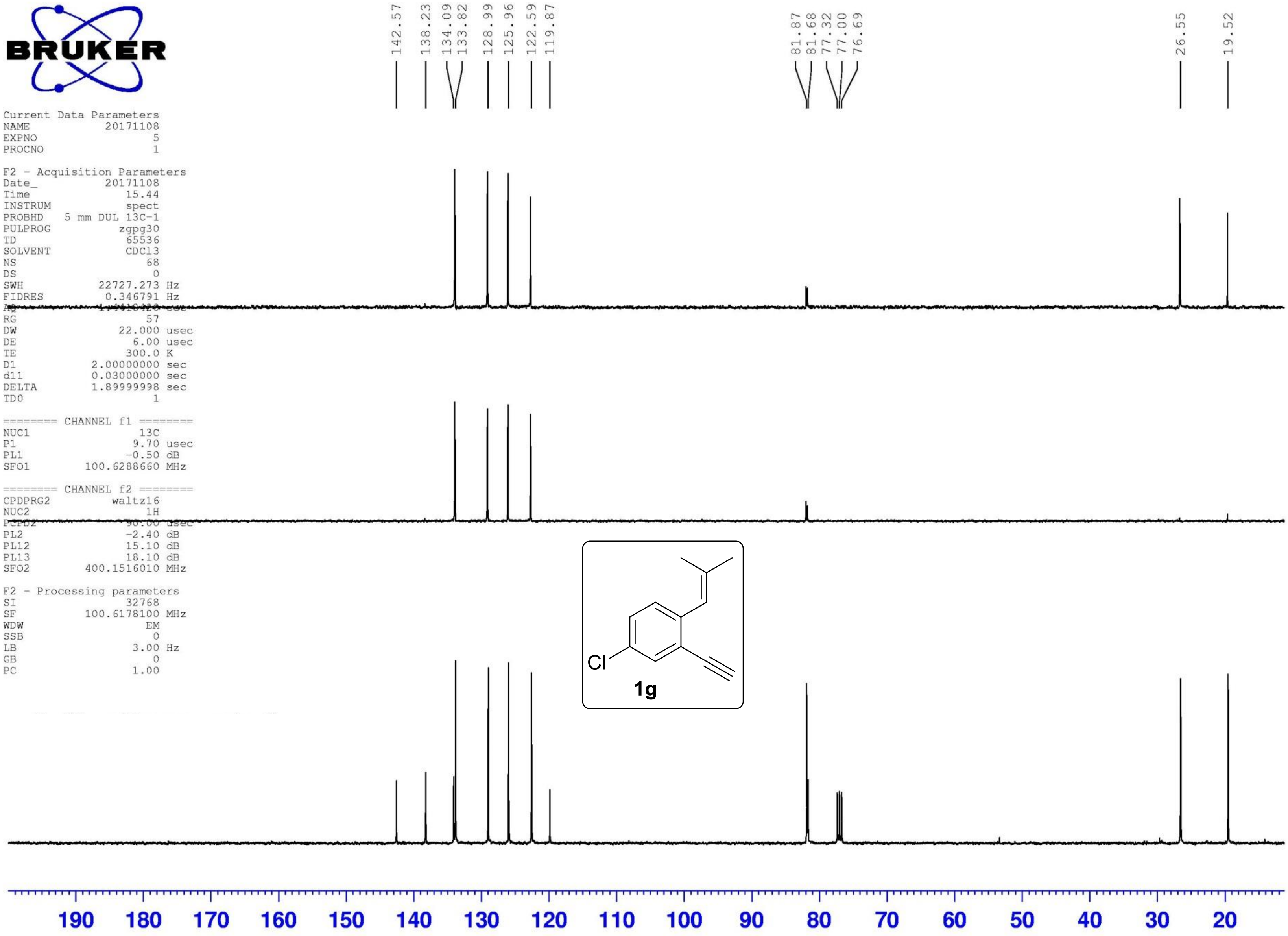
BRUKER
142.57
138.23
134.09
133.82
128.99
125.96
122.59
119.87
81.87
81.68
77.32
77.00
76.69
26.55
19.52
Current Data Parameters
NAME 20171108
EXPNO 5
PROCNO 1
F2 - Acquisition Parameters
Date_ 20171108
Time 15.44
INSTRUM spect
PROBHD 5 mm DUL 13C-1
PULPROG zgpg30
TD 65536
SOLVENT CDCl3
NS 68
DS 0
SWH 22727.273 Hz
FIDRES 0.346791 Hz
AQ [illegible] sec
RG 57
DW 22.000 usec
DE 6.00 usec
TE 300.0 K
D1 2.00000000 sec
d11 0.03000000 sec
DELTA 1.89999998 sec
TD0 1
======== CHANNEL f1 ========
NUC1 13C
P1 9.70 usec
PL1 -0.50 dB
SFO1 100.6288660 MHz
======== CHANNEL f2 ========
CPDPRG2 waltz16
NUC2 1H
PCPD2 90.00 usec
PL2 -2.40 dB
PL12 15.10 dB
PL13 18.10 dB
SFO2 400.1516010 MHz
F2 - Processing parameters
SI 32768
SF 100.6178100 MHz
WDW EM
SSB 0
LB 3.00 Hz
GB 0
PC 1.00
Cl
1g
190 180 170 160 150 140 130 120 110 100 90 80 70 60 50 40 30 20

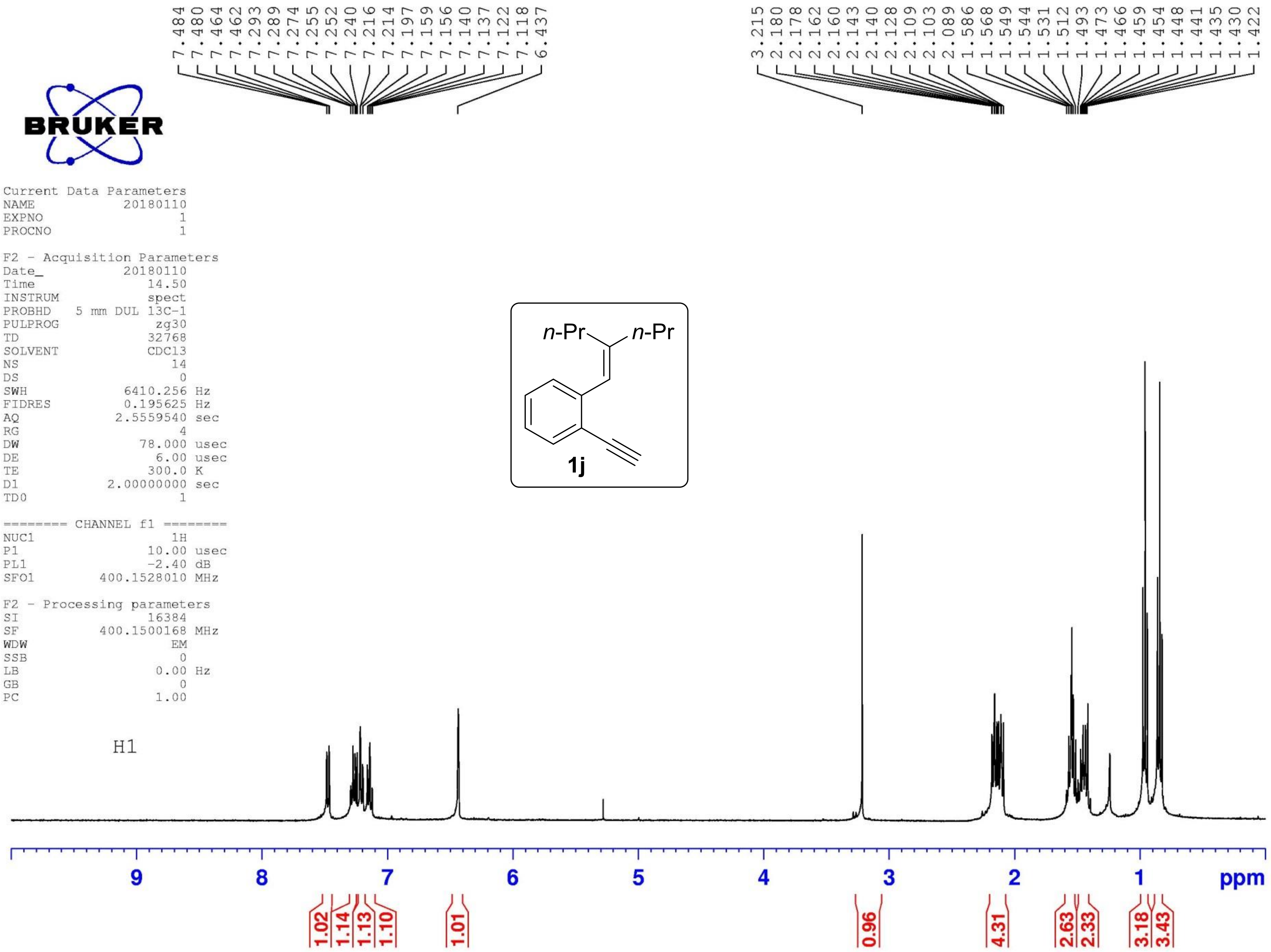
7.484
7.480
7.464
7.462
7.293
7.289
7.274
7.255
7.252
7.240
7.216
7.214
7.197
7.159
7.156
7.140
7.137
7.122
7.118
6.437
3.215
2.180
2.178
2.162
2.160
2.143
2.140
2.128
2.109
2.103
2.089
1.586
1.568
1.549
1.544
1.531
1.512
1.493
1.473
1.466
1.459
1.454
1.448
1.441
1.435
1.430
1.422
BRUKER
Current Data Parameters
NAME 20180110
EXPNO 1
PROCNO 1
F2 - Acquisition Parameters
Date_ 20180110
Time 14.50
INSTRUM spect
PROBHD 5 mm DUL 13C-1
PULPROG zg30
TD 32768
SOLVENT CDCl3
NS 14
DS 0
SWH 6410.256 Hz
FIDRES 0.195625 Hz
AQ 2.5559540 sec
RG 4
DW 78.000 usec
DE 6.00 usec
TE 300.0 K
D1 2.00000000 sec
TD0 1
======== CHANNEL f1 ========
NUC1 1H
P1 10.00 usec
PL1 -2.40 dB
SFO1 400.1528010 MHz
F2 - Processing parameters
SI 16384
SF 400.1500168 MHz
WDW EM
SSB 0
LB 0.00 Hz
GB 0
PC 1.00
H1
n-Pr
n-Pr
1j
9
8
7
6
5
4
3
2
1
ppm
1.02
1.14
1.13
1.10
1.01
0.96
4.31
2.63
2.33
3.18
3.43

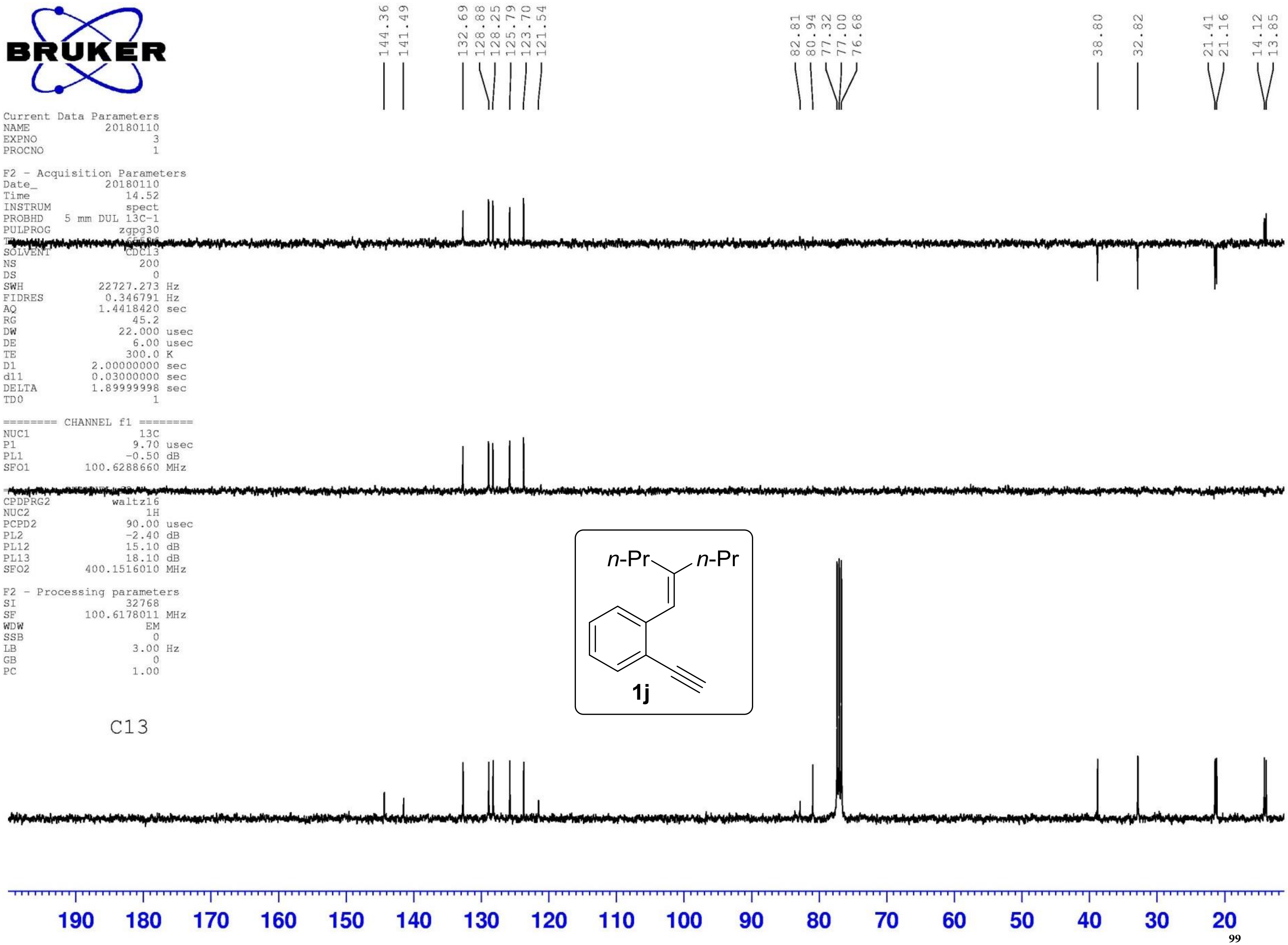
BRUKER
144.36
141.49
132.69
128.88
128.25
125.79
123.70
121.54
82.81
80.94
77.32
77.00
76.68
38.80
32.82
21.41
21.16
14.12
13.85
Current Data Parameters
NAME 20180110
EXPNO 3
PROCNO 1
F2 - Acquisition Parameters
Date_ 20180110
Time 14.52
INSTRUM spect
PROBHD 5 mm DUL 13C-1
PULPROG zgpg30
SOLVENT CDCl3
NS 200
DS 0
SWH 22727.273 Hz
FIDRES 0.346791 Hz
AQ 1.4418420 sec
RG 45.2
DW 22.000 usec
DE 6.00 usec
TE 300.0 K
D1 2.00000000 sec
d11 0.03000000 sec
DELTA 1.89999998 sec
TD0 1
======== CHANNEL f1 ========
NUC1 13C
P1 9.70 usec
PL1 -0.50 dB
SFO1 100.6288660 MHz
CPDPRG2 waltz16
NUC2 1H
PCPD2 90.00 usec
PL2 -2.40 dB
PL12 15.10 dB
PL13 18.10 dB
SFO2 400.1516010 MHz
F2 - Processing parameters
SI 32768
SF 100.6178011 MHz
WDW EM
SSB 0
LB 3.00 Hz
GB 0
PC 1.00
C13
190 180 170 160 150 140 130 120 110 100 90 80 70 60 50 40 30 20
n-Pr
n-Pr

1j

Current Data Parameters
NAME 20180716
EXPNO 1
PROCNO 1

F2 - Acquisition Parameters
Date_ 20180715
Time 22.53
INSTRUM spect
PROBHD 5 mm DUL 13C-1
PULPROG zg30
TD 32768
SOLVENT CDCl3
NS 13
DS 0
SWH 6410.256 Hz
FIDRES 0.195625 Hz
AQ 2.5559540 sec
RG 4
DW 78.000 usec
DE 6.00 usec
TE 300.0 K
D1 2.00000000 sec
TD0 1

======== CHANNEL f1 ========
NUC1 1H
P1 10.00 usec
PL1 -2.40 dB
SFO1 400.1528010 MHz

F2 - Processing parameters
SI 16384
SF 400.1500168 MHz
WDW EM
SSB 0
LB 0.00 Hz
GB 0
PC 1.00

RKS-5-229-2-P

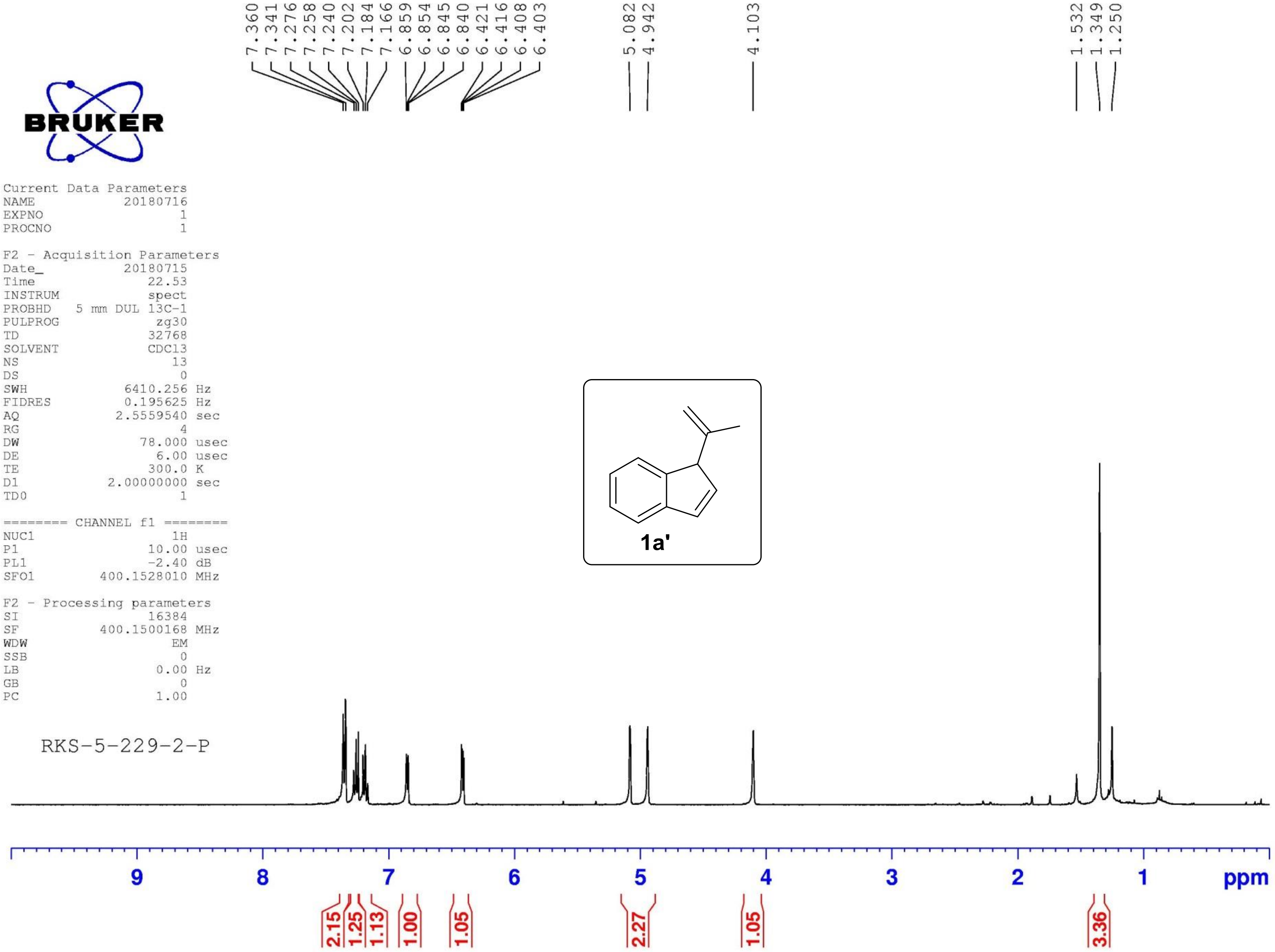

Current Data Parameters
NAME 20180716
EXPNO 2
PROCNO 1

F2 - Acquisition Parameters
Date_ 20180715
Time 22.55
INSTRUM spect
PROBHD 5 mm DUL 13C-1
PULPROG [illegible]
TD 65536
SOLVENT CDCl3
NS 6000
DS 0
SWH 22727.273 Hz
FIDRES 0.346791 Hz
AQ 1.4418420 sec
RG 57
DW 22.000 usec
DE 6.00 usec
TE 300.0 K
D1 2.00000000 sec
d11 0.03000000 sec
DELTA 1.89999998 sec
TD0 1

======== CHANNEL f1 ========
NUC1 13C
P1 9.70 usec
PL1 -0.50 dB
SFO1 100.6288660 MHz

======== CHANNEL f2 ========
CPDPRG2 waltz16
NUC2 1H
PCPD2 90.00 usec
PL2 -2.40 dB
PL12 15.10 dB
PL13 18.10 dB
SFO2 400.1516010 MHz

F2 - Processing parameters
SI 32768
SF 100.6178011 MHz
WDW EM
SSB 0
LB 3.00 Hz
GB 0
PC 1.00

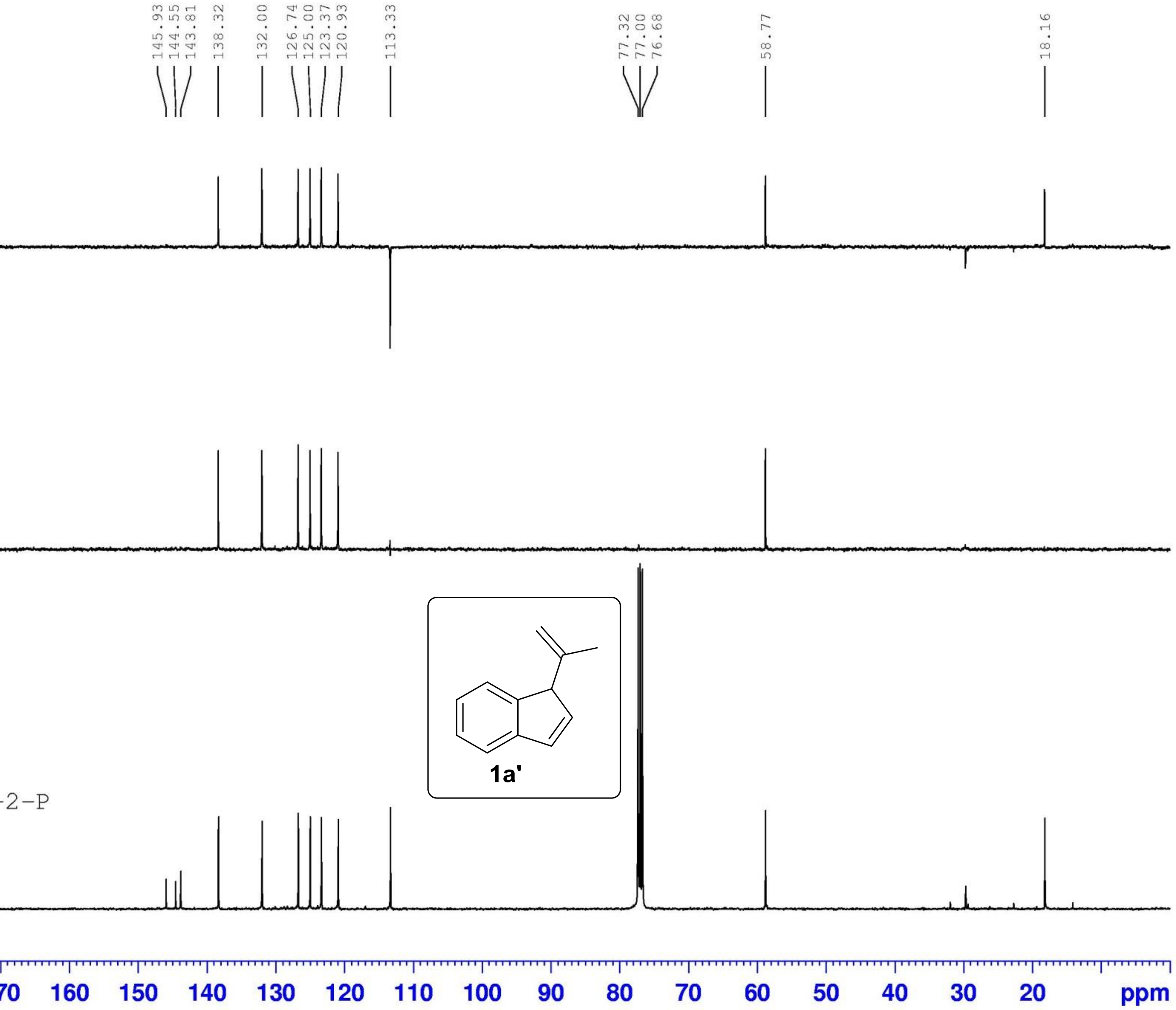

Current Data Parameters
NAME 20170913
EXPNO 2
PROCNO 1

F2 - Acquisition Parameters
Date_ 20170913
Time 15.23
INSTRUM spect
PROBHD 5 mm DUL 13C-1
PULPROG zg30
TD 32768
SOLVENT CDCl3
NS 11
DS 0
SWH 6410.256 Hz
FIDRES 0.195625 Hz
AQ 2.5559540 sec
RG 4
DW 78.000 usec
DE 6.00 usec
TE 300.0 K
D1 2.00000000 sec
TD0 1

======== CHANNEL f1 ========
NUC1 1H
P1 10.00 usec
PL1 -2.40 dB
SFO1 400.1528010 MHz

F2 - Processing parameters
SI 16384
SF 400.1500169 MHz
WDW EM
SSB 0
LB 0.00 Hz
GB 0
PC 1.00

:S-5-235--substra

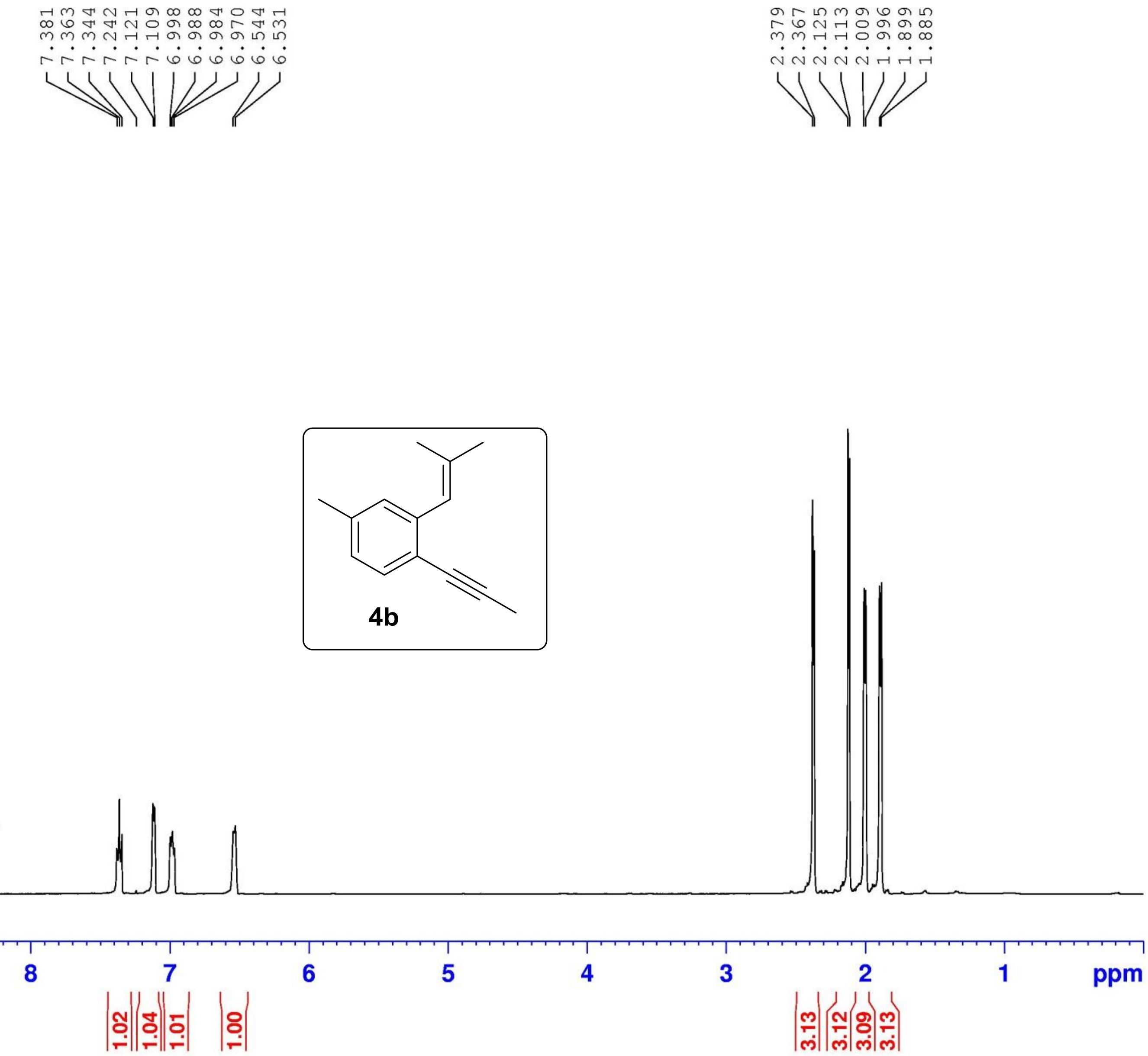

```
Current Data Parameters
NAME              20170913
EXPNO                    4
PROCNO                   1

F2 - Acquisition Parameters
Date_             20170913
Time                 15.29
INSTRUM              spect
PROBHD   5 mm DUL 13C-1
PULPROG            zgpg30
TD                   65536
SOLVENT              CDCl3
NS                      55
DS                       0
SWH           22727.273 Hz
FIDRES         0.346791 Hz
AQ            1.4418420 sec
RG                       4
DW               22.000 usec
DE                 6.00 usec
TE                300.0 K
D1           2.00000000 sec
d11          0.03000000 sec
DELTA        1.89999998 sec
TD0                      1

======== CHANNEL f1 ========
NUC1                   13C
P1                 9.70 usec
PL1               -0.50 dB
SFO1         100.6288660 MHz

======== CHANNEL f2 ========
CPDPRG2            waltz16
NUC2                    1H
PCPD2             90.00 usec
PL2               -2.40 dB
PL12              15.10 dB
PL13              18.10 dB
SFO2         400.1516010 MHz

F2 - Processing parameters
SI                   32768
SF           100.6178201 MHz
WDW                     EM
SSB                      0
LB                 3.00 Hz
GB                       0
PC                    1.00
```

RKS-5-235--substrate

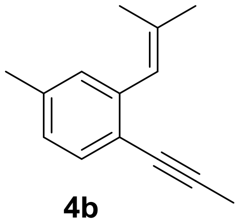

4b

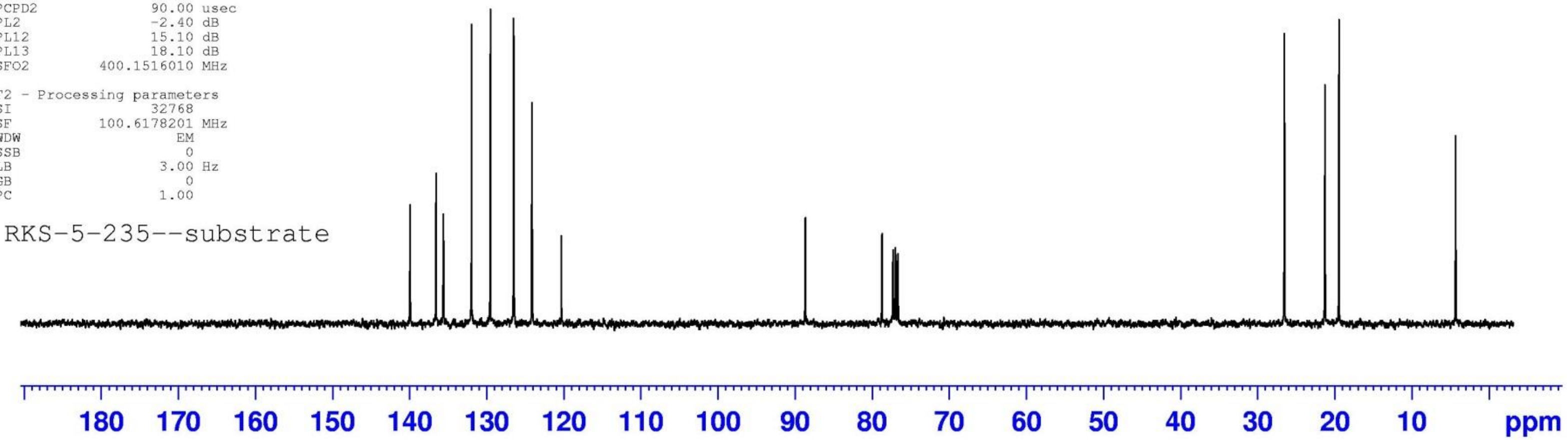

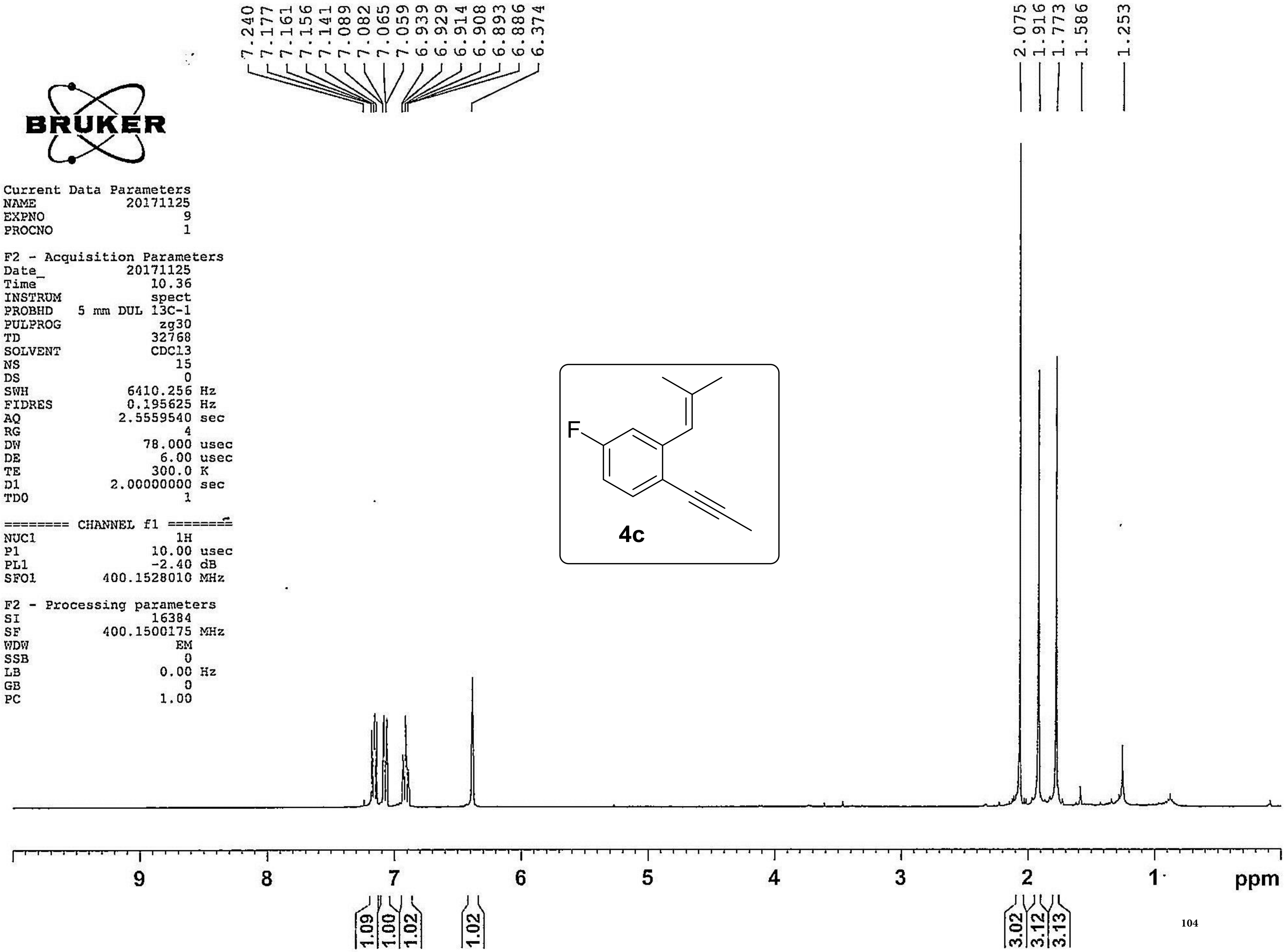
7.240
7.177
7.161
7.156
7.141
7.089
7.082
7.065
7.059
6.939
6.929
6.914
6.908
6.893
6.886
6.374
2.075
1.916
1.773
1.586
1.253
BRUKER
Current Data Parameters
NAME 20171125
EXPNO 9
PROCNO 1
F2 - Acquisition Parameters
Date_ 20171125
Time 10.36
INSTRUM spect
PROBHD 5 mm DUL 13C-1
PULPROG zg30
TD 32768
SOLVENT CDCl3
NS 15
DS 0
SWH 6410.256 Hz
FIDRES 0.195625 Hz
AQ 2.5559540 sec
RG 4
DW 78.000 usec
DE 6.00 usec
TE 300.0 K
D1 2.00000000 sec
TD0 1
======== CHANNEL f1 ========
NUC1 1H
P1 10.00 usec
PL1 -2.40 dB
SFO1 400.1528010 MHz
F2 - Processing parameters
SI 16384
SF 400.1500175 MHz
WDW EM
SSB 0
LB 0.00 Hz
GB 0
PC 1.00
F
4c
9
8
7
6
5
4
3
2
1
ppm
1.09
1.00
1.02
1.02
3.02
3.12
3.13

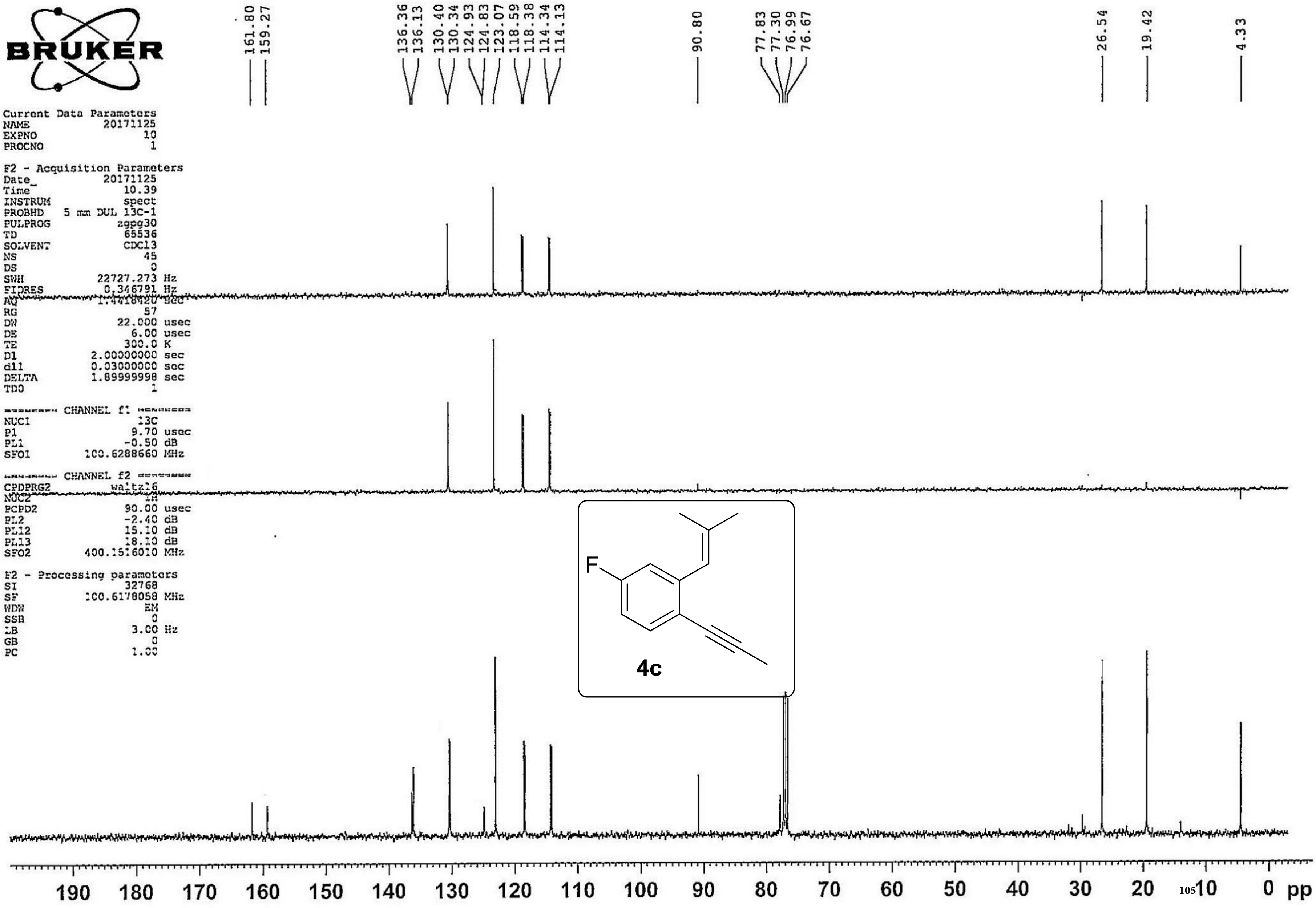
BRUKER
161.80
159.27
136.36
136.13
130.40
130.34
124.93
124.83
123.07
118.59
118.38
114.34
114.13
90.80
77.83
77.30
76.99
76.67
26.54
19.42
4.33
Current Data Parameters
NAME 20171125
EXPNO 10
PROCNO 1
F2 - Acquisition Parameters
Date_ 20171125
Time 10.39
INSTRUM spect
PROBHD 5 mm DUL 13C-1
PULPROG zgpg30
TD 65536
SOLVENT CDCl3
NS 45
DS 0
SWH 22727.273 Hz
FIDRES 0.346791 Hz
RG 57
DW 22.000 usec
DE 6.00 usec
TE 300.0 K
D1 2.00000000 sec
d11 0.03000000 sec
DELTA 1.89999998 sec
TD0 1
CHANNEL f1
NUC1 13C
P1 9.70 usec
PL1 -0.50 dB
SFO1 100.6288660 MHz
CHANNEL f2
CPDPRG2 waltz16
PCPD2 90.00 usec
PL2 -2.40 dB
PL12 15.10 dB
PL13 18.10 dB
SFO2 400.1516010 MHz
F2 - Processing parameters
SI 32768
SF 100.6178058 MHz
WDW EM
SSB 0
LB 3.00 Hz
GB 0
PC 1.00
F
4c
190 180 170 160 150 140 130 120 110 100 90 80 70 60 50 40 30 20 10 0 ppm

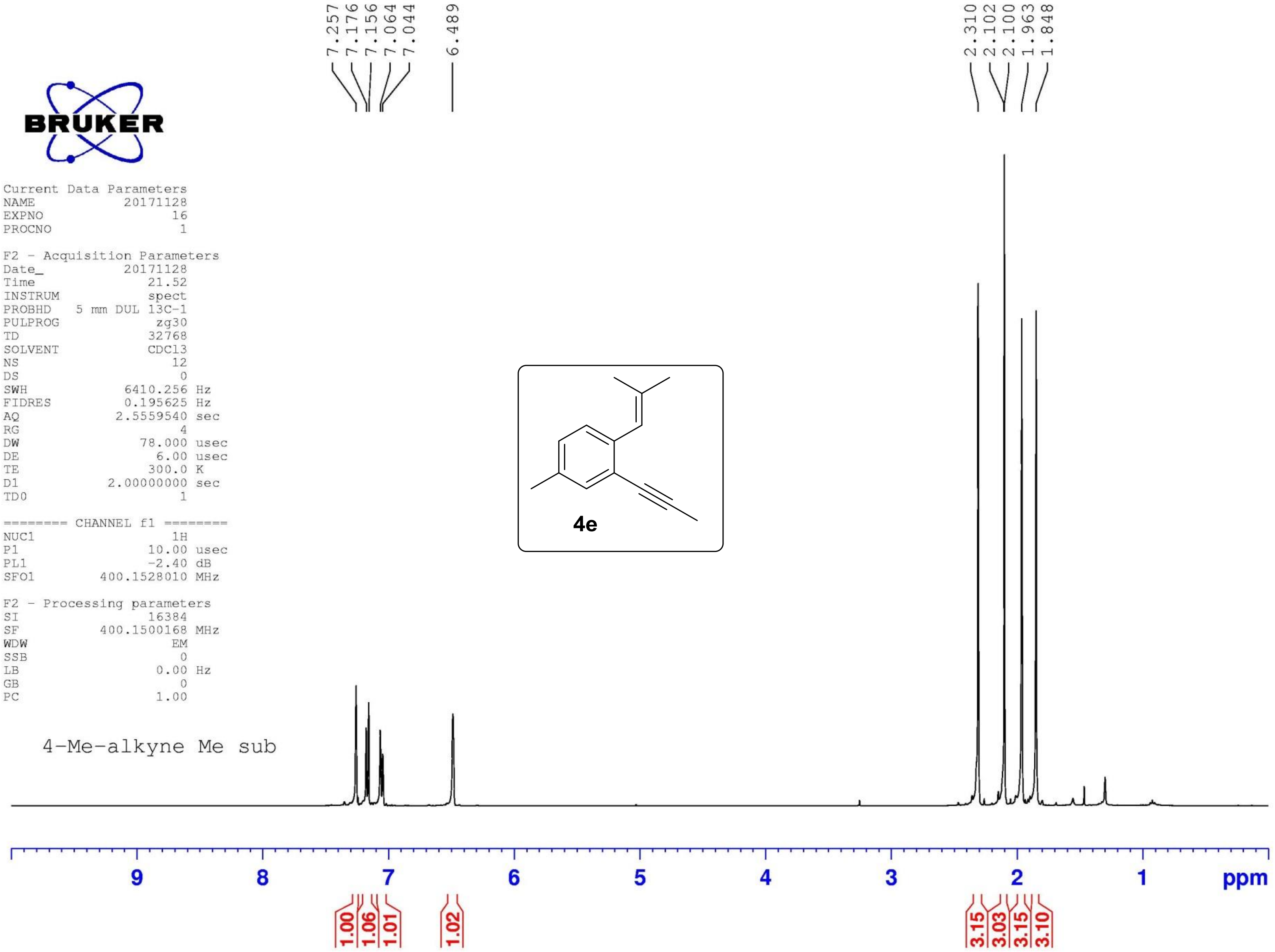
7.257
7.176
7.156
7.064
7.044
6.489
2.310
2.102
2.100
1.963
1.848
BRUKER
Current Data Parameters
NAME 20171128
EXPNO 16
PROCNO 1
F2 - Acquisition Parameters
Date_ 20171128
Time 21.52
INSTRUM spect
PROBHD 5 mm DUL 13C-1
PULPROG zg30
TD 32768
SOLVENT CDCl3
NS 12
DS 0
SWH 6410.256 Hz
FIDRES 0.195625 Hz
AQ 2.5559540 sec
RG 4
DW 78.000 usec
DE 6.00 usec
TE 300.0 K
D1 2.00000000 sec
TD0 1
======== CHANNEL f1 ========
NUC1 1H
P1 10.00 usec
PL1 -2.40 dB
SFO1 400.1528010 MHz
F2 - Processing parameters
SI 16384
SF 400.1500168 MHz
WDW EM
SSB 0
LB 0.00 Hz
GB 0
PC 1.00
4-Me-alkyne Me sub
4e
9
8
7
6
5
4
3
2
1
ppm
1.00
1.06
1.01
1.02
3.15
3.03
3.15
3.10

Current Data Parameters
NAME 20171128
EXPNO 17
PROCNO 1

F2 - Acquisition Parameters
Date_ 20171128
Time 21.54
INSTRUM spect
PROBHD 5 mm DUL 13C-1
PULPROG zgpg30
TD 65536
SOLVENT CDCl3
NS 59
DS 0
SWH [illegible] Hz
FIDRES 0.346791 Hz
AQ 1.4418420 sec
RG 64
DW 22.000 usec
DE 6.00 usec
TE 300.0 K
D1 2.00000000 sec
d11 0.03000000 sec
DELTA 1.89999998 sec
TD0 1

======== CHANNEL f1 ========
NUC1 13C
P1 9.70 usec
PL1 -0.50 dB
SFO1 100.6288660 MHz

======== CHANNEL f2 ========
CPDPRG2 waltz16
NUC2 1H
PCPD2 90.00 usec
PL2 -2.40 dB
PL12 15.10 dB
PL13 18.10 dB
SFO2 400.1516010 MHz

F2 - Processing parameters
SI 32768
SF 100.6178110 MHz
WDW EM
SSB 0
LB 3.00 Hz
GB 0
PC 1.00

4-Me-alkyne Me sub

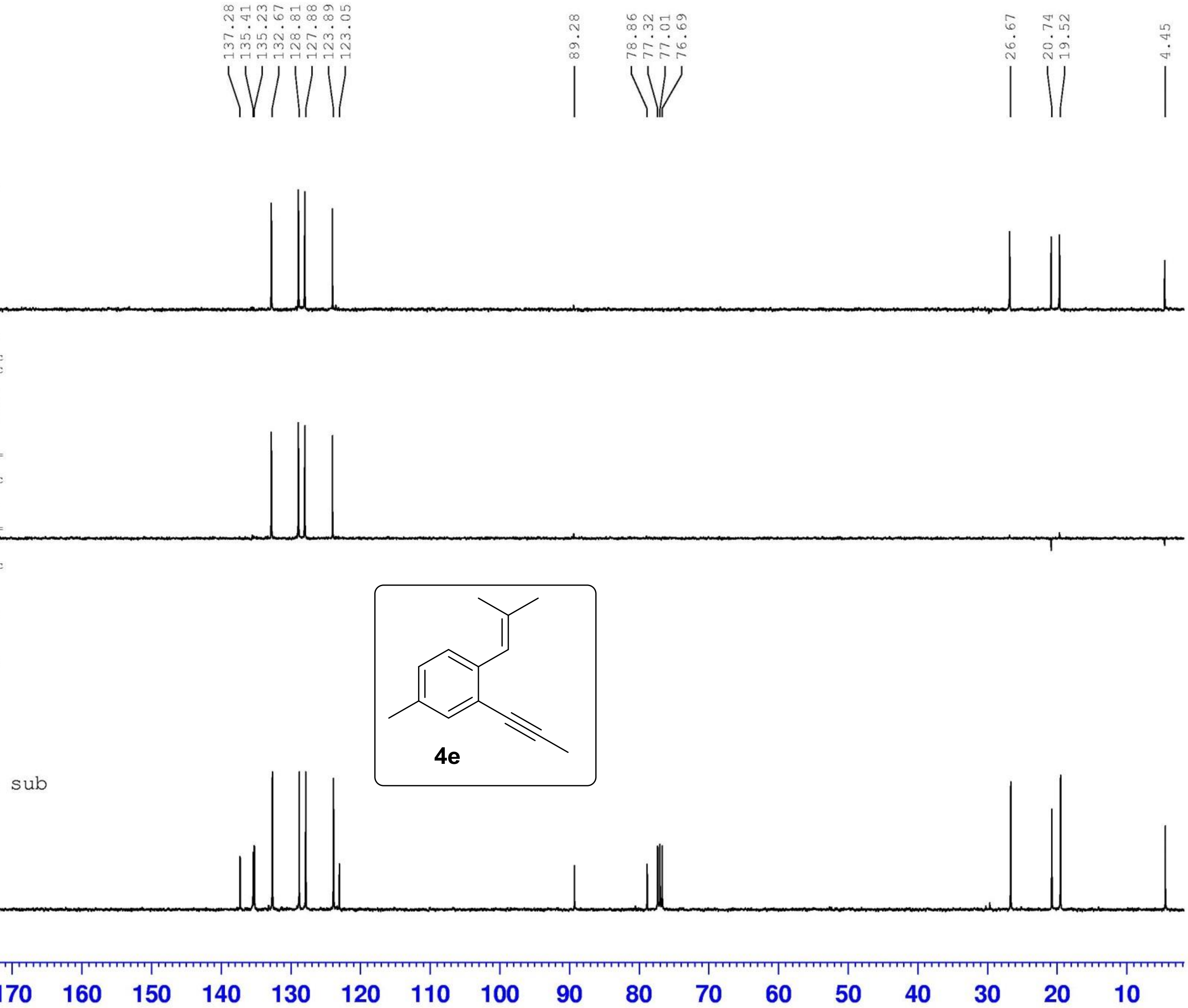

4e

Current Data Parameters
NAME 20171128
EXPNO 9
PROCNO 1

F2 - Acquisition Parameters
Date_ 20171128
Time 15.36
INSTRUM spect
PROBHD 5 mm DUL 13C-1
PULPROG zg30
TD 32768
SOLVENT CDCl3
NS 15
DS 0
SWH 6410.256 Hz
FIDRES 0.195625 Hz
AQ 2.5559540 sec
RG 4
DW 78.000 usec
DE 6.00 usec
TE 300.0 K
D1 2.00000000 sec
TD0 1

======== CHANNEL f1 ========
NUC1 1H
P1 10.00 usec
PL1 -2.40 dB
SFO1 400.1528010 MHz

F2 - Processing parameters
SI 16384
SF 400.1500175 MHz
WDW EM
SSB 0
LB 0.00 Hz
GB 0
PC 1.00

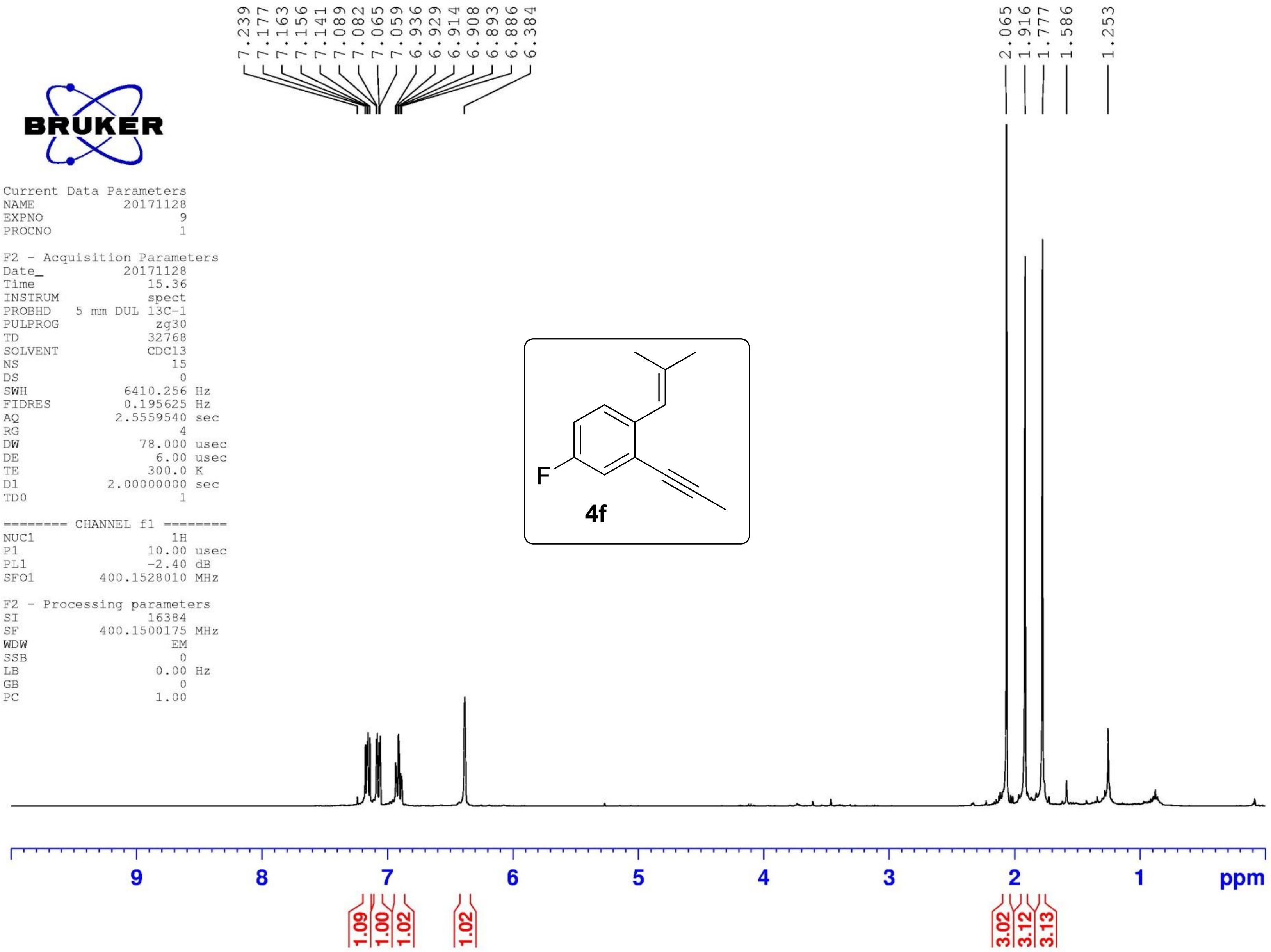

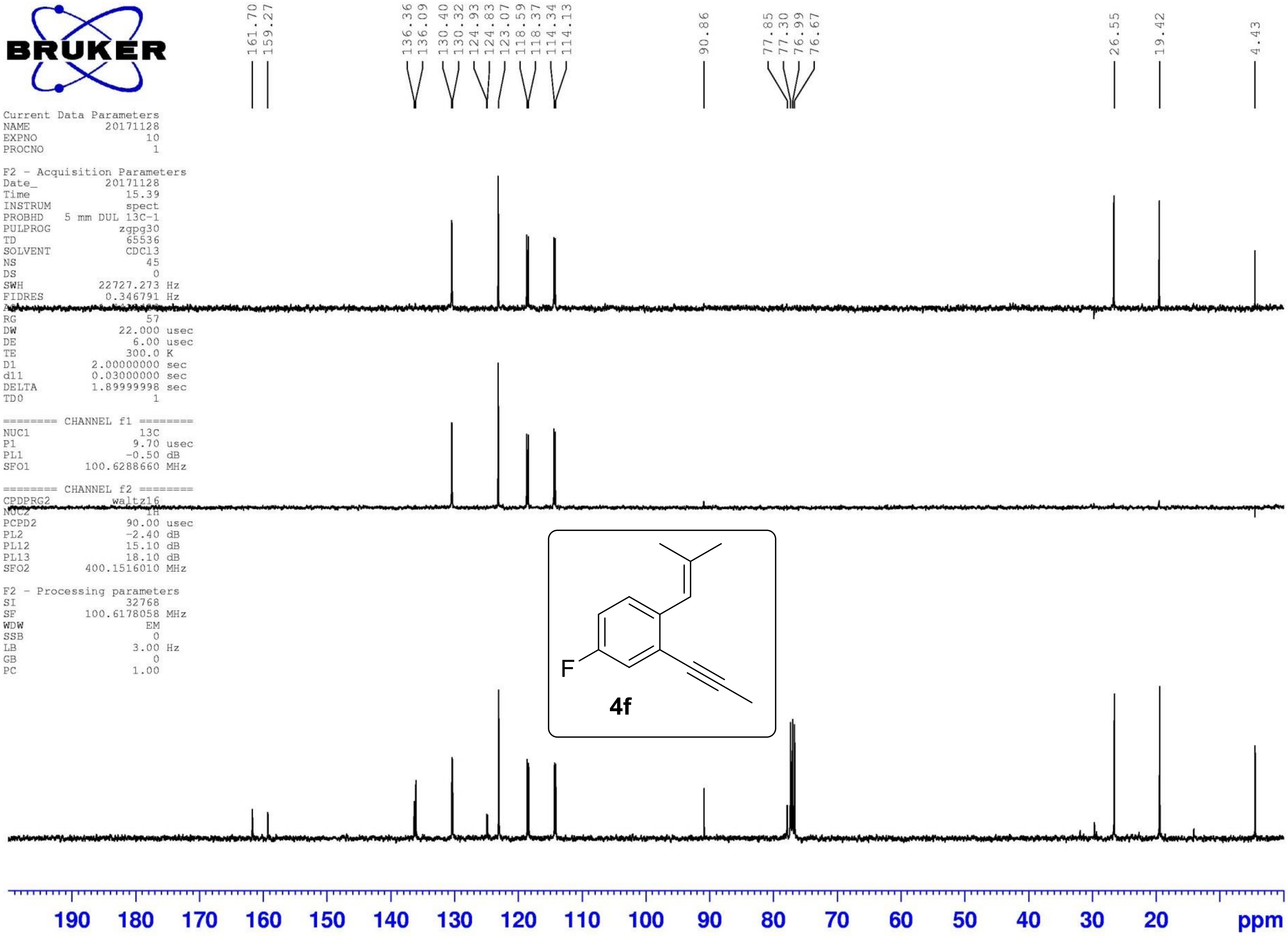

BRUKER
161.70
159.27
136.36
136.09
130.40
130.32
124.93
124.83
123.07
118.59
118.37
114.34
114.13
90.86
77.85
77.30
76.99
76.67
26.55
19.42
4.43
Current Data Parameters
NAME 20171128
EXPNO 10
PROCNO 1
F2 - Acquisition Parameters
Date_ 20171128
Time 15.39
INSTRUM spect
PROBHD 5 mm DUL 13C-1
PULPROG zgpg30
TD 65536
SOLVENT CDCl3
NS 45
DS 0
SWH 22727.273 Hz
FIDRES 0.346791 Hz
RG 57
DW 22.000 usec
DE 6.00 usec
TE 300.0 K
D1 2.00000000 sec
d11 0.03000000 sec
DELTA 1.89999998 sec
TD0 1
======== CHANNEL f1 ========
NUC1 13C
P1 9.70 usec
PL1 -0.50 dB
SFO1 100.6288660 MHz
======== CHANNEL f2 ========
CPDPRG2 waltz16
NUC2 1H
PCPD2 90.00 usec
PL2 -2.40 dB
PL12 15.10 dB
PL13 18.10 dB
SFO2 400.1516010 MHz
F2 - Processing parameters
SI 32768
SF 100.6178058 MHz
WDW EM
SSB 0
LB 3.00 Hz
GB 0
PC 1.00
F
4f
190 180 170 160 150 140 130 120 110 100 90 80 70 60 50 40 30 20 ppm

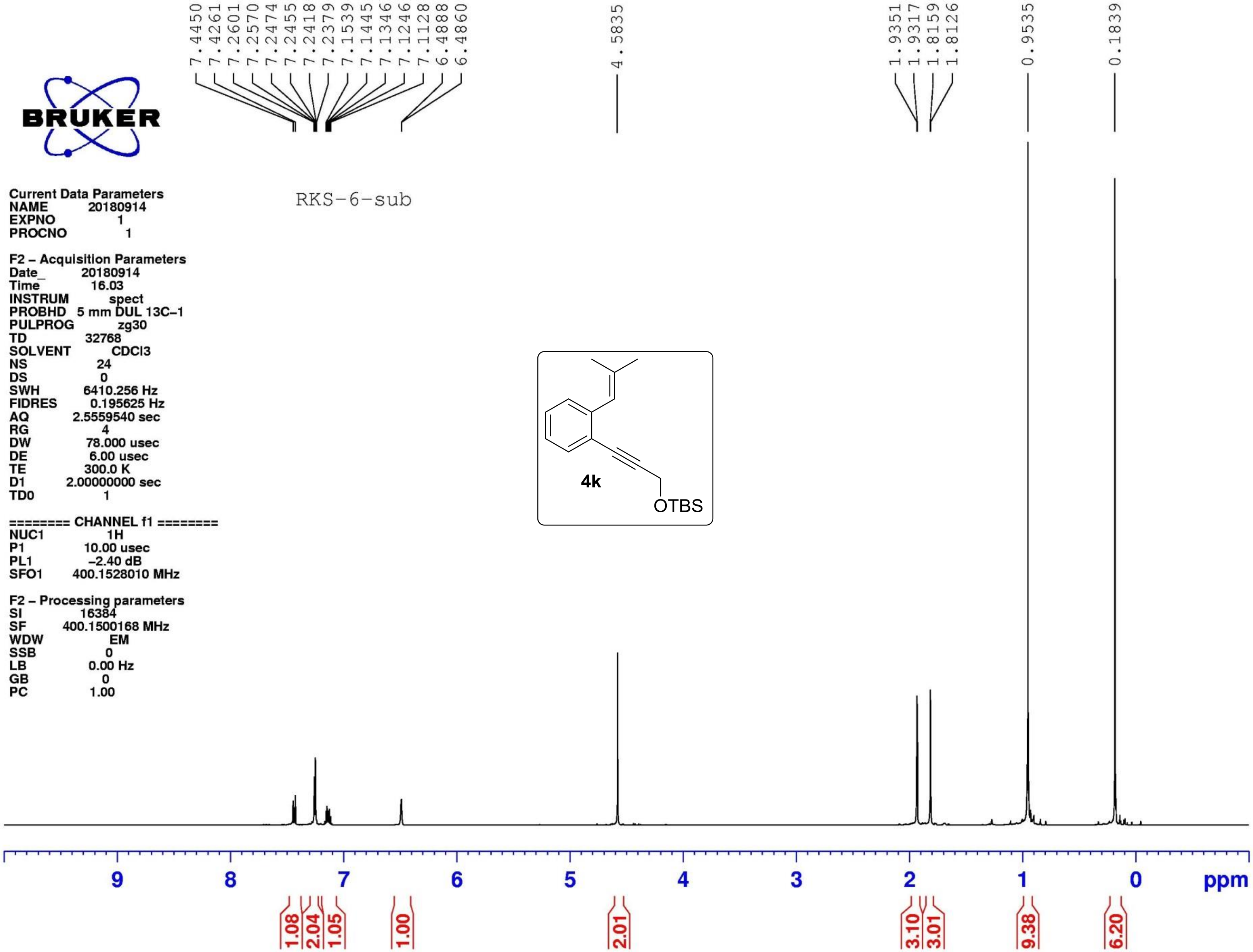
BRUKER
RKS-6-sub
Current Data Parameters
NAME 20180914
EXPNO 1
PROCNO 1
F2 - Acquisition Parameters
Date_ 20180914
Time 16.03
INSTRUM spect
PROBHD 5 mm DUL 13C-1
PULPROG zg30
TD 32768
SOLVENT CDCl3
NS 24
DS 0
SWH 6410.256 Hz
FIDRES 0.195625 Hz
AQ 2.5559540 sec
RG 4
DW 78.000 usec
DE 6.00 usec
TE 300.0 K
D1 2.00000000 sec
TD0 1
======== CHANNEL f1 ========
NUC1 1H
P1 10.00 usec
PL1 -2.40 dB
SFO1 400.1528010 MHz
F2 - Processing parameters
SI 16384
SF 400.1500168 MHz
WDW EM
SSB 0
LB 0.00 Hz
GB 0
PC 1.00
7.4450
7.4261
7.2601
7.2570
7.2474
7.2455
7.2418
7.2379
7.1539
7.1445
7.1346
7.1246
7.1128
6.4888
6.4860
4.5835
1.9351
1.9317
1.8159
1.8126
0.9535
0.1839
4k
OTBS
9
8
7
6
5
4
3
2
1
0
ppm
1.08
2.04
1.05
1.00
2.01
3.10
3.01
9.38
6.20

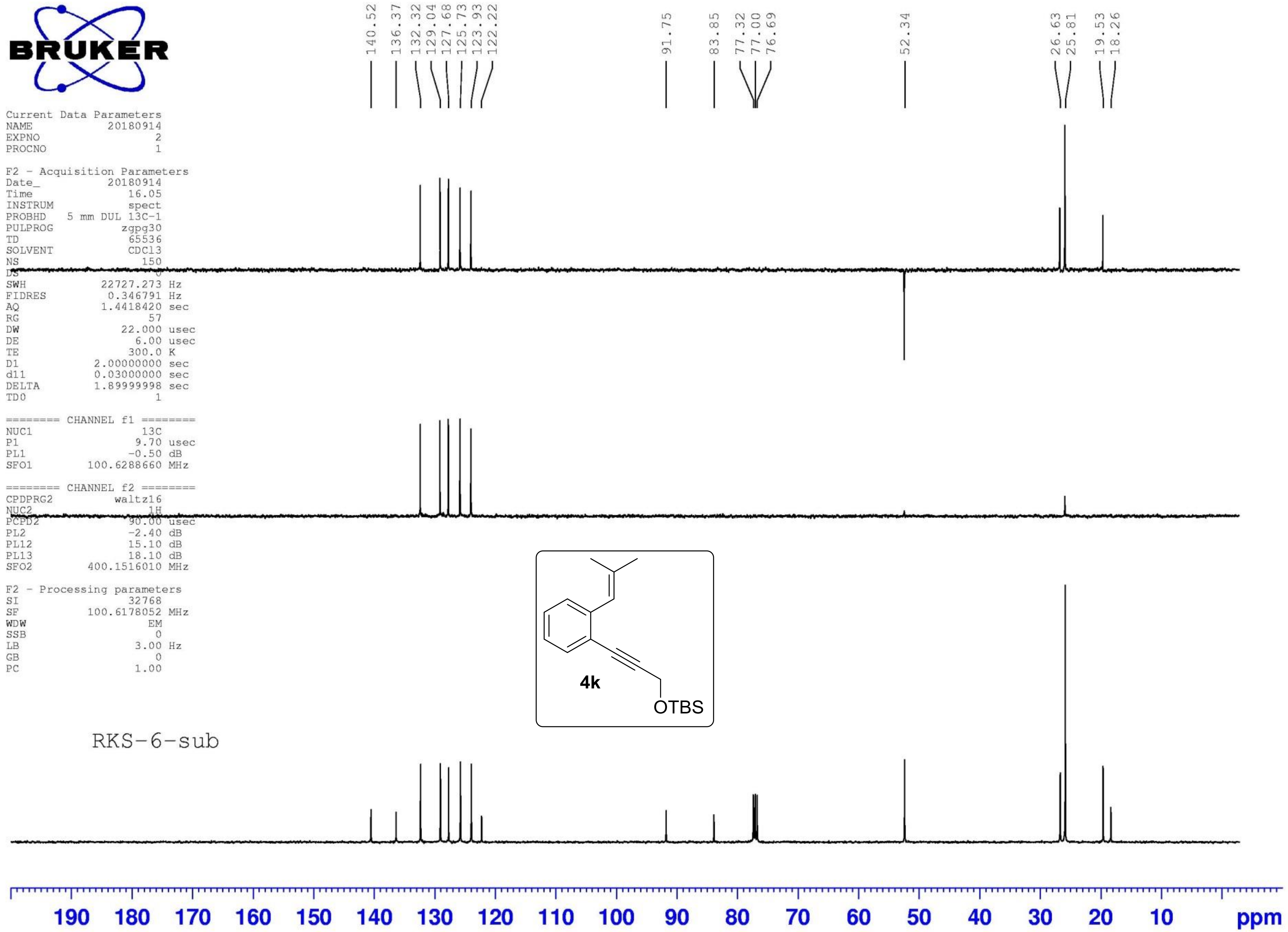
BRUKER
140.52
136.37
132.32
129.04
127.68
125.73
123.93
122.22
91.75
83.85
77.32
77.00
76.69
52.34
26.63
25.81
19.53
18.26
Current Data Parameters
NAME 20180914
EXPNO 2
PROCNO 1
F2 - Acquisition Parameters
Date_ 20180914
Time 16.05
INSTRUM spect
PROBHD 5 mm DUL 13C-1
PULPROG zgpg30
TD 65536
SOLVENT CDCl3
NS 150
DS 4
SWH 22727.273 Hz
FIDRES 0.346791 Hz
AQ 1.4418420 sec
RG 57
DW 22.000 usec
DE 6.00 usec
TE 300.0 K
D1 2.00000000 sec
d11 0.03000000 sec
DELTA 1.89999998 sec
TD0 1
======== CHANNEL f1 ========
NUC1 13C
P1 9.70 usec
PL1 -0.50 dB
SFO1 100.6288660 MHz
======== CHANNEL f2 ========
CPDPRG2 waltz16
NUC2 1H
PCPD2 90.00 usec
PL2 -2.40 dB
PL12 15.10 dB
PL13 18.10 dB
SFO2 400.1516010 MHz
F2 - Processing parameters
SI 32768
SF 100.6178052 MHz
WDW EM
SSB 0
LB 3.00 Hz
GB 0
PC 1.00
4k
OTBS
RKS-6-sub
190 180 170 160 150 140 130 120 110 100 90 80 70 60 50 40 30 20 10 ppm

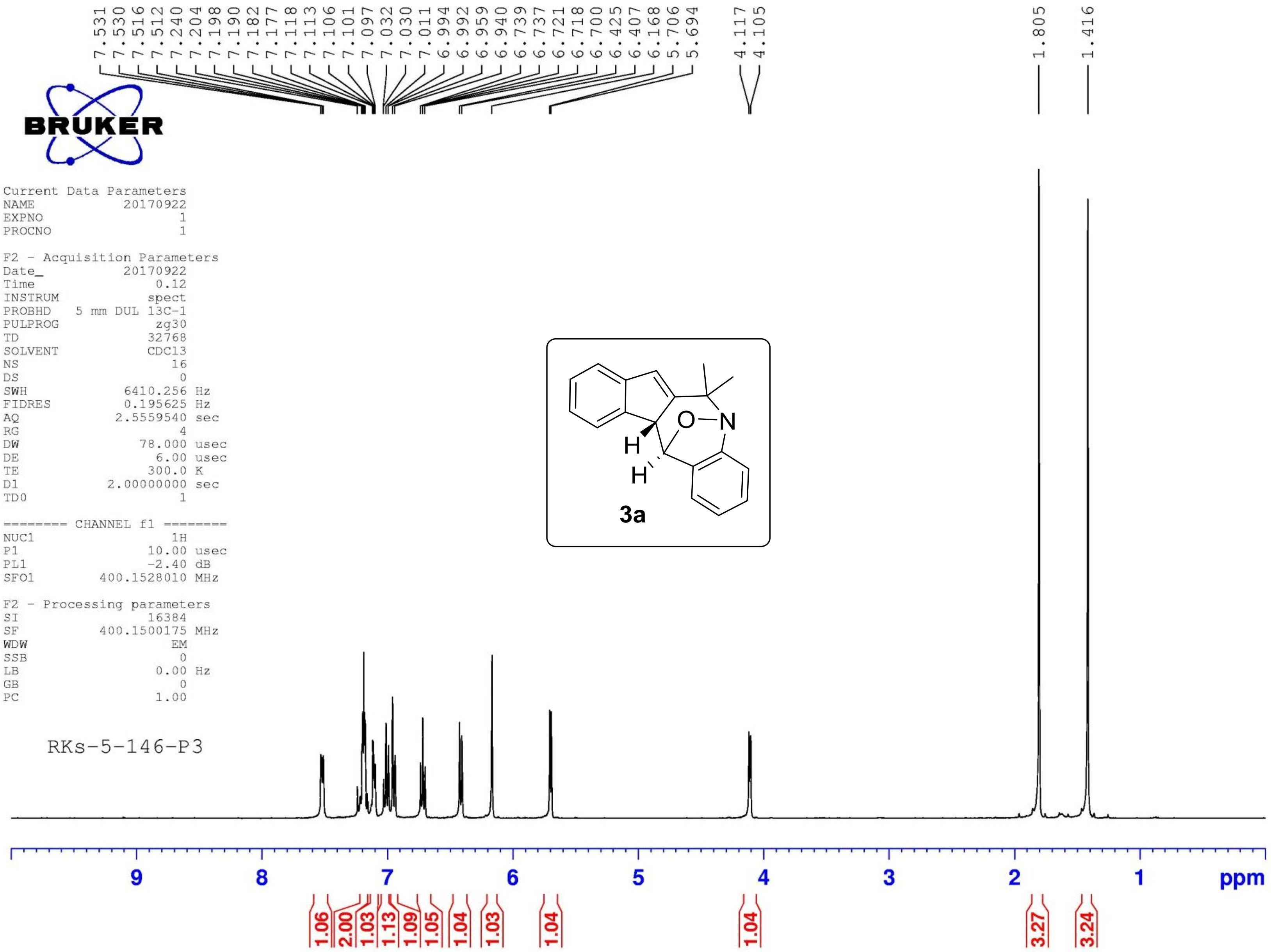
7.531
7.530
7.516
7.512
7.240
7.204
7.198
7.190
7.182
7.177
7.118
7.113
7.106
7.101
7.097
7.032
7.030
7.011
6.994
6.992
6.959
6.940
6.739
6.737
6.721
6.718
6.700
6.425
6.407
6.168
5.706
5.694
4.117
4.105
1.805
1.416
BRUKER
Current Data Parameters
NAME 20170922
EXPNO 1
PROCNO 1
F2 - Acquisition Parameters
Date_ 20170922
Time 0.12
INSTRUM spect
PROBHD 5 mm DUL 13C-1
PULPROG zg30
TD 32768
SOLVENT CDCl3
NS 16
DS 0
SWH 6410.256 Hz
FIDRES 0.195625 Hz
AQ 2.5559540 sec
RG 4
DW 78.000 usec
DE 6.00 usec
TE 300.0 K
D1 2.00000000 sec
TD0 1
======== CHANNEL f1 ========
NUC1 1H
P1 10.00 usec
PL1 -2.40 dB
SFO1 400.1528010 MHz
F2 - Processing parameters
SI 16384
SF 400.1500175 MHz
WDW EM
SSB 0
LB 0.00 Hz
GB 0
PC 1.00
RKs-5-146-P3
O
N
H
H
3a
9
8
7
6
5
4
3
2
1
ppm
1.06
2.00
1.03
1.13
1.09
1.05
1.04
1.03
1.04
1.04
3.27
3.24

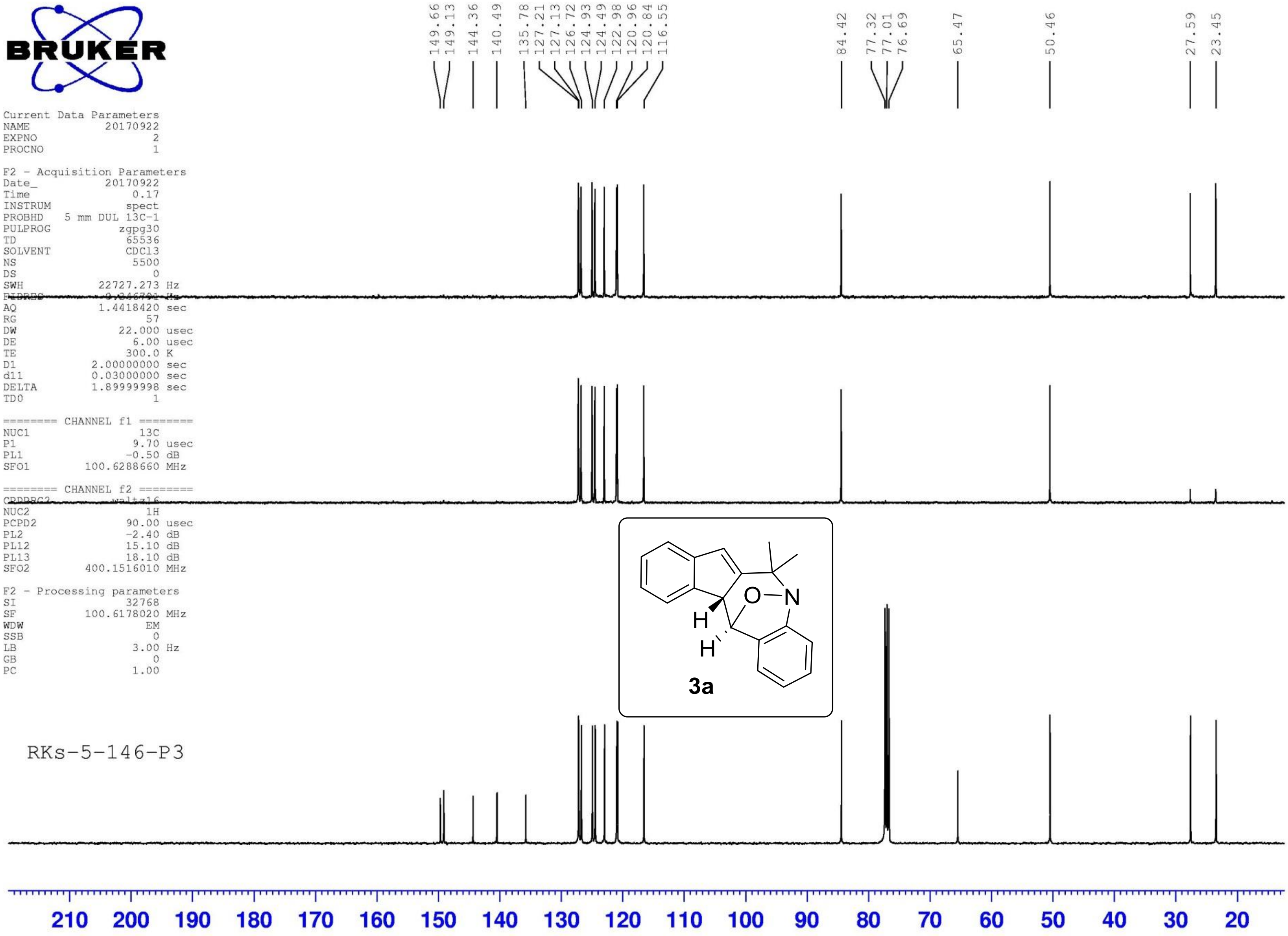
BRUKER
149.66
149.13
144.36
140.49
135.78
127.21
127.13
126.72
124.93
124.49
122.98
120.96
120.84
116.55
84.42
77.32
77.01
76.69
65.47
50.46
27.59
23.45
Current Data Parameters
NAME 20170922
EXPNO 2
PROCNO 1
F2 - Acquisition Parameters
Date_ 20170922
Time 0.17
INSTRUM spect
PROBHD 5 mm DUL 13C-1
PULPROG zgpg30
TD 65536
SOLVENT CDCl3
NS 5500
DS 0
SWH 22727.273 Hz
FIDRES 0.346791 Hz
AQ 1.4418420 sec
RG 57
DW 22.000 usec
DE 6.00 usec
TE 300.0 K
D1 2.00000000 sec
d11 0.03000000 sec
DELTA 1.89999998 sec
TD0 1
======== CHANNEL f1 ========
NUC1 13C
P1 9.70 usec
PL1 -0.50 dB
SFO1 100.6288660 MHz
======== CHANNEL f2 ========
CPDPRG2 waltz16
NUC2 1H
PCPD2 90.00 usec
PL2 -2.40 dB
PL12 15.10 dB
PL13 18.10 dB
SFO2 400.1516010 MHz
F2 - Processing parameters
SI 32768
SF 100.6178020 MHz
WDW EM
SSB 0
LB 3.00 Hz
GB 0
PC 1.00
RKs-5-146-P3
O
N
H
H
3a
210 200 190 180 170 160 150 140 130 120 110 100 90 80 70 60 50 40 30 20

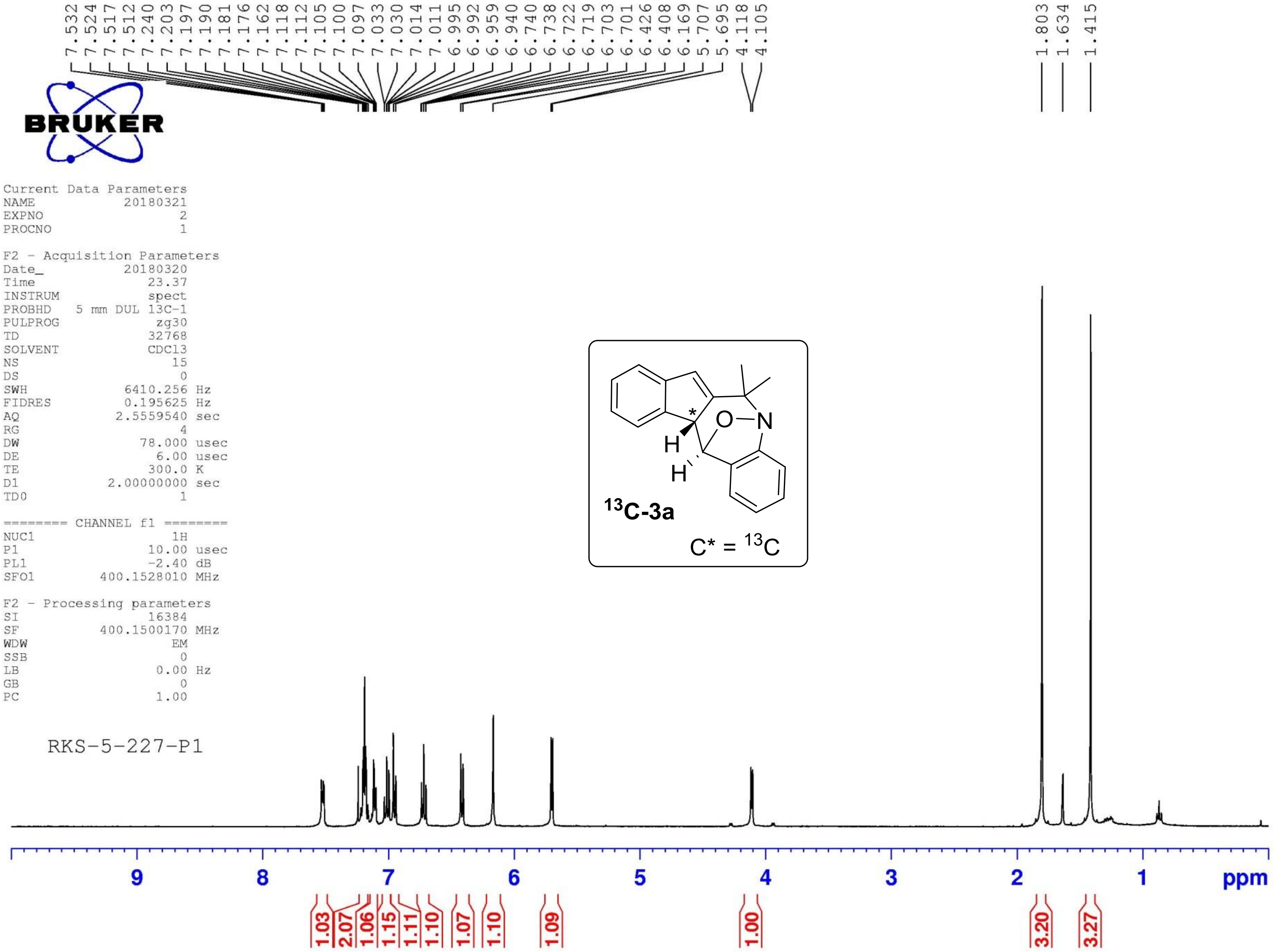
7.532 7.524 7.517 7.512 7.240 7.203 7.197 7.190 7.181 7.176 7.162 7.118 7.112 7.105 7.100 7.097 7.033 7.030 7.014 7.011 6.995 6.992 6.959 6.940 6.740 6.738 6.722 6.719 6.703 6.701 6.426 6.408 6.169 5.707 5.695 4.118 4.105
1.803 1.634 1.415
BRUKER
Current Data Parameters
NAME 20180321
EXPNO 2
PROCNO 1
F2 - Acquisition Parameters
Date_ 20180320
Time 23.37
INSTRUM spect
PROBHD 5 mm DUL 13C-1
PULPROG zg30
TD 32768
SOLVENT CDCl3
NS 15
DS 0
SWH 6410.256 Hz
FIDRES 0.195625 Hz
AQ 2.5559540 sec
RG 4
DW 78.000 usec
DE 6.00 usec
TE 300.0 K
D1 2.00000000 sec
TD0 1
======== CHANNEL f1 ========
NUC1 1H
P1 10.00 usec
PL1 -2.40 dB
SFO1 400.1528010 MHz
F2 - Processing parameters
SI 16384
SF 400.1500170 MHz
WDW EM
SSB 0
LB 0.00 Hz
GB 0
PC 1.00
RKS-5-227-P1
O
N
H
H
*
13C-3a
C* = 13C
9 8 7 6 5 4 3 2 1 ppm
1.03 2.07 1.06 1.15 1.11 1.10 1.07 1.10 1.09 1.00 3.20 3.27

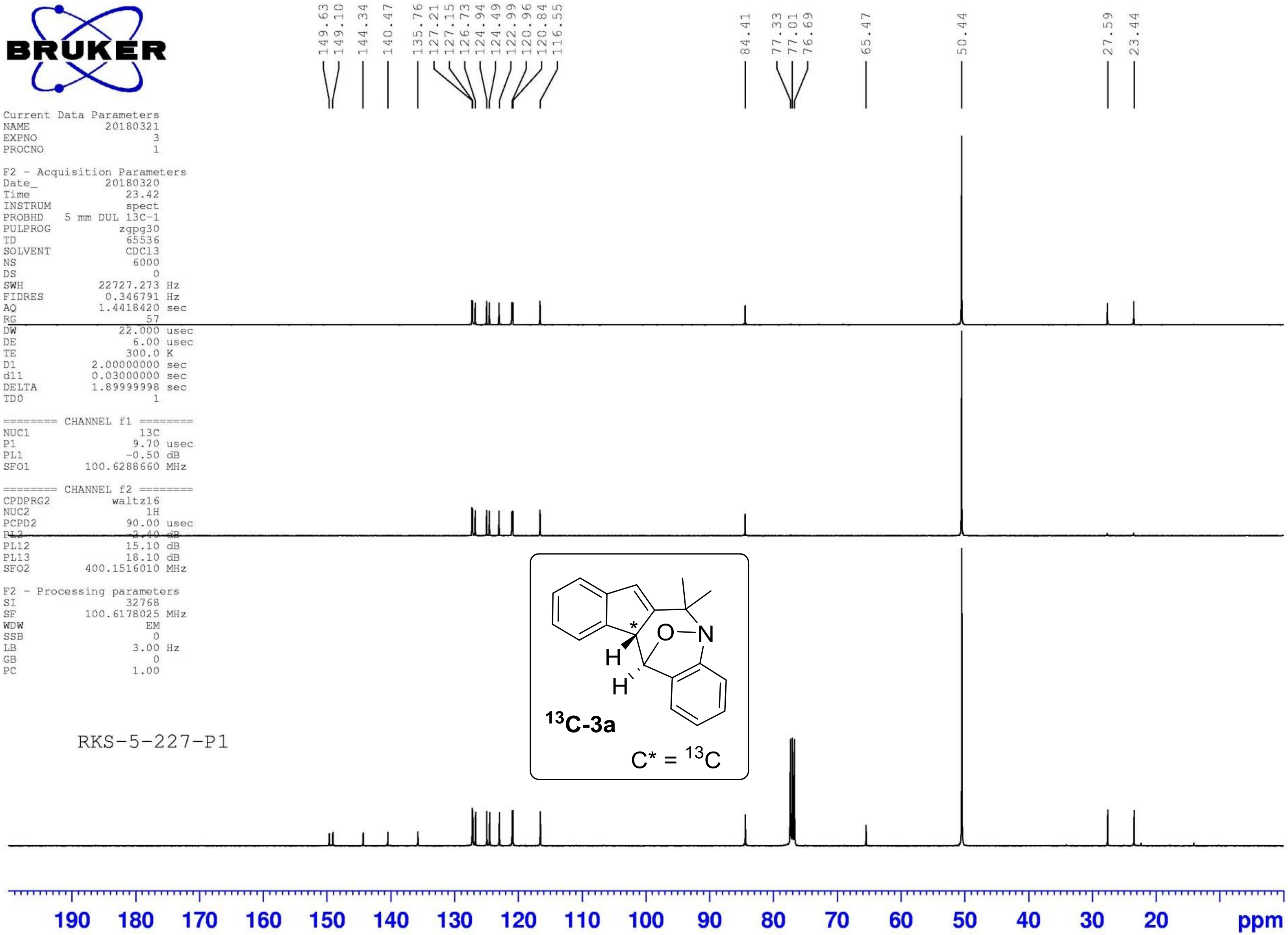
BRUKER
149.63
149.10
144.34
140.47
135.76
127.21
127.15
126.73
124.94
124.49
122.99
120.96
120.84
116.55
84.41
77.33
77.01
76.69
65.47
50.44
27.59
23.44
Current Data Parameters
NAME 20180321
EXPNO 3
PROCNO 1
F2 - Acquisition Parameters
Date_ 20180320
Time 23.42
INSTRUM spect
PROBHD 5 mm DUL 13C-1
PULPROG zgpg30
TD 65536
SOLVENT CDCl3
NS 6000
DS 0
SWH 22727.273 Hz
FIDRES 0.346791 Hz
AQ 1.4418420 sec
RG 57
DW 22.000 usec
DE 6.00 usec
TE 300.0 K
D1 2.00000000 sec
d11 0.03000000 sec
DELTA 1.89999998 sec
TD0 1
======== CHANNEL f1 ========
NUC1 13C
P1 9.70 usec
PL1 -0.50 dB
SFO1 100.6288660 MHz
======== CHANNEL f2 ========
CPDPRG2 waltz16
NUC2 1H
PCPD2 90.00 usec
PL2 -2.40 dB
PL12 15.10 dB
PL13 18.10 dB
SFO2 400.1516010 MHz
F2 - Processing parameters
SI 32768
SF 100.6178025 MHz
WDW EM
SSB 0
LB 3.00 Hz
GB 0
PC 1.00
RKS-5-227-P1
O
N
H
H
*
13C-3a
C* = 13C
190 180 170 160 150 140 130 120 110 100 90 80 70 60 50 40 30 20 ppm

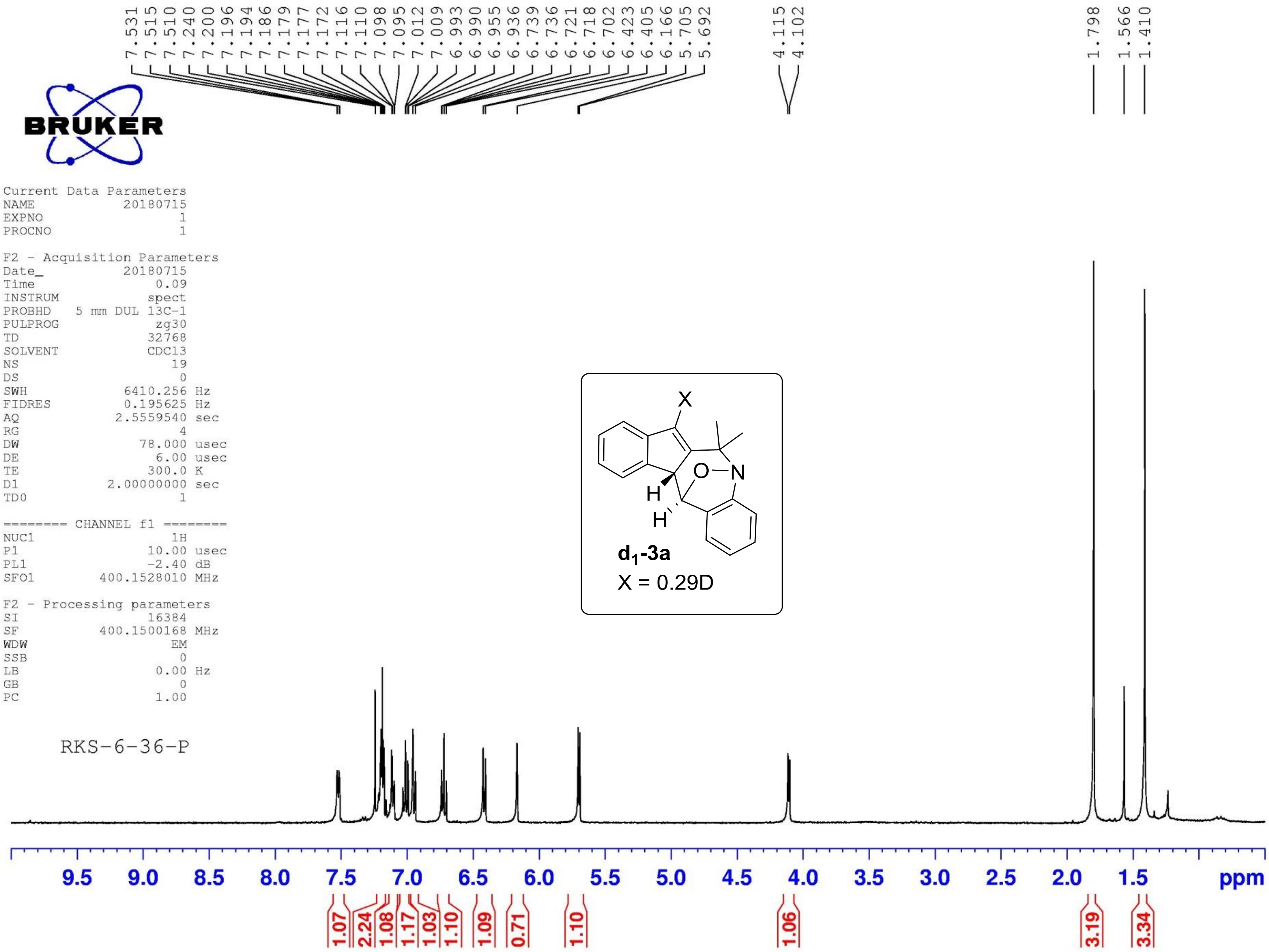

7.531
7.515
7.510
7.240
7.200
7.196
7.194
7.186
7.179
7.177
7.172
7.116
7.110
7.098
7.095
7.012
7.009
6.993
6.990
6.955
6.936
6.739
6.736
6.721
6.718
6.702
6.423
6.405
6.166
5.705
5.692
4.115
4.102
1.798
1.566
1.410
BRUKER
Current Data Parameters
NAME 20180715
EXPNO 1
PROCNO 1
F2 - Acquisition Parameters
Date_ 20180715
Time 0.09
INSTRUM spect
PROBHD 5 mm DUL 13C-1
PULPROG zg30
TD 32768
SOLVENT CDCl3
NS 19
DS 0
SWH 6410.256 Hz
FIDRES 0.195625 Hz
AQ 2.5559540 sec
RG 4
DW 78.000 usec
DE 6.00 usec
TE 300.0 K
D1 2.00000000 sec
TD0 1
======== CHANNEL f1 ========
NUC1 1H
P1 10.00 usec
PL1 -2.40 dB
SFO1 400.1528010 MHz
F2 - Processing parameters
SI 16384
SF 400.1500168 MHz
WDW EM
SSB 0
LB 0.00 Hz
GB 0
PC 1.00
RKS-6-36-P
X
O
N
H
H
d1-3a
X = 0.29D
9.5
9.0
8.5
8.0
7.5
7.0
6.5
6.0
5.5
5.0
4.5
4.0
3.5
3.0
2.5
2.0
1.5
ppm
1.07
2.24
1.08
1.17
1.03
1.10
1.09
0.71
1.10
1.06
3.19
3.34

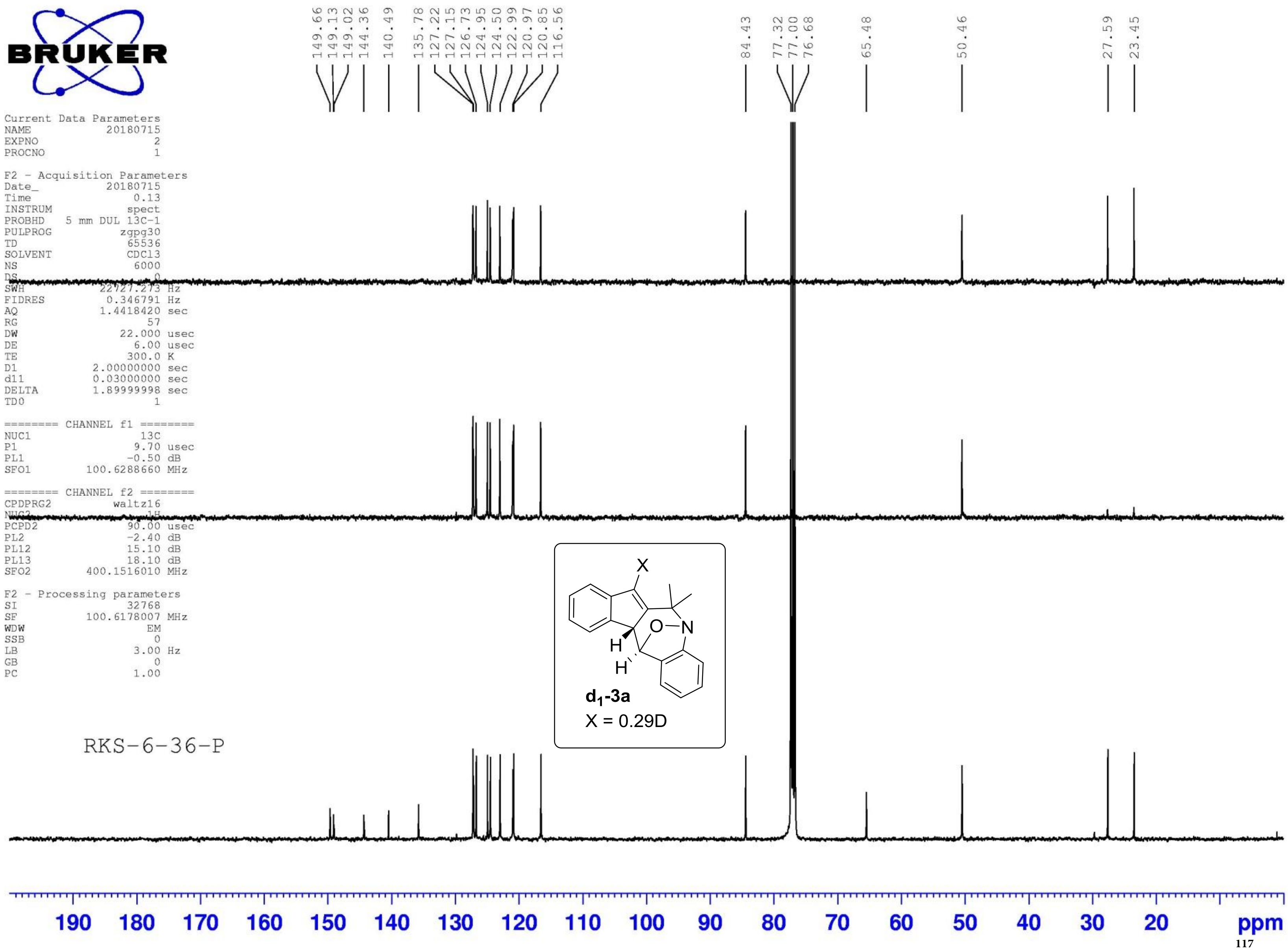

149.66
149.13
149.02
144.36
140.49
135.78
127.22
127.15
126.73
124.95
124.50
122.99
120.97
120.85
116.56
84.43
77.32
77.00
76.68
65.48
50.46
27.59
23.45
BRUKER
Current Data Parameters
NAME 20180715
EXPNO 2
PROCNO 1
F2 - Acquisition Parameters
Date_ 20180715
Time 0.13
INSTRUM spect
PROBHD 5 mm DUL 13C-1
PULPROG zgpg30
TD 65536
SOLVENT CDCl3
NS 6000
DS 0
SWH 22727.273 Hz
FIDRES 0.346791 Hz
AQ 1.4418420 sec
RG 57
DW 22.000 usec
DE 6.00 usec
TE 300.0 K
D1 2.00000000 sec
d11 0.03000000 sec
DELTA 1.89999998 sec
TD0 1
======== CHANNEL f1 ========
NUC1 13C
P1 9.70 usec
PL1 -0.50 dB
SFO1 100.6288660 MHz
======== CHANNEL f2 ========
CPDPRG2 waltz16
NUC2 1H
PCPD2 90.00 usec
PL2 -2.40 dB
PL12 15.10 dB
PL13 18.10 dB
SFO2 400.1516010 MHz
F2 - Processing parameters
SI 32768
SF 100.6178007 MHz
WDW EM
SSB 0
LB 3.00 Hz
GB 0
PC 1.00
RKS-6-36-P
X
O
N
H
H
d1-3a
X = 0.29D
190 180 170 160 150 140 130 120 110 100 90 80 70 60 50 40 30 20 ppm

```
Current Data Parameters
NAME             20171128
EXPNO                  15
PROCNO                  1

F2 - Acquisition Parameters
Date_            20171128
Time                21.46
INSTRUM             spect
PROBHD   5 mm DUL 13C-1
PULPROG              zg30
TD                  32768
SOLVENT             CDCl3
NS                     19
DS                      0
SWH              6410.256 Hz
FIDRES           0.195625 Hz
AQ              2.5559540 sec
RG                      4
DW                 78.000 usec
DE                   6.00 usec
TE                  300.0 K
D1            2.00000000 sec
TD0                     1

======== CHANNEL f1 ========
NUC1                   1H
P1                  10.00 usec
PL1                 -2.40 dB
SFO1          400.1528010 MHz

F2 - Processing parameters
SI                  16384
SF            400.1500174 MHz
WDW                    EM
SSB                     0
LB                   0.00 Hz
GB                      0
PC                   1.00
```

RKS-5-189

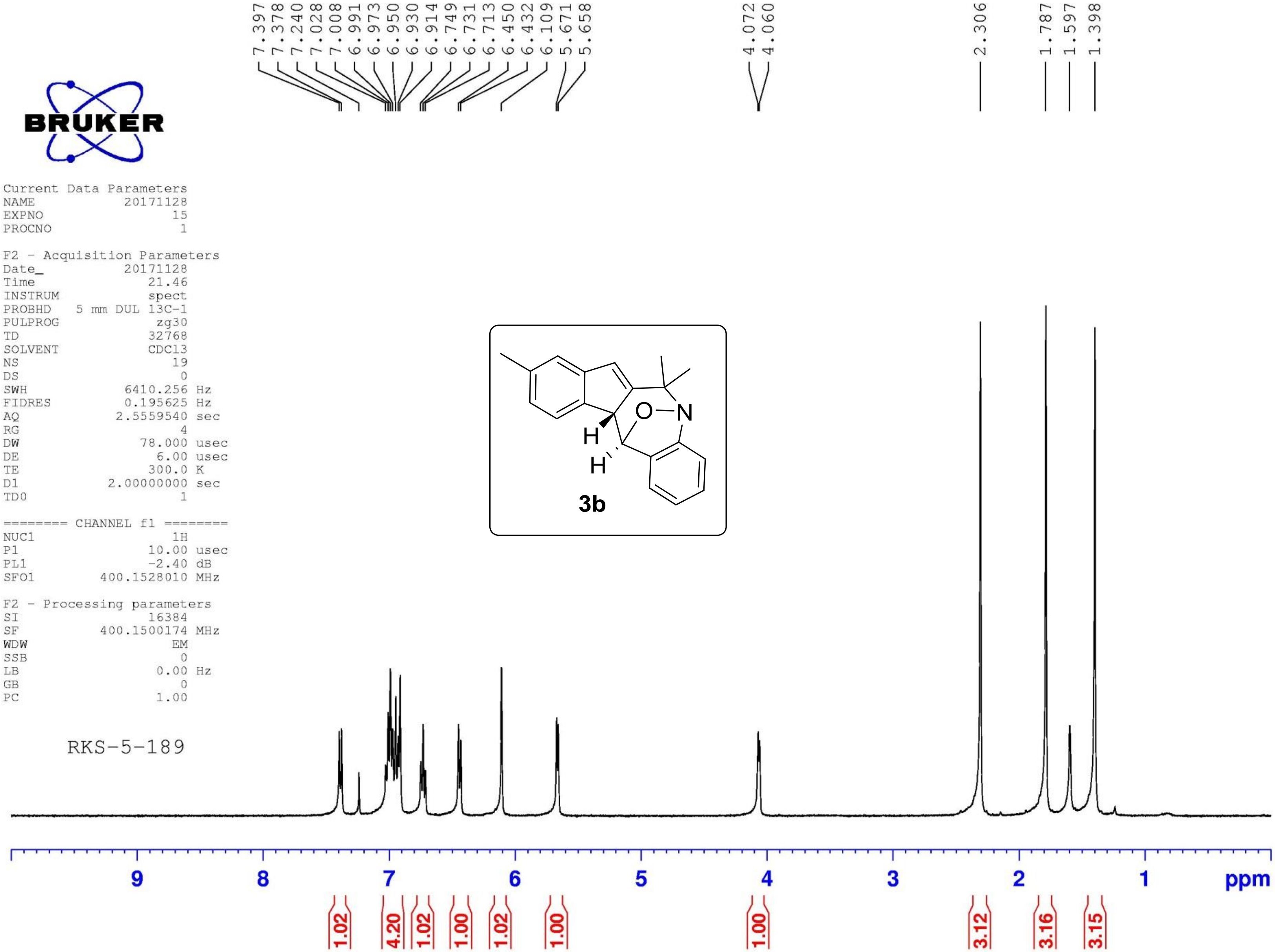

Current Data Parameters
NAME 20171128
EXPNO 2
PROCNO 1

F2 - Acquisition Parameters
Date_ 20171128
Time 0.06
INSTRUM spect
PROBHD 5 mm DUL 13C-1
PULPROG zgpg30
TD 65536
SOLVENT CDCl3
NS 6000
DS 0
SWH 22727.273 Hz
FIDRES 0.346791 Hz
AQ 1.4418420 sec
RG 57
DW 22.000 usec
DE 6.00 usec
TE 300.0 K
D1 2.00000000 sec
d11 0.03000000 sec
DELTA 1.89999998 sec
TD0 1

======== CHANNEL f1 ========
NUC1 13C
P1 9.70 usec
PL1 -0.50 dB
SFO1 100.6288660 MHz

======== CHANNEL f2 ========
CPDPRG2 waltz16
NUC2 1H
PCPD2 90.00 usec
PL2 -2.40 dB
PL12 15.10 dB
PL13 18.10 dB
SFO2 400.1516010 MHz

F2 - Processing parameters
SI 32768
SF 100.6178002 MHz
WDW EM
SSB 0
LB 3.00 Hz
GB 0
PC 1.00

RKS-5-189

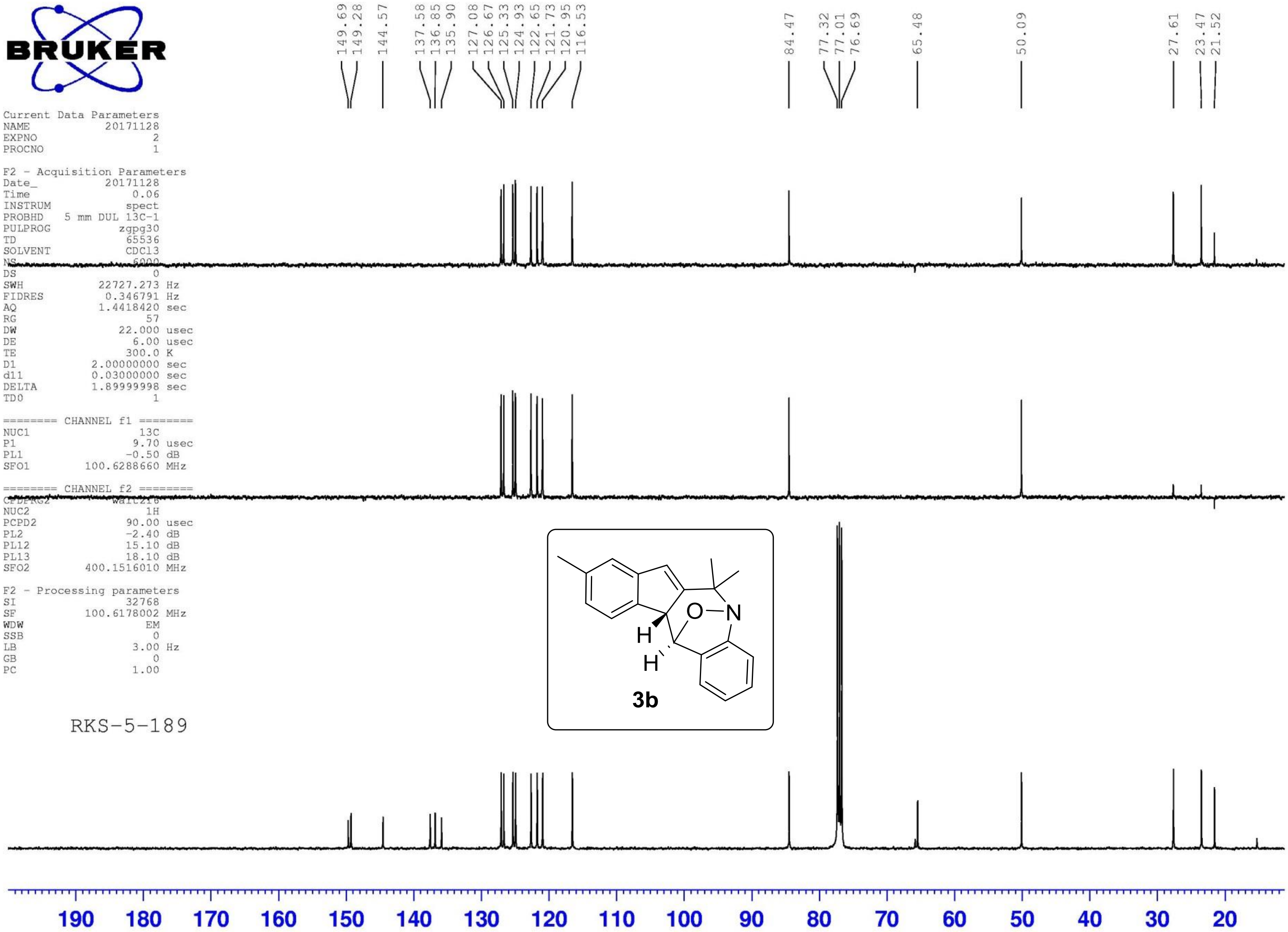

3b

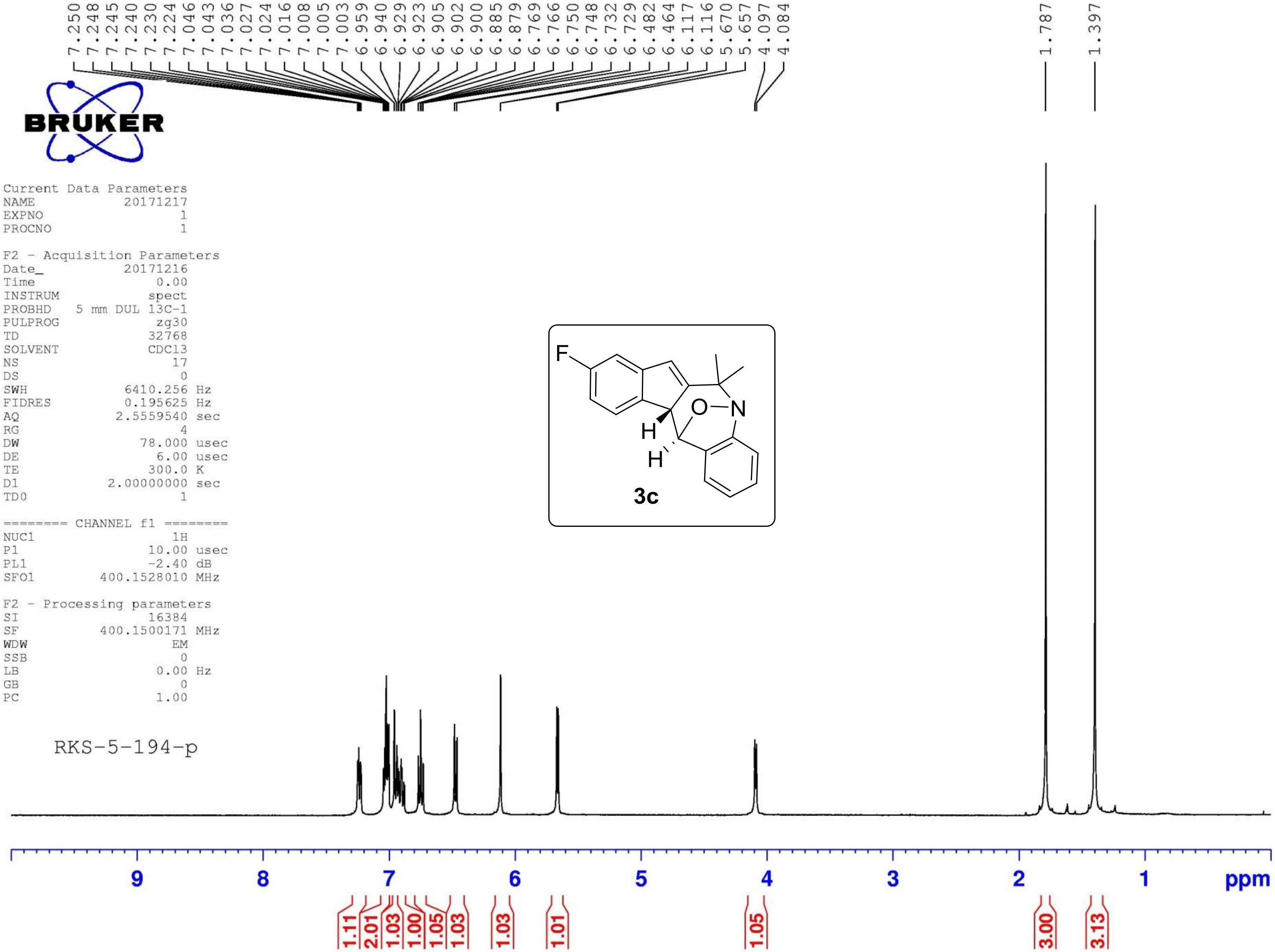
7.250 7.248 7.245 7.240 7.230 7.224 7.046 7.043 7.036 7.027 7.024 7.016 7.008 7.005 7.003 6.959 6.940 6.929 6.923 6.905 6.902 6.900 6.885 6.879 6.769 6.766 6.750 6.748 6.732 6.729 6.482 6.464 6.117 6.116 5.670 5.657 4.097 4.084
1.787
1.397
BRUKER
Current Data Parameters
NAME 20171217
EXPNO 1
PROCNO 1
F2 - Acquisition Parameters
Date_ 20171216
Time 0.00
INSTRUM spect
PROBHD 5 mm DUL 13C-1
PULPROG zg30
TD 32768
SOLVENT CDCl3
NS 17
DS 0
SWH 6410.256 Hz
FIDRES 0.195625 Hz
AQ 2.5559540 sec
RG 4
DW 78.000 usec
DE 6.00 usec
TE 300.0 K
D1 2.00000000 sec
TD0 1
======== CHANNEL f1 ========
NUC1 1H
P1 10.00 usec
PL1 -2.40 dB
SFO1 400.1528010 MHz
F2 - Processing parameters
SI 16384
SF 400.1500171 MHz
WDW EM
SSB 0
LB 0.00 Hz
GB 0
PC 1.00
RKS-5-194-p
9 8 7 6 5 4 3 2 1 ppm
1.11 2.01 1.03 1.00 1.05 1.03 1.03 1.01 1.05 3.00 3.13
F
O
N
H
H

3c

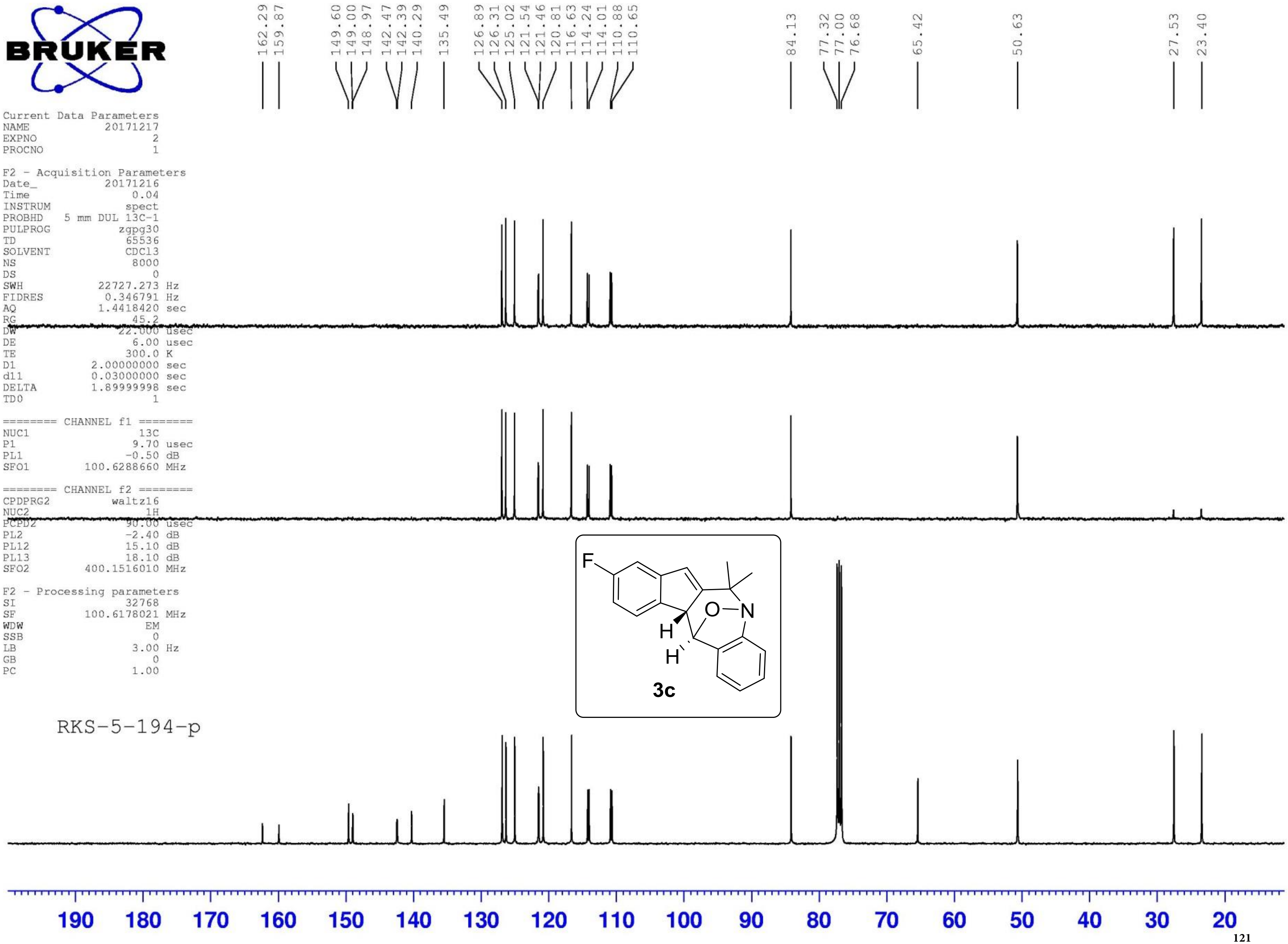
BRUKER
Current Data Parameters
NAME 20171217
EXPNO 2
PROCNO 1
F2 - Acquisition Parameters
Date_ 20171216
Time 0.04
INSTRUM spect
PROBHD 5 mm DUL 13C-1
PULPROG zgpg30
TD 65536
SOLVENT CDCl3
NS 8000
DS 0
SWH 22727.273 Hz
FIDRES 0.346791 Hz
AQ 1.4418420 sec
RG 45.2
DW 22.000 usec
DE 6.00 usec
TE 300.0 K
D1 2.00000000 sec
d11 0.03000000 sec
DELTA 1.89999998 sec
TD0 1
======== CHANNEL f1 ========
NUC1 13C
P1 9.70 usec
PL1 -0.50 dB
SFO1 100.6288660 MHz
======== CHANNEL f2 ========
CPDPRG2 waltz16
NUC2 1H
PCPD2 90.00 usec
PL2 -2.40 dB
PL12 15.10 dB
PL13 18.10 dB
SFO2 400.1516010 MHz
F2 - Processing parameters
SI 32768
SF 100.6178021 MHz
WDW EM
SSB 0
LB 3.00 Hz
GB 0
PC 1.00
RKS-5-194-p
162.29
159.87
149.60
149.00
148.97
142.47
142.39
140.29
135.49
126.89
126.31
125.02
121.54
121.46
120.81
116.63
114.24
114.01
110.88
110.65
84.13
77.32
77.00
76.68
65.42
50.63
27.53
23.40
F
O
N
H
H
3c
190
180
170
160
150
140
130
120
110
100
90
80
70
60
50
40
30
20

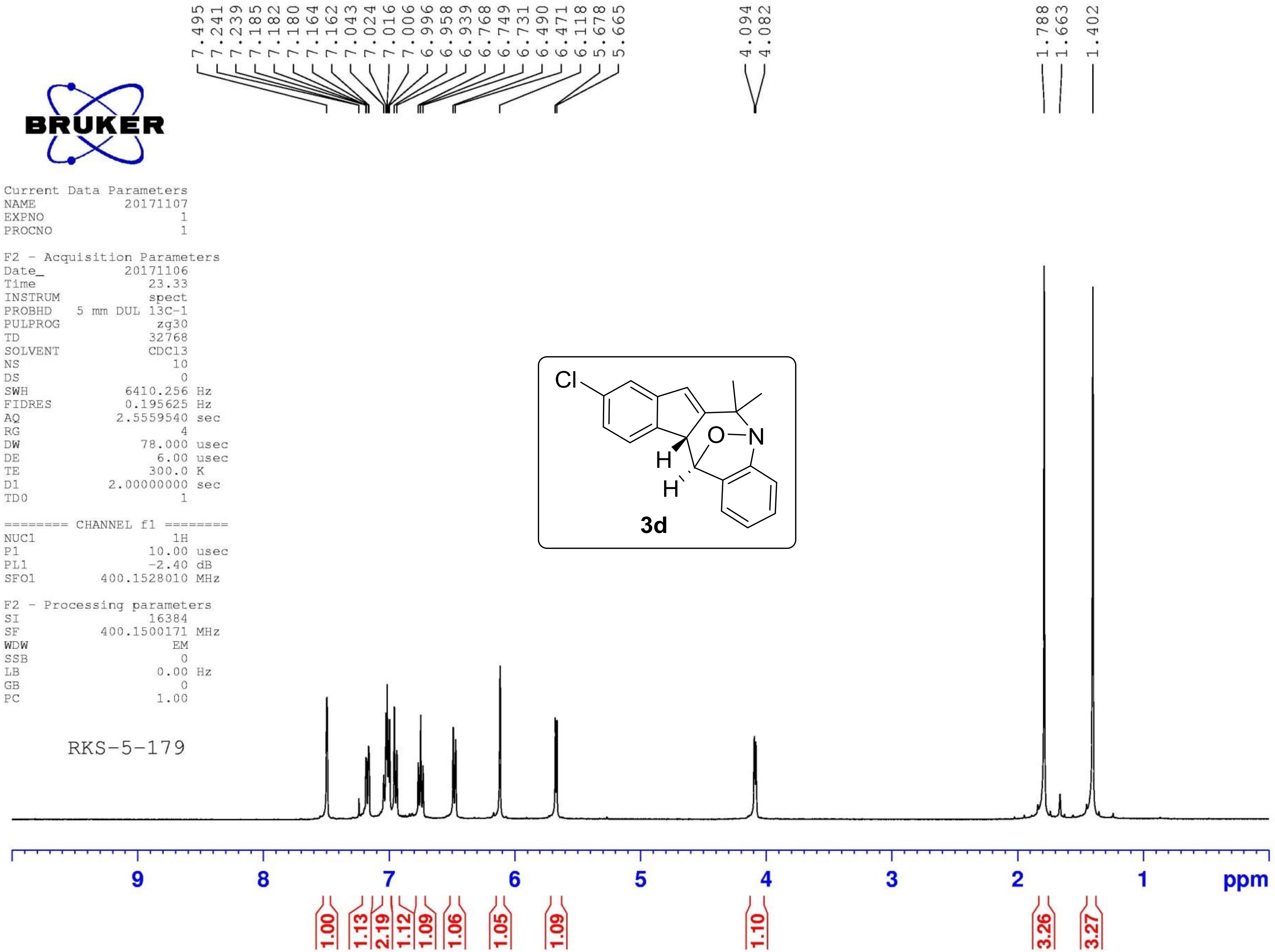
7.495
7.241
7.239
7.185
7.182
7.180
7.164
7.162
7.043
7.024
7.016
7.006
6.996
6.958
6.939
6.768
6.749
6.731
6.490
6.471
6.118
5.678
5.665
4.094
4.082
1.788
1.663
1.402
BRUKER
Current Data Parameters
NAME 20171107
EXPNO 1
PROCNO 1
F2 - Acquisition Parameters
Date_ 20171106
Time 23.33
INSTRUM spect
PROBHD 5 mm DUL 13C-1
PULPROG zg30
TD 32768
SOLVENT CDCl3
NS 10
DS 0
SWH 6410.256 Hz
FIDRES 0.195625 Hz
AQ 2.5559540 sec
RG 4
DW 78.000 usec
DE 6.00 usec
TE 300.0 K
D1 2.00000000 sec
TD0 1
======== CHANNEL f1 ========
NUC1 1H
P1 10.00 usec
PL1 -2.40 dB
SFO1 400.1528010 MHz
F2 - Processing parameters
SI 16384
SF 400.1500171 MHz
WDW EM
SSB 0
LB 0.00 Hz
GB 0
PC 1.00
RKS-5-179
Cl
O
N
H
H
3d
9
8
7
6
5
4
3
2
1
ppm
1.00
1.13
2.19
1.12
1.09
1.06
1.05
1.09
1.10
3.26
3.27

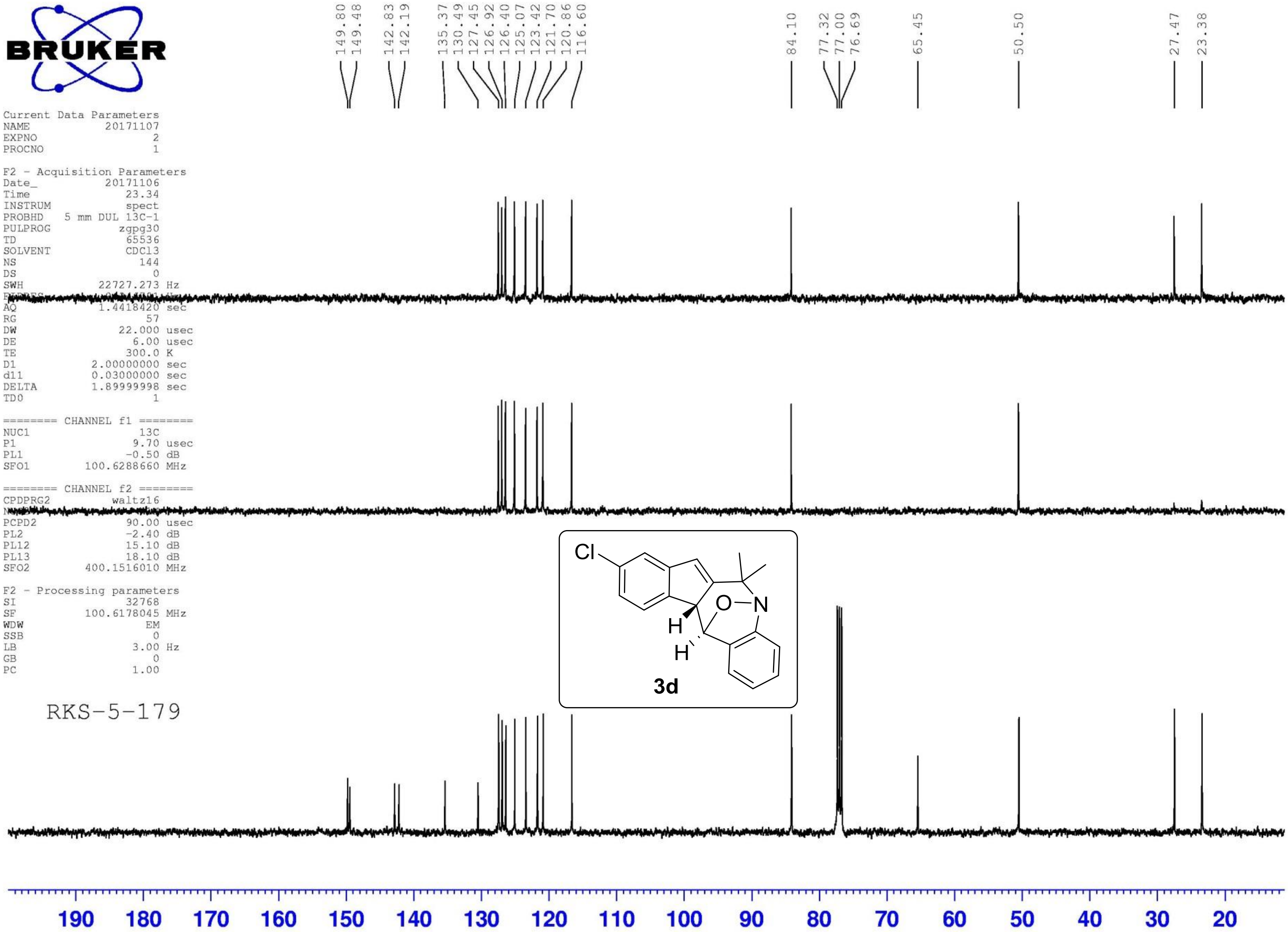

BRUKER
149.80
149.48
142.83
142.19
135.37
130.49
127.45
126.92
126.40
125.07
123.42
121.70
120.86
116.60
84.10
77.32
77.00
76.69
65.45
50.50
27.47
23.38
Current Data Parameters
NAME 20171107
EXPNO 2
PROCNO 1
F2 - Acquisition Parameters
Date_ 20171106
Time 23.34
INSTRUM spect
PROBHD 5 mm DUL 13C-1
PULPROG zgpg30
TD 65536
SOLVENT CDCl3
NS 144
DS 0
SWH 22727.273 Hz
AQ 1.4418420 sec
RG 57
DW 22.000 usec
DE 6.00 usec
TE 300.0 K
D1 2.00000000 sec
d11 0.03000000 sec
DELTA 1.89999998 sec
TD0 1
======== CHANNEL f1 ========
NUC1 13C
P1 9.70 usec
PL1 -0.50 dB
SFO1 100.6288660 MHz
======== CHANNEL f2 ========
CPDPRG2 waltz16
PCPD2 90.00 usec
PL2 -2.40 dB
PL12 15.10 dB
PL13 18.10 dB
SFO2 400.1516010 MHz
F2 - Processing parameters
SI 32768
SF 100.6178045 MHz
WDW EM
SSB 0
LB 3.00 Hz
GB 0
PC 1.00
RKS-5-179
Cl
O
N
H
H
3d
190
180
170
160
150
140
130
120
110
100
90
80
70
60
50
40
30
20

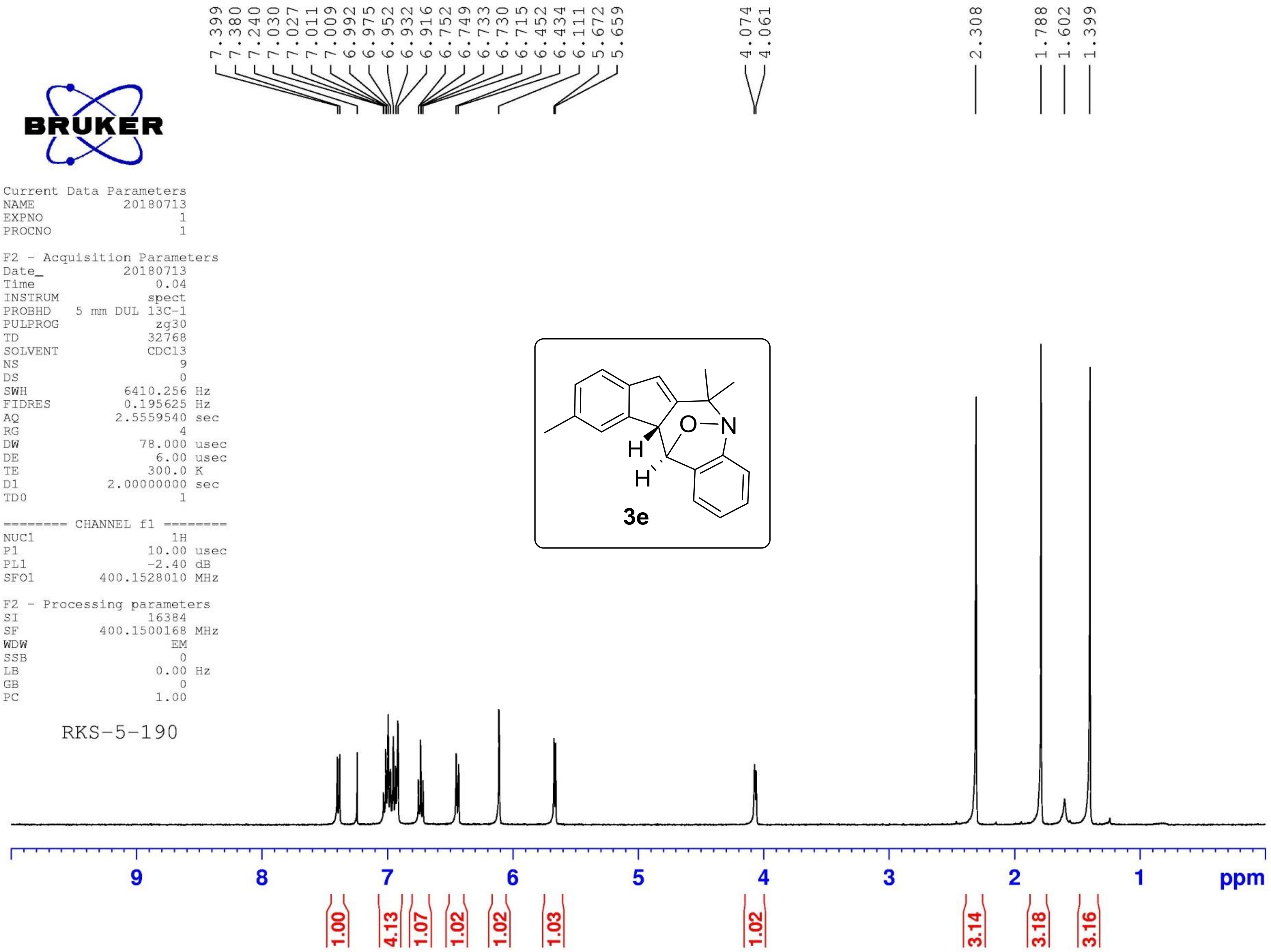
7.399
7.380
7.240
7.030
7.027
7.011
7.009
6.992
6.975
6.952
6.932
6.916
6.752
6.749
6.733
6.730
6.715
6.452
6.434
6.111
5.672
5.659
4.074
4.061
2.308
1.788
1.602
1.399
BRUKER
Current Data Parameters
NAME 20180713
EXPNO 1
PROCNO 1
F2 - Acquisition Parameters
Date_ 20180713
Time 0.04
INSTRUM spect
PROBHD 5 mm DUL 13C-1
PULPROG zg30
TD 32768
SOLVENT CDCl3
NS 9
DS 0
SWH 6410.256 Hz
FIDRES 0.195625 Hz
AQ 2.5559540 sec
RG 4
DW 78.000 usec
DE 6.00 usec
TE 300.0 K
D1 2.00000000 sec
TD0 1
======== CHANNEL f1 ========
NUC1 1H
P1 10.00 usec
PL1 -2.40 dB
SFO1 400.1528010 MHz
F2 - Processing parameters
SI 16384
SF 400.1500168 MHz
WDW EM
SSB 0
LB 0.00 Hz
GB 0
PC 1.00
RKS-5-190
O
N
H
H
3e
9
8
7
6
5
4
3
2
1
ppm
1.00
4.13
1.07
1.02
1.02
1.03
1.02
3.14
3.18
3.16

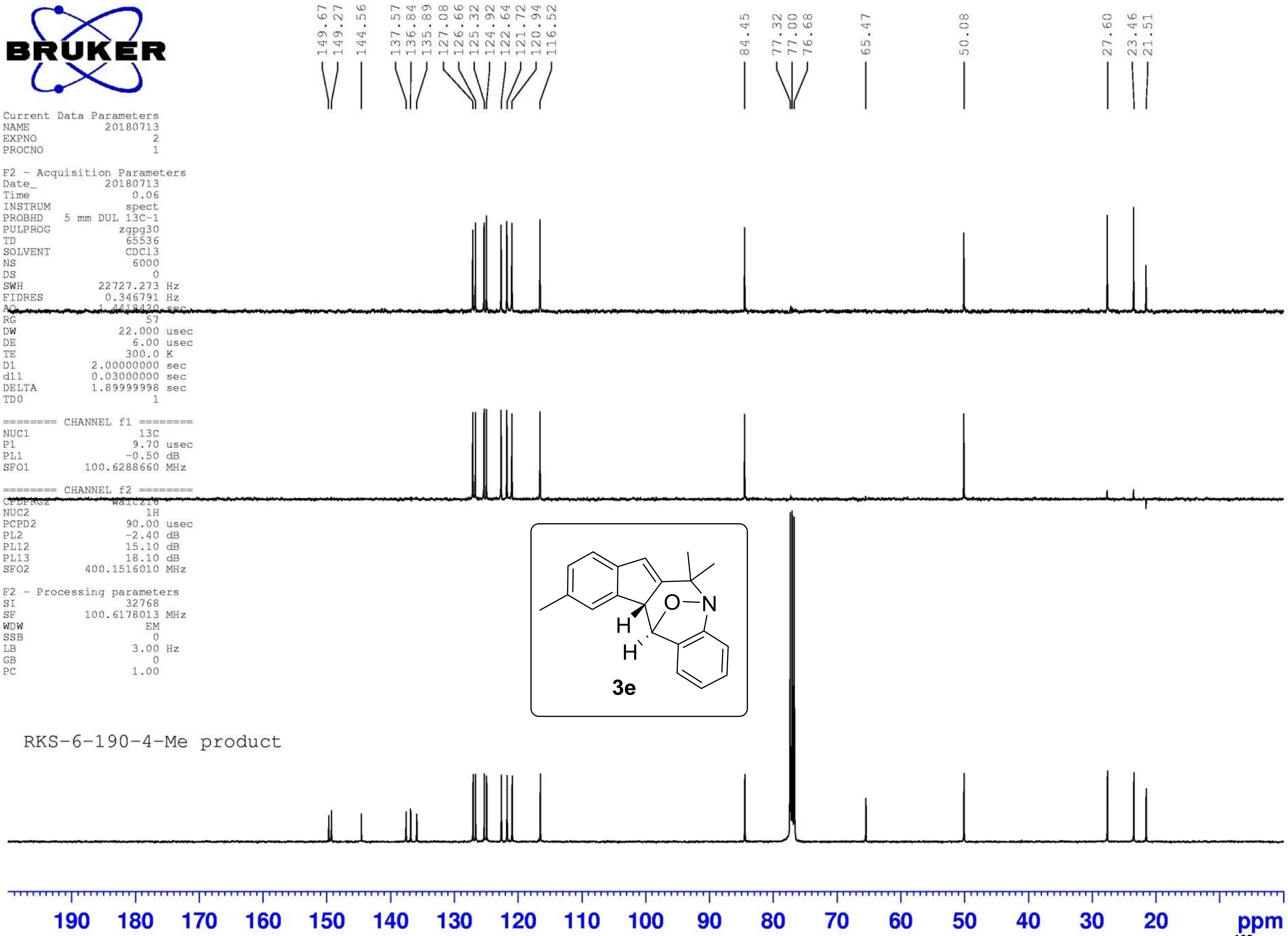
149.67
149.27
144.56
137.57
136.84
135.89
127.08
126.66
125.32
124.92
122.64
121.72
120.94
116.52
84.45
77.32
77.00
76.68
65.47
50.08
27.60
23.46
21.51
BRUKER
Current Data Parameters
NAME 20180713
EXPNO 2
PROCNO 1
F2 - Acquisition Parameters
Date_ 20180713
Time 0.06
INSTRUM spect
PROBHD 5 mm DUL 13C-1
PULPROG zgpg30
TD 65536
SOLVENT CDCl3
NS 6000
DS 0
SWH 22727.273 Hz
FIDRES 0.346791 Hz
AQ
RG 57
DW 22.000 usec
DE 6.00 usec
TE 300.0 K
D1 2.00000000 sec
d11 0.03000000 sec
DELTA 1.89999998 sec
TD0 1
======== CHANNEL f1 ========
NUC1 13C
P1 9.70 usec
PL1 -0.50 dB
SFO1 100.6288660 MHz
======== CHANNEL f2 ========
CPDPRG2 waltz16
NUC2 1H
PCPD2 90.00 usec
PL2 -2.40 dB
PL12 15.10 dB
PL13 18.10 dB
SFO2 400.1516010 MHz
F2 - Processing parameters
SI 32768
SF 100.6178013 MHz
WDW EM
SSB 0
LB 3.00 Hz
GB 0
PC 1.00
O
N
H
H
3e
RKS-6-190-4-Me product
190 180 170 160 150 140 130 120 110 100 90 80 70 60 50 40 30 20 ppm

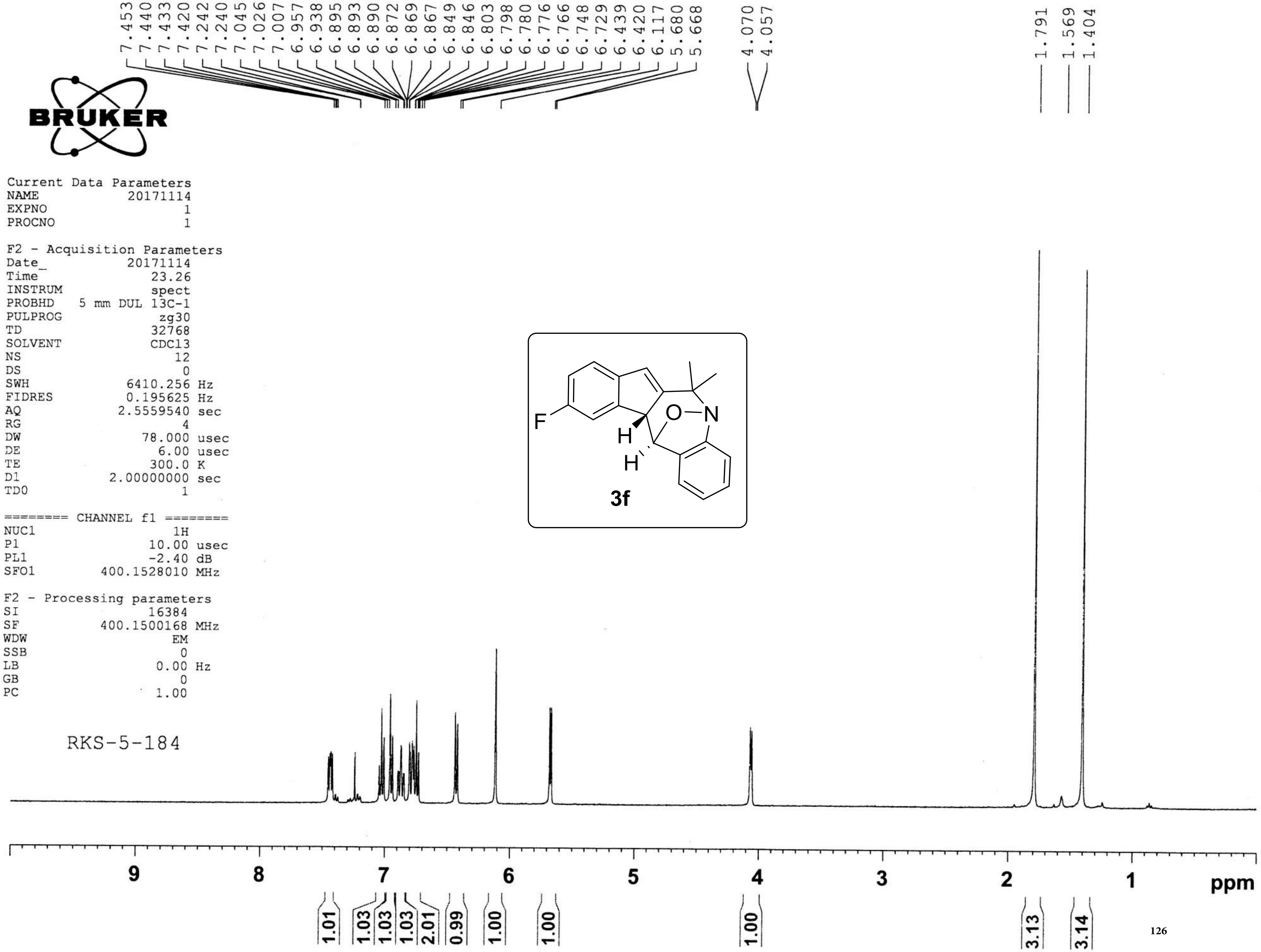

7.453
7.440
7.433
7.420
7.242
7.240
7.045
7.026
7.007
6.957
6.938
6.895
6.893
6.890
6.872
6.869
6.867
6.849
6.846
6.803
6.798
6.780
6.776
6.766
6.748
6.729
6.439
6.420
6.117
5.680
5.668
4.070
4.057
1.791
1.569
1.404
BRUKER
Current Data Parameters
NAME 20171114
EXPNO 1
PROCNO 1
F2 - Acquisition Parameters
Date_ 20171114
Time 23.26
INSTRUM spect
PROBHD 5 mm DUL 13C-1
PULPROG zg30
TD 32768
SOLVENT CDCl3
NS 12
DS 0
SWH 6410.256 Hz
FIDRES 0.195625 Hz
AQ 2.5559540 sec
RG 4
DW 78.000 usec
DE 6.00 usec
TE 300.0 K
D1 2.00000000 sec
TD0 1
======== CHANNEL f1 ========
NUC1 1H
P1 10.00 usec
PL1 -2.40 dB
SFO1 400.1528010 MHz
F2 - Processing parameters
SI 16384
SF 400.1500168 MHz
WDW EM
SSB 0
LB 0.00 Hz
GB 0
PC 1.00
RKS-5-184
F
O
N
H
H
3f
9
8
7
6
5
4
3
2
1
ppm
1.01
1.03
1.03
1.03
2.01
0.99
1.00
1.00
1.00
3.13
3.14

```
Current Data Parameters
NAME          20171115
EXPNO               10
PROCNO               1

F2 - Acquisition Parameters
Date_         20171115
Time             10.31
INSTRUM          spect
PROBHD   5 mm DUL 13C-1
PULPROG        zgpg30
TD              65536
SOLVENT         CDCl3
NS               1000
DS                  0
SWH         22727.273 Hz
FIDRES       0.346791 Hz
AQ          1.4418420 sec
RG               50.8
DW             22.000 usec
DE               6.00 usec
TE              300.0 K
D1         2.00000000 sec
d11        0.03000000 sec
DELTA      1.89999998 sec
TD0                 1

======== CHANNEL f1 ========
NUC1              13C
P1               9.70 usec
PL1             -0.50 dB
SFO1      100.6288660 MHz

======== CHANNEL f2 ========
CPDPRG2        waltz16
NUC2                1H
PCPD2           90.00 usec
PL2             -2.40 dB
PL12            15.10 dB
PL13            18.10 dB
SFO2      400.1516010 MHz

F2 - Processing parameters
SI              32768
SF        100.6178002 MHz
WDW                EM
SSB                 0
LB               3.00 Hz
GB                  0
PC               1.00
```

RKS-5-184

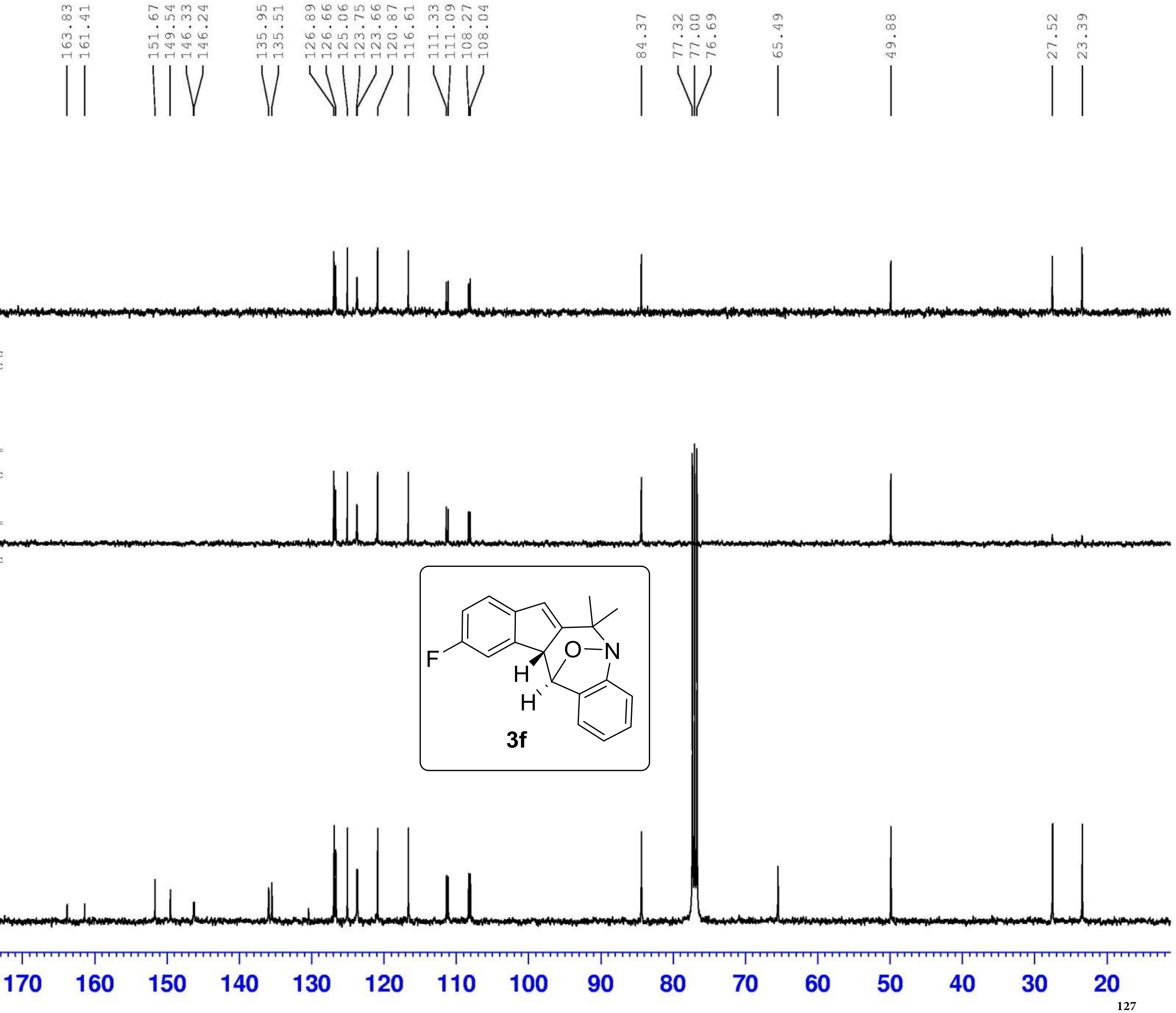

3f

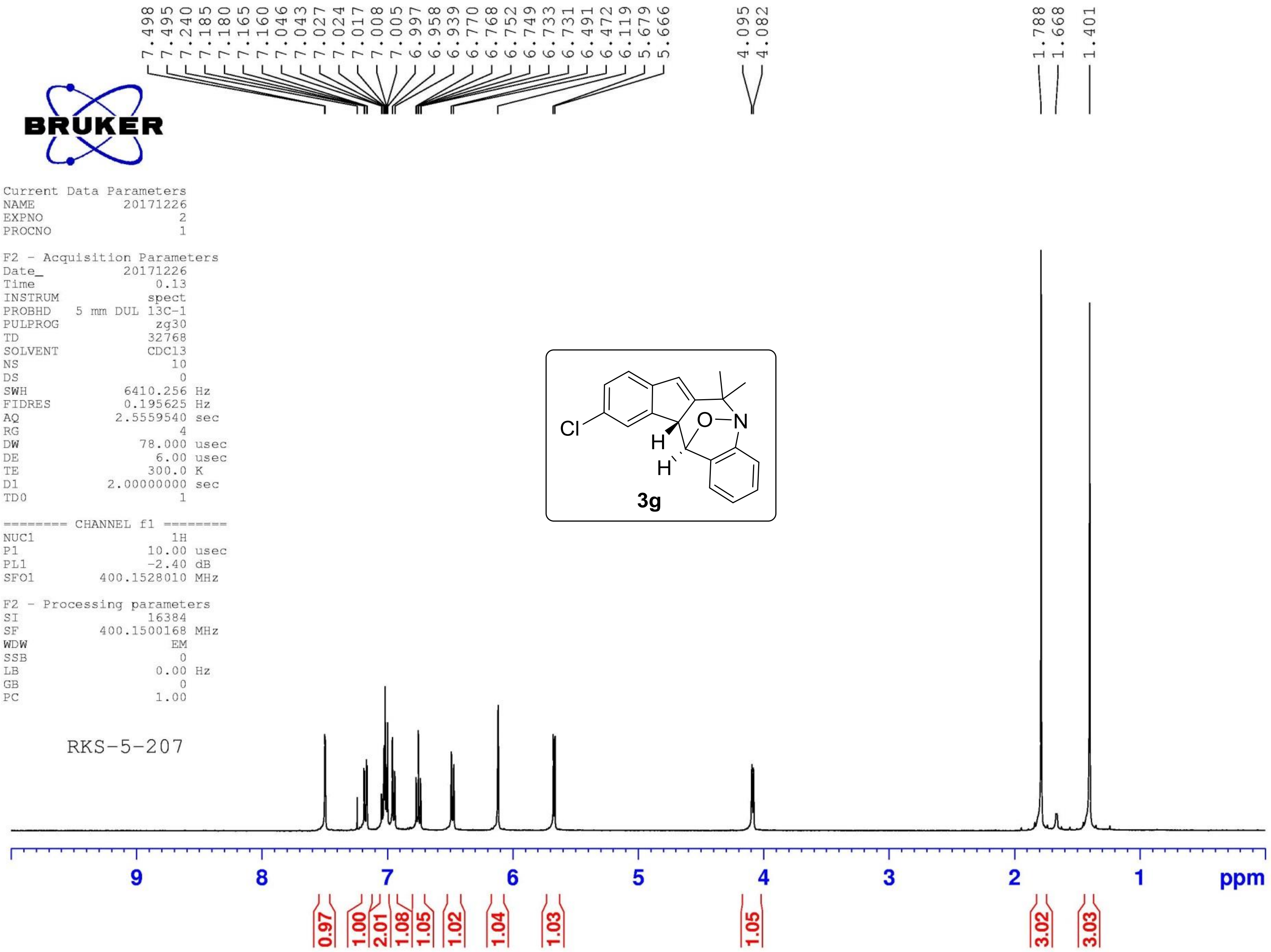

BRUKER

Current Data Parameters
NAME 20171226
EXPNO 2
PROCNO 1

F2 - Acquisition Parameters
Date_ 20171226
Time 0.13
INSTRUM spect
PROBHD 5 mm DUL 13C-1
PULPROG zg30
TD 32768
SOLVENT CDCl3
NS 10
DS 0
SWH 6410.256 Hz
FIDRES 0.195625 Hz
AQ 2.5559540 sec
RG 4
DW 78.000 usec
DE 6.00 usec
TE 300.0 K
D1 2.00000000 sec
TD0 1

======== CHANNEL f1 ========
NUC1 1H
P1 10.00 usec
PL1 -2.40 dB
SFO1 400.1528010 MHz

F2 - Processing parameters
SI 16384
SF 400.1500168 MHz
WDW EM
SSB 0
LB 0.00 Hz
GB 0
PC 1.00

RKS-5-207

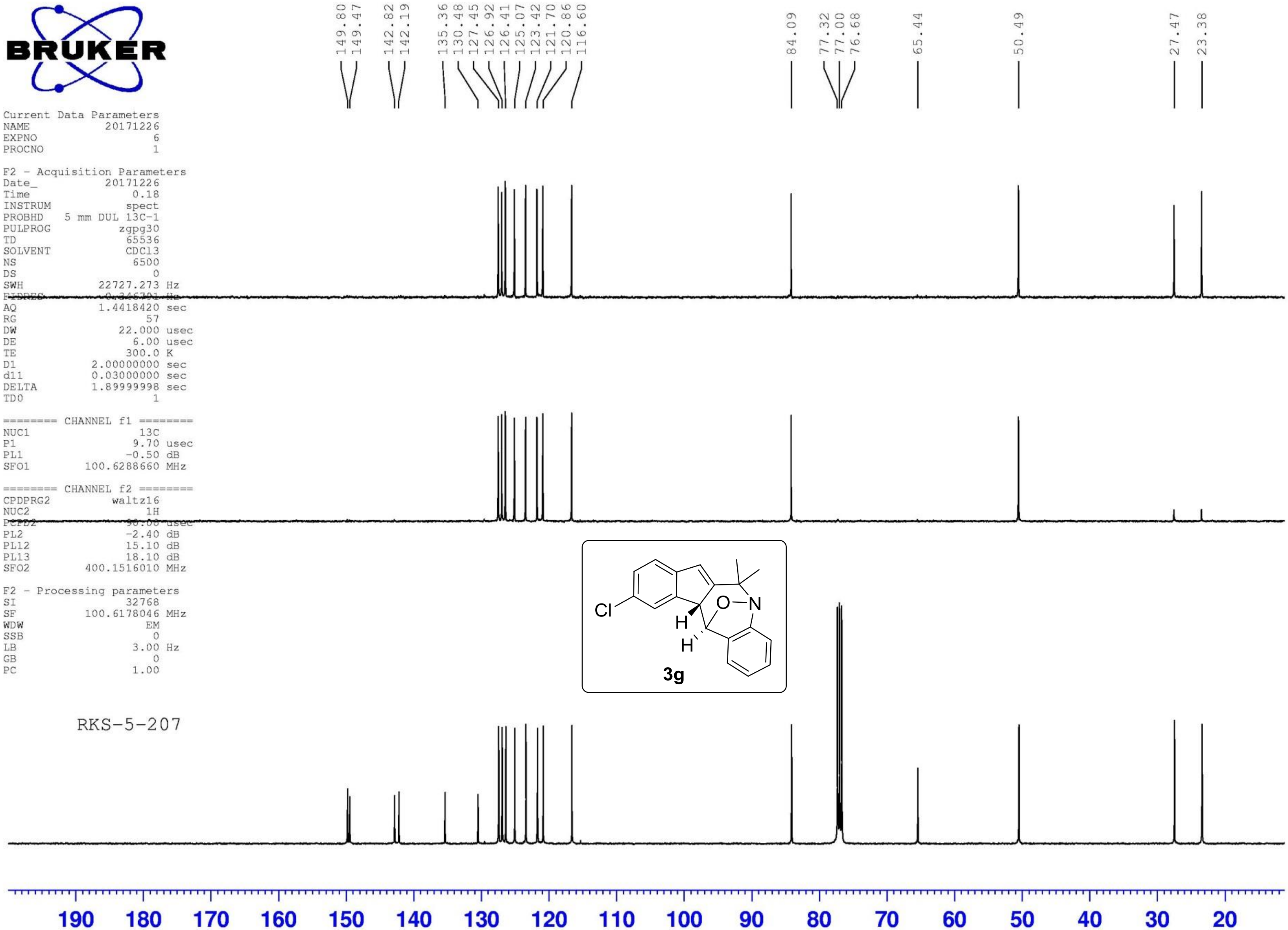
BRUKER
149.80
149.47
142.82
142.19
135.36
130.48
127.45
126.92
126.41
125.07
123.42
121.70
120.86
116.60
84.09
77.32
77.00
76.68
65.44
50.49
27.47
23.38
Current Data Parameters
NAME 20171226
EXPNO 6
PROCNO 1
F2 - Acquisition Parameters
Date_ 20171226
Time 0.18
INSTRUM spect
PROBHD 5 mm DUL 13C-1
PULPROG zgpg30
TD 65536
SOLVENT CDCl3
NS 6500
DS 0
SWH 22727.273 Hz
FIDRES 0.346791 Hz
AQ 1.4418420 sec
RG 57
DW 22.000 usec
DE 6.00 usec
TE 300.0 K
D1 2.00000000 sec
d11 0.03000000 sec
DELTA 1.89999998 sec
TD0 1
======== CHANNEL f1 ========
NUC1 13C
P1 9.70 usec
PL1 -0.50 dB
SFO1 100.6288660 MHz
======== CHANNEL f2 ========
CPDPRG2 waltz16
NUC2 1H
PCPD2 90.00 usec
PL2 -2.40 dB
PL12 15.10 dB
PL13 18.10 dB
SFO2 400.1516010 MHz
F2 - Processing parameters
SI 32768
SF 100.6178046 MHz
WDW EM
SSB 0
LB 3.00 Hz
GB 0
PC 1.00
RKS-5-207
Cl
O
N
H
H
3g
190 180 170 160 150 140 130 120 110 100 90 80 70 60 50 40 30 20

7.522 7.507 7.503 7.241 7.213 7.198 7.195 7.189 7.183 7.177 7.172 7.168 7.153 7.107 7.102 7.094 7.089 7.087 7.034 7.031 7.015 7.012 6.996 6.993 6.954 6.936 6.730 6.727 6.711 6.709 6.693 6.691 6.416 6.398 6.175 5.709 5.697 4.051 4.038 2.590 2.576 2.559 2.181 2.169 2.160 2.149 2.024 2.012 2.002 1.997 1.992 1.986 1.982 1.974 1.961 1.855 1.844 1.833 1.825 1.815 1.804 1.749 1.729 1.562 1.238

BRUKER

```
Current Data Parameters
NAME              20171107
EXPNO                    5
PROCNO                   1

F2 - Acquisition Parameters
Date_             20171107
Time                  0.02
INSTRUM              spect
PROBHD   5 mm DUL 13C-1
PULPROG               zg30
TD                   32768
SOLVENT              CDCl3
NS                      12
DS                       0
SWH               6410.256 Hz
FIDRES            0.195625 Hz
AQ               2.5559540 sec
RG                       4
DW                  78.000 usec
DE                    6.00 usec
TE                   300.0 K
D1              2.00000000 sec
TD0                      1

======== CHANNEL f1 ========
NUC1                    1H
P1                   10.00 usec
PL1                  -2.40 dB
SFO1          400.1528010 MHz

F2 - Processing parameters
SI                   16384
SF            400.1500166 MHz
WDW                     EM
SSB                      0
LB                    0.00 Hz
GB                       0
PC                    1.00
```

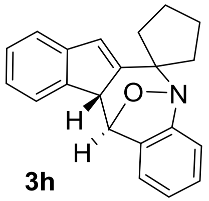

3h

RKS-5-174

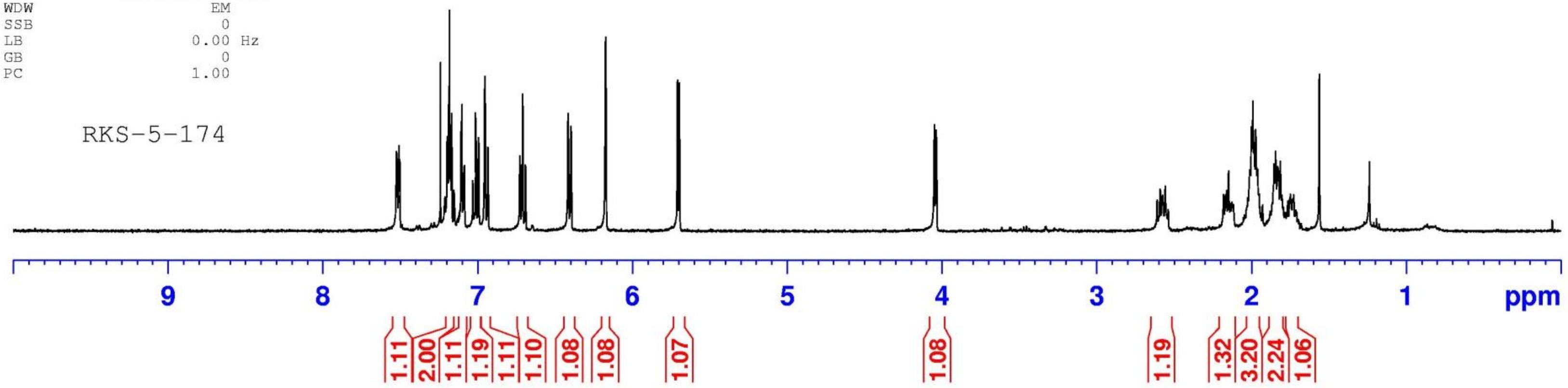

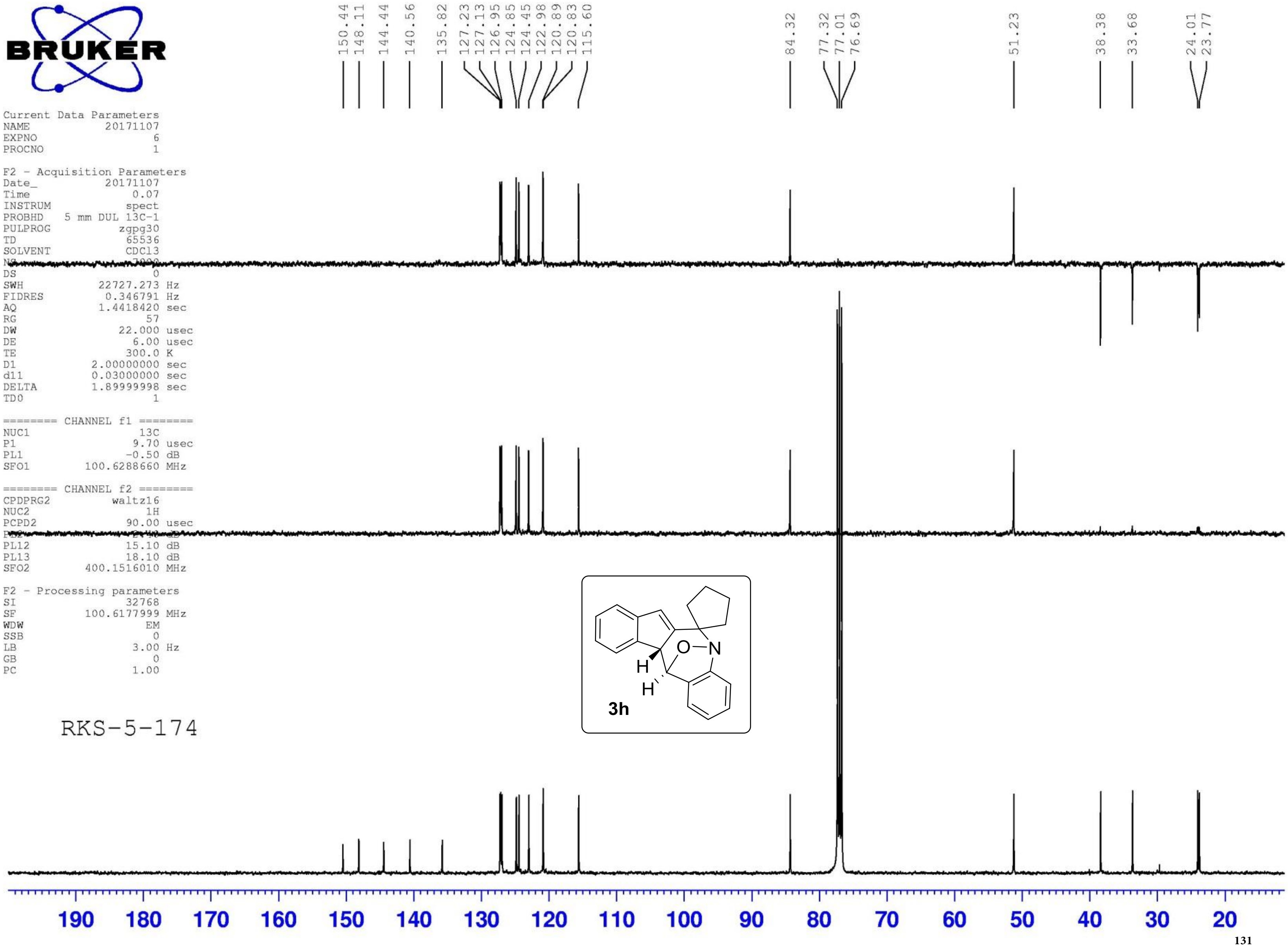
BRUKER
150.44
148.11
144.44
140.56
135.82
127.23
127.13
126.95
124.85
124.45
122.98
120.89
120.83
115.60
84.32
77.32
77.01
76.69
51.23
38.38
33.68
24.01
23.77
Current Data Parameters
NAME 20171107
EXPNO 6
PROCNO 1
F2 - Acquisition Parameters
Date_ 20171107
Time 0.07
INSTRUM spect
PROBHD 5 mm DUL 13C-1
PULPROG zgpg30
TD 65536
SOLVENT CDCl3
DS 0
SWH 22727.273 Hz
FIDRES 0.346791 Hz
AQ 1.4418420 sec
RG 57
DW 22.000 usec
DE 6.00 usec
TE 300.0 K
D1 2.00000000 sec
d11 0.03000000 sec
DELTA 1.89999998 sec
TD0 1
======== CHANNEL f1 ========
NUC1 13C
P1 9.70 usec
PL1 -0.50 dB
SFO1 100.6288660 MHz
======== CHANNEL f2 ========
CPDPRG2 waltz16
NUC2 1H
PCPD2 90.00 usec
PL12 15.10 dB
PL13 18.10 dB
SFO2 400.1516010 MHz
F2 - Processing parameters
SI 32768
SF 100.6177999 MHz
WDW EM
SSB 0
LB 3.00 Hz
GB 0
PC 1.00
RKS-5-174
O
N
H
H
3h
190
180
170
160
150
140
130
120
110
100
90
80
70
60
50
40
30
20

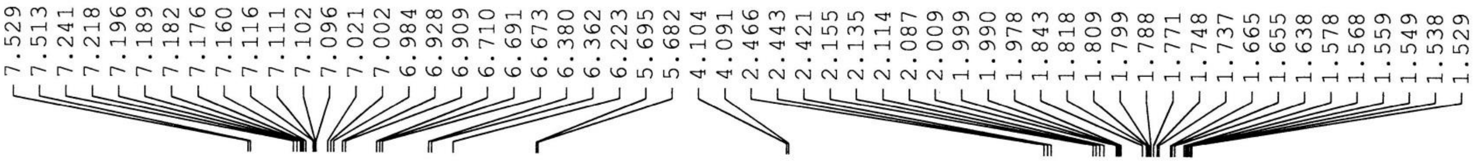

BRUKER

Current Data Parameters
NAME 20171024
EXPNO 20
PROCNO 1

F2 - Acquisition Parameters
Date_ 20171024
Time 17.37
INSTRUM spect
PROBHD 5 mm DUL 13C-1
PULPROG zg30
TD 32768
SOLVENT CDCl3
NS 13
DS 0
SWH 6410.256 Hz
FIDRES 0.195625 Hz
AQ 2.5559540 sec
RG 4
DW 78.000 usec
DE 6.00 usec
TE 300.0 K
D1 2.00000000 sec
TD0 1

======== CHANNEL f1 ========
NUC1 1H
P1 10.00 usec
PL1 -2.40 dB
SFO1 400.1528010 MHz

F2 - Processing parameters
SI 16384
SF 400.1500168 MHz
WDW EM
SSB 0
LB 0.00 Hz
GB 0
PC 1.00

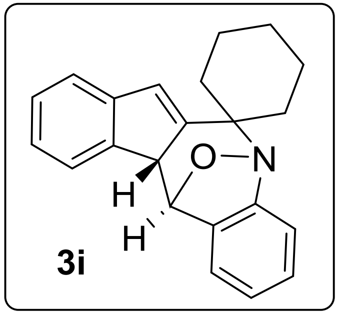

3i

RKS-5-168

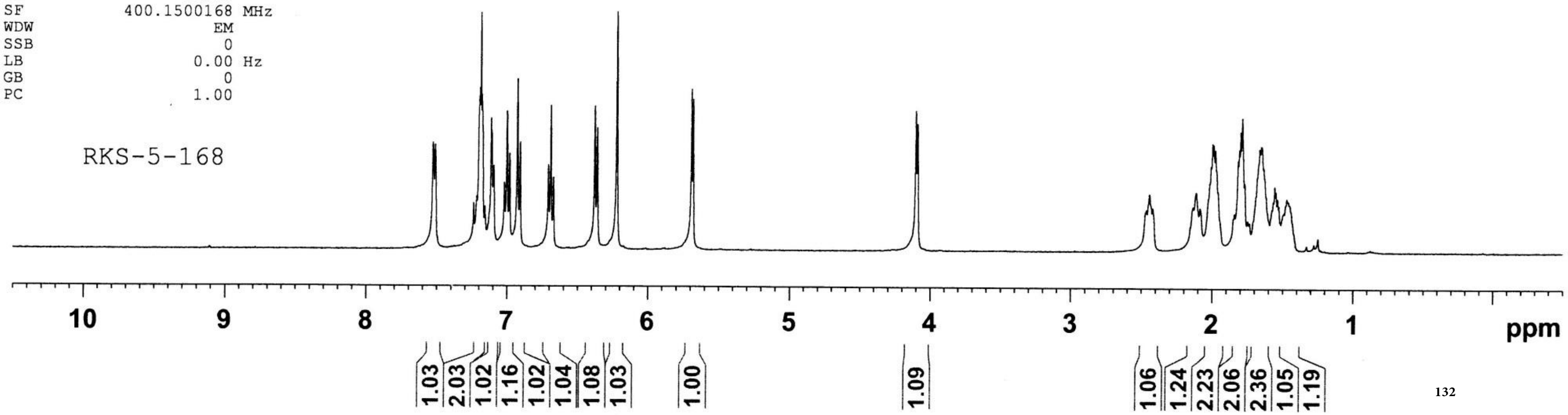

Current Data Parameters
NAME 20171025
EXPNO 2
PROCNO 1

F2 - Acquisition Parameters
Date_ 20171024
Time 23.13
INSTRUM spect
PROBHD 5 mm DUL 13C-1
PULPROG zgpg30
TD 65536
SOLVENT CDCl3
NS 600
[illegible]
SWH 22727.273 Hz
FIDRES 0.346791 Hz
AQ 1.4418420 sec
RG 40.3
DW 22.000 usec
DE 6.00 usec
TE 300.0 K
D1 2.00000000 sec
d11 0.03000000 sec
DELTA 1.89999998 sec
TD0 1

======== CHANNEL f1 ========
NUC1 13C
P1 9.70 usec
PL1 -0.50 dB
SFO1 100.6288660 MHz

======== CHANNEL f2 ========
CPDPRG2 waltz16
NUC2 [illegible]
PCPD2 90.00 usec
PL2 -2.40 dB
PL12 15.10 dB
PL13 18.10 dB
SFO2 400.1516010 MHz

F2 - Processing parameters
SI 32768
SF 100.6178045 MHz
WDW EM
SSB 0
LB 3.00 Hz
GB 0
PC 1.00

RKS-5-168

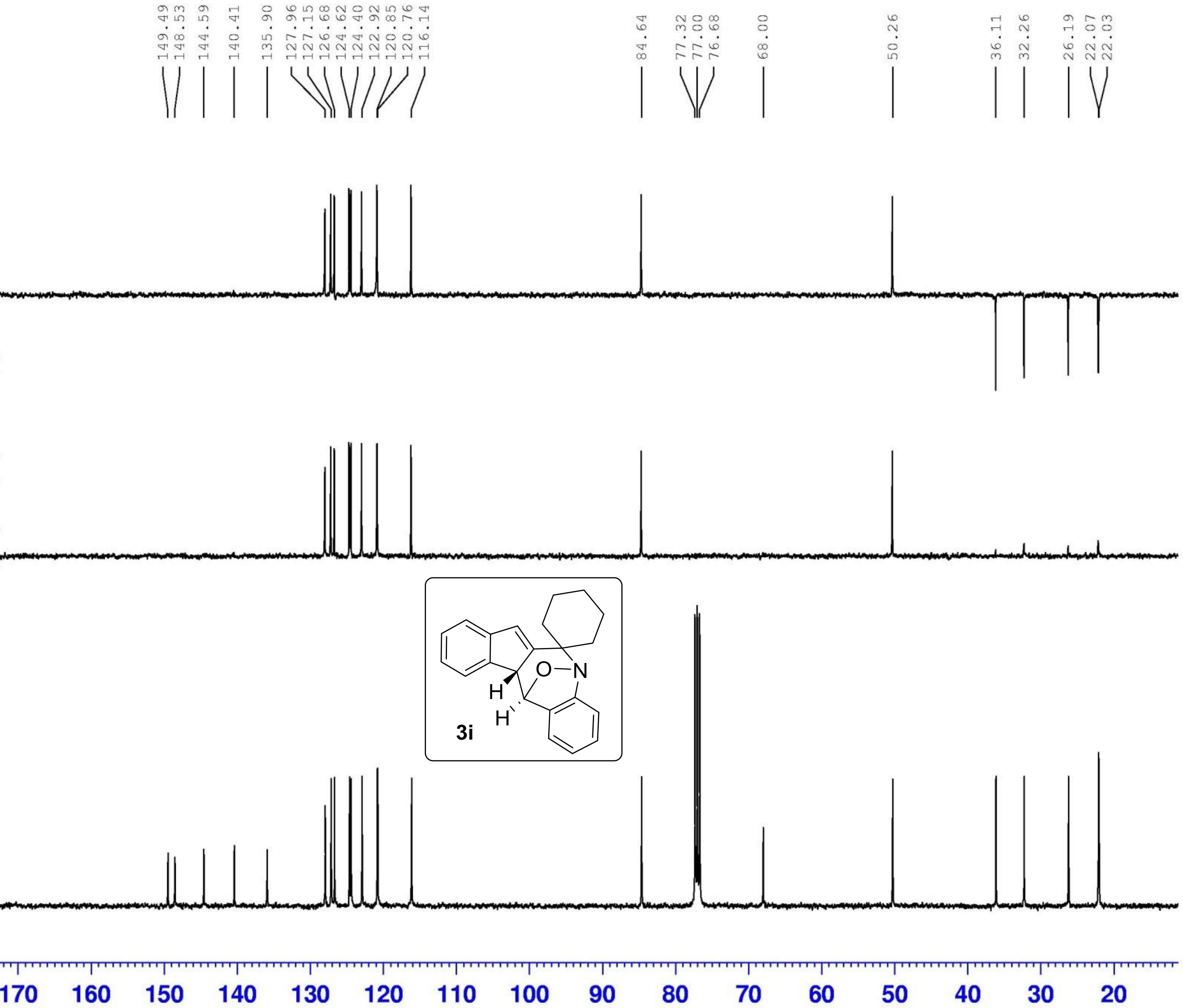

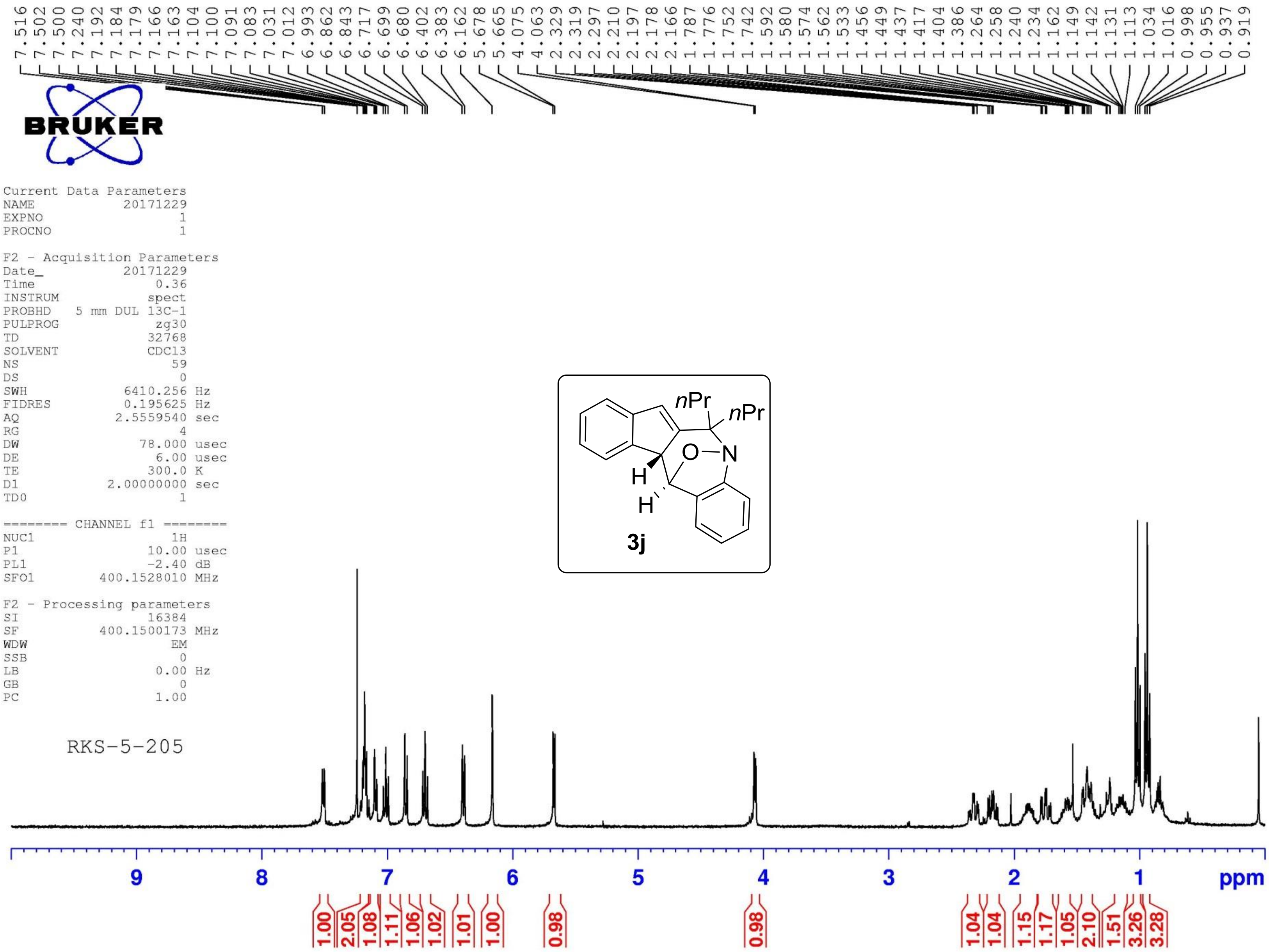
7.516
7.502
7.500
7.240
7.192
7.184
7.179
7.166
7.163
7.104
7.100
7.091
7.083
7.031
7.012
6.993
6.862
6.843
6.717
6.699
6.680
6.402
6.383
6.162
5.678
5.665
4.075
4.063
2.329
2.319
2.297
2.210
2.197
2.178
2.166
1.787
1.776
1.752
1.742
1.592
1.580
1.574
1.562
1.533
1.456
1.449
1.437
1.417
1.404
1.386
1.264
1.258
1.240
1.234
1.162
1.149
1.142
1.131
1.113
1.034
1.016
0.998
0.955
0.937
0.919
BRUKER
Current Data Parameters
NAME 20171229
EXPNO 1
PROCNO 1
F2 - Acquisition Parameters
Date_ 20171229
Time 0.36
INSTRUM spect
PROBHD 5 mm DUL 13C-1
PULPROG zg30
TD 32768
SOLVENT CDCl3
NS 59
DS 0
SWH 6410.256 Hz
FIDRES 0.195625 Hz
AQ 2.5559540 sec
RG 4
DW 78.000 usec
DE 6.00 usec
TE 300.0 K
D1 2.00000000 sec
TD0 1
======== CHANNEL f1 ========
NUC1 1H
P1 10.00 usec
PL1 -2.40 dB
SFO1 400.1528010 MHz
F2 - Processing parameters
SI 16384
SF 400.1500173 MHz
WDW EM
SSB 0
LB 0.00 Hz
GB 0
PC 1.00
RKS-5-205
nPr
nPr
O
N
H
H
3j
9
8
7
6
5
4
3
2
1
ppm
1.00
2.05
1.08
1.11
1.06
1.02
1.01
1.00
0.98
0.98
1.04
1.04
1.15
1.17
1.05
2.10
1.51
3.26
3.28

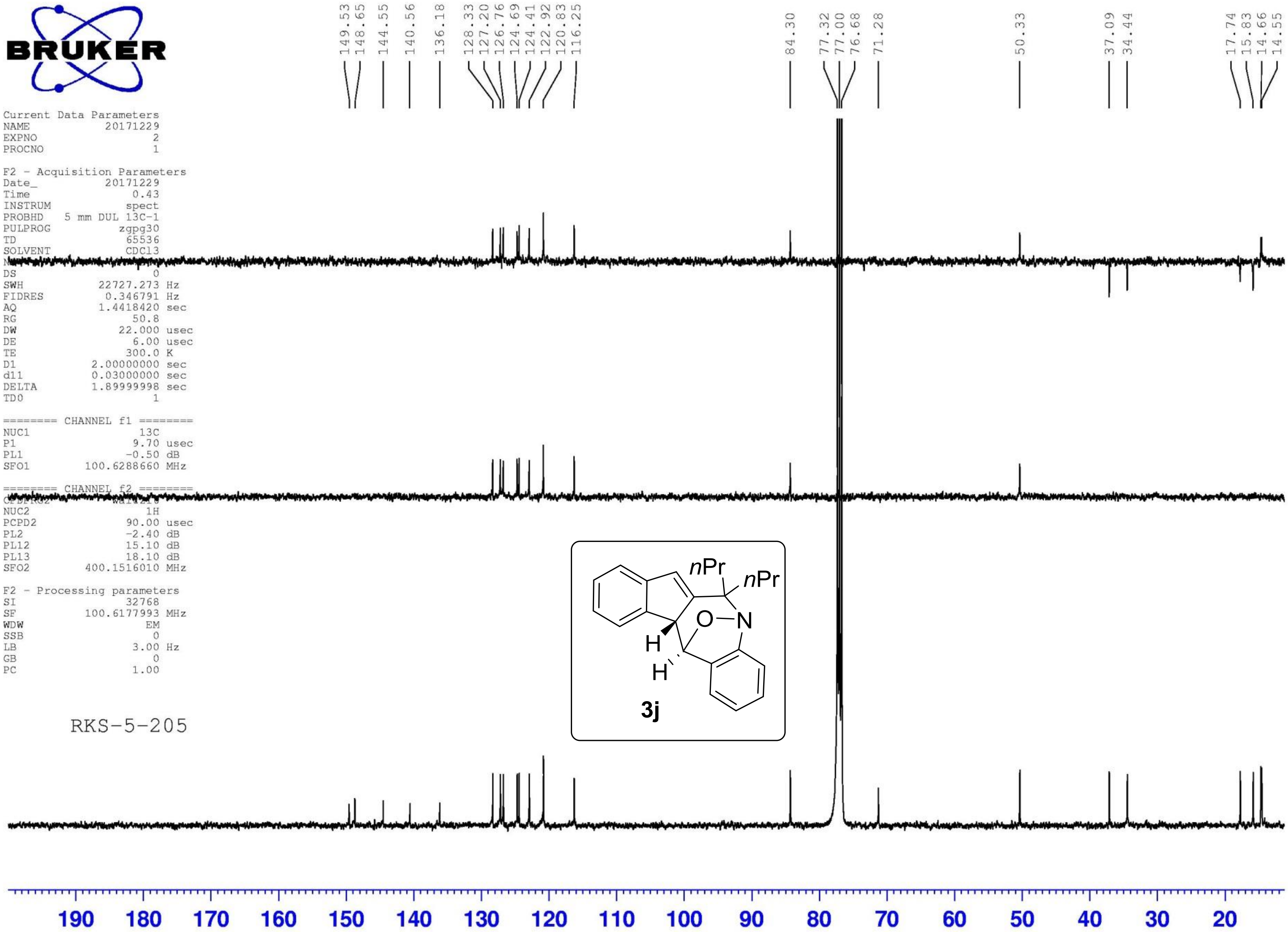
BRUKER
149.53
148.65
144.55
140.56
136.18
128.33
127.20
126.76
124.69
124.41
122.92
120.83
116.25
84.30
77.32
77.00
76.68
71.28
50.33
37.09
34.44
17.74
15.83
14.66
14.55
Current Data Parameters
NAME 20171229
EXPNO 2
PROCNO 1
F2 - Acquisition Parameters
Date_ 20171229
Time 0.43
INSTRUM spect
PROBHD 5 mm DUL 13C-1
PULPROG zgpg30
TD 65536
SOLVENT CDCl3
DS 0
SWH 22727.273 Hz
FIDRES 0.346791 Hz
AQ 1.4418420 sec
RG 50.8
DW 22.000 usec
DE 6.00 usec
TE 300.0 K
D1 2.00000000 sec
d11 0.03000000 sec
DELTA 1.89999998 sec
TD0 1
======== CHANNEL f1 ========
NUC1 13C
P1 9.70 usec
PL1 -0.50 dB
SFO1 100.6288660 MHz
======== CHANNEL f2 ========
NUC2 1H
PCPD2 90.00 usec
PL2 -2.40 dB
PL12 15.10 dB
PL13 18.10 dB
SFO2 400.1516010 MHz
F2 - Processing parameters
SI 32768
SF 100.6177993 MHz
WDW EM
SSB 0
LB 3.00 Hz
GB 0
PC 1.00
RKS-5-205
nPr
nPr
O
N
H
H
3j
190
180
170
160
150
140
130
120
110
100
90
80
70
60
50
40
30
20

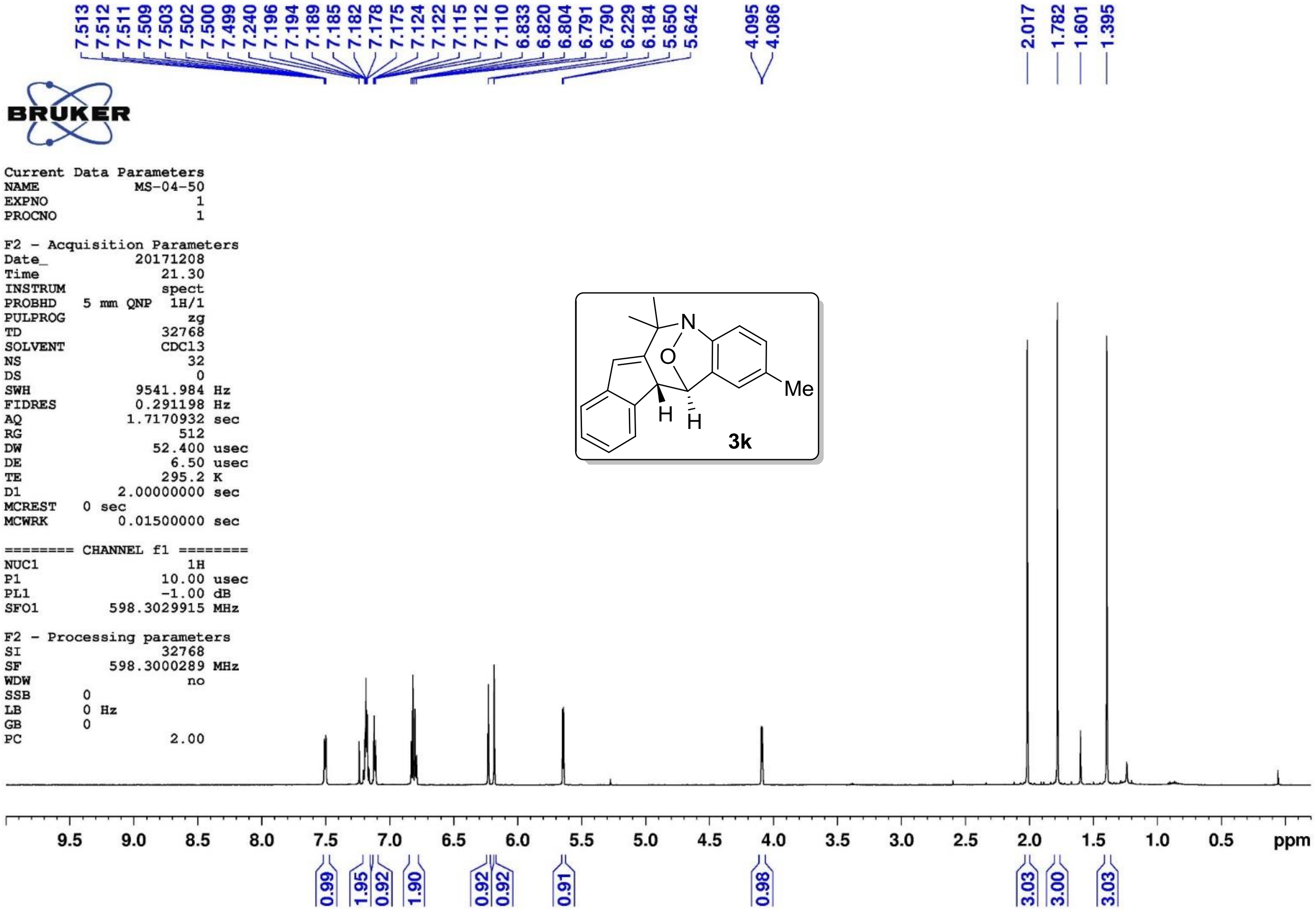

Current Data Parameters
NAME MS-04-50
EXPNO 1
PROCNO 1

F2 - Acquisition Parameters
Date_ 20171208
Time 21.30
INSTRUM spect
PROBHD 5 mm QNP 1H/1
PULPROG zg
TD 32768
SOLVENT CDCl3
NS 32
DS 0
SWH 9541.984 Hz
FIDRES 0.291198 Hz
AQ 1.7170932 sec
RG 512
DW 52.400 usec
DE 6.50 usec
TE 295.2 K
D1 2.00000000 sec
MCREST 0 sec
MCWRK 0.01500000 sec

======== CHANNEL f1 ========
NUC1 1H
P1 10.00 usec
PL1 -1.00 dB
SFO1 598.3029915 MHz

F2 - Processing parameters
SI 32768
SF 598.3000289 MHz
WDW no
SSB 0
LB 0 Hz
GB 0
PC 2.00

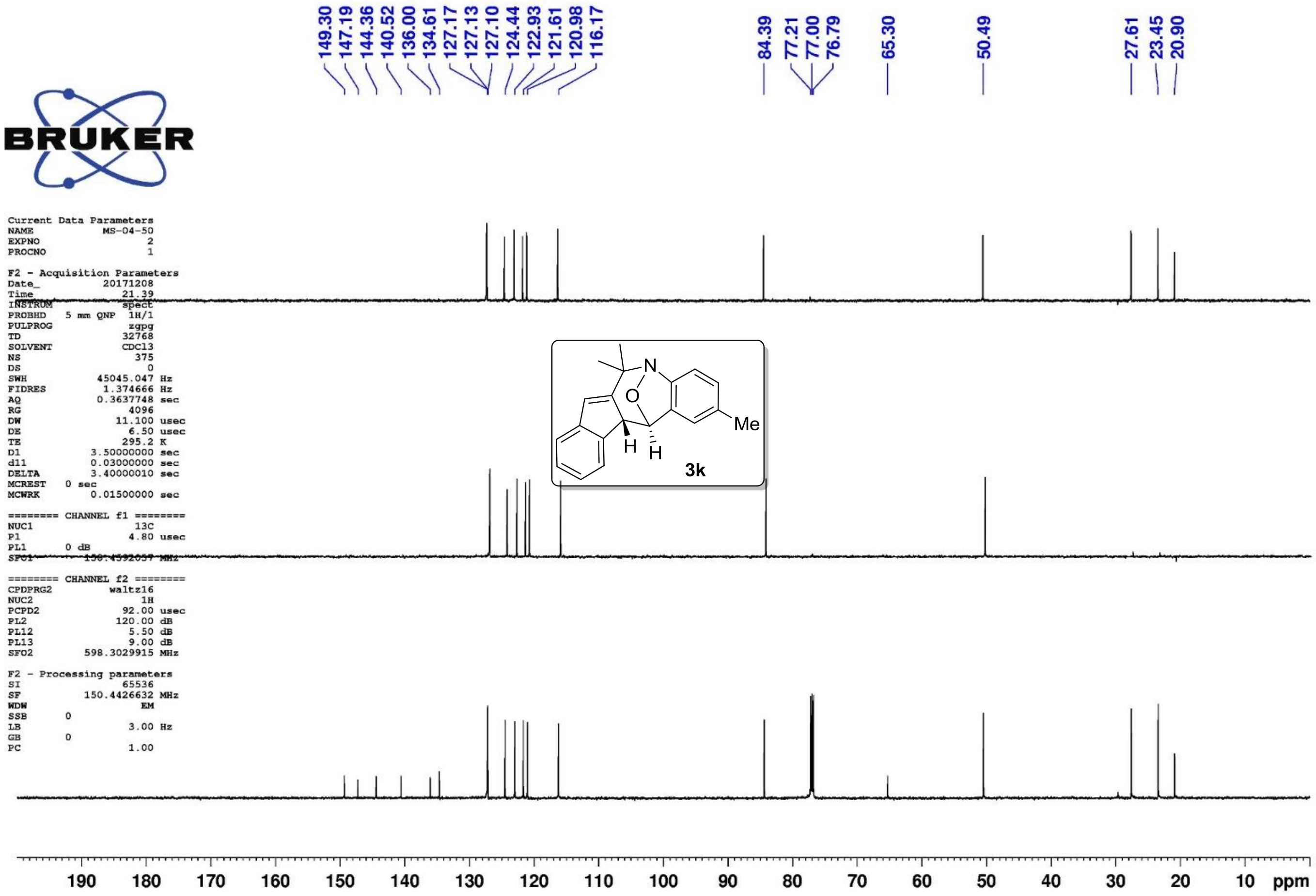
149.30
147.19
144.36
140.52
136.00
134.61
127.17
127.13
127.10
124.44
122.93
121.61
120.98
116.17
84.39
77.21
77.00
76.79
65.30
50.49
27.61
23.45
20.90
BRUKER
Current Data Parameters
NAME MS-04-50
EXPNO 2
PROCNO 1
F2 - Acquisition Parameters
Date_ 20171208
Time 21.39
INSTRUM spect
PROBHD 5 mm QNP 1H/1
PULPROG zgpg
TD 32768
SOLVENT CDCl3
NS 375
DS 0
SWH 45045.047 Hz
FIDRES 1.374666 Hz
AQ 0.3637748 sec
RG 4096
DW 11.100 usec
DE 6.50 usec
TE 295.2 K
D1 3.50000000 sec
d11 0.03000000 sec
DELTA 3.40000010 sec
MCREST 0 sec
MCWRK 0.01500000 sec
======== CHANNEL f1 ========
NUC1 13C
P1 4.80 usec
PL1 0 dB
SFO1 150.4592037 MHz
======== CHANNEL f2 ========
CPDPRG2 waltz16
NUC2 1H
PCPD2 92.00 usec
PL2 120.00 dB
PL12 5.50 dB
PL13 9.00 dB
SFO2 598.3029915 MHz
F2 - Processing parameters
SI 65536
SF 150.4426632 MHz
WDW EM
SSB 0
LB 3.00 Hz
GB 0
PC 1.00
N
O
Me
H
H
3k
190 180 170 160 150 140 130 120 110 100 90 80 70 60 50 40 30 20 10 ppm

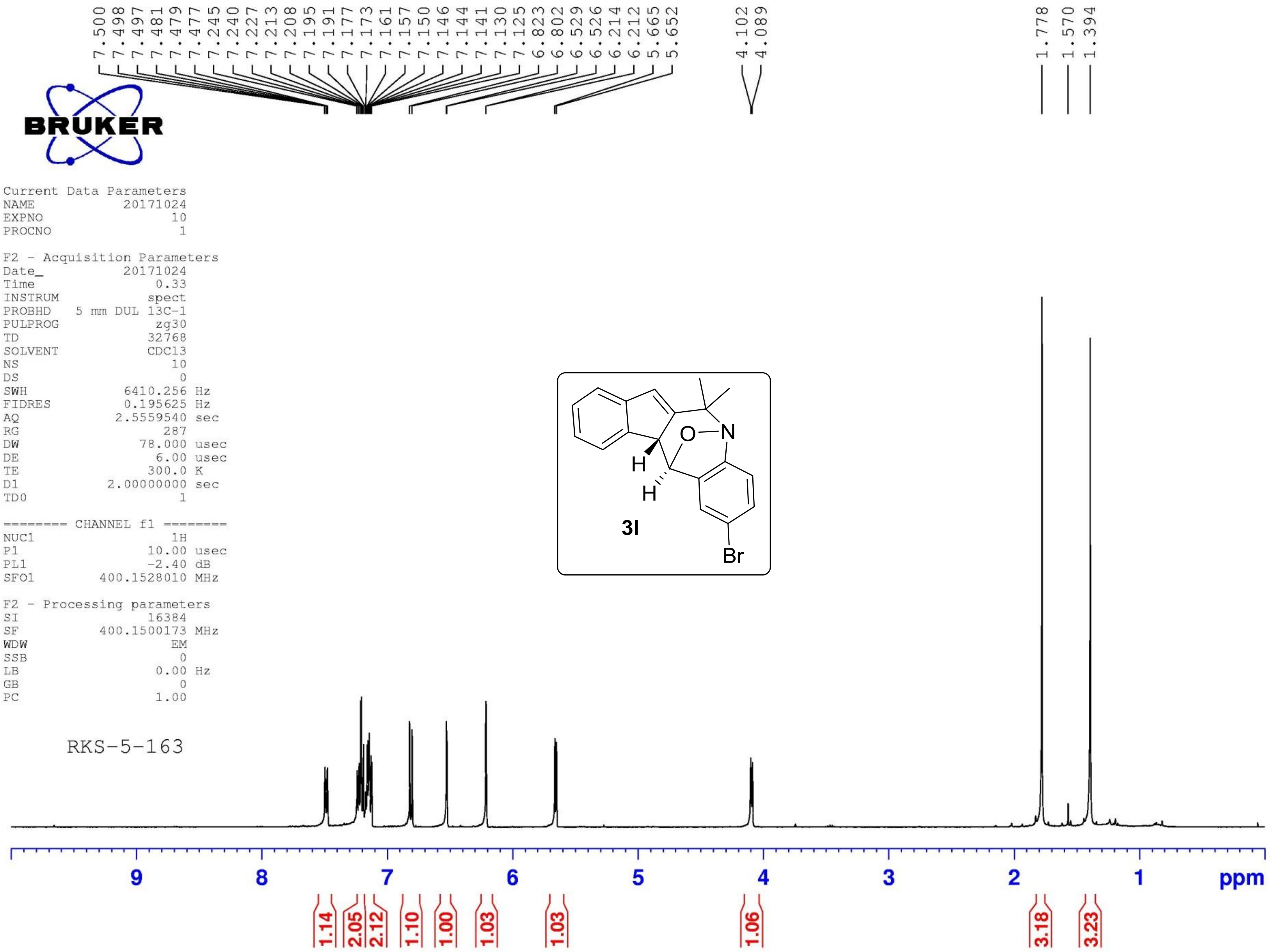
7.500
7.498
7.497
7.481
7.479
7.477
7.245
7.240
7.227
7.213
7.208
7.195
7.191
7.177
7.173
7.161
7.157
7.150
7.146
7.144
7.141
7.130
7.125
6.823
6.802
6.529
6.526
6.214
6.212
5.665
5.652
4.102
4.089
1.778
1.570
1.394
BRUKER
Current Data Parameters
NAME 20171024
EXPNO 10
PROCNO 1
F2 - Acquisition Parameters
Date_ 20171024
Time 0.33
INSTRUM spect
PROBHD 5 mm DUL 13C-1
PULPROG zg30
TD 32768
SOLVENT CDCl3
NS 10
DS 0
SWH 6410.256 Hz
FIDRES 0.195625 Hz
AQ 2.5559540 sec
RG 287
DW 78.000 usec
DE 6.00 usec
TE 300.0 K
D1 2.00000000 sec
TD0 1
======== CHANNEL f1 ========
NUC1 1H
P1 10.00 usec
PL1 -2.40 dB
SFO1 400.1528010 MHz
F2 - Processing parameters
SI 16384
SF 400.1500173 MHz
WDW EM
SSB 0
LB 0.00 Hz
GB 0
PC 1.00
RKS-5-163
O
N
H
H
3l
Br
9
8
7
6
5
4
3
2
1
ppm
1.14
2.05
2.12
1.10
1.00
1.03
1.03
1.06
3.18
3.23

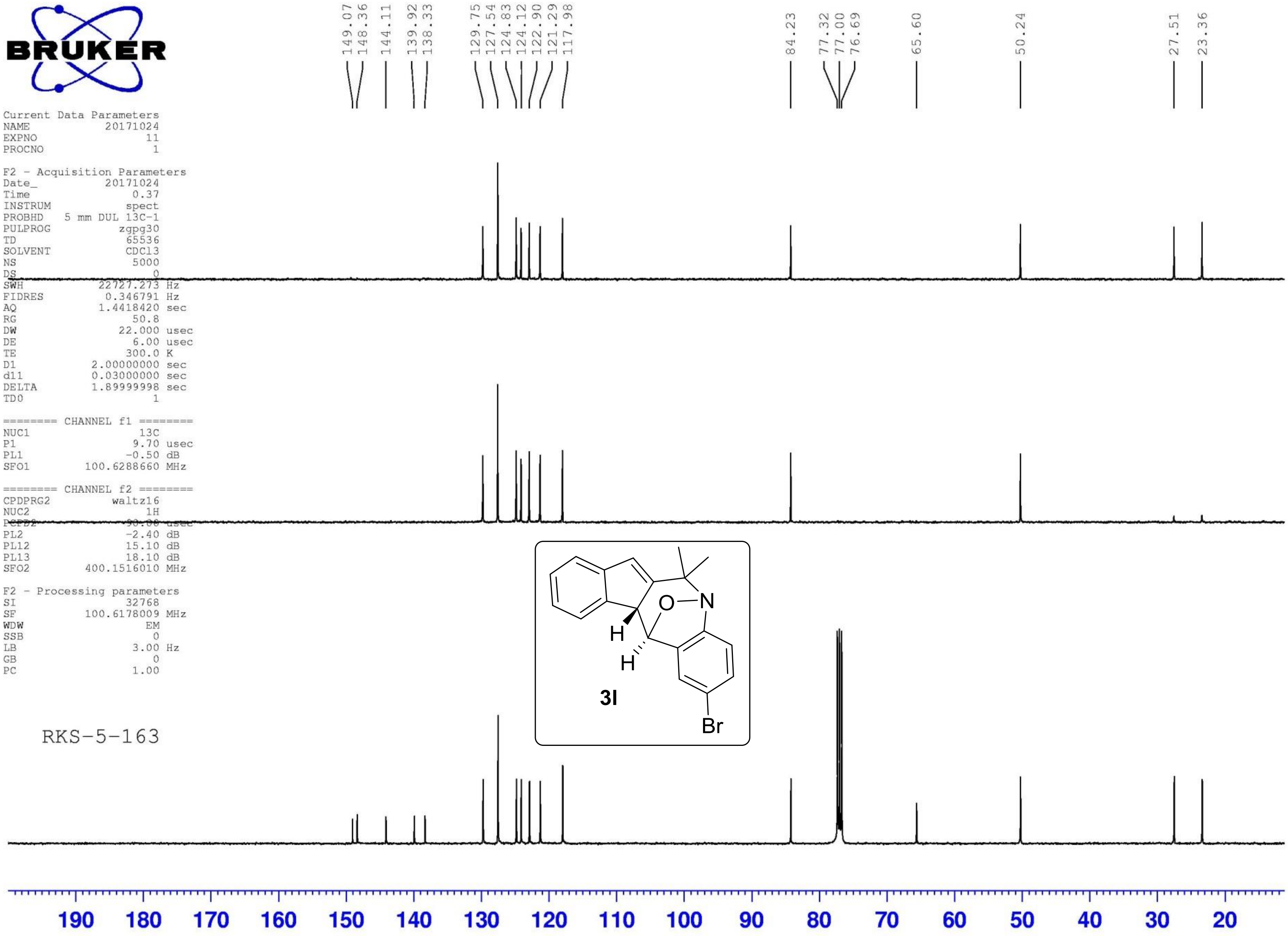

BRUKER
149.07
148.36
144.11
139.92
138.33
129.75
127.54
124.83
124.12
122.90
121.29
117.98
84.23
77.32
77.00
76.69
65.60
50.24
27.51
23.36
Current Data Parameters
NAME 20171024
EXPNO 11
PROCNO 1
F2 - Acquisition Parameters
Date_ 20171024
Time 0.37
INSTRUM spect
PROBHD 5 mm DUL 13C-1
PULPROG zgpg30
TD 65536
SOLVENT CDCl3
NS 5000
DS 0
SWH 22727.273 Hz
FIDRES 0.346791 Hz
AQ 1.4418420 sec
RG 50.8
DW 22.000 usec
DE 6.00 usec
TE 300.0 K
D1 2.00000000 sec
d11 0.03000000 sec
DELTA 1.89999998 sec
TD0 1
======== CHANNEL f1 ========
NUC1 13C
P1 9.70 usec
PL1 -0.50 dB
SFO1 100.6288660 MHz
======== CHANNEL f2 ========
CPDPRG2 waltz16
NUC2 1H
PCPD2 90.00 usec
PL2 -2.40 dB
PL12 15.10 dB
PL13 18.10 dB
SFO2 400.1516010 MHz
F2 - Processing parameters
SI 32768
SF 100.6178009 MHz
WDW EM
SSB 0
LB 3.00 Hz
GB 0
PC 1.00
RKS-5-163
O
N
H
H
3l
Br
190
180
170
160
150
140
130
120
110
100
90
80
70
60
50
40
30
20

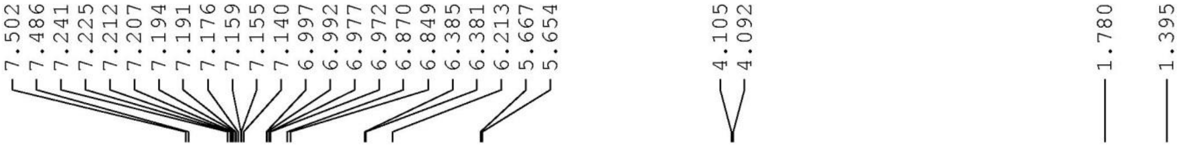

BRUKER

Current Data Parameters
NAME 20171031
EXPNO 1
PROCNO 1

F2 - Acquisition Parameters
Date_ 20171031
Time 0.28
INSTRUM spect
PROBHD 5 mm DUL 13C-1
PULPROG zg30
TD 32768
SOLVENT CDCl3
NS 11
DS 0
SWH 6410.256 Hz
FIDRES 0.195625 Hz
AQ 2.5559540 sec
RG 4
DW 78.000 usec
DE 6.00 usec
TE 300.0 K
D1 2.00000000 sec
TD0 1

======== CHANNEL f1 ========
NUC1 1H
P1 10.00 usec
PL1 -2.40 dB
SFO1 400.1528010 MHz

F2 - Processing parameters
SI 16384
SF 400.1500169 MHz
WDW EM
SSB 0
LB 0.00 Hz
GB 0
PC 1.00

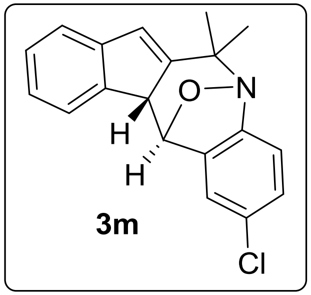

3m

RKS-5-162

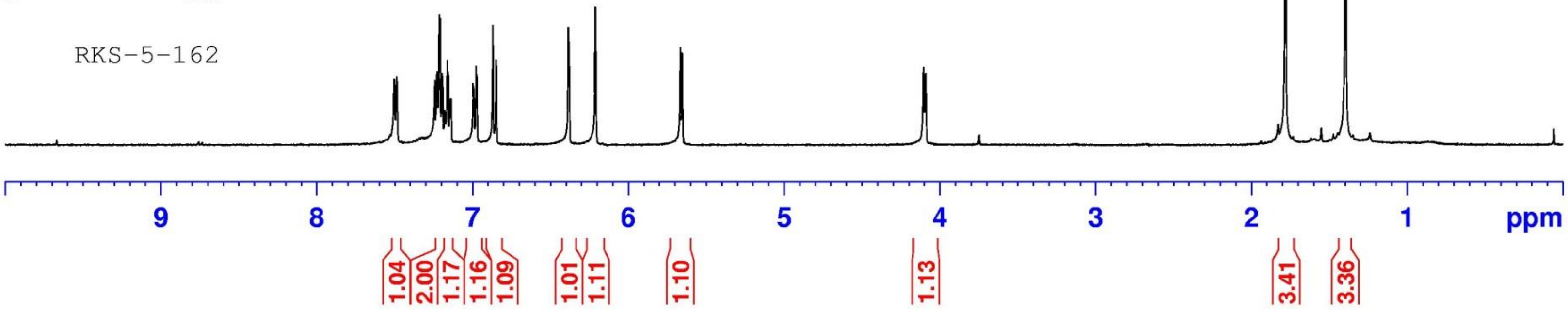

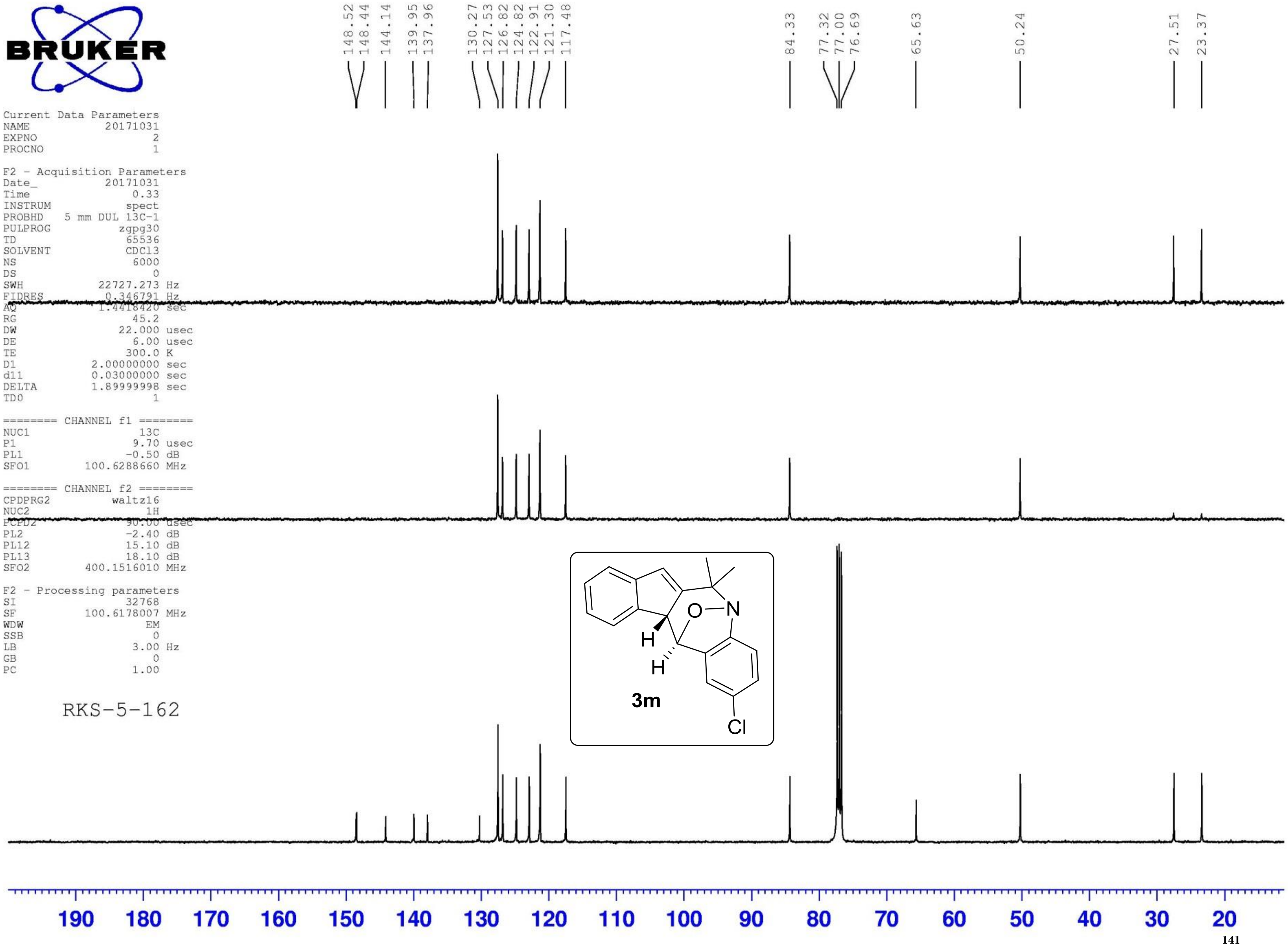
BRUKER
Current Data Parameters
NAME 20171031
EXPNO 2
PROCNO 1
F2 - Acquisition Parameters
Date_ 20171031
Time 0.33
INSTRUM spect
PROBHD 5 mm DUL 13C-1
PULPROG zgpg30
TD 65536
SOLVENT CDCl3
NS 6000
DS 0
SWH 22727.273 Hz
FIDRES 0.346791 Hz
AQ 1.4418420 sec
RG 45.2
DW 22.000 usec
DE 6.00 usec
TE 300.0 K
D1 2.00000000 sec
d11 0.03000000 sec
DELTA 1.89999998 sec
TD0 1
======== CHANNEL f1 ========
NUC1 13C
P1 9.70 usec
PL1 -0.50 dB
SFO1 100.6288660 MHz
======== CHANNEL f2 ========
CPDPRG2 waltz16
NUC2 1H
PCPD2 90.00 usec
PL2 -2.40 dB
PL12 15.10 dB
PL13 18.10 dB
SFO2 400.1516010 MHz
F2 - Processing parameters
SI 32768
SF 100.6178007 MHz
WDW EM
SSB 0
LB 3.00 Hz
GB 0
PC 1.00
RKS-5-162
148.52
148.44
144.14
139.95
137.96
130.27
127.53
126.82
124.82
122.91
121.30
117.48
84.33
77.32
77.00
76.69
65.63
50.24
27.51
23.37
O
N
H
H
3m
Cl
190
180
170
160
150
140
130
120
110
100
90
80
70
60
50
40
30
20

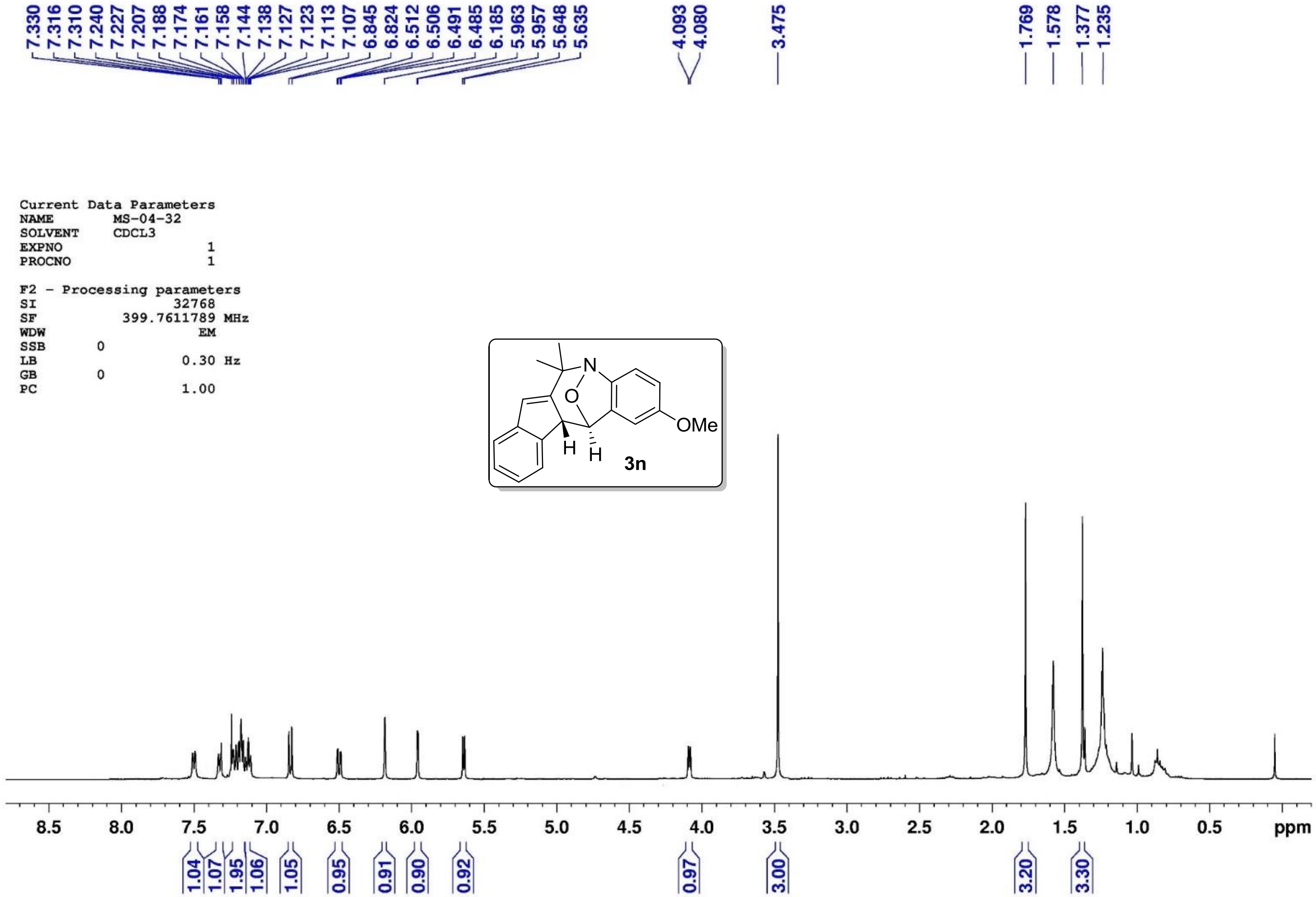
7.330 7.316 7.310 7.240 7.227 7.207 7.188 7.174 7.161 7.158 7.144 7.138 7.127 7.123 7.113 7.107 6.845 6.824 6.512 6.506 6.491 6.485 6.185 5.963 5.957 5.648 5.635
4.093 4.080
3.475
1.769 1.578 1.377 1.235
Current Data Parameters
NAME MS-04-32
SOLVENT CDCL3
EXPNO 1
PROCNO 1
F2 - Processing parameters
SI 32768
SF 399.7611789 MHz
WDW EM
SSB 0
LB 0.30 Hz
GB 0
PC 1.00
N
O
OMe
H
H
3n
8.5 8.0 7.5 7.0 6.5 6.0 5.5 5.0 4.5 4.0 3.5 3.0 2.5 2.0 1.5 1.0 0.5 ppm
1.04 1.07 1.95 1.06 1.05 0.95 0.91 0.90 0.92 0.97 3.00 3.20 3.30

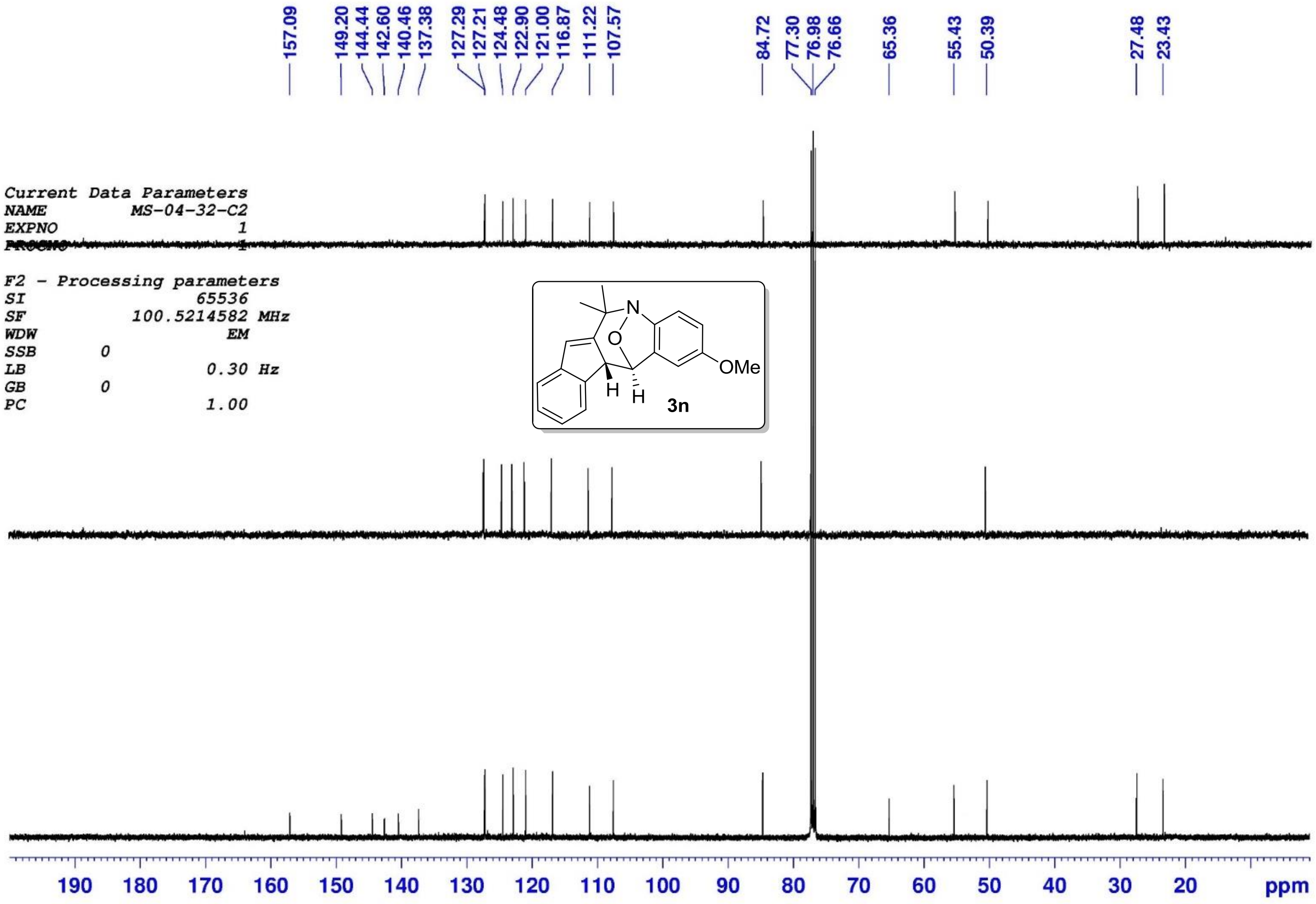
157.09
149.20
144.44
142.60
140.46
137.38
127.29
127.21
124.48
122.90
121.00
116.87
111.22
107.57
84.72
77.30
76.98
76.66
65.36
55.43
50.39
27.48
23.43
Current Data Parameters
NAME MS-04-32-C2
EXPNO 1
PROCNO 1
F2 - Processing parameters
SI 65536
SF 100.5214582 MHz
WDW EM
SSB 0
LB 0.30 Hz
GB 0
PC 1.00
N
O
OMe
H
H
3n
190 180 170 160 150 140 130 120 110 100 90 80 70 60 50 40 30 20 ppm

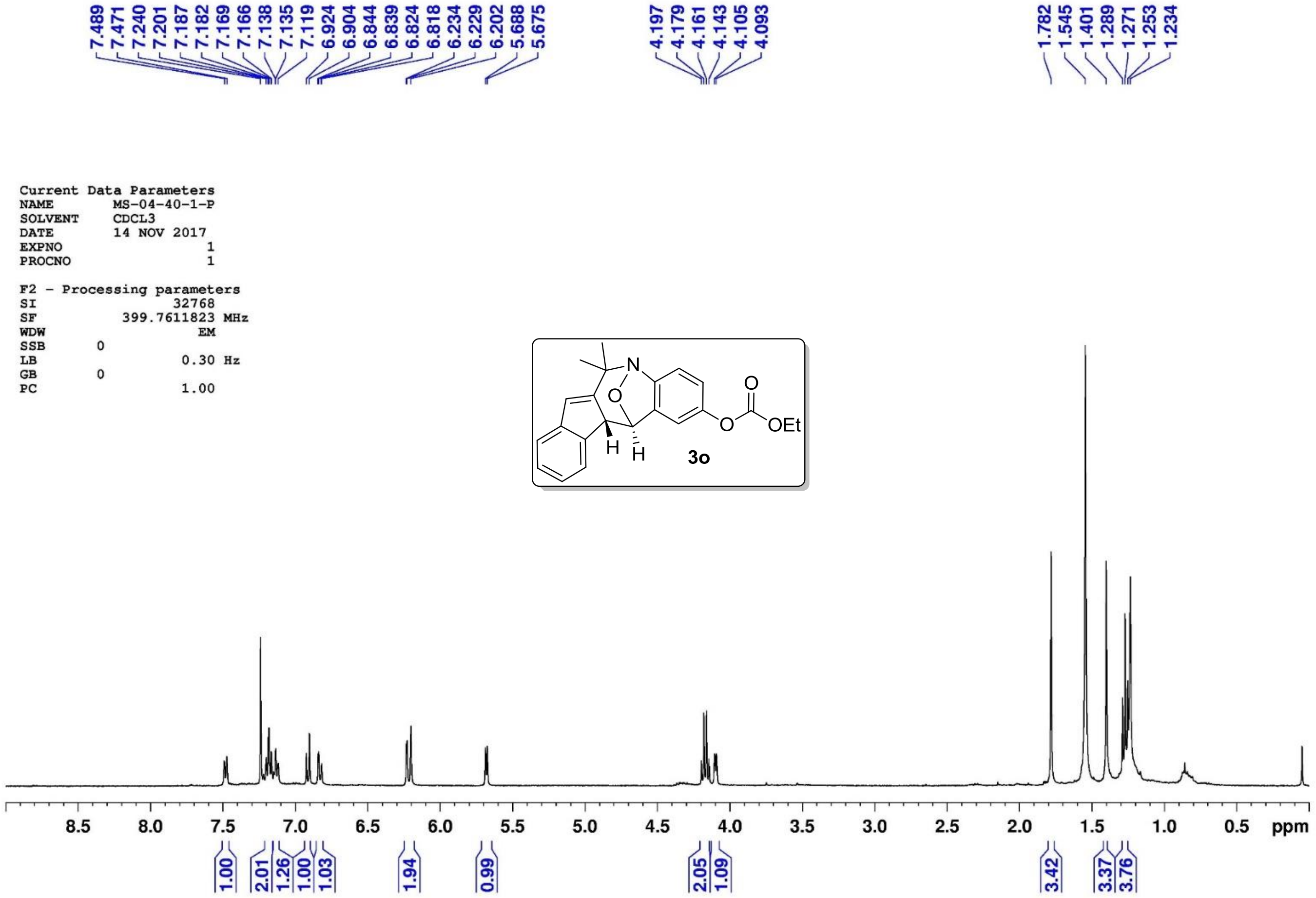
7.489 7.471 7.240 7.201 7.187 7.182 7.169 7.166 7.138 7.135 7.119 6.924 6.904 6.844 6.839 6.824 6.818 6.234 6.229 6.202 5.688 5.675
4.197 4.179 4.161 4.143 4.105 4.093
1.782 1.545 1.401 1.289 1.271 1.253 1.234
Current Data Parameters
NAME MS-04-40-1-P
SOLVENT CDCL3
DATE 14 NOV 2017
EXPNO 1
PROCNO 1
F2 - Processing parameters
SI 32768
SF 399.7611823 MHz
WDW EM
SSB 0
LB 0.30 Hz
GB 0
PC 1.00
N
O
O
O
OEt
H
H
3o
8.5 8.0 7.5 7.0 6.5 6.0 5.5 5.0 4.5 4.0 3.5 3.0 2.5 2.0 1.5 1.0 0.5 ppm
1.00 2.01 1.26 1.00 1.03 1.94 0.99 2.05 1.09 3.42 3.37 3.76

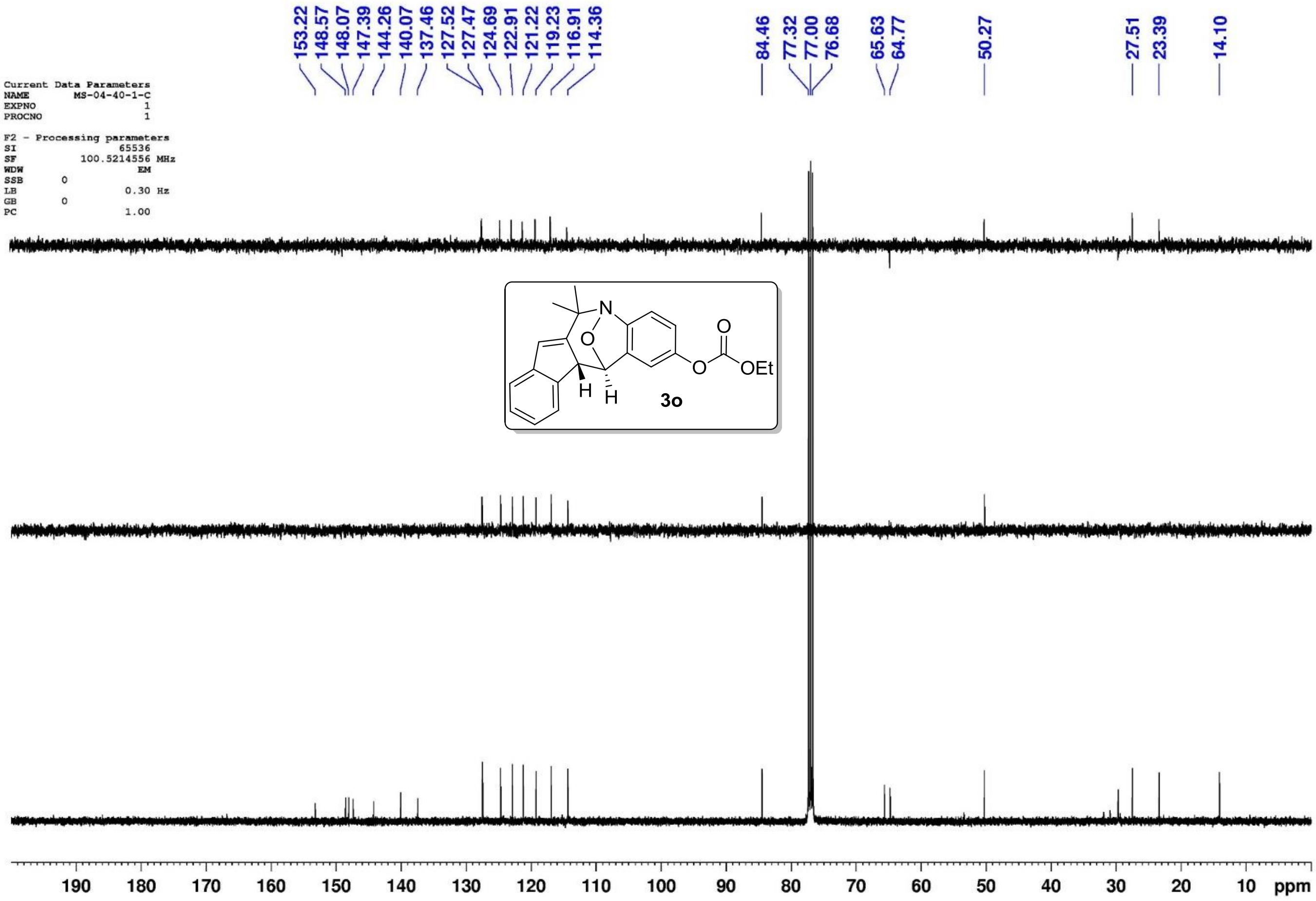
Current Data Parameters
NAME MS-04-40-1-C
EXPNO 1
PROCNO 1
F2 - Processing parameters
SI 65536
SF 100.5214556 MHz
WDW EM
SSB 0
LB 0.30 Hz
GB 0
PC 1.00
153.22
148.57
148.07
147.39
144.26
140.07
137.46
127.52
127.47
124.69
122.91
121.22
119.23
116.91
114.36
84.46
77.32
77.00
76.68
65.63
64.77
50.27
27.51
23.39
14.10
N
O
O
O
OEt
H
H
3o
190
180
170
160
150
140
130
120
110
100
90
80
70
60
50
40
30
20
10
ppm

Current Data Parameters
NAME VMS-04-033
EXPNO 1
PROCNO 1

F2 - Acquisition Parameters
Date_ 20171110
Time 5.43
INSTRUM spect
PROBHD 5 mm QNP 1H/1
PULPROG zg
TD 32768
SOLVENT CDCl3
NS 32
DS 0
SWH 10775.862 Hz
FIDRES 0.328853 Hz
AQ 1.5204852 sec
RG 512
DW 46.400 usec
DE 6.50 usec
TE 302.2 K
D1 2.00000000 sec
MCREST 0 sec
MCWRK 0.01500000 sec

======== CHANNEL f1 ========
NUC1 1H
P1 9.60 usec
PL1 1.00 dB
SFO1 598.4035904 MHz

F2 - Processing parameters
SI 32768
SF 598.4000252 MHz
WDW no
SSB 0
LB 0 Hz
GB 0
PC 1.00

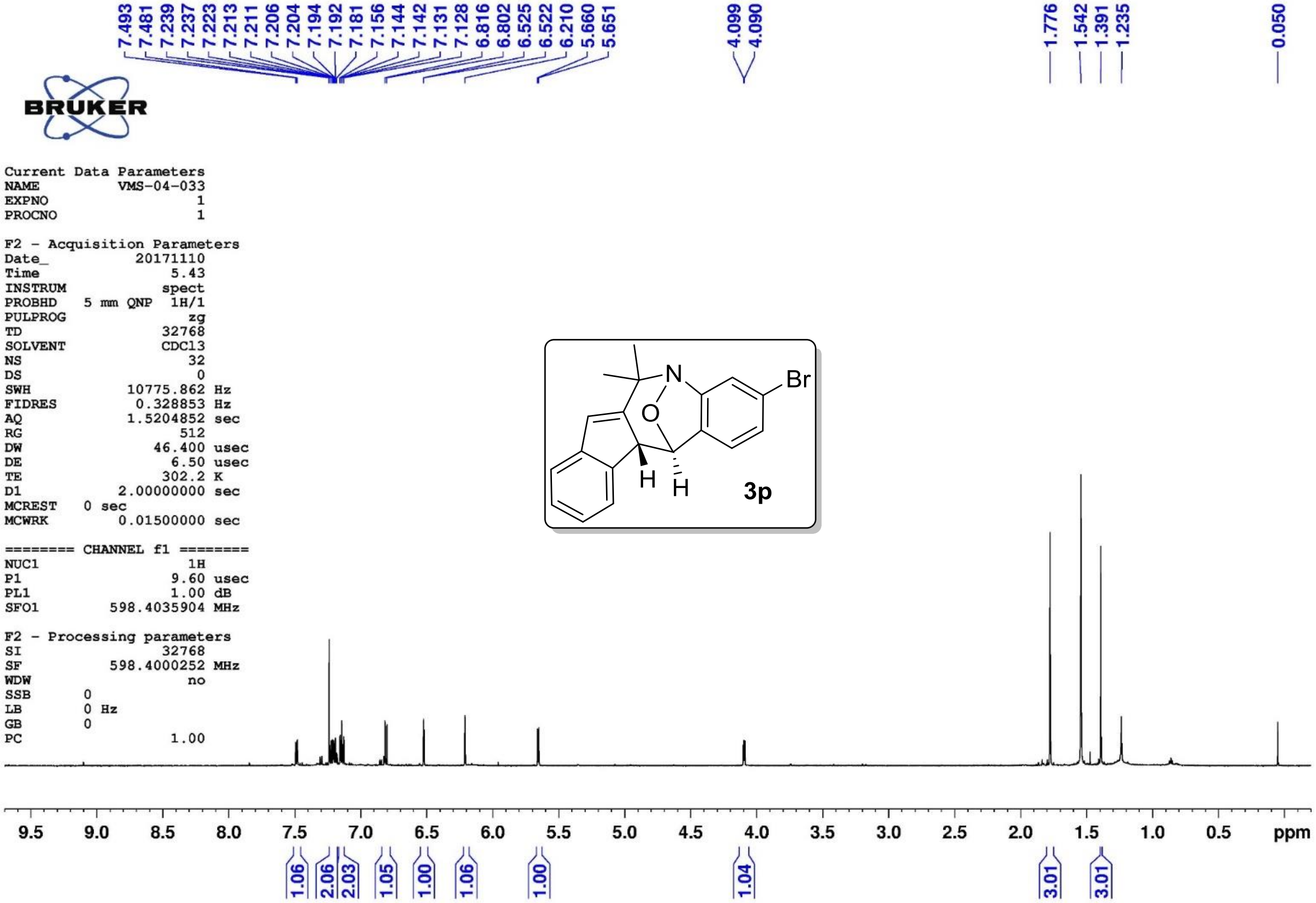

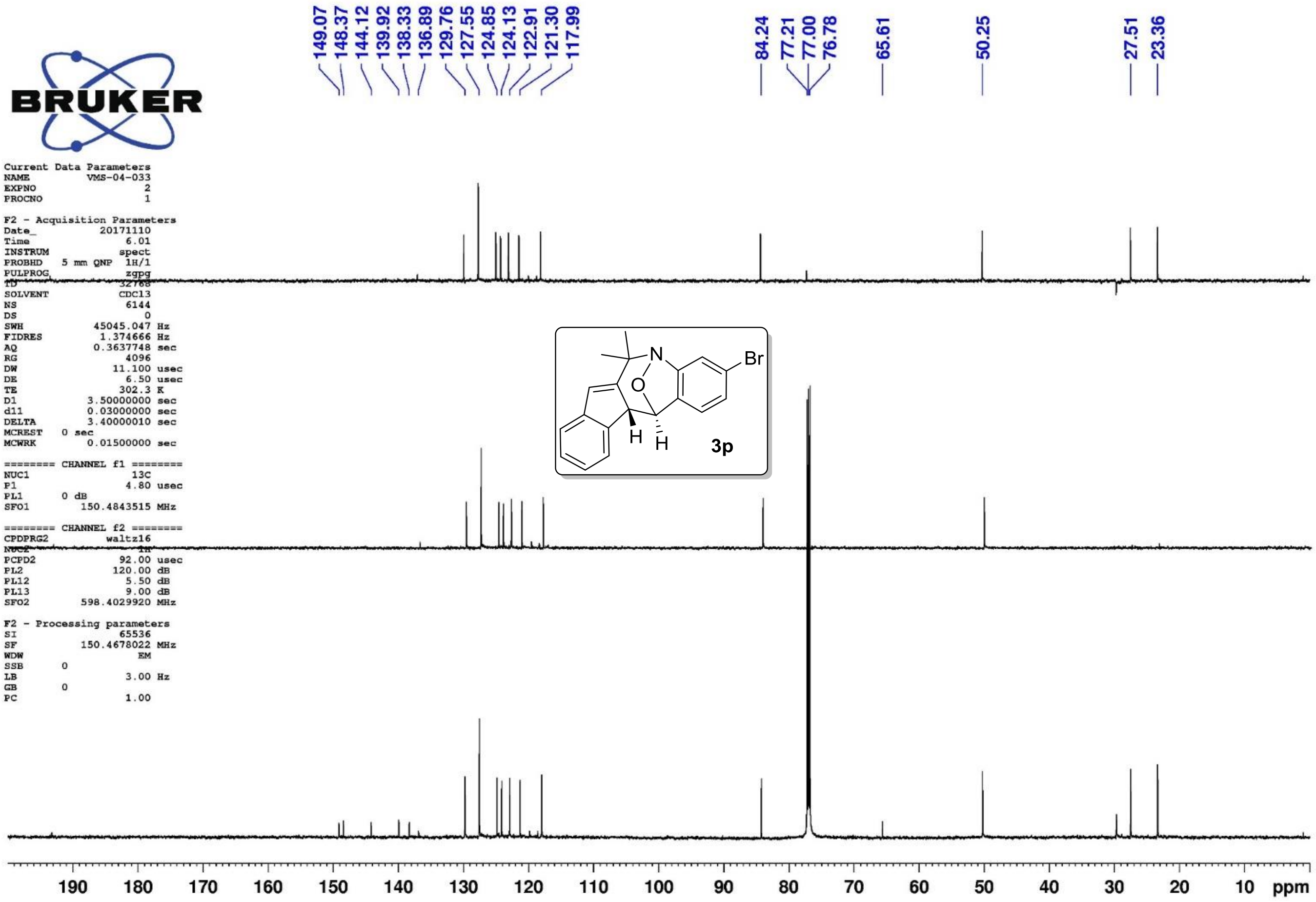
149.07
148.37
144.12
139.92
138.33
136.89
129.76
127.55
124.85
124.13
122.91
121.30
117.99
84.24
77.21
77.00
76.78
65.61
50.25
27.51
23.36
BRUKER
Current Data Parameters
NAME VMS-04-033
EXPNO 2
PROCNO 1
F2 - Acquisition Parameters
Date_ 20171110
Time 6.01
INSTRUM spect
PROBHD 5 mm QNP 1H/1
PULPROG zgpg
TD 32768
SOLVENT CDCl3
NS 6144
DS 0
SWH 45045.047 Hz
FIDRES 1.374666 Hz
AQ 0.3637748 sec
RG 4096
DW 11.100 usec
DE 6.50 usec
TE 302.3 K
D1 3.50000000 sec
d11 0.03000000 sec
DELTA 3.40000010 sec
MCREST 0 sec
MCWRK 0.01500000 sec
======== CHANNEL f1 ========
NUC1 13C
P1 4.80 usec
PL1 0 dB
SFO1 150.4843515 MHz
======== CHANNEL f2 ========
CPDPRG2 waltz16
NUC2 1H
PCPD2 92.00 usec
PL2 120.00 dB
PL12 5.50 dB
PL13 9.00 dB
SFO2 598.4029920 MHz
F2 - Processing parameters
SI 65536
SF 150.4678022 MHz
WDW EM
SSB 0
LB 3.00 Hz
GB 0
PC 1.00
N
O
Br
H
H
3p
190
180
170
160
150
140
130
120
110
100
90
80
70
60
50
40
30
20
10
ppm

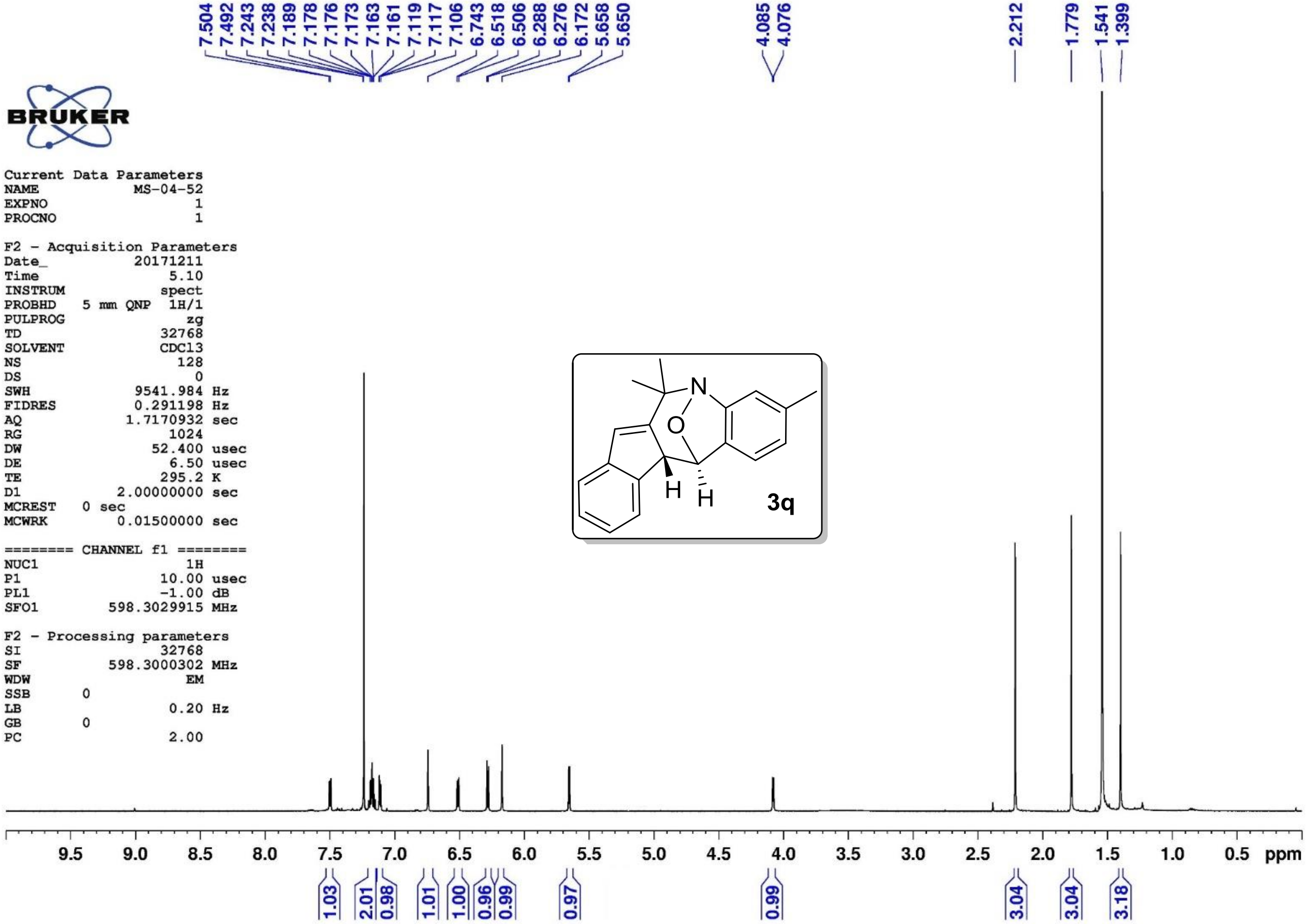
7.504
7.492
7.243
7.238
7.189
7.178
7.176
7.173
7.163
7.161
7.119
7.117
7.106
6.743
6.518
6.506
6.288
6.276
6.172
5.658
5.650
4.085
4.076
2.212
1.779
1.541
1.399
BRUKER
Current Data Parameters
NAME MS-04-52
EXPNO 1
PROCNO 1
F2 - Acquisition Parameters
Date_ 20171211
Time 5.10
INSTRUM spect
PROBHD 5 mm QNP 1H/1
PULPROG zg
TD 32768
SOLVENT CDCl3
NS 128
DS 0
SWH 9541.984 Hz
FIDRES 0.291198 Hz
AQ 1.7170932 sec
RG 1024
DW 52.400 usec
DE 6.50 usec
TE 295.2 K
D1 2.00000000 sec
MCREST 0 sec
MCWRK 0.01500000 sec
======== CHANNEL f1 ========
NUC1 1H
P1 10.00 usec
PL1 -1.00 dB
SFO1 598.3029915 MHz
F2 - Processing parameters
SI 32768
SF 598.3000302 MHz
WDW EM
SSB 0
LB 0.20 Hz
GB 0
PC 2.00
N
O
H
H
3q
9.5
9.0
8.5
8.0
7.5
7.0
6.5
6.0
5.5
5.0
4.5
4.0
3.5
3.0
2.5
2.0
1.5
1.0
0.5
ppm
1.03
2.01
0.98
1.01
1.00
0.96
0.99
0.97
0.99
3.04
3.04
3.18

Current Data Parameters
NAME MS-04-52-C
EXPNO 1
PROCNO 1

F2 - Processing parameters
SI 65536
SF 100.5214566 MHz
WDW EM
SSB 0
LB 0.30 Hz
GB 0
PC 1.00

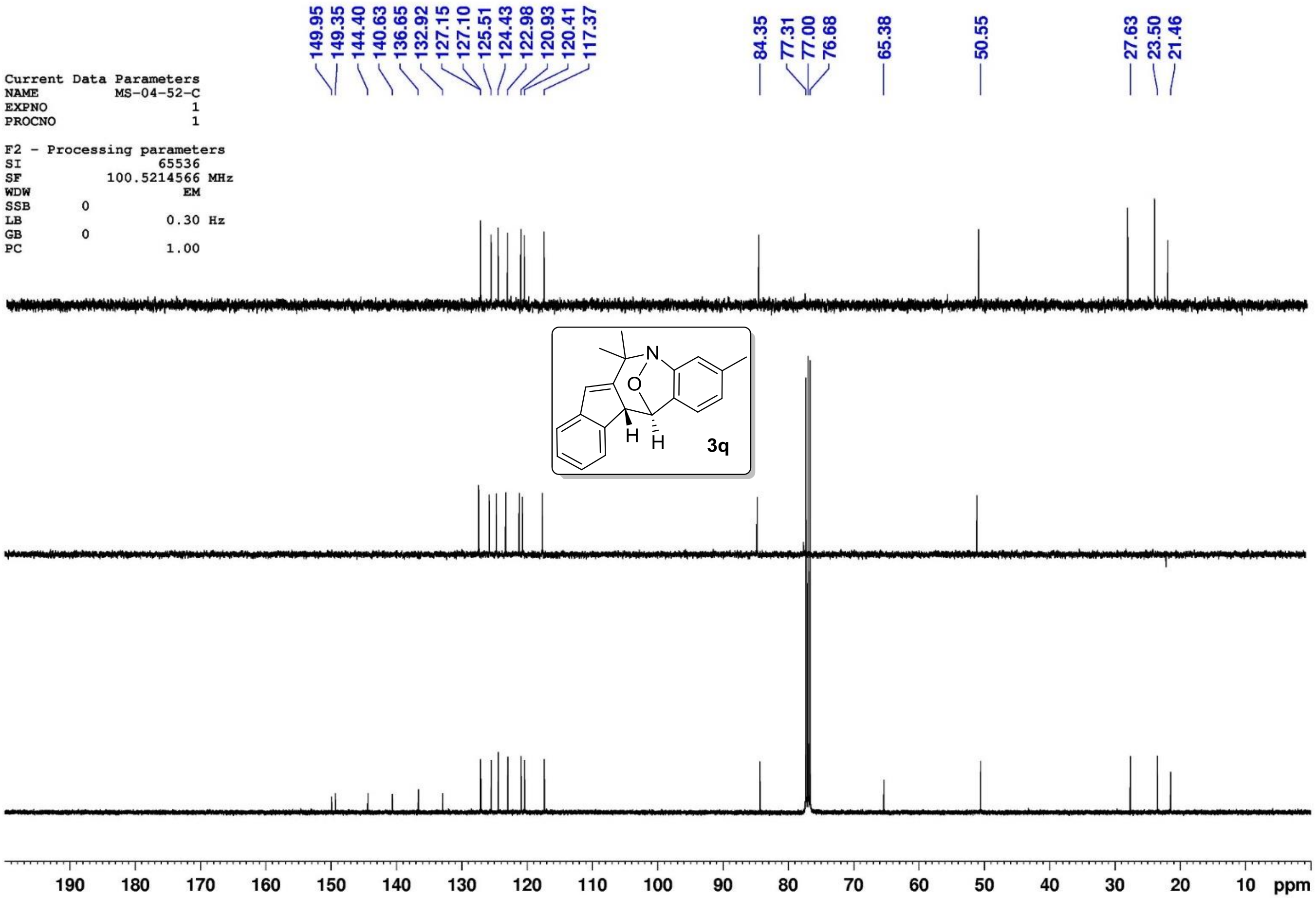

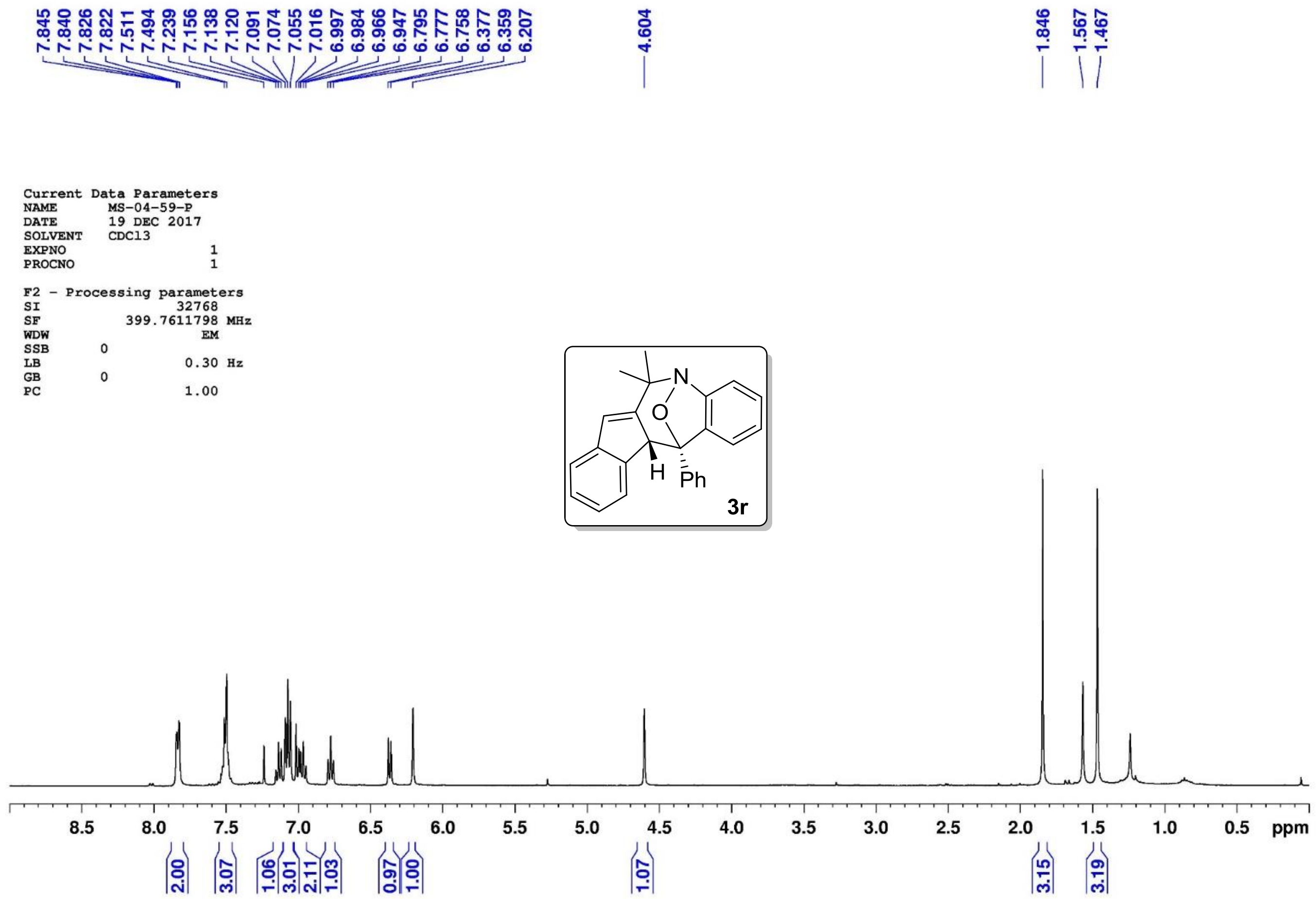
7.845
7.840
7.826
7.822
7.511
7.494
7.239
7.156
7.138
7.120
7.091
7.074
7.055
7.016
6.997
6.984
6.966
6.947
6.795
6.777
6.758
6.377
6.359
6.207
4.604
1.846
1.567
1.467
Current Data Parameters
NAME MS-04-59-P
DATE 19 DEC 2017
SOLVENT CDCl3
EXPNO 1
PROCNO 1
F2 - Processing parameters
SI 32768
SF 399.7611798 MHz
WDW EM
SSB 0
LB 0.30 Hz
GB 0
PC 1.00
N
O
H
Ph
3r
8.5
8.0
7.5
7.0
6.5
6.0
5.5
5.0
4.5
4.0
3.5
3.0
2.5
2.0
1.5
1.0
0.5
ppm
2.00
3.07
1.06
3.01
2.11
1.03
0.97
1.00
1.07
3.15
3.19

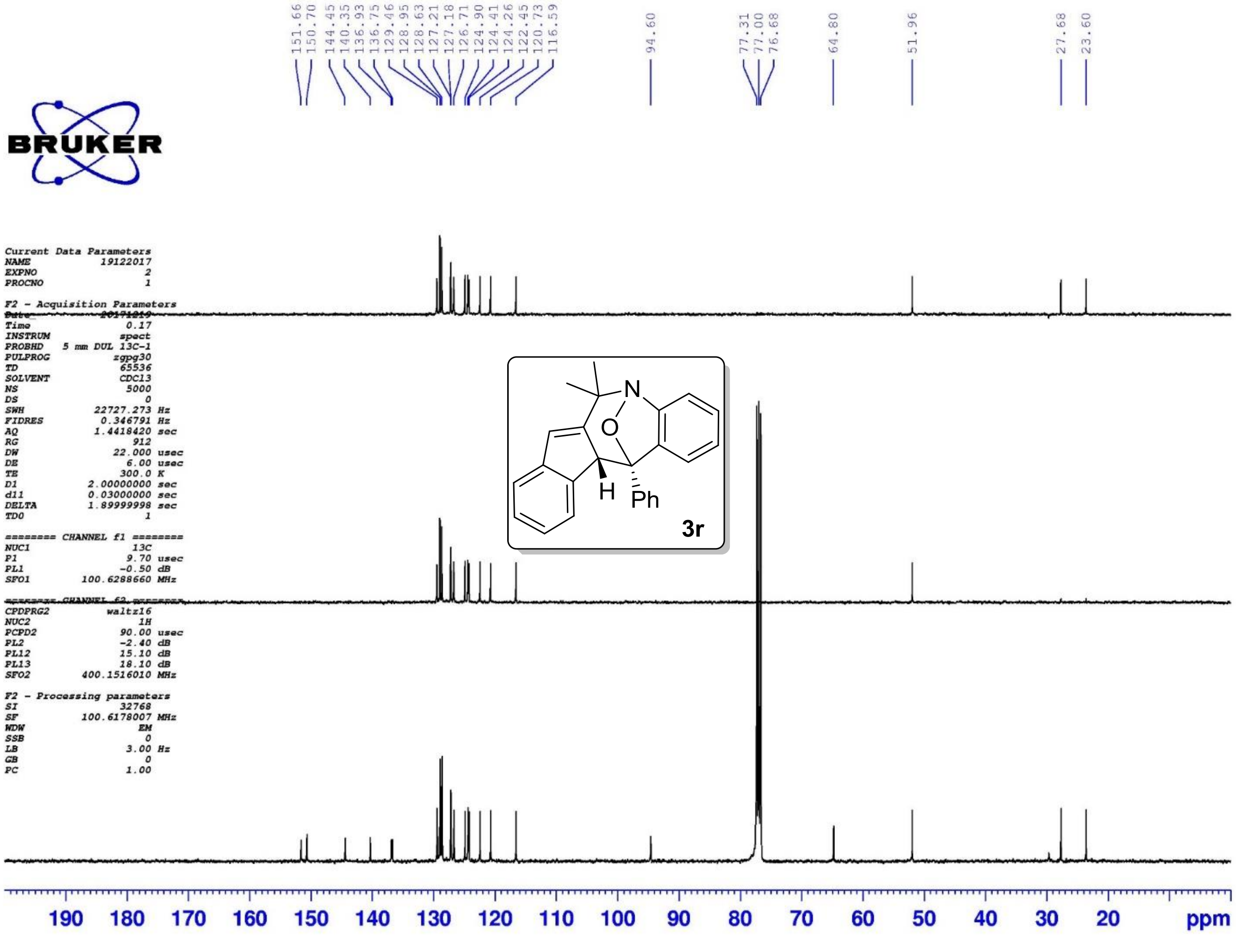
151.66
150.70
144.45
140.35
136.93
136.75
129.46
128.95
128.63
127.21
127.18
126.71
124.90
124.41
124.26
122.45
120.73
116.59
94.60
77.31
77.00
76.68
64.80
51.96
27.68
23.60
BRUKER
Current Data Parameters
NAME 19122017
EXPNO 2
PROCNO 1
F2 - Acquisition Parameters
Date_ 20171219
Time 0.17
INSTRUM spect
PROBHD 5 mm DUL 13C-1
PULPROG zgpg30
TD 65536
SOLVENT CDCl3
NS 5000
DS 0
SWH 22727.273 Hz
FIDRES 0.346791 Hz
AQ 1.4418420 sec
RG 912
DW 22.000 usec
DE 6.00 usec
TE 300.0 K
D1 2.00000000 sec
d11 0.03000000 sec
DELTA 1.89999998 sec
TD0 1
======== CHANNEL f1 ========
NUC1 13C
P1 9.70 usec
PL1 -0.50 dB
SFO1 100.6288660 MHz
======== CHANNEL f2 ========
CPDPRG2 waltz16
NUC2 1H
PCPD2 90.00 usec
PL2 -2.40 dB
PL12 15.10 dB
PL13 18.10 dB
SFO2 400.1516010 MHz
F2 - Processing parameters
SI 32768
SF 100.6178007 MHz
WDW EM
SSB 0
LB 3.00 Hz
GB 0
PC 1.00
N
O
H
Ph
3r
190
180
170
160
150
140
130
120
110
100
90
80
70
60
50
40
30
20
ppm

Current Data Parameters
NAME MS-04-30-P
SOLVENT CDCl3
DATE 02 NOV 2017
EXPNO 1
PROCNO 1

F2 - Processing parameters
SI 32768
SF 399.7611783 MHz
WDW EM
SSB 0
LB 0.30 Hz
GB 0
PC 1.00

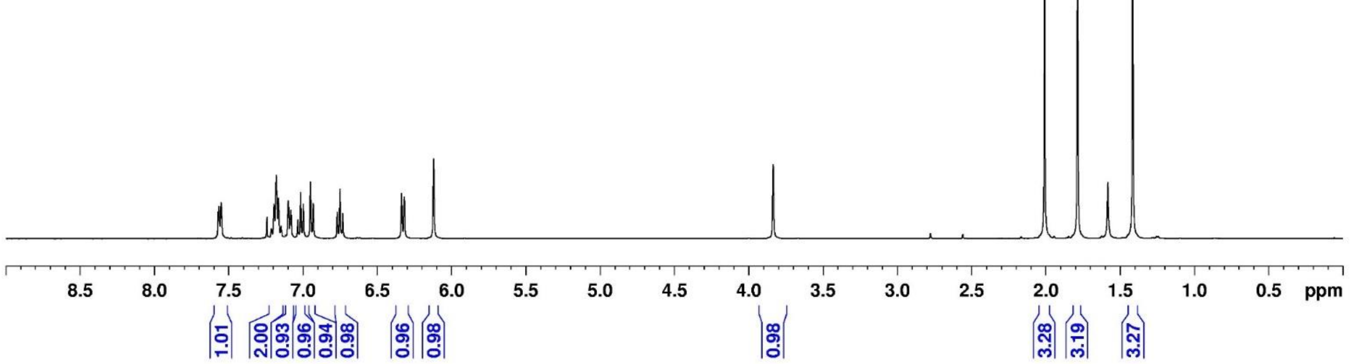

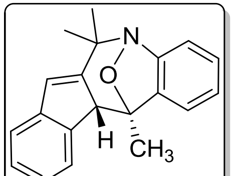

3s

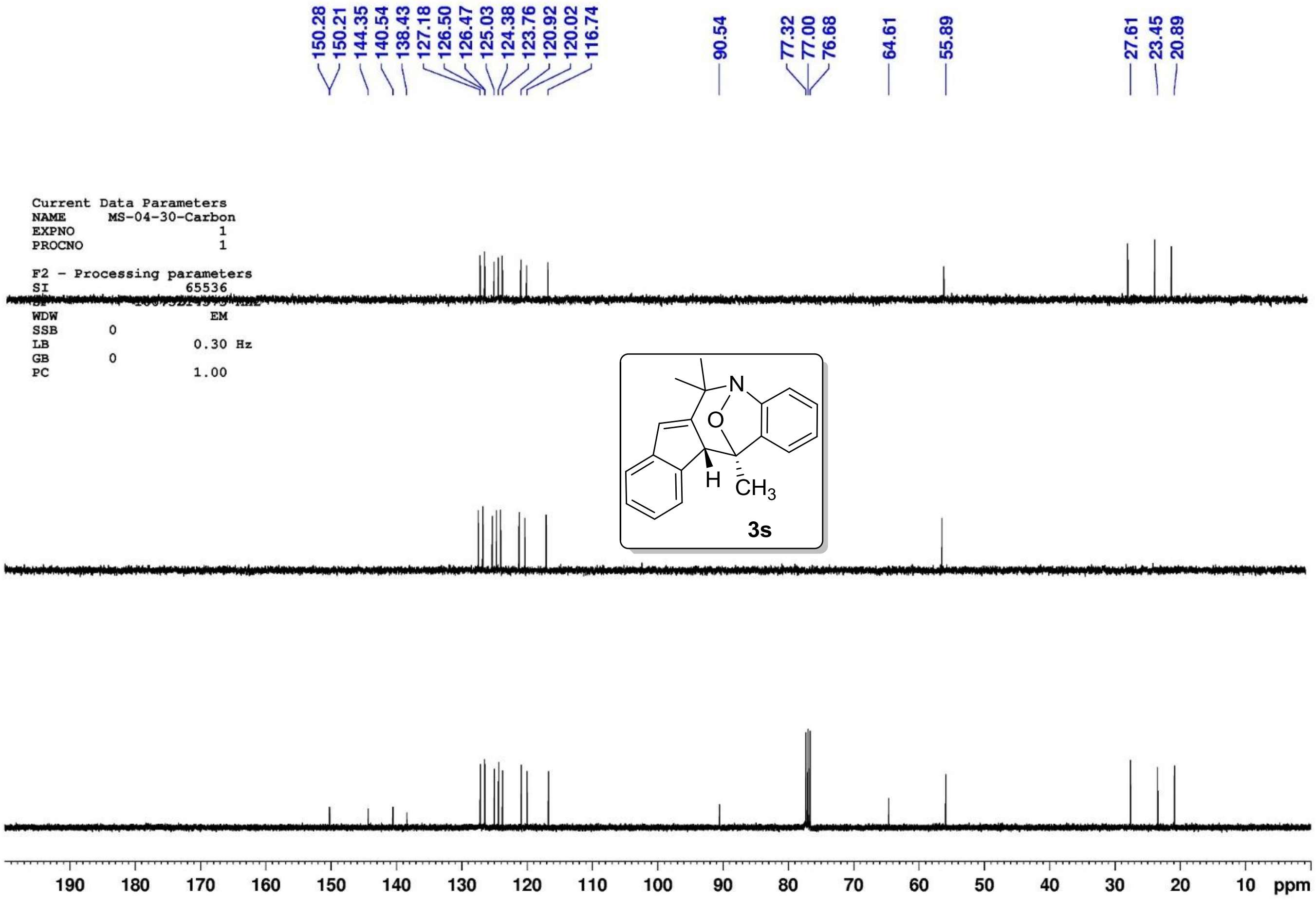
150.28
150.21
144.35
140.54
138.43
127.18
126.50
126.47
125.03
124.38
123.76
120.92
120.02
116.74
90.54
77.32
77.00
76.68
64.61
55.89
27.61
23.45
20.89
Current Data Parameters
NAME MS-04-30-Carbon
EXPNO 1
PROCNO 1
F2 - Processing parameters
SI 65536
WDW EM
SSB 0
LB 0.30 Hz
GB 0
PC 1.00
N
O
H
CH_3
3s
190 180 170 160 150 140 130 120 110 100 90 80 70 60 50 40 30 20 10 ppm

Current Data Parameters
NAME RKS-5-141-P-HT
EXPNO 1
PROCNO 1

F2 - Acquisition Parameters
Date_ 20170921
Time 14.53
INSTRUM spect
PROBHD 5 mm QNP 1H/1
PULPROG zg
TD 32768
SOLVENT CDCl3
NS 32
DS 0
SWH 9541.984 Hz
FIDRES 0.291198 Hz
AQ 1.7170932 sec
RG 256
DW 52.400 usec
DE 6.50 usec
TE 326.8 K
D1 2.00000000 sec
MCREST 0.00000000 sec
MCWRK 0.01500000 sec

======== CHANNEL f1 ========
NUC1 1H
P1 15.00 usec
PL1 -1.00 dB
SFO1 598.4035904 MHz

F2 - Processing parameters
SI 32768
SF 598.4000247 MHz
WDW no
SSB 0
LB 0.00 Hz
GB 0
PC 1.00

1D NMR plot parameters
CX 20.00 cm
CY 10.00 cm
F1P 11.000 ppm
F1 6582.40 Hz
F2P -0.500 ppm
F2 -299.20 Hz
PPMCM 0.57500 ppm/cm
HZCM 344.08002 Hz/cm

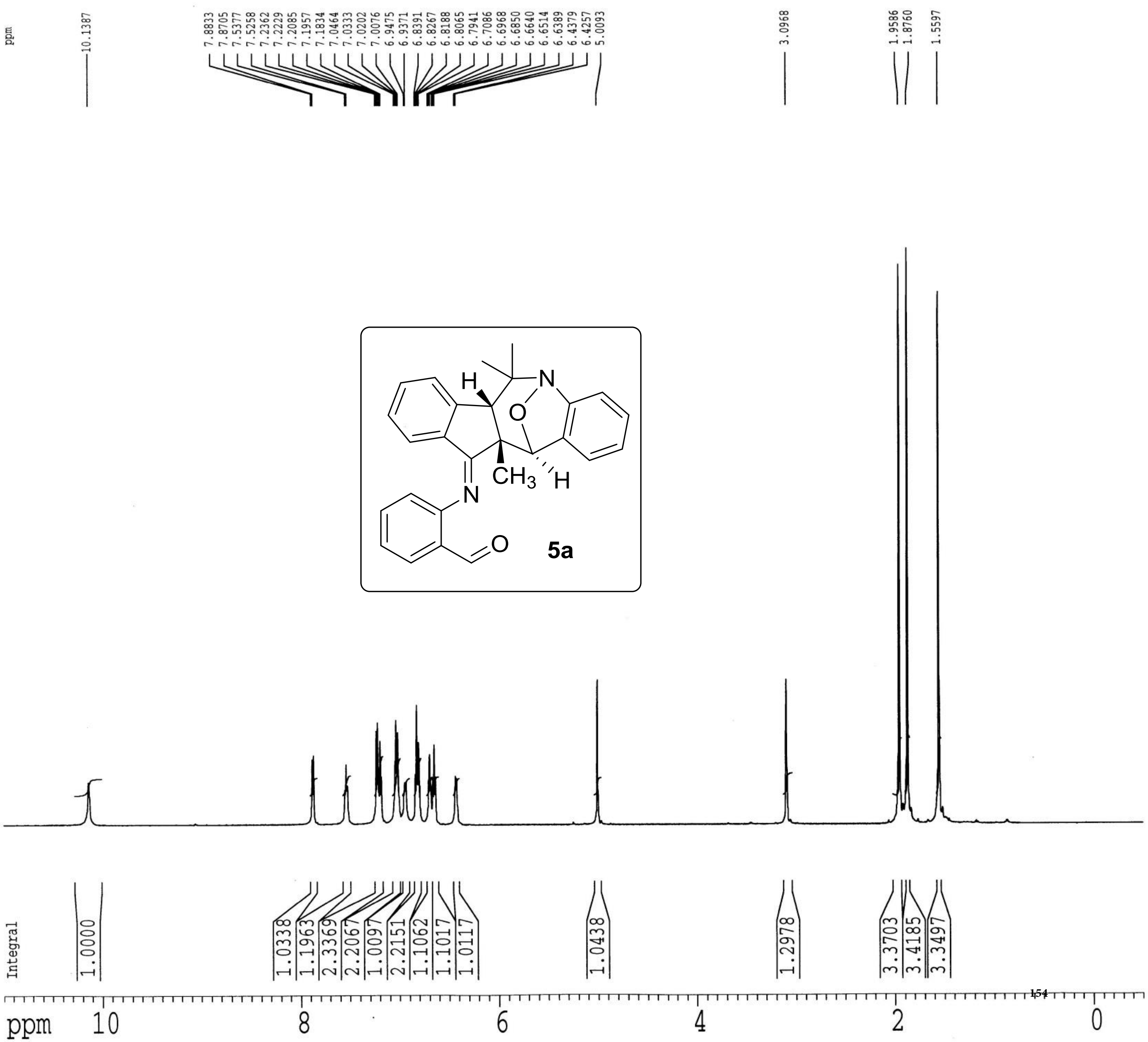

Current Data Parameters
NAME RKS-5-141-P-HT
EXPNO 2
PROCNO 1

F2 - Acquisition Parameters
Date_ 20170921
Time 14.31
INSTRUM spect
PROBHD 5 mm QNP 1H/1
PULPROG zgpg
TD 32768
SOLVENT CDCl3
NS 200
DS 0
SWH 45045.047 Hz
FIDRES 1.374666 Hz
AQ 0.3637748 sec
RG 4096
DW 11.100 usec
DE 6.50 usec
TE 323.9 K
D1 3.50000000 sec
d11 0.03000000 sec
DELTA 3.40000010 sec
MCREST 0.00000000 sec
MCWRK 0.01500000 sec

======== CHANNEL f1 ========
NUC1 13C
P1 4.80 usec
PL1 0.00 dB
SFO1 150.4828468 MHz

======== CHANNEL f2 ========
CPDPRG2 waltz16
NUC2 1H
PCPD2 92.00 usec
PL2 120.00 dB
PL12 5.50 dB
PL13 9.00 dB
SFO2 598.4029920 MHz

F2 - Processing parameters
SI 65536
SF 150.4677856 MHz
WDW EM
SSB 0
LB 3.00 Hz
GB 0
PC 1.00

1D NMR plot parameters
CX 20.00 cm
CY 3.00 cm
F1P 200.000 ppm
F1 30093.56 Hz
F2P 0.000 ppm
F2 0.00 Hz
PPMCM 10.00000 ppm/cm
HZCM 1504.67786 Hz/cm

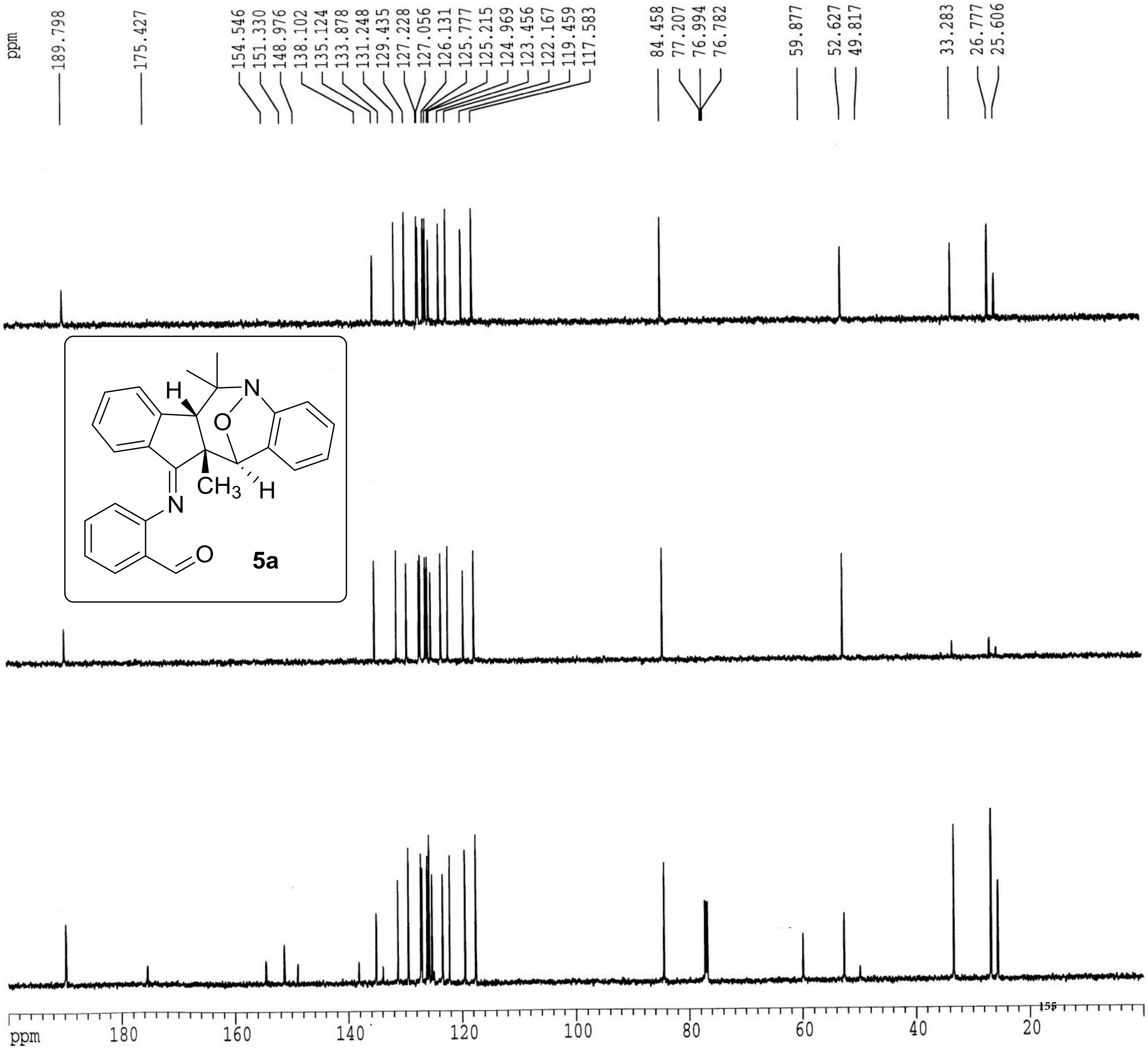

Current Data Parameters
NAME RKS-5-235-P2-HT
EXPNO 1
PROCNO 1

F2 - Acquisition Parameters
Date_ 20170915
Time 12.06
INSTRUM spect
PROBHD 5 mm QNP 1H/1
PULPROG zg
TD 32768
SOLVENT CDCl3
NS 32
DS 0
SWH 8389.262 Hz
FIDRES 0.256020 Hz
AQ 1.9530228 sec
RG 256
DW 59.600 usec
DE 6.50 usec
TE 323.4 K
D1 2.00000000 sec
MCREST 0.00000000 sec
MCWRK 0.01500000 sec

======== CHANNEL f1 ========
NUC1 1H
P1 15.00 usec
PL1 -1.00 dB
SFO1 598.4032912 MHz

F2 - Processing parameters
SI 32768
SF 598.4000247 MHz
WDW no
SSB 0
LB 0.00 Hz
GB 0
PC 10.00

1D NMR plot parameters
CX 20.00 cm
CY 10.00 cm
F1P 11.000 ppm
F1 6582.40 Hz
F2P -0.500 ppm
F2 -299.20 Hz
PPMCM 0.57500 ppm/cm
HZCM 344.08002 Hz/cm

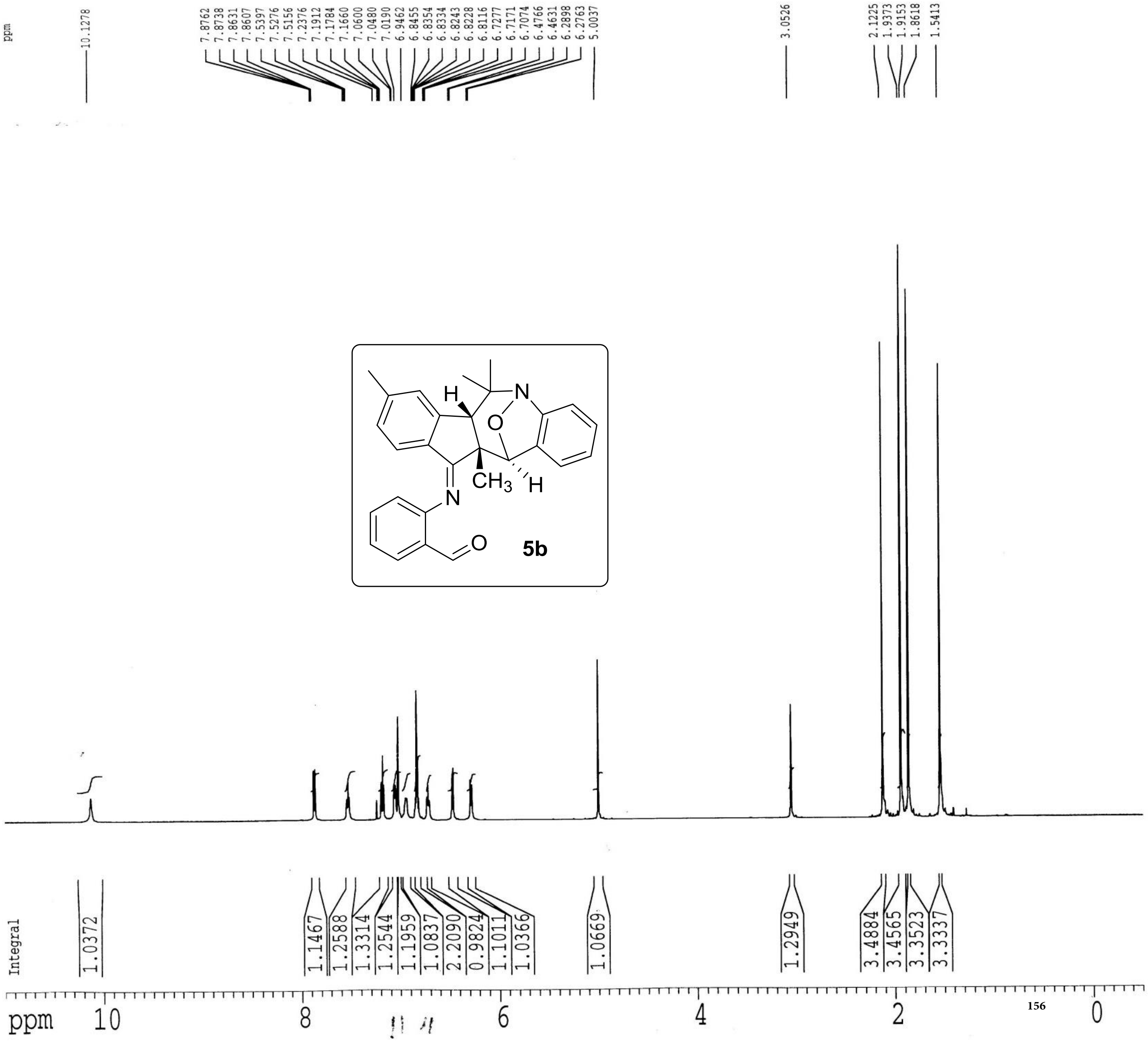

Current Data Parameters
NAME RKS-5-235-P2-HT
EXPNO 2
PROCNO 1

F2 - Acquisition Parameters
Date_ 20170915
Time 12.09
INSTRUM spect
PROBHD 5 mm QNP 1H/1
PULPROG zgpg
TD 32768
SOLVENT CDCl3
NS 200
DS 0
SWH 45045.047 Hz
FIDRES 1.374666 Hz
AQ 0.3637748 sec
RG 4096
DW 11.100 usec
DE 6.50 usec
TE 323.3 K
D1 3.50000000 sec
d11 0.03000000 sec
DELTA 3.40000010 sec
MCREST 0.00000000 sec
MCWRK 0.01500000 sec

======== CHANNEL f1 ========
NUC1 13C
P1 4.80 usec
PL1 0.00 dB
SFO1 150.4828468 MHz

======== CHANNEL f2 ========
CPDPRG2 waltz16
NUC2 1H
PCPD2 92.00 usec
PL2 120.00 dB
PL12 5.50 dB
PL13 9.00 dB
SFO2 598.4029920 MHz

F2 - Processing parameters
SI 65536
SF 150.4677918 MHz
WDW EM
SSB 0
LB 3.00 Hz
GB 0
PC 1.00

1D NMR plot parameters
CX 20.00 cm
CY 4.00 cm
F1P 200.000 ppm
F1 30093.56 Hz
F2P 0.000 ppm
F2 0.00 Hz
PPMCM 10.00000 ppm/cm
HZCM 1504.67786 Hz/cm

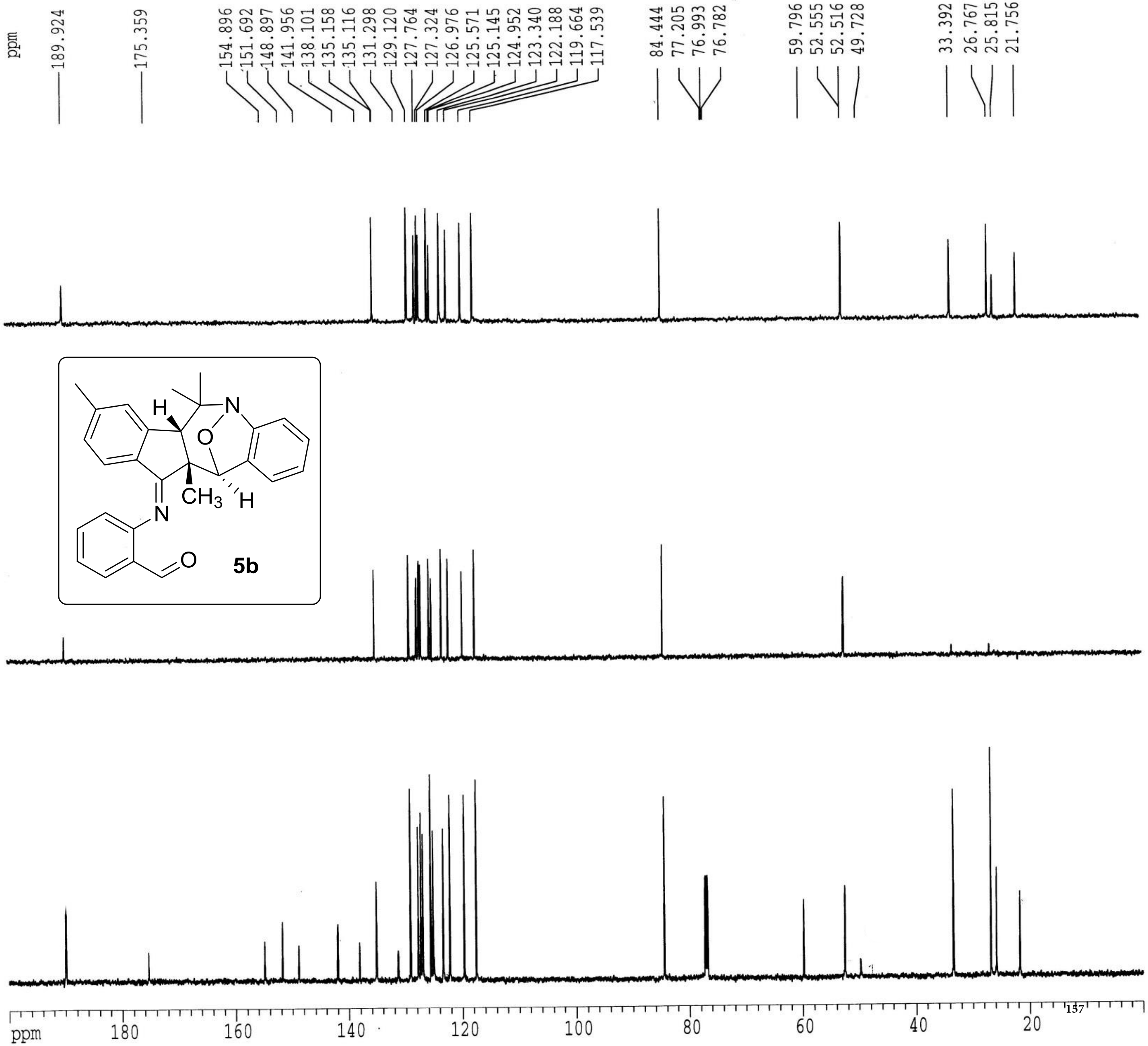

5b

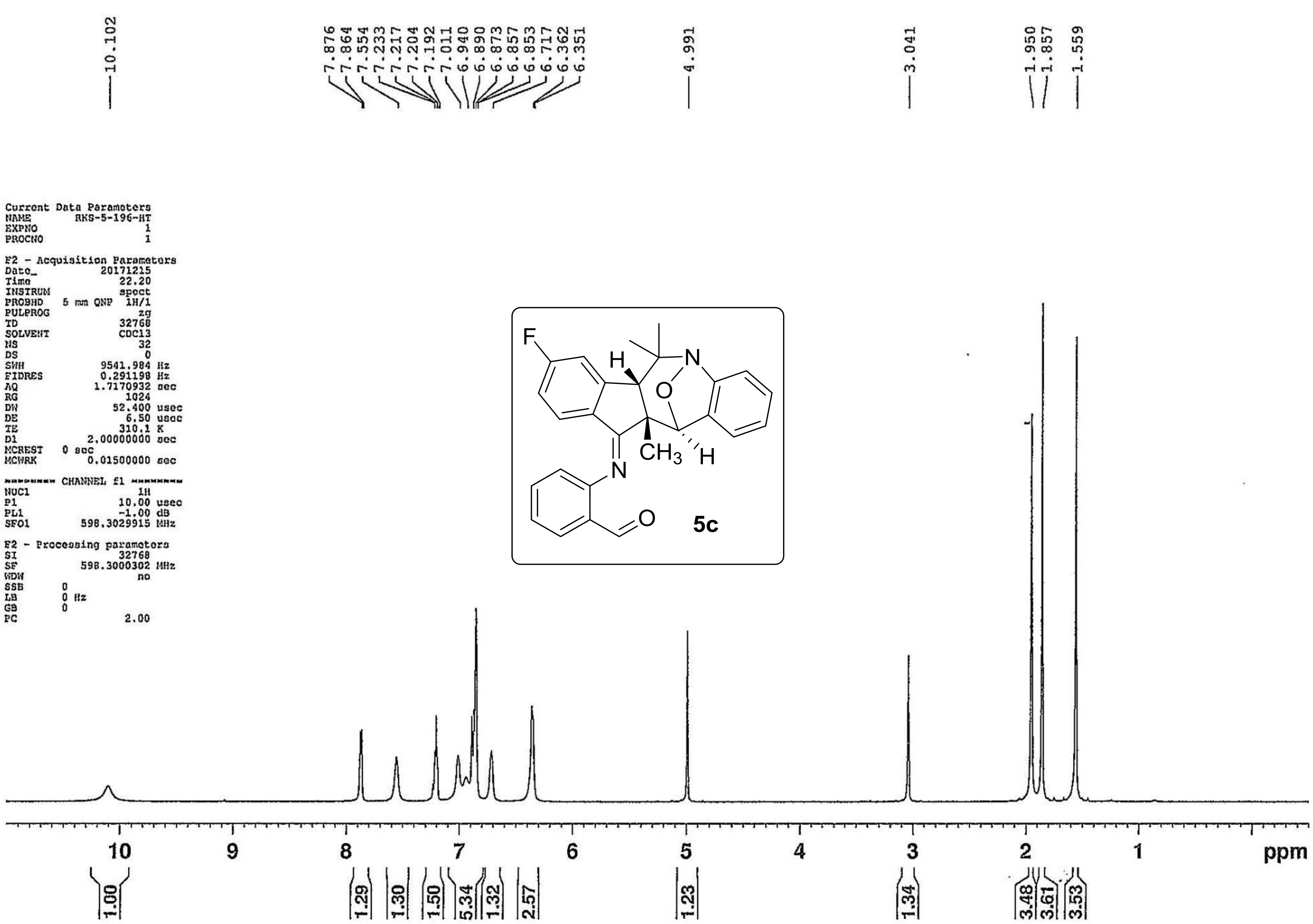
10.102
7.876
7.864
7.554
7.233
7.217
7.204
7.192
7.011
6.940
6.890
6.873
6.857
6.853
6.717
6.362
6.351
4.991
3.041
1.950
1.857
1.559
Current Data Parameters
NAME RKS-5-196-HT
EXPNO 1
PROCNO 1
F2 - Acquisition Parameters
Date_ 20171215
Time 22.20
INSTRUM spect
PROBHD 5 mm QNP 1H/1
PULPROG zg
TD 32768
SOLVENT CDCl3
NS 32
DS 0
SWH 9541.984 Hz
FIDRES 0.291198 Hz
AQ 1.7170932 sec
RG 1024
DW 52.400 usec
DE 6.50 usec
TE 310.1 K
D1 2.00000000 sec
MCREST 0 sec
MCWRK 0.01500000 sec
======== CHANNEL f1 ========
NUC1 1H
P1 10.00 usec
PL1 -1.00 dB
SFO1 598.3029915 MHz
F2 - Processing parameters
SI 32768
SF 598.3000302 MHz
WDW no
SSB 0
LB 0 Hz
GB 0
PC 2.00
F
H
N
O
CH3
H
N
O
5c
10
9
8
7
6
5
4
3
2
1
ppm
1.00
1.29
1.30
1.50
5.34
1.32
2.57
1.23
1.34
3.48
3.61
3.53

Current Data Parameters
NAME RKS-5-196-HT
EXPNO 2
PROCNO 1

F2 - Acquisition Parameters
Date_ 20171215
Time 6.33
INSTRUM spect
PROBHD 5 mm QNP 1H/1
PULPROG zgpg
TD 32768
SOLVENT CDCl3
NS 200
DS 0
SWH 45045.047 Hz
FIDRES 1.374666 Hz
AQ 0.3637748 sec
RG 4096
DW 11.100 usec
DE 6.50 usec
TE 310.1 K
D1 3.50000000 sec
d11 0.03000000 sec
DELTA 3.40000010 sec
MCREST 0.00000000 sec
MCWRK 0.01500000 sec

======== CHANNEL f1 ========
NUC1 13C
P1 4.80 usec
PL1 0.00 dB
SFO1 150.4592037 MHz

======== CHANNEL f2 ========
CPDPRG2 waltz16
NUC2 1H
PCPD2 92.00 usec
PL2 120.00 dB
PL12 5.50 dB
PL13 9.00 dB
SFO2 598.3029915 MHz

F2 - Processing parameters
SI 65536
SF 150.4426536 MHz
WDW EM
SSB 0
LB 3.00 Hz
GB 0
PC 1.00

1D NMR plot parameters
CX 20.00 cm
CY 4.00 cm
F1P 200.000 ppm
F1 30088.53 Hz
F2P 0.000 ppm
F2 0.00 Hz
PPMCM 10.00000 ppm/cm
HZCM 1504.42651 Hz/cm

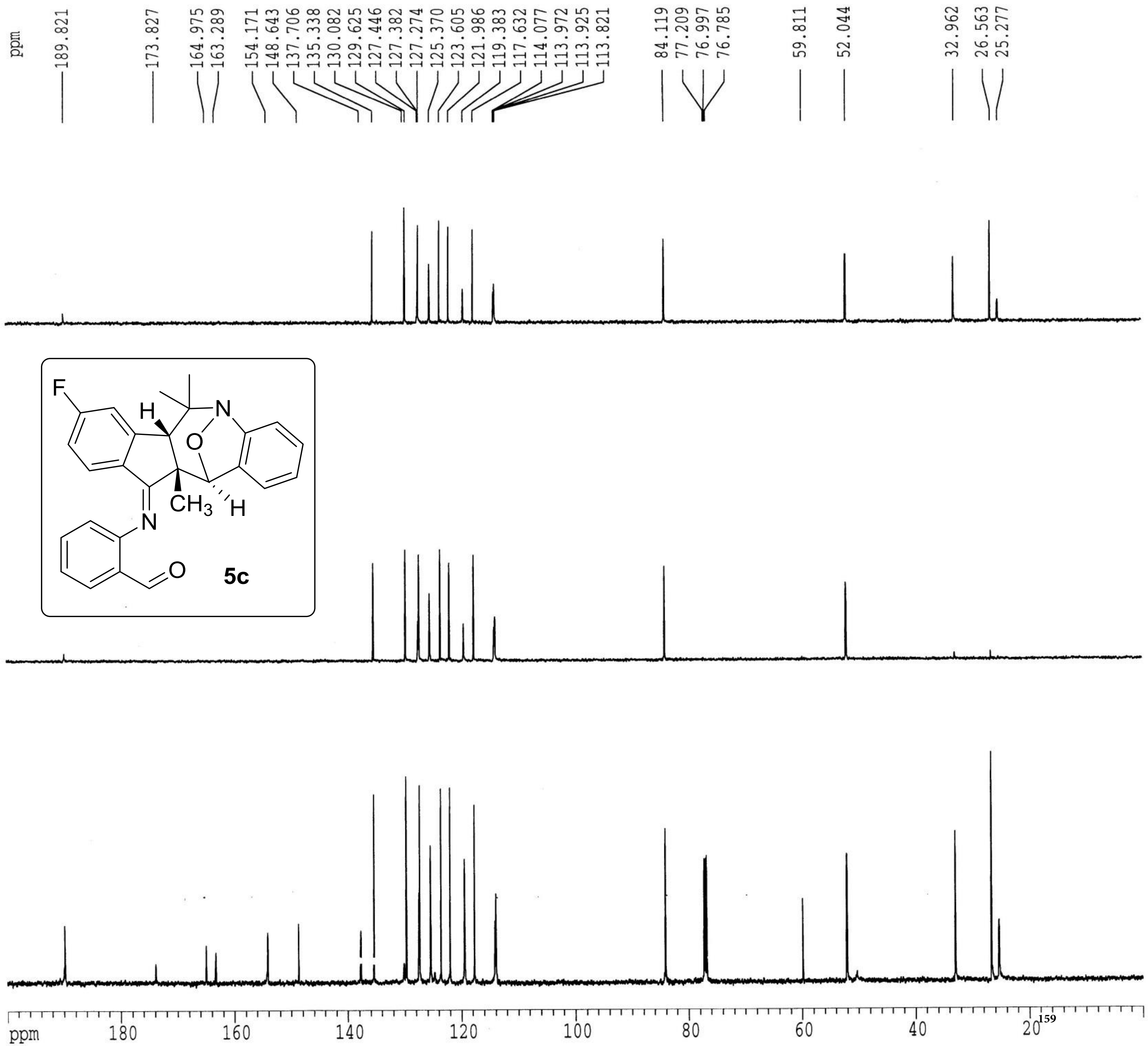

Current Data Parameters
NAME RKS-5-181-HT
EXPNO 100
PROCNO 1

F2 - Acquisition Parameters
Date_ 20171116
Time 11.52
INSTRUM spect
PROBHD 5 mm QNP 1H/1
PULPROG zg
TD 32768
SOLVENT CDCl3
NS 32
DS 0
SWH 9541.984 Hz
FIDRES 0.291198 Hz
AQ 1.7170932 sec
RG 512
DW 52.400 usec
DE 6.50 usec
TE 319.7 K
D1 2.00000000 sec
MCREST 0.00000000 sec
MCWRK 0.01500000 sec

======== CHANNEL f1 ========
NUC1 1H
P1 10.00 usec
PL1 -1.00 dB
SFO1 598.4032912 MHz

F2 - Processing parameters
SI 32768
SF 598.4000252 MHz
WDW no
SSB 0
LB 0.00 Hz
GB 0
PC 1.00

1D NMR plot parameters
CX 20.00 cm
CY 12.00 cm
F1P 11.000 ppm
F1 6582.40 Hz
F2P -0.500 ppm
F2 -299.20 Hz
PPMCM 0.57500 ppm/cm
HZCM 344.08002 Hz/cm

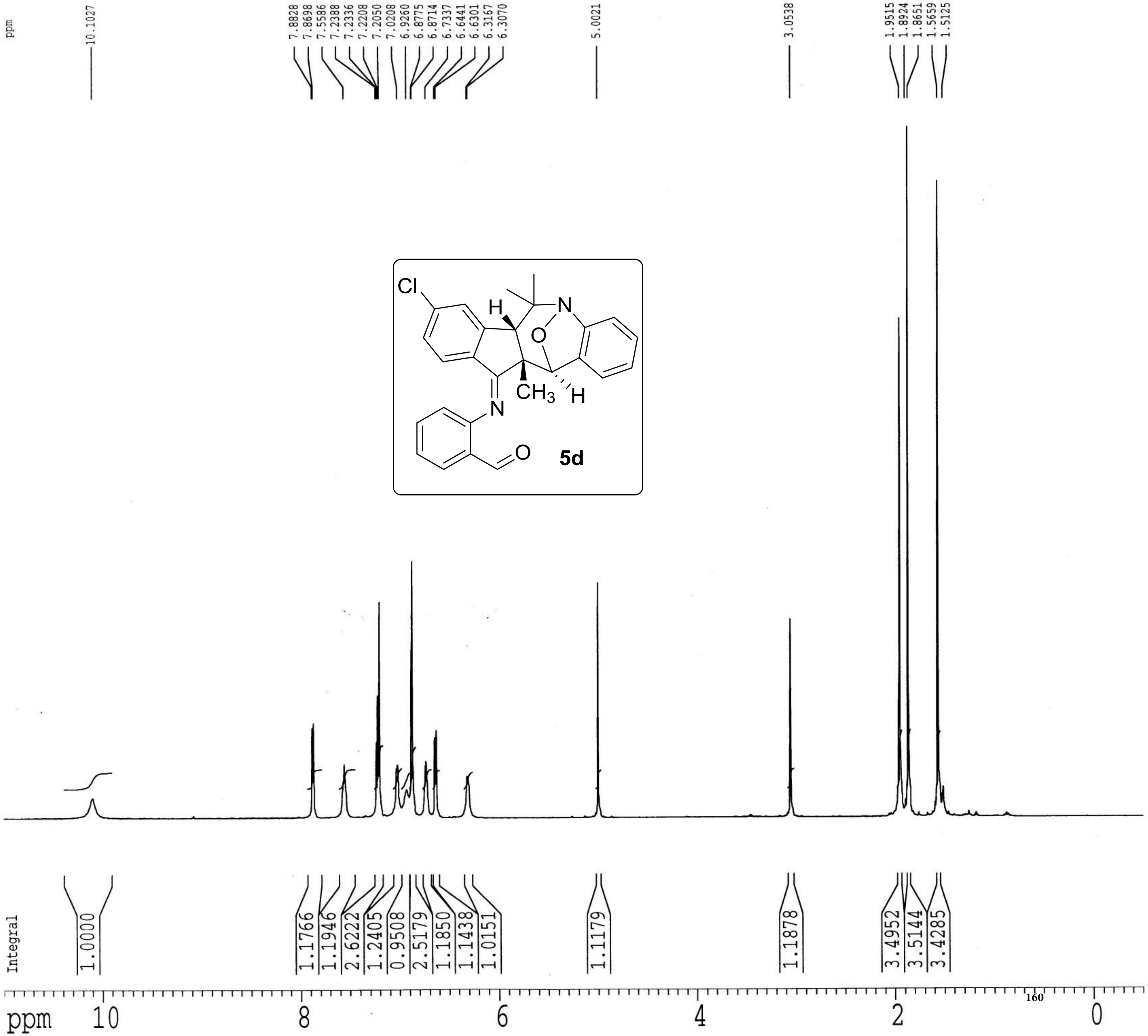

Current Data Parameters
NAME RKS-5-181-HT
EXPNO 2
PROCNO 1

F2 - Acquisition Parameters
Date_ 20171116
Time 7.47
INSTRUM spect
PROBHD 5 mm QNP 1H/1
PULPROG zgpg
TD 32768
SOLVENT CDCl3
NS 200
DS 0
SWH 45045.047 Hz
FIDRES 1.374666 Hz
AQ 0.3637748 sec
RG 4096
DW 11.100 usec
DE 6.50 usec
TE 326.5 K
D1 3.50000000 sec
d11 0.03000000 sec
DELTA 3.40000010 sec
MCREST 0.00000000 sec
MCWRK 0.01500000 sec

======== CHANNEL f1 ========
NUC1 13C
P1 4.80 usec
PL1 0.00 dB
SFO1 150.4843515 MHz

======== CHANNEL f2 ========
CPDPRG2 waltz16
NUC2 1H
PCPD2 92.00 usec
PL2 120.00 dB
PL12 5.50 dB
PL13 9.00 dB
SFO2 598.4029920 MHz

F2 - Processing parameters
SI 65536
SF 150.4677850 MHz
WDW EM
SSB 0
LB 3.00 Hz
GB 0
PC 1.00

1D NMR plot parameters
CX 20.00 cm
CY 3.00 cm
F1P 200.000 ppm
F1 30093.56 Hz
F2P 0.000 ppm
F2 0.00 Hz
PPMCM 10.00000 ppm/cm
HZCM 1504.67786 Hz/cm

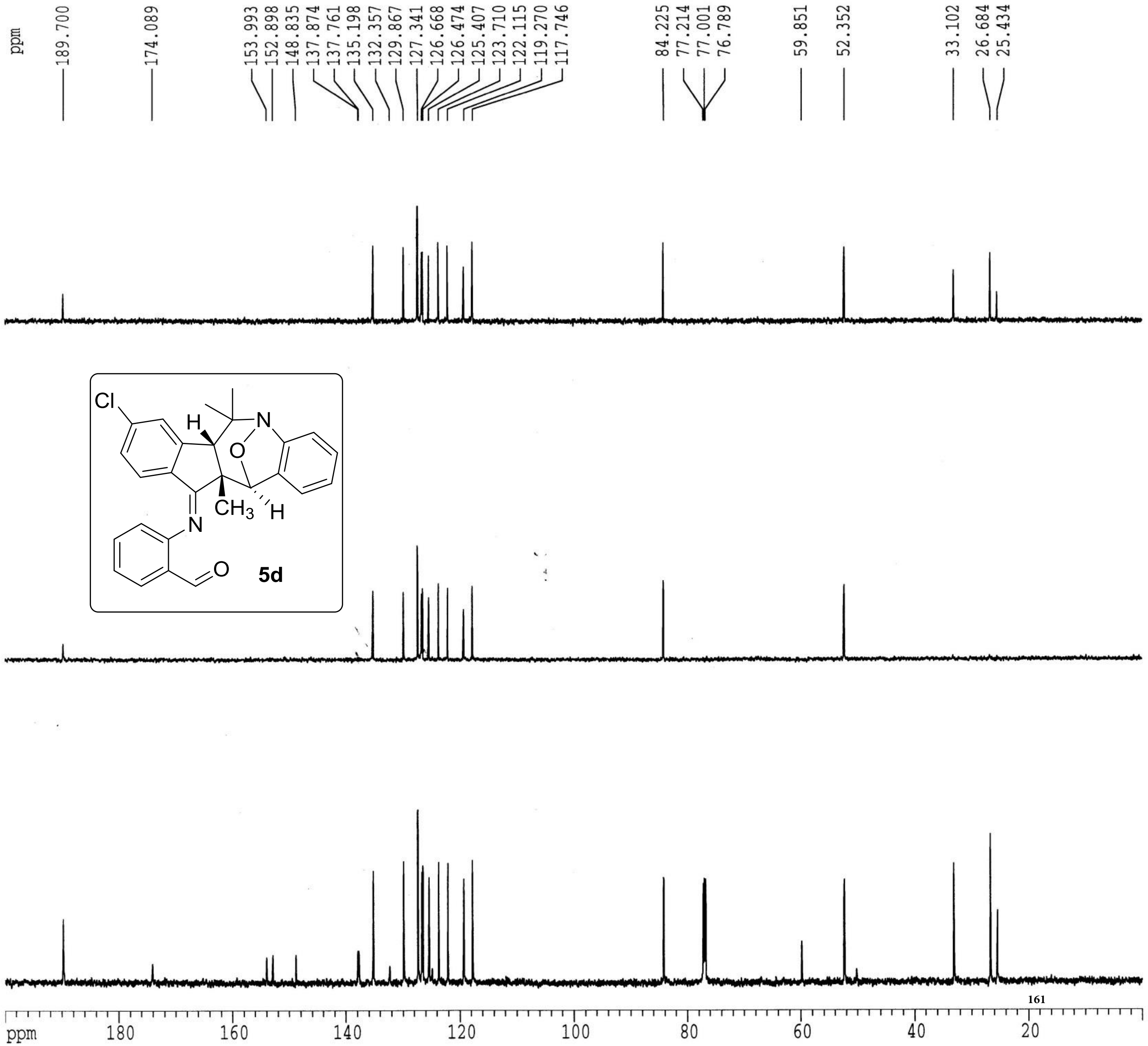

Current Data Parameters
NAME RKS-5-191-HT
EXPNO 1
PROCNO 1

F2 - Acquisition Parameters
Date_ 20171215
Time 5.08
INSTRUM spect
PROBHD 5 mm QNP 1H/1
PULPROG zg
TD 32768
SOLVENT CDCl3
NS 32
DS 0
SWH 9541.984 Hz
FIDRES 0.291198 Hz
AQ 1.7170932 sec
RG 1024
DW 52.400 usec
DE 6.50 usec
TE 310.5 K
D1 2.00000000 sec
MCREST 0.00000000 sec
MCWRK 0.01500000 sec

======== CHANNEL f1 ========
NUC1 1H
P1 10.00 usec
PL1 -1.00 dB
SFO1 598.3029915 MHz

F2 - Processing parameters
SI 32768
SF 598.3000302 MHz
WDW no
SSB 0
LB 0.00 Hz
GB 0
PC 2.00

1D NMR plot parameters
CX 20.00 cm
CY 20.00 cm
F1P 11.000 ppm
F1 6581.30 Hz
F2P -0.500 ppm
F2 -299.15 Hz
PPMCM 0.57500 ppm/cm
HZCM 344.02252 Hz/cm

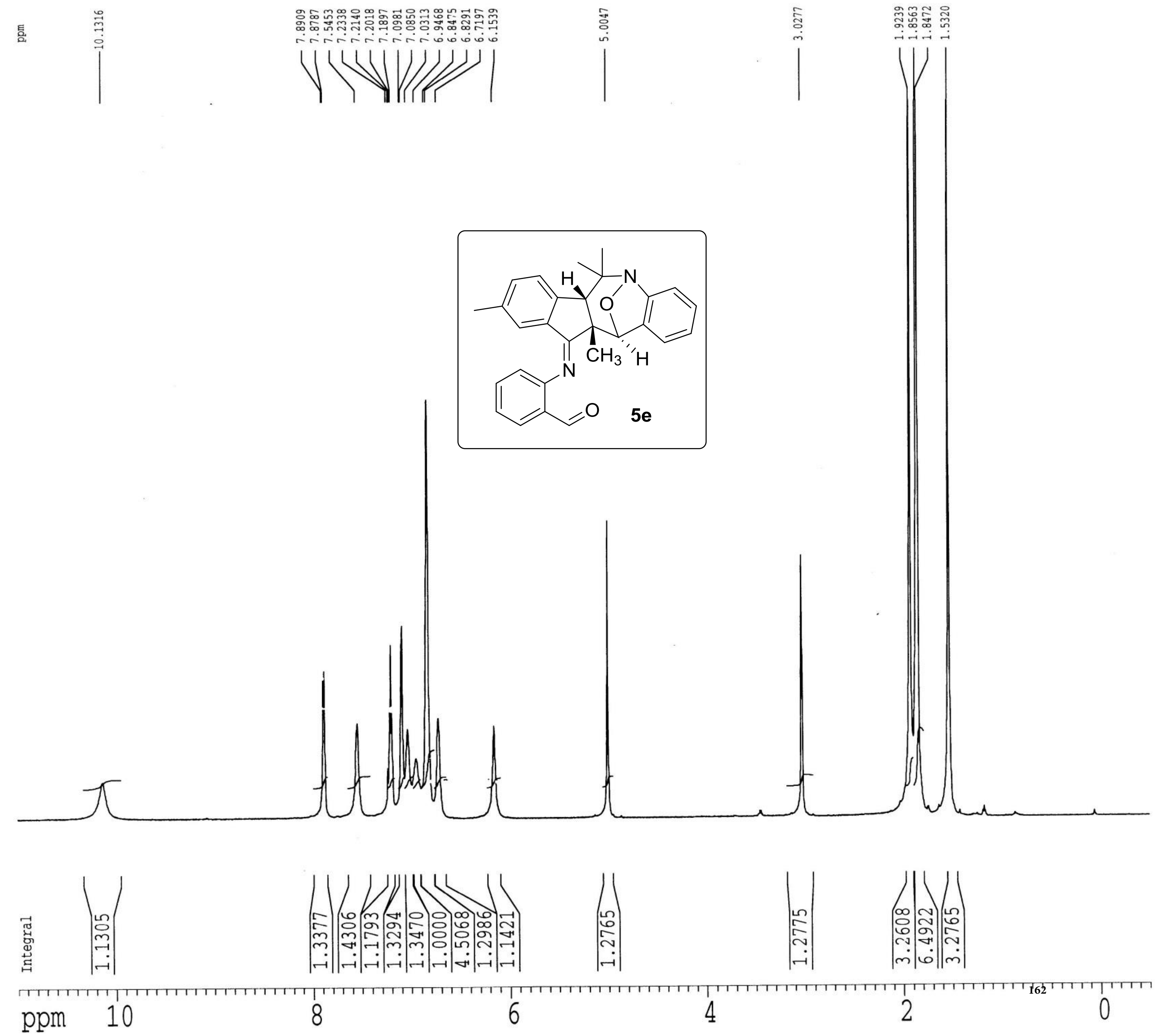

Current Data Parameters
NAME RKS-5-191-HT
EXPNO 2
PROCNO 1

F2 - Acquisition Parameters
Date_ 20171215
Time 5.16
INSTRUM spect
PROBHD 5 mm QNP 1H/1
PULPROG zgpg
TD 32768
SOLVENT CDCl3
NS 300
DS 0
SWH 45045.047 Hz
FIDRES 1.374666 Hz
AQ 0.3637748 sec
RG 4096
DW 11.100 usec
DE 6.50 usec
TE 310.4 K
D1 3.50000000 sec
d11 0.03000000 sec
DELTA 3.40000010 sec
MCREST 0.00000000 sec
MCWRK 0.01500000 sec

======== CHANNEL f1 ========
NUC1 13C
P1 4.80 usec
PL1 0.00 dB
SFO1 150.4592037 MHz

======== CHANNEL f2 ========
CPDPRG2 waltz16
NUC2 1H
PCPD2 92.00 usec
PL2 120.00 dB
PL12 5.50 dB
PL13 9.00 dB
SFO2 598.3029915 MHz

F2 - Processing parameters
SI 65536
SF 150.4426509 MHz
WDW EM
SSB 0
LB 3.00 Hz
GB 0
PC 1.00

1D NMR plot parameters
CX 20.00 cm
CY 4.00 cm
F1P 200.000 ppm
F1 30088.53 Hz
F2P 0.000 ppm
F2 0.00 Hz
PPMCM 10.00000 ppm/cm
HZCM 1504.42651 Hz/cm

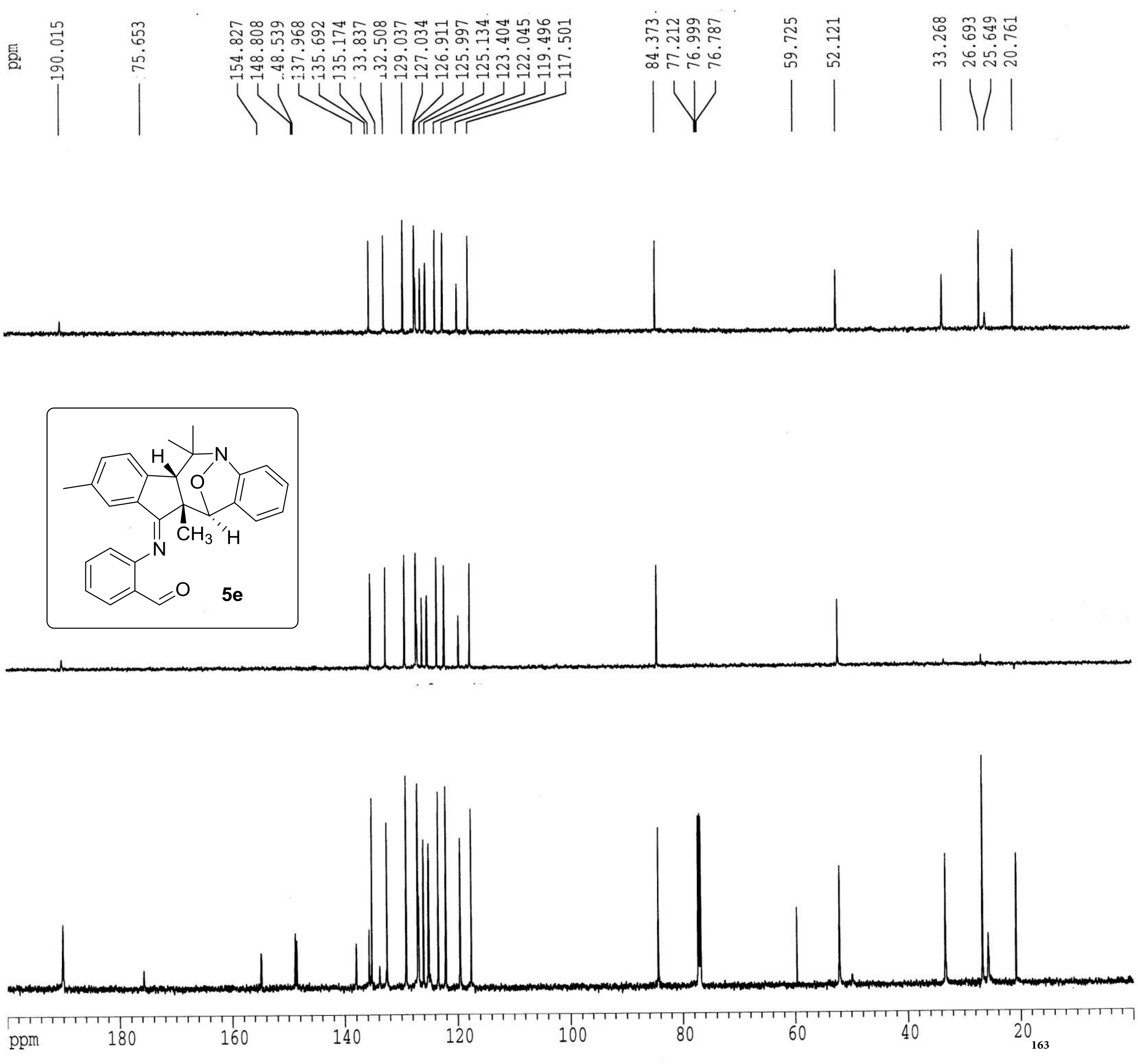

Current Data Parameters
NAME RKS-5-186-HT
EXPNO 1
PROCNO 1

F2 - Acquisition Parameters
Date_ 20171116
Time 9.53
INSTRUM spect
PROBHD 5 mm QNP 1H/1
PULPROG zg
TD 32768
SOLVENT CDCl3
NS 32
DS 0
SWH 9541.984 Hz
FIDRES 0.291198 Hz
AQ 1.7170932 sec
RG 512
DW 52.400 usec
DE 6.50 usec
TE 322.0 K
D1 2.00000000 sec
MCREST 0.00000000 sec
MCWRK 0.01500000 sec

======== CHANNEL f1 ========
NUC1 1H
P1 10.00 usec
PL1 -1.00 dB
SFO1 598.4032912 MHz

F2 - Processing parameters
SI 32768
SF 598.4000252 MHz
WDW no
SSB 0
LB 0.00 Hz
GB 0
PC 1.00

1D NMR plot parameters
CX 26.00 cm
CY 12.00 cm
F1P 11.000 ppm
F1 6582.40 Hz
F2P -0.500 ppm
F2 -299.20 Hz
PPMCM 0.57500 ppm/cm
HZCM 344.08002 Hz/cm

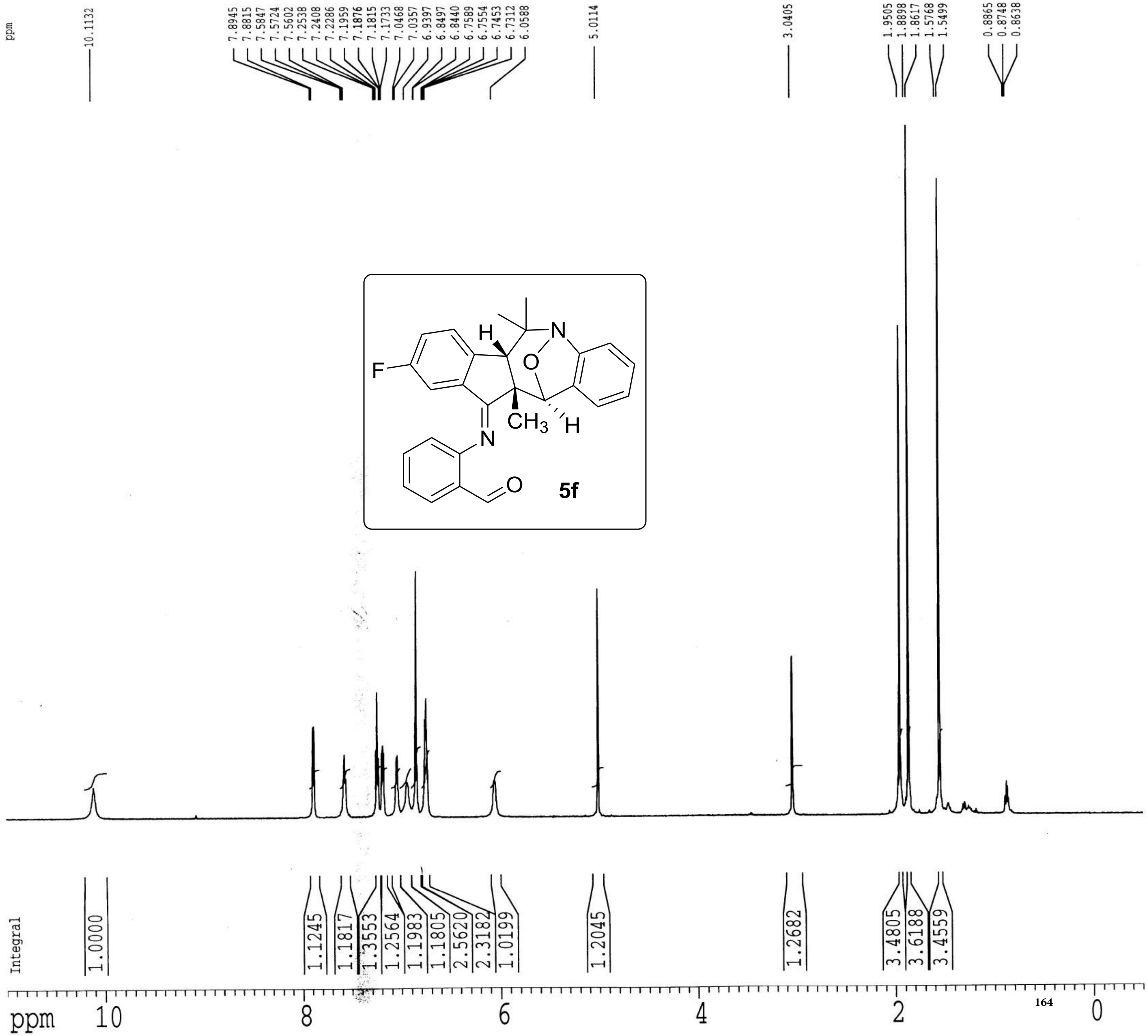

Current Data Parameters
NAME RKS-5-186-HT
EXPNO 2
PROCNO 1

F2 - Acquisition Parameters
Date_ 20171116
Time 9.55
INSTRUM spect
PROBHD 5 mm QNP 1H/1
PULPROG zgpg
TD 32768
SOLVENT CDCl3
NS 600
DS 0
SWH 45045.047 Hz
FIDRES 1.374666 Hz
AQ 0.3637748 sec
RG 4096
DW 11.100 usec
DE 6.50 usec
TE 322.1 K
D1 3.50000000 sec
d11 0.03000000 sec
DELTA 3.40000010 sec
MCREST 0.00000000 sec
MCWRK 0.01500000 sec

======== CHANNEL f1 ========
NUC1 13C
P1 4.80 usec
PL1 0.00 dB
SFO1 150.4843515 MHz

======== CHANNEL f2 ========
CPDPRG2 waltz16
NUC2 1H
PCPD2 92.00 usec
PL2 120.00 dB
PL12 5.50 dB
PL13 9.00 dB
SFO2 598.4029920 MHz

F2 - Processing parameters
SI 65536
SF 150.4677864 MHz
WDW EM
SSB 0
LB 3.00 Hz
GB 0
PC 1.00

1D NMR plot parameters
CX 20.00 cm
CY 3.50 cm
F1P 200.000 ppm
F1 30093.56 Hz
F2P 0.000 ppm
F2 0.00 Hz
PPMCM 10.00000 ppm/cm
HZCM 1504.67786 Hz/cm

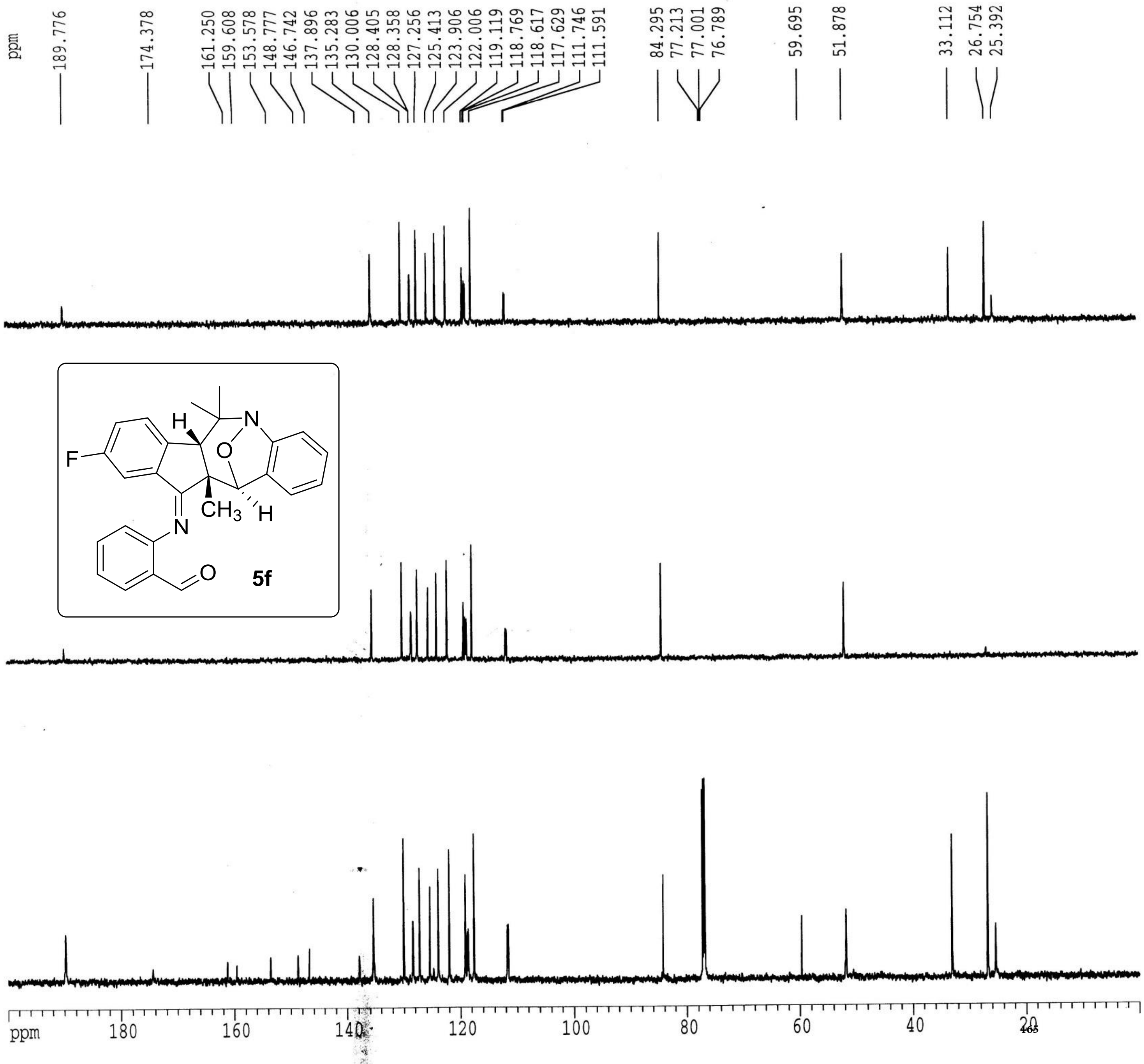

Current Data Parameters
NAME RKS-5-209-HT
EXPNO 1
PROCNO 1

F2 - Acqui..ion Parameters
Date_ 20180104
Time 11.54
INSTRUM spect
PROBHD 5 mm QNP 1H/1
PULPROG zg
TD 32768
SOLVENT CDCl3
NS 32
DS 0
SWH 9541.984 Hz
FIDRES 0.291198 Hz
AQ 1.7170932 sec
RG 1024
DW 52.400 usec
DE 6.50 usec
TE 307.7 K
D1 2.00000000 sec
MCREST 0.00000000 sec
MCWRK 0.01500000 sec

======== CHANNEL f1 ========
NUC1 1H
P1 10.00 usec
PL1 -1.00 dB
SFO1 598.3032907 MHz

F2 - Processing parameters
SI 32768
SF 598.3000276 MHz
WDW no
SSB 0
LB 0.00 Hz
GB 0
PC 2.00

1D NMR plot parameters
CX 20.00 cm
CY 8.00 cm
F1P 11.000 ppm
F1 6581.30 Hz
F2P -0.500 ppm
F2 -299.15 Hz
PPMCM 0.57500 ppm/cm
HZCM 344.02252 Hz/cm

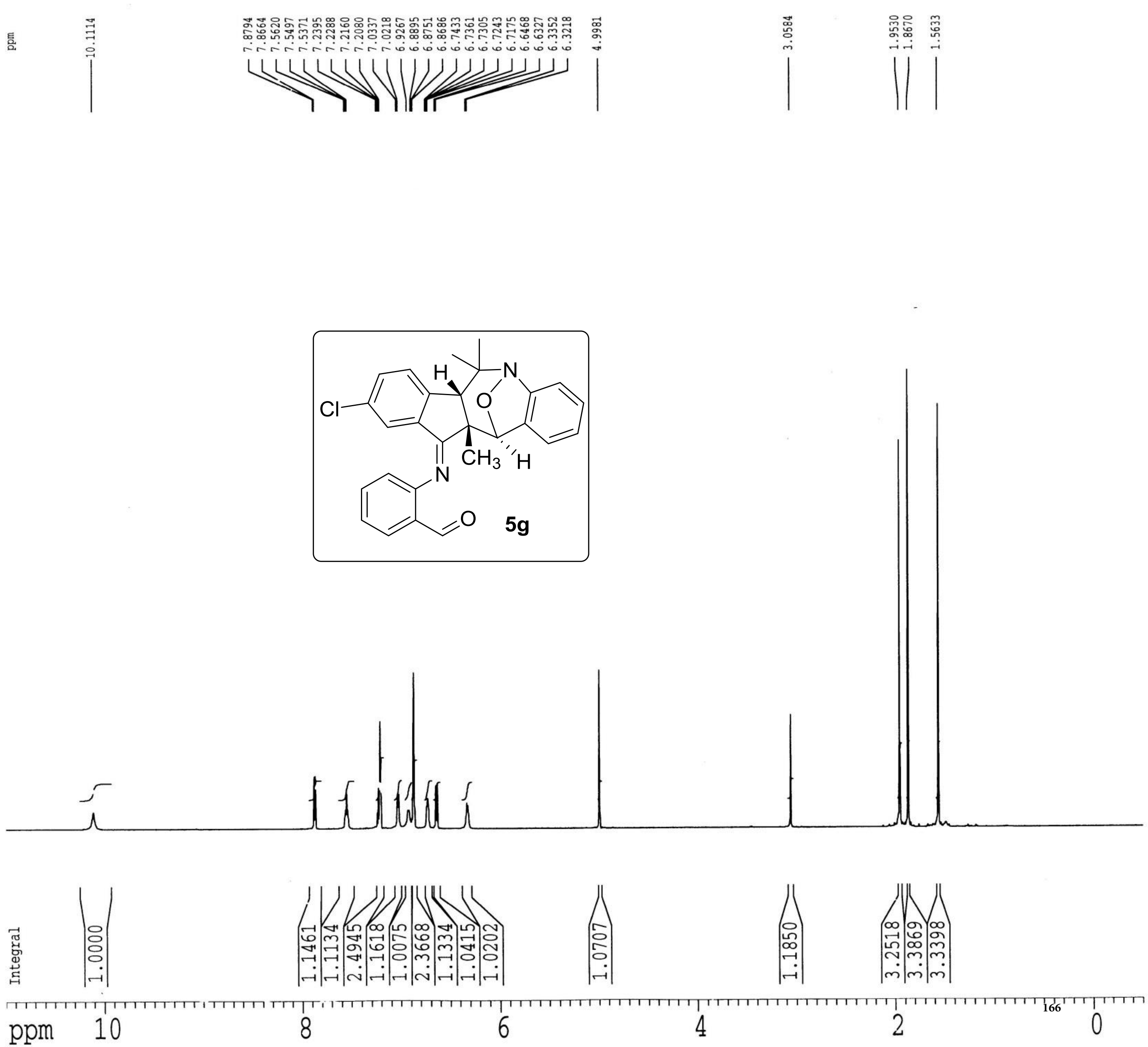

Current Data Parameters
NAME ;KS-5-209-HT
EXPNO 2
PROCNO 1

F2 - Acquisition Parameters
Date_ 20180104
Time 11.14
INSTRUM spect
PROBHD 5 mm QNP 1H/1
PULPROG zgpg
TD 32768
SOLVENT CDCl3
NS 266
DS 0
SWH 45045.047 Hz
FIDRES 1.374666 Hz
AQ 0.3637748 sec
RG 4096
DW 11.100 usec
DE 6.50 usec
TE 306.4 K
D1 3.50000000 sec
d11 0.03000000 sec
DELTA 3.40000010 sec
MCREST 0.00000000 sec
MCWRK 0.01500000 sec

======== CHANNEL f1 ========
NUC1 13C
P1 4.80 usec
PL1 0.00 dB
SFO1 150.4592037 MHz

======== CHANNEL f2 ========
CPDPRG2 waltz16
NUC2 1H
PCPD2 92.00 usec
PL2 120.00 dB
PL12 5.50 dB
PL13 9.00 dB
SFO2 598.3029915 MHz

F2 - Processing parameters
SI 65536
SF 150.4426474 MHz
WDW EM
SSB 0
LB 3.00 Hz
GB 0
PC 1.00

1D NMR plot parameters
CX 20.00 cm
CY 4.00 cm
F1P 200.000 ppm
F1 30088.53 Hz
F2P 0.000 ppm
F2 0.00 Hz
PPMCM 10.00000 ppm/cm
HZCM 1504.42639 Hz/cm

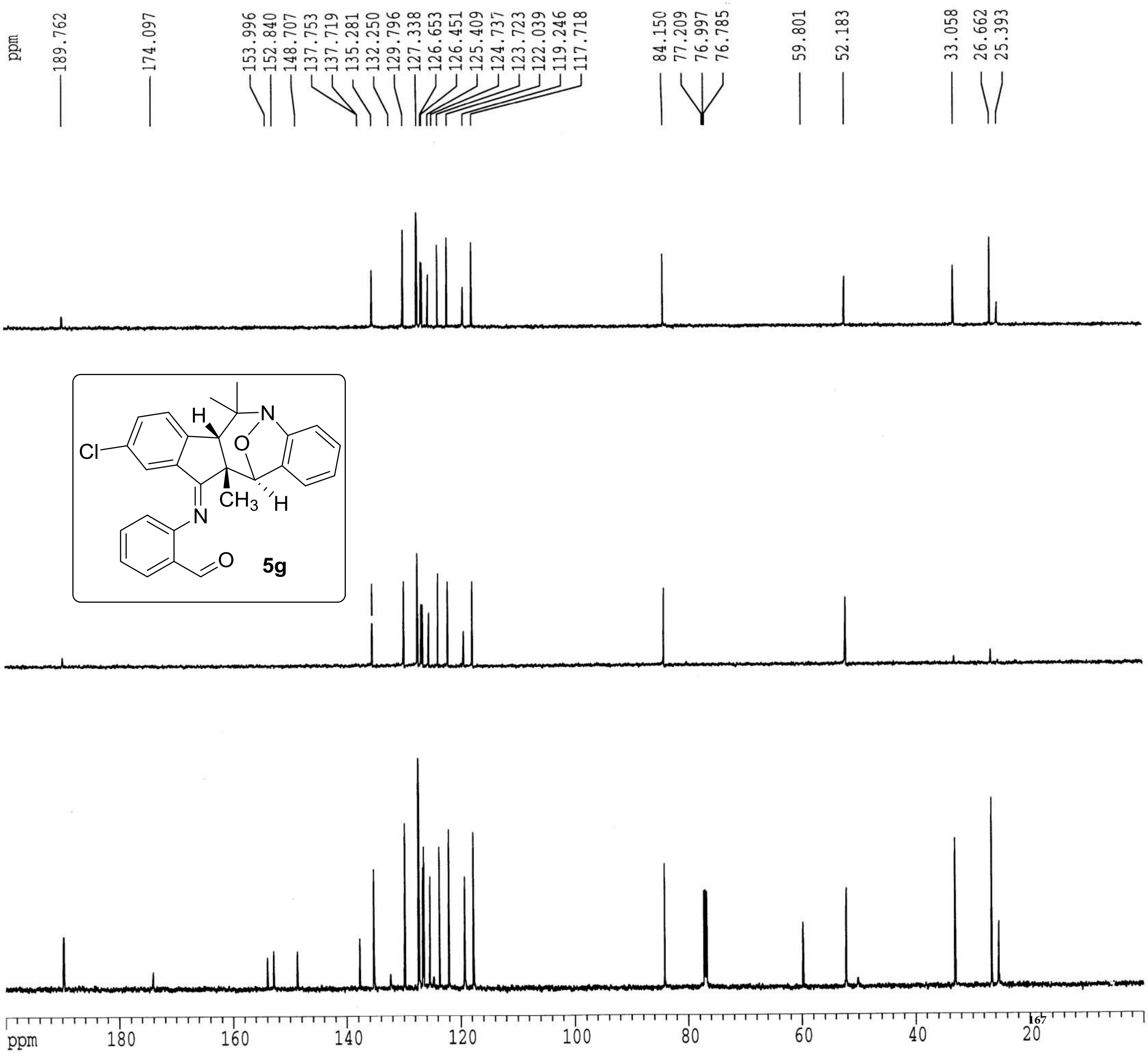

```
Current Data Parameters
NAME        RKS-5-180-HT
EXPNO                  1
PROCNO                 1

F2 - Acquisition Parameters
Date_           20171116
Time               22.54
INSTRUM            spect
PROBHD   5 mm QNP  1H/1
PULPROG               zg
TD                 32768
SOLVENT            CDCl3
NS                    32
DS                     0
SWH             9541.984 Hz
FIDRES          0.291198 Hz
AQ             1.7170932 sec
RG                   512
DW                52.400 usec
DE                  6.50 usec
TE                 320.8 K
D1            2.00000000 sec
MCREST   0 sec
MCWRK         0.01500000 sec

======== CHANNEL f1 ========
NUC1                  1H
P1                 10.00 usec
PL1                -1.00 dB
SFO1         598.4032912 MHz

F2 - Processing parameters
SI                 32768
SF           598.4000252 MHz
WDW                   no
SSB      0
LB       0 Hz
GB       0
PC                  1.00
```

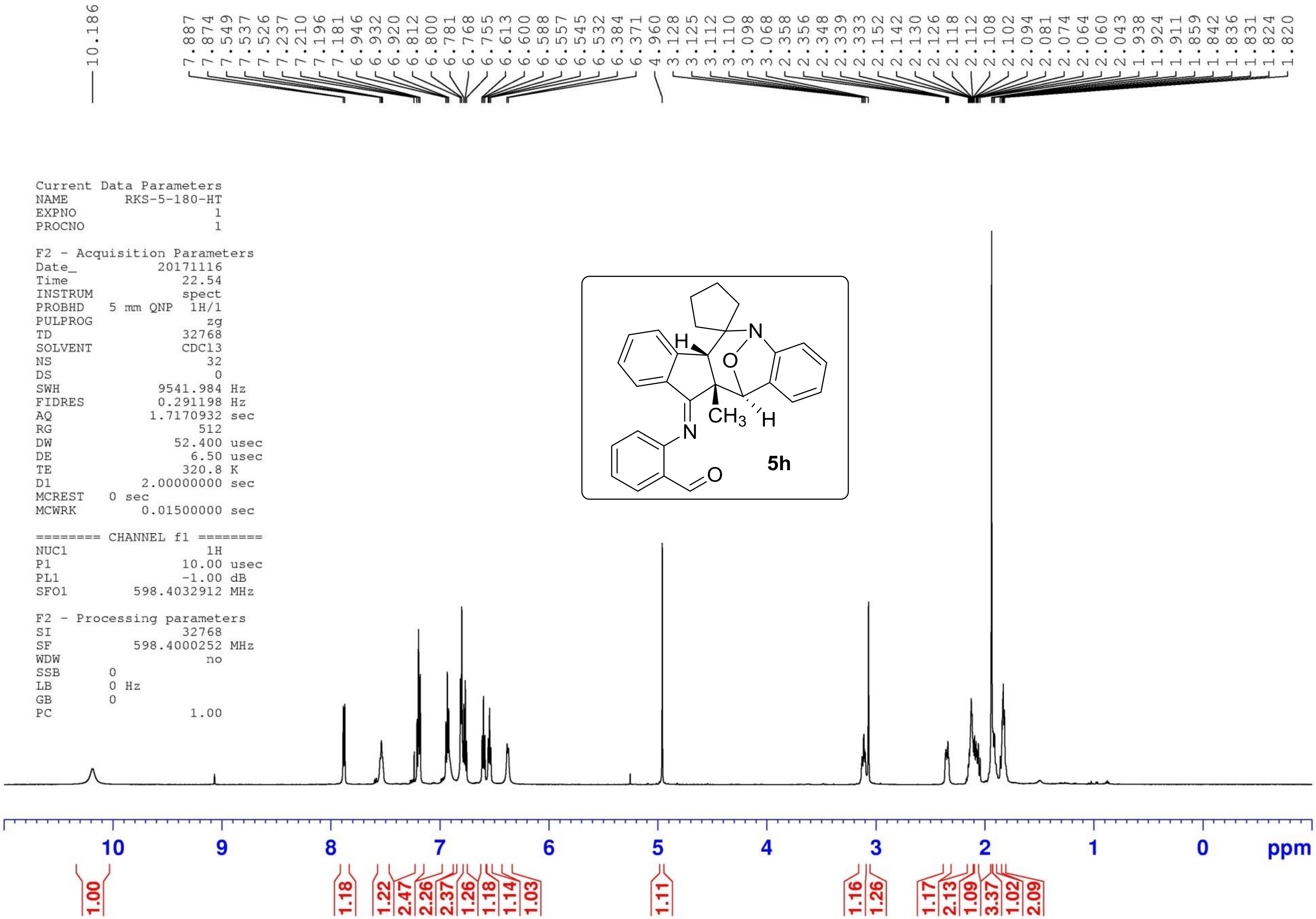

Current Data Parameters
NAME RKS-5-180-HT
EXPNO 2
PROCNO 1

F2 - Acquisition Parameters
Date_ 20171116
Time 6.55
INSTRUM spect
PROBHD 5 mm QNP 1H/1
PULPROG zgpg
TD 32768
SOLVENT CDCl3
NS 200
DS 0
SWH 45045.047 Hz
FIDRES 1.374666 Hz
AQ 0.3637748 sec
RG 4096
DW 11.100 usec
DE 6.50 usec
TE 321.1 K
D1 3.50000000 sec
d11 0.03000000 sec
DELTA 3.40000010 sec
MCREST 0.00000000 sec
MCWRK 0.01500000 sec

======== CHANNEL f1 ========
NUC1 13C
P1 4.80 usec
PL1 0.00 dB
SFO1 150.4843515 MHz

======== CHANNEL f2 ========
CPDPRG2 waltz16
NUC2 1H
PCPD2 92.00 usec
PL2 120.00 dB
PL12 5.50 dB
PL13 9.00 dB
SFO2 598.4029920 MHz

F2 - Processing parameters
SI 65536
SF 150.4677891 MHz
WDW EM
SSB 0
LB 3.00 Hz
GB 0
PC 1.00

1D NMR plot parameters
CX 20.00 cm
CY 4.00 cm
F1P 200.000 ppm
F1 30093.56 Hz
F2P 0.000 ppm
F2 0.00 Hz
PPMCM 10.00000 ppm/cm
HZCM 1504.67786 Hz/cm

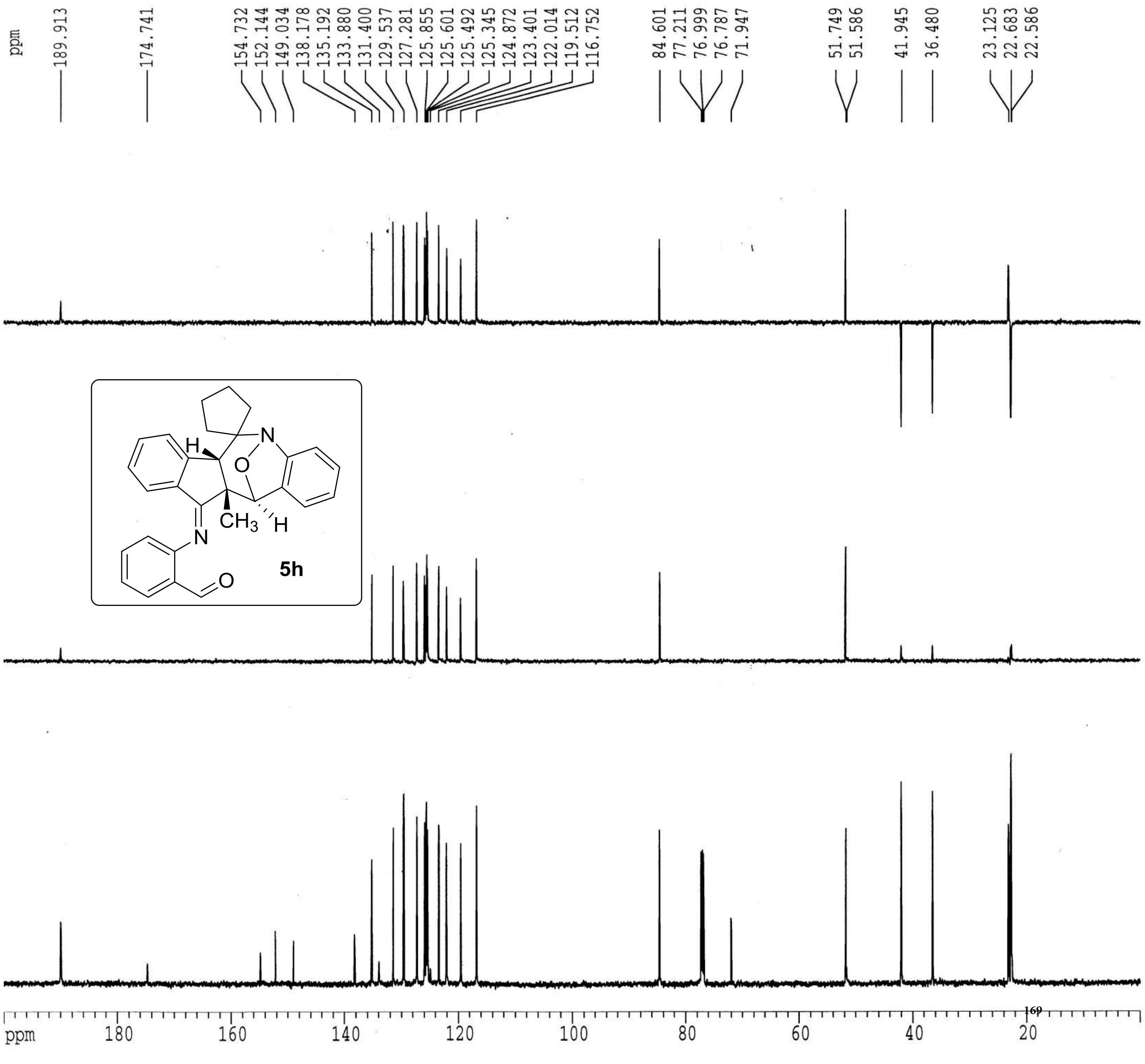

Current Data Parameters
NAME RKS-5-169-HT
EXPNO 1
PROCNO 1

F2 - Acquisition Parameters
Date_ 20171031
Time 7.28
INSTRUM spect
PROBHD 5 mm QNP 1H/1
PULPROG zg
TD 32768
SOLVENT CDCl3
NS 32
DS 0
SWH 10775.862 Hz
FIDRES 0.328853 Hz
AQ 1.5204852 sec
RG 512
DW 46.400 usec
DE 6.50 usec
TE 328.2 K
D1 2.00000000 sec
MCREST 0.00000000 sec
MCWRK 0.01500000 sec

======== CHANNEL f1 ========
NUC1 1H
P1 9.60 usec
PL1 1.00 dB
SFO1 598.4035904 MHz

F2 - Processing parameters
SI 32768
SF 598.4000252 MHz
WDW GM
SSB 0
LB -1.00 Hz
GB 0.5
PC 1.00

1D NMR plot parameters
CX 20.00 cm
CY 10.00 cm
F1P 11.000 ppm
F1 6582.40 Hz
F2P -0.500 ppm
F2 -299.20 Hz
PPMCM 0.57500 ppm/cm
HZCM 344.08002 Hz/cm

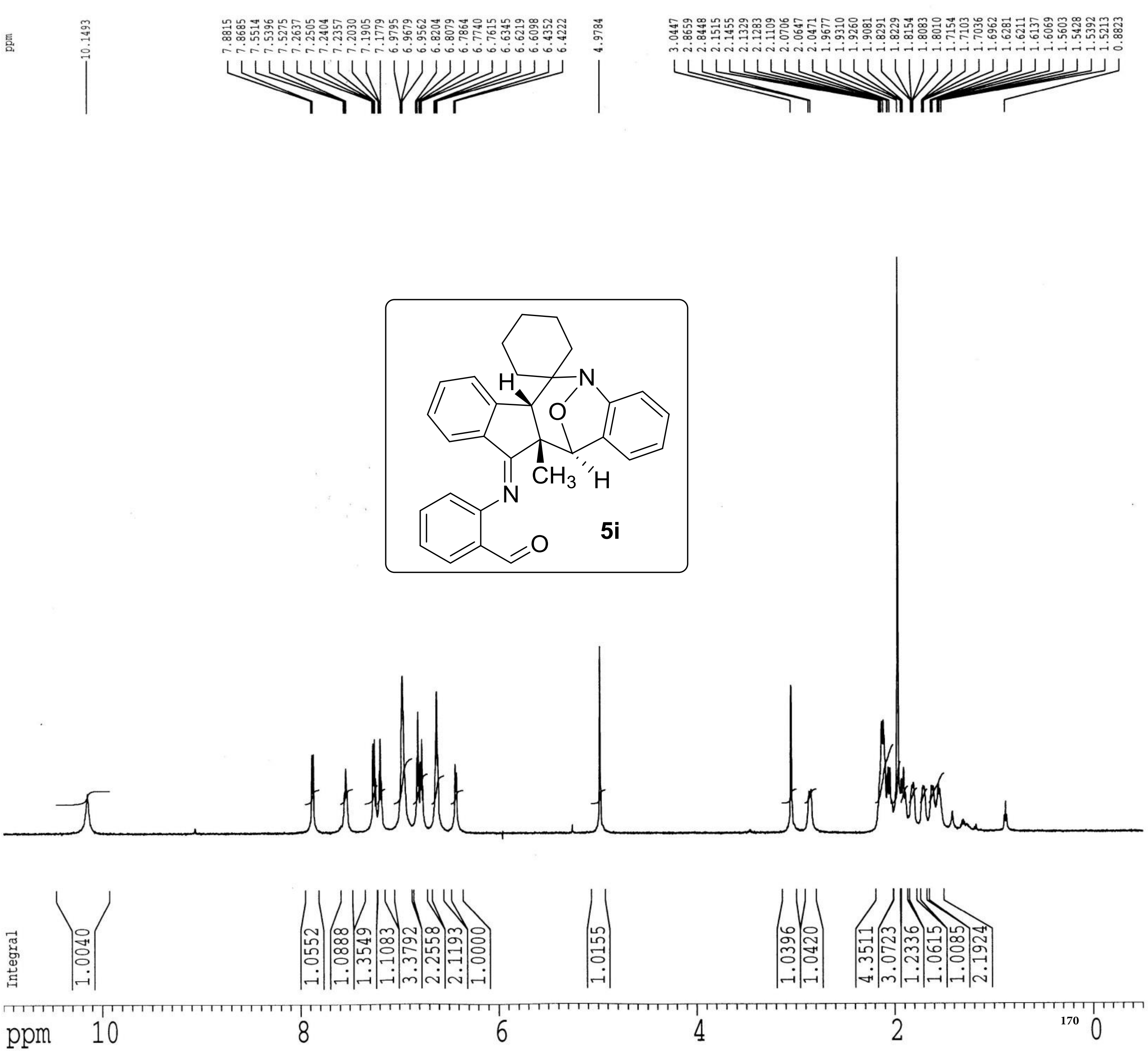

Current Data Parameters
NAME RKS-5-169-HT
EXPNO 2
PROCNO 1

F2 - Acquisition Parameters
Date_ 20171031
Time 7.39
INSTRUM spect
PROBHD 5 mm QNP 1H/1
PULPROG zgpg
TD 32768
SOLVENT CDCl3
NS 1024
DS 0
SWH 45045.047 Hz
FIDRES 1.374666 Hz
AQ 0.3637748 sec
RG 4096
DW 11.100 usec
DE 6.50 usec
TE 328.5 K
D1 3.50000000 sec
d11 0.03000000 sec
DELTA 3.40000010 sec
MCREST 0.00000000 sec
MCWRK 0.01500000 sec

======== CHANNEL f1 ========
NUC1 13C
P1 4.80 usec
PL1 0.00 dB
SFO1 150.4843515 MHz

======== CHANNEL f2 ========
CPDPRG2 waltz16
NUC2 1H
PCPD2 92.00 usec
PL2 120.00 dB
PL12 5.50 dB
PL13 9.00 dB
SFO2 598.4029920 MHz

F2 - Processing parameters
SI 65536
SF 150.4677802 MHz
WDW EM
SSB 0
LB 3.00 Hz
GB 0
PC 1.00

1D NMR plot parameters
CX 20.00 cm
CY 4.00 cm
F1P 200.000 ppm
F1 30093.55 Hz
F2P 0.000 ppm
F2 0.00 Hz
PPMCM 10.00000 ppm/cm
HZCM 1504.67773 Hz/cm

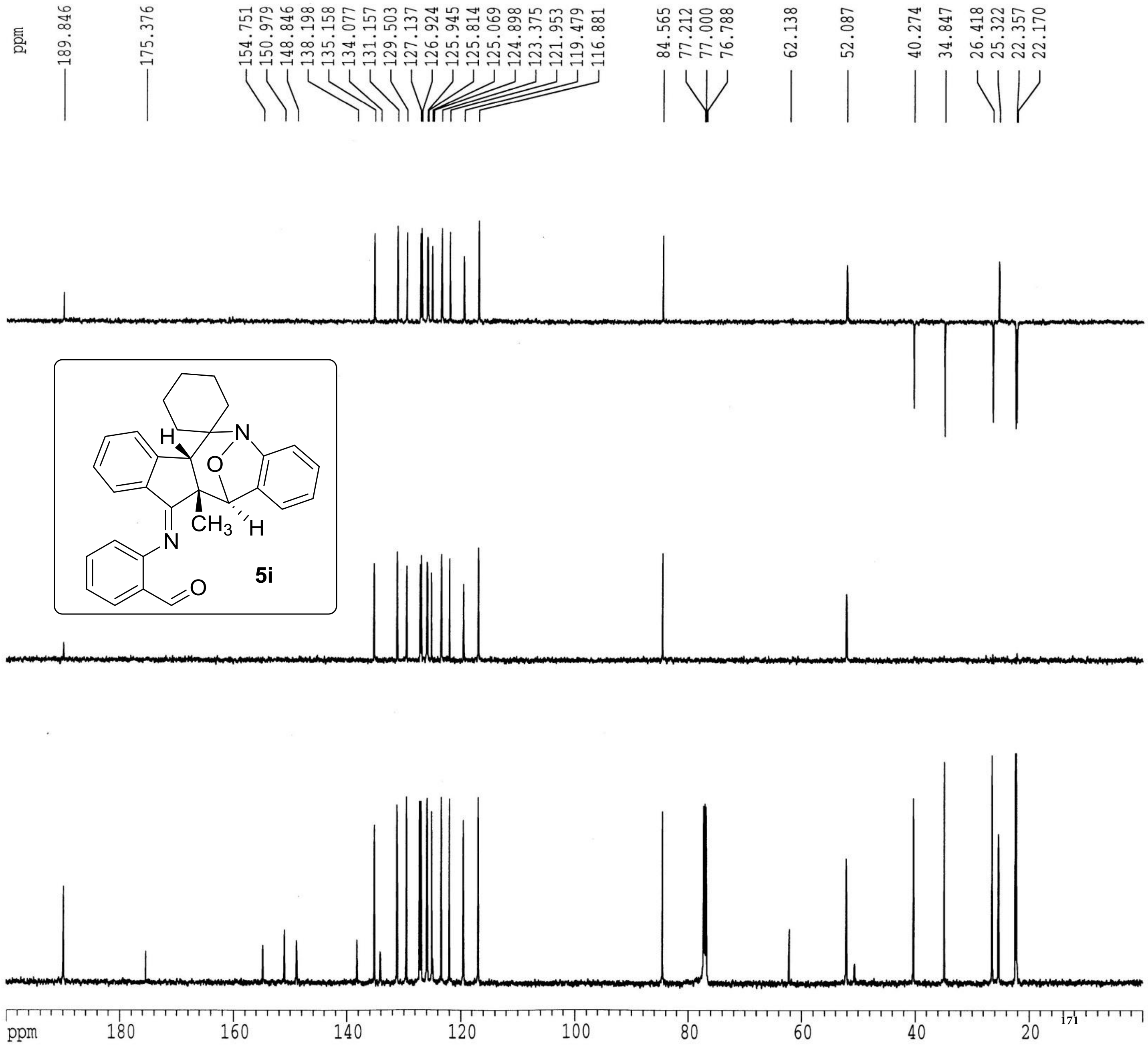

RKS-5-148-F3b

Current Data Parameters
NAME 20171002
EXPNO 1
PROCNO 1

F2 - Acquisition Parameters
Date_ 20171001
Time 22.58
INSTRUM spect
PROBHD 5 mm DUL 13C-1
PULPROG zg30
TD 32768
SOLVENT CDCl3
NS 18
DS 0
SWH 6410.256 Hz
FIDRES 0.195625 Hz
AQ 2.5559540 sec
RG 4
DW 78.000 usec
DE 6.00 usec
TE 300.0 K
D1 2.00000000 sec
TD0 1

======== CHANNEL f1 ========
NUC1 1H
P1 10.00 usec
PL1 -2.40 dB
SFO1 400.1528010 MHz

F2 - Processing parameters
SI 16384
SF 400.1500168 MHz
WDW EM
SSB 0
LB 0.00 Hz
GB 0
PC 1.00

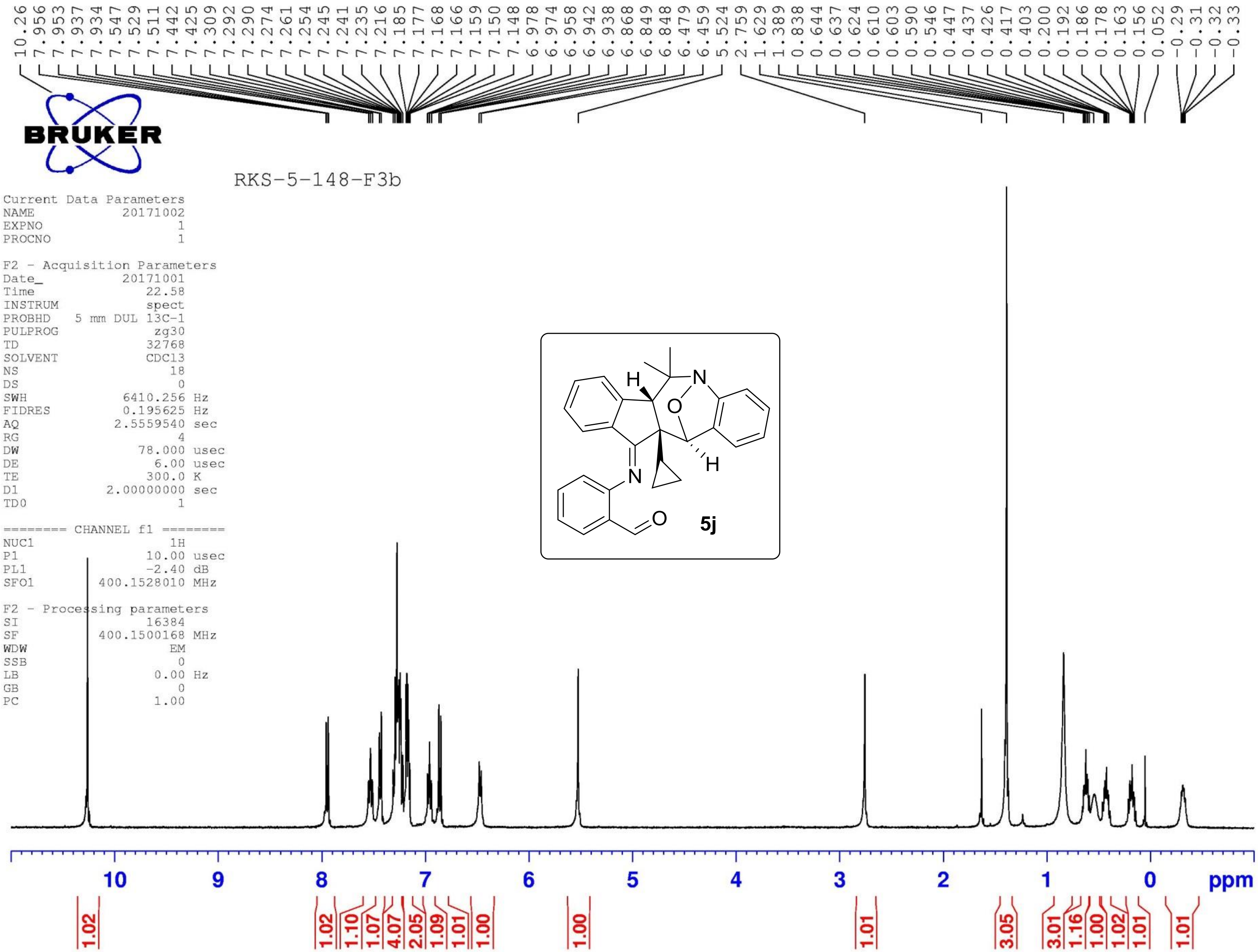

5j

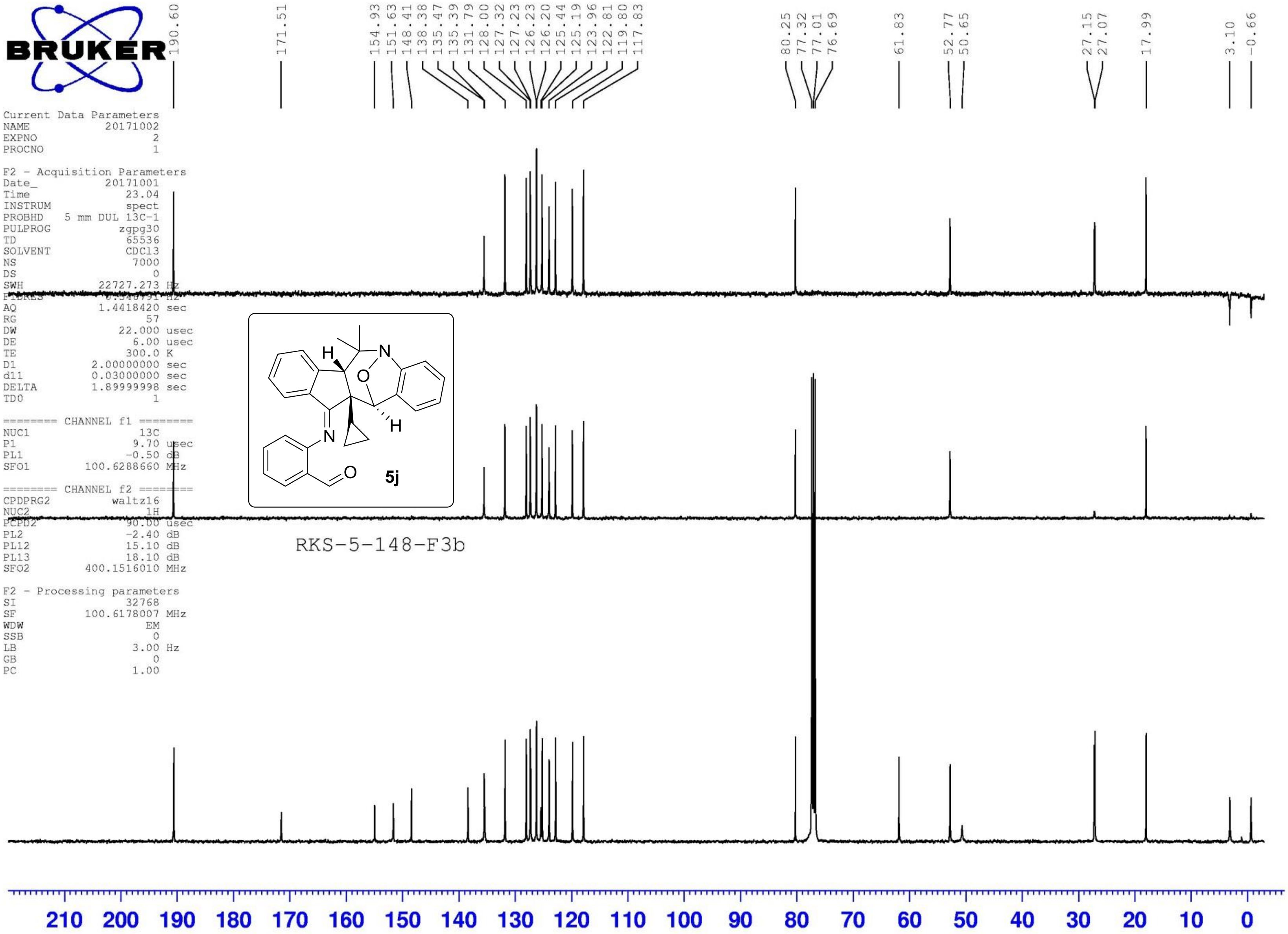

BRUKER
190.60
171.51
154.93
151.63
148.41
138.38
135.47
135.39
131.79
128.00
127.32
127.23
126.23
126.20
125.44
125.19
123.96
122.81
119.80
117.83
80.25
77.32
77.01
76.69
61.83
52.77
50.65
27.15
27.07
17.99
3.10
-0.66
Current Data Parameters
NAME 20171002
EXPNO 2
PROCNO 1
F2 - Acquisition Parameters
Date_ 20171001
Time 23.04
INSTRUM spect
PROBHD 5 mm DUL 13C-1
PULPROG zgpg30
TD 65536
SOLVENT CDCl3
NS 7000
DS 0
SWH 22727.273 Hz
AQ 1.4418420 sec
RG 57
DW 22.000 usec
DE 6.00 usec
TE 300.0 K
D1 2.00000000 sec
d11 0.03000000 sec
DELTA 1.89999998 sec
TD0 1
======== CHANNEL f1 ========
NUC1 13C
P1 9.70 usec
PL1 -0.50 dB
SFO1 100.6288660 MHz
======== CHANNEL f2 ========
CPDPRG2 waltz16
NUC2 1H
PCPD2 90.00 usec
PL2 -2.40 dB
PL12 15.10 dB
PL13 18.10 dB
SFO2 400.1516010 MHz
F2 - Processing parameters
SI 32768
SF 100.6178007 MHz
WDW EM
SSB 0
LB 3.00 Hz
GB 0
PC 1.00
5j
RKS-5-148-F3b
210 200 190 180 170 160 150 140 130 120 110 100 90 80 70 60 50 40 30 20 10 0

```
Current Data Parameters
NAME       RKS-6-73-P-HT
EXPNO                  1
PROCNO                 1

F2 - Acquisition Parameters
Date_           20180917
Time               10.22
INSTRUM            spect
PROBHD   5 mm QNP  1H/1
PULPROG               zg
TD                 32768
SOLVENT            CDCl3
NS                    32
DS                     0
SWH             8389.262 Hz
FIDRES          0.256020 Hz
AQ             1.9530228 sec
RG                   512
DW                59.600 usec
DE                  6.50 usec
TE                 317.9 K
D1            2.00000000 sec
MCREST    0 sec
MCWRK         0.01500000 sec

======== CHANNEL f1 ========
NUC1                  1H
P1                 10.00 usec
PL1                -5.00 dB
SFO1         598.3032907 MHz

F2 - Processing parameters
SI                 32768
SF           598.3000285 MHz
WDW                   no
SSB       0
LB        0 Hz
GB        0
PC                  2.00
```

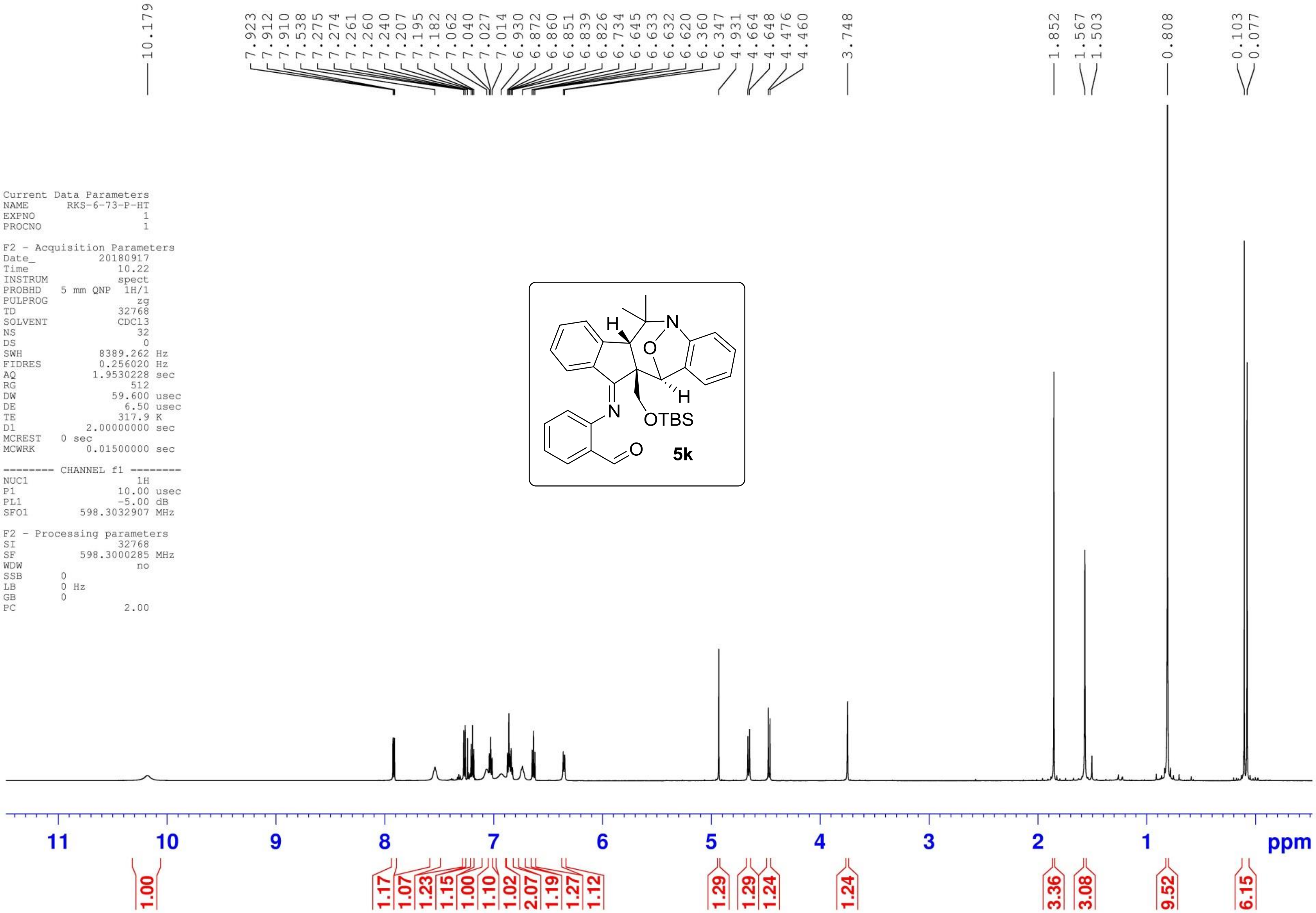

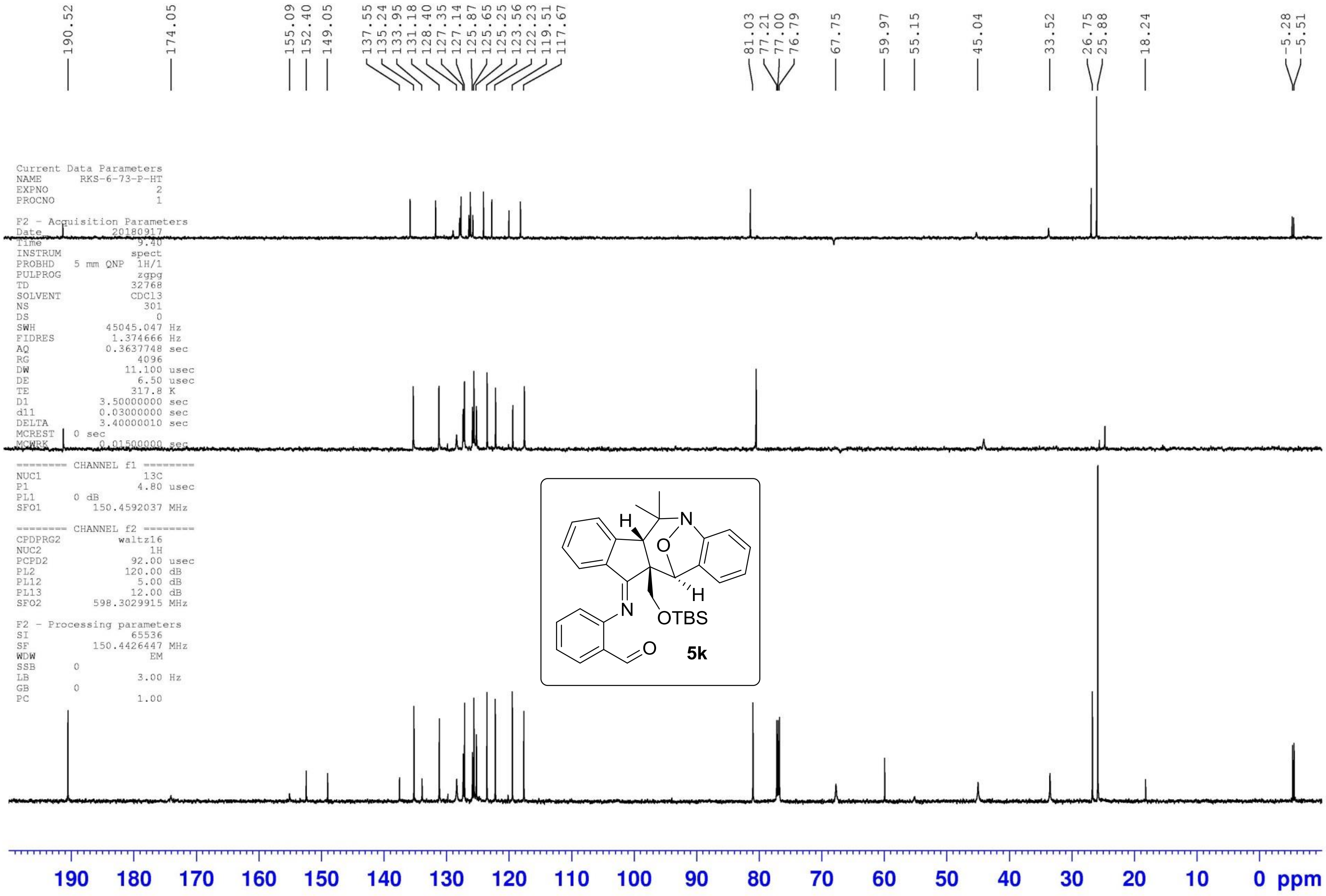
190.52
174.05
155.09
152.40
149.05
137.55
135.24
133.95
131.18
128.40
127.35
127.14
125.87
125.65
125.25
123.56
122.23
119.51
117.67
81.03
77.21
77.00
76.79
67.75
59.97
55.15
45.04
33.52
26.75
25.88
18.24
-5.28
-5.51
Current Data Parameters
NAME RKS-6-73-P-HT
EXPNO 2
PROCNO 1
F2 - Acquisition Parameters
Date 20180917
Time 9.40
INSTRUM spect
PROBHD 5 mm QNP 1H/1
PULPROG zgpg
TD 32768
SOLVENT CDCl3
NS 301
DS 0
SWH 45045.047 Hz
FIDRES 1.374666 Hz
AQ 0.3637748 sec
RG 4096
DW 11.100 usec
DE 6.50 usec
TE 317.8 K
D1 3.50000000 sec
d11 0.03000000 sec
DELTA 3.40000010 sec
MCREST 0 sec
MCWRK 0.01500000 sec
======== CHANNEL f1 ========
NUC1 13C
P1 4.80 usec
PL1 0 dB
SFO1 150.4592037 MHz
======== CHANNEL f2 ========
CPDPRG2 waltz16
NUC2 1H
PCPD2 92.00 usec
PL2 120.00 dB
PL12 5.00 dB
PL13 12.00 dB
SFO2 598.3029915 MHz
F2 - Processing parameters
SI 65536
SF 150.4426447 MHz
WDW EM
SSB 0
LB 3.00 Hz
GB 0
PC 1.00
H
N
O
H
N
OTBS
O
5k
190 180 170 160 150 140 130 120 110 100 90 80 70 60 50 40 30 20 10 0 ppm

Current Data Parameters
NAME RKS-5-157-HT
EXPNO 1
PROCNO 1

F2 - Acquisition Parameters
Date_ 20171016
Time 7.06
INSTRUM spect
PROBHD 5 mm QNP 1H/1
PULPROG noemul
TD 32768
SOLVENT CDCl3
NS 32
DS 0
SWH 8375.209 Hz
FIDRES 0.255591 Hz
AQ 1.9562995 sec
RG 512
DW 59.700 usec
DE 6.50 usec
TE 319.1 K
D1 1.00000000 sec
d11 0.03000000 sec
d12 0.00002000 sec
D20 0.05000000 sec
L4 50
MCREST 0.00000000 sec
MCWRK 0.01500000 sec

======== CHANNEL f1 ========
NUC1 1H
P1 10.00 usec
PL1 -1.00 dB
SFO1 598.4035904 MHz

======== CHANNEL f2 ========
FQ2LIST noedif.1
NUC2 1H
PL2 120.00 dB
PL14 50.00 dB
SFO2 598.6029930 MHz

F2 - Processing parameters
SI 32768
SF 598.4000244 MHz
WDW no
SSB 0
LB 0.00 Hz
GB 0
PC 1.00

1D NMR plot parameters
CX 20.00 cm
CY 10.00 cm
F1P 11.000 ppm
F1 6582.40 Hz
F2P -0.500 ppm
F2 -299.20 Hz
PPMCM 0.57500 ppm/cm
HZCM 344.08002 Hz/cm

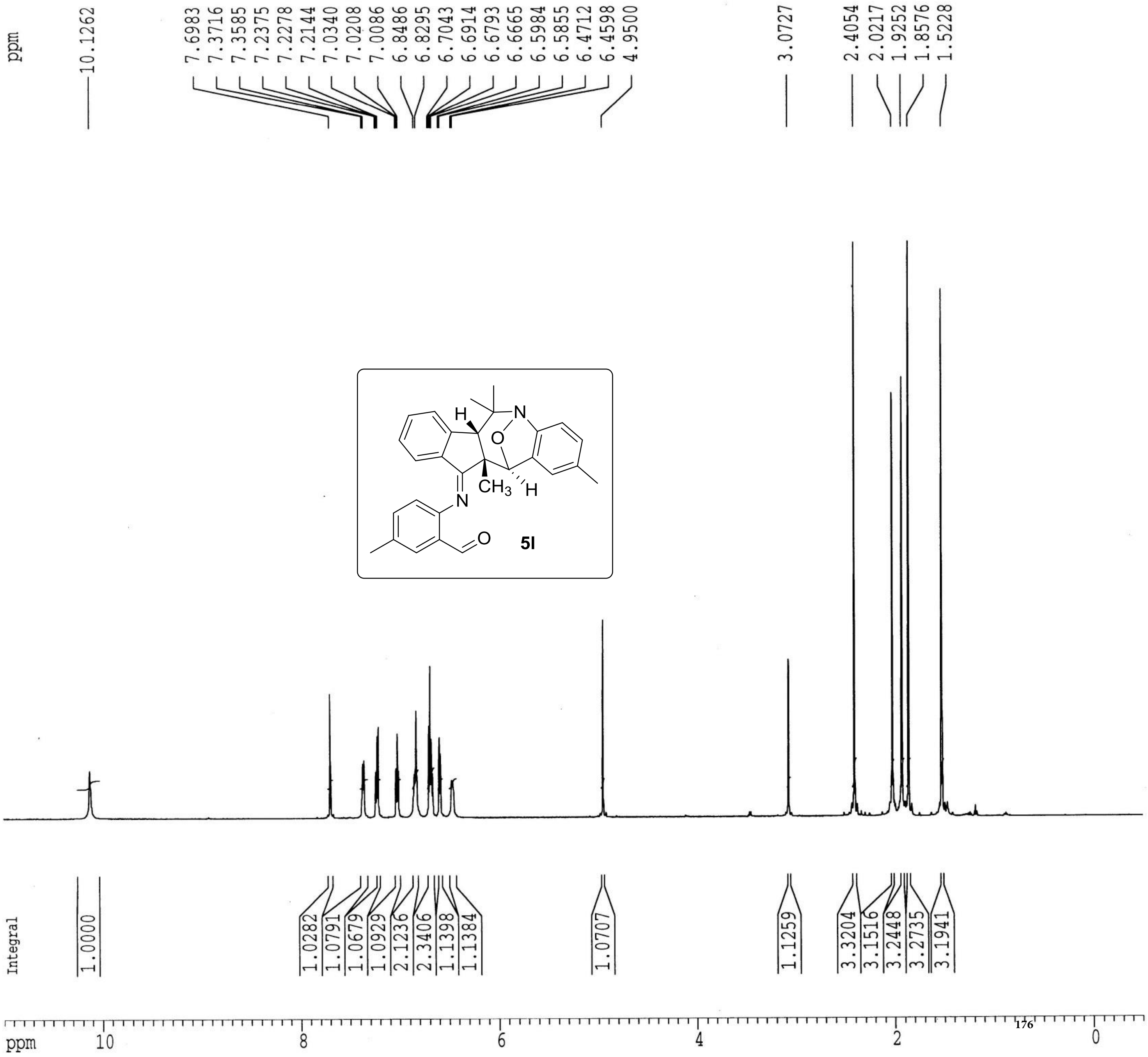

Current Data Parameters
NAME RKS-5-157-HT
EXPNO 2
PROCNO 1

F2 - Acquisition Parameters
Date_ 20171016
Time 7.19
INSTRUM spect
PROBHD 5 mm QNP 1H/1
PULPROG zgpg
TD 32768
SOLVENT CDCl3
NS 400
DS 0
SWH 45045.047 Hz
FIDRES 1.374666 Hz
AQ 0.3637748 sec
RG 4096
DW 11.100 usec
DE 6.50 usec
TE 319.8 K
D1 3.50000000 sec
d11 0.03000000 sec
DELTA 3.40000010 sec
MCREST 0.00000000 sec
MCWRK 0.01500000 sec

======== CHANNEL f1 ========
NUC1 13C
P1 4.80 usec
PL1 0.00 dB
SFO1 150.4828468 MHz

======== CHANNEL f2 ========
CPDPRG2 waltz16
NUC2 1H
PCPD2 92.00 usec
PL2 120.00 dB
PL12 5.50 dB
PL13 9.00 dB
SFO2 598.4029920 MHz

F2 - Processing parameters
SI 65536
SF 150.4677856 MHz
WDW EM
SSB 0
LB 3.00 Hz
GB 0
PC 1.00

1D NMR plot parameters
CX 20.00 cm
CY 4.00 cm
F1P 200.000 ppm
F1 30093.56 Hz
F2P 0.000 ppm
F2 0.00 Hz
PPMCM 10.00000 ppm/cm
HZCM 1504.67786 Hz/cm

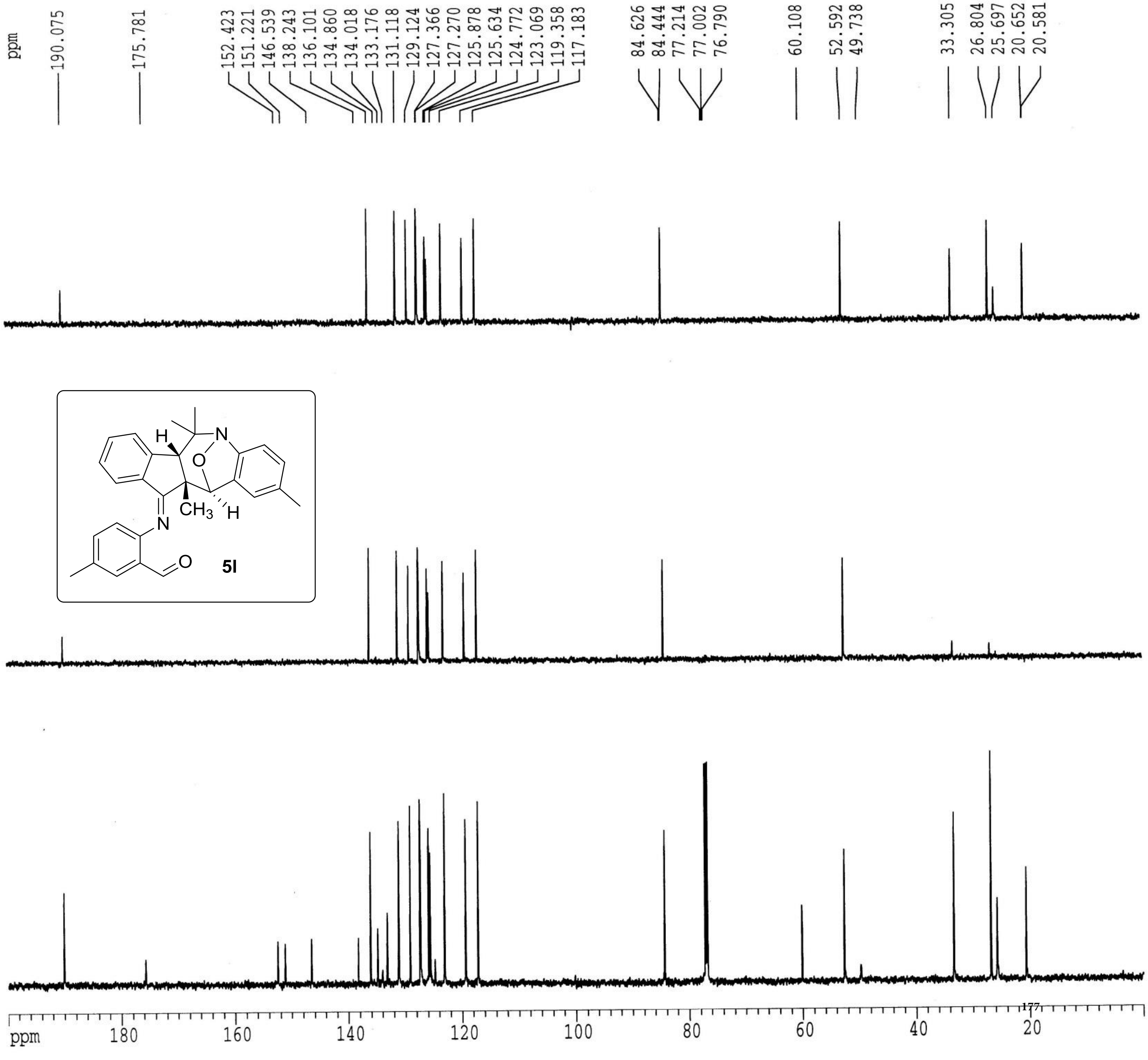

5l

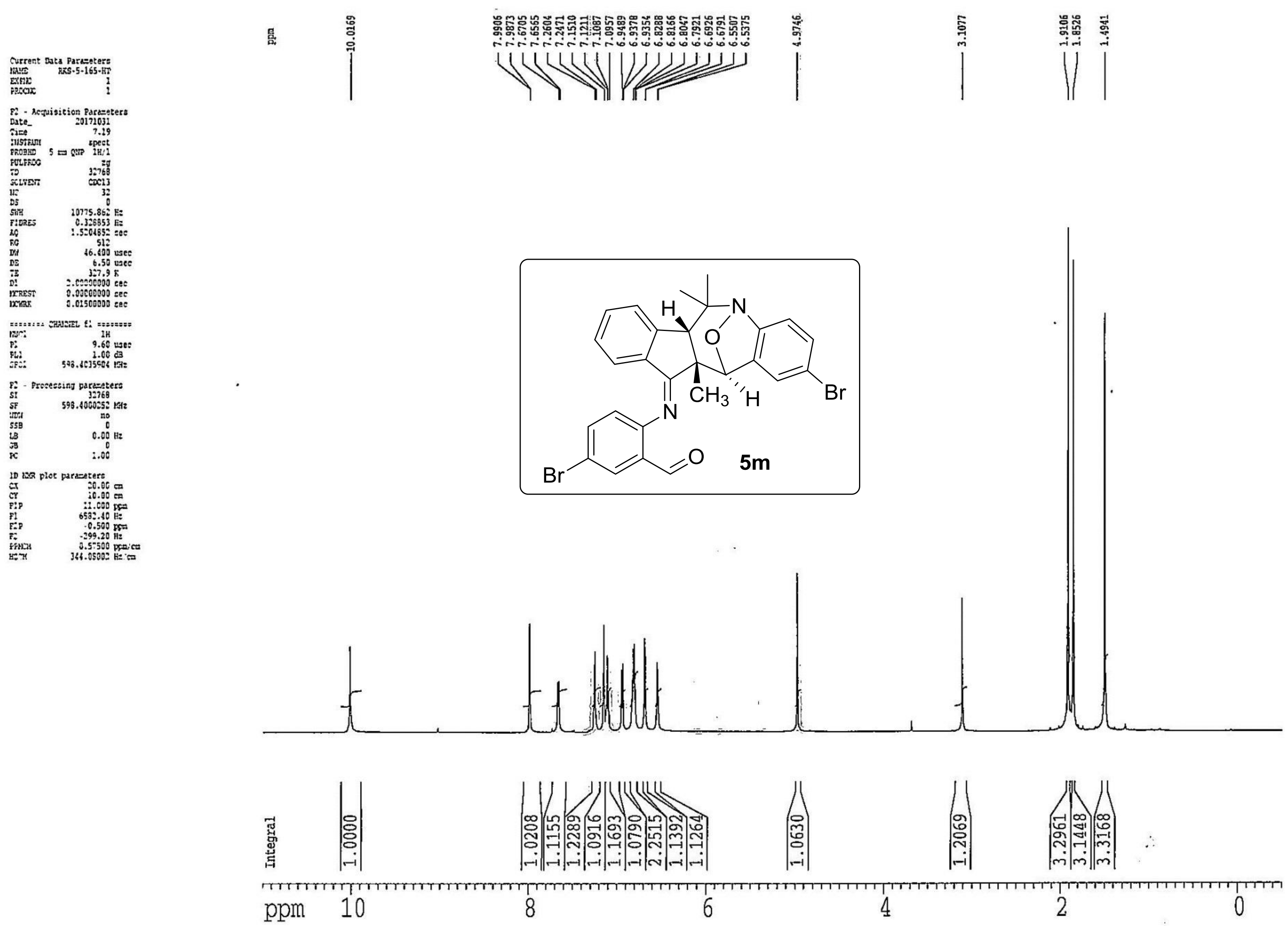
Current Data Parameters
NAME RKS-5-165-HT
EXPNO 1
PROCNO 1
F2 - Acquisition Parameters
Date_ 20171031
Time 7.19
INSTRUM spect
PROBHD 5 mm QNP 1H/1
PULPROG zg
TD 32768
SOLVENT CDCl3
NS 32
DS 0
SWH 10775.862 Hz
FIDRES 0.328853 Hz
AQ 1.5204852 sec
RG 512
DW 46.400 usec
DE 6.50 usec
TE 327.9 K
D1 1.00000000 sec
MCREST 0.00000000 sec
MCWRK 0.01500000 sec
======== CHANNEL f1 ========
NUC1 1H
P1 9.60 usec
PL1 1.00 dB
SFO1 598.4035904 MHz
F2 - Processing parameters
SI 32768
SF 598.4000252 MHz
WDW no
SSB 0
LB 0.00 Hz
GB 0
PC 1.00
1D NMR plot parameters
CX 20.00 cm
CY 10.00 cm
F1P 11.000 ppm
F1 6582.40 Hz
F2P -0.500 ppm
F2 -299.20 Hz
PPMCM 0.57500 ppm/cm
HZCM 344.05002 Hz/cm
ppm
10.0169
7.9906
7.9873
7.6705
7.6565
7.2604
7.2471
7.1510
7.1211
7.1087
7.0957
6.9489
6.9378
6.9354
6.8288
6.8166
6.8047
6.7921
6.6926
6.6791
6.5507
6.5375
4.9746
3.1077
1.9106
1.8526
1.4941
H
N
O
CH3
H
Br
N
Br
O
5m
Integral
1.0000
1.0208
1.1155
1.2289
1.0916
1.1693
1.0790
2.2515
1.1392
1.1264
1.0630
1.2069
3.2961
3.1448
3.3168
ppm
10
8
6
4
2
0

Current Data Parameters
NAME RKS-5-165-HT
EXPNO 2
PROCNO 1

F2 - Acquisition Parameters
Date_ 20171031
Time 6.09
INSTRUM spect
PROBHD 5 mm QNP 1H/1
PULPROG zgpg
TD 32768
SOLVENT CDCl3
NS 277
DS 0
SWH 45045.047 Hz
FIDRES 1.374666 Hz
AQ 0.3637748 sec
RG 4096
DW 11.100 usec
DE 6.50 usec
TE 322.6 K
D1 3.50000000 sec
d11 0.03000000 sec
DELTA 3.40000010 sec
MCREST 0.00000000 sec
MCWRK 0.01500000 sec

======== CHANNEL f1 ========
NUC1 13C
P1 4.80 usec
PL1 0.00 dB
SFO1 150.4843515 MHz

======== CHANNEL f2 ========
CPDPRG2 waltz16
NUC2 1H
PCPD2 92.00 usec
PL2 120.00 dB
PL12 5.50 dB
PL13 9.00 dB
SFO2 598.4029920 MHz

F2 - Processing parameters
SI 65536
SF 150.4677864 MHz
WDW EM
SSB 0
LB 3.00 Hz
GB 0
PC 1.00

1D NMR plot parameters
CX 20.00 cm
CY 4.00 cm
F1P 200.000 ppm
F1 30093.56 Hz
F2P 0.000 ppm
F2 0.00 Hz
PPMCM 10.00000 ppm/cm
HZCM 1504.67786 Hz/cm

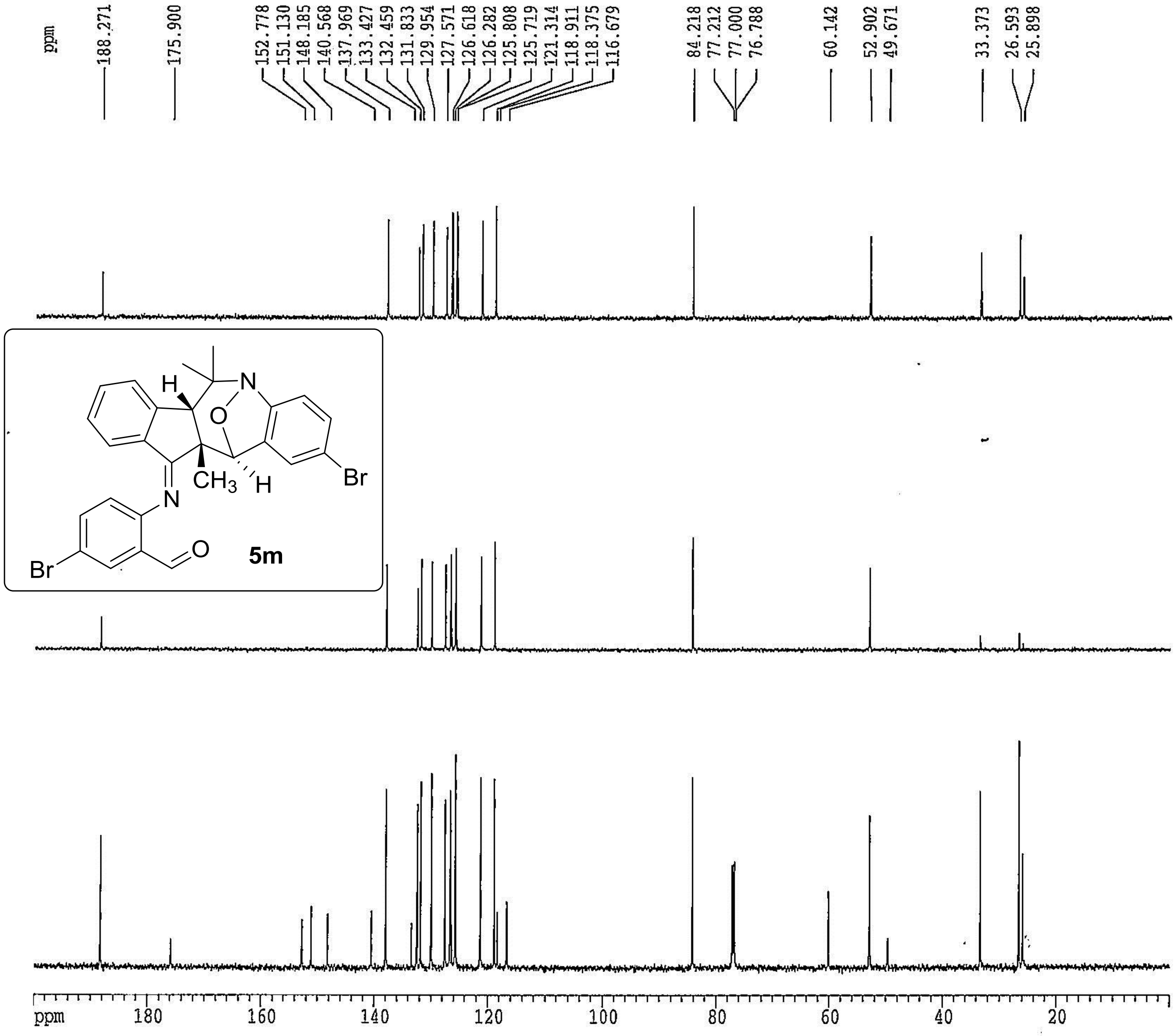

Current Data Parameters
NAME RKS-5-158-HT
EXPNO 100
PROCNO 1

F2 - Acquisition Parameters
Date_ 20171023
Time 10.39
INSTRUM spect
PROBHD 5 mm QNP 1H/1
PULPROG zg
TD 32768
SOLVENT CDCl3
NS 32
DS 0
SWH 8375.209 Hz
FIDRES 0.255591 Hz
AQ 1.9562995 sec
RG 512
DW 59.700 usec
DE 6.50 usec
TE 327.1 K
D1 1.00000000 sec
MCREST 0.00000000 sec
MCWRK 0.01500000 sec

======== CHANNEL f1 ========
NUC1 1H
P1 10.00 usec
PL1 -1.00 dB
SFO1 598.4035904 MHz

F2 - Processing parameters
SI 32768
SF 598.4000235 MHz
WDW no
SSB 0
LB 0.00 Hz
GB 0
PC 1.00

1D NMR plot parameters
CX 20.00 cm
CY 10.00 cm
F1P 11.000 ppm
F1 6582.40 Hz
F2P -0.500 ppm
F2 -299.20 Hz
PPMCM 0.57500 ppm/cm
HZCM 344.08002 Hz/cm

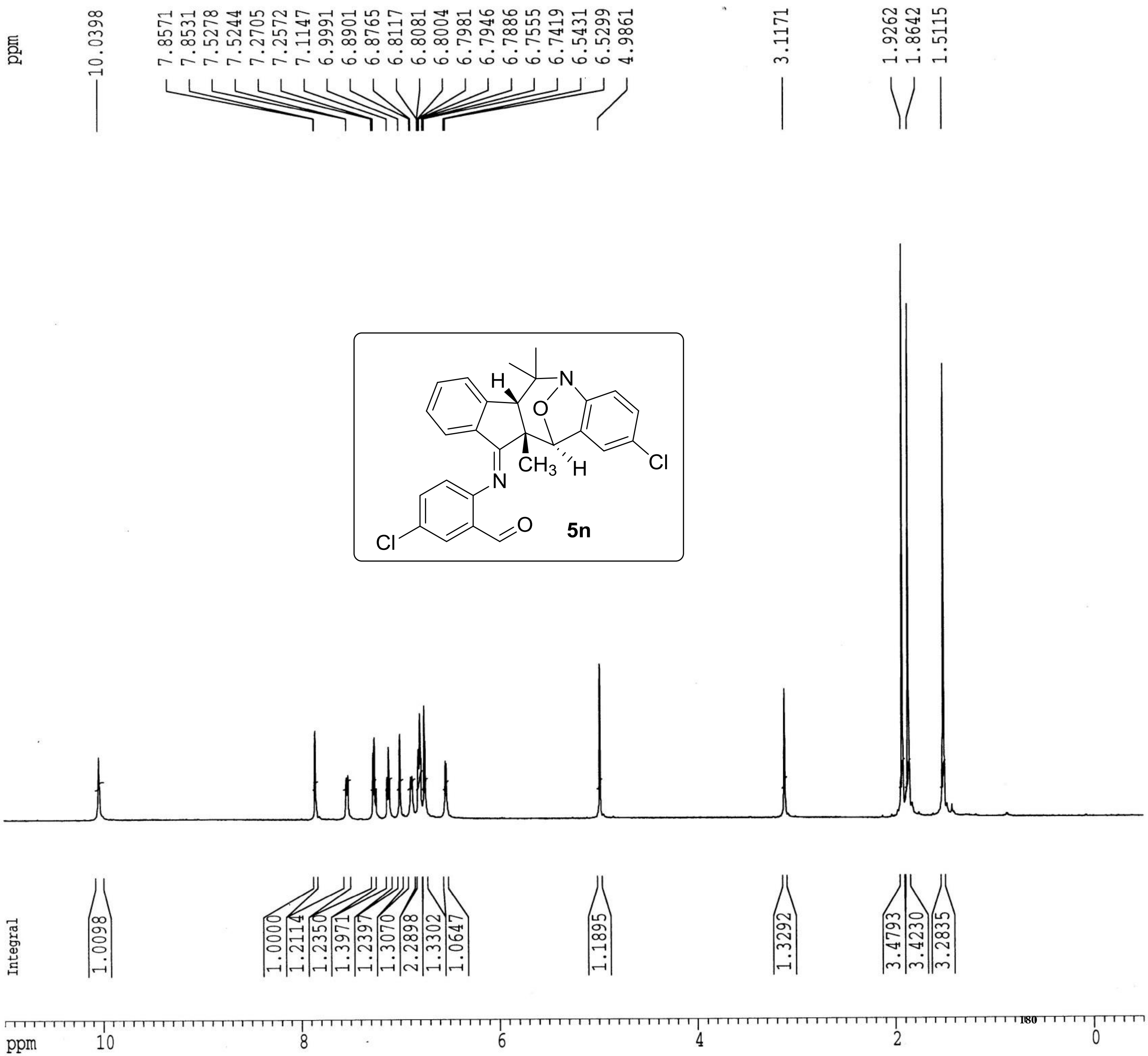

Current Data Parameters
NAME RKS-5-158-HT
EXPNO 2
PROCNO 1

F2 - Acquisition Parameters
Date_ 20171023
Time 9.44
INSTRUM spect
PROBHD 5 mm QNP 1H/1
PULPROG zgpg
TD 32768
SOLVENT CDCl3
NS 500
DS 0
SWH 45045.047 Hz
FIDRES 1.374666 Hz
AQ 0.3637748 sec
RG 4096
DW 11.100 usec
DE 6.50 usec
TE 323.5 K
D1 3.50000000 sec
d11 0.03000000 sec
DELTA 3.40000010 sec
MCREST 0.00000000 sec
MCWRK 0.01500000 sec

======== CHANNEL f1 ========
NUC1 13C
P1 4.80 usec
PL1 0.00 dB
SFO1 150.4828468 MHz

======== CHANNEL f2 ========
CPDPRG2 waltz16
NUC2 1H
PCPD2 92.00 usec
PL2 120.00 dB
PL12 5.50 dB
PL13 9.00 dB
SFO2 598.4029920 MHz

F2 - Processing parameters
SI 65536
SF 150.4677815 MHz
WDW EM
SSB 0
LB 3.00 Hz
GB 0
PC 1.00

1D NMR plot parameters
CX 20.00 cm
CY 3.00 cm
F1P 200.000 ppm
F1 30093.56 Hz
F2P 0.000 ppm
F2 0.00 Hz
PPMCM 10.00000 ppm/cm
HZCM 1504.67786 Hz/cm

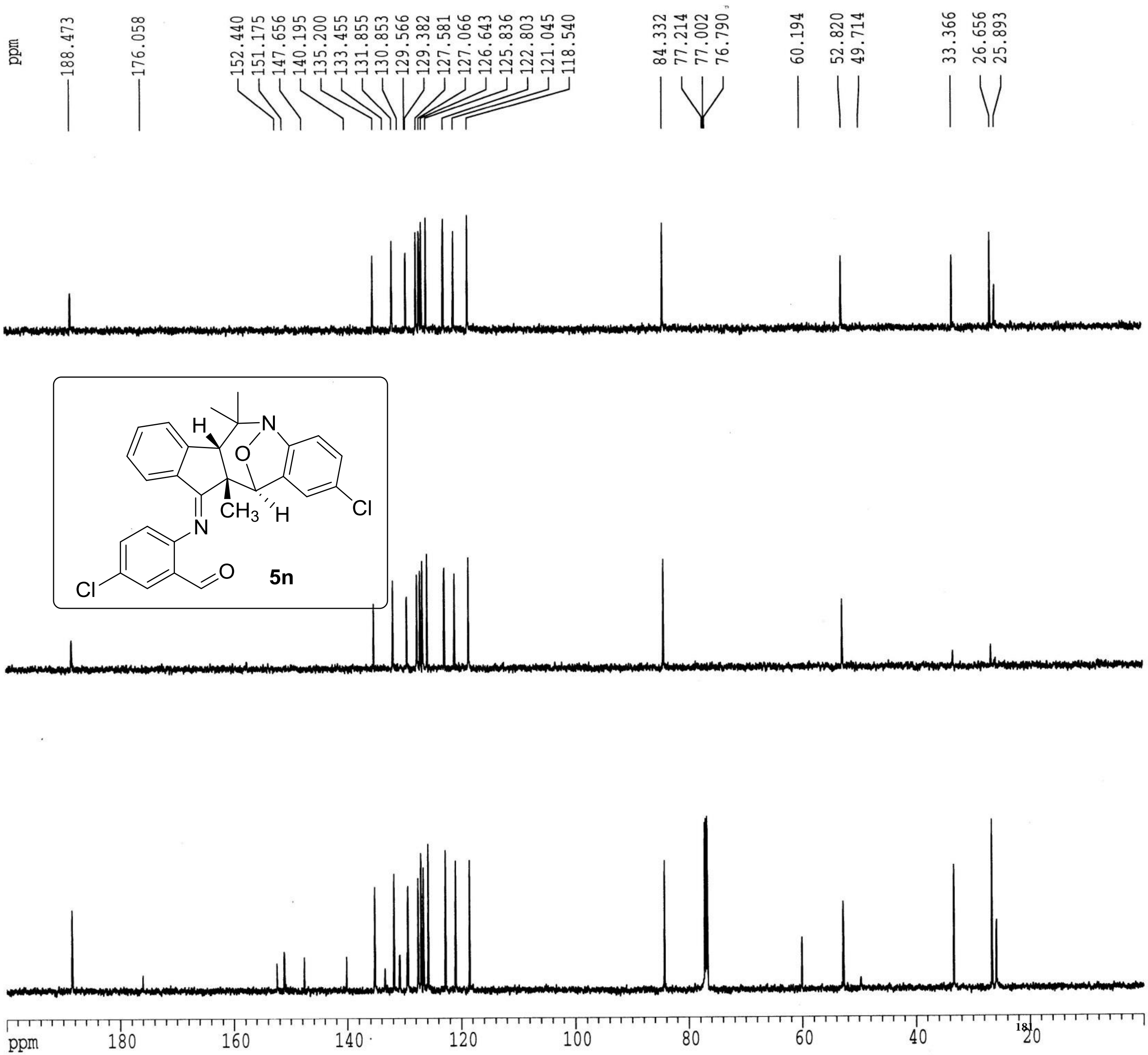

BRUKER

Current Data Parameters
NAME MS-04-32-2-HT
EXPNO 1
PROCNO 1

F2 - Acquisition Parameters
Date_ 20171122
Time 1.26
INSTRUM spect
PROBHD 5 mm QNP 1H/1
PULPROG zg
TD 32768
SOLVENT CDCl3
NS 32
DS 0
SWH 9541.984 Hz
FIDRES 0.291198 Hz
AQ 1.7170932 sec
RG 512
DW 52.400 usec
DE 6.50 usec
TE 313.4 K
D1 2.00000000 sec
MCREST 0 sec
MCWRK 0.01500000 sec

======== CHANNEL f1 ========
NUC1 1H
P1 10.00 usec
PL1 -1.00 dB
SFO1 598.3029915 MHz

F2 - Processing parameters
SI 32768
SF 598.3000284 MHz
WDW no
SSB 0
LB 0 Hz
GB 0
PC 1.00

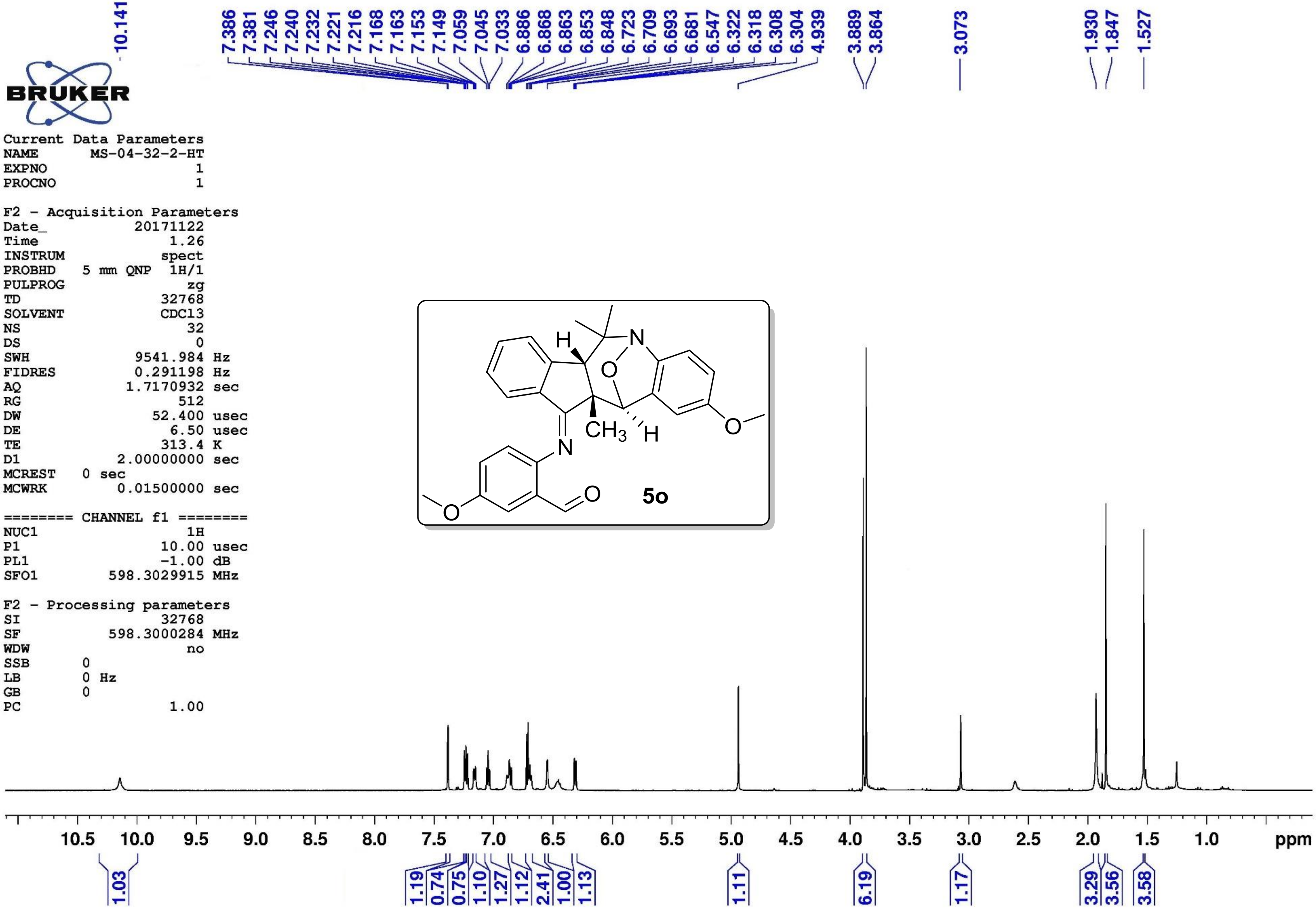

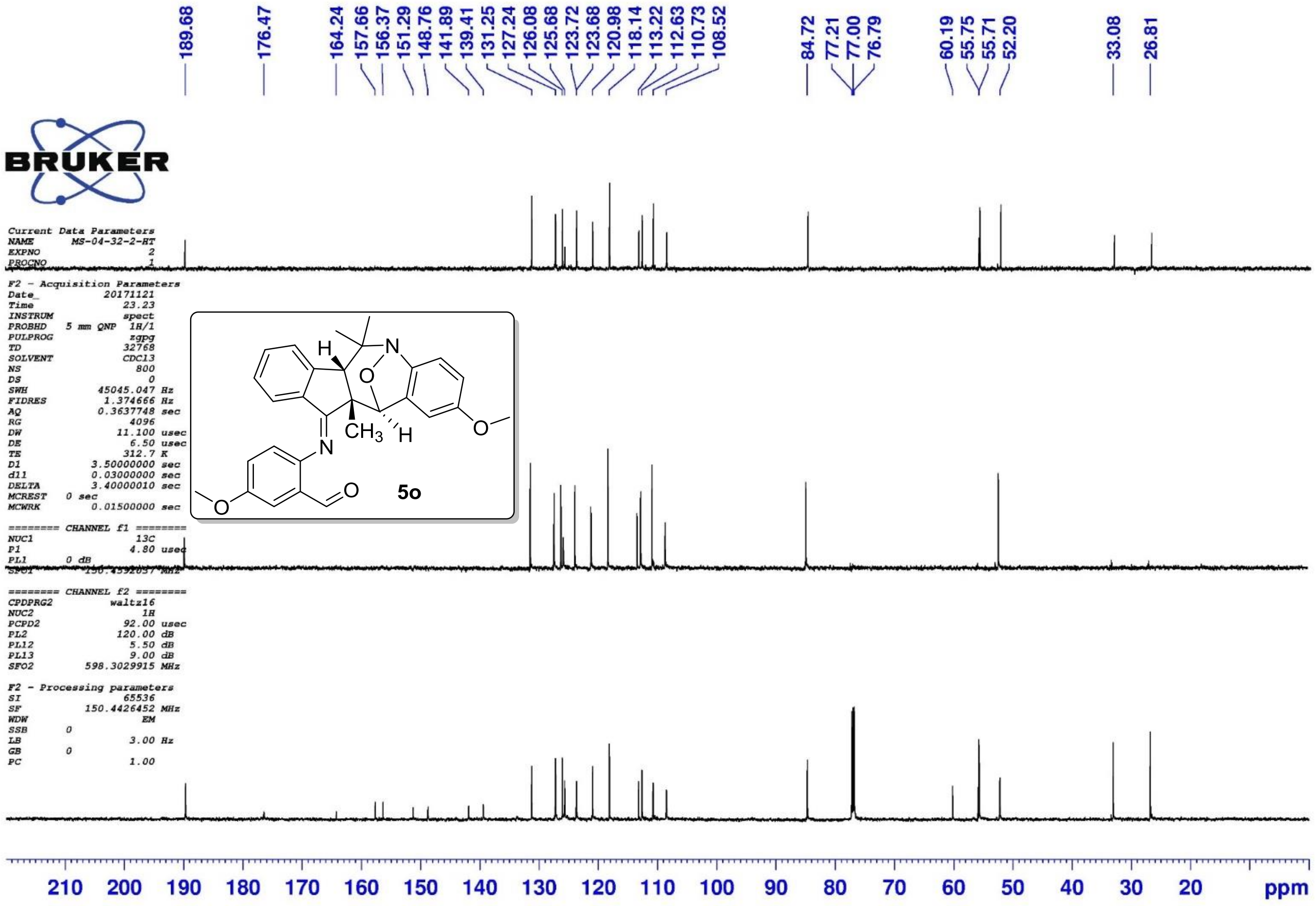

189.68
176.47
164.24
157.66
156.37
151.29
148.76
141.89
139.41
131.25
127.24
126.08
125.68
123.72
123.68
120.98
118.14
113.22
112.63
110.73
108.52
84.72
77.21
77.00
76.79
60.19
55.75
55.71
52.20
33.08
26.81
BRUKER
Current Data Parameters
NAME MS-04-32-2-HT
EXPNO 2
PROCNO 1
F2 - Acquisition Parameters
Date_ 20171121
Time 23.23
INSTRUM spect
PROBHD 5 mm QNP 1H/1
PULPROG zgpg
TD 32768
SOLVENT CDCl3
NS 800
DS 0
SWH 45045.047 Hz
FIDRES 1.374666 Hz
AQ 0.3637748 sec
RG 4096
DW 11.100 usec
DE 6.50 usec
TE 312.7 K
D1 3.50000000 sec
d11 0.03000000 sec
DELTA 3.40000010 sec
MCREST 0 sec
MCWRK 0.01500000 sec
======== CHANNEL f1 ========
NUC1 13C
P1 4.80 usec
PL1 0 dB
SFO1 150.4592037 MHz
======== CHANNEL f2 ========
CPDPRG2 waltz16
NUC2 1H
PCPD2 92.00 usec
PL2 120.00 dB
PL12 5.50 dB
PL13 9.00 dB
SFO2 598.3029915 MHz
F2 - Processing parameters
SI 65536
SF 150.4426452 MHz
WDW EM
SSB 0
LB 3.00 Hz
GB 0
PC 1.00
H
N
O
CH3
H
O
N
O
O
5o
210 200 190 180 170 160 150 140 130 120 110 100 90 80 70 60 50 40 30 20 ppm

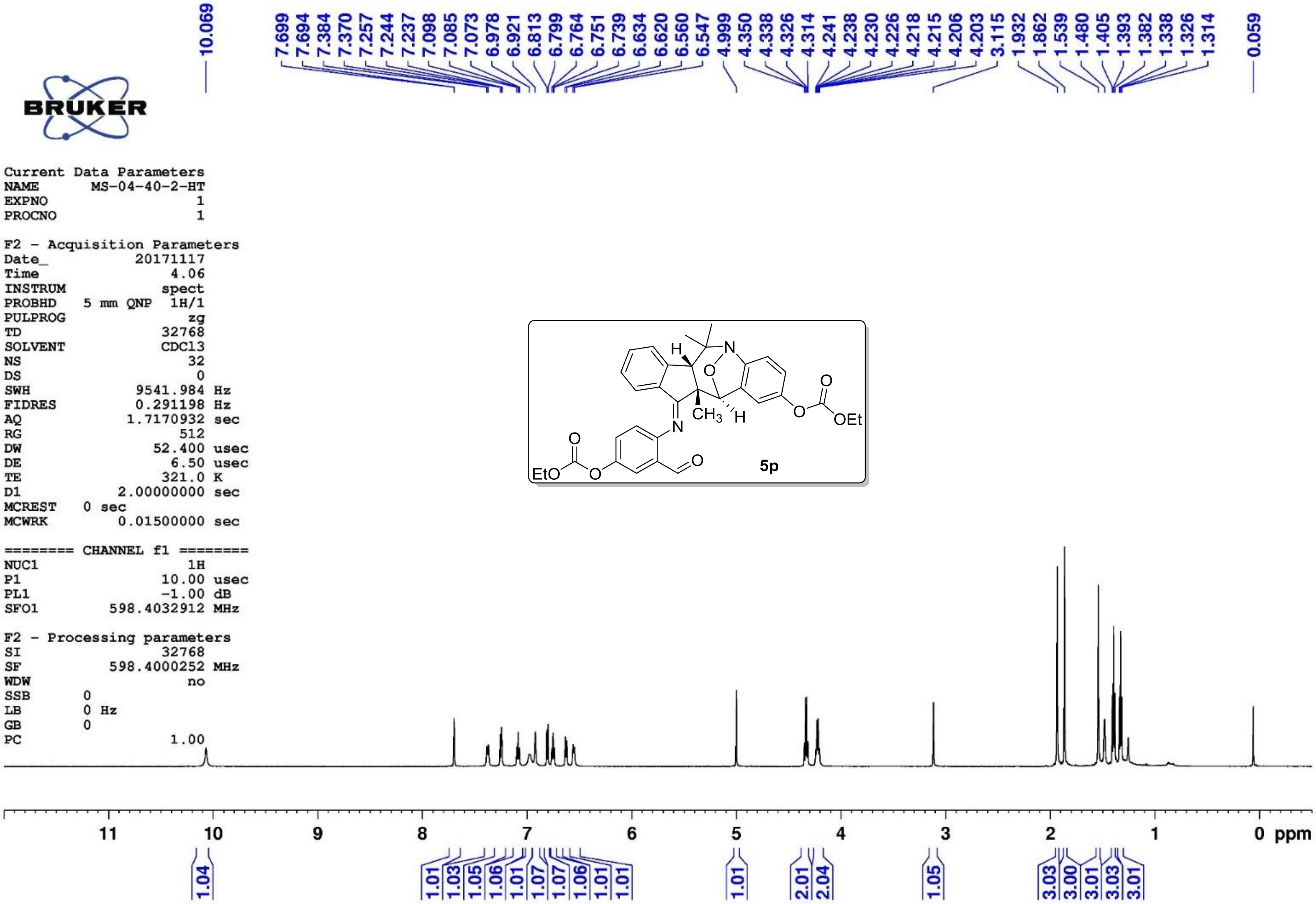
10.069
7.699
7.694
7.384
7.370
7.257
7.244
7.237
7.098
7.085
7.073
6.978
6.921
6.813
6.799
6.764
6.751
6.739
6.634
6.620
6.560
6.547
4.999
4.350
4.338
4.326
4.314
4.241
4.238
4.230
4.226
4.218
4.215
4.206
4.203
3.115
1.932
1.862
1.539
1.480
1.405
1.393
1.382
1.338
1.326
1.314
0.059
BRUKER
Current Data Parameters
NAME MS-04-40-2-HT
EXPNO 1
PROCNO 1
F2 - Acquisition Parameters
Date_ 20171117
Time 4.06
INSTRUM spect
PROBHD 5 mm QNP 1H/1
PULPROG zg
TD 32768
SOLVENT CDCl3
NS 32
DS 0
SWH 9541.984 Hz
FIDRES 0.291198 Hz
AQ 1.7170932 sec
RG 512
DW 52.400 usec
DE 6.50 usec
TE 321.0 K
D1 2.00000000 sec
MCREST 0 sec
MCWRK 0.01500000 sec
======== CHANNEL f1 ========
NUC1 1H
P1 10.00 usec
PL1 -1.00 dB
SFO1 598.4032912 MHz
F2 - Processing parameters
SI 32768
SF 598.4000252 MHz
WDW no
SSB 0
LB 0 Hz
GB 0
PC 1.00
11 10 9 8 7 6 5 4 3 2 1 0 ppm
1.04
1.01
1.03
1.05
1.06
1.01
1.07
1.07
1.06
1.01
1.01
1.01
2.01
2.04
1.05
3.03
3.00
3.01
3.03
3.01
H
N
O
O
CH3
H
O
OEt
N
O
EtO
O
O

5p

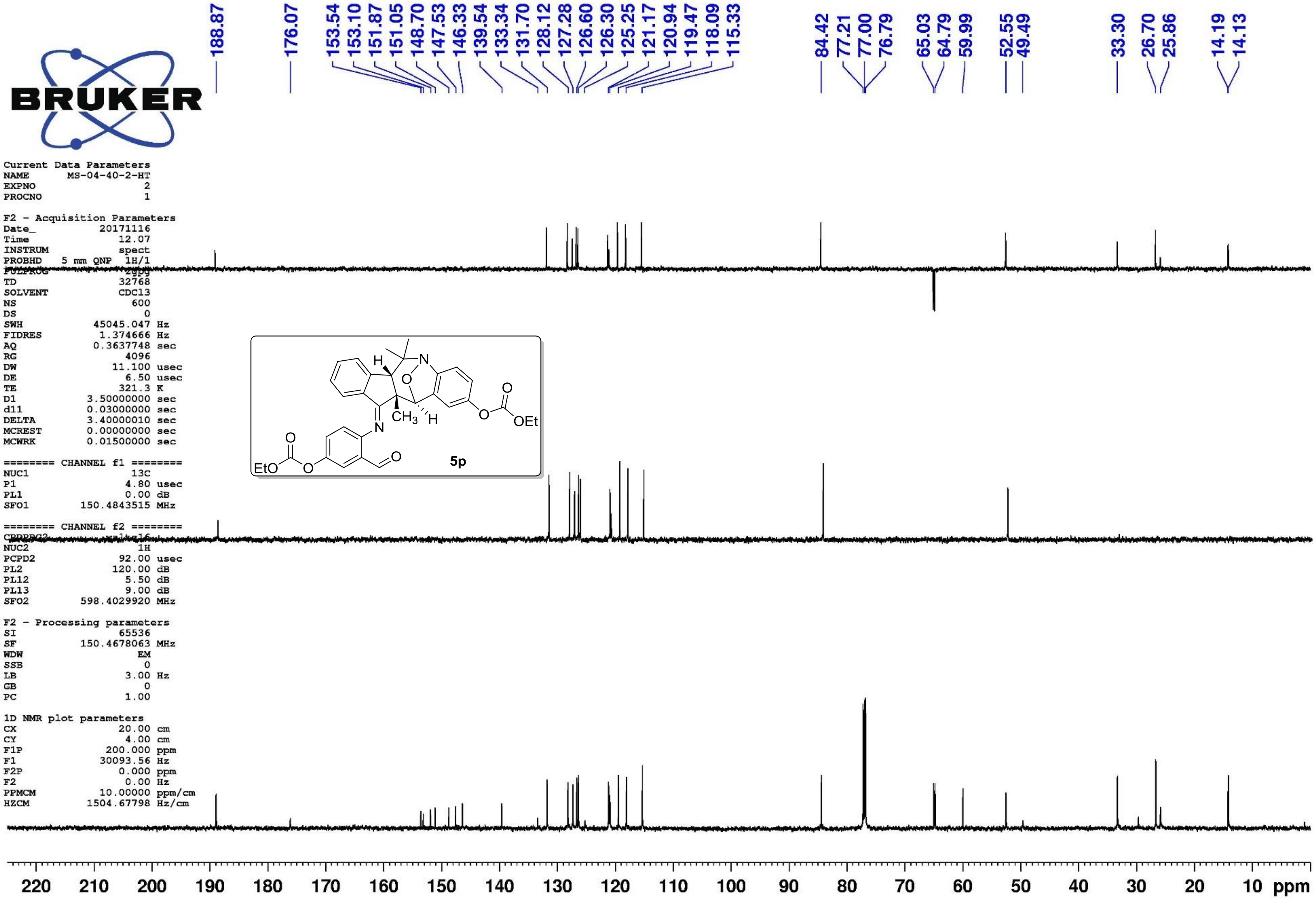
188.87
176.07
153.54
153.10
151.87
151.05
148.70
147.53
146.33
139.54
133.34
131.70
128.12
127.28
126.60
126.30
125.25
121.17
120.94
119.47
118.09
115.33
84.42
77.21
77.00
76.79
65.03
64.79
59.99
52.55
49.49
33.30
26.70
25.86
14.19
14.13
BRUKER
Current Data Parameters
NAME MS-04-40-2-HT
EXPNO 2
PROCNO 1
F2 - Acquisition Parameters
Date_ 20171116
Time 12.07
INSTRUM spect
PROBHD 5 mm QNP 1H/1
PULPROG zgpg
TD 32768
SOLVENT CDCl3
NS 600
DS 0
SWH 45045.047 Hz
FIDRES 1.374666 Hz
AQ 0.3637748 sec
RG 4096
DW 11.100 usec
DE 6.50 usec
TE 321.3 K
D1 3.50000000 sec
d11 0.03000000 sec
DELTA 3.40000010 sec
MCREST 0.00000000 sec
MCWRK 0.01500000 sec
======== CHANNEL f1 ========
NUC1 13C
P1 4.80 usec
PL1 0.00 dB
SFO1 150.4843515 MHz
======== CHANNEL f2 ========
CPDPRG2 waltz16
NUC2 1H
PCPD2 92.00 usec
PL2 120.00 dB
PL12 5.50 dB
PL13 9.00 dB
SFO2 598.4029920 MHz
F2 - Processing parameters
SI 65536
SF 150.4678063 MHz
WDW EM
SSB 0
LB 3.00 Hz
GB 0
PC 1.00
1D NMR plot parameters
CX 20.00 cm
CY 4.00 cm
F1P 200.000 ppm
F1 30093.56 Hz
F2P 0.000 ppm
F2 0.00 Hz
PPMCM 10.00000 ppm/cm
HZCM 1504.67798 Hz/cm
220 210 200 190 180 170 160 150 140 130 120 110 100 90 80 70 60 50 40 30 20 10 ppm
H
N
O
CH3
H
N
O
OEt
O
EtO
O
O

5p

```
Current Data Parameters
NAME          MS-04-38-HT
EXPNO                   1
PROCNO                  1

F2 - Acquisition Parameters
Date_            20171111
Time                 3.09
INSTRUM             spect
PROBHD   5 mm QNP  1H/1
PULPROG                zg
TD                  32768
SOLVENT             CDCl3
NS                     32
DS                      0
SWH              8389.262 Hz
FIDRES           0.256020 Hz
AQ              1.9530228 sec
RG                    512
DW                 59.600 usec
DE                   6.50 usec
TE                  320.5 K
D1             2.00000000 sec
MCREST    0 sec
MCWRK          0.01500000 sec

======== CHANNEL f1 ========
NUC1                   1H
P1                   9.60 usec
PL1                  1.00 dB
SFO1          598.4032912 MHz

F2 - Processing parameters
SI                  32768
SF            598.4000252 MHz
WDW                    no
SSB       0
LB        0 Hz
GB        0
PC                   1.00
```

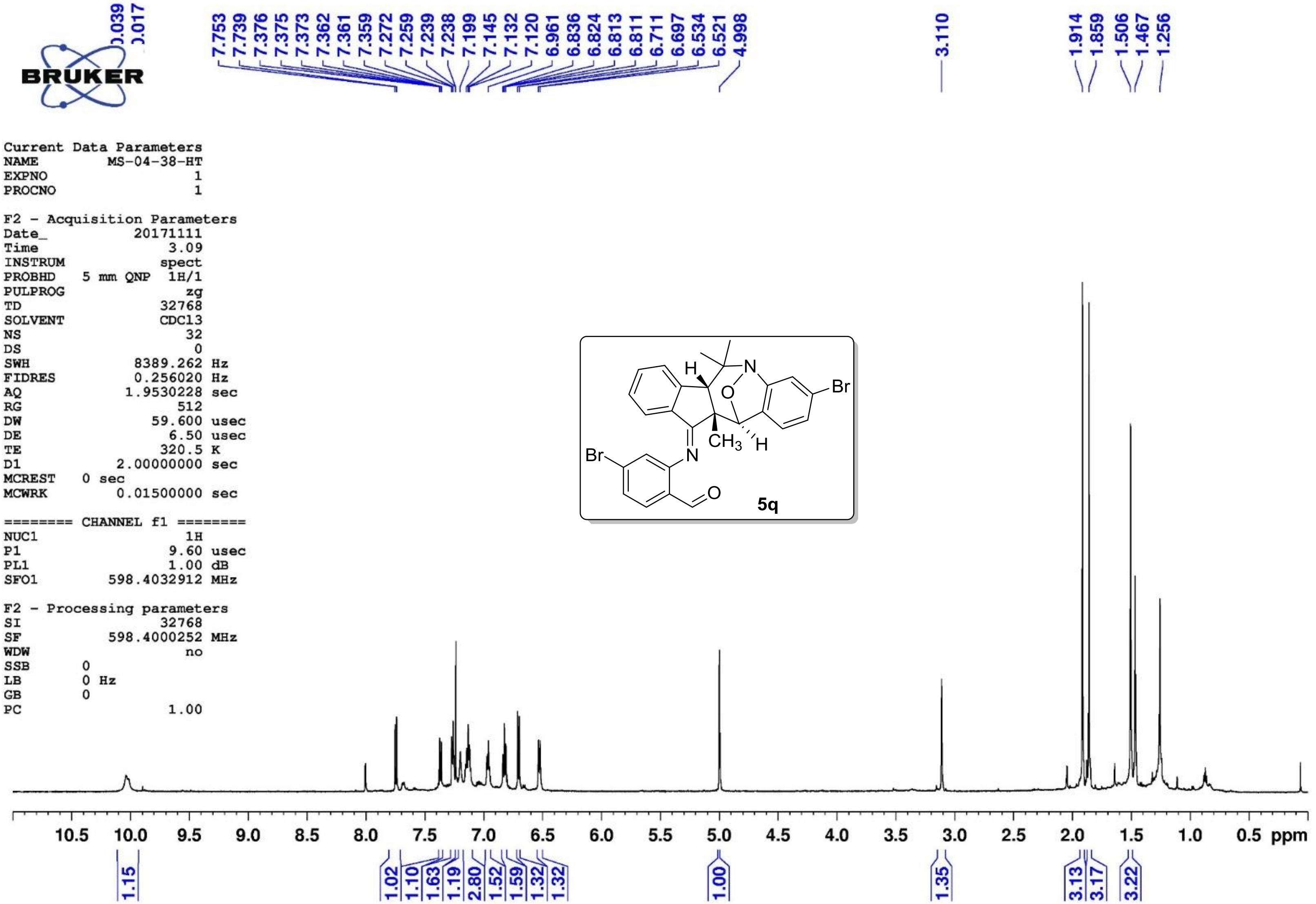

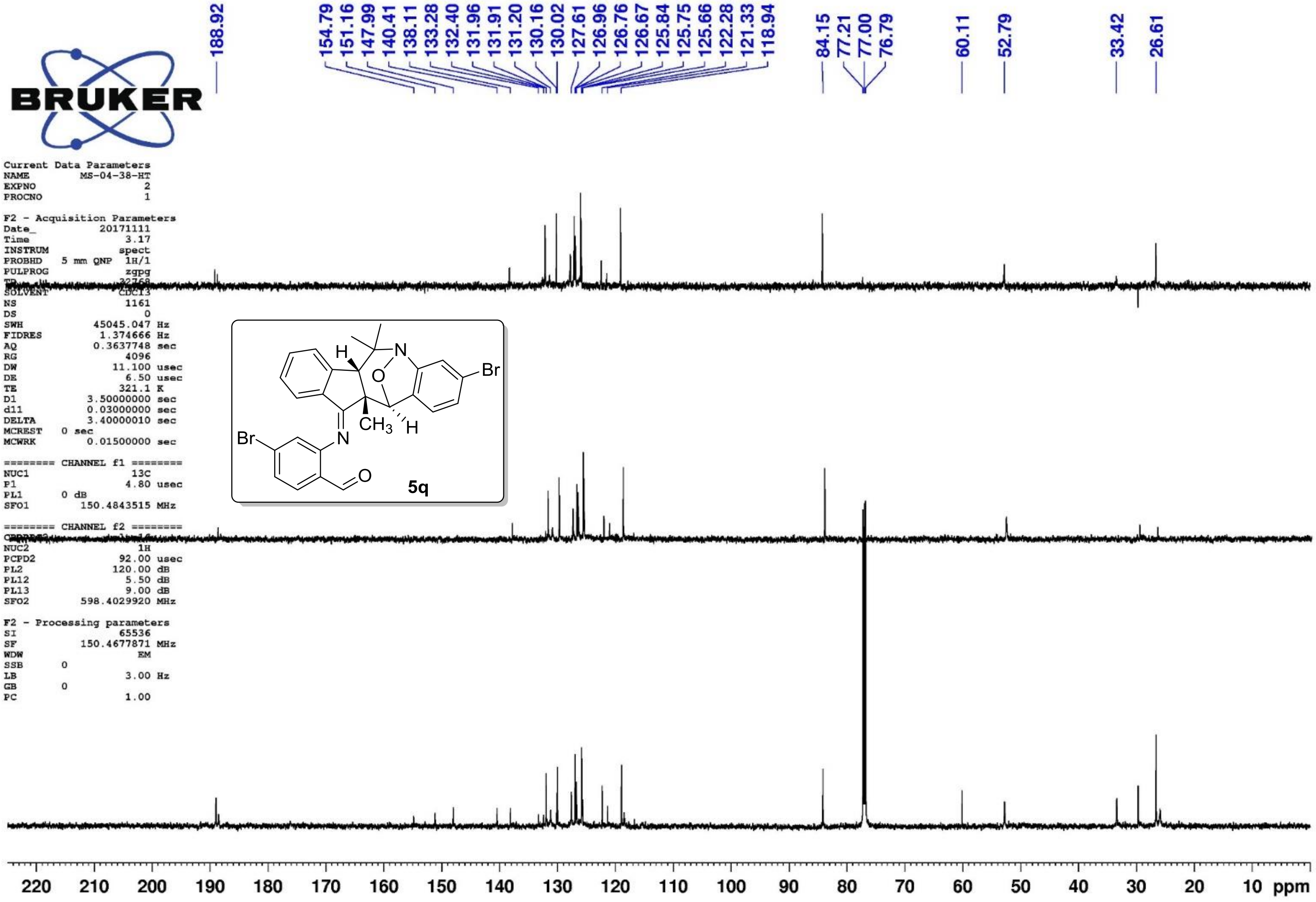
188.92
154.79
151.16
147.99
140.41
138.11
133.28
132.40
131.96
131.91
131.20
130.16
130.02
127.61
126.96
126.76
126.67
125.84
125.75
125.66
122.28
121.33
118.94
84.15
77.21
77.00
76.79
60.11
52.79
33.42
26.61
BRUKER
Current Data Parameters
NAME MS-04-38-HT
EXPNO 2
PROCNO 1
F2 - Acquisition Parameters
Date_ 20171111
Time 3.17
INSTRUM spect
PROBHD 5 mm QNP 1H/1
PULPROG zgpg
SOLVENT CDCl3
NS 1161
DS 0
SWH 45045.047 Hz
FIDRES 1.374666 Hz
AQ 0.3637748 sec
RG 4096
DW 11.100 usec
DE 6.50 usec
TE 321.1 K
D1 3.50000000 sec
d11 0.03000000 sec
DELTA 3.40000010 sec
MCREST 0 sec
MCWRK 0.01500000 sec
======== CHANNEL f1 ========
NUC1 13C
P1 4.80 usec
PL1 0 dB
SFO1 150.4843515 MHz
======== CHANNEL f2 ========
NUC2 1H
PCPD2 92.00 usec
PL2 120.00 dB
PL12 5.50 dB
PL13 9.00 dB
SFO2 598.4029920 MHz
F2 - Processing parameters
SI 65536
SF 150.4677871 MHz
WDW EM
SSB 0
LB 3.00 Hz
GB 0
PC 1.00
H
N
O
Br
CH3
H
Br
N
O
5q
220
210
200
190
180
170
160
150
140
130
120
110
100
90
80
70
60
50
40
30
20
10
ppm

Current Data Parameters
NAME MS-04-53-HT
EXPNO 1
PROCNO 1

F2 - Acquisition Parameters
Date_ 20171212
Time 5.18
INSTRUM spect
PROBHD 5 mm QNP 1H/1
PULPROG zg
TD 32768
SOLVENT CDCl3
NS 32
DS 0
SWH 9541.984 Hz
FIDRES 0.291198 Hz
AQ 1.7170932 sec
RG 1024
DW 52.400 usec
DE 6.50 usec
TE 313.5 K
D1 2.00000000 sec
MCREST 0 sec
MCWRK 0.01500000 sec

======== CHANNEL f1 ========
NUC1 1H
P1 10.00 usec
PL1 -1.00 dB
SFO1 598.3029915 MHz

F2 - Processing parameters
SI 32768
SF 598.3000302 MHz
WDW no
SSB 0
LB 0 Hz
GB 0
PC 2.00

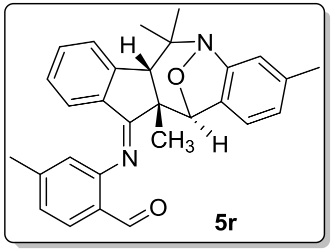

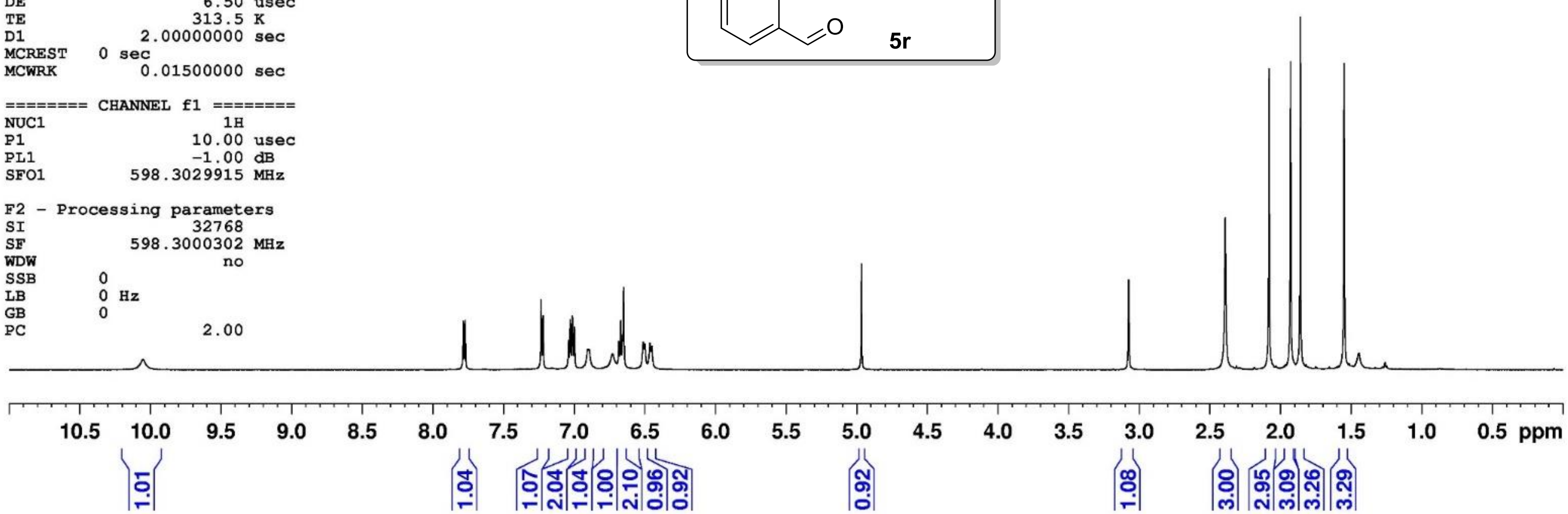

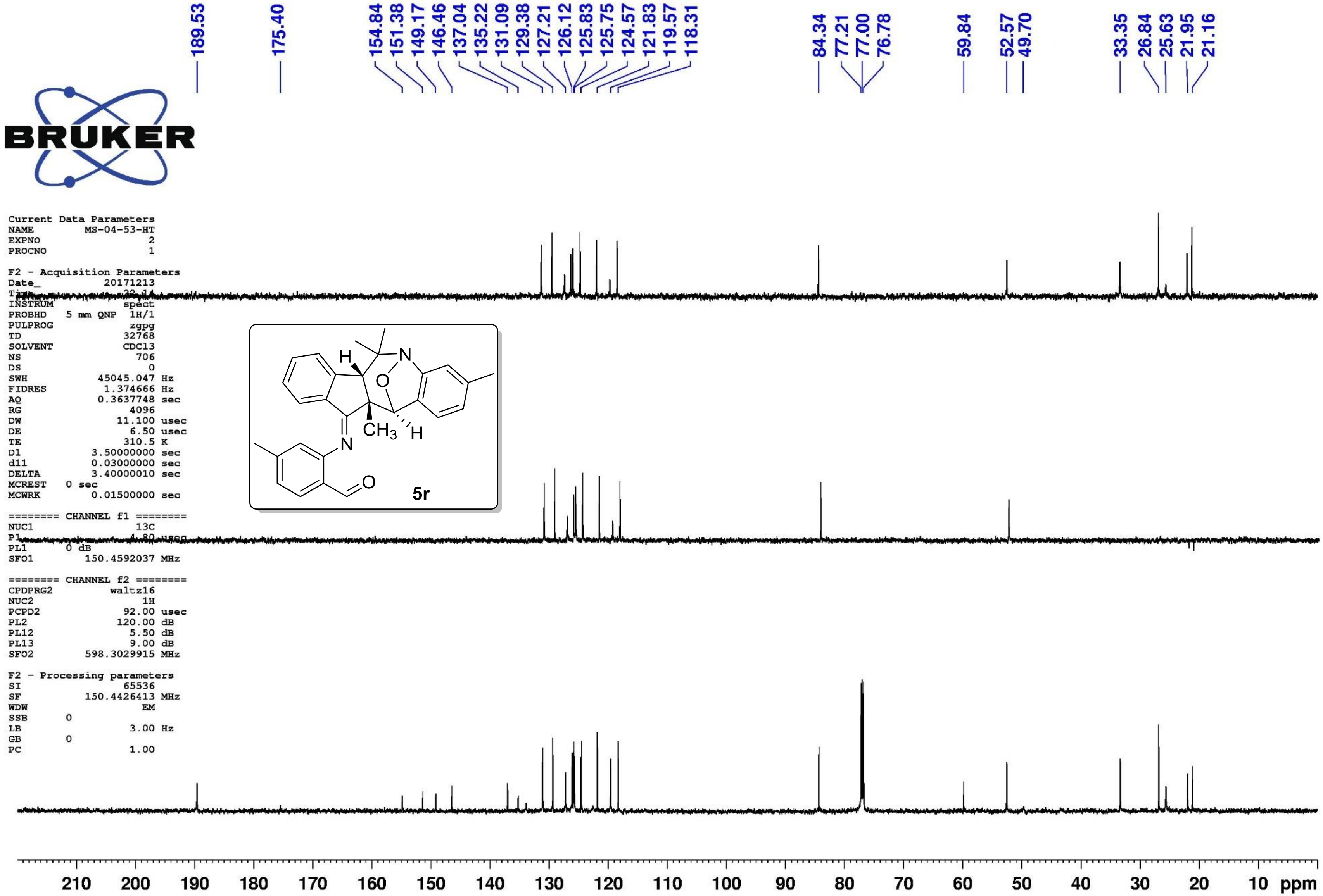

189.53
175.40
154.84
151.38
149.17
146.46
137.04
135.22
131.09
129.38
127.21
126.12
125.83
125.75
124.57
121.83
119.57
118.31
84.34
77.21
77.00
76.78
59.84
52.57
49.70
33.35
26.84
25.63
21.95
21.16
BRUKER
Current Data Parameters
NAME MS-04-53-HT
EXPNO 2
PROCNO 1
F2 - Acquisition Parameters
Date_ 20171213
INSTRUM spect
PROBHD 5 mm QNP 1H/1
PULPROG zgpg
TD 32768
SOLVENT CDCl3
NS 706
DS 0
SWH 45045.047 Hz
FIDRES 1.374666 Hz
AQ 0.3637748 sec
RG 4096
DW 11.100 usec
DE 6.50 usec
TE 310.5 K
D1 3.50000000 sec
d11 0.03000000 sec
DELTA 3.40000010 sec
MCREST 0 sec
MCWRK 0.01500000 sec
======== CHANNEL f1 ========
NUC1 13C
PL1 0 dB
SFO1 150.4592037 MHz
======== CHANNEL f2 ========
CPDPRG2 waltz16
NUC2 1H
PCPD2 92.00 usec
PL2 120.00 dB
PL12 5.50 dB
PL13 9.00 dB
SFO2 598.3029915 MHz
F2 - Processing parameters
SI 65536
SF 150.4426413 MHz
WDW EM
SSB 0
LB 3.00 Hz
GB 0
PC 1.00
H
N
O
CH3
H
N
O
5r
210 200 190 180 170 160 150 140 130 120 110 100 90 80 70 60 50 40 30 20 10 ppm

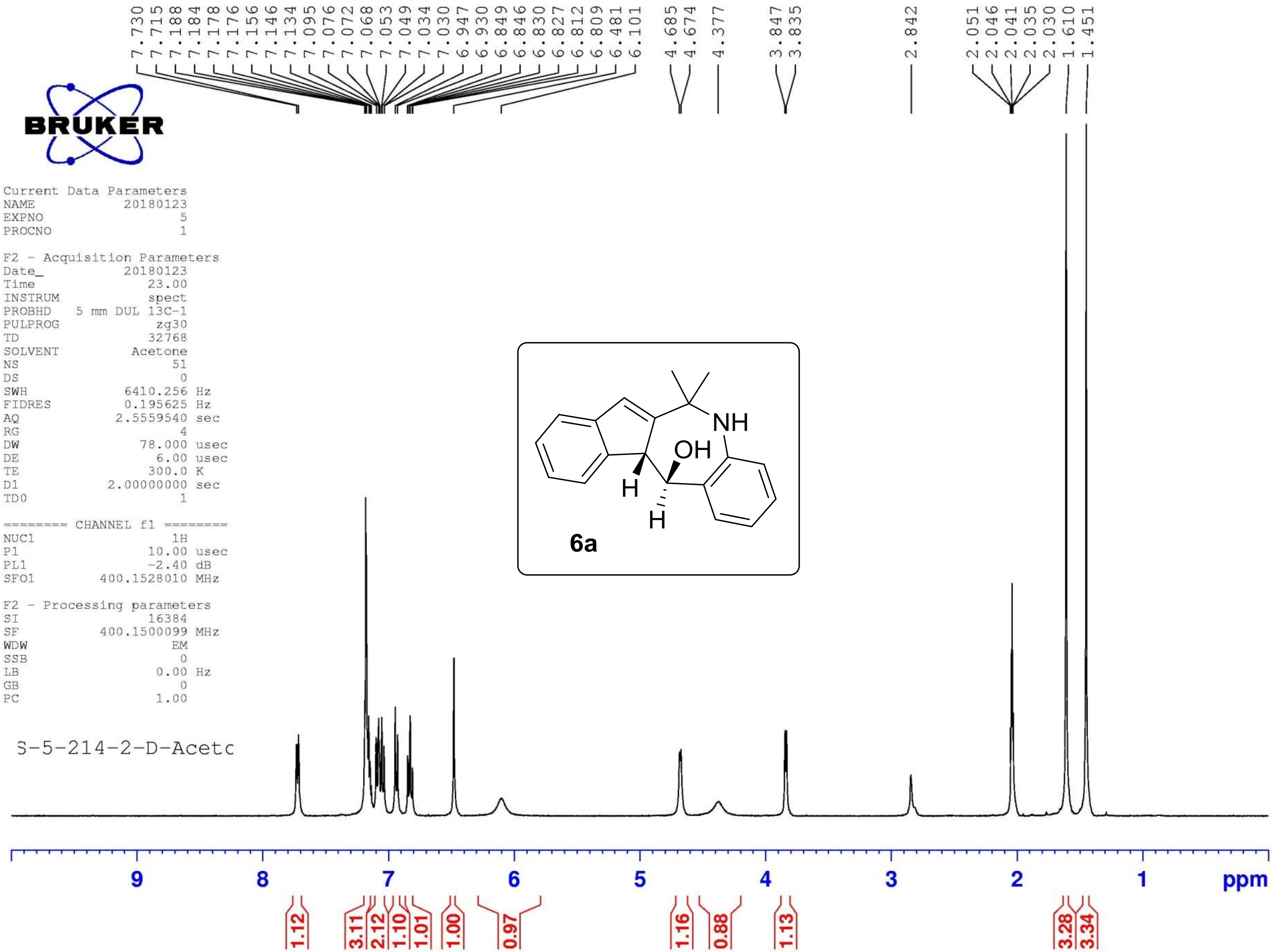
7.730
7.715
7.188
7.184
7.178
7.176
7.156
7.146
7.134
7.095
7.076
7.072
7.068
7.053
7.049
7.034
7.030
6.947
6.930
6.849
6.846
6.830
6.827
6.812
6.809
6.481
6.101
4.685
4.674
4.377
3.847
3.835
2.842
2.051
2.046
2.041
2.035
2.030
1.610
1.451
BRUKER
Current Data Parameters
NAME 20180123
EXPNO 5
PROCNO 1
F2 - Acquisition Parameters
Date_ 20180123
Time 23.00
INSTRUM spect
PROBHD 5 mm DUL 13C-1
PULPROG zg30
TD 32768
SOLVENT Acetone
NS 51
DS 0
SWH 6410.256 Hz
FIDRES 0.195625 Hz
AQ 2.5559540 sec
RG 4
DW 78.000 usec
DE 6.00 usec
TE 300.0 K
D1 2.00000000 sec
TD0 1
======== CHANNEL f1 ========
NUC1 1H
P1 10.00 usec
PL1 -2.40 dB
SFO1 400.1528010 MHz
F2 - Processing parameters
SI 16384
SF 400.1500099 MHz
WDW EM
SSB 0
LB 0.00 Hz
GB 0
PC 1.00
S-5-214-2-D-Acetc
NH
OH
H
H
6a
9
8
7
6
5
4
3
2
1
ppm
1.12
3.11
2.12
1.10
1.01
1.00
0.97
1.16
0.88
1.13
3.28
3.34

Current Data Parameters
NAME 20180123
EXPNO 2
PROCNO 1

F2 - Acquisition Parameters
Date_ 20180123
Time 17.26
INSTRUM spect
PROBHD 5 mm DUL 13C-1
PULPROG zgpg30
TD 65536
SOLVENT CDCl3
NS 80
DS 0
SWH 22727.273 Hz
FIDRES 0.346791 Hz
AQ 1.4418420 sec
RG 57
DW 22.000 usec
DE 6.00 usec
TE 300.0 K
D1 2.00000000 sec
d11 0.03000000 sec
DELTA 1.89999998 sec
TD0 1

======== CHANNEL f1 ========
NUC1 13C
P1 9.70 usec
PL1 -0.50 dB
SFO1 100.6288660 MHz

======== CHANNEL f2 ========
CPDPRG2 waltz16
NUC2 1H
PCPD2 90.00 usec
PL2 -2.40 dB
PL12 15.10 dB
PL13 18.10 dB
SFO2 400.1516010 MHz

F2 - Processing parameters
SI 32768
SF 100.6178150 MHz
WDW EM
SSB 0
LB 3.00 Hz
GB 0
PC 1.00

RKS-5-214-2

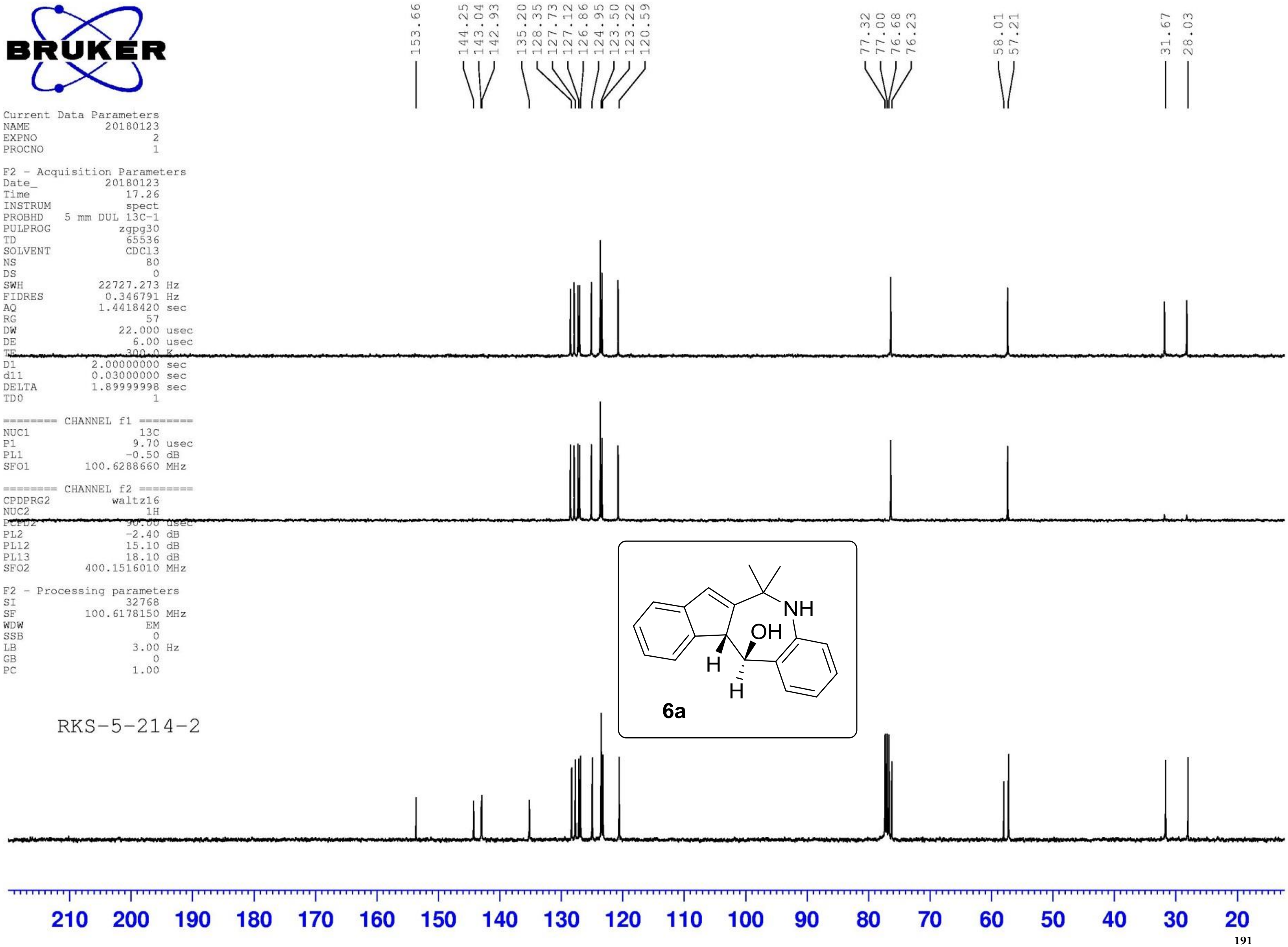

6a

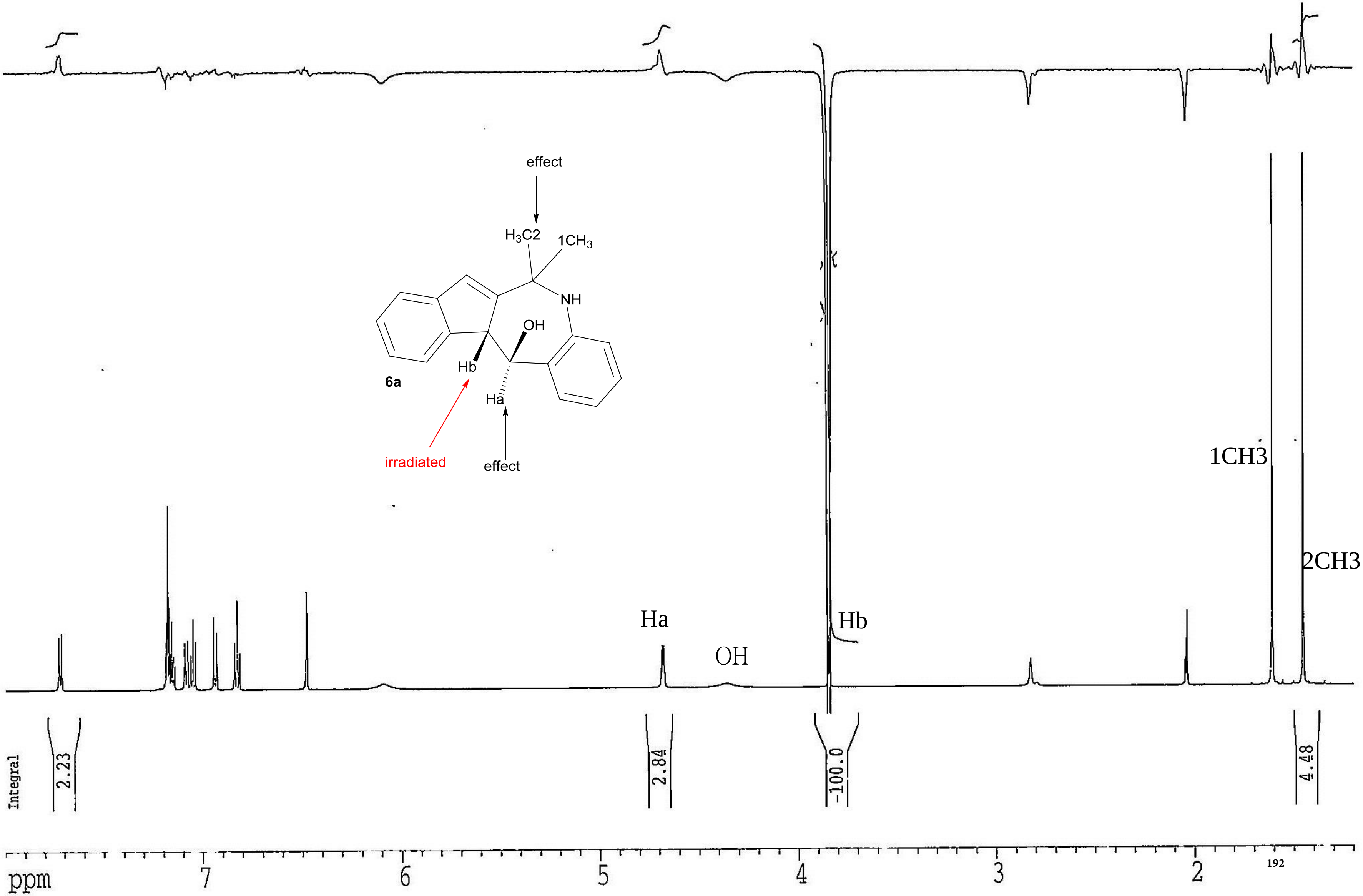
effect
H_3C2
$1CH_3$
NH
OH
Hb
6a
Ha
irradiated
effect
1CH3
2CH3
Ha
OH
Hb
Integral
2.23
2.84
-100.0
4.48
ppm
7
6
5
4
3
2

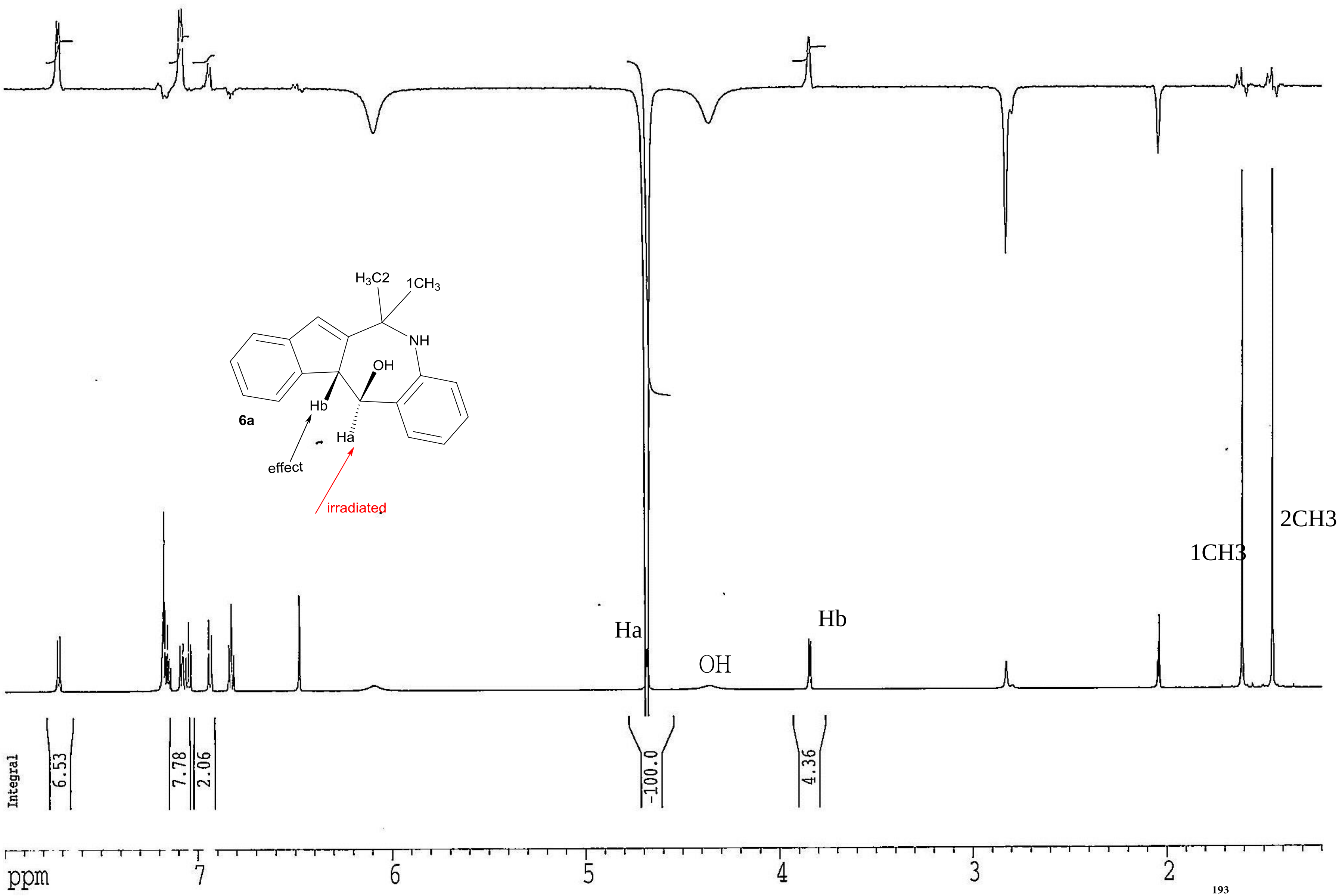
H3C2
1CH3
NH
OH
Hb
Ha
6a
effect
irradiated
Ha
OH
Hb
1CH3
2CH3
Integral
6.53
7.78
2.06
-100.0
4.36
ppm
7
6
5
4
3
2

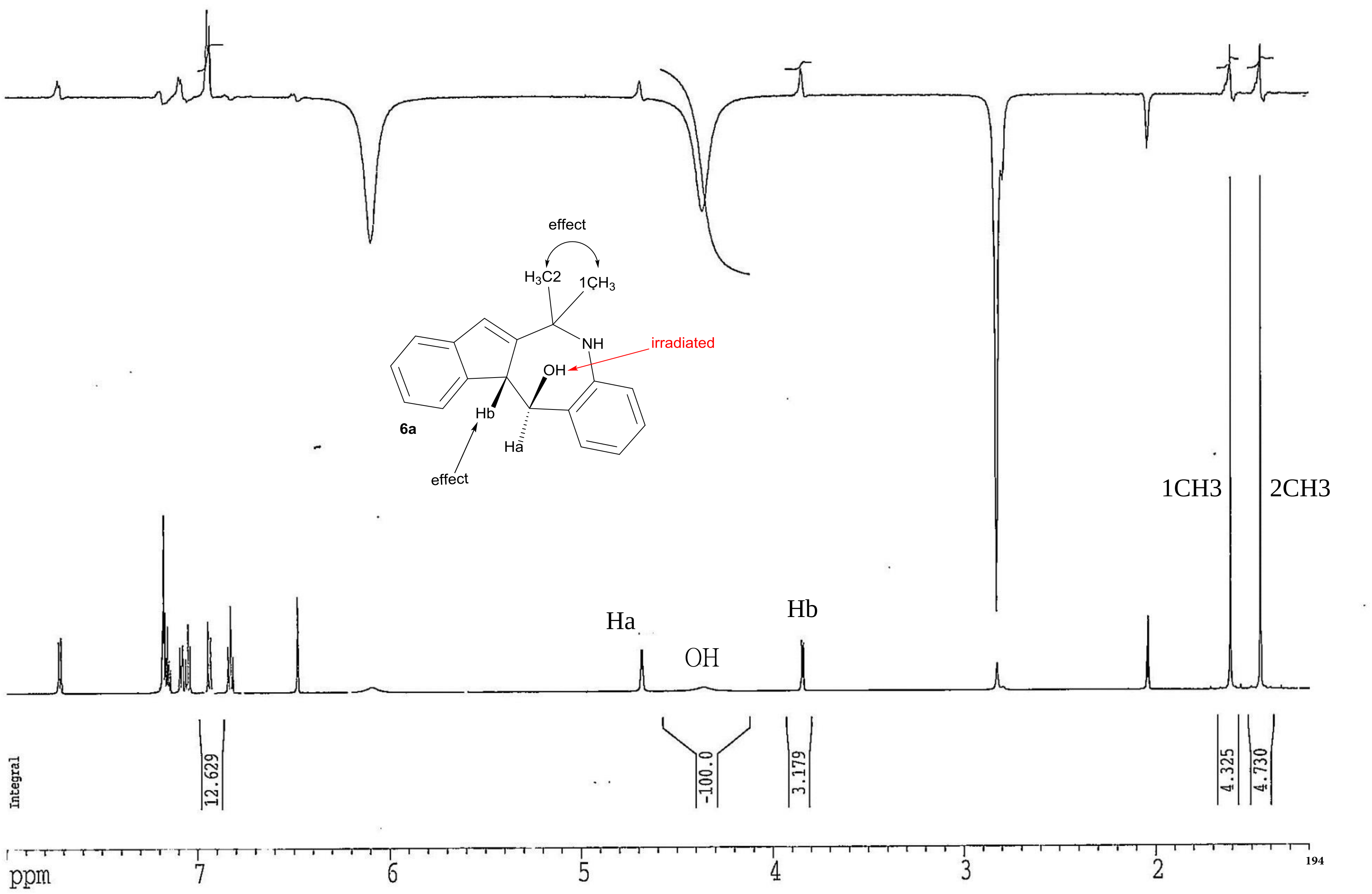
effect
H3C2
1CH3
NH
irradiated
OH
Hb
Ha
6a
effect
1CH3
2CH3
Ha
OH
Hb
Integral
12.629
-100.0
3.179
4.325
4.730
ppm
7
6
5
4
3
2

```
Current Data Parameters
NAME        RKS-5-214
EXPNO              1
PROCNO             1

F2 - Acquisition Parameters
Date_         20180124
Time             12.40
INSTRUM          spect
PROBHD   5 mm QNP 1H/1
PULPROG             zg
TD               32768
SOLVENT        Acetone
NS                  32
DS                   0
SWH           9541.984 Hz
FIDRES        0.291198 Hz
AQ           1.7170932 sec
RG                 512
DW              52.400 usec
DE                6.50 usec
TE               296.9 K
D1          2.00000000 sec
MCREST      0.00000000 sec
MCWRK       0.01500000 sec

======== CHANNEL f1 ========
NUC1                1H
P1               10.00 usec
PL1              -1.00 dB
SFO1       598.3029915 MHz

F2 - Processing parameters
SI               32768
SF         598.3000200 MHz
WDW                 no
SSB                  0
LB                0.00 Hz
GB                   0
PC                2.00

1D NMR plot parameters
CX               20.00 cm
CY               15.00 cm
F1P             10.000 ppm
F1             5983.00 Hz
F2P             -0.500 ppm
F2             -299.15 Hz
PPMCM          0.52500 ppm/cm
HZCM         314.10751 Hz/cm
```

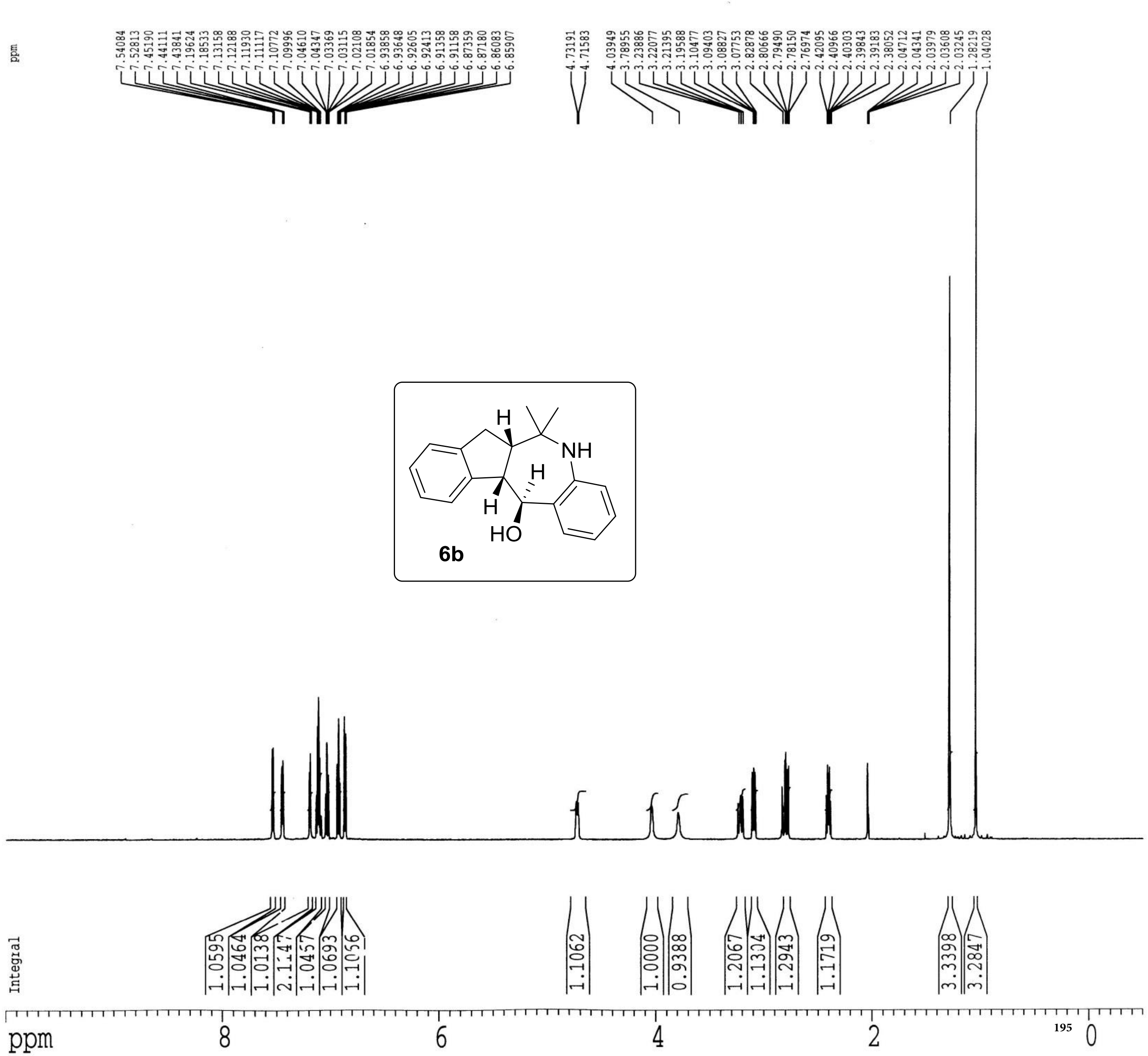

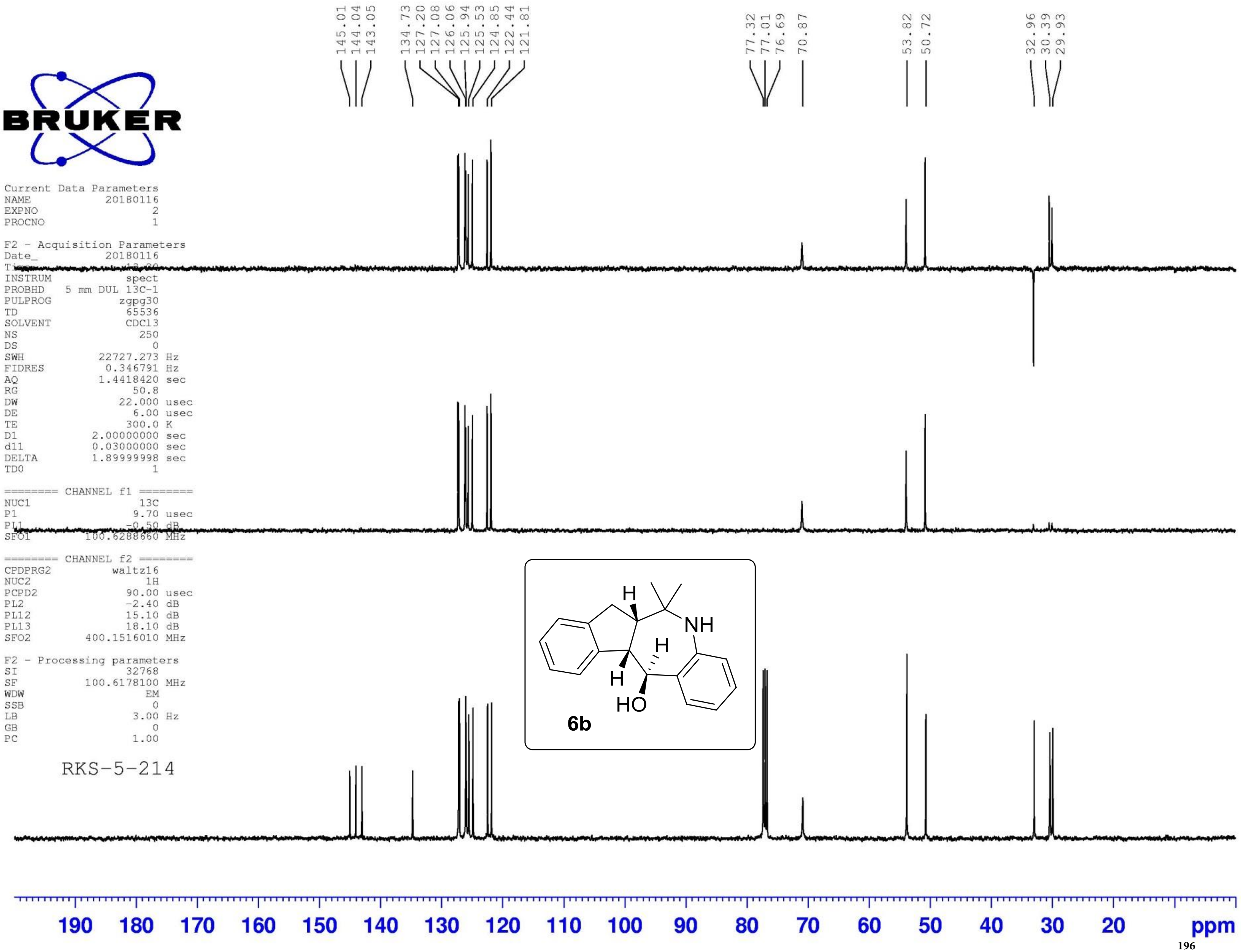
145.01
144.04
143.05
134.73
127.20
127.08
126.06
125.94
125.53
124.85
122.44
121.81
77.32
77.01
76.69
70.87
53.82
50.72
32.96
30.39
29.93
Current Data Parameters
NAME 20180116
EXPNO 2
PROCNO 1
F2 - Acquisition Parameters
Date_ 20180116
Time [illegible]
INSTRUM spect
PROBHD 5 mm DUL 13C-1
PULPROG zgpg30
TD 65536
SOLVENT CDCl3
NS 250
DS 0
SWH 22727.273 Hz
FIDRES 0.346791 Hz
AQ 1.4418420 sec
RG 50.8
DW 22.000 usec
DE 6.00 usec
TE 300.0 K
D1 2.00000000 sec
d11 0.03000000 sec
DELTA 1.89999998 sec
TD0 1
======== CHANNEL f1 ========
NUC1 13C
P1 9.70 usec
PL1 -0.50 dB
SFO1 100.6288660 MHz
======== CHANNEL f2 ========
CPDPRG2 waltz16
NUC2 1H
PCPD2 90.00 usec
PL2 -2.40 dB
PL12 15.10 dB
PL13 18.10 dB
SFO2 400.1516010 MHz
F2 - Processing parameters
SI 32768
SF 100.6178100 MHz
WDW EM
SSB 0
LB 3.00 Hz
GB 0
PC 1.00
RKS-5-214
190 180 170 160 150 140 130 120 110 100 90 80 70 60 50 40 30 20 ppm
H
NH
H
H
HO

6b

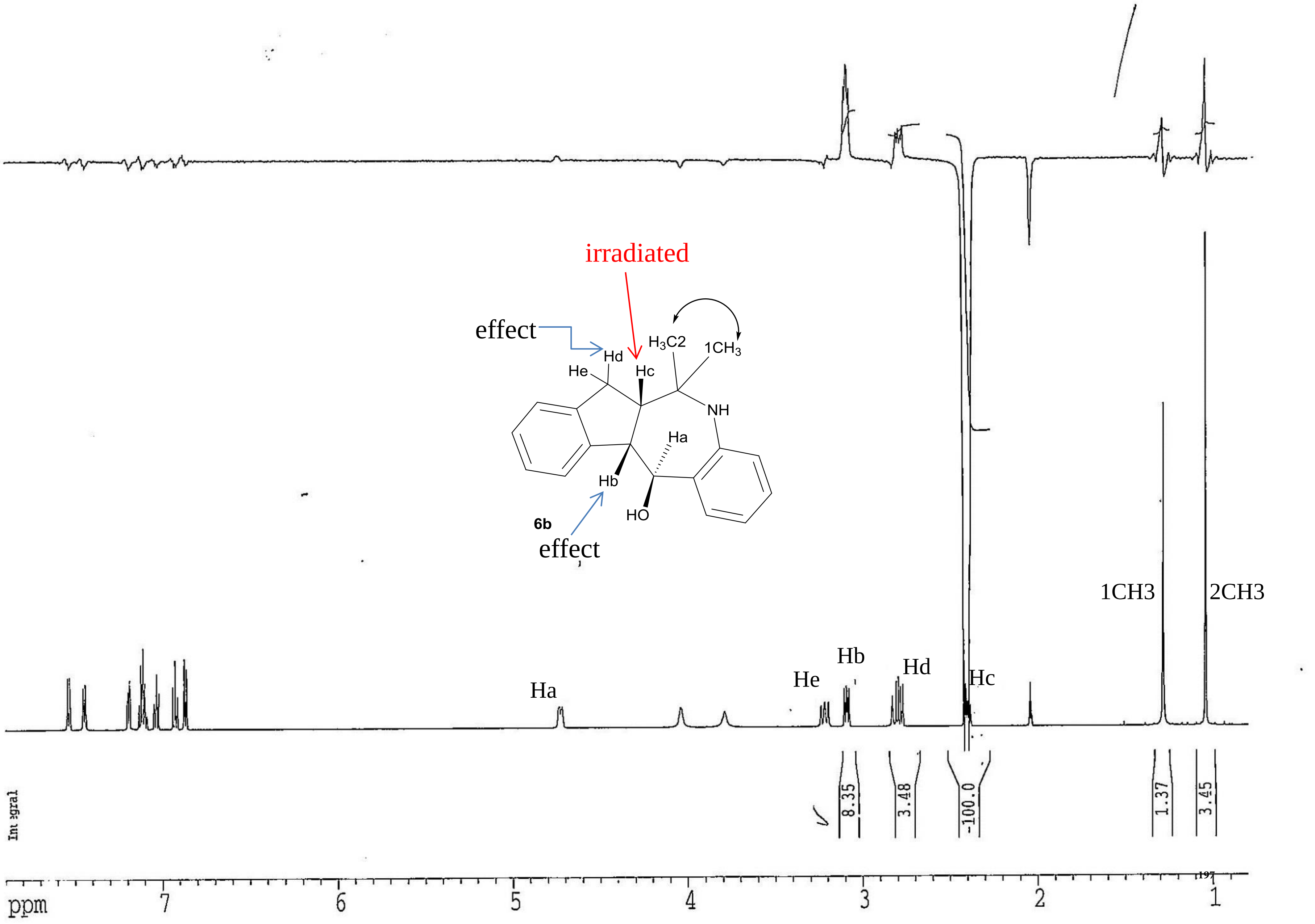

irradiated
effect
Hd
He
Hc
H3C2
1CH3
NH
Ha
Hb
HO
6b
effect
1CH3
2CH3
Ha
He
Hb
Hd
Hc
8.35
3.48
-100.0
1.37
3.45
Integral
ppm
7
6
5
4
3
2
1

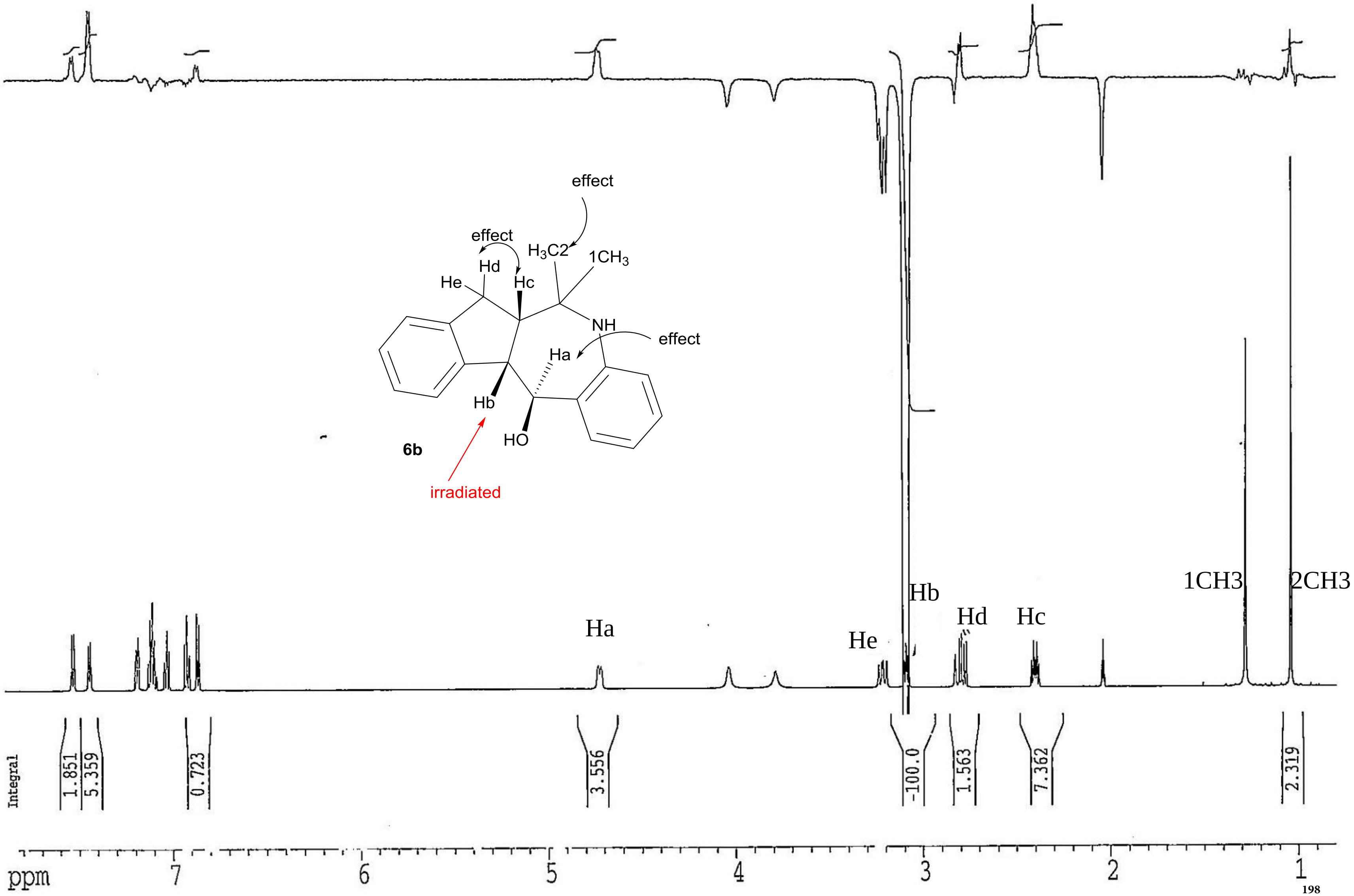
effect
effect
H3C2
1CH3
Hd
He
Hc
NH
effect
Ha
Hb
HO
6b
irradiated
Ha
He
Hb
Hd
Hc
1CH3
2CH3
Integral
1.851
5.359
0.723
3.556
-100.0
1.563
7.362
2.319
ppm
7
6
5
4
3
2
1

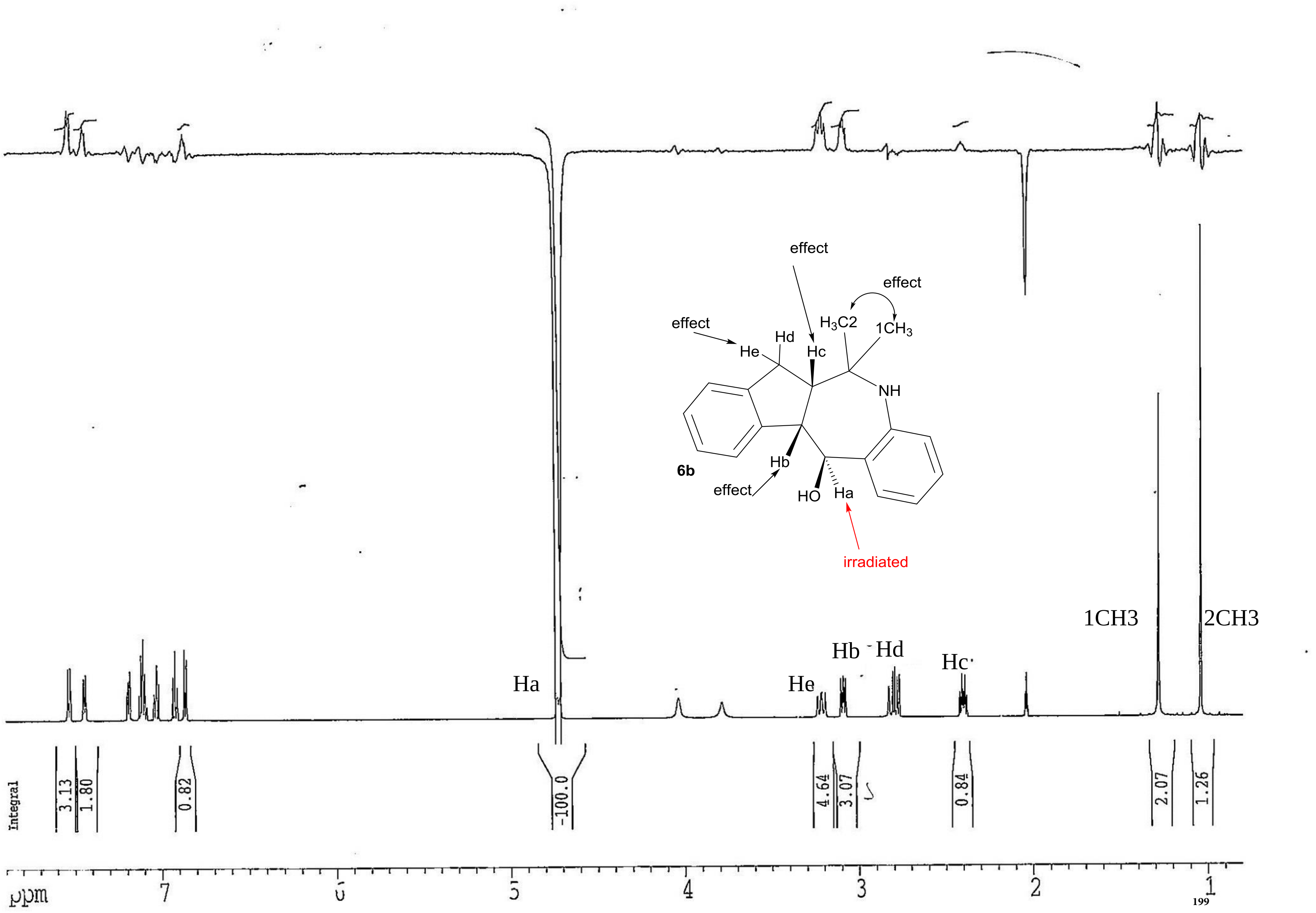
effect
effect
effect
He
Hd
Hc
H_3C2
$1CH_3$
NH
6b
Hb
effect
HO
Ha
irradiated
1CH3
2CH3
Ha
He
Hb
Hd
Hc
Integral
3.13
1.80
0.82
-100.0
4.64
3.07
0.84
2.07
1.26
ppm
7
5
4
3
2
1

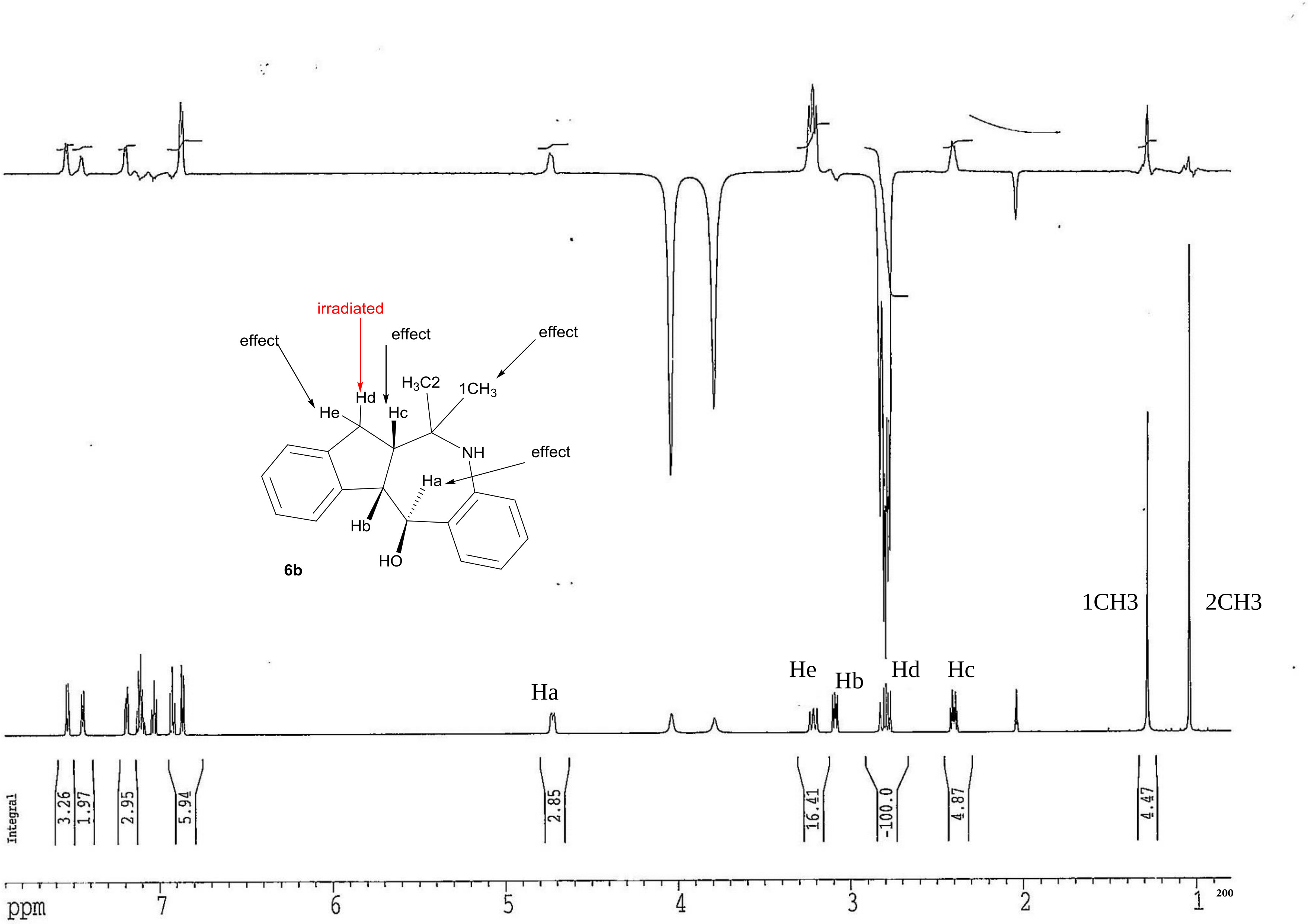

irradiated
effect
effect
effect
effect
He
Hd
Hc
H_3C2
$1CH_3$
NH
Ha
Hb
HO
6b
1CH3
2CH3
He
Hb
Hd
Hc
Ha
Integral
3.26
1.97
2.95
5.94
2.85
16.41
-100.0
4.87
4.47
ppm
7
6
5
4
3
2
1

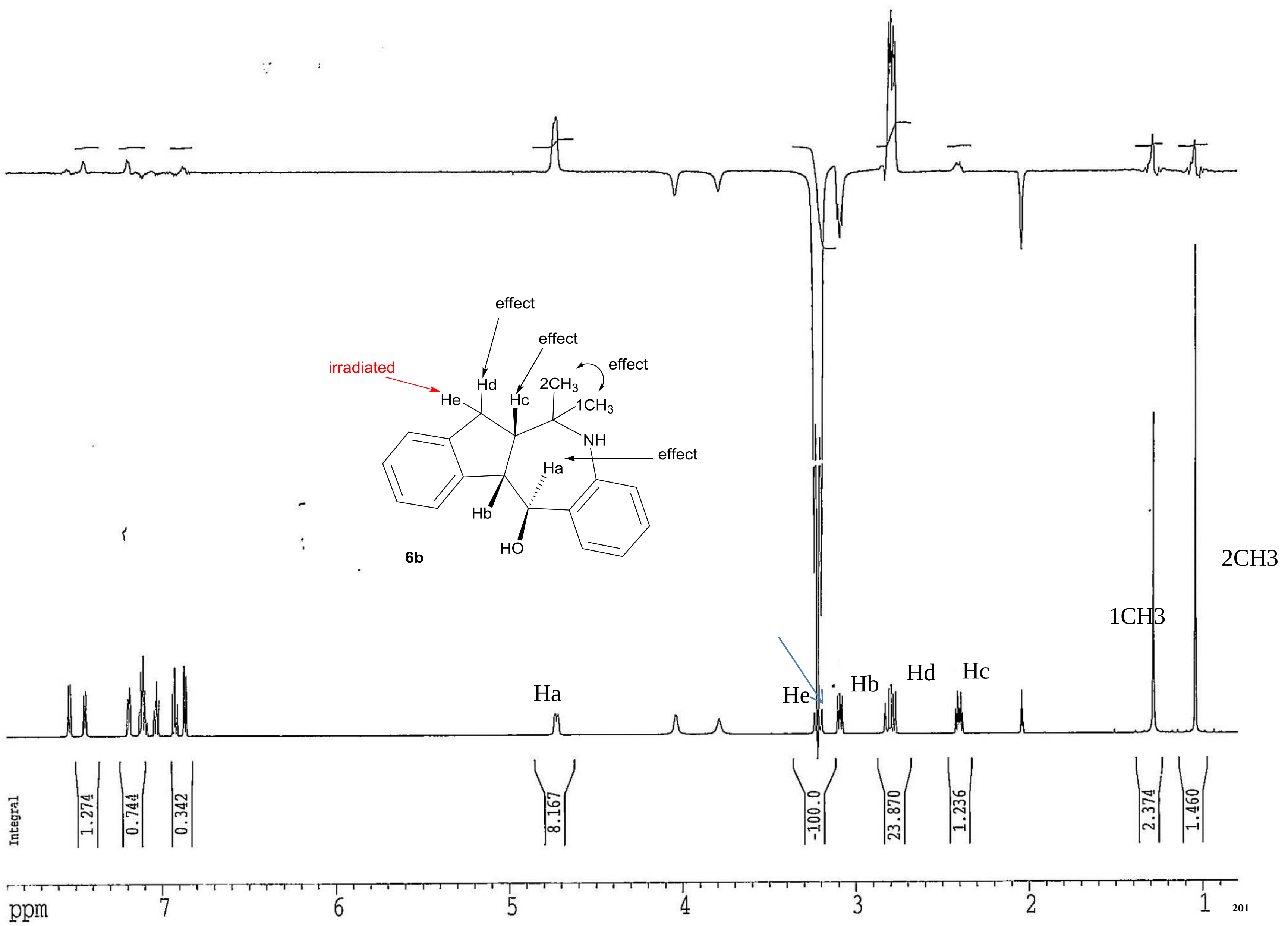
effect
effect
effect
irradiated
Hd
He
Hc
2CH3
1CH3
NH
effect
Ha
Hb
HO
6b
2CH3
1CH3
Ha
He
Hb
Hd
Hc
Integral
1.274
0.744
0.342
8.167
-100.0
23.870
1.236
2.374
1.460
ppm
7
6
5
4
3
2
1

Current Data Parameters
NAME 20180112
EXPNO 5
PROCNO 1

F2 - Acquisition Parameters
Date_ 20180112
Time 0.00
INSTRUM spect
PROBHD 5 mm DUL 13C-1
PULPROG zg30
TD 32768
SOLVENT CDCl3
NS 25
DS 0
SWH 6410.256 Hz
FIDRES 0.195625 Hz
AQ 2.5559540 sec
RG 4
DW 78.000 usec
DE 6.00 usec
TE 300.0 K
D1 2.00000000 sec
TD0 1

======== CHANNEL f1 ========
NUC1 1H
P1 10.00 usec
PL1 -2.40 dB
SFO1 400.1528010 MHz

F2 - Processing parameters
SI 16384
SF 400.1500170 MHz
WDW EM
SSB 0
LB 0.00 Hz
GB 0
PC 1.00

RKS-5-213-P

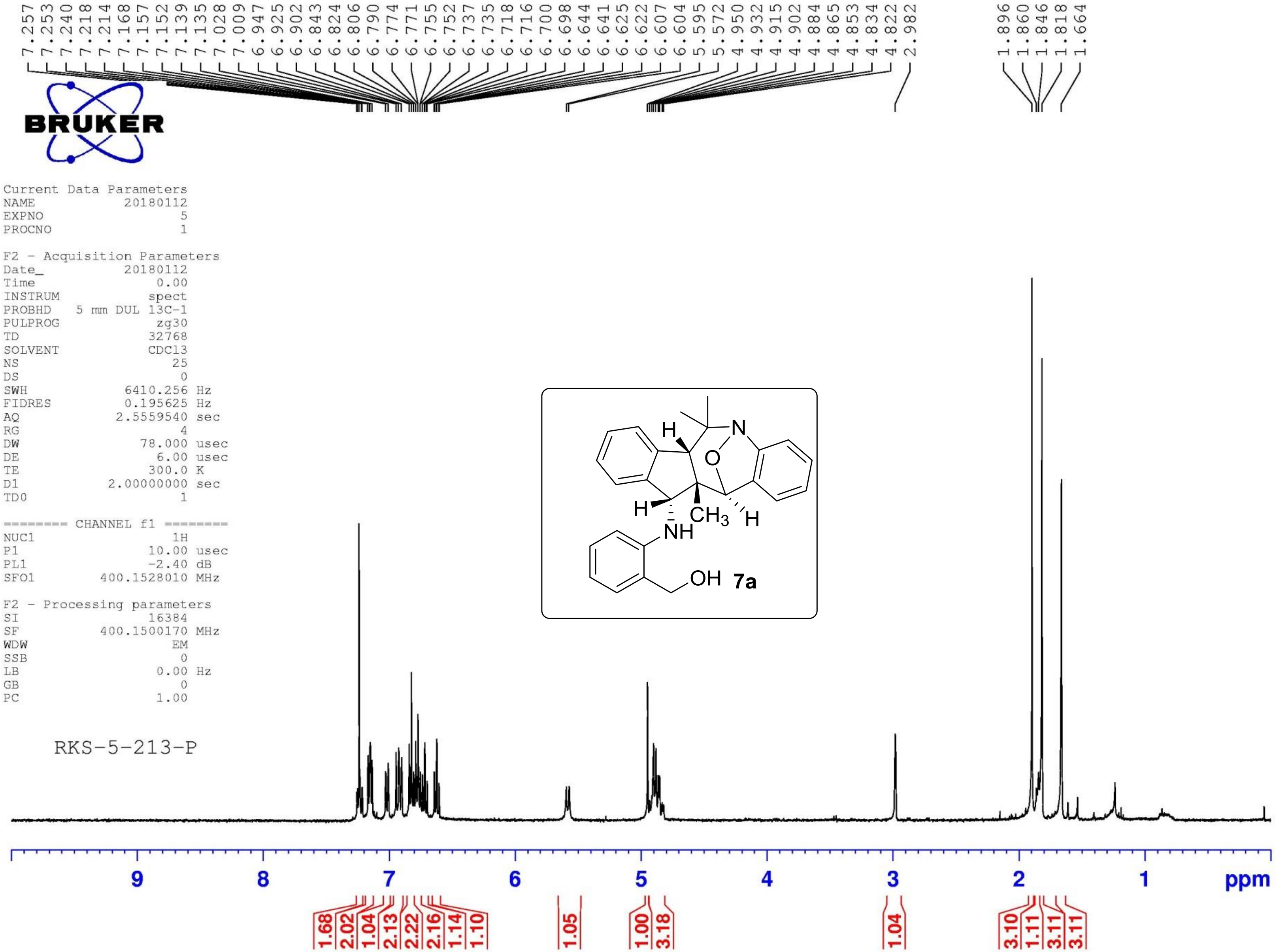

7a

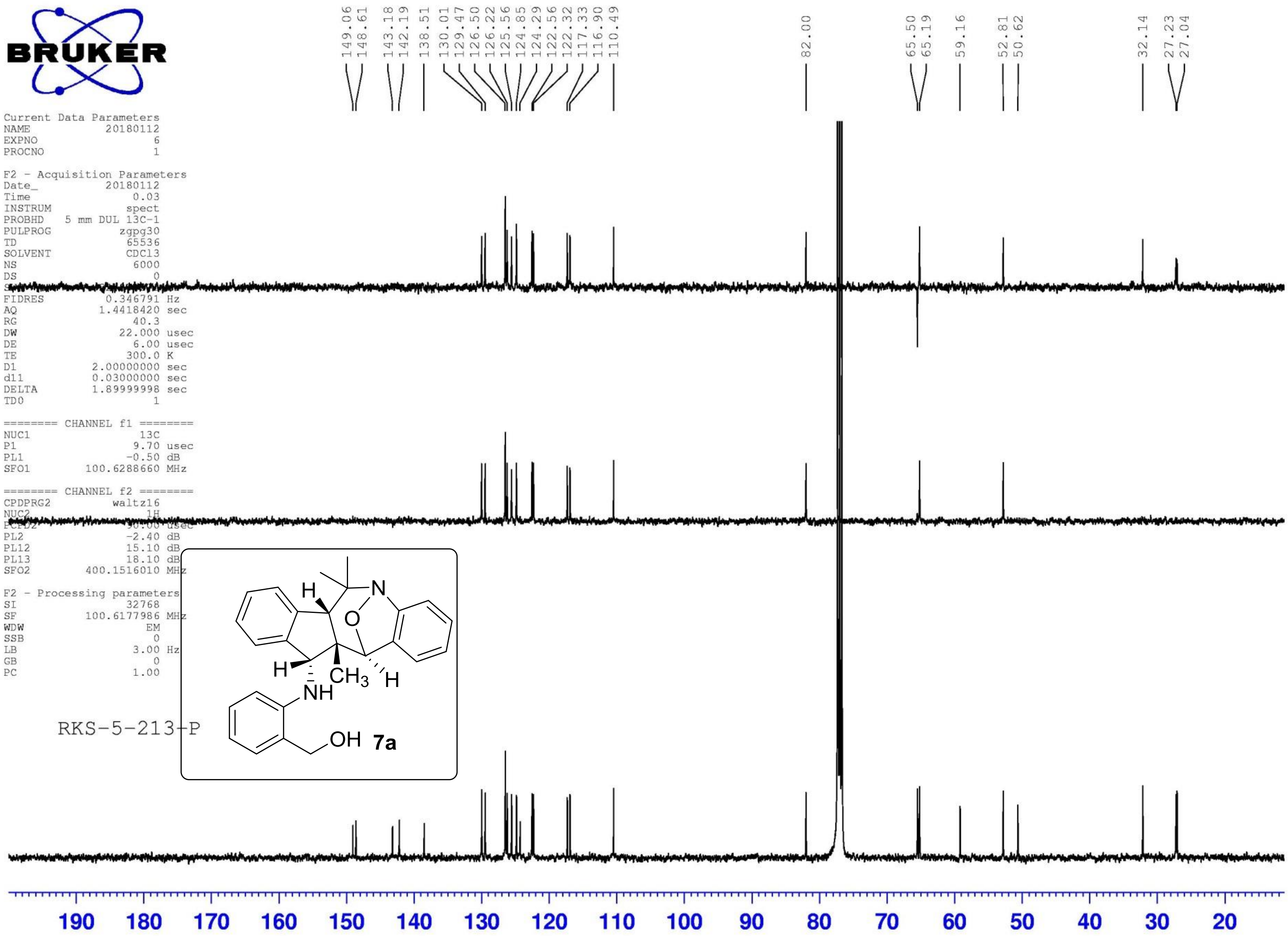
BRUKER
149.06
148.61
143.18
142.19
138.51
130.01
129.47
126.50
126.22
125.56
124.85
124.29
122.56
122.32
117.33
116.90
110.49
82.00
65.50
65.19
59.16
52.81
50.62
32.14
27.23
27.04
Current Data Parameters
NAME 20180112
EXPNO 6
PROCNO 1
F2 - Acquisition Parameters
Date_ 20180112
Time 0.03
INSTRUM spect
PROBHD 5 mm DUL 13C-1
PULPROG zgpg30
TD 65536
SOLVENT CDCl3
NS 6000
DS 0
FIDRES 0.346791 Hz
AQ 1.4418420 sec
RG 40.3
DW 22.000 usec
DE 6.00 usec
TE 300.0 K
D1 2.00000000 sec
d11 0.03000000 sec
DELTA 1.89999998 sec
TD0 1
======== CHANNEL f1 ========
NUC1 13C
P1 9.70 usec
PL1 -0.50 dB
SFO1 100.6288660 MHz
======== CHANNEL f2 ========
CPDPRG2 waltz16
NUC2 1H
PL2 -2.40 dB
PL12 15.10 dB
PL13 18.10 dB
SFO2 400.1516010 MHz
F2 - Processing parameters
SI 32768
SF 100.6177986 MHz
WDW EM
SSB 0
LB 3.00 Hz
GB 0
PC 1.00
RKS-5-213-P
H
N
O
H
CH3
H
NH
OH
7a
190
180
170
160
150
140
130
120
110
100
90
80
70
60
50
40
30
20

Current Data Parameters
NAME 20180223
EXPNO 1
PROCNO 1

F2 - Acquisition Parameters
Date_ 20180223
Time 14.27
INSTRUM spect
PROBHD 5 mm DUL 13C-1
PULPROG zg30
TD 32768
SOLVENT CDCl3
NS 8
DS 0
SWH 6410.256 Hz
FIDRES 0.195625 Hz
AQ 2.5559540 sec
RG 4
DW 78.000 usec
DE 6.00 usec
TE 300.0 K
D1 2.00000000 sec
TD0 1

======== CHANNEL f1 ========
NUC1 1H
P1 10.00 usec
PL1 -2.40 dB
SFO1 400.1528010 MHz

F2 - Processing parameters
SI 16384
SF 400.1500169 MHz
WDW EM
SSB 0
LB 0.00 Hz
GB 0
PC 1.00

RKS-5-219

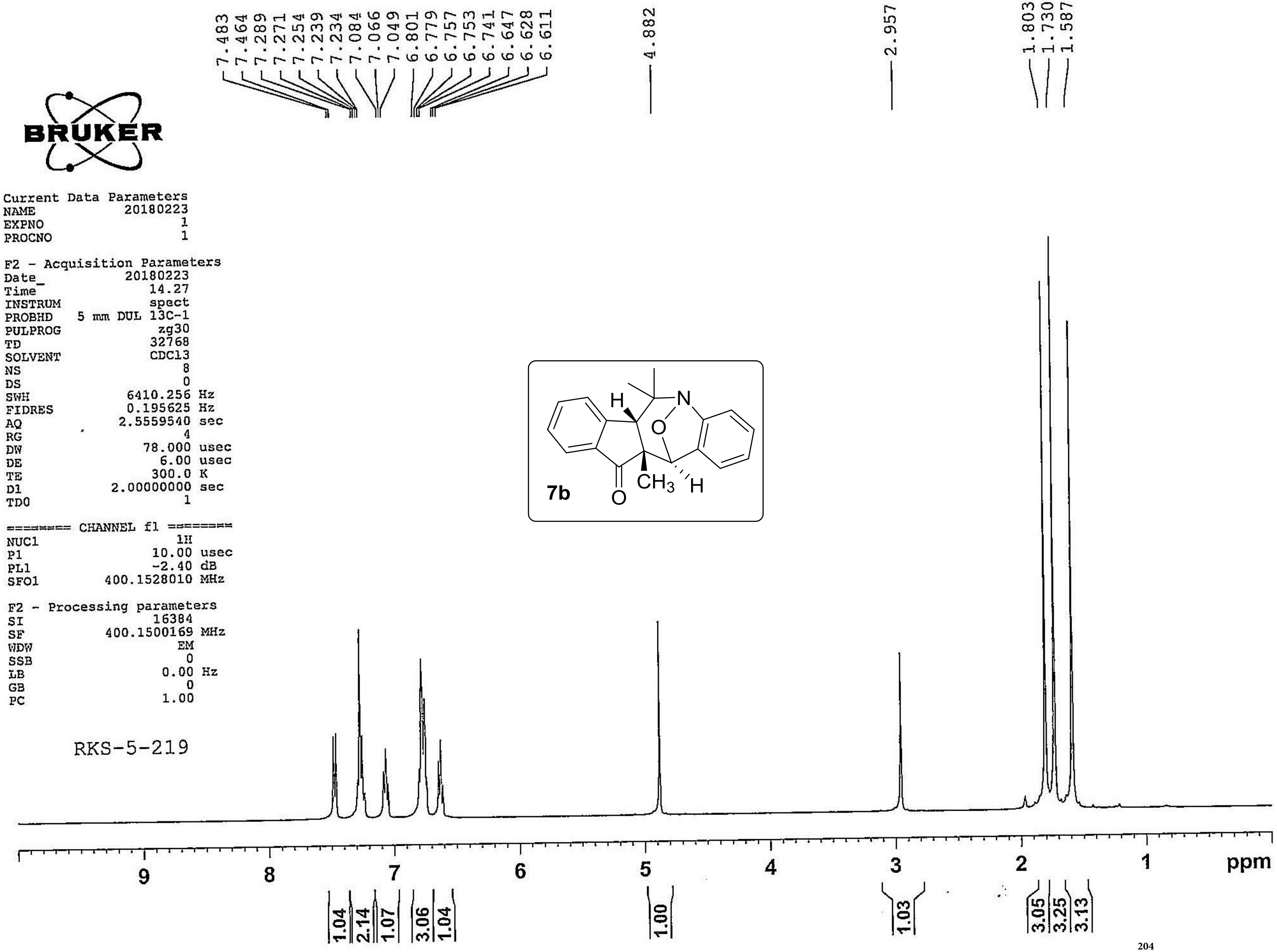

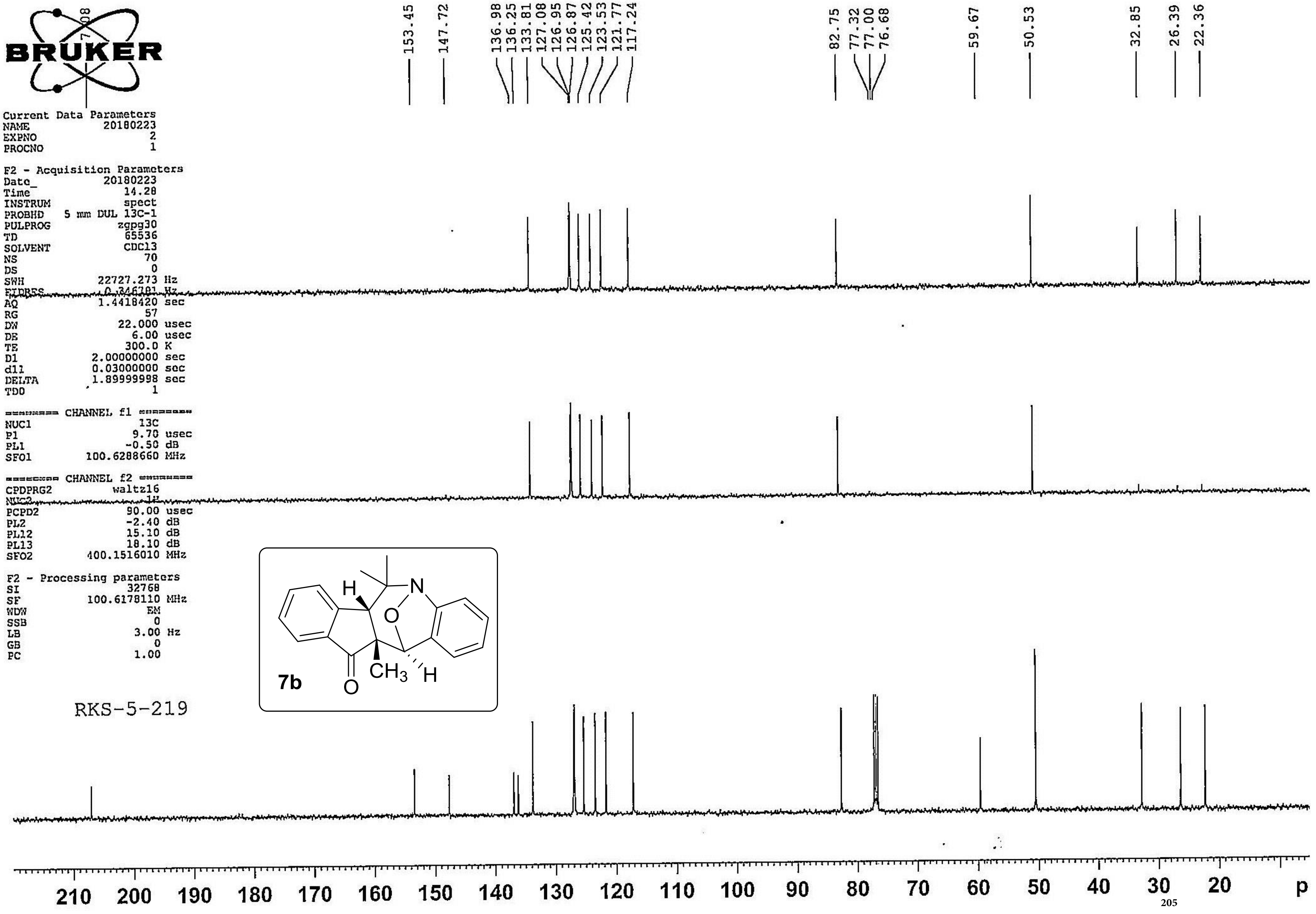
BRUKER
Current Data Parameters
NAME 20180223
EXPNO 2
PROCNO 1
F2 - Acquisition Parameters
Date_ 20180223
Time 14.28
INSTRUM spect
PROBHD 5 mm DUL 13C-1
PULPROG zgpg30
TD 65536
SOLVENT CDCl3
NS 70
DS 0
SWH 22727.273 Hz
FIDRES 0.346791 Hz
AQ 1.4418420 sec
RG 57
DW 22.000 usec
DE 6.00 usec
TE 300.0 K
D1 2.00000000 sec
d11 0.03000000 sec
DELTA 1.89999998 sec
TD0 1
======== CHANNEL f1 ========
NUC1 13C
P1 9.70 usec
PL1 -0.50 dB
SFO1 100.6288660 MHz
======== CHANNEL f2 ========
CPDPRG2 waltz16
NUC2 1H
PCPD2 90.00 usec
PL2 -2.40 dB
PL12 15.10 dB
PL13 18.10 dB
SFO2 400.1516010 MHz
F2 - Processing parameters
SI 32768
SF 100.6178110 MHz
WDW EM
SSB 0
LB 3.00 Hz
GB 0
PC 1.00
RKS-5-219
153.45
147.72
136.98
136.25
133.81
127.08
126.95
126.87
125.42
123.53
121.77
117.24
82.75
77.32
77.00
76.68
59.67
50.53
32.85
26.39
22.36
H
N
O
CH3
H
O
7b
210
200
190
180
170
160
150
140
130
120
110
100
90
80
70
60
50
40
30
20
p

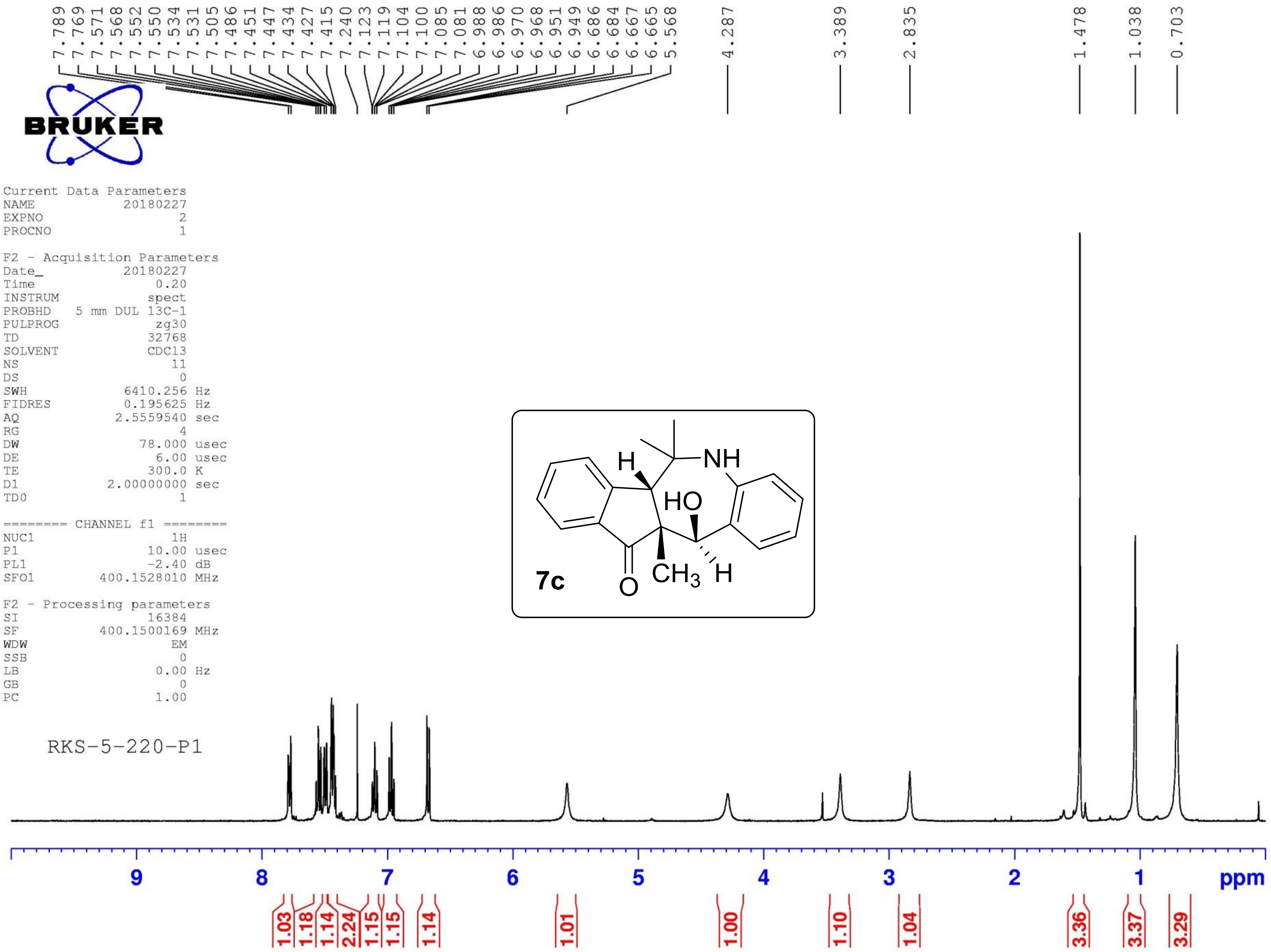
7.789 7.769 7.571 7.568 7.552 7.550 7.534 7.531 7.505 7.486 7.451 7.447 7.434 7.427 7.415 7.240 7.123 7.119 7.104 7.100 7.085 7.081 6.988 6.986 6.970 6.968 6.951 6.949 6.686 6.684 6.667 6.665 5.568
4.287
3.389
2.835
1.478
1.038
0.703
BRUKER
Current Data Parameters
NAME 20180227
EXPNO 2
PROCNO 1
F2 - Acquisition Parameters
Date_ 20180227
Time 0.20
INSTRUM spect
PROBHD 5 mm DUL 13C-1
PULPROG zg30
TD 32768
SOLVENT CDCl3
NS 11
DS 0
SWH 6410.256 Hz
FIDRES 0.195625 Hz
AQ 2.5559540 sec
RG 4
DW 78.000 usec
DE 6.00 usec
TE 300.0 K
D1 2.00000000 sec
TD0 1
======== CHANNEL f1 ========
NUC1 1H
P1 10.00 usec
PL1 -2.40 dB
SFO1 400.1528010 MHz
F2 - Processing parameters
SI 16384
SF 400.1500169 MHz
WDW EM
SSB 0
LB 0.00 Hz
GB 0
PC 1.00
RKS-5-220-P1
H
NH
HO
7c
O
CH3
H
9
8
7
6
5
4
3
2
1
ppm
1.03 1.18 1.14 2.24 1.15 1.15 1.14
1.01
1.00
1.10
1.04
3.36
3.37
3.29

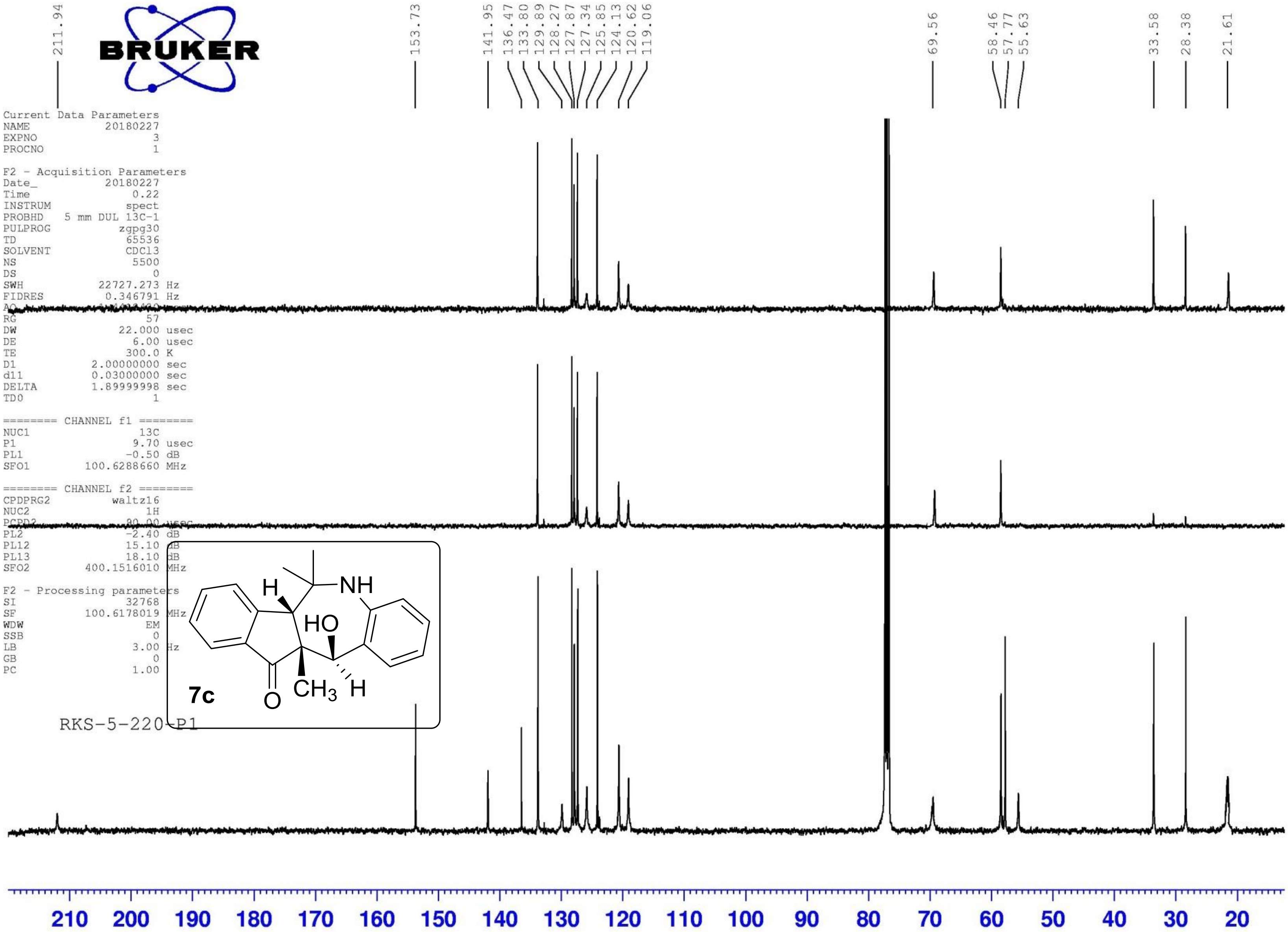
211.94
BRUKER
153.73
141.95
136.47
133.80
129.89
128.27
127.87
127.34
125.85
124.13
120.62
119.06
69.56
58.46
57.77
55.63
33.58
28.38
21.61
Current Data Parameters
NAME 20180227
EXPNO 3
PROCNO 1
F2 - Acquisition Parameters
Date_ 20180227
Time 0.22
INSTRUM spect
PROBHD 5 mm DUL 13C-1
PULPROG zgpg30
TD 65536
SOLVENT CDCl3
NS 5500
DS 0
SWH 22727.273 Hz
FIDRES 0.346791 Hz
RG 57
DW 22.000 usec
DE 6.00 usec
TE 300.0 K
D1 2.00000000 sec
d11 0.03000000 sec
DELTA 1.89999998 sec
TD0 1
======== CHANNEL f1 ========
NUC1 13C
P1 9.70 usec
PL1 -0.50 dB
SFO1 100.6288660 MHz
======== CHANNEL f2 ========
CPDPRG2 waltz16
NUC2 1H
PL2 -2.40 dB
PL12 15.10 dB
PL13 18.10 dB
SFO2 400.1516010 MHz
F2 - Processing parameters
SI 32768
SF 100.6178019 MHz
WDW EM
SSB 0
LB 3.00 Hz
GB 0
PC 1.00
RKS-5-220-P1
H
NH
HO
O
CH3
H
7c
210
200
190
180
170
160
150
140
130
120
110
100
90
80
70
60
50
40
30
20

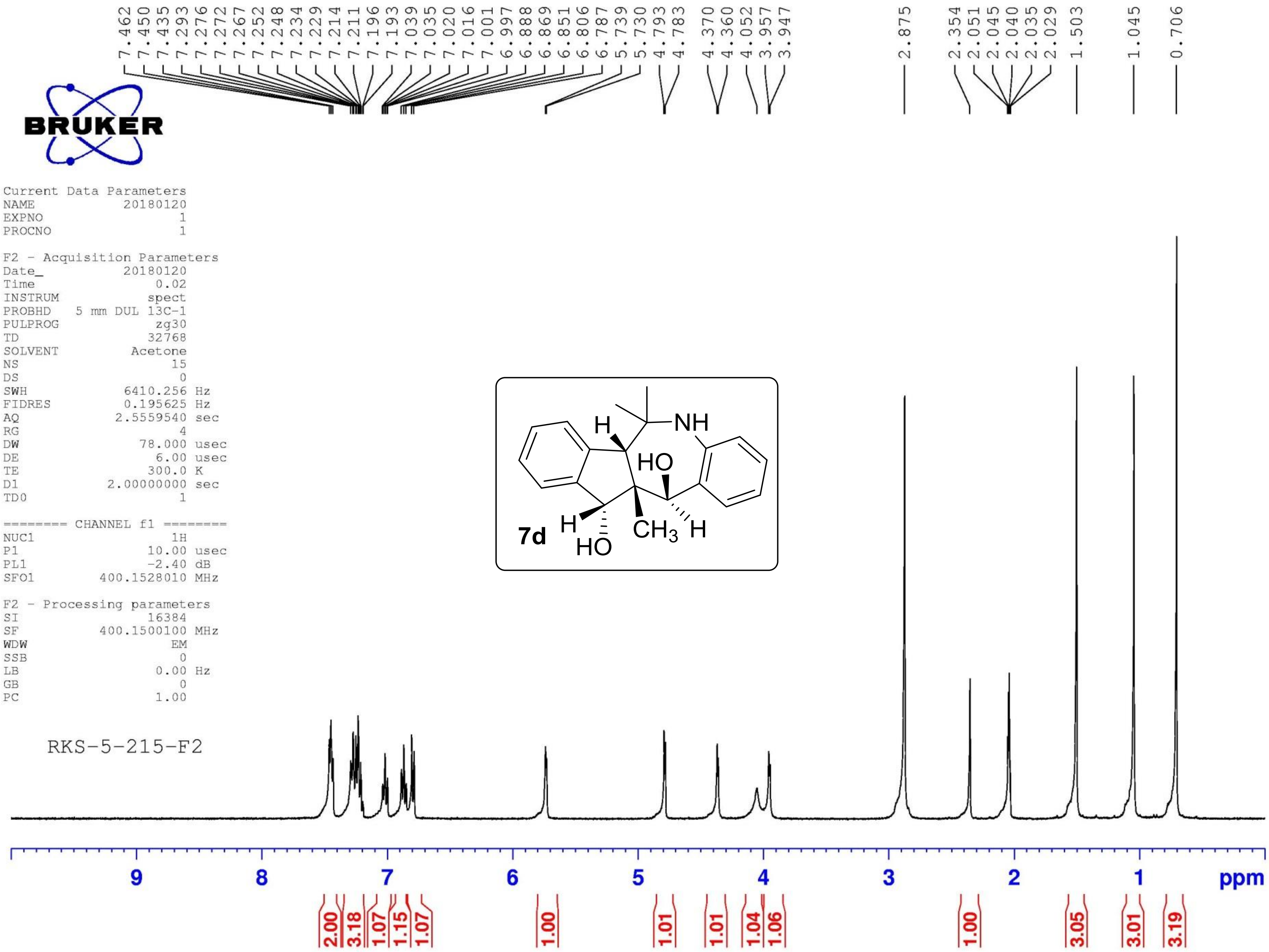
7.462
7.450
7.435
7.293
7.276
7.272
7.267
7.252
7.248
7.234
7.229
7.214
7.211
7.196
7.193
7.039
7.035
7.020
7.016
7.001
6.997
6.888
6.869
6.851
6.806
6.787
5.739
5.730
4.793
4.783
4.370
4.360
4.052
3.957
3.947
2.875
2.354
2.051
2.045
2.040
2.035
2.029
1.503
1.045
0.706
BRUKER
Current Data Parameters
NAME 20180120
EXPNO 1
PROCNO 1
F2 - Acquisition Parameters
Date_ 20180120
Time 0.02
INSTRUM spect
PROBHD 5 mm DUL 13C-1
PULPROG zg30
TD 32768
SOLVENT Acetone
NS 15
DS 0
SWH 6410.256 Hz
FIDRES 0.195625 Hz
AQ 2.5559540 sec
RG 4
DW 78.000 usec
DE 6.00 usec
TE 300.0 K
D1 2.00000000 sec
TD0 1
======== CHANNEL f1 ========
NUC1 1H
P1 10.00 usec
PL1 -2.40 dB
SFO1 400.1528010 MHz
F2 - Processing parameters
SI 16384
SF 400.1500100 MHz
WDW EM
SSB 0
LB 0.00 Hz
GB 0
PC 1.00
RKS-5-215-F2
H
NH
HO
H
HO
CH3
H
7d
9
8
7
6
5
4
3
2
1
ppm
2.00
3.18
1.07
1.15
1.07
1.00
1.01
1.01
1.04
1.06
1.00
3.05
3.01
3.19

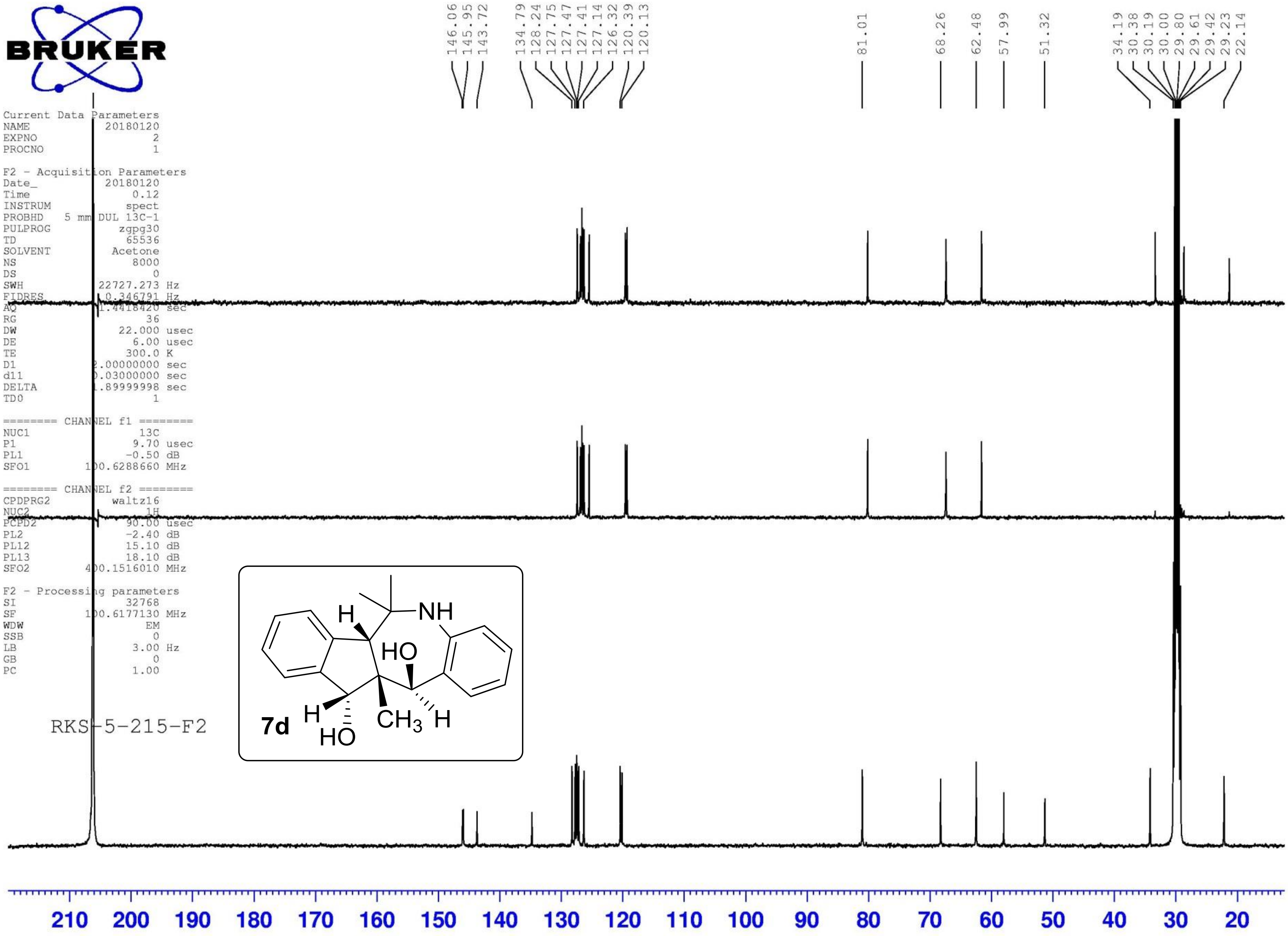
BRUKER
Current Data Parameters
NAME 20180120
EXPNO 2
PROCNO 1
F2 - Acquisition Parameters
Date_ 20180120
Time 0.12
INSTRUM spect
PROBHD 5 mm DUL 13C-1
PULPROG zgpg30
TD 65536
SOLVENT Acetone
NS 8000
DS 0
SWH 22727.273 Hz
FIDRES 0.346791 Hz
AQ 1.4418420 sec
RG 36
DW 22.000 usec
DE 6.00 usec
TE 300.0 K
D1 2.00000000 sec
d11 0.03000000 sec
DELTA 1.89999998 sec
TD0 1
======== CHANNEL f1 ========
NUC1 13C
P1 9.70 usec
PL1 -0.50 dB
SFO1 100.6288660 MHz
======== CHANNEL f2 ========
CPDPRG2 waltz16
NUC2 1H
PCPD2 90.00 usec
PL2 -2.40 dB
PL12 15.10 dB
PL13 18.10 dB
SFO2 400.1516010 MHz
F2 - Processing parameters
SI 32768
SF 100.6177130 MHz
WDW EM
SSB 0
LB 3.00 Hz
GB 0
PC 1.00
RKS-5-215-F2
146.06
145.95
143.72
134.79
128.24
127.75
127.47
127.41
127.14
126.32
120.39
120.13
81.01
68.26
62.48
57.99
51.32
34.19
30.38
30.19
30.00
29.80
29.61
29.42
29.23
22.14
NH
H
HO
H
CH3
H
HO
7d
210
200
190
180
170
160
150
140
130
120
110
100
90
80
70
60
50
40
30
20

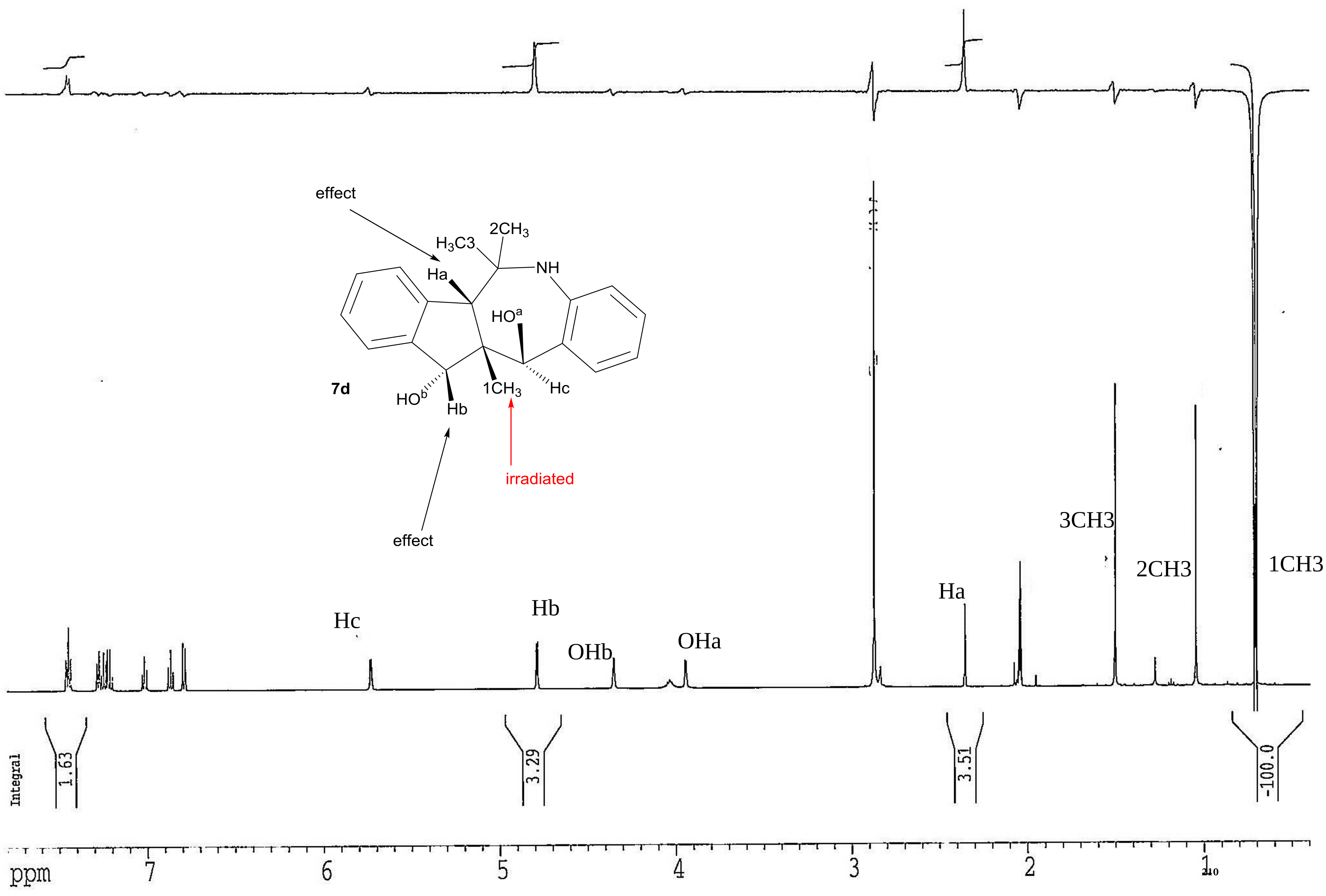

effect
2CH3
H3C3
NH
Ha
HOa
7d
1CH3
Hc
HOb
Hb
irradiated
effect
3CH3
2CH3
1CH3
Ha
Hb
Hc
OHb
OHa
Integral
1.63
3.29
3.51
-100.0
ppm
7
6
5
4
3
2
1

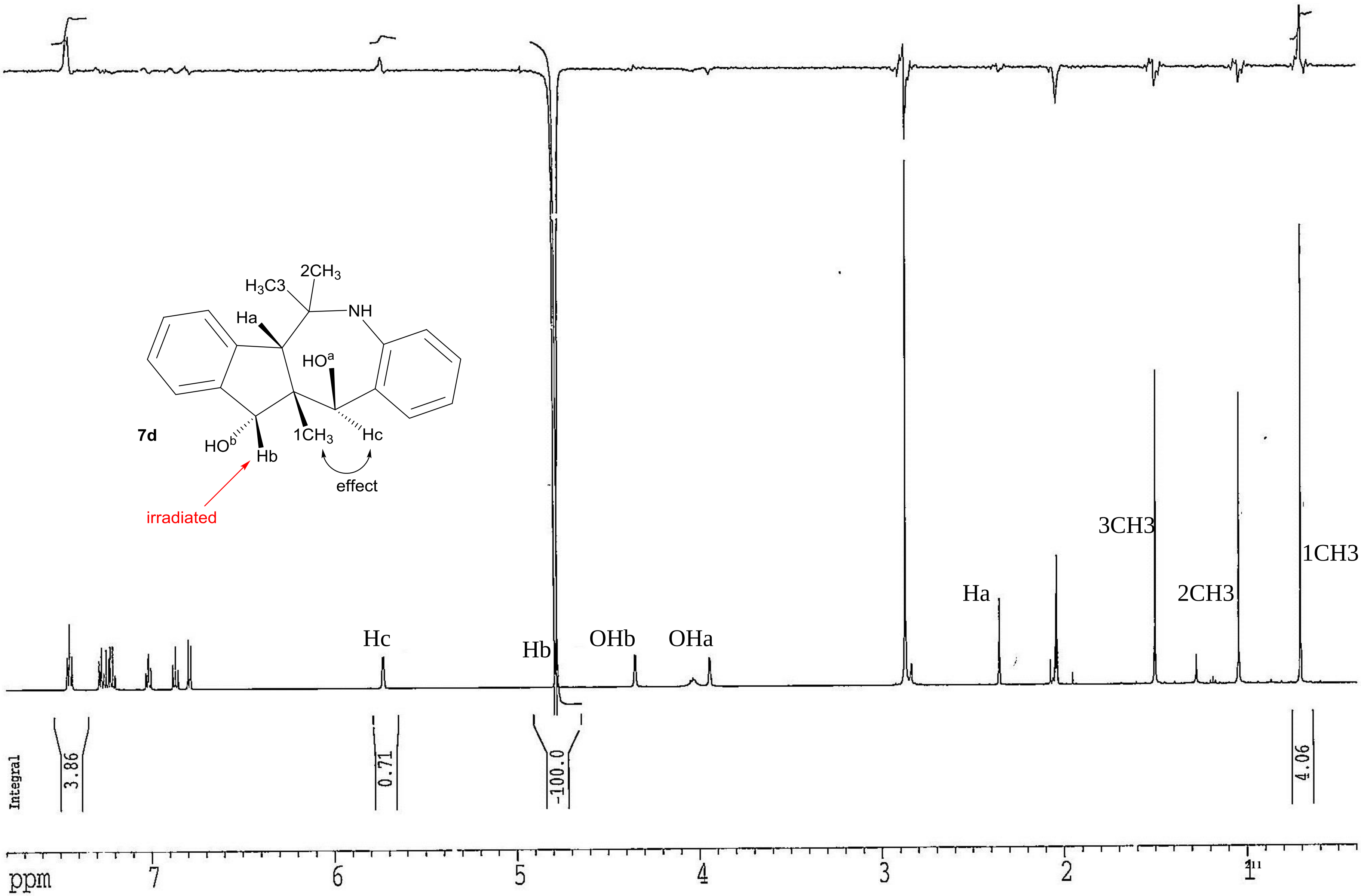

H3C3
2CH3
Ha
NH
HOa
7d
HOb
Hb
1CH3
Hc
effect
irradiated
Hc
Hb
OHb
OHa
Ha
3CH3
2CH3
1CH3
Integral
3.86
0.71
-100.0
4.06
ppm
7
6
5
4
3
2
1

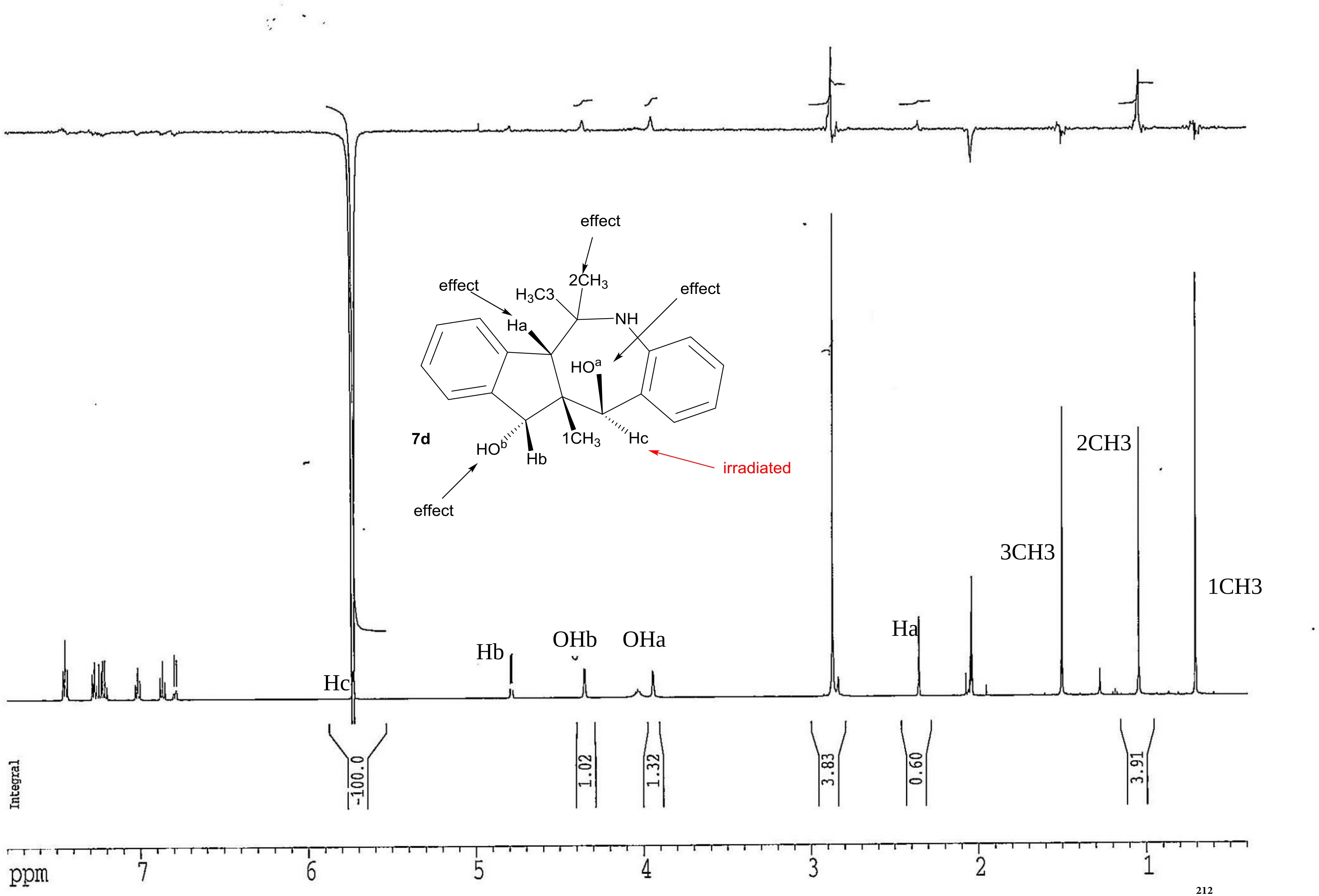
effect
effect
H_3C3
$2CH_3$
effect
Ha
NH
HO^a
7d
HO^b
Hb
$1CH_3$
Hc
irradiated
effect
2CH3
3CH3
1CH3
Ha
Hb
OHb
OHa
Hc
Integral
-100.0
1.02
1.32
3.83
0.60
3.91
ppm
7
6
5
4
3
2
1

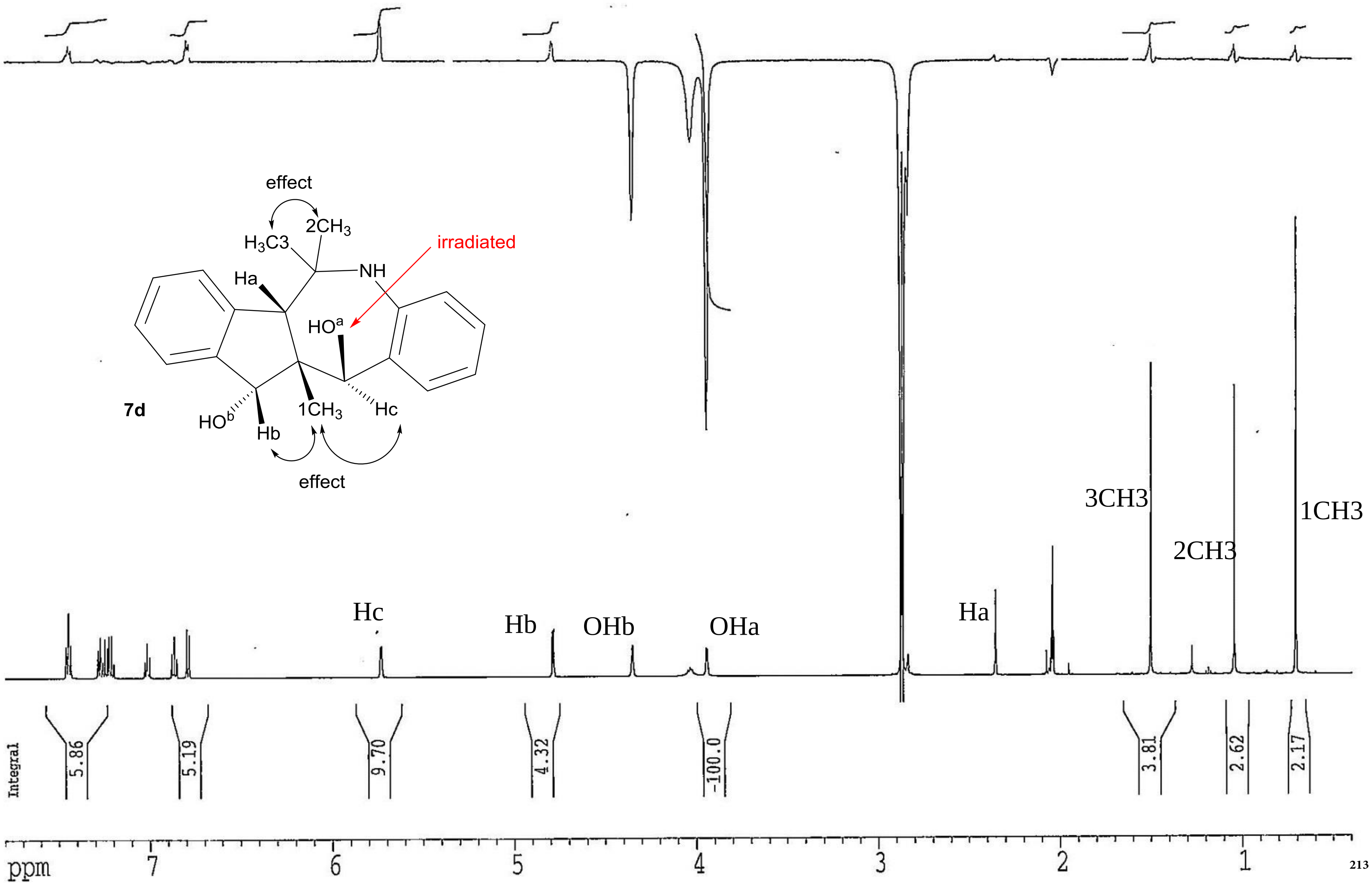

effect
2CH3
H3C3
irradiated
Ha
NH
HOa
7d
HOb
Hb
1CH3
Hc
effect
Hc
Hb
OHb
OHa
Ha
3CH3
2CH3
1CH3
Integral
5.86
5.19
9.70
4.32
-100.0
3.81
2.62
2.17
ppm
7
6
5
4
3
2
1

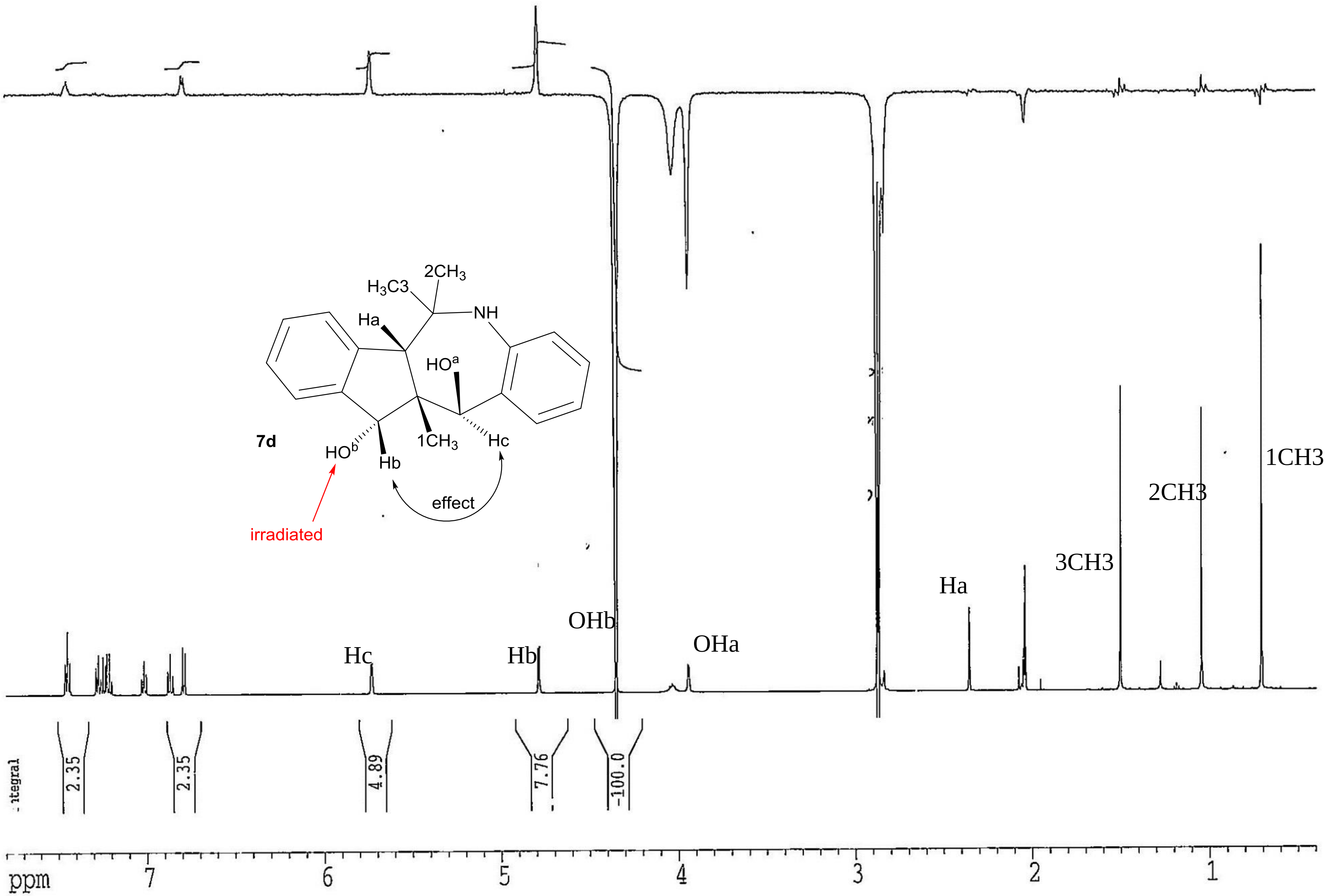
7d
H3C3
2CH3
NH
Ha
HOa
HOb
Hb
1CH3
Hc
effect
irradiated
Hc
Hb
OHb
OHa
Ha
3CH3
2CH3
1CH3
Integral
2.35
2.35
4.89
7.76
-100.0
ppm
7
6
5
4
3
2
1

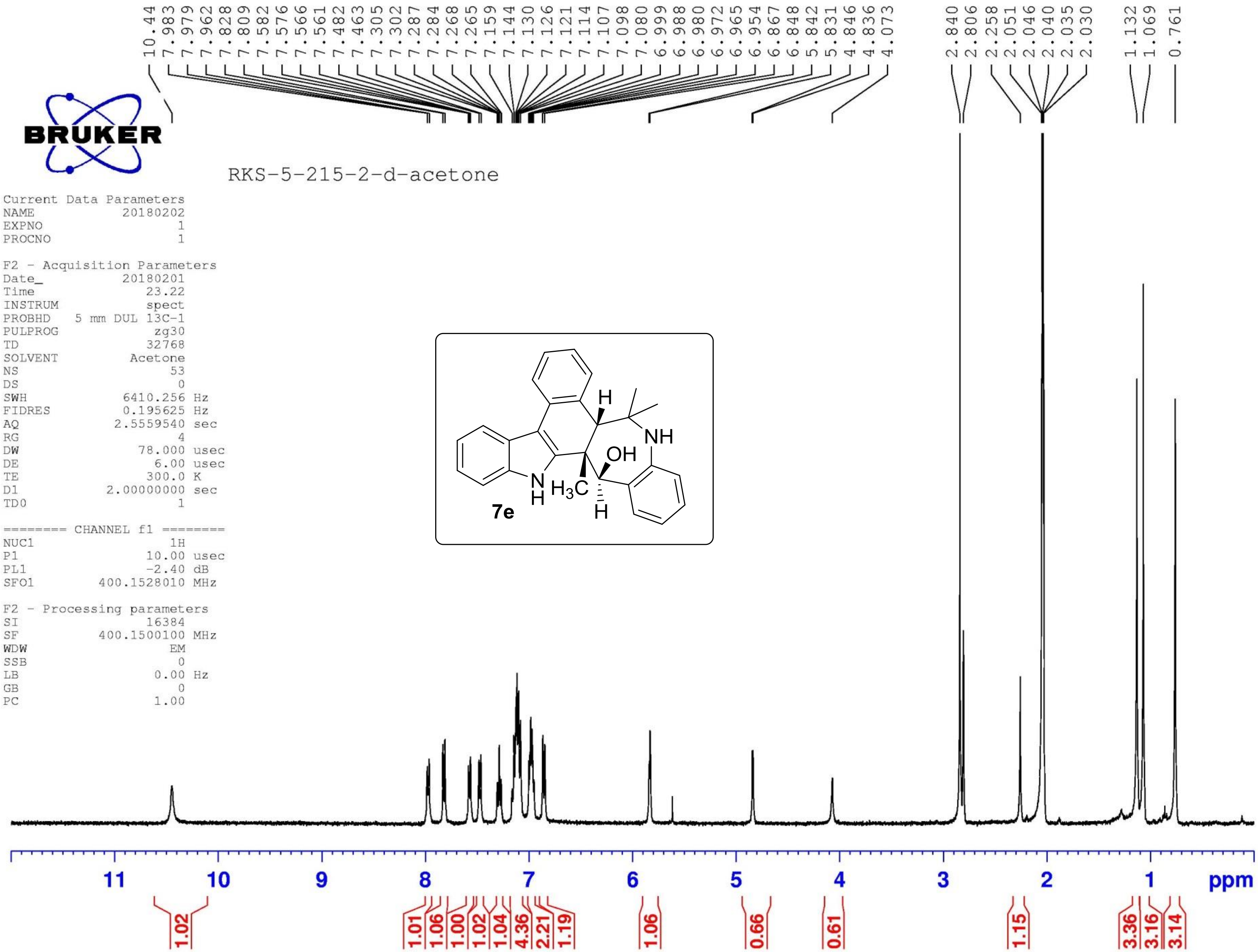
BRUKER
RKS-5-215-2-d-acetone
Current Data Parameters
NAME 20180202
EXPNO 1
PROCNO 1
F2 - Acquisition Parameters
Date_ 20180201
Time 23.22
INSTRUM spect
PROBHD 5 mm DUL 13C-1
PULPROG zg30
TD 32768
SOLVENT Acetone
NS 53
DS 0
SWH 6410.256 Hz
FIDRES 0.195625 Hz
AQ 2.5559540 sec
RG 4
DW 78.000 usec
DE 6.00 usec
TE 300.0 K
D1 2.00000000 sec
TD0 1
======== CHANNEL f1 ========
NUC1 1H
P1 10.00 usec
PL1 -2.40 dB
SFO1 400.1528010 MHz
F2 - Processing parameters
SI 16384
SF 400.1500100 MHz
WDW EM
SSB 0
LB 0.00 Hz
GB 0
PC 1.00
10.44 7.983 7.979 7.962 7.828 7.809 7.582 7.576 7.566 7.561 7.482 7.463 7.305 7.302 7.287 7.284 7.268 7.265 7.159 7.144 7.130 7.126 7.121 7.114 7.107 7.098 7.080 6.999 6.988 6.980 6.972 6.965 6.954 6.867 6.848 5.842 5.831 4.846 4.836 4.073
2.840 2.806 2.258 2.051 2.046 2.040 2.035 2.030
1.132 1.069 0.761
H
NH
OH
N
H
H3C
H
7e
11 10 9 8 7 6 5 4 3 2 1 ppm
1.02 1.01 1.06 1.00 1.02 1.04 4.36 2.21 1.19 1.06 0.66 0.61 1.15 3.36 3.16 3.14

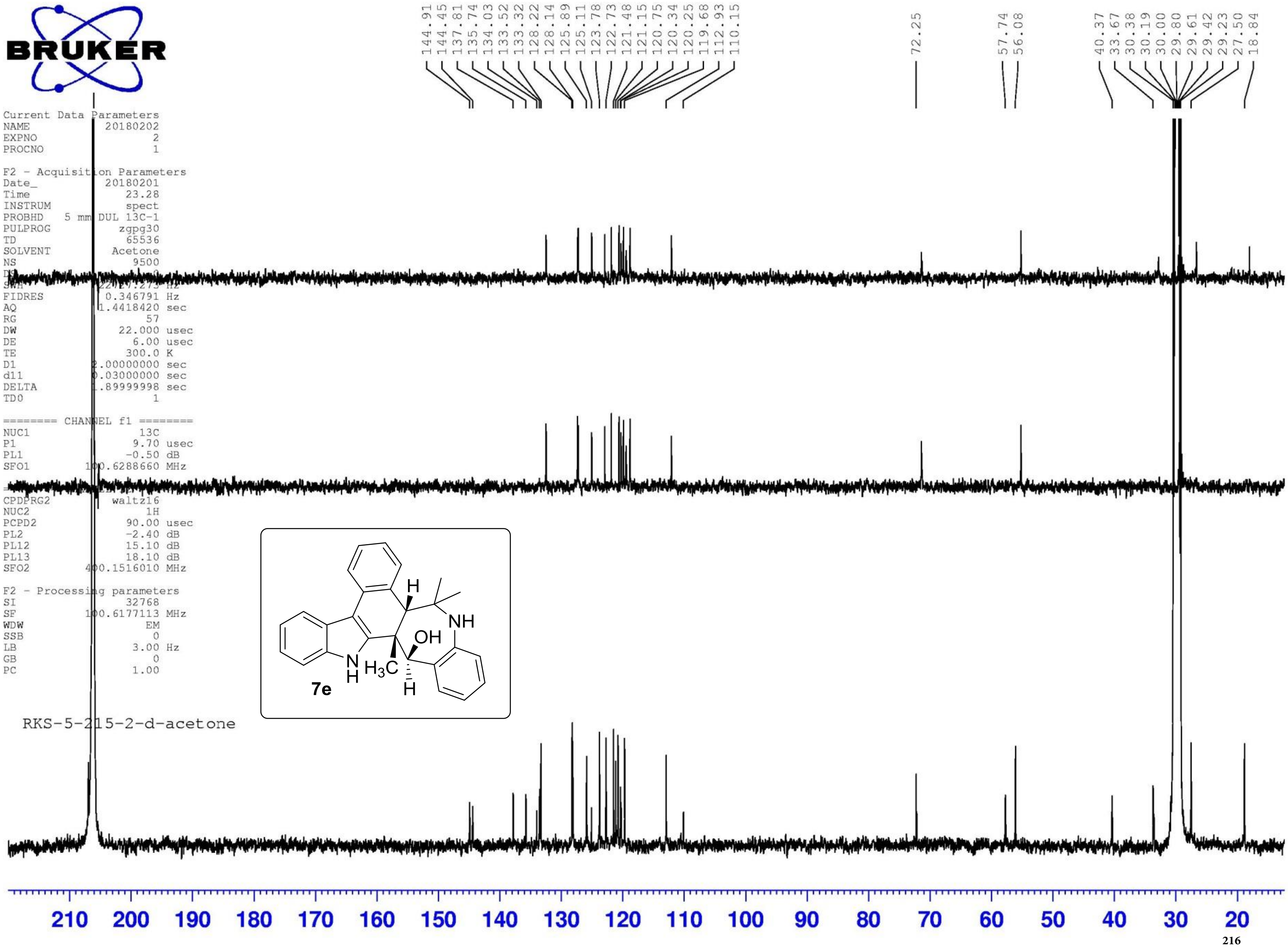
BRUKER
144.91
144.45
137.81
135.74
134.03
133.52
133.32
128.22
128.14
125.89
125.11
123.78
122.73
121.48
121.15
120.75
120.34
120.25
119.68
112.93
110.15
72.25
57.74
56.08
40.37
33.67
30.38
30.19
30.00
29.80
29.61
29.42
29.23
27.50
18.84
Current Data Parameters
NAME 20180202
EXPNO 2
PROCNO 1
F2 - Acquisition Parameters
Date_ 20180201
Time 23.28
INSTRUM spect
PROBHD 5 mm DUL 13C-1
PULPROG zgpg30
TD 65536
SOLVENT Acetone
NS 9500
FIDRES 0.346791 Hz
AQ 1.4418420 sec
RG 57
DW 22.000 usec
DE 6.00 usec
TE 300.0 K
D1 2.00000000 sec
d11 0.03000000 sec
DELTA 1.89999998 sec
TD0 1
======== CHANNEL f1 ========
NUC1 13C
P1 9.70 usec
PL1 -0.50 dB
SFO1 100.6288660 MHz
CPDPRG2 waltz16
NUC2 1H
PCPD2 90.00 usec
PL2 -2.40 dB
PL12 15.10 dB
PL13 18.10 dB
SFO2 400.1516010 MHz
F2 - Processing parameters
SI 32768
SF 100.6177113 MHz
WDW EM
SSB 0
LB 3.00 Hz
GB 0
PC 1.00
7e
RKS-5-215-2-d-acetone
210 200 190 180 170 160 150 140 130 120 110 100 90 80 70 60 50 40 30 20
